# Enantioselective electrochemical nickel-catalyzed vinylogous radical reactions

**DOI:** 10.1126/sciadv.adu5594

**Published:** 2025-03-19

**Authors:** Jiayin Zhang, Minghao Liu, Wenyuan Zhang, Chang Guo

**Affiliations:** Hefei National Research Center for Physical Sciences at the Microscale and Department of Chemistry, University of Science and Technology of China, Hefei, 230026, China.

## Abstract

Highly functionalized structural motifs with extended chiral carbon chains are prevalent in a wide range of bioactive compounds and play critical roles in the production of various functionalized molecules. Here, we describe a nickel-catalyzed asymmetric radical-based electrochemical functionalization of silyl polyenolates at α-, γ-, ε-, and η-positions. Driven by electric current, this methodology provides a sustainable route to access enantioenriched dicarbonyls via vinylogous radical pathways. It demonstrates excellent functional groups tolerance, mild reaction conditions, broad substrate compatibility, formation of quaternary stereocenters at remote positions, and high levels of regio- and enantioselectivity (up to 98% enantiomeric excess). Mechanistic investigations indicate that ferrocene-based electron transfer mediators are pivotal in the anodic oxidation process, facilitating the generation of nickel-bound α-carbonyl radicals while suppressing the undesired oxidation of silyl polyenolates, thus guiding the selection of mediators for electrocatalytic systems. The versatility of catalytic asymmetric electrosynthesis is highlighted by the preparation of valuable enantioenriched building blocks and the total synthesis of (-)-ethosuximide.

## Teaser

Nickel electrocatalysis allows for highly enantioselective access to dicarbonyl compounds via vinylogous radical reactions.

## INTRODUCTION

The asymmetric vinylogous reaction represents an effective method for producing highly functionalized optically pure bioactive compounds in an atom-efficient manner, allowing the introduction of stereocenters at remote functional group sites (*1*). The nucleophilic character of π-extended enolate-type chains facilitates the propagation of reactivity from α-site to distant γ-, ε-, and η-carbon positions through conjugated unsaturated bonds (*2*, *3*). Classic catalytic asymmetric processes, such as Aldol (*4*–*7*), Mannich (*8*–*10*), Michael (*11*), and substitution (*12*–*14*) reactions, can form vinylogous carbon-carbon and carbon-heteroatom bonds through two-electron ionic pathways. However, hurdles remain in enantioselective C(sp^3^)-H vinylogous functionalization of polyenolate species with nucleophiles, prompting the investigation of radical routes that can permit umpolung reactions and lower energy barriers via polarity inversion in catalytic intermediates (*15*–*17*).

**Fig. 1.**
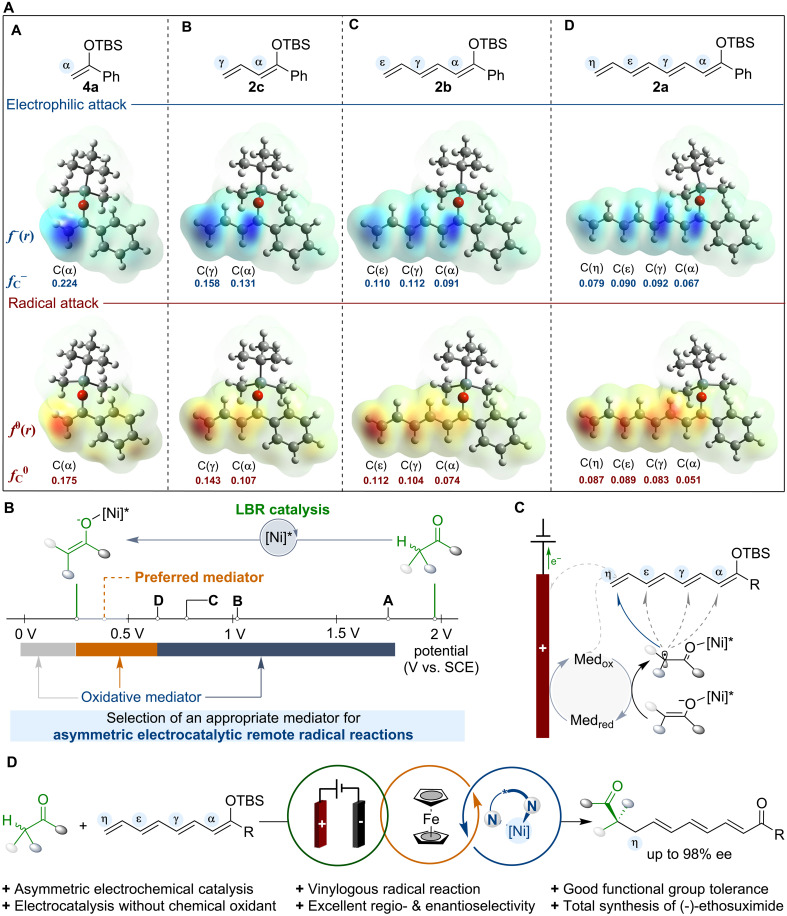
Concept and design of radical-based asymmetric electrochemical remote catalysis involving anodic oxidative processes. (**A**) Electrophilic and radical attack characteristics of vinylogous systems (**A** to **D**). *f*^ −^(*r*)/*f*_C_^−^: electrophilic attack, *f*^ 0^(*r*)/*f*_C_^0^: radical attack. (**B**) Evaluation of redox mediators to oxidize Lewis acid–bound enolate intermediates, generating LBR species and preventing oxidation of silyl polyenolates for asymmetric electrocatalysis. The onset oxidation potentials of **A**, **B**, **C**, and **D** are approximately +1.78, +1.04, +0.78, and +0.63 V, respectively (vide infra). (**C**) Remote radical addition with polyenolate species: Electrochemically generated electrophilic radicals are stereoselectively intercepted by silyl polyenolates. (**D**) This work features Cp_2_Fe-mediated asymmetric electrooxidative vinylogous radical addition of silyl polyenolates via nickel catalysis.

To tackle the demanding task of remote radical reactions, three challenges need to be addressed. First, regioselectivity poses a substantial obstacle. Initial density functional theory calculations revealed that Fukui functions [*f*^ −^(*r*)] for extended enolate derivatives showed the expected nucleophilic reactivity patterns (Fig. 1A, **A** to **D**). According to condensed Fukui functions (*f_C_*^−^), it is critical to address the less distinct ionic reactivity patterns of types **C** (α = 0.091, γ = 0.112, ε = 0.110) and **D** (α = 0.067, γ = 0.092, ε = 0.090, η = 0.079) in comparison to types **A** (α = 0.224) and **B** (α = 0.131, γ = 0.158). The calculation results also indicate that in vinylogous systems, as the length of the olefin chain increases, radical attacks are more favorable at the terminal position compared to ionic reactions [Fig. 1A, *f*^ 0^(*r*) and *f*_C_^0^]. In addition, the formation of a radical via terminal addition is stabilized by resonance with the adjacent π-system, which results in enhanced thermodynamic stability of the radical intermediate, thus making it a favorable site for radical reactions. Therefore, a radical-based strategy is proposed to tackle the regioselectivity issue by selectively targeting the terminal position of polyenolate species, leveraging the steric hindrance and conjugation effects of the allylic radical intermediate (*15*–*17*). This approach may resolve the challenging α/γ/ε/η regioselectivity dilemma for unbiased unbranched enols and enable previously difficult η-functionalizations of enols.

An additional challenge in remote radical reactions is achieving effective stereocontrol at sites that are distant from the chiral catalyst. The control of formation and stereochemical interception of short-lived, open-shell intermediates in vinylogous reactions continues to be a key difficulty, driving advancements in radical chemistry (*18**–**20*) and asymmetric catalysis (*21*, *22*). The combination of asymmetric aminocatalysis with the principle of vinylogy typically provides a powerful synthetic tool for generating a stereogenic center distant from the catalytic action site (*22*, *23*). However, the capability of the chiral link in the vinylogous system to sequester the radical intermediate and convey stereochemical information remotely to the in situ formed stereocenter presents challenges. Electrosynthesis has emerged as a highly efficient, sustainable, and versatile platform for producing radical intermediates, eliminating the requirement for stoichiometric amounts of oxidants or reductants (*24*–*33*). The Sibi group (*34*–*38*) pioneered the development of chiral Lewis acid–catalyzed radical reactions for the enantioselective addition to α,β-unsaturated carbonyl substrates. Recently, Lewis acid–bound radical (LBR)–based catalysis has demonstrated its effectiveness in achieving stereocontrol in a broad spectrum of electrochemical reactions, offering dependable routes for diverse bond formations during electrolysis (*39*–*45*). Building on advancements in asymmetric electrosynthesis (*46*–*48*), we believe that the implementation of electrocatalytic methods could enable radical-based pathways for remote functionalization with high stereoselectivity (*49*–*66*). LBR catalysis effectively reduces the oxidation potential of carbonyl substrates, facilitating electrocatalysis and minimizing competing racemic reactions (Fig. 1B).

The reactivity of remote radical reactions poses the third challenge. Asymmetric organic electrosynthesis encounters problems including substantial overoxidation, electrode passivation, and undesirable side reactions near the electrode surface due to concentration effects. Thus, redox mediators in electrosynthesis offer an ideal alternative for producing reactive species at the electrode surface, enabling interactions with substrates via single electron transfer (SET) and providing a feasible pathway for indirect electrolysis (Fig. 1C) (*67*–*70*). As the number of alkenyl chains increases, oxidation potentials gradually decrease from **A** to **D** with considerable variances. Consequently, introducing substituents with various electronic properties permits the selection of appropriate electrochemical mediators to generate distinct radical intermediates (*71*), providing a reliable strategy for developing flexible asymmetric remote radical reactions. Notably, the electrochemical formation of chiral nickel-bound α-keto radicals offers a promising opportunity to enhance enantioselectivity in remote radical attacks with diverse silyl polyenolates, addressing notable gaps in reactivity and facilitating the synthesis of structurally unique chiral dicarbonyls (*46*–*48*).

Here, we demonstrate that ferrocene-mediated chiral Lewis acid electrocatalysis enables enantioselective vinylogous radical reactions, using extended enolate derivatives at α-, γ-, ε-, and η-positions (Fig. 1D). The chiral Lewis acid operates in both the electrochemical step and asymmetric induction, proving versatile with a variety of coupling partners under mild electrochemical conditions. The influence of the redox mediator on electrochemical vinylogous radical reactions has prompted mechanistic investigations, with promising implications for developing mediators in challenging electrocatalytic methodologies. The developed method provides high regio- and enantioselectivity, offering an alternative pathway for the enantioselective total synthesis of (-)-ethosuximide.

## RESULTS

### Reaction development

To validate our hypothesis, we initiated the reaction using racemic benzoxazolyl acetate (**1a**) and silyl tetraenol ether (**2a**) in an undivided cell with a constant current of 2.0 mA at room temperature to identify the optimal chiral ligand (Table 1). Under electrolytic conditions, using a tridentate pyridine bisoxazoline (PyBox) ligand (**L1**), an indane-fused Box ligand (**L2**), or a benzyl-substituted Box ligand (**L3**) resulted in low enantiomeric excess (ee) of the desired product **3a** for accessing umpolung reactivity (*72*–*74*). The ee of **3a** increased to 93% with the use of Box ligand **L4**, featuring a phenyl group. Further optimization with Box ligands bearing varied substituents on the bridging carbon (**L5** to **L10**) revealed that **L10**, with a cyclopentyl group, delivered **3a** in 78% yield and 96% ee (entry 1). Control experiments were carried out to elucidate the process of electricity-driven vinylogous radical reactions. The absence of ferrocene (Cp_2_Fe, entry 2), electric current (entry 3), chiral ligand **L10** (entry 4), or Lewis acid (entry 5) completely inhibited the reaction, thus ruling out competing racemic processes. The introduction of *^n^*Bu_4_NPF_6_ led to 67% yield without affecting enantioselectivity (entry 6). Using carbon rod (C) as the anodic material resulted in decreased yield while maintaining ee (entry 7).

**Table 1.**
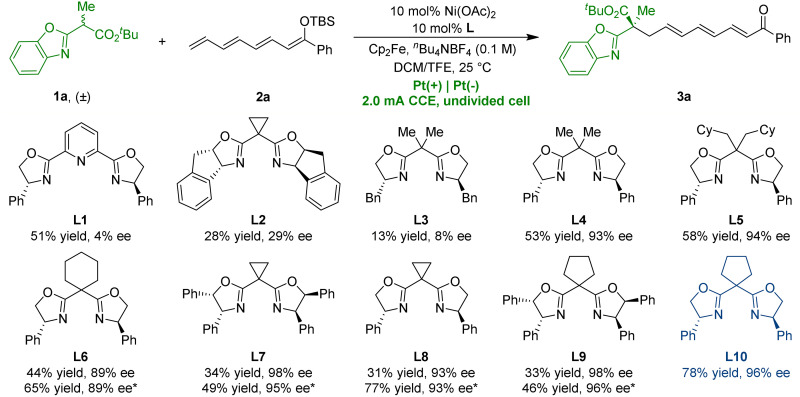
Screening of reaction conditions. Unless otherwise specified, all reactions were carried out using **1a** (0.1 mmol, 1.0 equiv), **2a** (0.3 mmol, 3.0 equiv), Ni(OAc)_2_ (10 mol %), **L** (10 mol %), Cp_2_Fe (10 mol %), *^n^*Bu_4_NBF_4_ (0.1 M), and DCM/TFE = 2:1 (3.0 ml) at 25°C under constant current conditions in an undivided cell. ^*^DCM/TFE = 1:1 (3.0 ml). Enantiomeric excess was analyzed by chiral HPLC. TFE, 2,2,2-trifluoroethanol; nd, not detected; CCE, Constant current electrolysis.

Entry	Variation from the standard conditions with L10 as the ligand	Results
**1**	**None**	**78% yield, 96% ee**
2	In the absence of Cp_2_Fe	nd
3	In the absence of electric current	nd
4	In the absence of **L10**	nd
5	In the absence of Ni(OAc)_2_	nd
6	*^n^*Bu_4_NPF_6_ instead of *^n^*Bu_4_NBF_4_	67% yield, 96% ee
7	Carbon rod as the anode	46% yield, 96% ee

### Mechanistic considerations

**Fig. 2.**
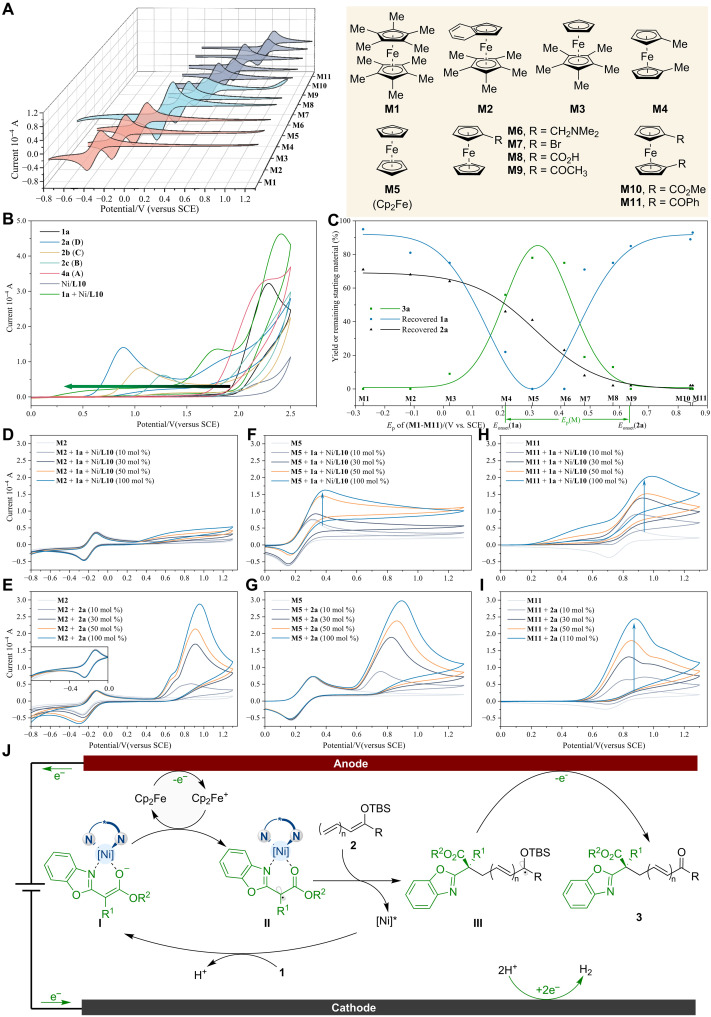
Mechanistic investigation. (**A**) Evaluation of redox potentials for mediators. (**B**) CV analysis of relevant compounds in the catalytic system. (**C**) Assessment of ferrocenyl mediators in electrocatalysis. (**D**) Titration of Ni/**L10**-**1a** with **M2**. (**E**) Titration of **2a** with **M2**. (**F**) Titration of Ni/**L10**-**1a** with **M5**. (**G**) Titration of **2a** with **M5**. (**H**) Titration of Ni/**L10**-**1a** with **M11**. (**I**) Titration of **2a** with **M11**. (**J**) Proposed mediator-assisted asymmetric electrocatalytic cycle.

Given the vital role of ferrocene as an electrochemical mediator in the reaction (*75*), extensive mechanistic studies were conducted to clarify its function (Fig. 2). Initially, cyclic voltammetry (CV) was used to systematically investigate the redox potentials of ferrocene derivatives within the relevant range, revealing reversible redox chemistry and stable redox states (*70*, *71*). Notably, electron-donating substituents on the ferrocene decreased the oxidation potential, while electron-withdrawing substituents increased this parameter (Fig. 2A).

Following the introduction of the chiral nickel catalyst, the onset oxidative potential of benzoxazolyl acetate **1a** underwent a decrease from approximately +1.95 to +0.21 V (versus SCE), indicating the ease of oxidation of the Ni-bound enolate species (Ni/**L10**-**1a**) under standard conditions (Fig. 2B). The onset oxidation potential for **2a** was determined to be approximately +0.63 V (versus SCE). Consequently, a series of ferrocenyl mediators with peak oxidation potentials ranging from −0.27 to +0.85 V (**M1**-**M11**, versus SCE) was evaluated by altering substituents to assess their effects on asymmetric vinylogous radical processes.

A comprehensive study revealed a bell-shaped curve relationship in reaction yields with mediators of varying oxidation potentials (Fig. 2C) (*76*). Reactions with low-potential mediators (type **I**: **M1** to **M3**), which were insufficient for oxidizing nickel enolate complexes (Ni/**L10**-**1a**) and **2a**, resulted in low product yields. This suggests that the oxidation process was inhibited, as evidenced by the substantial recovery of **1a** and **2a** after the reaction. When conducted with mediators having slightly higher anodic potentials (type **II**: **M4** to **M8**), which were suitable for Ni-bound enolate oxidation but inadequate for the oxidation of **2a**, moderate to good yields of product **3a** were achieved. However, increasing the mediator oxidation potential (type **III**: **M9** to **M11**) resulted in low yields, with a substantial residue of **1a** and an almost complete disappearance of **2a**. These results indicate that mediators with potentials exceeding +0.64 V (versus SCE) promote the competitive oxidation of excess **2a** (*E*_onset_ = +0.63V) in the presence of catalytic Ni/**L10**-**1a**. This also implies that the asymmetric vinylogous reaction does not occur through the direct oxidation of **2a** to form a radical cation intermediate.

To better understand the underlying process, electron transfer in the reaction system was further investigated by monitoring CV during titrating Ni/**L10**-**1a** or **2a** with a ferrocenyl mediator. Using **M2** as a representative type **I** mediator, the concentration of the nickel catalyst showed no effect on the redox behavior of **M2** (Fig. 2D). Gradually increasing the concentration of substrate **2a** led to an enhancement in the reduction peak intensity of **M2** (refer to Fig. 2E), suggesting an electron transfer between the radical cation of **2a** and **M2**. Restricting the scan range (from −0.8 to 0 V) with increasing **2a** concentration did not enhance the reduction peak intensity of **M2**, providing further support for this conclusion. Therefore, the anodically generated cationic ferrocenium (**M2**^+^) does not directly oxidize **2a** as an oxidation mediator, which is consistent with the considerable amount of residual **2a** observed when **M2** is used as the mediator.

When **M5** was used as a representative type **II** mediator, the oxidation peak intensity increased notably, while the reduction peak intensity declined progressively as the nickel complex concentration increased (Fig. 2F). The addition of **2a** to **M5** resulted in no catalytic current, indicating minimal electron exchange between **2a** and ferrocenium of **M5** (Fig. 2G). Given the effectiveness of type **II** mediators in facilitating asymmetric vinylogous reactions, mediators within this potential range primarily interact with the nickel-bound enolate intermediate and are insufficiently reactive to directly oxidize **2a**.

Further utilization of **M11** as a type **III** mediator results in a considerable catalytic current for both the nickel complex and **2a** (Fig. 2, H and I). The substantial remaining amount of **1a** and the complete consumption of **2a** under these conditions suggest that **2a** interferes with the electron transfer between the ferrocenium of **M11** and the nickel enolate species, thereby reducing the production of α-keto radicals that are necessary for vinylogous reactions. Collectively, these findings provide insights into the pathways by which the α-keto radical intermediates, generated anodically and indirectly via oxidation, contribute to the formation of the final product **3**.

On the basis of the comprehensive mechanistic insights, a plausible catalytic mechanism involving electricity-triggered LBR catalysis is proposed in Fig. 2J. Asymmetric electrochemical catalysis initiates with the condensation of benzoxazolyl acetate (**1**) with a nickel catalyst, resulting in nickel-bound enolate intermediate (**I**) that is preferentially oxidized over other components. Crucially, the oxidative potential of the mediator (Cp_2_Fe) falls between that of the nickel-bound enolate complex (**I**) and substrate **2**. Consequently, the anodically formed Cp_2_Fe^+^ reacts with nickel-bound enolate intermediate (**I**) via SET to generate LBR intermediate (**II**), which subsequently reacts with electron-rich polyenolate **2** to form ketyl radical species (**III**). Last, a second anodic-electron oxidation event produces the corresponding product **3** and regenerates the nickel catalyst.

### Substrate scope

**Fig. 3.**
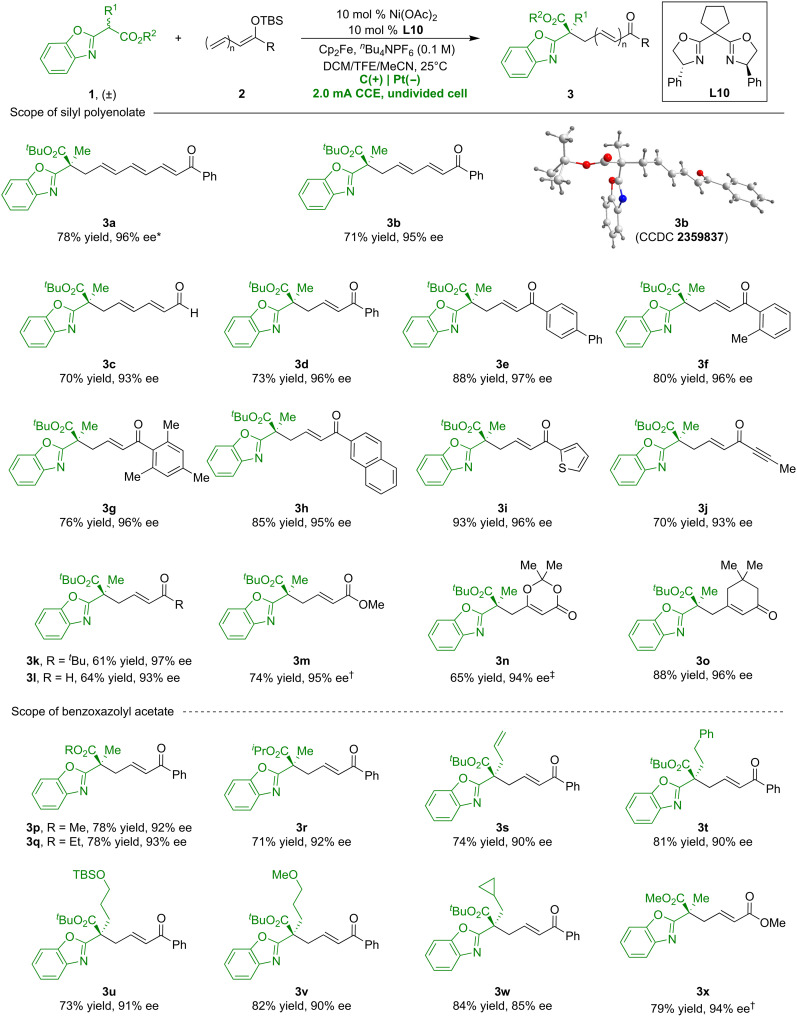
Substrate scope of vinylogous radical reactions. Unless otherwise specified, all reactions were carried out using racemic **1** (0.1 mmol), **2** (0.3 mmol), *^n^*Bu_4_NPF_6_ (0.3 mmol), Ni(OAc)_2_ (10 mol %), **L10** (10 mol %), Cp_2_Fe (10 mol %), and dichloromethane (DCM)/2,2,2-trifluoroethanol (TFE)/acetonitrile (MeCN) = 1.0:1.5:0.5 (3 ml) at 25°C in an undivided cell. ^*^Pt(+)|Pt(−), *^n^*Bu_4_NBF_4_ (0.3 mmol), DCM/TFE = 2:1 (3 ml). ^†^Pt(+)|Pt(−), **L9** (10 mol %), tetrahydrofuran (THF)/TFE = 1:1 (3 ml). ^‡^**L7** (10 mol %), DCM/methanol/MeCN = 1:1:1 (3 ml). CCE, Constant current electrolysis.

With the optimal reaction conditions established, we investigated the versatility of this enantioselective vinylogous radical reaction (Fig. 3). Our methodology efficiently generates η- (**3a**) and ε- (**3b**) derivatives from extended double bond systems, despite reduced electron density at remote sites. The absolute configuration of **3b** was confirmed via x-ray diffraction, and the others were assigned analogously. Aldehyde-derived trienols also proved effective, affording product in 70% yield and 93% ee (**3c**). Ketone-derived dienols with different functional groups led to γ-substituted products (**3d**-**3k**). Under standard conditions, silyloxydiene produced α,β-unsaturated aldehyde **3l** with 64% yield and 93% ee. Enols derived from methyl crotonate, dioxinone, and cyclic ketones were also suitable substrates (**3m**-**3o**). Further investigation with substituted racemic benzoxazolyl acetates **1** revealed that variations in the ester group had a little effect on the reaction outcomes (**3p**-**3r**). Various α-substituents including allylic (**3s**) and alkyl (**3t**-**3x**) groups were well tolerated, consistently delivering high yields and enantioselectivities. These results demonstrate the broad substrate applicability of the current electrocatalyzed vinylogous radical reaction.

**Fig. 4.**
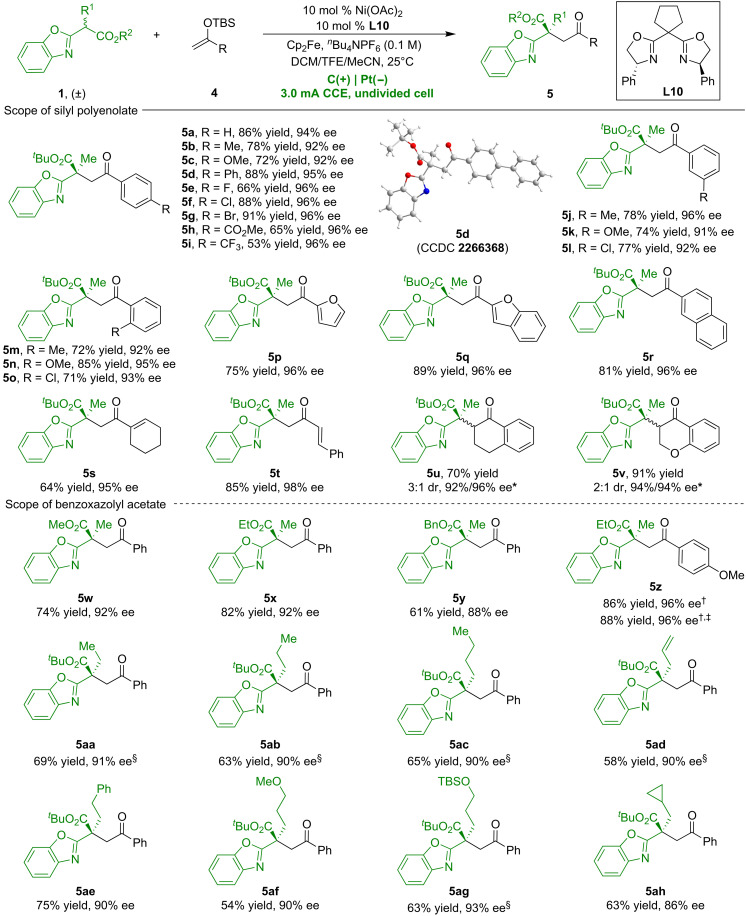
Nickel-catalyzed asymmetric electrochemical alkylation. Unless otherwise specified, all reactions were carried out using racemic **1** (0.1 mmol), **4** (0.3 mmol), *^n^*Bu_4_NPF_6_ (0.3 mmol), Ni(OAc)_2_ (10 mol %), **L10** (10 mol %), Cp_2_Fe (10 mol %), and DCM/TFE/MeCN = 1.0:1.5:0.5 (3 ml) at 25°C in an undivided cell. ^*^Pt(+)|Pt(−), THF/TFE = 1:2 (3 ml). ^†^**L9** (10 mol %). ^‡^1 mmol scale. ^§^At 10°C.

To further illustrate the versatility of this nickel-catalyzed electrochemical process, we explored its application in asymmetric alkylation (Fig. 4). Using the Ni/**L10** complex, the catalytic electrochemical interaction between **1a** and silyl enol ether **4a** successfully generated α-substituted product **5a** with 86% yield and 94% ee. Subsequent exploration into the generality of this nickel-catalyzed asymmetric alkylation revealed that diverse silyl enol ethers **4** varying functional groups are excellent coupling partners, reliably delivering desirable products with high yields and enantioselectivities (**5a**-**5t**). Excitingly, enol silyl ethers derived from cyclic ketones can also achieve good yields and enantioselectivity, albeit with limited diastereoselectivity (**5u** and **5v**). X-ray diffraction analysis confirmed the *S* configuration of **5d**. Evaluation of racemic benzoxazolyl acetates **1** confirmed their compatibility with the reaction conditions, leading to a range of alkylated products (**5w**-**5ah**) with high enantioselectivities. Furthermore, the electrocatalytic alkylation approach demonstrated its practical utility for larger-scale applications by effectively scaling the reaction to 1 mmol, consistently producing product **5z** with a high yield and excellent enantioselectivity.

### Synthetic applications

**Fig. 5.**
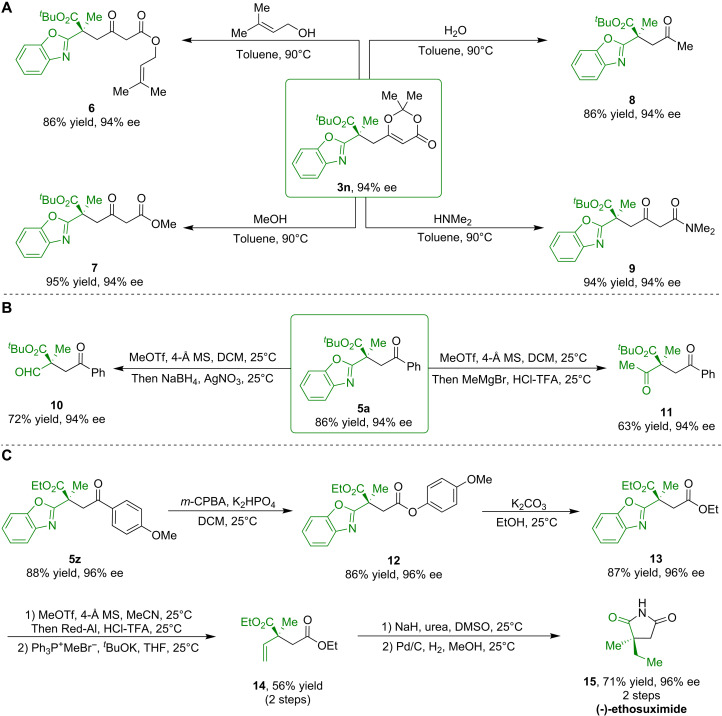
Synthetic utility and asymmetric total synthesis. (**A**) Versatile functionalization of product **3n**. (**B**) Derivatization of enantioenriched product **5a**. (**C**) Asymmetric total synthesis of (-)-ethosuximide. DMSO, dimethyl sulfoxide; MeOH, methanol; MeOTf, Methyl trifluoromethanesulfonate; MS, Molecular sieves.

To illustrate the synthetic utility of this methodology, we used the chiral dioxinone derivative **3n** in a variety of transformations to produce enantioenriched building blocks such as esters (**6** and **7**), ketone (**8**), and amide (**9**) in high yields with maintained enantioselectivity (Fig. 5A). Moreover, the benzoxazole moiety in enantioenriched product **5a** was readily converted to an aldehyde group in **10** and a ketone group in **11**, facilitating the construction of structurally diverse molecules with all-carbon quaternary stereocenters (Fig. 5B).

Our enantioselective methodology was further validated through the asymmetric total synthesis of (-)-ethosuximide (Fig. 5C). The enantioenriched ketone **5z** underwent Baeyer-Villiger oxidation and transesterification to produce the corresponding ester **13**. The removal of the benzoxazole moiety led to an aldehyde, which underwent a Wittig reaction to afford an alkene (**14**) in 56% yield over two steps. Treatment of compound **14** with urea resulted in the formation of a cyclic imide, which was then hydrogenated in the presence of a Pd/C catalyst to produce the desired (-)-ethosuximide (**15**) without any loss of enantiopurity.

## DISCUSSION

We have established highly electrochemical nickel-catalyzed vinylogous radical reactions at remote sites with exceptional regio- and enantioselectivity. The reaction features a broad substrate scope, mild electrochemical reaction conditions, and excellent functional group tolerance. Detailed studies support the key role of Cp_2_Fe as a mediator facilitating the oxidation of nickel-bound enolate complexes while protecting polyenolate substrates from undesired side reactions. The applicability of this method was further demonstrated by the manufacture of valuable enantiomerically enriched building blocks and the total synthesis of (-)-ethosuximide. We anticipate that this work on Cp_2_Fe-assisted Lewis acid electrocatalysis will inspire future efforts in combining asymmetric catalysis with electrosynthesis, particularly in addressing challenging asymmetric radical transformations.

## MATERIALS AND METHODS

### General information

Unless otherwise noted, materials were obtained from commercial suppliers and used without further purification. The instrument for electrolysis is dual display potentiostat (DJS-292B) (made in China). Flash chromatography was performed using silica gel (SiliaFlash P60, 230-400 mesh) from SiliCycle. The electrochemical reactions were performed in an undivided cell equipped with a platinum plate (1.0 cm by 1.0 cm by 0.2 mm) or a carbon rod (*d* = 6 mm) as an anode and a platinum plate (1.0 cm by 1.0 cm by 0.2 mm) as a cathode. All reactions were carried out in flame-dried glassware under a dry nitrogen atmosphere. Proton nuclear magnetic resonance (^1^H NMR) spectra and carbon nuclear magnetic resonance (^13^C NMR) spectra were recorded at 25°C on Bruker Advance 400 MHz NMR spectrometers or Bruker Advance 500 MHz NMR spectrometers. High-resolution mass spectral analysis was performed on Waters XEVO G2 Q-TOF. Optical rotations were determined at 589 nm (sodium D line) by using a PerkinElmer 343 polarimeter. The measurement of enantiomeric excesses was performed on Waters-Alliance (2998, Photodiode Array Detector).

### General procedure for asymmetric electrochemical vinylogous radical reaction

A 10-ml reaction tube was charged with racemic benzoxazolyl acetate **1** (0.1 mmol, 1.0 equiv), silyl enol ether **2** or **4** (0.3 mmol, 3.0 equiv), nickel complex derived from (*R*,*R*)-**L10** (0.01 mmol, 0.1 equiv), Cp_2_Fe (0.01 mmol, 0.1 equiv), *^n^*Bu_4_NPF_6_ (0.1 M), dichloromethane (DCM; 1 ml), acetonitrile (MeCN; 0.5 ml), and 2,2,2-trifluoroethanol (TFE; 1.5 ml) under an argon atmosphere. The reaction tube was equipped with a carbon rod (diameter = 6 mm) as the anode and a platinum plate (1.0 cm by 1.0 cm by 0.2 mm) as the cathode. Constant current electrolysis was carried out at 25°C until complete consumption of the substrate, monitored by thin-layer chromatography. The mixture was concentrated under reduced pressure and purified by flash column chromatography on silica gel to afford the desired product **3** or **5**.
