# Supplementary material for: Enantioselective electrochemical nickel-catalyzed vinylogous radical reactions
Source: Sci Adv. 2025 Mar 19;11(12):eadu5594. doi: 10.1126/sciadv.adu5594 (PMC11922052; doi:10.1126/sciadv.adu5594)
Supplement: Supplementary file 1 — Supplementary Text Figs. S1 to S23 Tables S1 to S10 [file sciadv.adu5594_sm.pdf]

Science Advances
AAAS

Supplementary Materials for

Enantioselective electrochemical nickel-catalyzed vinylogous radical reactions

Jiayin Zhang *et al.*

Corresponding author: Chang Guo, guochang@ustc.edu.cn

Sci. Adv. **11**, eadu5594 (2025)
DOI: 10.1126/sciadv.adu5594

This PDF file includes:

Supplementary Text
Figs. S1 to S23
Tables S1 to S10

1. General information

Unless otherwise noted, all reagents were purchased from commercial suppliers and used without further purification. Flash chromatography was performed using silica gel (SiliaFlash P60, 230-400 mesh) from SiliCycle. The electrochemical reactions were performed in an undivided cell equipped with a platinum plate (1.0 cm × 1.0 cm × 0.2 mm) or carbon rod (d = 6 mm) as anode and platinum plate (1.0 cm × 1.0 cm × 0.2 mm) as a cathode. All reactions were carried out in flame-dried glassware under a dry nitrogen atmosphere. TLC visualization was performed under a UV lamp. Cyclic voltammograms were recorded on a CHI 760E potentiostat. Proton nuclear magnetic resonance (^{1}H NMR) spectra and carbon nuclear magnetic resonance (^{13}C NMR) spectra were recorded at 25 ℃ on Bruker Advance 400 MHz NMR spectrometers or Bruker Advance 500 MHz NMR spectrometers. Chemical shifts for ^{1}H NMR spectra are reported as δ in units of parts per million (ppm) downfield from $SiMe_4$ (δ 0.00) and relative to the signal of chloroform-*d* (δ 7.26, singlet). Multiplicities were given as: s (singlet); d (doublet); t (triplet); q (quartet); dd (doublet of doublets); dt (doublet of triplets); m (multiplets). Coupling constants are reported as a *J* value in Hz. ^{13}C NMR spectra are reported as δ in units of parts per million (ppm) downfield from $SiMe_4$ (δ 0.00) and relative to the signal of chloroform-*d* (δ 77.16, triplet). High-resolution mass spectral analysis (HRMS) was performed on Waters XEVO G2 Q-TOF. Optical rotations were determined at 589 nm (sodium D line) by using a Perkin-Elmer-343 polarimeter. The relative and absolute configurations of **3b** and **5d** were assigned by the X-ray analysis. The measurement of enantiomeric excesses was performed on Waters-Alliance (2998, Photodiode Array Detector). CHIRALPAK IA, IC, IE, IF, IG, AD-H, AS-H, and CHIRALCEL OD-H columns were purchased from Daicel Chemical Industries, LTD.

2. Photographic guide for electrochemical reactions

2.1 Overview of materials used

From left to right: 1) Electrochemical cell; 2) platinum plate (1.0 cm × 1.0 cm × 0.2 mm) anode or carbon rod (d = 6 mm) anode and platinum plate (1.0 cm × 1.0 cm × 0.2 mm) cathode, purchased from Tianjin AIDAhengsheng Science-Technology Development Co., Ltd.

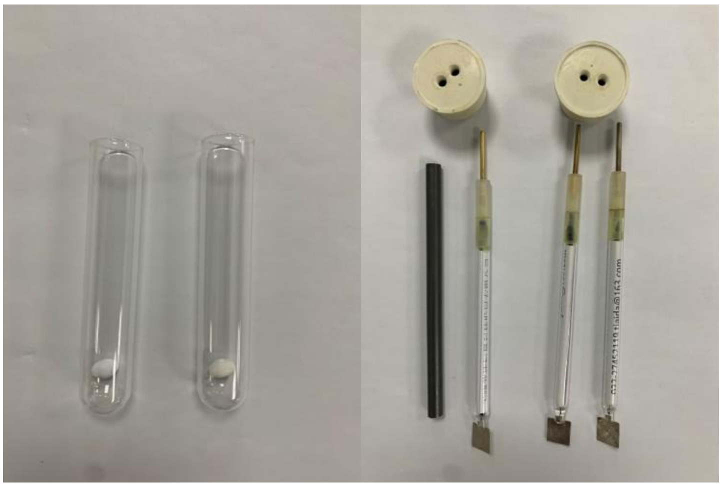

2.2 Assembling the cell

The distance from the anode to the cathode is 5 mm

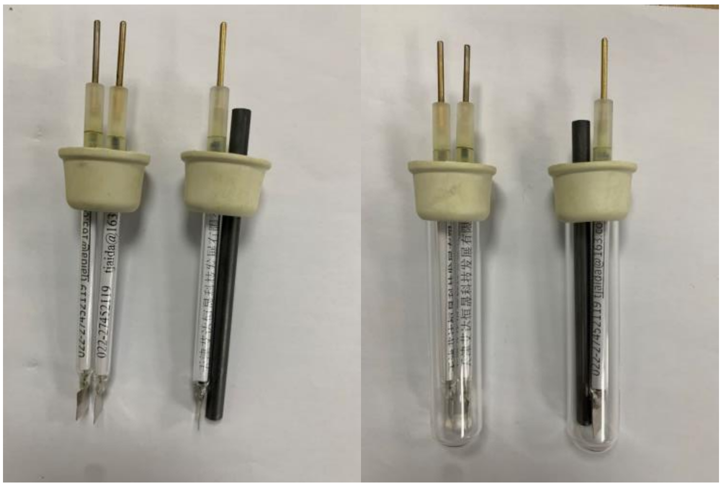

2.3 Electrolysis

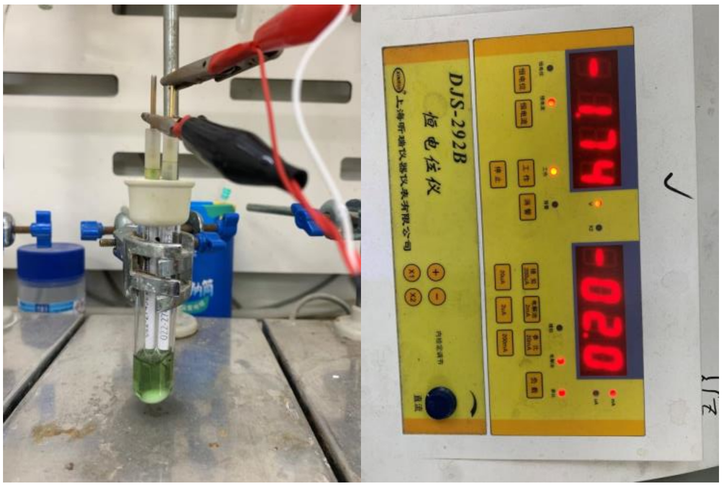

3. Optimization studies

Table S1: Side reaction detection

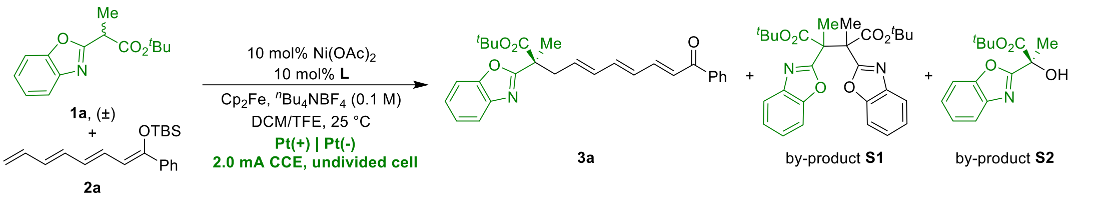

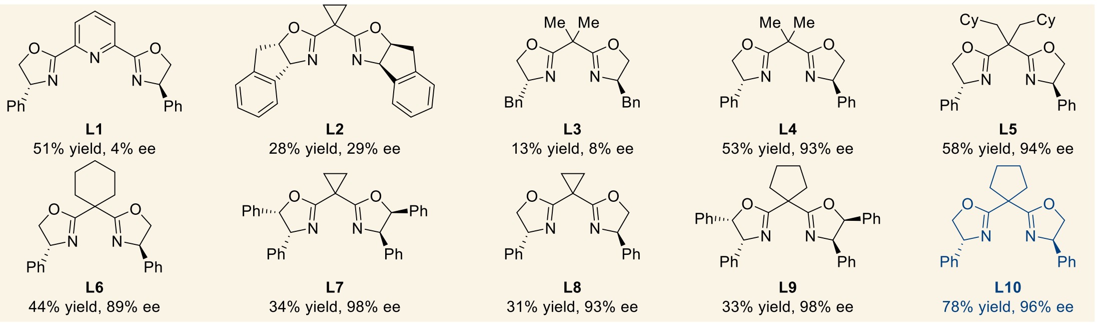

entry	L	3a	S1	S2
1	**L6**	44% yield, 89% ee	11% yield	13% yield
2	**L8**	31% yield, 93% ee	14% yield	15% yield
3	**L9**	33% yield, 98% ee	15% yield	10% yield

Unless otherwise specified, all reactions were carried out using **1a** (0.1 mmol, 1.0 equiv), **2a** (0.3 mmol, 3.0 equiv), $Ni(OAc)_2$ (10 mol%), **L** (10 mol%), Cp_2Fe (10 mol%), $^nBu_4NBF_4$ (0.1 M), and DCM/TFE = 2:1 (3.0 mL) at 25 ℃ under constant-current conditions in an undivided cell.

Table S2: Optimization of the reaction conditions for 3a

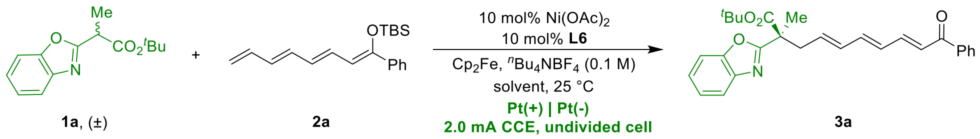

entry	electrode	solvent	results
1	Pt(+)|Pt(-)	DCM/TFE = 2:1	44% yield, 89% ee
**2**	**Pt(+)|Pt(-)**	**DCM/TFE = 1.5:1.5**	**65% yield, 89% ee**
3	Pt(+)|Pt(-)	THF/TFE = 1.5:1.5	trace
4	Pt(+)|Pt(-)	THF/TFE = 1:2	trace
5	Pt(+)|Pt(-)	acetone/TFE = 2:1	trace
6	Pt(+)|Pt(-)	EtOAc/TFE = 2:1	trace
7	Pt(+)|Pt(-)	DCM/THF/TFE = 1:1:1	39% yield, 88% ee
8	Pt(+)|Pt(-)	DCM/$CHCl_3$/TFE = 1:1:1	51% yield, 80% ee
9	Pt(+)|Pt(-)	DCM/$CHCl_3$/TFE = 1.5:0.5:1	54% yield, 81% ee
10	Pt(+)|Pt(-)	DCM/$CHCl_3$/TFE = 1:0.5:1.5	37% yield, 82% ee
11	Pt(+)|Pt(-)	DCM/MeOH/TFE = 1:1:1	trace
12	Pt(+)|Pt(-)	DCM/$CHCl_3$/TFE = 1.75:0.25:1	45% yield, 84% ee

Unless otherwise specified, all reactions were carried out using **1a** (0.1 mmol, 1.0 equiv), **2a** (0.3 mmol, 3.0 equiv), $Ni(OAc)_2$ (10 mol%), **L6** (10 mol%), Cp_2Fe (10 mol%), $^nBu_4NBF_4$ (0.1 M), solvent (3.0 mL) at 25 ℃ under constant-current conditions in an undivided cell.

Table S3: Further optimization of the reaction conditions for 3a

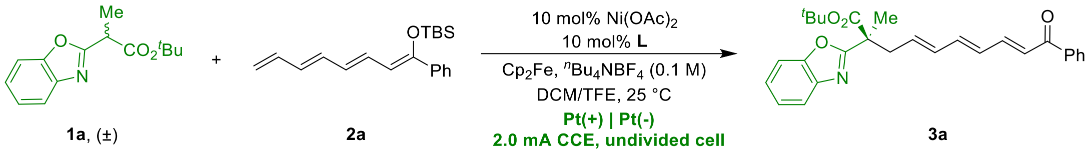

entry	L	yield (%)	ee (%)
1	**L6**	65	89
2	**L7**	49	95
3	**L8**	77	93
4	**L9**	46	96

Unless otherwise specified, all reactions were carried out using **1a** (0.1 mmol, 1.0 equiv), **2a** (0.3 mmol, 3.0 equiv), $Ni(OAc)_2$ (10 mol%), **L** (10 mol%), Cp_2Fe (10 mol%), $^{n}Bu_4NBF_4$ (0.1 M), DCM/TFE = 1:1 (3.0 mL) at 25 ℃ under constant-current conditions in an undivided cell.

Table S4: Solvent effect

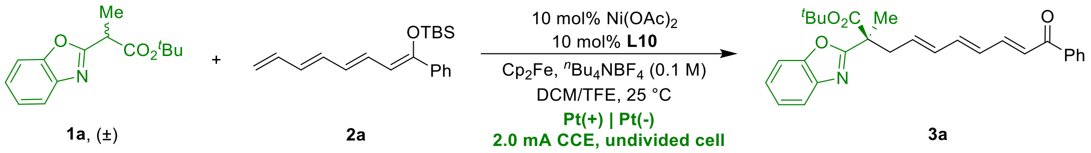

entry	variation from the standard conditions	results
1	none	78% yield, 96% ee
2	add 0.5 mL of MeCN	65% yield, 96% ee

Unless otherwise specified, all reactions were carried out using **1a** (0.1 mmol, 1.0 equiv), **2a** (0.3 mmol, 3.0 equiv), $Ni(OAc)_2$ (10 mol%), **L10** (10 mol%), Cp_2Fe (10 mol%), $^{n}Bu_4NBF_4$ (0.1 M), DCM/TFE = 2:1 (3.0 mL) at 25 ℃ under constant-current conditions in an undivided cell.

Table S5: Optimization of the reaction conditions for 3b

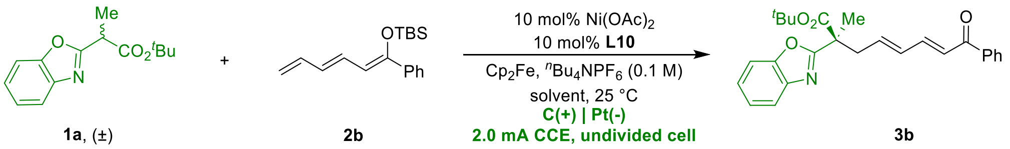

entry	electrode	solvent	results
1	Pt(+)|Pt(-)	DCM/TFE = 2:1	41% yield, 92% ee
2	C(+)|Pt(-)	DCM/TFE = 2:1	45% yield, 95% ee
3	C(+)|Pt(-)	DCM/TFE/THF = 1:1.5:0.5	47% yield, 94% ee
4	C(+)|Pt(-)	DCM/TFE/EtOAc = 1:1.5:0.5	54% yield, 95% ee
5	C(+)|Pt(-)	DCM/TFE/MeCN = 1:1.5:0.5	71% yield, 95% ee

Unless otherwise specified, all reactions were carried out using **1a** (0.1 mmol, 1.0 equiv), **2a** (0.3 mmol, 3.0 equiv), $Ni(OAc)_2$ (10 mol%), **L10** (10 mol%), Cp_2Fe (10 mol%), $^{n}Bu_4NPF_6$ (0.1 M), solvent (3.0 mL) at 25 ℃ under constant-current conditions in an undivided cell.

Table S6: Optimization of the reaction conditions for 3m

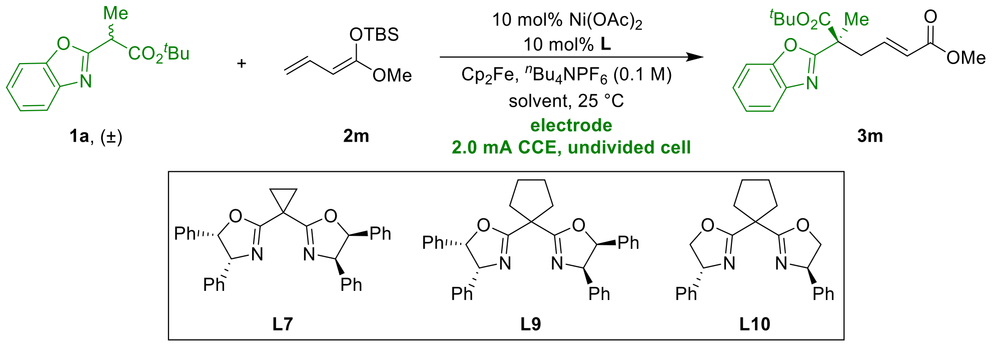

entry	L	electrode	solvent	results
1	**L7**	C(+)|Pt(-)	DCM/TFE/MeCN = 1:1.5:0.5	9% yield, 96% ee
2	**L9**	C(+)|Pt(-)	DCM/TFE/MeCN = 1:1.5:0.5	15% yield, 95% ee
3	**L10**	C(+)|Pt(-)	DCM/TFE/MeCN = 1:1.5:0.5	16% yield, 89% ee
4	**L9**	Pt(+)|Pt(-)	DCM/TFE = 1.5:1.5	20% yield, 97% ee
**5**	**L9**	**Pt(+)|Pt(-)**	**THF/TFE = 1.5:1.5**	**74% yield, 95% ee**

Unless otherwise specified, all reactions were carried out using **1a** (0.1 mmol, 1.0 equiv), **2** (0.3 mmol, 3.0 equiv), $Ni(OAc)_2$ (10 mol%), **L** (10 mol%), Cp_2Fe (10 mol%), $^{n}Bu_4NPF_6$ (0.1 M), solvent (3.0 mL) at 25 ℃ under constant-current conditions in an undivided cell.

Table S7: Optimization of the reaction conditions for 5u

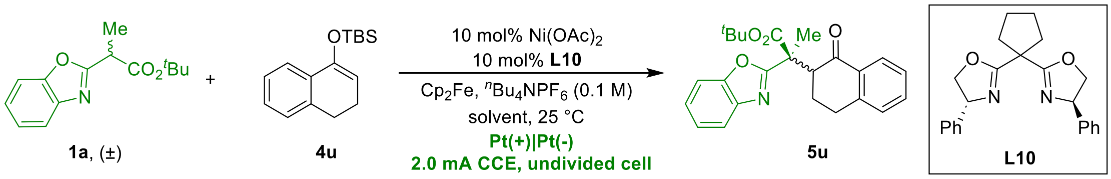

entry	solvent	yield (%)	dr	ee (%)
1	DCM/TFE = 1.5:1.5	22	2:1	88/89
2	THF/TFE = 1.5:1.5	46	3:1	88/90
3	THF/TFE = 2:1	31	4:1	85/86
4	THF/TFE/DCM = 1:1:1	49	3:1	94/90
**5**	**THF/TFE = 1:2**	**70**	**3:1**	**92/96**

Unless otherwise specified, all reactions were carried out using **1a** (0.1 mmol, 1.0 equiv), **2** (0.3 mmol, 3.0 equiv), $Ni(OAc)_2$ (10 mol%), **L** (10 mol%), Cp_2Fe (10 mol%), $^{n}Bu_4NPF_6$ (0.1 M), solvent (3.0 mL) at 25 ℃ under constant-current conditions in an undivided cell.

4. Synthesis and characterization of products

The nickel catalyst was synthesized according to a previously reported method. $Ni(OAc)_2 \cdot 4H_2O$ (1 mmol) and ligand (1 mmol) were mixed in acetonitrile (3 mL) and menthol (3 mL), and the mixture was stirred at room temperature for 4 h. After removing the solvent, the residue was dissolved in dichloromethane and evaporated to dryness. The resulting preformed nickel complex can be used directly for the subsequent reaction. Benzoxazolyl acetates containing different ester groups and silyl enol ethers were synthesized according to the reported literature.

Procedure for nickel-catalyzed asymmetric electrochemical alkylation

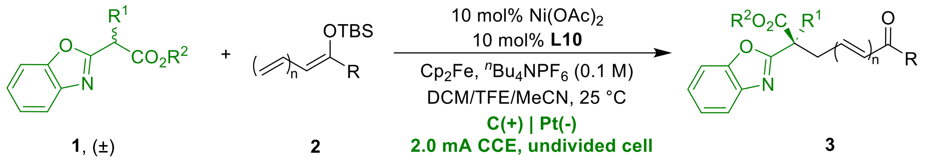

A 10 mL reaction tube was charged with racemic benzoxazolyl acetate **1** (0.1 mmol, 1.0 equiv), silyl enol ether **2** (0.3 mmol, 3.0 equiv), nickel complex derived from (*R*,*R*)-**L10** (0.01 mmol, 0.1 equiv), Cp_2Fe (0.01 mmol, 0.1 equiv), $^nBu_4NPF_6$ (0.1 M), DCM (1 mL), MeCN (0.5 mL), and TFE (1.5 mL) under argon atmosphere. The reaction tube was equipped with a carbon rod (d = 6 mm) as anode and a platinum plate (1.0 cm × 1.0 cm × 0.2 mm) as cathode. The constant current (2.0 mA) electrolysis was carried out at 25 °C until complete consumption of the substrate (monitored by TLC). The mixture was treated with trifluoroacetic acid (20 μL) and stirred for 10 minutes. After then the mixture was concentrated under reduced pressure, and purified by flash column chromatography on silica gel to afford the desired product **3**.

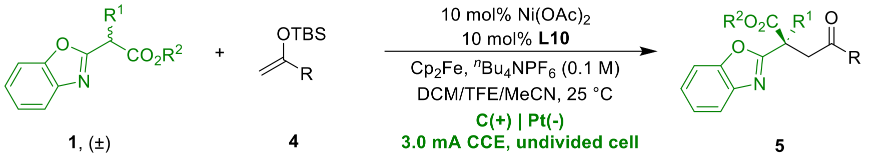

A 10 mL reaction tube was charged with racemic benzoxazolyl acetate **1** (0.1 mmol, 1.0 equiv), silyl enol ether **4** (0.3 mmol, 3.0 equiv), nickel complex derived from (*R*,*R*)-**L10** (0.01 mmol, 0.1 equiv), Cp_2Fe (0.01 mmol, 0.1 equiv), $^nBu_4NPF_6$ (0.1 M), DCM (1 mL), MeCN (0.5 mL), and TFE (1.5 mL) under argon atmosphere. The reaction tube was equipped with a carbon rod (d = 6 mm) as the anode and a platinum plate (1.0 cm × 1.0 cm × 0.2 mm) as the cathode. The constant current (3.0 mA) electrolysis was carried out at 25 °C until complete consumption of the substrate (monitored by TLC). The mixture was concentrated under reduced pressure, and purified by flash column chromatography on silica gel to afford the desired product **5**.

***tert*-Butyl (*S*,4*E*,6*E*,8*E*)-2-(benzo[*d*]oxazol-2-yl)-2-methyl-10-oxo-10-phenyldeca-4,6,8-trienoate (3a)**

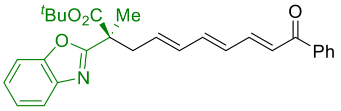

A 10 mL reaction tube was charged with racemic **1a** (0.1 mmol, 1 equiv), silyl enol ether **2a** (0.3 mmol, 3 equiv), nickel complex derived from (*R*,*R*)-**L10** (0.01 mmol, 0.1 equiv), Cp_2Fe (0.01 mmol, 0.1 equiv), $^nBu_4NBF_4$ (0.1 M), DCM (2 mL), and TFE (1.0 mL) under argon atmosphere. The reaction tube was equipped with a platinum plate (1.0 cm × 1.0 cm × 0.2 mm) as the anode and a platinum plate (1.0 cm × 1.0 cm × 0.2 mm) as the cathode. The constant current (2.0 mA) electrolysis was carried out at 25 °C for 5 h until complete consumption of the substrate (monitored by TLC). The mixture was treated with trifluoroacetic acid (20 μL) and stirred for 10 minutes. After then the mixture was concentrated under

reduced pressure, and purified by flash column chromatography on silica gel to afford the desired product **3a** (34.6mg, 78%). **^{1}H NMR (400 MHz, CDCl$_3$)** δ 7.97 – 7.90 (m, 2H), 7.77 – 7.70 (m, 1H), 7.59 – 7.51 (m, 2H), 7.50 – 7.40 (m, 3H), 7.37 – 7.31 (m, 2H), 6.96 (d, *J* = 15.0 Hz, 1H), 6.60 (dd, *J* = 14.8, 10.8 Hz, 1H), 6.37 (dd, *J* = 14.8, 11.4 Hz, 1H), 6.29 (dd, *J* = 15.0, 10.9 Hz, 1H), 5.92 (dt, *J* = 15.1, 7.6 Hz, 1H), 3.10 – 2.97 (m, 2H), 1.69 (s, 3H), 1.39 (s, 9H). **^{13}C NMR (100 MHz, CDCl$_3$)** δ 190.6, 170.9, 166.9, 151.0, 144.8, 141.7, 140.9, 138.3, 134.2, 133.8, 132.7, 130.2, 128.7, 128.5, 125.2, 125.1, 124.4, 120.2, 110.7, 82.5, 49.7, 40.6, 28.0, 21.5. **ESI-MS:** calculated $[C_{28}H_{29}NO_4 + Na]^+$: 466.1989, found: 466.1987. $[\alpha]^{20}_D$ = +53.9 (c = 1.01, CH_2Cl_2). The product was analyzed by HPLC to determine the enantiomeric excess: 96% ee (CHIRALPAK IC, *n*-hexane/*i*-PrOH = 90/10, detector: 320 nm, T = 25 °C, flow rate: 1 mL/min), t_1 (major) = 25.89 min, t_2 (minor) = 28.24 min.

***tert*-Butyl (*S*,4*E*,6*E*)-2-(benzo[*d*]oxazol-2-yl)-2-methyl-8-oxo-8-phenylocta-4,6-dienoate (3b)**

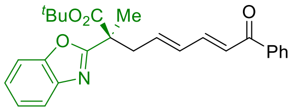

Reaction time: 2 h. **^{1}H NMR (400 MHz, CDCl$_3$)** δ 7.95 – 7.87 (m, 2H), 7.78 – 7.70 (m, 1H), 7.58 – 7.50 (m, 2H), 7.49 – 7.43 (m, 2H), 7.38 – 7.30 (m, 3H), 6.88 (d, *J* = 15.1 Hz, 1H), 6.41 (dd, *J* = 15.0, 11.1 Hz, 1H), 6.21 (dt, *J* = 15.0, 7.5 Hz, 1H), 3.13 – 3.02 (m, 2H), 1.71 (s, 3H), 1.40 (s, 9H). **^{13}C NMR (100 MHz, CDCl$_3$)** δ 190.9, 170.8, 166.7, 151.0, 144.3, 140.9, 139.1, 138.1, 133.1, 132.8, 128.7, 128.5, 125.2, 125.1, 124.5, 120.2, 110.7, 82.6, 49.5, 40.7, 28.0, 21.5. **ESI-MS:** calculated $[C_{26}H_{27}NO_4 + Na]^+$: 440.1832, found: 440.1832. $[\alpha]^{20}_D$ = +51.2 (c = 0.99, CH_2Cl_2). The product was analyzed by HPLC to determine the enantiomeric excess: 95% ee (CHIRALPAK IE, *n*-hexane/*i*-PrOH = 90/10, detector: 270 nm, T = 25 °C, flow rate: 1 mL/min), t_1 (minor) = 26.92 min, t_2 (major) = 31.18 min.

***tert*-Butyl (*S*,4*E*,6*E*)-2-(benzo[*d*]oxazol-2-yl)-2-methyl-8-oxoocta-4,6-dienoate (3c)**

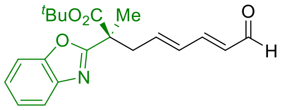

Reaction time: 2 h. **^{1}H NMR (400 MHz, CDCl$_3$)** δ 9.52 (d, *J* = 8.0 Hz, 1H), 7.78 – 7.69 (m, 1H), 7.57 – 7.50 (m, 1H), 7.41 – 7.31 (m, 2H), 7.04 (dd, *J* = 15.3, 10.7 Hz, 1H), 6.41 (dd, *J* = 15.1, 10.8 Hz, 1H), 6.25 (dt, *J* = 15.1, 7.5 Hz, 1H), 6.08 (dd, *J* = 15.3, 8.0 Hz, 1H), 3.15 – 3.00 (m, 2H), 1.71 (s, 3H), 1.40 (s, 9H). **^{13}C NMR (100 MHz, CDCl$_3$)** δ 194.0, 170.7, 166.6, 151.6, 151.0, 140.9, 140.1, 132.5, 131.3, 125.3, 124.5, 120.2, 110.7, 82.7, 49.6, 40.7, 28.0, 21.6. **ESI-MS:** calculated $[C_{20}H_{23}NO_4 + Na]^+$: 364.1519, found: 364.1520. $[\alpha]^{20}_D$ = +64.3 (c = 0.68, CH_2Cl_2). The product was analyzed by HPLC to determine the enantiomeric excess: 93% ee (CHIRALPAK AD-H, *n*-hexane/*i*-PrOH = 90/10, detector: 248 nm, T = 25 °C, flow rate: 1 mL/min), t_1 (minor) = 5.71 min, t_2 (major) = 6.38 min.

***tert*-Butyl (*S*,*E*)-2-(benzo[*d*]oxazol-2-yl)-2-methyl-6-oxo-6-phenylhex-4-enoate (3d)**

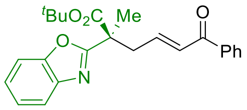

Reaction time: 2 h. **^{1}H NMR (400 MHz, CDCl$_3$)** δ 7.85 – 7.79 (m, 2H), 7.76 – 7.71 (m, 1H), 7.56 – 7.50 (m, 2H), 7.45 – 7.39 (m, 2H), 7.37 – 7.31 (m, 2H), 7.03 – 6.88 (m, 2H), 3.23 – 3.11 (m, 2H), 1.76 (s, 3H), 1.40 (s, 9H). **^{13}C NMR (100 MHz, CDCl$_3$)** δ 190.5, 170.7, 166.5, 151.0, 143.1, 140.9, 137.7, 132.9, 129.9, 128.7, 128.7, 125.2, 124.5, 120.3, 110.7, 82.8, 49.5, 40.2, 27.9, 21.6. **ESI-MS:** calculated $[C_{24}H_{25}NO_4 + Na]^+$: 414.1676, found: 414.1688. $[\alpha]^{20}_D$ = +2.3 (c = 0.9, CH_2Cl_2). The product was analyzed by HPLC to determine the enantiomeric excess: 96% ee (CHIRALPAK IC, *n*-hexane/*i*-PrOH = 95/5, detector: 254 nm, T = 25 °C, flow rate: 1 mL/min), t_1 (minor) = 14.07 min, t_2 (major) = 16.75 min.

tert-Butyl (S,E)-6-([1,1'-biphenyl]-4-yl)-2-(benzo[d]oxazol-2-yl)-2-methyl-6-oxohex-4-enoate (3e)

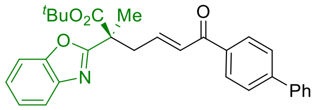

Reaction time: 2 h. **^{1}H NMR (400 MHz, CDCl$_3$)** δ 7.96 – 7.88 (m, 2H), 7.77 – 7.72 (m, 1H), 7.67 – 7.59 (m, 4H), 7.56 – 7.51 (m, 1H), 7.50 – 7.44 (m, 2H), 7.43 – 7.38 (m, 1H), 7.37 – 7.32 (m, 2H), 7.06 – 6.94 (m, 2H), 3.27 – 3.12 (m, 2H), 1.78 (s, 3H), 1.41 (s, 9H). **^{13}C NMR (100 MHz, CDCl$_3$)** δ 189.9, 170.7, 166.5, 151.0, 145.6, 143.0, 140.9, 140.0, 136.3, 129.8, 129.3, 129.1, 128.3, 127.4, 127.3, 125.2, 124.5, 120.3, 110.7, 82.8, 49.5, 40.3, 28.0, 21.6. **ESI-MS:** calculated [$C_{30}H_{29}NO_4$ + Na]$^+$: 490.1989, found: 490.1988. $[\alpha]^{20}_{D}$ = +37.4 (c = 1.03, CH_2Cl_2). The product was analyzed by HPLC to determine the enantiomeric excess: 97% ee (CHIRALPAK IC, *n*-hexane/*i*-PrOH = 90/10, detector: 254 nm, T = 25 °C, flow rate: 1 mL/min), t_1 (minor) = 15.40 min, t_2 (major) = 17.12 min.

tert-Butyl (S,E)-2-(benzo[d]oxazol-2-yl)-2-methyl-6-oxo-6-(o-tolyl)hex-4-enoate (3f)

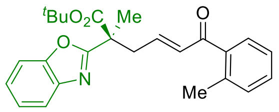

Reaction time: 2 h. **^{1}H NMR (400 MHz, CDCl$_3$)** δ 7.75 – 7.70 (m, 1H), 7.53 – 7.48 (m, 1H), 7.37 – 7.32 (m, 2H), 7.32 – 7.26 (m, 2H), 7.22 – 7.12 (m, 2H), 6.73 – 6.63 (m, 1H), 6.56 (d, *J* = 15.7 Hz, 1H), 3.20 – 3.06 (m, 2H), 2.34 (s, 3H), 1.72 (s, 3H), 1.37 (s, 9H). **^{13}C NMR (100 MHz, CDCl$_3$)** δ 196.2, 170.6, 166.4, 151.0, 144.6, 140.9, 138.4, 137.1, 134.2, 131.4, 130.6, 128.3, 125.4, 125.3, 124.5, 120.3, 110.7, 82.8, 49.4, 40.2, 27.9, 21.7, 20.3. **ESI-MS:** calculated [$C_{25}H_{27}NO_4$ + Na]$^+$: 428.1832, found: 428.1831. $[\alpha]^{20}_{D}$ = +33.3 (c = 1.09, CH_2Cl_2). The product was analyzed by HPLC to determine the enantiomeric excess: 96% ee (CHIRALPAK IC, *n*-hexane/*i*-PrOH = 90/10, detector: 240 nm, T = 25 °C, flow rate: 1 mL/min), t_1 (minor) = 11.68 min, t_2 (major) = 13.19 min.

tert-Butyl (S,E)-2-(benzo[d]oxazol-2-yl)-6-mesityl-2-methyl-6-oxohex-4-enoate (3g)

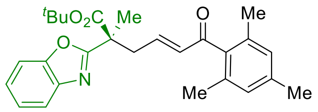

Reaction time: 2 h. **^{1}H NMR (400 MHz, CDCl$_3$)** δ 7.78 (d, *J* = 16.4 Hz, 1H), 7.74 – 7.69 (m, 1H), 7.55 – 7.48 (m, 1H), 7.36 – 7.30 (m, 2H), 6.90 (s, 2H), 6.42 (d, *J* = 16.4 Hz, 1H), 3.71 (d, *J* = 17.5 Hz, 1H), 3.52 (d, *J* = 17.5 Hz, 1H), 2.33 (s, 6H), 2.28 (s, 3H), 1.86 (s, 3H), 1.43 (s, 9H). **^{13}C NMR (100 MHz, CDCl$_3$)** δ 196.1, 170.4, 167.1, 151.0, 141.5, 141.1, 138.8, 137.3, 131.0, 130.9, 129.4, 125.0, 124.4, 120.2, 110.8, 82.4, 47.5, 27.9, 21.9, 21.3, 21.2. **ESI-MS:** calculated [$C_{27}H_{31}NO_4$ + Na]$^+$: 456.2145, found: 456.2146. $[\alpha]^{20}_{D}$ = +14.1 (c = 1.10, CH_2Cl_2). The product was analyzed by HPLC to determine the enantiomeric excess: 96% ee (CHIRALPAK IG, *n*-hexane/*i*-PrOH = 90/10, detector: 300 nm, T = 25 °C, flow rate: 1 mL/min), t_1 (minor) = 7.43 min, t_2 (major) = 8.97 min.

tert-Butyl (S,E)-2-(benzo[d]oxazol-2-yl)-2-methyl-6-(naphthalen-2-yl)-6-oxohex-4-enoate (3h)

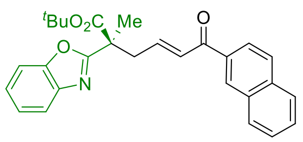

Reaction time: 2 h. **^{1}H NMR (400 MHz, CDCl$_3$)** δ 8.33 (s, 1H), 7.97 – 7.92 (m, 1H), 7.91 – 7.84 (m, 3H), 7.78 – 7.72 (m, 1H), 7.61 – 7.51 (m, 3H), 7.38 – 7.31 (m, 2H), 7.13 – 7.01 (m, 2H), 3.30 – 3.15 (m, 2H), 1.80 (s, 3H), 1.41 (s, 9H). **^{13}C NMR (100 MHz, CDCl$_3$)** δ 190.3, 170.7, 166.5, 151.0, 143.0, 140.9, 135.5, 134.9, 132.5, 130.3, 129.8, 129.6, 128.6, 128.5, 127.9, 126.8, 125.2, 124.5, 120.3, 110.7, 82.8, 49.5, 40.3, 27.9, 21.6. **ESI-MS:** calculated [$C_{28}H_{27}NO_4$ + Na]$^+$: 464.1832, found: 464.1830. $[\alpha]^{20}_{D}$ = +34.5 (c = 1.27, CH_2Cl_2). The product was analyzed by HPLC to determine the enantiomeric excess: 95% ee (CHIRALPAK IC, *n*-hexane/*i*-PrOH = 90/10, detector: 254 nm, T = 25 °C, flow rate: 1 mL/min), t_1 (minor) = 16.21 min, t_2 (major) = 19.61 min.

tert-Butyl (_S,E_)-2-(benzo[_d_]oxazol-2-yl)-2-methyl-6-oxo-6-(thiophen-2-yl)hex-4-enoate (3i)

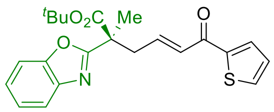

Reaction time: 2 h. **^{1}H NMR (400 MHz, CDCl$_3$)** δ 7.76 – 7.70 (m, 1H), 7.66 (dd, J = 3.8, 0.9 Hz, 1H), 7.63 (dd, J = 4.9, 0.9 Hz, 1H), 7.55 – 7.49 (m, 1H), 7.37 – 7.31 (m, 2H), 7.11 (dd, J = 4.9, 3.9 Hz, 1H), 7.05 (dt, J = 15.1, 7.5 Hz, 1H), 6.86 (d, J = 15.2 Hz, 1H), 3.23 – 3.10 (m, 2H), 1.76 (s, 3H), 1.40 (s, 9H). **^{13}C NMR (100 MHz, CDCl$_3$)** δ 181.8, 170.6, 166.4, 150.9, 144.9, 142.3, 140.9, 134.1, 132.2, 129.3, 128.3, 125.2, 124.5, 120.2, 110.7, 82.8, 49.4, 40.0, 27.9, 21.5. **ESI-MS:** calculated [$C_{22}H_{23}NO_4S$ + Na]$^+$: 420.1240, found: 420.1241. $[\alpha]^{20}_D$ = +36.5 (c = 1.23, CH_2Cl_2). The product was analyzed by HPLC to determine the enantiomeric excess: 96% ee (CHIRALPAK IC, _n_-hexane/_i_-PrOH = 90/10, detector: 300 nm, T = 25 °C, flow rate: 1 mL/min), t_1 (minor) = 17.40 min, t_2 (major) = 22.07 min.

tert-Butyl (_S,E_)-2-(benzo[_d_]oxazol-2-yl)-2-methyl-6-oxonon-4-en-7-ynoate (3j)

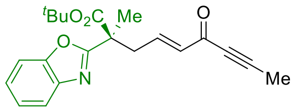

Reaction time: 2 h. **^{1}H NMR (400 MHz, CDCl$_3$)** δ 7.78 – 7.70 (m, 1H), 7.56 – 7.50 (m, 1H), 7.39 – 7.33 (m, 2H), 7.10 (dt, J = 15.4, 7.6 Hz, 1H), 6.22 (d, J = 15.7 Hz, 1H), 3.21 – 3.06 (m, 2H), 1.98 (s, 3H), 1.72 (s, 3H), 1.42 (s, 9H). **^{13}C NMR (100 MHz, CDCl$_3$)** δ 178.3, 170.4, 166.4, 151.0, 147.3, 140.9, 135.6, 125.3, 124.6, 120.3, 110.7, 91.1, 83.0, 78.3, 49.3, 39.9, 27.9, 21.7, 4.2. **ESI-MS:** calculated [$C_{21}H_{23}NO_4$ + Na]$^+$: 376.1519, found: 376.1518. $[\alpha]^{20}_D$ = +26.3 (c = 1.21, CH_2Cl_2). The product was analyzed by HPLC to determine the enantiomeric excess: 93% ee (CHIRALPAK IC, _n_-hexane/_i_-PrOH = 90/10, detector: 240 nm, T = 25 °C, flow rate: 1 mL/min), t_1 (minor) = 18.27 min, t_2 (major) = 21.35 min.

tert-Butyl (_S,E_)-2-(benzo[_d_]oxazol-2-yl)-2,7,7-trimethyl-6-oxooct-4-enoate (3k)

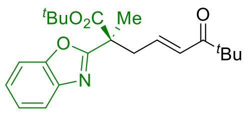

Reaction time: 2 h. **^{1}H NMR (400 MHz, CDCl$_3$)** δ 7.75 – 7.69 (m, 1H), 7.53 – 7.48 (m, 1H), 7.35 – 7.30 (m, 2H), 6.85 (dt, J = 15.2, 7.6 Hz, 1H), 6.55 (d, J = 15.2 Hz, 1H), 3.16 – 2.98 (m, 2H), 1.71 (s, 3H), 1.40 (s, 9H), 1.05 (s, 9H). **^{13}C NMR (100 MHz, CDCl$_3$)** δ 203.8, 170.7, 166.5, 151.0, 140.7, 128.3, 125.1, 124.4, 120.2, 110.6, 82.7, 49.4, 42.9, 39.9, 27.9, 26.1, 21.4 **ESI-MS:** calculated [$C_{22}H_{29}NO_4$ + Na]$^+$: 394.1989, found: 394.1987. $[\alpha]^{20}_D$ = +34.4 (c = 1.04, CH_2Cl_2). The product was analyzed by HPLC to determine the enantiomeric excess: 97% ee (CHIRALPAK IC, _n_-hexane/_i_-PrOH = 95/5, detector: 240 nm, T = 25 °C, flow rate: 1 mL/min), t_1 (minor) = 7.57 min, t_2 (major) = 9.22 min.

tert-Btyl (_S,E_)-2-(benzo[_d_]oxazol-2-yl)-2-methyl-6-oxohex-4-enoate (3l)

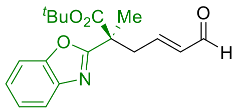

Reaction time: 2 h. **^{1}H NMR (500 MHz, CDCl$_3$)** δ 9.49 (d, J = 7.9 Hz, 1H), 7.77 – 7.70 (m, 1H), 7.57 – 7.49 (m, 1H), 7.40 – 7.31 (m, 2H), 6.87 (dt, J = 15.0, 7.4 Hz, 1H), 6.20 (dd, J = 15.6, 7.9 Hz, 1H), 3.26 – 3.08 (m, 2H), 1.73 (s, 3H), 1.40 (s, 9H). **^{13}C NMR (125 MHz, CDCl$_3$)** δ 193.7, 170.4, 166.1, 152.1, 151.0, 140.8, 136.2, 125.4, 124.6, 120.3, 110.7, 83.1, 49.2, 40.1, 27.9, 21.8. **ESI-MS:** calculated [$C_{18}H_{21}NO_4$ + Na]$^+$: 338.1363, found: 338.1363. $[\alpha]^{20}_D$ = +29.8 (c = 0.60, CH_2Cl_2). The product was analyzed by HPLC to determine the enantiomeric excess: 93% ee (CHIRALPAK IC, _n_-hexane/_i_-PrOH = 90/10, detector: 225 nm, T = 25 °C, flow rate: 1 mL/min), t_1 (minor) = 12.50 min, t_2 (major) = 14.06 min.

6-(_tert_-Butyl) 1-methyl (_S,E_)-5-(benzo[_d_]oxazol-2-yl)-5-methylhex-2-enedioate (3m)

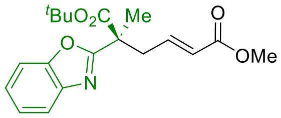

Reaction time: 3 h. **^{1}H NMR (400 MHz, CDCl$_3$)** δ 7.80 – 7.66 (m, 1H), 7.61 – 7.48 (m, 1H), 7.45 – 7.29 (m, 2H), 6.91 (dt, J = 15.4, 7.6 Hz, 1H), 5.94 (dt, J = 15.5, 1.4 Hz, 1H), 3.70 (s, 3H), 3.36 – 2.92 (m, 2H), 1.70 (s, 3H), 1.39 (s, 9H). **^{13}C NMR (100 MHz, CDCl$_3$)** δ 170.6, 166.4, 166.4, 151.0,

143.1, 140.9, 125.2, 125.0, 124.5, 120.3, 110.7, 82.8, 51.7, 49.2, 39.6, 27.9, 21.4. **ESI-MS:** calculated $[C_{19}H_{23}NO_5 + Na]^+$: 368.1468, found: 368.1475. The product was analyzed by HPLC to determine the enantiomeric excess: 95% ee (CHIRALPAK IE, *n*-hexane/*i*-PrOH = 90/10, detector: 254 nm, T = 25 °C, flow rate: 1 mL/min), t_1 (minor) = 7.76 min, t_2 (major) = 8.41 min.

***tert*-Butyl (*S*)-2-(benzo[*d*]oxazol-2-yl)-3-(2,2-dimethyl-4-oxo-4H-1,3-dioxin-6-yl)-2-methylpropanoate (3n)**

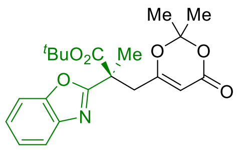

A 10 mL reaction tube was charged with racemic **1a** (0.1 mmol, 1 equiv), silyl enol ether 6-((tert-butyldimethylsilyl)oxy)-2,2-dimethyl-4H-1,3-dioxin-4-one (0.3 mmol, 3 equiv), nickel complex derived from (*R*,*R*)-**L7** (0.01 mmol, 0.1 equiv), Cp_2Fe (0.01 mmol, 0.1 equiv), $^nBu_4NPF_6$ (0.1 M), DCM (1.0 mL), MeCN (1.0 mL), MeOH (1.0 mL) under argon atmosphere. The reaction tube was equipped with a carbon rod (d = 6 mm) as the anode and a platinum plate (1.0 cm × 1.0 cm × 0.2 mm) as the cathode. The constant current (2.0 mA) electrolysis was carried out at 25 °C for 2 h until complete consumption of the substrate (monitored by TLC). The mixture was concentrated under reduced pressure, and purified by flash column chromatography on silica gel to afford the desired product **3n** (25.2 mg, 65%). **^{1}H NMR (400 MHz, $CDCl_3$)** δ 7.75 – 7.69 (m, 1H), 7.55 – 7.50 (m, 1H), 7.38 – 7.32 (m, 2H), 5.31 (s, 1H), 3.24 (d, *J* = 14.6 Hz, 1H), 3.08 (d, *J* = 14.6 Hz, 1H), 1.76 (s, 3H), 1.41 (s, 9H). **^{13}C NMR (100 MHz, $CDCl_3$)** δ 169.9, 167.2, 165.9, 160.8, 150.9, 140.8, 125.4, 124.6, 120.1, 110.6, 106.8, 96.5, 83.0, 48.3, 40.5, 27.9, 24.9, 24.8, 21.2. **ESI-MS:** calculated $[C_{21}H_{25}NO_6 + Na]^+$: 410.1574, found: 410.1574. $[\alpha]^{20}_D$ = +61.8 (c = 1.00, CH_2Cl_2). The product was analyzed by HPLC to determine the enantiomeric excess: 94% ee (CHIRALPAK IE, *n*-hexane/*i*-PrOH = 85/15, detector: 235 nm, T = 25 °C, flow rate: 1 mL/min), t_1 (minor) = 13.74 min, t_2 (major) = 15.38 min.

***tert*-butyl (*S*)-2-(benzo[*d*]oxazol-2-yl)-3-(5,5-dimethyl-3-oxocyclohex-1-en-1-yl)-2-methylpropanoate (3o)**

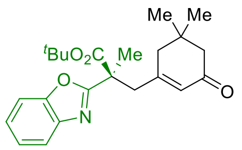

Reaction time: 2 h. **^{1}H NMR (500 MHz, $CDCl_3$)** δ 7.75 – 7.70 (m, 1H), 7.54 – 7.49 (m, 1H), 7.38 – 7.32 (m, 2H), 5.88 (s, 1H), 3.16 (d, *J* = 13.8 Hz, 1H), 3.09 (d, *J* = 13.8 Hz, 1H), 2.15 (s, 2H), 2.07 (d, *J* = 18.2 Hz, 1H), 1.90 (d, *J* = 18.2 Hz, 1H), 1.72 (s, 3H), 1.41 (s, 9H), 0.94 (s, 3H), 0.90 (s, 3H). **^{13}C NMR (125 MHz, $CDCl_3$)** δ 199.6, 170.8, 166.5, 158.0, 150.8, 140.8, 129.1, 125.4, 124.6, 120.3, 110.6, 83.0, 50.9, 49.2, 44.7, 44.6, 33.8, 28.3, 28.2, 27.9, 21.5. **ESI-MS:** calculated $[C_{23}H_{29}NO_4 + Na]^+$: 406.1989, found: 406.1985. $[\alpha]^{20}_D$ = +64.3 (c = 1.00, CH_2Cl_2). The product was analyzed by HPLC to determine the enantiomeric excess: 96% ee (CHIRALPAK IC, *n*-hexane/*i*-PrOH = 90/10, detector: 240 nm, T = 25 °C, flow rate: 1 mL/min), t_1 (minor) = 25.40 min, t_2 (major) = 28.91 min.

Methyl (*S*,*E*)-2-(benzo[*d*]oxazol-2-yl)-2-methyl-6-oxo-6-phenylhex-4-enoate (3p)

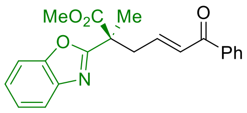

Reaction time: 2 h. **^{1}H NMR (400 MHz, $CDCl_3$)** δ 7.85 – 7.79 (m, 2H), 7.77 – 7.71 (m, 1H), 7.57 – 7.50 (m, 2H), 7.45 – 7.40 (m, 2H), 7.38 – 7.33 (m, 2H), 6.99 – 6.90 (m, 2H), 3.75 (s, 3H), 3.29 – 3.14 (m, 2H), 1.81 (s, 3H). **^{13}C NMR (100 MHz, $CDCl_3$)** δ 190.4, 172.3, 165.8, 151.0, 142.5, 140.9, 137.6, 133.0, 130.1, 128.7, 125.4, 124.7, 120.4, 110.9, 53.3, 48.8, 40.3, 21.6. **ESI-MS:** calculated $[C_{21}H_{19}NO_4 + Na]^+$: 372.1206, found: 372.1205. $[\alpha]^{20}_D$ = +49.3 (c = 0.90, CH_2Cl_2). The product was analyzed by HPLC to determine the enantiomeric excess: 92% ee (CHIRALPAK IF, *n*-hexane/*i*-PrOH = 90/10, detector: 240 nm, T = 25 °C, flow rate: 1 mL/min), t_1 (major) = 14.21 min, t_2 (minor) = 15.44 min.

Ethyl (*S*,*E*)-2-(benzo[*d*]oxazol-2-yl)-2-methyl-6-oxo-6-phenylhex-4-enoate (3q)

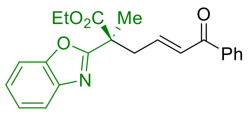

Reaction time: 2 h. **^{1}H NMR (400 MHz, $CDCl_3$)** δ 7.85 – 7.79 (m, 2H), 7.78 – 7.71 (m, 1H), 7.57 – 7.49 (m, 2H), 7.46 – 7.39 (m, 2H), 7.38 – 7.32 (m, 2H), 7.01 – 6.89 (m, 2H), 4.21 (q, *J* = 7.1 Hz, 2H), 3.30 – 3.12 (m, 2H), 1.81 (s, 3H), 1.21 (t, *J* = 7.1 Hz, 3H). **^{13}C NMR (100 MHz, $CDCl_3$)** δ 190.4, 171.7, 166.0, 151.0, 142.6, 140.9, 137.6, 133.0, 130.0, 128.7, 125.4, 124.6, 120.3, 110.8, 62.2, 48.8, 40.2, 21.6, 14.2. **ESI-MS:** calculated $[C_{22}H_{21}NO_4 + Na]^+$: 386.1363, found: 386.1363. $[\alpha]^{20}_D$ = +39.0 (c = 0.95, CH_2Cl_2). The product was analyzed by HPLC to determine the enantiomeric excess: 93% ee (CHIRALPAK IF, *n*-hexane/*i*-PrOH = 90/10, detector: 270 nm, T = 25 °C, flow rate: 1 mL/min), t_1 (major) = 12.74 min, t_2 (minor) = 13.77 min.

Isopropyl (*S*,*E*)-2-(benzo[*d*]oxazol-2-yl)-2-methyl-6-oxo-6-phenylhex-4-enoate (3r)

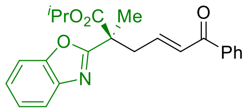

Reaction time: 2 h. **^{1}H NMR (400 MHz, $CDCl_3$)** δ 7.85 – 7.79 (m, 2H), 7.77 – 7.73 (m, 1H), 7.56 – 7.50 (m, 2H), 7.45 – 7.39 (m, 2H), 7.38 – 7.32 (m, 2H), 7.03 – 6.89 (m, 2H), 5.13 – 5.03 (m, 1H), 3.27 – 3.12 (m, 2H), 1.79 (s, 3H), 1.19 (d, *J* = 2.6 Hz, 3H), 1.17 (d, *J* = 2.6 Hz, 3H). **^{13}C NMR (100 MHz, $CDCl_3$)** δ 190.4, 171.2, 166.1, 151.0, 142.8, 140.9, 137.6, 132.9, 130.0, 128.7, 128.7, 125.3, 124.6, 120.3, 110.7, 69.9, 48.9, 40.2, 21.6, 21.6, 21.5. **ESI-MS:** calculated $[C_{23}H_{23}NO_4 + Na]^+$: 400.1519, found: 400.1519. $[\alpha]^{20}_D$ = +36.1 (c = 0.89, CH_2Cl_2). The product was analyzed by HPLC to determine the enantiomeric excess: 92% ee (CHIRALPAK IC, *n*-hexane/*i*-PrOH = 90/10, detector: 254 nm, T = 25 °C, flow rate: 1 mL/min), t_1 (minor) = 16.69 min, t_2 (major) = 18.04 min.

***tert*-Butyl (*S*,*E*)-2-allyl-2-(benzo[*d*]oxazol-2-yl)-6-oxo-6-phenylhex-4-enoate (3s)**

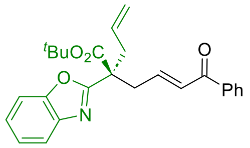

Reaction time: 2 h. **^{1}H NMR (400 MHz, $CDCl_3$)** δ 7.82 – 7.77 (m, 2H), 7.77 – 7.71 (m, 1H), 7.57 – 7.49 (m, 2H), 7.44 – 7.38 (m, 2H), 7.39 – 7.32 (m, 2H), 6.99 – 6.84 (m, 2H), 5.72 (ddt, *J* = 17.3, 10.1, 7.3 Hz, 1H), 5.26 – 5.08 (m, 2H), 3.18 (d, *J* = 6.2 Hz, 2H), 3.01 (d, *J* = 7.3 Hz, 2H), 1.40 (s, 9H). **^{13}C NMR (100 MHz, $CDCl_3$)** δ 190.5, 169.5, 165.5, 150.9, 142.9, 140.8, 137.6, 132.9, 131.9, 129.9, 128.7, 125.3, 124.6, 120.4, 120.1, 110.7, 83.1, 53.0, 38.6, 37.1, 28.0. **ESI-MS:** calculated $[C_{26}H_{27}NO_4 + Na]^+$: 440.1832, found: 440.1832. $[\alpha]^{20}_D$ = +17.7 (c = 1.03, CH_2Cl_2). The product was analyzed by HPLC to determine the enantiomeric excess: 90% ee (CHIRALPAK IC, *n*-hexane/*i*-PrOH = 95/5, detector: 254 nm, T = 25 °C, flow rate: 1 mL/min), t_1 (minor) = 12.26 min, t_2 (major) = 15.93 min.

***tert*-Butyl (*S*,*E*)-2-(benzo[*d*]oxazol-2-yl)-6-oxo-2-phenethyl-6-phenylhex-4-enoate (3t)**

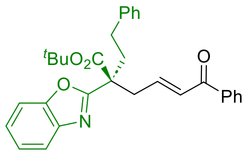

Reaction time: 2 h. **^{1}H NMR (400 MHz, $CDCl_3$)** δ 7.82 – 7.77 (m, 2H), 7.77 – 7.71 (m, 1H), 7.56 – 7.50 (m, 2H), 7.44 – 7.33 (m, 4H), 7.28 – 7.22 (m, 2H), 7.20 – 7.14 (m, 3H), 7.05 – 6.89 (m, 2H), 3.28 (d, *J* = 6.5 Hz, 2H), 2.77 – 2.48 (m, 4H), 1.43 (s, 9H). **^{13}C NMR (100 MHz, $CDCl_3$)** δ 190.5, 169.8, 165.6, 150.9, 142.9, 141.0, 140.8, 137.6, 132.9, 129.8, 128.7, 128.7, 128.6, 128.5, 126.3, 125.3, 124.6, 120.4, 110.7, 83.1, 53.4, 37.6, 36.4, 30.8, 28.0. **ESI-MS:** calculated $[C_{31}H_{31}NO_4 + Na]^+$: 504.2145, found: 504.2145. $[\alpha]^{20}_D$ = +25.0 (c = 1.31, CH_2Cl_2). The product was analyzed by HPLC to determine the enantiomeric excess: 90% ee (CHIRALPAK IC, *n*-hexane/*i*-PrOH = 95/5, detector: 254 nm, T = 25 °C, flow rate: 1 mL/min), t_1 (minor) = 14.52 min, t_2 (major) = 17.59 min.

***tert*-Butyl (*S*,*E*)-2-(benzo[*d*]oxazol-2-yl)-2-(3-((tert-butyldimethylsilyl)oxy)propyl)-6-oxo-6-phenyl-hex-4-enoate (3u)**

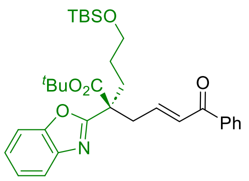

Reaction time: 2 h. **^{1}H NMR (400 MHz, $CDCl_3$)** δ 7.82 – 7.77 (m, 2H), 7.77 – 7.71 (m, 1H), 7.56 – 7.48 (m, 2H), 7.45 – 7.38 (m, 2H), 7.37 – 7.31 (m, 2H), 7.00 – 6.85 (m, 2H), 3.62 (t, *J* = 6.4 Hz, 2H), 3.26 – 3.12 (m, 2H), 2.35 – 2.19 (m, 2H), 1.67 – 1.47 (m, 2H), 1.40 (s, 9H), 0.85 (s, 9H), 0.01 (s, 3H), 0.01 (s, 3H). **^{13}C NMR (100 MHz, $CDCl_3$)** δ 190.6, 170.0, 165.9, 150.9, 143.2, 140.8, 137.6, 132.9, 129.7, 128.7, 128.6, 125.2, 124.5, 120.3, 110.7, 82.8, 63.0, 53.1, 37.5, 30.8, 28.0, 27.7, 26.0, 18.4, -5.2. **ESI-MS:** calculated $[C_{32}H_{43}NO_5Si + Na]^+$: 572.2803, found: 572.2804. $[\alpha]^{20}_D$ = +22.0 (c = 1.33, CH_2Cl_2). The product was analyzed by HPLC to determine the enantiomeric excess: 91% ee (CHIRALPAK IC, *n*-hexane/*i*-PrOH = 95/5, detector: 254 nm, T = 25 °C, flow rate: 1 mL/min), t_1 (minor) = 8.25 min, t_2 (major) = 10.73 min.

***tert*-Butyl (*S*,*E*)-2-(benzo[*d*]oxazol-2-yl)-2-(3-methoxypropyl)-6-oxo-6-phenylhex-4-enoate (3v)**

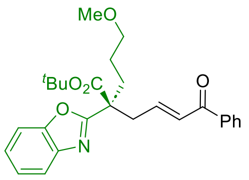

Reaction time: 2 h. **^{1}H NMR (400 MHz, $CDCl_3$)** δ 7.80 (m, 2H), 7.76 – 7.69 (m, 1H), 7.55 – 7.50 (m, 2H), 7.41 (m, 2H), 7.38 – 7.31 (m, 2H), 6.99 – 6.86 (m, 2H), 3.39 (t, *J* = 6.4 Hz, 2H), 3.29 (s, 3H), 3.22 – 3.17 (m, 2H), 2.33 – 2.25 (m, 2H), 1.72 – 1.62 (m, 1H), 1.59 – 1.50 (m, 1H), 1.40 (s, 9H). **^{13}C NMR (100 MHz, $CDCl_3$)** δ 190.6, 169.9, 165.8, 150.9, 143.0, 140.8, 137.6, 132.9, 129.8, 128.7, 128.6, 125.2, 124.5, 120.3, 110.7, 82.9, 72.4, 58.7, 53.1, 37.5, 31.0, 28.0, 24.6. **ESI-MS:** calculated $[C_{27}H_{31}NO_5 + Na]^+$: 4472.2094, found: 4472.2093. $[\alpha]^{20}_D$ = +28.2 (c = 1.23, CH_2Cl_2). The product was analyzed by HPLC to determine the enantiomeric excess: 90% ee (CHIRALPAK IC, *n*-hexane/*i*-PrOH = 90/10, detector: 254 nm, T = 25 °C, flow rate: 1 mL/min), t_1 (minor) = 15.05 min, t_2 (major) = 16.49 min.

***tert*-Butyl (*S*,*E*)-2-(benzo[*d*]oxazol-2-yl)-2-(cyclopropylmethyl)-6-oxo-6-phenylhex-4-enoate (3w)**

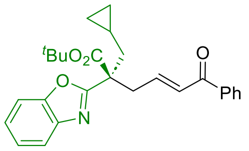

Reaction time: 2 h. **^{1}H NMR (400 MHz, $CDCl_3$)** δ 7.81 – 7.76 (m, 2H), 7.75 – 7.70 (m, 1H), 7.55 – 7.49 (m, 2H), 7.43 – 7.36 (m, 2H), 7.37 – 7.31 (m, 2H), 7.01 – 6.87 (m, 2H), 3.43 – 3.22 (m, 2H), 2.27 – 2.10 (m, 2H), 1.39 (s, 9H), 0.78 – 0.64 (m, 1H), 0.51 – 0.42 (m, 1H), 0.41 – 0.32 (m, 1H), 0.16 – 0.08 (m, 1H), 0.01 – -0.07 (m, 1H). **^{13}C NMR (100 MHz, $CDCl_3$)** δ 190.6, 170.0, 166.1, 150.8, 143.5, 140.8, 137.6, 132.9, 129.6, 128.6, 128.6, 125.2, 124.5, 120.3, 110.6, 82.8, 54.0, 39.1, 37.4, 28.0, 6.2, 4.7, 4.4. **ESI-MS:** calculated $[C_{27}H_{29}NO_4 + Na]^+$: 154.1989, found: 454.1991. $[\alpha]^{20}_D$ = +14.6 (c = 1.21, CH_2Cl_2). The product was analyzed by HPLC to determine the enantiomeric excess: 85% ee (CHIRALPAK IC, *n*-hexane/*i*-PrOH = 95/5, detector: 254 nm, T = 25 °C, flow rate: 1 mL/min), t_1 (minor) = 12.44 min, t_2 (major) = 15.64 min.

Dimethyl (*S*,*E*)-5-(benzo[*d*]oxazol-2-yl)-5-methylhex-2-enedioate (3x)

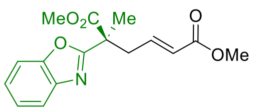

Reaction time: 3 h. **^{1}H NMR (500 MHz, $CDCl_3$)** δ 7.81 – 7.69 (m, 1H), 7.57 – 7.46 (m, 1H), 7.39 – 7.30 (m, 2H), 6.88 (dt, *J* = 15.4, 7.6 Hz, 1H), 5.94 (d, *J* = 15.5 Hz, 1H), 3.73 (s, 3H), 3.70 (s, 3H), 3.20 – 2.98 (m, 2H), 1.75 (s, 3H). **^{13}C NMR (125 MHz, $CDCl_3$)** δ 172.1, 166.3, 165.7, 151.0, 142.6, 140.9, 125.4, 125.3, 124.6, 120.4, 110.8, 53.2, 51.7, 48.5, 39.6, 21.4. **ESI-MS:** calculated $[C_{16}H_{17}NO_5 + Na]^+$: 326.0999, found: 326.1008. The product was analyzed by HPLC to determine the enantiomeric excess: 94% ee (CHIRALPAK IE, *n*-hexane/*i*-PrOH = 90/10, detector: 212 nm, T = 25 °C, flow rate: 1 mL/min), t_1 (major) = 12.00 min, t_2 (minor) = 12.67 min.

tert-Butyl (S)-2-(benzo[d]oxazol-2-yl)-2-methyl-4-oxo-4-phenylbutanoate (5a)

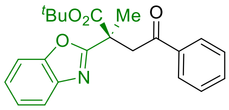

Reaction time: 1.5 h. (31.4 mg, 86%). **^{1}H NMR (500 MHz, $CDCl_3$)** δ 8.06 – 7.97 (m, 2H), 7.76 – 7.68 (m, 1H), 7.61 – 7.55 (m, 1H), 7.54 – 7.50 (m, 1H), 7.49 – 7.44 (m, 2H), 7.37 – 7.28 (m, 2H), 4.08 (d, *J* = 18.0 Hz, 1H), 3.87 (d, *J* = 18.0 Hz, 1H), 1.88 (s, 3H), 1.42 (s, 9H). **^{13}C NMR (125 MHz, $CDCl_3$)** δ 196.2, 170.4, 167.1, 151.0, 141.1, 136.9, 133.4, 128.7, 128.2, 125.0, 124.4, 120.2, 110.7, 82.4, 47.4, 45.3, 27.8, 22.0. **ESI-MS:** calculated $[C_{22}H_{23}NO_4 + Na]^+$: 388.1519, found: 388.1526. $[\alpha]^{20}_D$ = +10.3 (c = 1.20, CH_2Cl_2). The product was analyzed by HPLC to determine the enantiomeric excess: 94% ee (CHIRALPAK IA, *n*-hexane/*i*-PrOH = 95/5, detector: 240 nm, T = 25 °C, flow rate: 1 mL/min), t_1 (minor) = 6.43 min, t_2 (major) = 7.38 min.

tert-Butyl (S)-2-(benzo[d]oxazol-2-yl)-2-methyl-4-oxo-4-(p-tolyl)butanoate (5b)

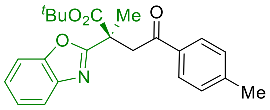

Reaction time: 2 h. (29.6 mg, 78%). **^{1}H NMR (500 MHz, $CDCl_3$)** δ 7.95 – 7.90 (m, 2H), 7.73 – 7.69 (m, 1H), 7.55 – 7.48 (m, 1H), 7.34 – 7.30 (m, 2H), 7.28 – 7.27 (m, 1H), 7.26 – 7.25 (m, 1H), 4.05 (d, *J* = 17.9 Hz, 1H), 3.84 (d, *J* = 17.9 Hz, 1H), 2.41 (s, 3H), 1.87 (s, 3H), 1.41 (s, 9H). **^{13}C NMR (125 MHz, $CDCl_3$)** δ 195.8, 170.5, 167.2, 151.0, 144.3, 141.1, 134.4, 129.4, 128.3, 125.0, 124.3, 120.1, 110.7, 82.3, 47.4, 45.2, 27.8, 22.0, 21.8. **ESI-MS:** calculated $[C_{23}H_{25}NO_4 + Na]^+$: 402.1676, found: 402.1684. $[\alpha]^{20}_D$ = +10.1 (c = 0.99, CH_2Cl_2). The product was analyzed by HPLC to determine the enantiomeric excess: 92% ee (CHIRALPAK IC, *n*-hexane/*i*-PrOH = 95/5, detector: 260 nm, T = 25 °C, flow rate: 1 mL/min), t_1 (minor) = 11.43 min, t_2 (major) = 15.17 min.

tert-Butyl (S)-2-(benzo[d]oxazol-2-yl)-4-(4-methoxyphenyl)-2-methyl-4-oxobutanoate (5c)

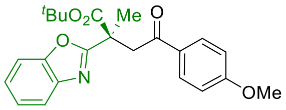

Reaction time: 2 h. (28.6 mg, 72%). **^{1}H NMR (500 MHz, $CDCl_3$)** δ 8.02 – 7.96 (m, 2H), 7.75 – 7.68 (m, 1H), 7.54 – 7.48 (m, 1H), 7.35 – 7.29 (m, 2H), 6.97 – 6.91 (m, 2H), 4.02 (d, *J* = 17.8 Hz, 1H), 3.87 (s, 3H), 3.82 (d, *J* = 17.8 Hz, 1H), 1.86 (s, 3H), 1.41 (s, 9H). **^{13}C NMR (125 MHz, $CDCl_3$)** δ 194.7, 170.6, 167.3, 163.8, 151.0, 141.1, 130.5, 130.0, 125.0, 124.4, 120.1, 113.9, 110.8, 82.3, 55.6, 47.5, 44.9, 27.8, 22.0. **ESI-MS:** calculated $[C_{23}H_{25}NO_5 + Na]^+$: 418.1625, found: 418.1630. $[\alpha]^{20}_D$ = +10.1 (c = 0.93, CH_2Cl_2). The product was analyzed by HPLC to determine the enantiomeric excess: 92% ee (CHIRALPAK IC, *n*-hexane/*i*-PrOH = 85/15, detector: 270 nm, T = 25 °C, flow rate: 1 mL/min), t_1 (minor) = 8.97 min, t_2 (major) = 11.40 min.

tert-Butyl (S)-4-([1,1'-biphenyl]-4-yl)-2-(benzo[d]oxazol-2-yl)-2-methyl-4-oxobutanoate (5d)

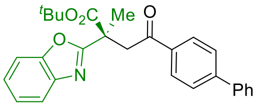

Reaction time: 2 h. (39.0 mg, 88%). **^{1}H NMR (500 MHz, $CDCl_3$)** δ 8.12 – 8.06 (m, 2H), 7.75 – 7.71 (m, 1H), 7.71 – 7.67 (m, 2H), 7.65 – 7.61 (m, 2H), 7.54 – 7.50 (m, 1H), 7.50 – 7.45 (m, 2H), 7.43 – 7.38 (m, 1H), 7.36 – 7.30 (m, 2H), 4.10 (d, *J* = 17.9 Hz, 1H), 3.90 (d, *J* = 17.9 Hz, 1H), 1.89 (s, 3H), 1.43 (s, 9H). **^{13}C NMR (125 MHz, $CDCl_3$)** δ 195.9, 170.5, 167.1, 151.0, 146.1, 141.1, 139.9, 135.6, 129.1, 128.8, 128.4, 127.4, 127.4, 125.0, 124.4, 120.2, 110.8, 82.4, 47.5, 45.4, 27.9, 22.0. **ESI-MS:** calculated $[C_{28}H_{27}NO_4 + H]^+$: 442.2013, found: 442.2017. $[\alpha]^{20}_D$ = +10.6 (c = 1.30, CH_2Cl_2). The product was analyzed by HPLC to determine the enantiomeric excess: 95% ee (CHIRALPAK IA, *n*-hexane/*i*-PrOH = 95/5, detector: 270 nm, T = 25 °C, flow rate: 1 mL/min), t_1 (minor) = 11.26 min, t_2 (major) = 13.20 min.

***tert*-Butyl (*S*)-2-(benzo[*d*]oxazol-2-yl)-4-(4-fluorophenyl)-2-methyl-4-oxobutanoate (5e)**

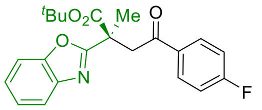

Reaction time: 2 h. (25.4 mg, 66%). **^{1}H NMR (500 MHz, CDCl$_3$)** δ 8.08 – 8.00 (m, 2H), 7.74 – 7.68 (m, 1H), 7.55 – 7.49 (m, 1H), 7.36 – 7.30 (m, 2H), 7.17 – 7.10 (m, 2H), 4.04 (d, *J* = 17.9 Hz, 1H), 3.82 (d, *J* = 17.9 Hz, 1H), 1.87 (s, 3H), 1.42 (s, 9H). **^{13}C NMR (125 MHz, CDCl$_3$)** δ 194.7, 170.4, 167.0, 166.0 (d, *J* = 254.9 Hz), 151.0, 141.1, 133.3 (d, *J* = 2.9 Hz), 130.9 (d, *J* = 9.5 Hz), 125.1, 124.4, 120.2, 115.9 (d, *J* = 21.9 Hz), 110.8, 82.5, 47.5, 45.2, 27.8, 22.0. **^{19}F NMR (471 MHz, CDCl$_3$)** δ -104.8. **ESI-MS:** calculated [$C_{22}H_{22}FNO_4$ + Na]$^+$: 406.1425, found: 406.1434. $[\alpha]^{20}_{D}$ = +11.3 (c = 0.77, CH_2Cl_2). The product was analyzed by HPLC to determine the enantiomeric excess: 96% ee (CHIRALPAK IA, *n*-hexane/*i*-PrOH = 95/5, detector: 240 nm, T = 25 °C, flow rate: 1 mL/min), t_1 (minor) = 8.30 min, t_2 (major) = 10.69 min.

***tert*-Butyl (*S*)-2-(benzo[*d*]oxazol-2-yl)-4-(4-chlorophenyl)-2-methyl-4-oxobutanoate (5f)**

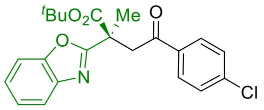

Reaction time: 1.5 h. (35.1 mg, 88%). **^{1}H NMR (500 MHz, CDCl$_3$)** δ 7.99 – 7.92 (m, 2H), 7.71 (dt, *J* = 7.6, 3.1 Hz, 1H), 7.55 – 7.47 (m, 1H), 7.49 – 7.40 (m, 2H), 7.37 – 7.28 (m, 2H), 4.03 (d, *J* = 18.0 Hz, 1H), 3.81 (d, *J* = 17.9 Hz, 1H), 1.87 (s, 3H), 1.41 (s, 9H). **^{13}C NMR (125 MHz, CDCl$_3$)** δ 195.1, 170.3, 166.9, 151.0, 141.0, 139.9, 135.2, 129.6, 129.1, 125.1, 124.4, 120.2, 110.8, 82.5, 47.5, 45.3, 27.8, 22.0. **ESI-MS:** calculated [$C_{22}H_{22}ClNO_4$ + Na]$^+$: 422.1130, found: 422.1133. $[\alpha]^{20}_{D}$ = +10.9 (c = 1.07, CH_2Cl_2). The product was analyzed by HPLC to determine the enantiomeric excess: 96% ee (CHIRALPAK IA, *n*-hexane/*i*-PrOH = 95/5, detector: 240 nm, T = 25 °C, flow rate: 1 mL/min), t_1 (minor) = 9.12 min, t_2 (major) = 11.98 min.

***tert*-Butyl (*S*)-2-(benzo[*d*]oxazol-2-yl)-4-(4-bromophenyl)-2-methyl-4-oxobutanoate (5g)**

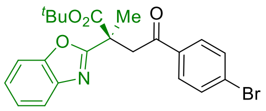

Reaction time: 1.5 h. (40.5 mg, 91%). **^{1}H NMR (500 MHz, CDCl$_3$)** δ 7.90 – 7.85 (m, 2H), 7.73 – 7.68 (m, 1H), 7.64 – 7.59 (m, 2H), 7.54 – 7.49 (m, 1H), 7.36 – 7.30 (m, 2H), 4.03 (d, *J* = 18.0 Hz, 1H), 3.80 (d, *J* = 18.0 Hz, 1H), 1.87 (s, 3H), 1.41 (s, 9H). **^{13}C NMR (125 MHz, CDCl$_3$)** δ 195.3, 170.3, 166.9, 151.0, 141.0, 135.6, 132.1, 129.7, 128.7, 125.1, 124.4, 120.2, 110.8, 82.5, 47.5, 45.3, 27.8, 22.0. **ESI-MS:** calculated [$C_{22}H_{22}BrNO_4$ + Na]$^+$: 466.0624, found: 466.0634. $[\alpha]^{20}_{D}$ = +11.3 (c = 1.27, CH_2Cl_2). The product was analyzed by HPLC to determine the enantiomeric excess: 96% ee (CHIRALPAK IA, *n*-hexane/*i*-PrOH = 95/5, detector: 240 nm, T = 25 °C, flow rate: 1 mL/min), t_1 (minor) = 9.71 min, t_2 (major) = 12.94 min.

Methyl (*S*)-4-(3-(benzo[*d*]oxazol-2-yl)-4-(*tert*-butoxy)-3-methyl-4-oxobutanoyl)benzoate (5h)

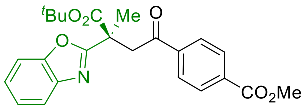

Reaction time: 1.5 h. (27.3 mg, 65%). **^{1}H NMR (500 MHz, CDCl$_3$)** δ 8.15 – 8.11 (m, 2H), 8.09 – 8.05 (m, 2H), 7.73 – 7.68 (m, 1H), 7.55 – 7.49 (m, 1H), 7.35 – 7.31 (m, 2H), 4.09 (d, *J* = 18.0 Hz, 1H), 3.96 (s, 3H), 3.86 (d, *J* = 18.0 Hz, 1H), 1.88 (s, 3H), 1.42 (s, 9H). **^{13}C NMR (125 MHz, CDCl$_3$)** δ 195.9, 170.3, 166.8, 166.3, 151.0, 141.0, 140.0, 134.2, 130.0, 128.1, 125.1, 124.4, 120.2, 110.8, 82.5, 52.6, 47.5, 45.7, 27.8, 22.0.. **ESI-MS:** calculated [$C_{24}H_{25}NO_6$ + Na]$^+$: 446.1574, found: 446.1584. $[\alpha]^{20}_{D}$ = +10.5 (c = 0.90, CH_2Cl_2). The product was analyzed by HPLC to determine the enantiomeric excess: 96% ee (CHIRALPAK IA, *n*-hexane/*i*-PrOH = 95/5, detector: 240 nm, T = 25 °C, flow rate: 1 mL/min), t_1 (minor) = 12.65 min, t_2 (major) = 14.77 min.

***tert*-Butyl (*S*)-2-(benzo[*d*]oxazol-2-yl)-2-methyl-4-oxo-4-(4-(trifluoromethyl)phenyl)butanoate (5i)**

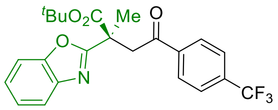

Reaction time: 1.5 h. (23.0 mg, 53%). **^{1}H NMR (400 MHz, CDCl$_3$)** δ 8.16 – 8.07 (m, 2H), 7.77 – 7.67 (m, 3H), 7.55 – 7.49 (m, 1H), 7.36 – 7.30 (m, 2H), 4.08 (d, *J* = 18.0 Hz, 1H), 3.85 (d, *J* = 18.0 Hz, 1H), 1.88 (s, 3H), 1.42

(s, 9H). **^{13}C NMR (100 MHz, CDCl$_3$)** δ 195.5, 170.3, 166.7, 151.0, 141.0, 139.5, 134.7 (d, *J* = 32.7 Hz), 128.6, 125.9 (q, *J* = 3.7 Hz), 125.1, 124.5, 123.7 (d, *J* = 272.8 Hz), 120.2, 110.8, 82.6, 47.5, 45.6, 27.8, 22.1. **^{19}F NMR (376 MHz, CDCl$_3$)** δ -63.1. **ESI-MS:** calculated $[C_{23}H_{22}F_3NO_4 + Na]^+$: 456.1393, found: 456.1401. $[\alpha]^{20}_D$ = +10.3 (c = 0.83, CH_2Cl_2). The product was analyzed by HPLC to determine the enantiomeric excess: 96% ee (CHIRALPAK IA, *n*-hexane/*i*-PrOH = 95/5, detector: 240 nm, T = 25 °C, flow rate: 1 mL/min), t_1 (minor) = 7.40 min, t_2 (major) = 10.48 min.

***tert*-Butyl (*S*)-2-(benzo[*d*]oxazol-2-yl)-2-methyl-4-oxo-4-(m-tolyl)butanoate (5j)**

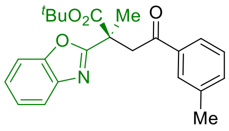

Reaction time: 1.5 h. (29.6 mg, 78%). **^{1}H NMR (400 MHz, CDCl$_3$)** δ 7.85 – 7.79 (m, 2H), 7.74 – 7.69 (m, 1H), 7.54 – 7.49 (m, 1H), 7.41 – 7.31 (m, 4H), 4.06 (d, *J* = 18.0 Hz, 1H), 3.86 (d, *J* = 18.0 Hz, 1H), 1.86 (s, 3H), 1.42 (s, 9H). **^{13}C NMR (100 MHz, CDCl$_3$)** δ 196.4, 170.5, 167.2, 151.0, 141.1, 138.6, 136.9, 134.2, 128.7, 128.6, 125.4, 125.0, 124.4, 120.2, 110.8, 82.3, 47.4, 45.4, 27.8, 22.0, 21.5. **ESI-MS:** calculated $[C_{23}H_{25}NO_4 + Na]^+$: 402.1676, found: 402.1685. $[\alpha]^{20}_D$ = +11.8 (c = 0.93, CH_2Cl_2). The product was analyzed by HPLC to determine the enantiomeric excess: 96% ee (CHIRALPAK IA, *n*-hexane/*i*-PrOH = 95/5, detector: 245 nm, T = 25 °C, flow rate: 1 mL/min), t_1 (minor) = 5.46 min, t_2 (major) = 5.93 min.

***tert*-Butyl (*S*)-2-(benzo[*d*]oxazol-2-yl)-4-(3-methoxyphenyl)-2-methyl-4-oxobutanoate (5k)**

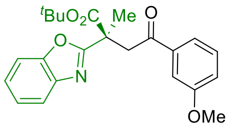

Reaction time: 1.5 h. (29.4 mg, 74%). **^{1}H NMR (400 MHz, CDCl$_3$)** δ 7.74 – 7.69 (m, 1H), 7.64 – 7.60 (m, 1H), 7.54 – 7.50 (m, 2H), 7.41 – 7.36 (m, 1H), 7.35 – 7.29 (m, 2H), 7.15 – 7.10 (m, 1H), 4.05 (d, *J* = 18.0 Hz, 1H), 3.86 (d, *J* = 18.0 Hz, 1H), 3.85 (s, 3H), 1.87 (s, 3H), 1.42 (s, 9H). **^{13}C NMR (100 MHz, CDCl$_3$)** δ 196.1, 170.5, 167.1, 160.0, 151.0, 141.1, 138.2, 129.7, 125.0, 124.4, 120.9, 120.2, 120.1, 112.2, 110.7, 82.4, 55.6, 47.5, 45.4, 27.8, 22.0. **ESI-MS:** calculated $[C_{23}H_{25}NO_5 + Na]^+$: 418.1625, found: 418.1631. $[\alpha]^{20}_D$ = +8.7 (c = 0.97, CH_2Cl_2). The product was analyzed by HPLC to determine the enantiomeric excess: 91% ee (CHIRALPAK IA, *n*-hexane/*i*-PrOH = 95/5, detector: 240 nm, T = 25 °C, flow rate: 1 mL/min), t_1 (minor) = 6.83 min, t_2 (major) = 7.34 min.

***tert*-Butyl (*S*)-2-(benzo[*d*]oxazol-2-yl)-4-(3-chlorophenyl)-2-methyl-4-oxobutanoate (5l)**

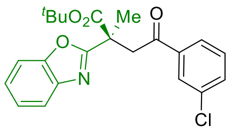

Reaction time: 2 h. (30.6 mg, 77%). **^{1}H NMR (400 MHz, CDCl$_3$)** δ 8.01 – 7.96 (m, 1H), 7.93 – 7.86 (m, 1H), 7.74 – 7.68 (m, 1H), 7.58 – 7.50 (m, 2H), 7.45 – 7.39 (m, 1H), 7.37 – 7.30 (m, 2H), 4.04 (d, *J* = 18.1 Hz, 1H), 3.82 (d, *J* = 18.1 Hz, 1H), 1.87 (s, 3H), 1.42 (s, 9H). **^{13}C NMR (100 MHz, CDCl$_3$)** δ 195.1, 170.3, 166.8, 151.0, 141.0, 138.4, 135.1, 133.4, 130.1, 128.3, 126.3, 125.1, 124.4, 120.2, 110.8, 82.5, 47.4, 45.4, 27.8, 22.0. **ESI-MS:** calculated $[C_{22}H_{22}ClNO_4 + Na]^+$: 422.1130, found: 422.1140. $[\alpha]^{20}_D$ = +10.2 (c = 1.00, CH_2Cl_2). The product was analyzed by HPLC to determine the enantiomeric excess: 92% ee (CHIRALPAK IA, *n*-hexane/*i*-PrOH = 95/5, detector: 240 nm, T = 25 °C, flow rate: 1 mL/min), t_1 (minor) = 5.81 min, t_2 (major) = 6.84 min.

***tert*-Butyl (*S*)-2-(benzo[*d*]oxazol-2-yl)-2-methyl-4-oxo-4-(o-tolyl)butanoate (5m)**

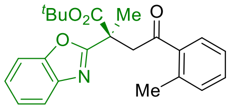

Reaction time: 2 h. (27.2 mg, 72%). **^{1}H NMR (400 MHz, CDCl$_3$)** δ 7.77 – 7.64 (m, 2H), 7.55 – 7.46 (m, 1H), 7.39 – 7.26 (m, 4H), 7.26 – 7.21 (m, 1H), 3.96 (d, *J* = 17.9 Hz, 1H), 3.78 (d, *J* = 17.9 Hz, 1H), 2.48 (s, 3H), 1.88 (s, 3H), 1.43 (s, 9H). **^{13}C NMR (100 MHz, CDCl$_3$)** δ 200.3, 170.5, 167.0, 151.0, 141.1, 138.2, 137.8, 132.1, 131.5, 128.5, 125.8, 125.0, 124.4, 120.1, 110.7, 82.3, 48.2, 47.6, 27.9, 22.1, 21.3. **ESI-MS:** calculated $[C_{23}H_{25}NO_4 + Na]^+$: 402.1676, found: 402.1682. $[\alpha]^{20}_D$ = +19.5 (c = 0.83, CH_2Cl_2). The product was analyzed by HPLC

to determine the enantiomeric excess: 92% ee (CHIRALPAK IC, *n*-hexane/*i*-PrOH = 95/5, detector: 240 nm, T = 25 °C, flow rate: 1 mL/min), t_1 (minor) = 7.98 min, t_2 (major) = 9.45 min.

***tert*-Butyl (*S*)-2-(benzo[*d*]oxazol-2-yl)-4-(2-methoxyphenyl)-2-methyl-4-oxobutanoate (5n)**

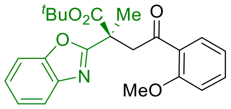

Reaction time: 4 h. (33.6 mg, 85%). **^{1}H NMR (400 MHz, $CDCl_3$)** δ 7.76 – 7.67 (m, 2H), 7.52 – 7.43 (m, 2H), 7.34 – 7.27 (m, 2H), 7.03 – 6.94 (m, 2H), 4.02 (d, *J* = 18.5 Hz, 1H), 3.93 (s, 3H), 3.89 (d, *J* = 18.5 Hz, 1H), 1.86 (s, 3H), 1.43 (s, 9H). **^{13}C NMR (100 MHz, $CDCl_3$)** δ 198.2, 170.8, 167.4, 158.8, 151.0, 141.2, 133.9, 130.5, 128.1, 124.8, 124.2, 120.8, 120.1, 111.6, 110.7, 82.1, 55.7, 50.5, 47.8, 27.9, 22.0. **ESI-MS:** calculated $[C_{23}H_{25}NO_5 + Na]^+$: 418.1625, found: 418.1632. $[\alpha]^{20}_D$ = +13.2 (c = 1.10, CH_2Cl_2). The product was analyzed by HPLC to determine the enantiomeric excess: 95% ee (CHIRALPAK IC, *n*-hexane/*i*-PrOH = 95/5, detector: 254 nm, T = 25 °C, flow rate: 1 mL/min), t_1 (minor) = 19.80 min, t_2 (major) = 21.23 min.

***tert*-Butyl (*S*)-2-(benzo[*d*]oxazol-2-yl)-4-(2-chlorophenyl)-2-methyl-4-oxobutanoate (5o)**

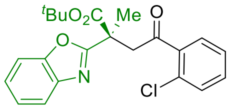

Reaction time: 2 h. (28.3 mg, 71%). **^{1}H NMR (400 MHz, $CDCl_3$)** δ 7.72 – 7.66 (m, 1H), 7.57 – 7.48 (m, 2H), 7.43 – 7.29 (m, 5H), 3.97 (d, *J* = 18.2 Hz, 1H), 3.83 (d, *J* = 18.2 Hz, 1H), 1.89 (s, 3H), 1.45 (s, 9H). **^{13}C NMR (100 MHz, $CDCl_3$)** δ 199.5, 170.2, 166.6, 151.0, 141.1, 139.2, 131.9, 130.9, 130.6, 129.1, 127.0, 125.1, 124.4, 120.2, 110.8, 82.6, 49.5, 47.8, 27.9, 22.0. **ESI-MS:** calculated $[C_{22}H_{22}ClNO_4 + Na]^+$: 422.1130, found: 422.1143. $[\alpha]^{20}_D$ = +23 (c = 0.8, CH_2Cl_2). The product was analyzed by HPLC to determine the enantiomeric excess: 93% ee (CHIRALPAK IA, *n*-hexane/*i*-PrOH = 95/5, detector: 240 nm, T = 25 °C, flow rate: 1 mL/min), t_1 (minor) = 6.17 min, t_2 (major) = 6.90 min.

***tert*-Butyl (*S*)-2-(benzo[*d*]oxazol-2-yl)-4-(furan-2-yl)-2-methyl-4-oxobutanoate (5p)**

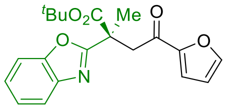

Reaction time: 2 h. (26.5 mg, 75%). **^{1}H NMR (500 MHz, $CDCl_3$)** δ 7.75 – 7.67 (m, 1H), 7.60 – 7.55 (m, 1H), 7.54 – 7.48 (m, 1H), 7.36 – 7.28 (m, 2H), 7.26 – 7.22 (m, 1H), 6.54 (dd, *J* = 3.5, 1.7 Hz, 1H), 3.90 (d, *J* = 17.6 Hz, 1H), 3.71 (d, *J* = 17.6 Hz, 1H), 1.86 (s, 3H), 1.42 (s, 9H). **^{13}C NMR (125 MHz, $CDCl_3$)** δ 185.4, 170.3, 166.8, 152.6, 151.0, 146.5, 141.1, 125.0, 124.4, 120.2, 117.3, 112.5, 110.7, 82.5, 47.4, 44.7, 27.8, 21.9. **ESI-MS:** calculated $[C_{20}H_{21}NO_5 + Na]^+$: 378.1312, found: 378.1319. $[\alpha]^{20}_D$ = +18.6 (c = 0.87, CH_2Cl_2). The product was analyzed by HPLC to determine the enantiomeric excess: 96% ee (CHIRALPAK IA, *n*-hexane/*i*-PrOH = 95/5, detector: 240 nm, T = 25 °C, flow rate: 1 mL/min), t_1 (minor) = 7.99 min, t_2 (major) = 10.10 min.

***tert*-Butyl (*S*)-2-(benzo[*d*]oxazol-2-yl)-4-(benzofuran-2-yl)-2-methyl-4-oxobutanoate (5q)**

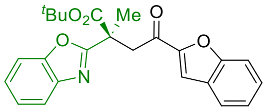

Reaction time: 2 h. (36.1 mg, 89%). **^{1}H NMR (500 MHz, $CDCl_3$)** δ 7.73 – 7.69 (m, 2H), 7.59 – 7.55 (m, 2H), 7.54 – 7.51 (m, 1H), 7.50 – 7.46 (m, 1H), 7.35 – 7.29 (m, 3H), 4.05 (d, *J* = 17.7 Hz, 1H), 3.85 (d, *J* = 17.7 Hz, 1H), 1.90 (s, 3H), 1.44 (s, 9H). **^{13}C NMR (125 MHz, $CDCl_3$)** δ 187.5, 170.2, 166.7, 155.7, 152.5, 151.0, 141.1, 128.5, 127.1, 125.1, 124.4, 124.1, 123.5, 120.2, 113.0, 112.6, 110.8, 82.6, 47.4, 45.2, 27.9, 22.0. **ESI-MS:** calculated $[C_{24}H_{23}NO_5 + H]^+$: 406.1649, found: 406.1852. $[\alpha]^{20}_D$ = +13.6 (c = 1.17, CH_2Cl_2). The product was analyzed by HPLC to determine the enantiomeric excess: 96% ee (CHIRALPAK IA, *n*-hexane/*i*-PrOH = 95/5, detector: 285 nm, T = 25 °C, flow rate: 1 mL/min), t_1 (minor) = 9.54 min, t_2 (major) = 10.30 min.

***tert*-Butyl (*S*)-2-(benzo[*d*]oxazol-2-yl)-2-methyl-4-(naphthalen-2-yl)-4-oxobutanoate (5r)**

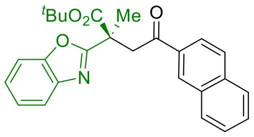

Reaction time: 2 h. (33.5 mg, 81%). **^{1}H NMR (500 MHz, CDCl$_3$)** δ 8.57 (m, 1H), 8.06 (dd, *J* = 8.6, 1.7 Hz, 1H), 7.98 (d, *J* = 8.0 Hz, 1H), 7.89 (m, 2H), 7.76 – 7.70 (m, 1H), 7.65 – 7.51 (m, 3H), 7.38 – 7.29 (m, 2H), 4.23 (d, *J* = 17.8 Hz, 1H), 4.01 (d, *J* = 17.8 Hz, 1H), 1.92 (s, 3H), 1.43 (s, 9H). **^{13}C NMR (125 MHz, CDCl$_3$)** δ 196.2, 170.5, 167.2, 151.0, 141.1, 135.8, 134.2, 132.6, 130.0, 129.7, 128.7, 128.6, 127.9, 127.0, 125.0, 124.4, 123.8, 120.2, 110.8, 82.4, 47.6, 45.3, 27.9, 22.1. **ESI-MS:** calculated [$C_{26}H_{25}NO_4$ + Na]$^+$: 438.1676, found: 438.1683. $[\alpha]^{20}_{D}$ = +8.0 (c = 1.07, CH_2Cl_2). The product was analyzed by HPLC to determine the enantiomeric excess: 96% ee (CHIRALPAK IA, *n*-hexane/*i*-PrOH = 95/5, detector: 249 nm, T = 25 °C, flow rate: 1 mL/min), t_1 (minor) = 8.79 min, t_2 (major) = 9.50 min.

***tert*-Butyl (*S*)-2-(benzo[*d*]oxazol-2-yl)-4-(cyclohex-1-en-1-yl)-2-methyl-4-oxobutanoate (5s)**

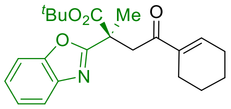

A 10 mL flask was charged with **1a** (0.1 mmol, 1 equiv), ((1-(cyclohex-1-en-1-yl)vinyl)oxy)trimethylsilane (0.3 mmol, 3 equiv), Ni catalyst (0.01 mmol, 0.1 equiv), $^{n}Bu_4NPF_6$ (0.1 M), Cp_2Fe (0.01 mmol, 0.1 equiv), DCM (1 mL), MeCN (0.5 mL), and TFE (1.5 mL) under argon atmosphere. The flask was equipped with a carbon rod (d = 6 mm) as the anode and a platinum plate (1.0 cm × 1.0 cm × 0.2 mm) as the cathode. The constant current (3.0 mA) electrolysis was carried out at 25 °C until complete consumption of the substrate (monitored by TLC). The mixture was concentrated under reduced pressure, and purified by silica gel chromatography to afford the desired product **5s** (23.5 mg, 64%). Reaction time: 1.5 h. **^{1}H NMR (400 MHz, CDCl$_3$)** δ 7.75 – 7.66 (m, 1H), 7.53 – 7.47 (m, 1H), 7.33 – 7.28 (m, 2H), 7.01 – 6.96 (m, 1H), 3.68 (d, *J* = 17.4 Hz, 1H), 3.54 (d, *J* = 17.4 Hz, 1H), 2.26 – 2.18 (m, 4H), 1.78 (s, 3H), 1.65 – 1.55 (m, 4H), 1.41 (s, 9H). **^{13}C NMR (100 MHz, CDCl$_3$)** δ 197.2, 170.7, 167.4, 150.9, 141.2, 140.5, 139.4, 124.9, 124.3, 120.1, 110.7, 82.1, 47.5, 43.8, 27.9, 26.2, 23.1, 22.0, 21.9, 21.6. **ESI-MS:** calculated [$C_{22}H_{27}NO_4$ + Na]$^+$: 392.1832, found: 392.1840. $[\alpha]^{20}_{D}$ = +3.3 (c = 0.77, CH_2Cl_2). The product was analyzed by HPLC to determine the enantiomeric excess: 95% ee (CHIRALPAK IA, *n*-hexane/*i*-PrOH = 95/5, detector: 235 nm, T = 25 °C, flow rate: 1 mL/min), t_1 (minor) = 5.45 min, t_2 (major) = 6.07 min.

***tert*-Butyl (*S*,*E*)-2-(benzo[*d*]oxazol-2-yl)-2-methyl-4-oxo-6-phenylhex-5-enoate (5t)**

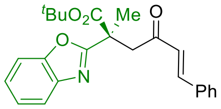

Reaction time: 1.5 h. (33.4 mg, 85%). **^{1}H NMR (500 MHz, CDCl$_3$)** δ 7.75 – 7.68 (m, 1H), 7.61 (d, *J* = 16.2 Hz, 1H), 7.58 – 7.49 (m, 3H), 7.43 – 7.36 (m, 3H), 7.35 – 7.29 (m, 2H), 6.76 (d, *J* = 16.2 Hz, 1H), 3.75 (d, *J* = 17.6 Hz, 1H), 3.57 (d, *J* = 17.5 Hz, 1H), 1.84 (s, 3H), 1.43 (s, 9H). **^{13}C NMR (125 MHz, CDCl$_3$)** δ 196.1, 170.4, 167.0, 151.0, 143.2, 141.1, 134.4, 130.7, 129.1, 128.5, 126.1, 125.0, 124.4, 120.2, 110.8, 82.4, 47.4, 47.2, 27.8, 21.9. **ESI-MS:** calculated [$C_{24}H_{25}NO_4$ + H]$^+$: 392.1856, found: 392.1860. $[\alpha]^{20}_{D}$ = +26.7 (c = 1.03, CH_2Cl_2). The product was analyzed by HPLC to determine the enantiomeric excess: 98% ee (CHIRALPAK IA, *n*-hexane/*i*-PrOH = 95/5, detector: 280 nm, T = 25 °C, flow rate: 1 mL/min), t_1 (minor) = 9.02 min, t_2 (major) = 9.54 min.

***tert*-Butyl (2*S*)-2-(benzo[*d*]oxazol-2-yl)-2-(1-oxo-1,2,3,4-tetrahydronaphthalen-2-yl)propanoate (5u)**

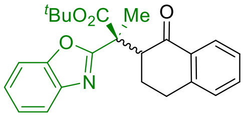

Reaction time: 5 h. (27.4 mg, 70%, 3:1 dr).

major isomer: **^{1}H NMR (400 MHz, CDCl$_3$)** δ 8.04 (dd, *J* = 7.8, 1.5 Hz, 1H), 7.71 – 7.65 (m, 1H), 7.50 – 7.42 (m, 2H), 7.35 – 7.21 (m, 4H), 3.68 (dd, *J* = 13.8, 4.1 Hz, 1H), 3.15 (ddd, *J* = 17.1, 12.9, 4.4 Hz, 1H), 3.01 (dt, *J* = 16.5, 3.5 Hz, 1H), 2.23 – 2.13 (m, 1H), 2.09 – 1.96 (m, 1H), 1.88 (s, 3H), 1.45 (s, 9H). **^{13}C NMR (100 MHz, CDCl$_3$)** δ 196.0, 171.0, 166.3, 150.9, 143.5, 141.1, 133.5, 132.8, 128.6, 127.9, 126.8, 124.7, 124.1, 120.2, 110.7, 82.3, 54.5, 51.3, 29.9,

27.9, 25.5, 19.9. **ESI-MS:** calculated [$C_{24}H_{25}NO_4$ + Na]$^+$: 414.1676, found: 414.1682. The product was analyzed by HPLC to determine the enantiomeric excess: 92% ee (CHIRALPAK IA, *n*-hexane/*i*-PrOH = 95/5, detector: 245 nm, T = 25 °C, flow rate: 1 mL/min), t_1 (minor) = 7.34 min, t_2 (major) = 8.87 min.
minor isomer: **^{1}H NMR (400 MHz, $CDCl_3$)** δ 8.04 (dd, *J* = 7.8, 1.4 Hz, 1H), 7.84 – 7.69 (m, 1H), 7.61 – 7.52 (m, 1H), 7.48 (td, *J* = 7.5, 1.5 Hz, 1H), 7.41 – 7.23 (m, 4H), 4.16 – 3.92 (m, 1H), 3.20 (ddd, *J* = 17.1, 11.5, 5.7 Hz, 1H), 2.98 (dt, *J* = 16.8, 3.5 Hz, 1H), 2.17 – 2.00 (m, 2H), 1.79 (s, 3H), 1.43 (s, 9H). **^{13}C NMR (100MHz, $CDCl_3$)** δ 196.4, 169.8, 166.4, 151.0, 143.8, 141.1, 133.6, 132.9, 128.8, 127.7, 126.8, 125.1, 124.5, 120.3, 110.8, 82.1, 53.9, 51.4, 29.9, 27.9, 26.1, 16.4. **ESI-MS:** calculated [$C_{24}H_{25}NO_4$ + Na]$^+$: 414.1676, found: 414.1692. The product was analyzed by HPLC to determine the enantiomeric excess: 96% ee (CHIRALPAK IG, *n*-hexane/*i*-PrOH = 90/10, detector: 254 nm, T = 25 °C, flow rate: 1 mL/min), t_1 (minor) = 10.05 min, t_2 (major) = 11.26 min.

***tert*-Butyl (2*S*)-2-(benzo[*d*]oxazol-2-yl)-2-(4-oxochroman-3-yl)propanoate (5v)**

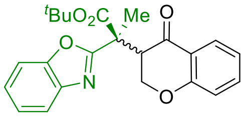

Reaction time: 5 h. (35.8 mg, 91%, 2:1 dr).
major isomer: **^{1}H NMR (400 MHz, $CDCl_3$)** δ 7.81 (dd, *J* = 7.9, 1.8 Hz, 1H), 7.65 – 7.55 (m, 1H), 7.43 – 7.33 (m, 2H), 7.26 – 7.17 (m, 2H), 6.99 – 6.90 (m, 1H), 6.86 (dd, *J* = 8.4, 1.0 Hz, 1H), 4.44 (d, *J* = 9.0 Hz, 2H), 3.83 (t, *J* = 8.9 Hz, 1H), 1.81 (s, 3H), 1.36 (s, 9H). **^{13}C NMR (100 MHz, $CDCl_3$)** δ 190.8, 170.0, 165.1, 161.6, 150.9, 140.9, 136.0, 127.8, 125.1, 124.4, 121.7, 121.4, 120.4, 117.8, 110.8, 83.0, 68.6, 51.9, 49.2, 27.9, 20.9. **ESI-MS:** calculated [$C_{23}H_{23}NO_5$ + Na]$^+$: 416.1468, found: 416.1478. The product was analyzed by HPLC to determine the enantiomeric excess: 94% ee (CHIRALPAK IG, *n*-hexane/*i*-PrOH = 90/10, detector: 240 nm, T = 25 °C, flow rate: 1 mL/min), t_1 (minor) = 10.32 min, t_2 (major) = 13.69 min.
minor isomer: **^{1}H NMR (400 MHz, $CDCl_3$)** δ 7.90 (dd, *J* = 7.9, 1.8 Hz, 1H), 7.79 – 7.68 (m, 1H), 7.58 – 7.51 (m, 1H), 7.52 – 7.45 (m, 1H), 7.41 – 7.31 (m, 2H), 7.03 (ddd, *J* = 8.0, 7.1, 1.1 Hz, 1H), 6.97 (dd, *J* = 8.3, 1.1 Hz, 1H), 4.73 (dd, *J* = 10.9, 4.9 Hz, 1H), 4.53 (dd, *J* = 13.1, 10.9 Hz, 1H), 4.28 (dd, *J* = 13.1, 4.9 Hz, 1H), 1.80 (s, 3H), 1.43 (s, 9H). **^{13}C NMR (100 MHz, $CDCl_3$)** δ 191.2, 169.1, 165.2, 161.7, 151.0, 141.0, 136.1, 127.6, 125.4, 124.6, 121.6, 121.4, 120.4, 117.8, 110.9, 82.8, 68.9, 50.8, 48.9, 27.8, 17.6. **ESI-MS:** calculated [$C_{23}H_{23}NO_5$ + Na]$^+$: 416.1468, found: 416.1472. The product was analyzed by HPLC to determine the enantiomeric excess: 94% ee (CHIRALPAK IE, *n*-hexane/*i*-PrOH = 90/10, detector: 240 nm, T = 25 °C, flow rate: 1 mL/min), t_1 (minor) = 10.15 min, t_2 (major) = 13.70 min.

Methyl (*S*)-2-(benzo[*d*]oxazol-2-yl)-2-methyl-4-oxo-4-phenylbutanoate (5w)

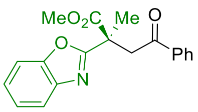

Reaction time: 1.5 h. (23.9 mg, 74%). **^{1}H NMR (500 MHz, $CDCl_3$)** δ 8.04 – 7.99 (m, 2H), 7.75 – 7.69 (m, 1H), 7.59 (t, *J* = 7.4 Hz, 1H), 7.54 – 7.44 (m, 3H), 7.37 – 7.30 (m, 2H), 4.15 (d, *J* = 18.0 Hz, 1H), 3.92 (d, *J* = 18.0 Hz, 1H), 3.77 (s, 3H), 1.92 (s, 3H). **^{13}C NMR (125 MHz, $CDCl_3$)** δ 196.2, 172.2, 166.4, 151.0, 141.0, 136.6, 133.6, 128.8, 128.2, 125.2, 124.5, 120.3, 110.9, 53.3, 46.6, 45.5, 22.0. **ESI-MS:** calculated [$C_{19}H_{17}NO_4$ + H]$^+$: 324.1230, found: 324.1234. $[\alpha]^{20}_D$ = +20.4 (c = 0.90, CH_2Cl_2). The product was analyzed by HPLC to determine the enantiomeric excess: 92% ee (CHIRALPAK IA, *n*-hexane/*i*-PrOH = 95/5, detector: 240 nm, T = 25 °C, flow rate: 1 mL/min), t_1 (minor) = 12.89 min, t_2 (major) = 14.71 min.

Ethyl (*S*)-2-(benzo[*d*]oxazol-2-yl)-2-methyl-4-oxo-4-phenylbutanoate (5x)

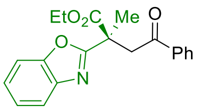

Reaction time: 1.5 h. (27.5 mg, 82%). **^{1}H NMR (500 MHz, $CDCl_3$)** δ 8.05 – 7.98 (m, 2H), 7.75 – 7.69 (m, 1H), 7.61 – 7.55 (m, 1H), 7.55 – 7.44 (m, 3H), 7.36 – 7.29 (m, 2H), 4.30 – 4.19 (m, 2H), 4.15 (d, *J* = 18.0 Hz, 1H), 3.91 (d, *J* = 18.0 Hz, 1H), 1.92 (s, 3H), 1.22 (t, *J* = 7.1 Hz, 3H). **^{13}C NMR (125 MHz, $CDCl_3$)** δ 196.2, 171.6, 166.6, 151.0, 141.1,

136.7, 133.6, 128.8, 128.2, 125.2, 124.5, 120.2, 110.9, 62.1, 46.7, 45.4, 21.9, 14.1. **ESI-MS:** calculated $[C_{20}H_{19}NO_4 + H]^+$: 338.1387, found: 338.1391. $[\alpha]^{20}_D$ = +15.3 (c = 0.80, CH_2Cl_2). The product was analyzed by HPLC to determine the enantiomeric excess: 92% ee (CHIRALPAK IA, *n*-hexane/*i*-PrOH = 95/5, detector: 240 nm, T = 25 °C, flow rate: 1 mL/min), t_1 (minor) = 12.17 min, t_2 (major) = 13.49 min.

Benzyl (*S*)-2-(benzo[*d*]oxazol-2-yl)-2-methyl-4-oxo-4-phenylbutanoate (5y)

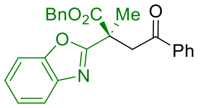

Reaction time: 1.5 h. (24.3 mg, 61%). **^{1}H NMR (500 MHz, $CDCl_3$)** δ 8.06 – 7.95 (m, 2H), 7.74 – 7.67 (m, 1H), 7.62 – 7.54 (m, 1H), 7.52 – 7.43 (m, 3H), 7.37 – 7.28 (m, 2H), 7.28 – 7.20 (m, 5H), 5.23 (d, *J* = 12.5 Hz, 1H), 5.20 (d, *J* = 12.5 Hz, 1H), 4.16 (d, *J* = 18.0 Hz, 1H), 3.92 (d, *J* = 18.0 Hz, 1H), 1.94 (s, 3H). **^{13}C NMR (125 MHz, $CDCl_3$)** δ 196.1, 171.4, 166.4, 151.0, 141.0, 136.6, 135.6, 133.6, 128.8, 128.5, 128.2, 128.2, 128.0, 125.2, 124.5, 120.2, 110.8, 67.6, 46.8, 45.4, 21.9. **ESI-MS:** calculated $[C_{25}H_{21}NO_4 + H]^+$: 400.1543, found: 400.1554. $[\alpha]^{20}_D$ = +18.1 (c = 0.77, CH_2Cl_2). The product was analyzed by HPLC to determine the enantiomeric excess: 88% ee (CHIRALPAK IC, *n*-hexane/*i*-PrOH = 95/5, detector: 240 nm, T = 25 °C, flow rate: 1 mL/min), t_1 (major) = 20.79 min, t_2 (minor) = 23.28 min.

Ethyl (*S*)-2-(benzo[*d*]oxazol-2-yl)-4-(4-methoxyphenyl)-2-methyl-4-oxobutanoate (5z)

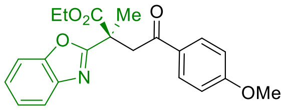

Reaction time: 1.5 h. (31.6 mg, 86%). **^{1}H NMR (400 MHz, $CDCl_3$)** δ 8.02 – 7.97 (m, 2H), 7.75 – 7.69 (m, 1H), 7.54 – 7.49 (m, 1H), 7.35 – 7.29 (m, 2H), 6.96 – 6.91 (m, 2H), 4.27 – 4.18 (m, 2H), 4.10 (d, *J* = 17.8 Hz, 1H), 3.86 (d, *J* = 17.8 Hz, 1H), 3.86 (s, 3H), 1.91 (s, 3H), 1.21 (t, *J* = 7.1 Hz, 3H). **^{13}C NMR (100 MHz, $CDCl_3$)** δ 194.6, 171.6, 166.7, 163.8, 150.9, 141.0, 130.5, 129.8, 125.1, 124.4, 120.2, 113.8, 110.8, 62.0, 55.6, 46.7, 45.0, 21.9, 14.0. **ESI-MS:** calculated $[C_{21}H_{21}NO_5 + Na]^+$: 390.1312, found: 390.1311. $[\alpha]^{20}_D$ = +19.7 (c = 1.01, CH_2Cl_2). The product was analyzed by HPLC to determine the enantiomeric excess: 96% ee (CHIRALPAK IC, *n*-hexane/*i*-PrOH = 80/20, detector: 254 nm, T = 25 °C, flow rate: 1 mL/min), t_1 (minor) = 18.84 min, t_2 (major) = 21.66 min.

***tert*-Butyl (*S*)-2-(benzo[*d*]oxazol-2-yl)-2-ethyl-4-oxo-4-phenylbutanoate (5aa)**

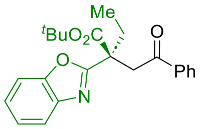

Reaction time: 2 h. (26.2 mg, 69%). **^{1}H NMR (400 MHz, $CDCl_3$)** δ 8.09 – 7.99 (m, 2H), 7.76 – 7.66 (m, 1H), 7.61 – 7.43 (m, 4H), 7.35 – 7.27 (m, 2H), 4.07 (d, *J* = 18.0 Hz, 1H), 3.92 (d, *J* = 18.0 Hz, 1H), 2.51 (dq, *J* = 15.1, 7.6 Hz, 1H), 2.39 (dq, *J* = 14.9, 7.5 Hz, 1H), 1.41 (s, 9H), 0.86 (t, *J* = 7.6 Hz, 3H). **^{13}C NMR (100 MHz, $CDCl_3$)** δ 196.5, 170.1, 166.3, 151.0, 141.0, 136.9, 133.4, 128.7, 128.2, 124.9, 124.3, 120.1, 110.7, 82.2, 51.7, 41.7, 27.9, 27.7, 9.2. **ESI-MS:** calculated $[C_{23}H_{25}NO_4 + Na]^+$: 402.1676, found: 402.1677. $[\alpha]^{20}_D$ = +20.9 (c = 0.83, CH_2Cl_2). The product was analyzed by HPLC to determine the enantiomeric excess: 91% ee (CHIRALPAK IA, *n*-hexane/*i*-PrOH = 97/3, detector: 240 nm, T = 25 °C, flow rate: 1 mL/min), t_1 (minor) = 7.71 min, t_2 (major) = 10.49 min.

***tert*-Butyl (*S*)-2-(benzo[*d*]oxazol-2-yl)-2-(2-oxo-2-phenylethyl)pentanoate (5ab)**

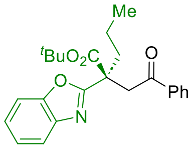

Reaction time: 2 h. (24.7 mg, 63%). **^{1}H NMR (400 MHz, $CDCl_3$)** δ 8.07 – 8.00 (m, 2H), 7.73 – 7.66 (m, 1H), 7.61 – 7.43 (m, 4H), 7.35 – 7.28 (m, 2H), 4.08 (d, *J* = 18.1 Hz, 1H), 3.93 (d, *J* = 18.1 Hz, 1H), 2.47 – 2.37 (m, 1H), 2.36 – 2.26 (m, 1H), 1.41 (s, 9H), 1.36 – 1.25 (m, 1H), 1.19 – 1.05 (m, 1H), 0.89 (t, *J* = 7.2 Hz, 3H). **^{13}C NMR (100 MHz, $CDCl_3$)** δ 196.5, 170.1, 166.5, 151.0, 141.0, 136.9, 133.4, 128.7, 128.2, 124.9, 124.3, 120.1, 110.8, 82.3, 51.3, 42.2, 36.8, 27.9, 18.1, 14.4. **ESI-MS:** calculated $[C_{24}H_{27}NO_4 + Na]^+$: 416.1832, found: 416.1837. $[\alpha]^{20}_D$ = +23.1 (c = 0.60, CH_2Cl_2). The product was analyzed by HPLC to determine the

enantiomeric excess: 90% ee (CHIRALPAK IA, *n*-hexane/*i*-PrOH = 97/3, detector: 240 nm, T = 25 °C, flow rate: 1 mL/min), t_1 (minor) = 8.31 min, t_2 (major) = 9.87 min.

***tert*-Butyl (*S*)-2-(benzo[*d*]oxazol-2-yl)-2-(2-oxo-2-phenylethyl)hexanoate (5ac)**

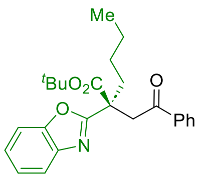

Reaction time: 2 h. (26.6 mg, 65%). **^{1}H NMR (400 MHz, CDCl$_3$)** δ 8.08 – 8.00 (m, 2H), 7.73 – 7.67 (m, 1H), 7.61 – 7.55 (m, 1H), 7.54 – 7.45 (m, 3H), 7.34 – 7.29 (m, 2H), 4.08 (d, *J* = 18.1 Hz, 1H), 3.93 (d, *J* = 18.1 Hz, 1H), 2.45 (m, 1H), 2.39 – 2.26 (m, 1H), 1.40 (s, 9H), 1.32 – 1.18 (m, 3H), 1.12 – 1.00 (m, 1H), 0.83 (t, *J* = 7.1 Hz, 3H). **^{13}C NMR (100 MHz, CDCl$_3$)** δ 196.5, 170.2, 166.5, 151.0, 141.0, 136.9, 133.4, 128.7, 128.2, 124.9, 124.3, 120.2, 110.8, 82.3, 51.3, 42.2, 34.3, 27.9, 26.9, 22.9, 14.0. **ESI-MS:** calculated [$C_{25}H_{29}NO_4$ + Na]$^+$: 430.1989, found: 430.1997. $[\alpha]^{20}_D$ = +13.0 (c = 0.83, CH_2Cl_2). The product was analyzed by HPLC to determine the enantiomeric excess: 90% ee (CHIRALPAK IA, *n*-hexane/*i*-PrOH = 95/5, detector: 254 nm, T = 25 °C, flow rate: 1 mL/min), t_1 (minor) = 5.97 min, t_2 (major) = 7.10 min.

***tert*-Butyl (*S*)-2-(benzo[*d*]oxazol-2-yl)-2-(2-oxo-2-phenylethyl)pent-4-enoate (5ad)**

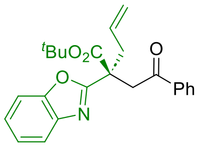

Reaction time: 2 h. (22.7 mg, 58%). **^{1}H NMR (400 MHz, CDCl$_3$)** δ 8.06 – 8.00 (m, 2H), 7.77 – 7.69 (m, 1H), 7.61 – 7.51 (m, 2H), 7.51 – 7.43 (m, 2H), 7.38 – 7.29 (m, 2H), 5.68 – 5.53 (m, 1H), 5.09 – 4.96 (m, 2H), 4.06 (d, *J* = 18.2 Hz, 1H), 3.92 (d, *J* = 18.2 Hz, 1H), 3.26 (dd, *J* = 14.2, 6.8 Hz, 1H), 3.11 (dd, *J* = 14.2, 8.3 Hz, 1H), 1.41 (s, 9H). **^{13}C NMR (101 MHz, CDCl$_3$)** δ 196.5, 169.5, 165.8, 151.0, 141.0, 136.9, 133.4, 132.7, 128.7, 128.2, 125.1, 124.4, 120.2, 119.9, 110.8, 82.6, 51.0, 41.8, 38.7, 27.9. **ESI-MS:** calculated [$C_{24}H_{25}NO_4$ + Na]$^+$: 414.1676, found: 414.1686. $[\alpha]^{20}_D$ = -16.4 (c = 1.00, CH_2Cl_2). The product was analyzed by HPLC to determine the enantiomeric excess: 90% ee (CHIRALPAK IA, *n*-hexane/*i*-PrOH = 95/5, detector: 240 nm, T = 25 °C, flow rate: 1 mL/min), t_1 (minor) = 6.12 min, t_2 (major) = 7.75 min.

***tert*-Butyl (*S*)-2-(benzo[*d*]oxazol-2-yl)-4-oxo-2-phenethyl-4-phenylbutanoate (5ae)**

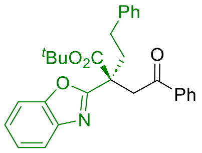

Reaction time: 2 h. (34.0 mg, 75%). **^{1}H NMR (500 MHz, CDCl$_3$)** δ 8.07 – 7.96 (m, 2H), 7.77 – 7.65 (m, 1H), 7.61 – 7.55 (m, 1H), 7.55 – 7.50 (m, 1H), 7.47 (m, 2H), 7.39 – 7.29 (m, 2H), 7.22 – 7.16 (m, 2H), 7.16 – 7.07 (m, 3H), 4.13 (d, *J* = 18.0 Hz, 1H), 3.98 (d, *J* = 18.0 Hz, 1H), 2.86 – 2.71 (m, 1H), 2.70 – 2.57 (m, 2H), 2.55 – 2.45 (m, 1H), 1.43 (s, 9H). **^{13}C NMR (125 MHz, CDCl$_3$)** δ 196.4, 169.9, 166.1, 151.0, 141.1, 141.0, 136.9, 133.4, 128.7, 128.5, 128.5, 128.2, 126.1, 125.0, 124.4, 120.2, 110.8, 82.5, 51.4, 42.5, 36.4, 31.3, 27.9. **ESI-MS:** calculated [$C_{29}H_{29}NO_4$ + Na]$^+$: 478.1989, found: 478.2000. $[\alpha]^{20}_D$ = +13.6 (c = 1.00, CH_2Cl_2). The product was analyzed by HPLC to determine the enantiomeric excess: 90% ee (CHIRALPAK IA, *n*-hexane/*i*-PrOH = 95/5, detector: 254 nm, T = 25 °C, flow rate: 1 mL/min), t_1 (minor) = 7.58 min, t_2 (major) = 8.16 min.

***tert*-Butyl (*S*)-2-(benzo[*d*]oxazol-2-yl)-5-methoxy-2-(2-oxo-2-phenylethyl)pentanoate (5af)**

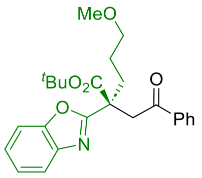

Reaction time: 2 h. (22.7 mg, 54%). **^{1}H NMR (500 MHz, CDCl$_3$)** δ 8.05 – 8.01 (m, 2H), 7.72 – 7.67 (m, 1H), 7.60 – 7.55 (m, 1H), 7.53 – 7.50 (m, 1H), 7.49 – 7.44 (m, 2H), 7.33 – 7.28 (m, 2H), 4.08 (d, *J* = 18.0 Hz, 1H), 3.93 (d, *J* = 18.0 Hz, 1H), 3.32 (t, *J* = 6.6 Hz, 2H), 3.23 (s, 3H), 2.56 – 2.45 (m, 1H), 2.44 – 2.31 (m, 1H), 1.63 – 1.51 (m, 1H), 1.46 – 1.42 (m, 1H). 1.41 (s, 9H). **^{13}C NMR (125 MHz, CDCl$_3$)** δ 196.4, 170.0, 166.2, 151.0, 141.0, 136.9, 133.4, 128.7, 128.2, 125.0, 124.3, 120.2, 110.8, 82.4, 72.5, 58.6, 51.0, 42.4, 31.4, 27.9, 25.1. **ESI-MS:** calculated [$C_{29}H_{29}NO_5$ + H]$^+$: 424.2118, found: 424.2118. $[\alpha]^{20}_D$

= +17.0 (c = 0.70, CH_2Cl_2). The product was analyzed by HPLC to determine the enantiomeric excess: 90% ee (CHIRALPAK IC, *n*-hexane/*i*-PrOH = 95/5, detector: 254 nm, T = 25 °C, flow rate: 1 mL/min), t_1 (major) = 11.54 min, t_2 (major) = 13.89 min.

***tert*-Butyl (*S*)-2-(benzo[*d*]oxazol-2-yl)-5-((tert-butyldimethylsilyl)oxy)-2-(2-oxo-2-phenylethyl)pentanoate (5ag)**

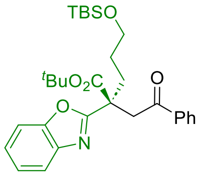

Reaction time: 2 h. (32.9 mg, 63%). **^{1}H NMR (500 MHz, $CDCl_3$)** δ 8.06 – 8.01 (m, 2H), 7.70 – 7.67 (m, 1H), 7.61 – 7.55 (m, 1H), 7.53 – 7.50 (m, 1H), 7.50 – 7.45 (m, 2H), 7.33 – 7.29 (m, 2H), 4.09 (d, *J* = 18.0 Hz, 1H), 3.92 (d, *J* = 18.0 Hz, 1H), 3.55 (t, *J* = 6.4 Hz, 2H), 2.52 – 2.43 (m, 1H), 2.39 – 2.32 (m, 1H), 1.56 – 1.46 (m, 1H), 1.41 (s, 9H), 1.36 – 1.27 (m, 1H), 0.82 (s, 9H), -0.03 (s, 3H), -0.04 (s, 3H). **^{13}C NMR (125 MHz, $CDCl_3$)** δ 196.4, 170.0, 166.3, 151.0, 141.0, 136.9, 133.4, 128.7, 128.2, 124.9, 124.3, 120.2, 110.8, 82.3, 62.9, 51.1, 42.4, 31.2, 28.2, 27.9, 26.0, 18.4, -5.3. **ESI-MS:** calculated $[C_{30}H_{41}NO_5Si + Na]^+$: 546.2646, found: 546.2659. $[\alpha]^{20}_D$ = +17.8 (c = 0.83, CH_2Cl_2). The product was analyzed by HPLC to determine the enantiomeric excess: 93% ee (CHIRALPAK IC, *n*-hexane/*i*-PrOH = 98/2, detector: 254 nm, T = 25 °C, flow rate: 1 mL/min), t_1 (major) = 5.64 min, t_2 (minor) = 9.45 min.

***tert*-Butyl (*S*)-2-(benzo[*d*]oxazol-2-yl)-2-(cyclopropylmethyl)-4-oxo-4-phenylbutanoate (5ah)**

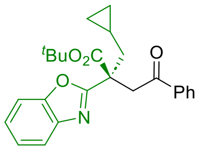

Reaction time: 2 h. (25.5 mg, 63%). **^{1}H NMR (500 MHz, $CDCl_3$)** δ 8.09 – 8.02 (m, 2H), 7.74 – 7.69 (m, 1H), 7.61 – 7.56 (m, 1H), 7.55 – 7.51 (m, 1H), 7.51 – 7.46 (m, 2H), 7.35 – 7.30 (m, 2H), 4.23 (d, *J* = 18.2 Hz, 1H), 4.17 (d, *J* = 18.2 Hz, 1H), 2.49 (dd, *J* = 14.5, 6.4 Hz, 1H), 2.25 (dd, *J* = 14.5, 7.5 Hz, 1H), 1.40 (s, 9H), 0.58 – 0.48 (m, 1H), 0.40 – 0.32 (m, 1H), 0.30 – 0.22 (m, 1H), -0.01 – -0.07 (m, 1H), -0.11 – -0.17 (m, 1H). **^{13}C NMR (125 MHz, $CDCl_3$)** δ 196.8, 170.0, 166.6, 150.9, 141.1, 136.9, 133.4, 128.8, 128.2, 125.0, 124.4, 120.1, 110.8, 82.3, 51.7, 42.1, 38.7, 27.9, 6.6, 4.8, 3.9. **ESI-MS:** calculated $[C_{25}H_{27}NO_4 + Na]^+$: 428.1832, found: 428.1840. $[\alpha]^{20}_D$ = +4.2 (c = 0.77, CH_2Cl_2). The product was analyzed by HPLC to determine the enantiomeric excess: 86% ee (CHIRALPAK IA, *n*-hexane/*i*-PrOH = 95/5, detector: 235 nm, T = 25 °C, flow rate: 1 mL/min), t_1 (minor) = 6.61 min, t_2 (major) = 8.67 min.

5. Synthetic transformations and applications

5.1 Synthetic transformations of 3n

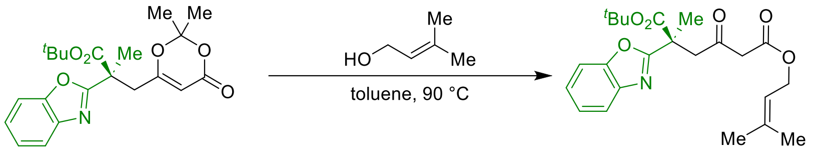

3n, 94% ee

6
86% yield, 94% ee

1-(*tert*-Butyl) 6-(3-methylbut-2-en-1-yl) (*S*)-2-(benzo[*d*]oxazol-2-yl)-2-methyl-4-oxohexanedioate (6). A Schlenk tube with a stirring bar was charged with compound **3n** (38.7 mg, 0.1 mmol), 3-methylbut-2-en-1-ol (10.0 equiv), and freshly dried toluene (1.0 mL). The resulting mixture was heated to 90 ℃ in an oil bath and stirred until the starting material was consumed (monitored by TLC). The reaction mixture was cooled to room temperature, directly put onto silica gel, and purified by silica gel chromatography to afford the desired product **6** (35.8 mg, 86%). **^{1}H NMR (400 MHz, CDCl$_3$)** δ 7.72 – 7.67 (m, 1H), 7.54 – 7.48 (m, 1H), 7.35 – 7.29 (m, 2H), 5.38 – 5.30 (m, 1H), 4.64 (d, *J* = 7.3 Hz, 2H), 3.59 (d, *J* = 18.1 Hz, 1H), 3.56 – 3.49 (m, 2H), 3.39 (d, *J* = 18.1 Hz, 1H), 1.79 (s, 3H), 1.75 (s, 3H), 1.70 (s, 3H), 1.41 (s, 9H). **^{13}C NMR (100 MHz, CDCl$_3$)** δ 199.1, 170.0, 167.0, 166.4, 151.0, 141.0, 140.1, 125.1, 124.4, 120.2, 118.0, 110.7, 82.6, 62.4, 49.8, 49.0, 47.4, 27.8, 25.9, 21.7, 18.2. **ESI-MS:** calculated $[C_{23}H_{29}NO_6 + Na]^+$: 438.1887, found: 438.1887. $[\alpha]^{20}_D$ = +11.9 (c = 0.90, CH_2Cl_2). The product was analyzed by HPLC to determine the enantiomeric excess: 94% ee (CHIRALPAK AS-H, *n*-hexane/*i*-PrOH = 95/5, detector: 268 nm, T = 25 °C, flow rate: 1 mL/min), t_1 (minor) = 7.99 min, t_2 (major) = 8.41 min.

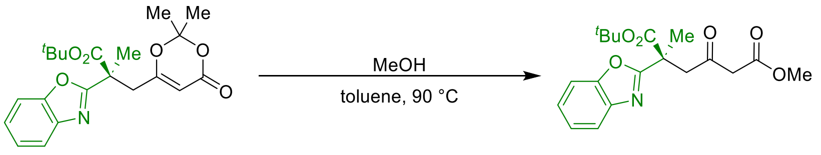

3n, 94% ee

7
95% yield, 94% ee

1-(*tert*-Butyl) 6-methyl (*S*)-2-(benzo[*d*]oxazol-2-yl)-2-methyl-4-oxohexanedioate (7). A Schlenk tube with a stirring bar was charged with compound **3n** (38.7 mg, 0.1 mmol), MeOH (10.0 equiv), and freshly dried toluene (1.0 mL). The resulting mixture was heated to 90 ℃ in an oil bath and stirred until the starting material was consumed (monitored by TLC). The reaction mixture was cooled to room temperature, directly put onto silica gel, and purified by silica gel chromatography to afford the desired product **7** (34.2 mg, 95%). **^{1}H NMR (400 MHz, CDCl$_3$)** δ 7.74 – 7.65 (m, 1H), 7.56 – 7.46 (m, 1H), 7.38 – 7.29 (m, 2H), 3.74 (s, 3H), 3.59 (d, *J* = 18.0 Hz, 1H), 3.56 (s, 2H), 3.39 (d, *J* = 18.0 Hz, 1H), 1.80 (s, 3H), 1.42 (s, 9H). **^{13}C NMR (100 MHz, CDCl$_3$)** δ 199.1, 170.0, 167.4, 166.4, 151.0, 141.0, 125.1, 124.4, 120.2, 110.7, 82.7, 52.5, 49.5, 49.1, 47.5, 27.8, 21.8. **ESI-MS:** calculated $[C_{19}H_{23}NO_6 + Na]^+$: 384.1418, found: 384.1418. $[\alpha]^{20}_D$ = +13.0 (c = 1.07, CH_2Cl_2). The product was analyzed by HPLC to determine the enantiomeric excess: 94% ee (CHIRALCEL OD-H, *n*-hexane/*i*-PrOH = 95/5, detector: 246 nm, T = 25 °C, flow rate: 1 mL/min), t_1 (minor) = 8.52 min, t_2 (major) = 12.66 min.

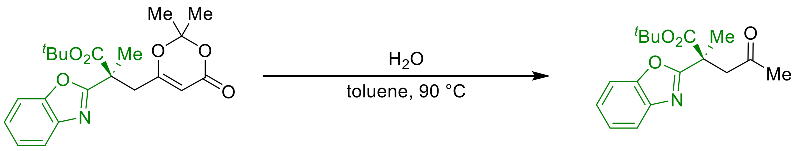

3n, 94% ee

8
86% yield, 94% ee

***tert*-Butyl (*S*)-2-(benzo[*d*]oxazol-2-yl)-2-methyl-4-oxopentanoate (8)**. A Schlenk tube with a stirring

bar was charged with compound **3n** (38.7 mg, 0.1 mmol), H_2O (10.0 equiv), and freshly dried toluene (1.0 mL). The resulting mixture was heated to 90 ℃ in an oil bath and stirred until the starting material was consumed (monitored by TLC). The reaction mixture was cooled to room temperature, directly put onto silica gel, and purified by silica gel chromatography to afford the desired product **8** (26.1 mg, 86%). **^{1}H NMR (400 MHz, $CDCl_3$)** δ 7.73 – 7.66 (m, 1H), 7.54 – 7.48 (m, 1H), 7.35 – 7.29 (m, 2H), 3.49 (d, *J* = 18.0 Hz, 1H), 3.28 (d, *J* = 18.0 Hz, 1H), 2.22 (s, 3H), 1.79 (s, 3H), 1.42 (s, 9H). **^{13}C NMR (100 MHz, $CDCl_3$)** δ 204.8, 170.3, 166.8, 151.0, 141.0, 125.0, 124.4, 120.1, 110.7, 82.4, 49.7, 47.3, 30.6, 27.8, 21.9. **ESI-MS:** calculated $[C_{27}H_{21}NO_4 + Na]^+$: 326.1363, found: 326.1363. $[\alpha]^{20}_D$ = +7.3 (c = 0.87, CH_2Cl_2). The product was analyzed by HPLC to determine the enantiomeric excess: 94% ee (CHIRALPAK IE, *n*-hexane/*i*-PrOH = 85/15, detector: 240 nm, T = 25 °C, flow rate: 1 mL/min), t_1 (minor) = 6.58 min, t_2 (major) = 10.17 min.

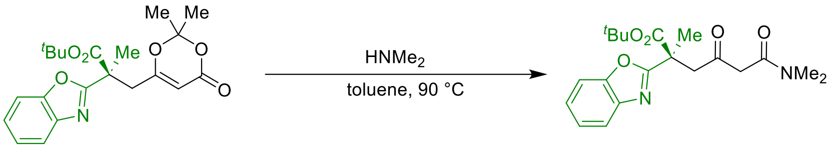

3n, 94% ee

9
94% yield, 94% ee

***tert*-Butyl (*S*)-2-(benzo[*d*]oxazol-2-yl)-6-(dimethylamino)-2-methyl-4,6-dioxohexanoate (9).** A Schlenk tube with a stirring bar was charged with compound **3n** (38.7 mg, 0.1 mmol), $HNMe_2$ (10.0 equiv), and freshly dried toluene (1.0 mL). The resulting mixture was heated to 90 ℃ in an oil bath and stirred until the starting material was consumed (monitored by TLC). The reaction mixture was cooled to room temperature, directly put onto silica gel, and purified by silica gel chromatography to afford the desired product **9** (35.0 mg, 94%). **^{1}H NMR (400 MHz, $CDCl_3$)** δ 7.70 – 7.63 (m, 1H), 7.56 – 7.48 (m, 1H), 7.34 – 7.28 (m, 2H), 3.66 (s, 2H), 3.54 (d, *J* = 17.9 Hz, 1H), 3.40 (d, *J* = 17.9 Hz, 1H), 3.01 (s, 3H), 2.97 (s, 3H), 1.81 (s, 3H), 1.42 (s, 9H). **^{13}C NMR (100 MHz, $CDCl_3$)** δ 200.8, 170.3, 166.6, 166.5, 151.0, 140.9, 125.0, 124.4, 120.0, 110.7, 82.5, 50.0, 48.9, 47.6, 38.0, 35.6, 27.8, 22.0. **ESI-MS:** calculated $[C_{20}H_{26}N_2O_5 + Na]^+$: 397.1734, found: 397.1734. $[\alpha]^{20}_D$ = +22.1 (c = 1.17, CH_2Cl_2). The product was analyzed by HPLC to determine the enantiomeric excess: 94% ee (CHIRALPAK AS-H, *n*-hexane/*i*-PrOH = 95/5, detector: 273 nm, T = 25 °C, flow rate: 1 mL/min), t_1 (major) = 7.62 min, t_2 (minor) = 8.42 min.

5.2 Synthetic transformations of 5a

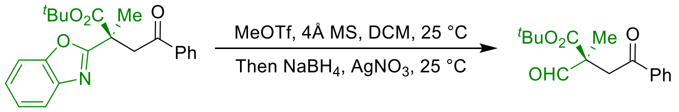

5a
94% ee

10
72% yield, 94% ee

***tert*-Butyl (*S*)-2-formyl-2-methyl-4-oxo-4-phenylbutanoate (10).** A solution of **5a** (36.5 mg, 0.1 mmol, 94% ee) and activated 4Å molecular sieves (150 mg) was vigorously stirred in DCM (1.5 mL) at 25 ℃ under an argon atmosphere for 20 minutes. Methyl trifluoromethanesulfonate (57 µL, 0.5 mol, 5.0 equiv) was then added, and the reaction mixture was stirred at 25 ℃ for 3 h. After complete consumption of the starting material **5a**, the reaction was concentrated to give the crude *N*-methylbenzoxazolium salt. The residue was dissolved in MeOH (2 mL), cooled to 0 ℃, and followed by the addition of $NaBH_4$ (0.12 mmol). After the starting material was consumed, as determined by TLC, the mixture was diluted with acetone, filtered through a pad of Celite, and concentrated to obtain the crude benzoxazolines. To the vigorously stirred solution of the crude benzoxazolines in CH_2Cl_2 (0.3 mL) and CH_3CN (1.5 mL), H_2O

(0.2 mL), and $AgNO_3$ (0.3 mmol) were added successively. The mixture was stirred at 25 °C and monitored by TLC. Subsequently, it was diluted with 1 M phosphate buffer at pH 7 (2 mL). After stirring for 15 minutes, the reaction mixture was further diluted with 1 M phosphate buffer at pH 7 (5 mL) and partially concentrated to remove CH_3CN. The resulting suspension was extracted with EtOAc, and the combined organic layers were dried over $MgSO_4$, filtered through a pad of Celite, and concentrated. The residue was purified by flash column chromatography on silica gel to afford **10** (20.0 mg, 72%). **^{1}H NMR (500 MHz, CDCl$_3$)** δ 10.15 (d, *J* = 0.8 Hz, 1H), 7.98 – 7.90 (m, 2H), 7.62 – 7.54 (m, 1H), 7.49 – 7.44 (m, 2H), 3.63 (d, *J* = 17.9 Hz, 1H), 3.51 (d, *J* = 17.9 Hz, 1H), 1.46 (s, 9H), 1.40 (s, 3H). **^{13}C NMR (125 MHz, CDCl$_3$)** δ 201.3, 197.2, 171.9, 136.1, 133.6, 128.8, 128.3, 82.4, 54.7, 45.2, 28.0, 20.4. **ESI-MS:** calculated $[C_{16}H_{20}O_4 + Na]^+$: 299.1254, found: 299.1251. $[\alpha]^{20}_D$ = -21.1 (c = 0.70, CH_2Cl_2). The product was analyzed by HPLC to determine the enantiomeric excess: 94% ee (CHIRALPAK IC, *n*-hexane/*i*-PrOH =95/5, detector: 240 nm, T = 25 °C, flow rate: 1 mL/min), t_1(major) = 18.14 min, t_2(minor) = 20.89 min.

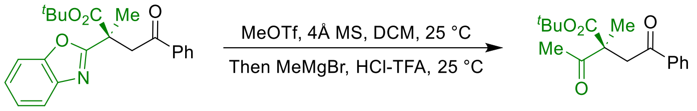

5a 94% ee → **11** 63% yield, 94% ee

***tert*-Butyl (*S*)-2-acetyl-2-methyl-4-oxo-4-phenylbutanoate (11).** A solution of **5a** (36.5 mg, 0.1 mmol, 94% ee) and activated 4Å molecular sieves (150 mg) was vigorously stirred in DCM (1.5 mL) at 25 ℃ under an argon atmosphere for 20 minutes. Methyl trifluoromethanesulfonate (57 µL, 0.5 mol, 5.0 equiv) was then added, and the reaction mixture was stirred at 25 ℃ for 3 h. After complete consumption of the starting material **5a**, the reaction was concentrated to give the crude *N*-methylbenzoxazolium salt. The residue was dissolved in tetrahydrofuran (2 mL) and cooled to 0 ℃. A solution of methylmagnesium bromide in tetrahydrofuran (1 M, 120 µL, 0.12 mmol, 1.2 equiv) was added dropwise by syringe. The reaction mixture was stirred for 10 minutes at 25 °C. Subsequently, aqueous hydrochloric acid solution (4 mL, 1 M) and trifluoroacetic acid (74 µL, 1 mmol, 10 equiv) were added successively. After stirring for 3 h at 50 °C, the biphasic reaction mixture was cooled to 25 °C and extracted with ethyl acetate. The combined organic layers were dried over $MgSO_4$, filtered, and concentrated under vacuum. The residue was purified by silica gel chromatography to afford the desired product **11** (18.3 mg, 63%). **^{1}H NMR (400 MHz, CDCl$_3$)** δ 8.01 – 7.93 (m, 2H), 7.60 – 7.54 (m, 1H), 7.49 – 7.43 (m, 2H), 3.67 – 3.52 (m, 2H), 2.33 (s, 3H), 1.54 (s, 3H), 1.44 (s, 9H). **^{13}C NMR (100 MHz, CDCl$_3$)** δ 206.2, 197.4 171.4, 136.8, 133.4, 128.7, 128.2 82.1 58.1, 44.7 27.8, 26.5, 20.8. **ESI-MS:** calculated $[C_{17}H_{22}O_4 + Na]^+$: 313.1410, found: 313.1404. $[\alpha]^{20}_D$ = -27.8 (c = 1.00, CH_2Cl_2). The product was analyzed by HPLC to determine the enantiomeric excess: 94% ee (CHIRALPAK IC, *n*-hexane/*i*-PrOH =95/5, detector: 240 nm, T = 25 °C, flow rate: 1 mL/min), t_1(minor) = 13.50 min, t_2(major) = 14.85 min.

5.3 Synthesis of (-)-Ethosuximide

Scale-up experiments for electrochemical alkylation reaction:

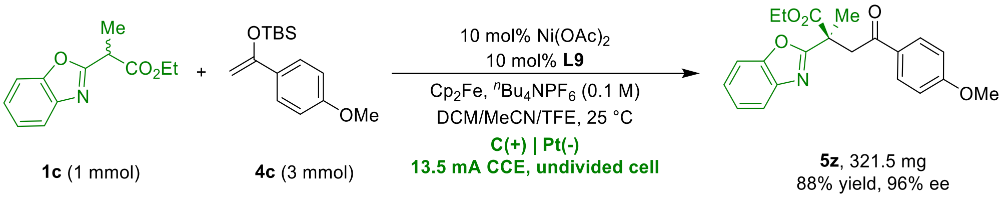

1c (1 mmol) + **4c** (3 mmol) → **5z**, 321.5 mg, 88% yield, 96% ee

A 25 mL reaction tube was charged with racemic benzoxazolyl acetate **1c** (1 mmol, 1 equiv), silyl enol ether **4c** (3 mmol, 3 equiv), nickel complex derived from **L9** (0.1 mmol, 0.1 equiv), Cp_2Fe (0.1 mmol,

0.1 equiv), $^{n}Bu_4NPF_6$ (0.1 M), DCM (7.0 mL), MeCN (3.5 mL), and TFE (10.5 mL) under argon atmosphere. The reaction tube was equipped with a carbon plate (2.0 cm × 2.0 cm × 3 mm) as anode and a platinum plate (2.0 cm × 2.0 cm × 0.2 mm) as cathode. The constant current (13.5 mA) electrolysis was carried out at 25 °C for 2 h until complete consumption of the substrate (monitored by TLC). The mixture was concentrated under reduced pressure, and purified by flash column chromatography on silica gel to afford the desired product **5z** (321.5 mg, 88%).

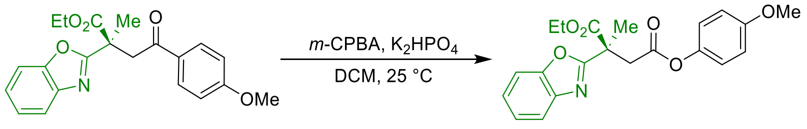

5z, 96% ee

12
86% yield, 96% ee

1-Ethyl 4-(4-methoxyphenyl) (*S*)-2-(benzo[*d*]oxazol-2-yl)-2-methylsuccinate (12). To a mixture of **5z** (36.7 mg, 0.1 mmol) and K_2HPO_4 (0.25 mmol, 2.5 equiv) in CH_2Cl_2 (2.0 mL) was added *m*-CPBA (0.6 mmol, 6.0 equiv). The mixture was stirred at room temperature for 24 h. Then, the reaction mixture was diluted with CH_2Cl_2, washed with 5% Na_2CO_3, and followed by the extraction with CH_2Cl_2. The organic layers were combined and dried over $MgSO_4$. The resulting solution was concentrated under reduced pressure, and purified by column chromatography on a silica gel using hexane/EtOAc to give the desired product **12** (33.1 mg, 86%). **^{1}H NMR (400 MHz, $CDCl_3$)** δ 7.79 – 7.67 (m, 1H), 7.56 – 7.49 (m, 1H), 7.37 – 7.30 (m, 2H), 7.02 – 6.94 (m, 2H), 6.89 – 6.81 (m, 2H), 4.23 (q, *J* = 7.0 Hz, 2H), 3.76 (s, 3H), 3.54 (d, *J* = 16.4 Hz, 1H), 3.44 (d, *J* = 16.4 Hz, 1H), 1.95 (s, 3H), 1.22 (t, *J* = 7.1 Hz, 3H). **^{13}C NMR (100 MHz, $CDCl_3$)** δ 171.1, 169.1, 165.6 157.4, 151.0, 144.0, 140.9, 125.3, 124.5, 122.3, 120.3, 114.5, 110.8, 62.4, 55.7, 47.1, 41.5, 21.9, 14.1. **ESI-MS:** calculated $[C_{21}H_{21}NO_6 + Na]^+$: 406.1261, found: 406.1261. $[\alpha]^{20}_{D}$ = +9.5 (c = 0.89, CH_2Cl_2). The product was analyzed by HPLC to determine the enantiomeric excess: 96% ee (CHIRALPAK IC, *n*-hexane/*i*-PrOH = 90/10, detector: 230 nm, T = 25 °C, flow rate: 1 mL/min), t_1 (minor) = 15.61 min, t_2 (major) = 17.86 min.

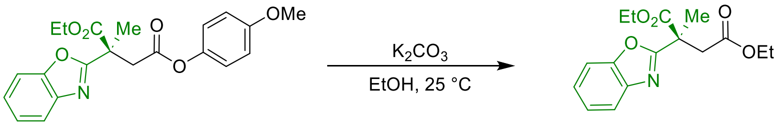

12, 96% ee

13
87% yield, 96% ee

Diethyl (*S*)-2-(benzo[*d*]oxazol-2-yl)-2-methylsuccinate (13). To a solution of **12** (38.3 mg, 0.1 mmol) in EtOH (1.0 mL) was added K_2CO_3 (0.12 mmol, 1.2 equiv), and the reaction mixture was stirred at room temperature for 3 h. After the starting material was consumed (monitored by TLC), the reaction was filtered through a pad of Celite. After concentration, the residue was purified by column chromatography on a silica gel using hexane/EtOAc to give the desired product **13** (26.4 mg, 87%). **^{1}H NMR (400 MHz, $CDCl_3$)** δ 7.81 – 7.65 (m, 1H), 7.58 – 7.47 (m, 1H), 7.39 – 7.28 (m, 2H), 4.22 (q, *J* = 7.1 Hz, 2H), 4.11 (q, *J* = 7.1 Hz, 2H), 3.32 (d, *J* = 16.3 Hz, 1H), 3.22 (d, *J* = 16.3 Hz, 1H), 1.87 (s, 3H), 1.22 (t, *J* = 7.1 Hz, 3H), 1.17 (t, *J* = 7.1 Hz, 3H). **^{13}C NMR (100 MHz, $CDCl_3$)** δ 171.2, 170.0, 165.9, 151.0, 141.0, 125.2, 124.4, 120.3, 110.7, 62.2, 60.8, 46.9, 41.4, 21.7, 14.1, 14.0. **ESI-MS:** calculated $[C_{16}H_{19}NO_5 + Na]^+$: 328.1155, found: 328.1155. $[\alpha]^{20}_{D}$ = +15.5 (c = 0.73, CH_2Cl_2). The product was analyzed by HPLC to determine the enantiomeric excess: 96% ee (CHIRALPAK IC, *n*-hexane/*i*-PrOH = 85/15, detector: 230 nm, T = 25 °C, flow rate: 1 mL/min), t_1 (minor) = 9.91 min, t_2 (major) = 12.08 min.

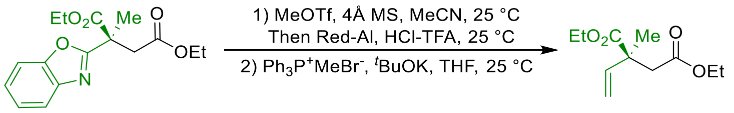

13, 96% ee

14, 56% yield (2 steps)

Diethyl (*S*)-2-methyl-2-vinylsuccinate (14). A solution of **13** (30.5 mg, 0.1 mmol) in MeCN (1.5 mL) containing activated 4Å molecular sieves (150 mg) was vigorously stirred at 25 ℃ under an argon atmosphere for 20 minutes. Methyl trifluoromethanesulfonate (57 µL, 0.5 mol, 5.0 equiv) was then added, and the reaction mixture was stirred at 25 ℃ for 4 h. After complete consumption of the starting material **13**, the reaction was filtered through a pad of Celite and concentrated to give the crude *N*-methylbenzoxazolium salt. The residue was dissolved in tetrahydrofuran (2 mL) and cooled to 0 ℃, followed by the addition of sodium bis(2-methoxyethoxy)aluminum hydride in toluene (Red-Al, 70% wt, 0.3 mmol, 3.0 equiv). The reaction mixture was stirred for 10 minutes at 0 °C. Subsequently, aqueous hydrochloric acid solution (2 mL, 1 M) and trifluoroacetic acid (74 µL, 1 mmol, 10 equiv) were added successively. After stirring for 12 h at 50 °C, the biphasic reaction mixture was cooled to 25 °C and extracted with ethyl acetate. The combined organic layers were dried over $MgSO_4$, filtered, and concentrated to give the crude aldehyde. To a 10 mL Schlenk tube was added methyl triphenylphosphonium bromide (0.3 mmol, 3.0 equiv), ^{t}BuOK (0.3 mmol, 3.0 equiv) and dry THF (1.5 mL), the mixture was stirred at room temperature for 1 h. Then, the diluted crude aldehyde in dry THF (0.5 mL) was added, and the reaction was stirred at room temperature overnight. The reaction was filtered through a pad of Celite. After concentration, the residue was purified by column chromatography on a silica gel using hexane/EtOAc to give the desired product **14** (12.0 mg, 56%). **^{1}H NMR (400 MHz, $CDCl_3$)** δ 6.06 (dd, *J* = 17.6, 10.7 Hz, 1H), 5.15 (d, *J* = 3.4 Hz, 1H), 5.11 (d, *J* = 3.1 Hz, 1H), 4.21 – 4.08 (m, 4H), 2.84 (d, *J* = 16.2 Hz, 1H), 2.60 (d, *J* = 16.2 Hz, 1H), 1.39 (s, 3H), 1.29 – 1.21 (m, 6H). **^{13}C NMR (100 MHz, $CDCl_3$)** δ 174.7, 171.0, 140.7, 114.2, 61.1, 60.6, 46.7, 43.0, 21.9, 14.3, 14.2. **ESI-MS:** calculated $[C_{11}H_{18}NO_4 + Na]^+$: 237.1097, found: 237.1096. $[\alpha]^{20}_D$ = +8.4 (c = 0.50, CH_2Cl_2).

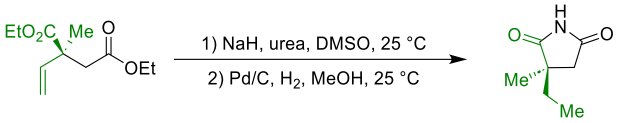

14

15, 71% yield, 96% ee (2 steps)
(-)-ethosuximide

(*R*)-3-ethyl-3-methylpyrrolidine-2,5-dione (15). Dry dimethylsulfoxide (1.5 mL), diethyl (*S*)-2-methyl-2-vinylsuccinate **14** (21.4 mg, 0.1 mmol), and urea (0.2 mmol, 2.0 equiv) were stirred and treated with a 60% dispersion of sodium hydride in mineral oil (0.3 mmol, 3.0 equiv). The solution was stirred for 24 h at room temperature. Then, the solution was diluted with ethyl acetate and acidified with HCl (6 M) at 0 °C. The aqueous phase was then extracted three times with ethyl acetate. The combined organic phase was dried over anhydrous $MgSO_4$ and concentrated to give the crude product. The crude product and Pd/C (5 wt%, 6.5 mg) were suspended in anhydrous methanol (3 mL), then H_2 gas was gently bubbled directly through the solution via a stainless-steel needle at room temperature. After stirring for 3 h, the reaction was complete (monitored by TLC). The solvent was removed in vacuo and the product was dissolved in ethyl acetate. The resulting solution was filtrated through a plug of celite and followed by the evaporation of the solvent. The crude material was purified by column chromatography to give compound **15** (10.0 mg, 71%). **^{1}H NMR (400 MHz, $CDCl_3$)** δ 8.56 (s, 1H), 2.67 (d, *J* = 18.4 Hz, 1H), 2.67 (d, *J* = 18.4 Hz, 1H), 2.47 (d, *J* = 18.4 Hz, 1H), 1.81 – 1.69 (m, 1H), 1.66 – 1.55 (m, 1H), 1.32 (s, 3H), 0.93 (t, *J* = 7.4 Hz, 3H). **^{13}C NMR (100 MHz, $CDCl_3$)** δ 183.5, 176.6, 45.8, 41.6, 31.0, 23.9, 8.8.

ESI-MS: calculated $[C_7H_{11}NO_2 + Na]^+$: 164.0682, found: 164.0681. $[\alpha]^{20}_D$ = -20.5 (c = 0.83, $CHCl_3$). The product was analyzed by HPLC to determine the enantiomeric excess: 96% ee (CHIRALPAK IC, *n*-hexane/*i*-PrOH = 90/10, detector: 253 nm, T = 25 °C, flow rate: 1 mL/min), t_1 (minor) = 17.75 min, t_2 (major) = 18.54 min.

6. Mechanistic studies.

6.1 Cyclic voltammetry studies

Cyclic voltammograms were recorded on a CHI 760E instrument using a glassy carbon disk working electrode (diameter, 3 mm), a Pt wire auxiliary electrode, an SCE reference electrode, and a scan rate of 100 mV/s.

Note: *Onset Potential* (E_{onset}) - The potential at which the extrapolation of the linear portion of the CV baseline and the extrapolation of the linear portion of the electrochemical wave intersect one another.

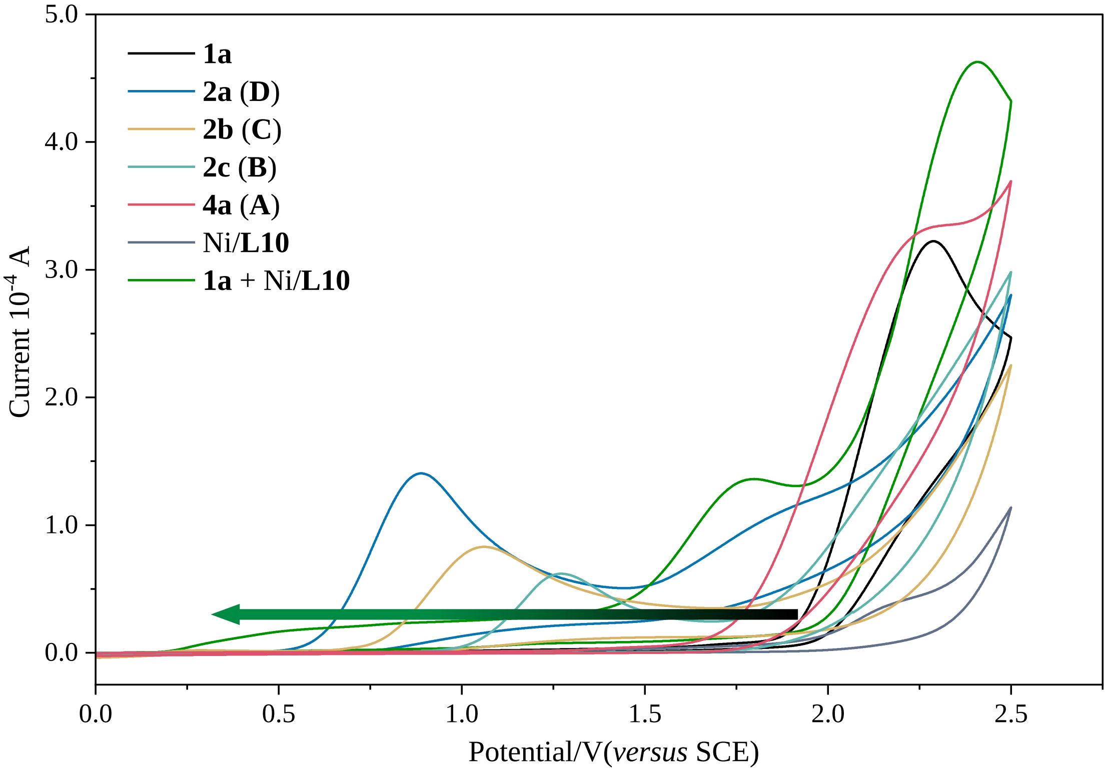

Fig. S1. Cyclic voltammogram was obtained by subjecting **1a** (33 mM), **2a** (16.7 mM), Ni/**L10** (3.3 mM), CF_3CH_2ONa (16.7 mM) in an electrolyte of $^nBu_4NBF_4$ (0.1 M) in DCM/TFE (2.0 mL/1.0 mL).

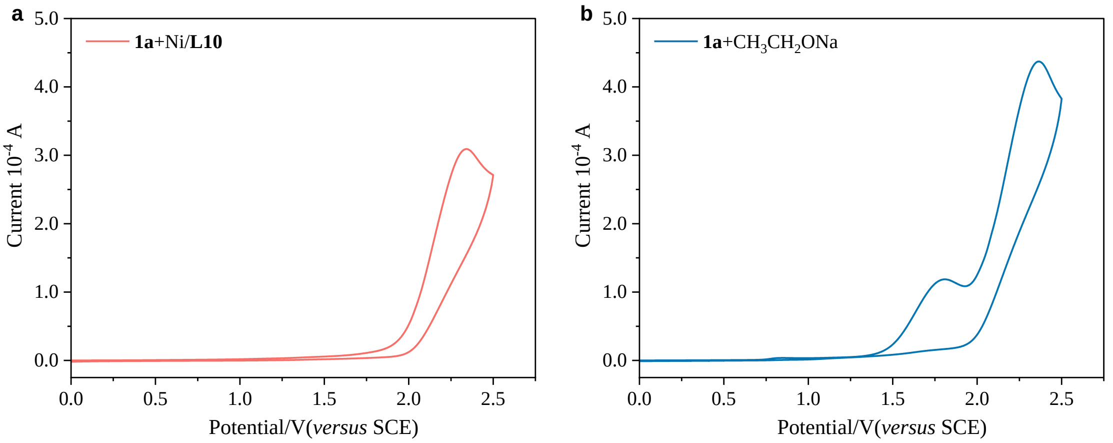

Fig. S2. Cyclic voltammogram was obtained by subjecting **1a** (33 mM), Ni/**L10** (3.3 mM), CF_3CH_2ONa (16.7 mM) in an electrolyte of $^nBu_4NBF_4$ (0.1 M) in DCM/TFE (2.0 mL/1.0 mL).

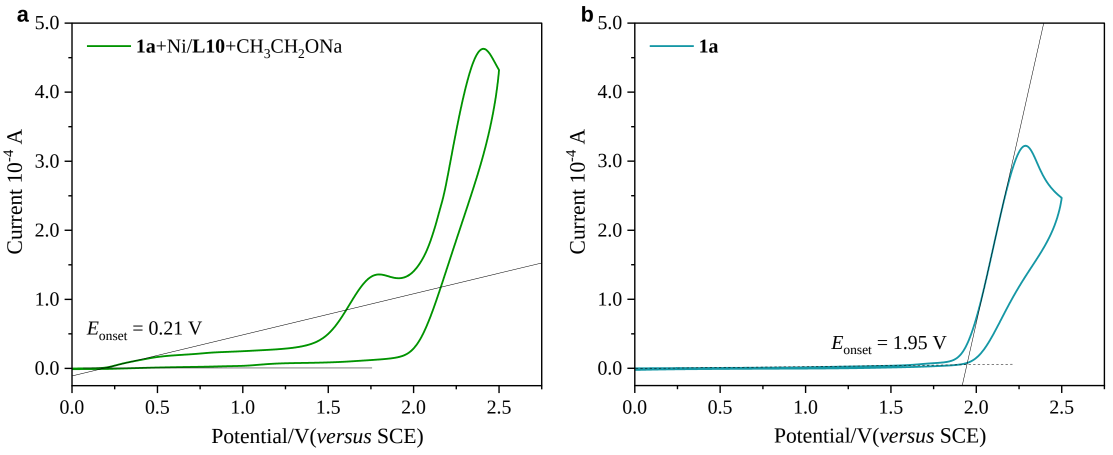

Fig. S3. Cyclic voltammogram was obtained by subjecting **1a** (33 mM), Ni/**L10** (3.3 mM), CF_3CH_2ONa (16.7 mM) in an electrolyte of $^{n}Bu_4NBF_4$ (0.1 M) in DCM/TFE (2.0 mL/1.0 mL). (**a**) The onset potential of the nickel-bound-enolate intermediate was measured approximately at +0.21 V (*versus* SCE). (**b**) The onset potential of **1a** was measured approximately at +1.95 V (*versus* SCE).

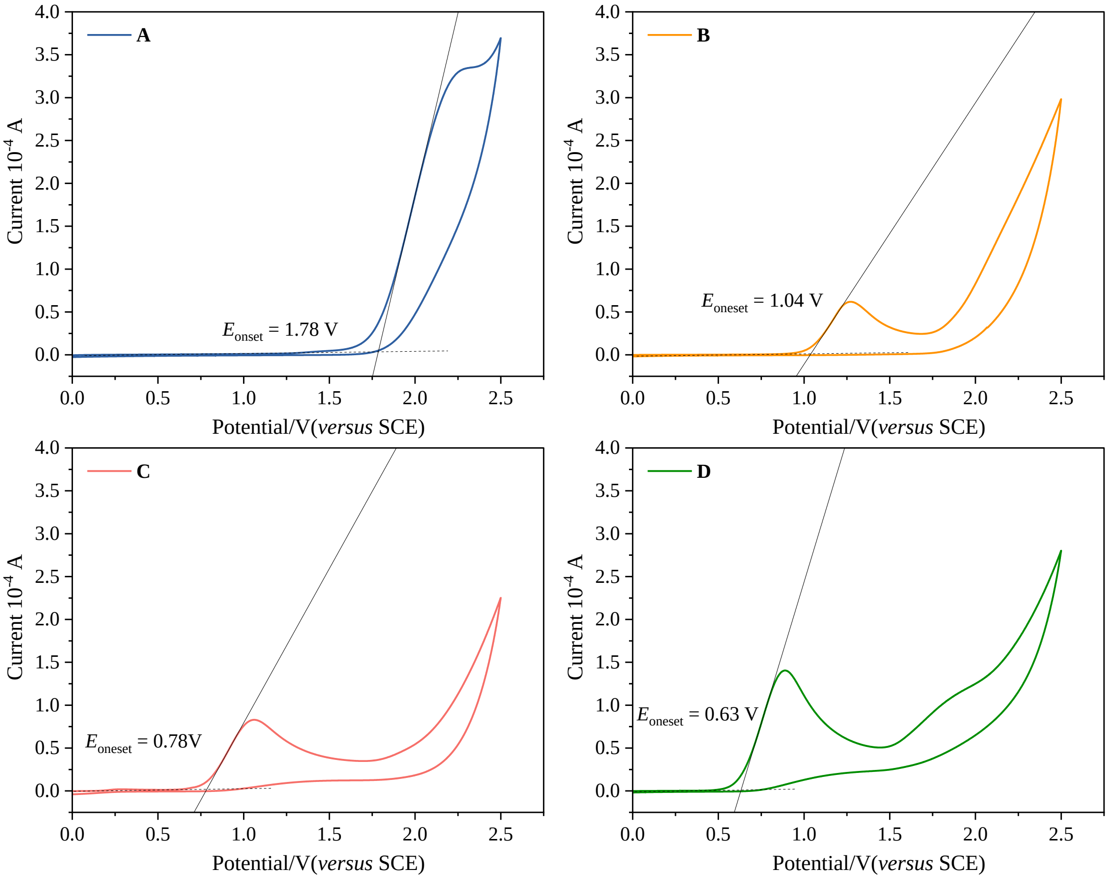

Fig. S4. Cyclic voltammogram was obtained by subjecting **A** (16.7 mM), **B** (16.7 mM), **C** (16.7 mM), and **D** (16.7 mM), in an electrolyte of $^{n}Bu_4NBF_4$ (0.1 M) in DCM/TFE (2.0 mL/1.0 mL). (**a**) The onset potential of **A** was measured approximately at +1.78 V (*versus* SCE). (**b**) The onset potential of **B** was measured approximately at +1.04 V (*versus* SCE). (**c**) The onset potential of **C** was measured approximately at +0.78 V (*versus* SCE). (**d**) The onset potential of **D** was measured approximately at +0.63 V (*versus* SCE).

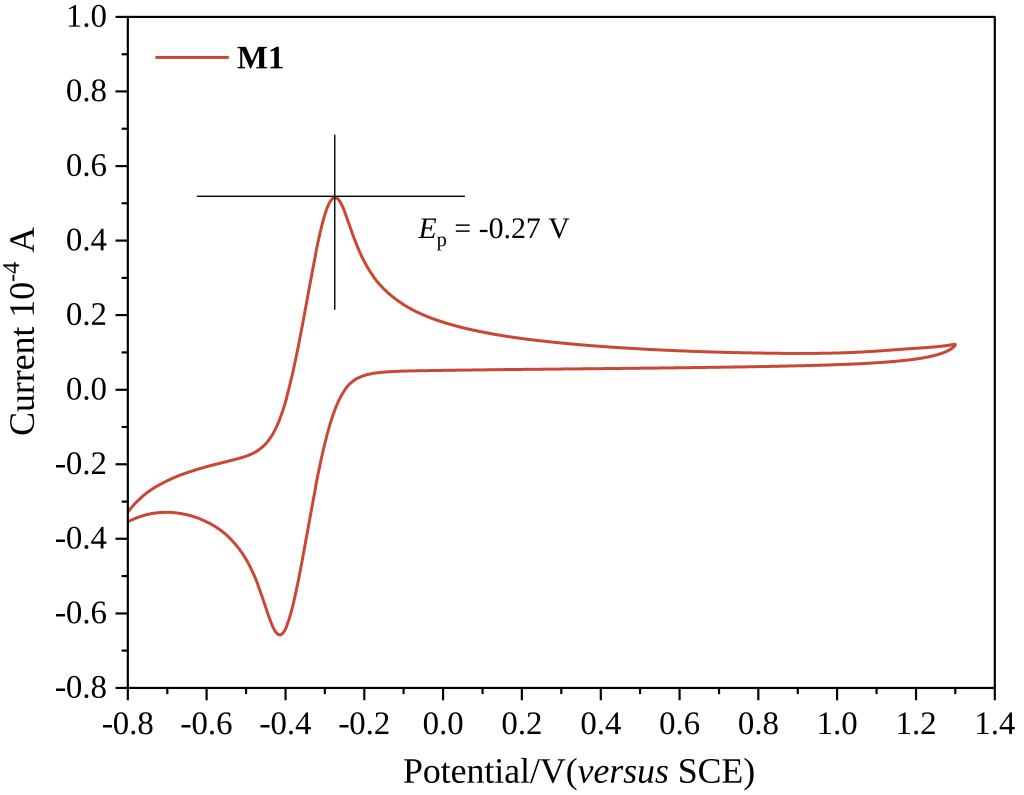

Fig. S5. Cyclic voltammogram was obtained by subjecting **M1** (3.3 mM) to an electrolyte of $^nBu_4NBF_4$ (0.1 M) in DCM/TFE (2.0 mL/1.0 mL).

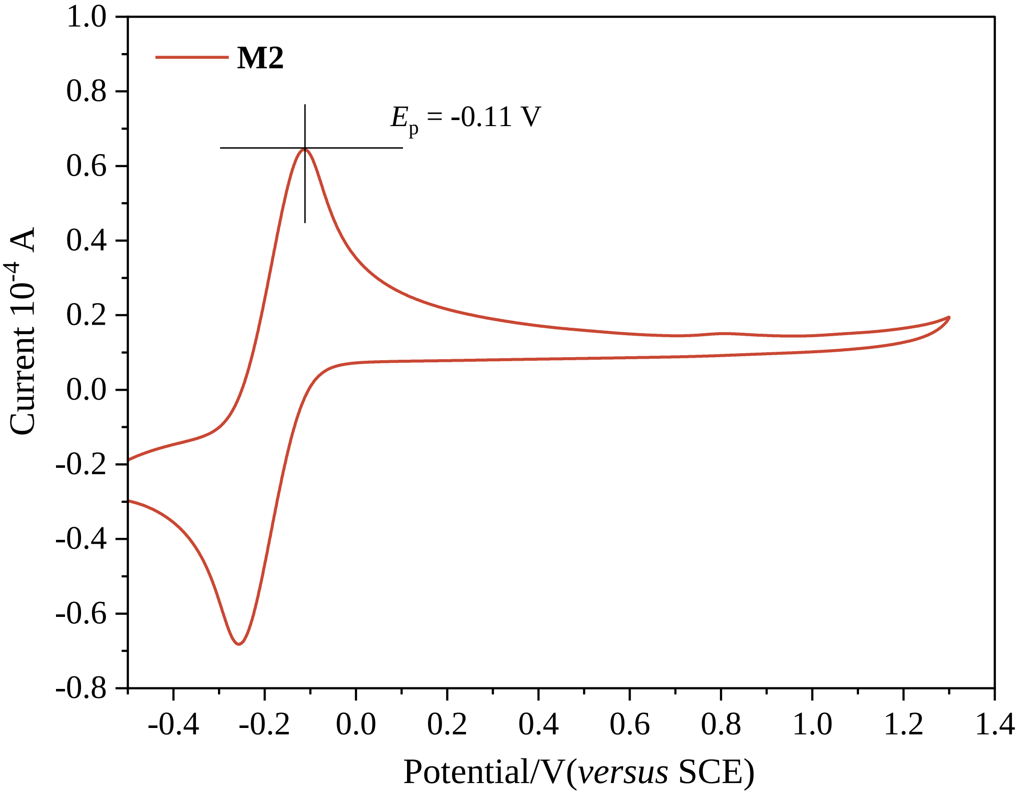

Fig. S6. Cyclic voltammogram was obtained by subjecting **M2** (3.3 mM) to an electrolyte of $^nBu_4NBF_4$ (0.1 M) in DCM/TFE (2.0 mL/1.0 mL).

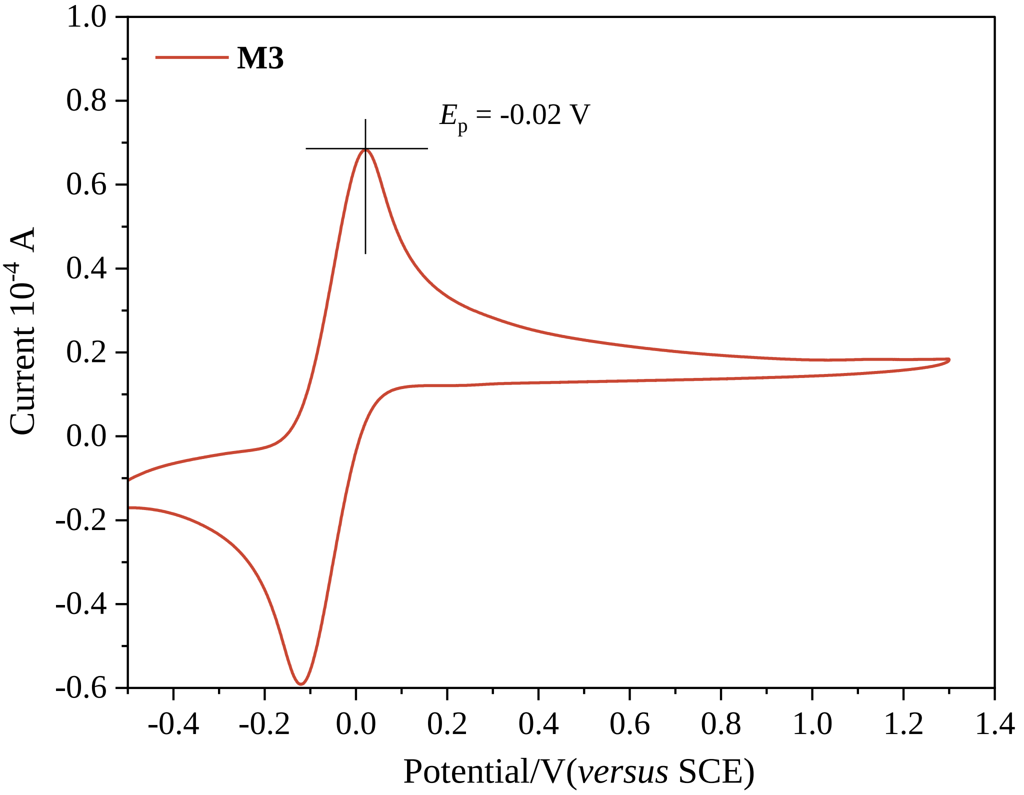

Fig. S7. Cyclic voltammogram was obtained by subjecting **M3** (3.3 mM) to an electrolyte of ${}^{n}Bu_4NBF_4$ (0.1 M) in DCM/TFE (2.0 mL/1.0 mL).

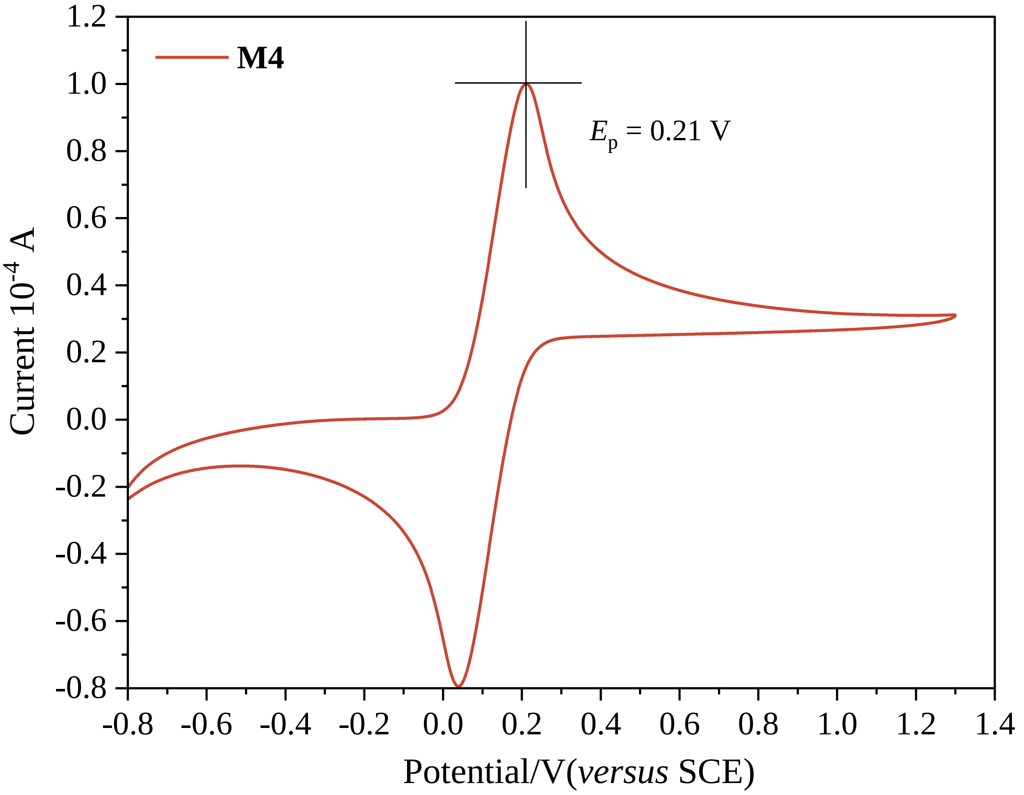

Fig. S8. Cyclic voltammogram was obtained by subjecting **M4** (3.3 mM) to an electrolyte of ${}^{n}Bu_4NBF_4$ (0.1 M) in DCM/TFE (2.0 mL/1.0 mL).

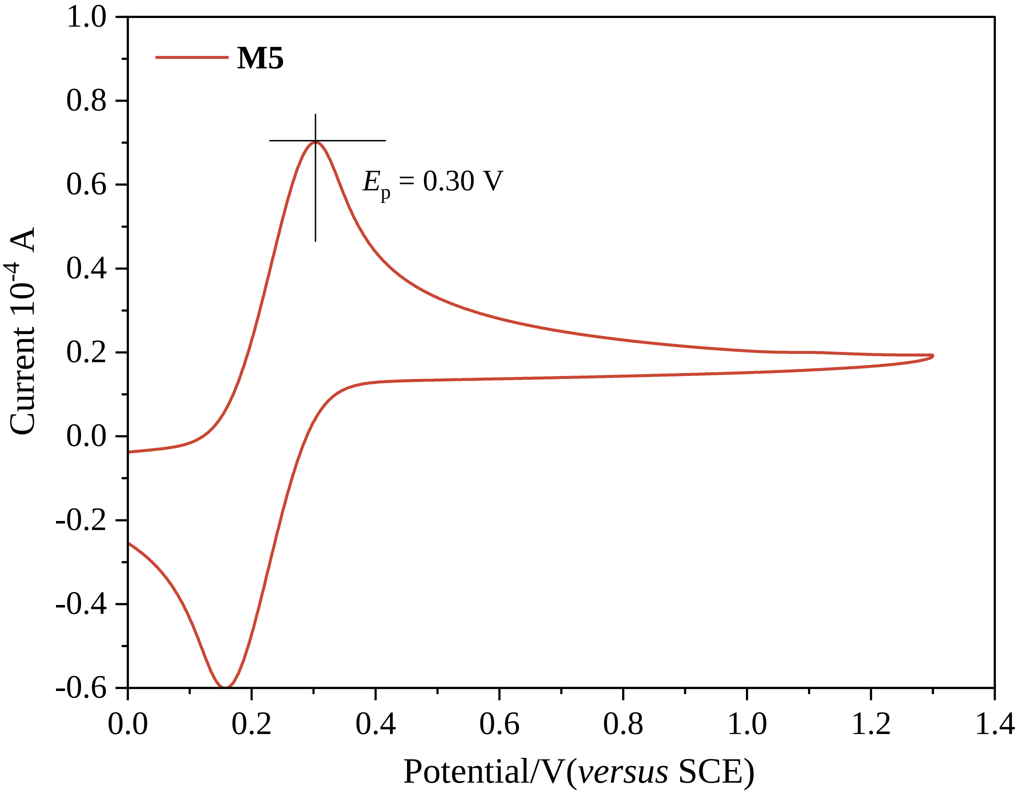

Fig. S9. Cyclic voltammogram was obtained by subjecting **M5** (3.3 mM) to an electrolyte of $^{n}Bu_4NBF_4$ (0.1 M) in DCM/TFE (2.0 mL/1.0 mL).

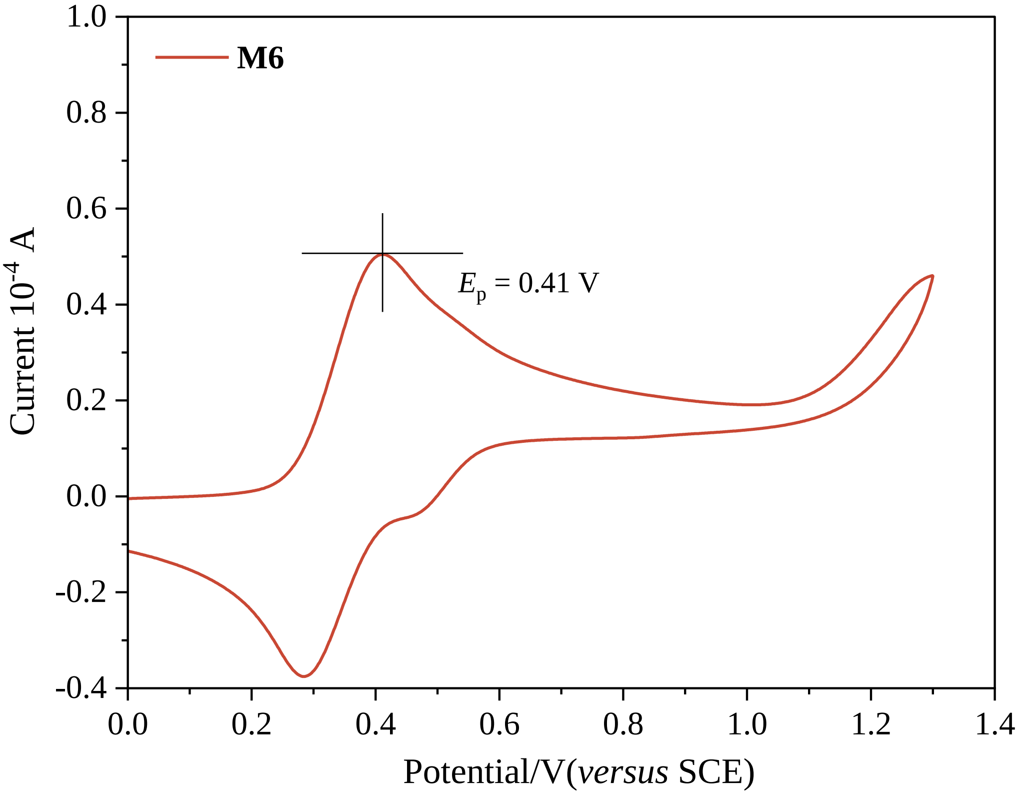

Fig. S10. Cyclic voltammogram was obtained by subjecting **M6** (3.3 mM) to an electrolyte of $^{n}Bu_4NBF_4$ (0.1 M) in DCM/TFE (2.0 mL/1.0 mL).

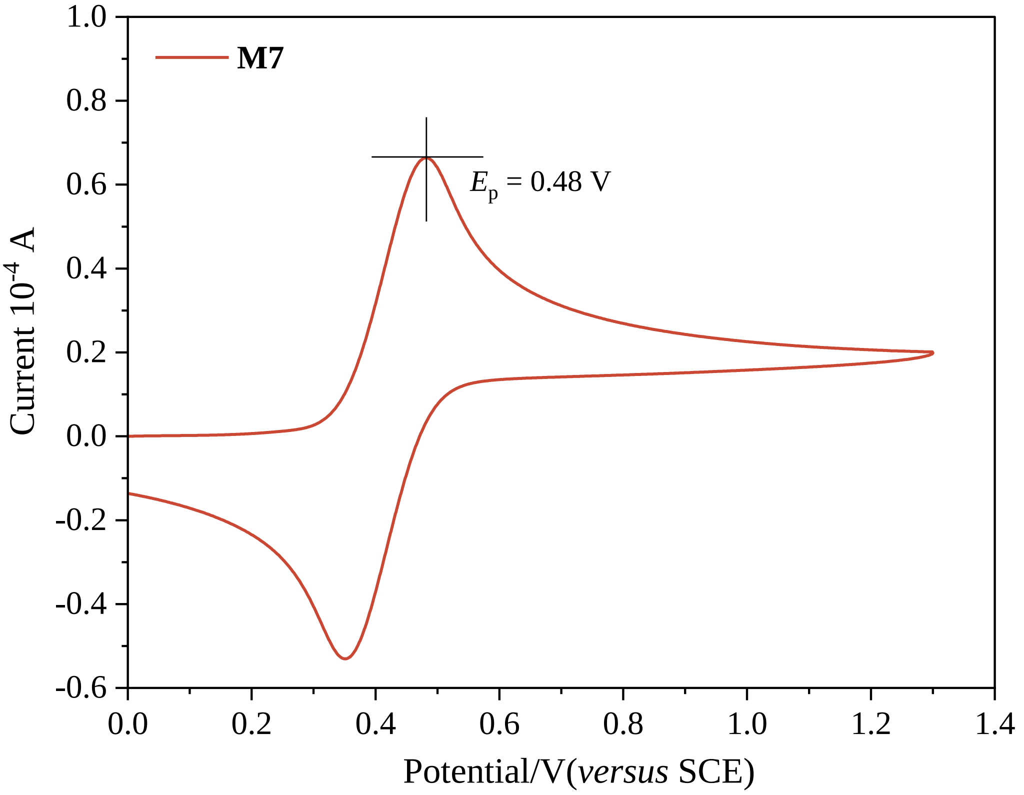

Fig. S11. Cyclic voltammogram was obtained by subjecting **M7** (3.3 mM) to an electrolyte of $^{n}Bu_4NBF_4$ (0.1 M) in DCM/TFE (2.0 mL/1.0 mL).

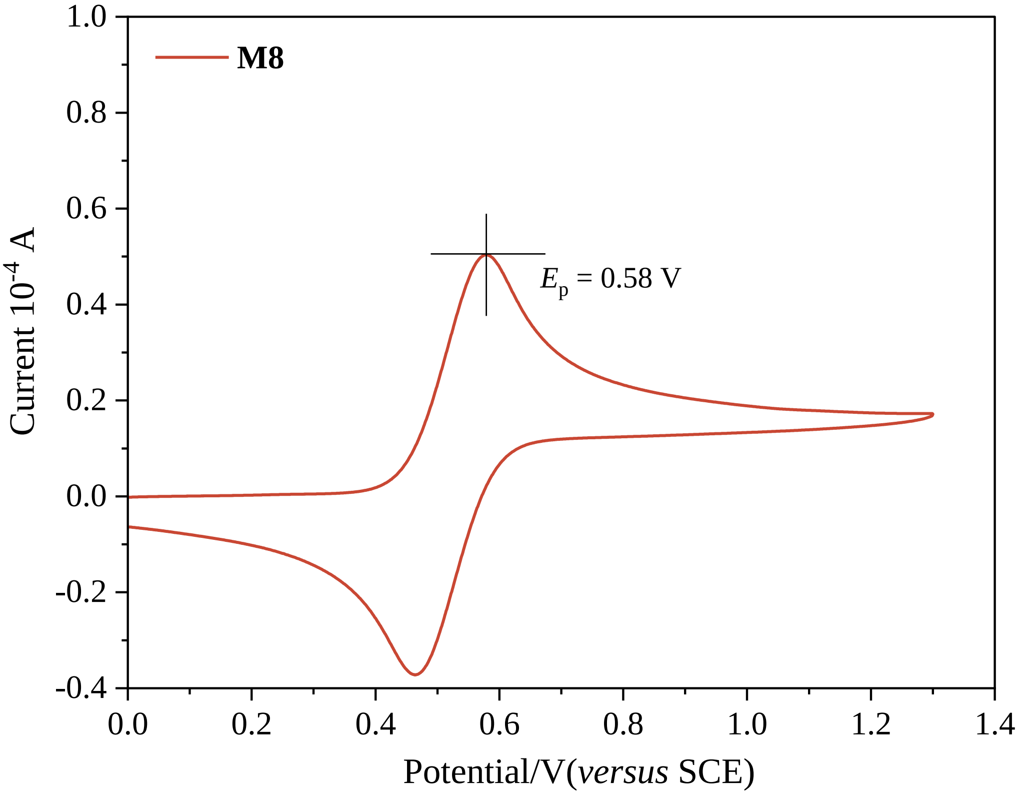

Fig. S12. Cyclic voltammogram was obtained by subjecting **M8** (3.3 mM) to an electrolyte of $^{n}Bu_4NBF_4$ (0.1 M) in DCM/TFE (2.0 mL/1.0 mL).

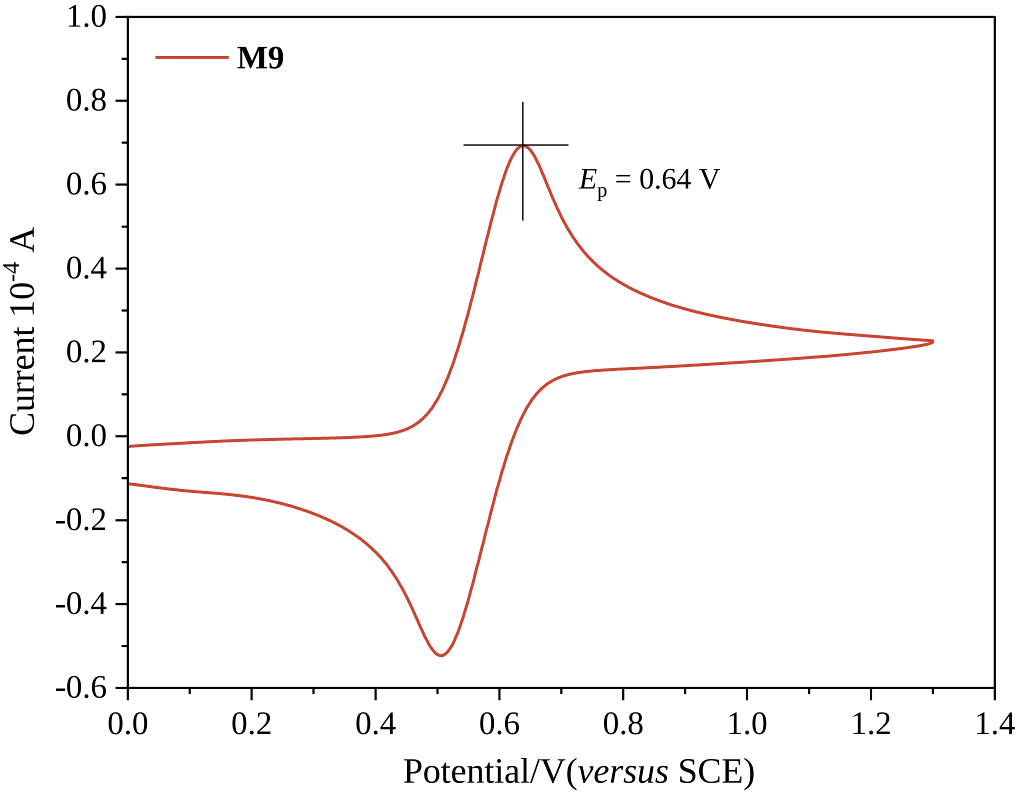

Fig. S13. Cyclic voltammogram was obtained by subjecting **M9** (3.3 mM) to an electrolyte of $^{n}Bu_4NBF_4$ (0.1 M) in DCM/TFE (2.0 mL/1.0 mL).

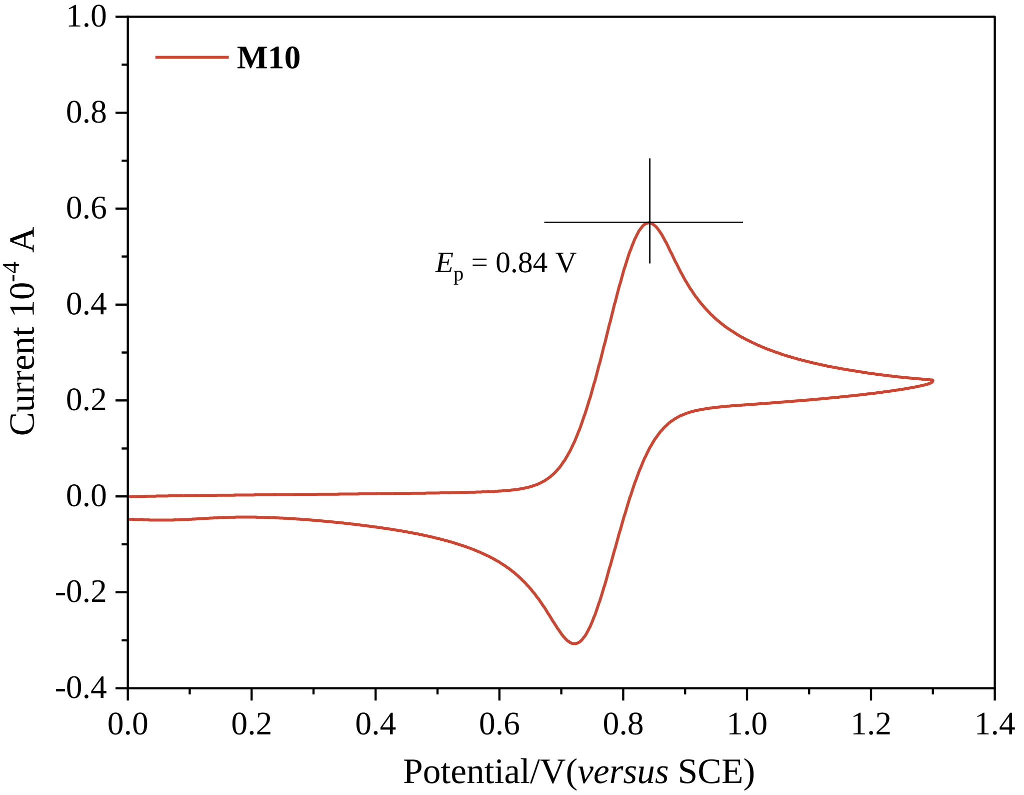

Fig. S14. Cyclic voltammogram was obtained by subjecting **M10** (3.3 mM) to an electrolyte of $^{n}Bu_4NBF_4$ (0.1 M) in DCM/TFE (2.0 mL/1.0 mL).

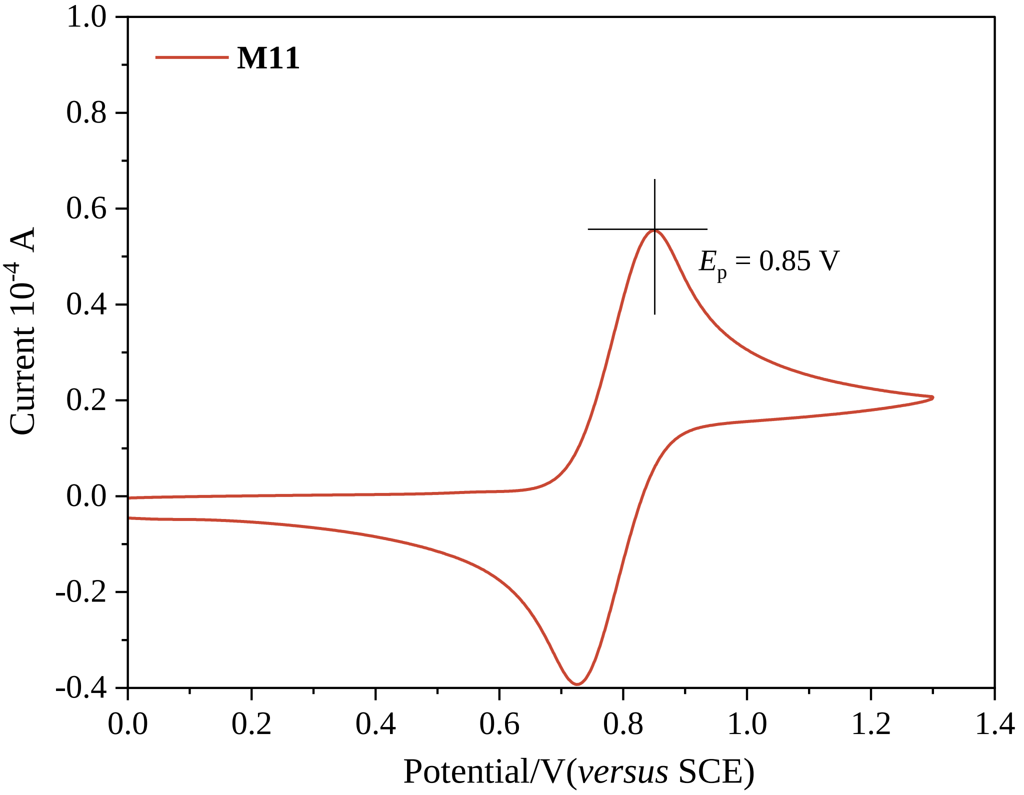

Fig. S15. Cyclic voltammogram was obtained by subjecting **M11** (3.3 mM) to an electrolyte of $^{n}Bu_4NBF_4$ (0.1 M) in DCM/TFE (2.0 mL/1.0 mL).

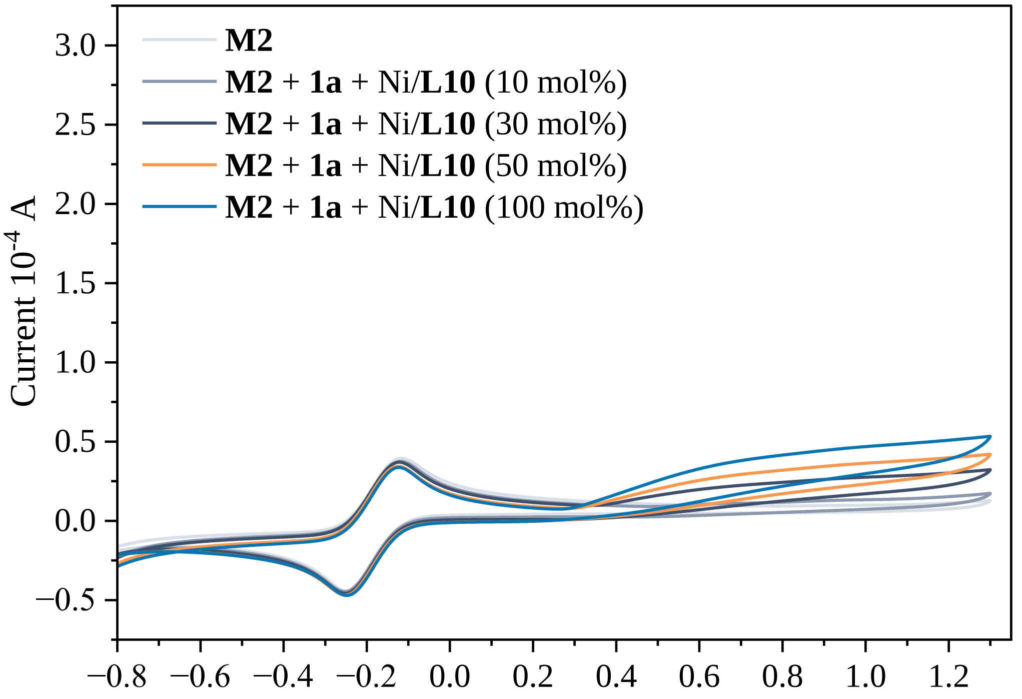

Fig. S16. Titration of nickel complex [**1a** + CF_3CH_2ONa + Ni/**L10** (3.3 mM to 33 mM)] to **M2** (3.3 mM) monitored by CV. Cyclic voltammogram of **M2** (3.3 mM), **1a** (33 mM), and CF_3CH_2ONa (16.7 mM) with different loadings of Ni/**L10** (10-100 mol%) with $^{n}Bu_4NBF_4$ (0.1 M) in DCM/TFE (2.0 mL/1.0 mL).

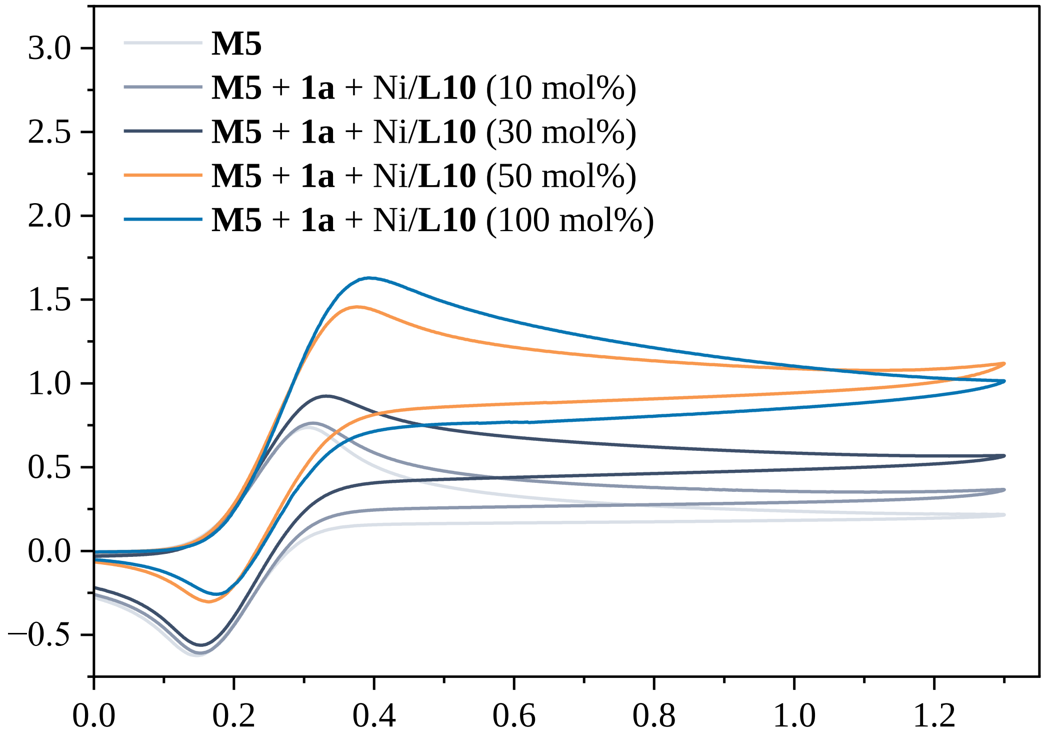

Fig. S17. Titration of nickel complex [**1a** + CF_3CH_2ONa + Ni/**L10** (3.3 mM to 33 mM)] to **M5** (3.3 mM) monitored by CV. Cyclic voltammogram of **M5** (3.3 mM), **1a** (33 mM), and CF_3CH_2ONa (16.7 mM) with different loadings of Ni/**L10** (10-100 mol%) with $^{n}Bu_4NBF_4$ (0.1 M) in DCM/TFE (2.0 mL/1.0 mL).

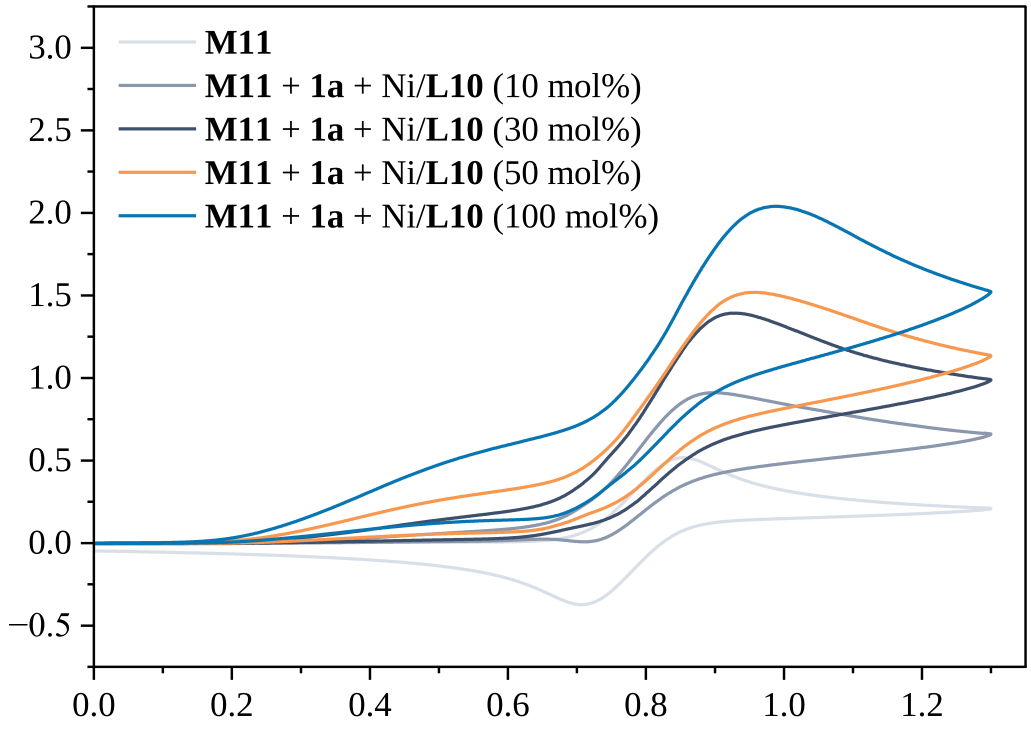

Fig. S18. Titration of nickel complex [**1a** + CF_3CH_2ONa + Ni/**L10** (3.3 mM to 33 mM)] to **M11** (3.3 mM) monitored by CV. Cyclic voltammogram of **M11** (3.3 mM), **1a** (33 mM), and CF_3CH_2ONa (16.7 mM) with different loadings of Ni/**L10** (10-100 mol%) with $^{n}Bu_4NBF_4$ (0.1 M) in DCM/TFE (2.0 mL/1.0 mL).

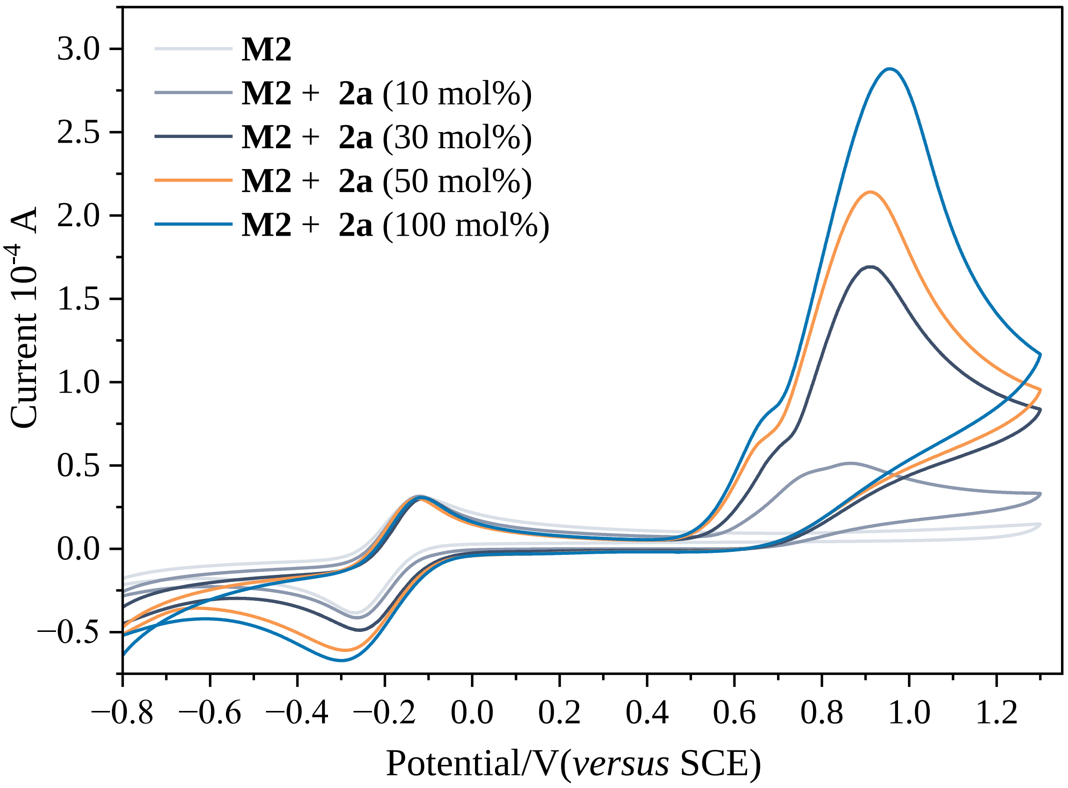

Fig. S19. Titration of **2a** (3.3 mM to 33 mM) to **M2** (3.3 mM) monitored by CV. Cyclic voltammogram of **M2** (3.3 mM) with different loadings of **2a** (10-100 mol%) with $^{n}Bu_4NBF_4$ (0.1 M) in DCM/TFE (2.0 mL/1.0 mL).

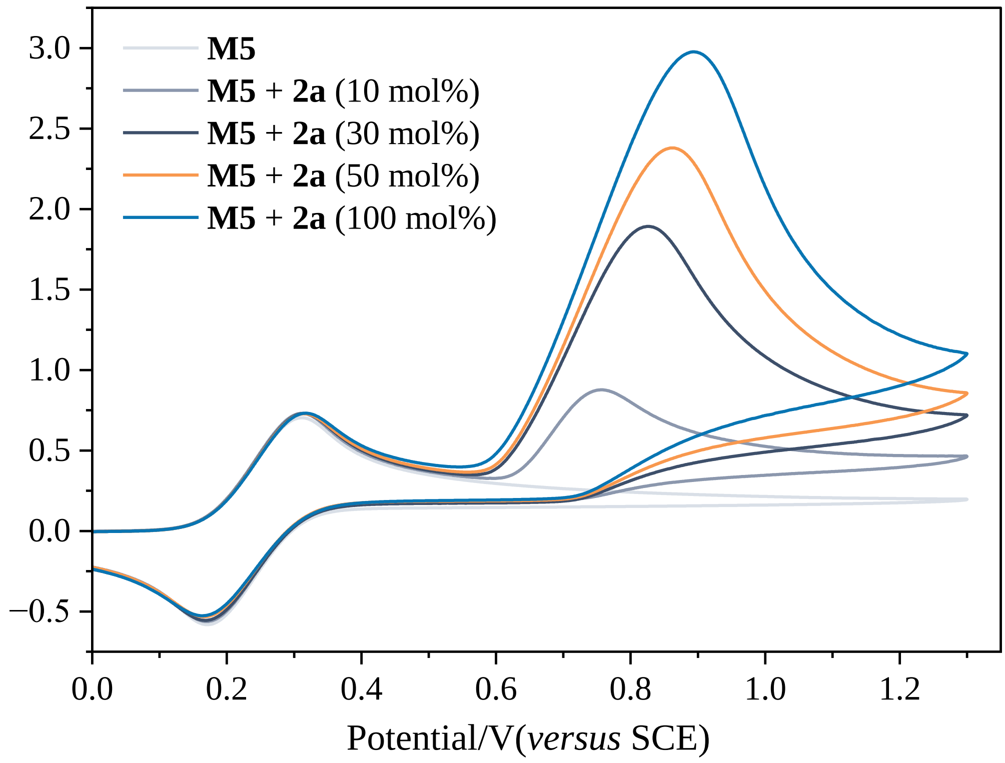

Fig. S20. Titration of **2a** (3.3 mM to 33 mM) to **M5** (3.3 mM) monitored by CV. Cyclic voltammogram of **M5** (3.3 mM) with different loadings of **2a** (10-100 mol%) with $^{n}Bu_4NBF_4$ (0.1 M) in DCM/TFE (2.0 mL/1.0 mL).

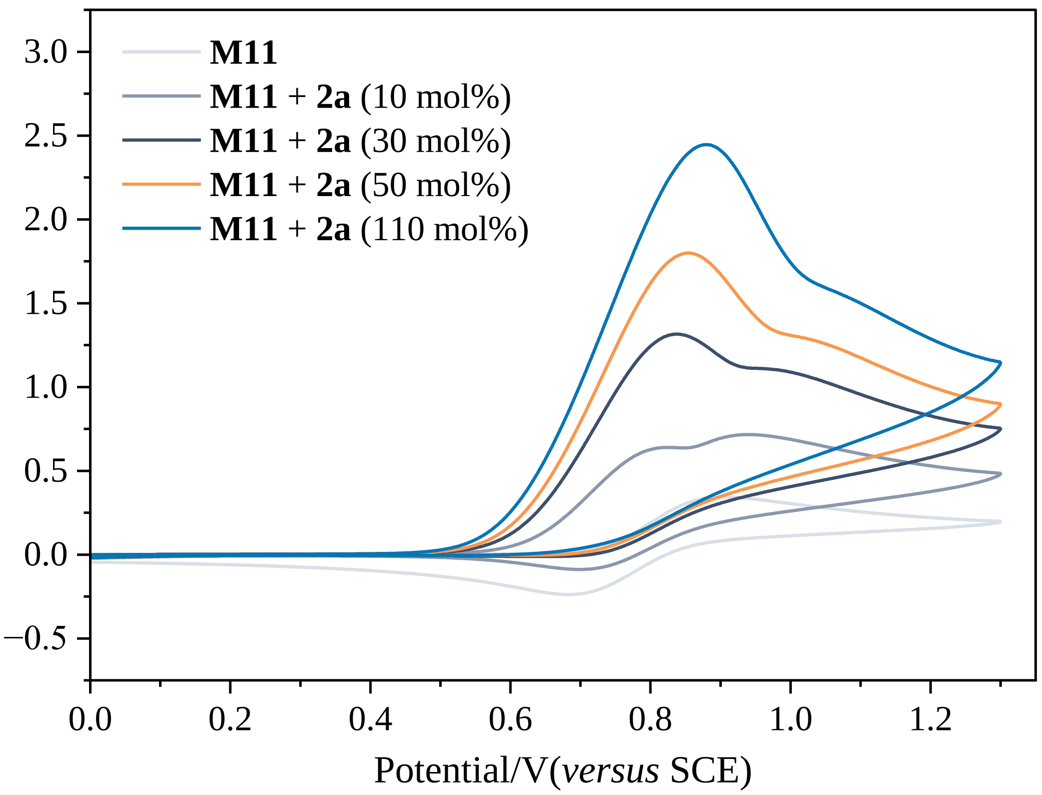

Fig. S21. Titration of **2a** (3.3 mM to 33 mM) to **M11** (3.3 mM) monitored by CV. Cyclic voltammogram of **M11** (3.3 mM) with different loadings of **2a** (10-100 mol%) with ^{n}Bu$_4$NBF$_4$ (0.1 M) in DCM/TFE (2.0 mL/1.0 mL).

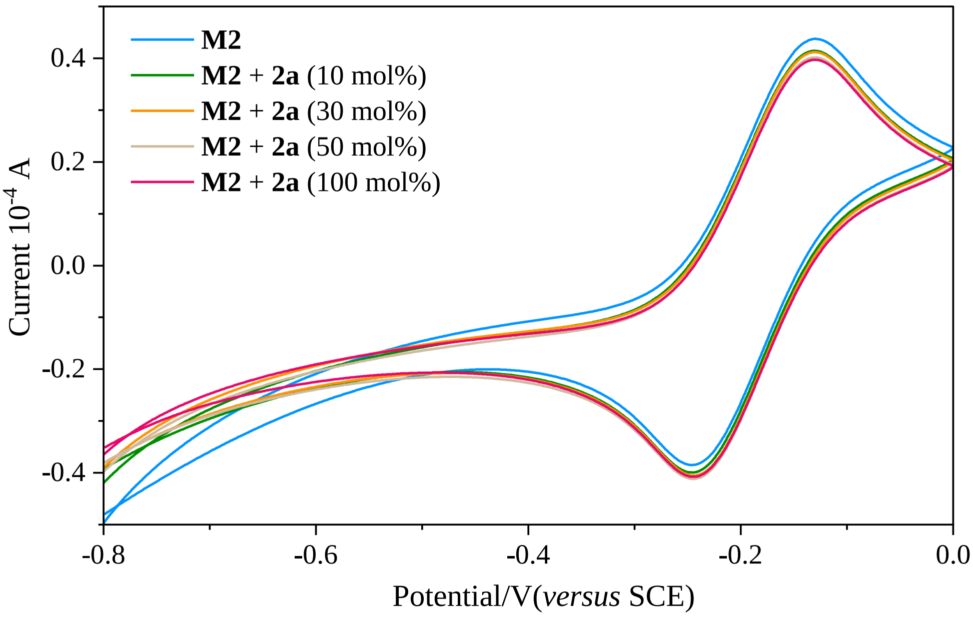

Fig. S22. Titration of **2a** (3.3 mM to 33 mM) to **M2** (3.3 mM) monitored by CV. Cyclic voltammogram of **M2** (3.3 mM) with different loadings of **2a** (10-100 mol%) with ^{n}Bu$_4$NBF$_4$ (0.1 M) in DCM/TFE (2.0 mL/1.0 mL).

6.2 The relationship between reaction yields and mediators with various oxidation potentials

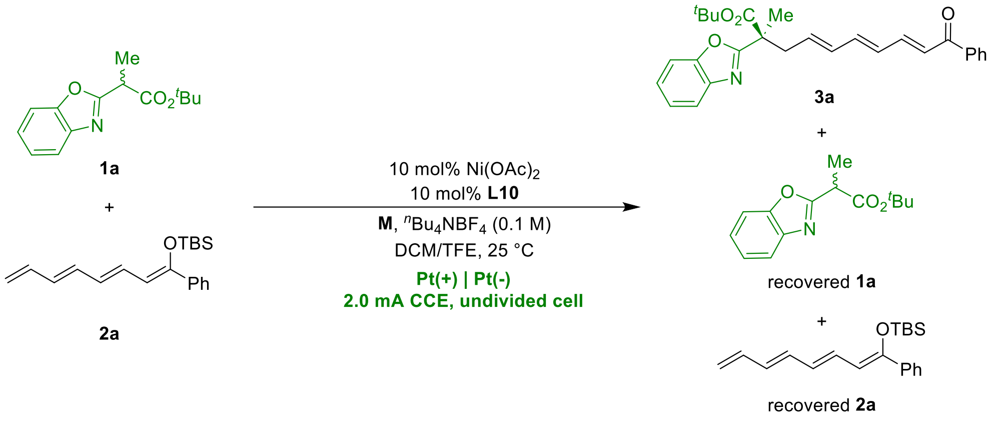

A 10 mL reaction tube was charged with racemic **1a** (0.1 mmol, 1 equiv), silyl enol ether **2a** (0.3 mmol, 3 equiv), nickel complex derived from (*R*,*R*)-**L10** (0.01 mmol, 0.1 equiv), ferrocenyl mediator **M** (0.01 mmol, 0.1 equiv), $^{n}Bu_4NBF_4$ (0.1 M), DCM (2 mL), and TFE (1.0 mL) under argon atmosphere. The reaction tube was equipped with a platinum plate (1.0 cm × 1.0 cm × 0.2 mm) as anode and a platinum plate (1.0 cm × 1.0 cm × 0.2 mm) as cathode. The constant current (2.0 mA) electrolysis was carried out at 25 °C for 5 h. After that the mixture was concentrated under reduced pressure, and purified by flash column chromatography on silica gel to afford the desired product **3a**, the recovered **1a**, and the recovered **2a**.

Table S8. The yield of product **3a**, recovered **1a**, and recovered **2a** corresponding to different ferrocenyl mediators **M**.

	Peak potential of **M** (*vs* SCE) (E_p)	yield of **3a** (%)	yield of recovered **1a** (%)	yield of recovered **2a** (%)
**M1**	-0.274	0	95	71
**M2**	-0.113	0	81	68
**M3**	0.021	9	75	64
**M4**	0.211	56	22	46
**M5**	0.303	78	0	41
**M6**	0.412	75	0	23
**M7**	0.481	19	71	8
**M8**	0.578	13	75	2
**M9**	0.639	0	85	2
**M10**	0.842	0	89	2
**M11**	0.850	0	93	2

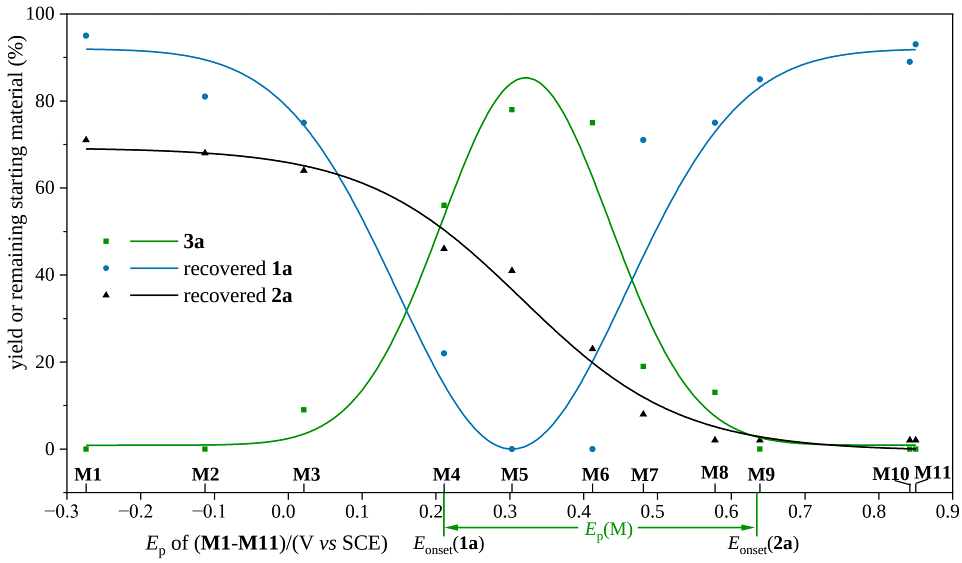

Fig. S23. A comprehensive study showed a bell-shaped curve relationship in reaction yields with mediators of varying oxidation potentials.

6.3 The radical clock experiments

To gain more insight into the possible radical reaction mechanism, cyclopropane **16** was employed as a radical clock substrate to capture the radical intermediate. When using **1a** as the substrate, we successfully obtained the ring-opening product **17** in 81% yield and 93% ee. In contrast, when using **2a** as the substrate, no desired product was observed. When **16** was added as a radical trap to the model reaction system, we successfully obtained the corresponding ring-opening product **17** as well as the alkylation product **3a**. These results suggested that the reaction proceeds via the oxidation of substrate **1a** to generate a radical intermediate, which then undergoes further reaction steps.

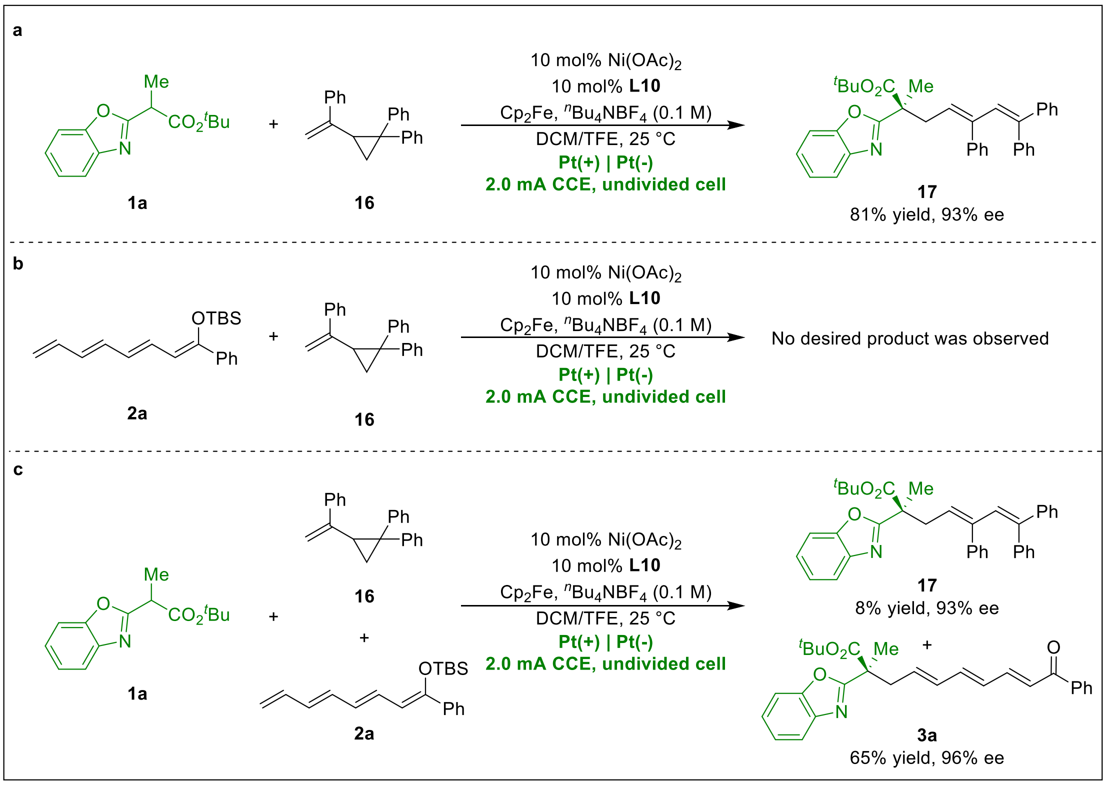

General procedure for the radical clock experiments. A 10 mL reaction tube was charged with racemic benzoxazolyl acetate **1a** (0.1 mmol, 1.0 equiv), or silyl enol ether **2a** (0.3 mmol, 3.0 equiv), or **16** (0.3 mmol, 3.0 equiv), nickel complex derived from (*R*,*R*)-**L10** (0.01 mmol, 0.1 equiv), Cp_2Fe (0.01 mmol, 0.1 equiv), $^{n}Bu_4NBF_4$ (0.1 M), DCM (2 mL), and TFE (1 mL) under an argon atmosphere. The reaction tube was equipped with a platinum plate (1.0 cm X 1.0 cm X 0.2 mm) as the anode and a platinum plate (1.0 cm X 1.0 cm X 0.2 mm) as the cathode. Constant current electrolysis was carried out at 25 ℃ for 5 h, monitored by thin-layer chromatography.

Procedure for the preparation of 17. A 10 mL reaction tube was charged with racemic benzoxazolyl acetate **1a** (0.1 mmol, 1.0 equiv), **16** (0.3 mmol, 3.0 equiv), nickel complex derived from (*R*,*R*)-**L10** (0.01 mmol, 0.1 equiv), Cp_2Fe (0.01 mmol, 0.1 equiv), $^{n}Bu_4NBF_4$ (0.1 M), DCM (2 mL), and TFE (1 mL) under an argon atmosphere. The reaction tube was equipped with a platinum plate (1.0 cm X 1.0 cm X 0.2 mm) as the anode and a platinum plate (1.0 cm X 1.0 cm X 0.2 mm) as the cathode. Constant current electrolysis was carried out at 25 ℃ for 5 h, monitored by thin-layer chromatography. The mixture was concentrated under reduced pressure, and purified by flash column chromatography on silica gel to afford the desired product **17** (43.8 mg, 71%). **^{1}H NMR (400 MHz, $CDCl_3$)** δ 7.53 (d, *J* = 7.8 Hz, 1H), 7.35 – 7.24 (m, 6H), 7.19 – 7.08 (m, 7H), 7.08 – 7.03 (m, 2H), 6.99 – 6.94 (m, 2H), 6.90 – 6.83 (m, 2H), 6.37 (d, *J* = 11.5 Hz, 1H), 3.77 – 3.66 (m, 2H), 1.62 (s, 3H), 1.32 (s, 9H). **^{13}C NMR (100 MHz, $CDCl_3$)** δ 171.7, 166.4, 150.6, 144.4, 143.3, 142.6, 140.7, 139.7, 138.0, 131.1, 130.7, 128.2, 128.2, 127.9, 127.9, 127.6, 126.7, 126.6, 124.7, 123.9, 123.9, 119.8, 110.4, 82.3, 50.1, 36.3, 27.9, 21.5.**ESI-MS:** calculated $[C_{37}H_{35}NO_3 + Na]^+$: 564.2509, found: 564.2522. The product was analyzed by HPLC to determine the enantiomeric excess: 93% ee (CHIRALPAK IE, *n*-hexane/*i*-PrOH = 90/10, detector: 330 nm, T = 25 °C, flow rate: 1 mL/min), t_1 (major) = 4.85 min, t_2 (minor) = 5.15 min.

7. X-ray crystallography data

7.1 Characterize of (*S*)-3b by X-ray crystallography

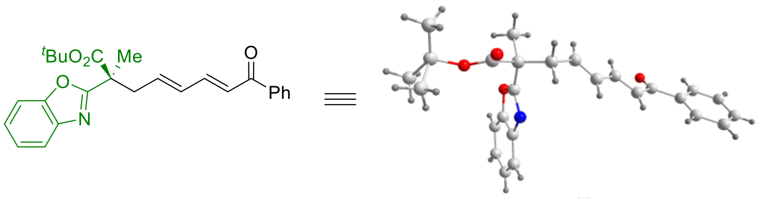

(*S*)-**3b**   (*S*)-**3b** (CCDC **2359837**)

Chemical formula	$C_{26}H_{27}NO_4$
Formula weight	417.48
Space group	P 1 2_1 1
Z	2
a, Å	10.2029(2)
b, Å	10.3745(2)
c, Å	10.8390(2)
α, °	90
β, °	90.2920(10)
γ, °	90
V, Å^3	1147.29(4)

7.2 Characterize of (*S*)-5d by X-ray crystallography

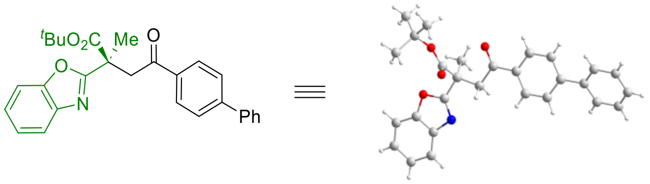

(*S*)-**5d**   (*S*)-**5d** CCDC (2266368)

Chemical formula	$C_{28}H_{27}NO_4$
Formula weight	441.50
Space group	P 1 2_1 1
Z	2
a, Å	8.8732(2)
b, Å	8.5579(2)
c, Å	17.6548(5)
α, °	90
β, °	101.762(2)
γ, °	90
V, Å^3	1312.48(6)

8. Computational Details

8.1 Computational Methods

All calculations were performed with Gaussian16 Rev. C.01 package. The ωB97X-D functional with the def2-SVP basis set were used for geometry optimizations of all structures. Frequency analysis at the same level was performed to validate each structure as a local minimum or a transition states and to gain the thermal correction to Gibbs free energy at 298.15K using Grimme's QRRHO by Shermo (version 2.5). For single-point calculations, a larger basis set def2-TZVP and SMD solvation model were used to obtain energies and wavefunctions. Since the solvent parameters for the mixed solvent (DCM/TFE=2:1) are not available under Gaussian, the full SMD parameters of the mixed solvent are defined by weighted averaging the SMD parameters of pure solvents based on the volume ratio (Eps=14.862, EpsInf=1.908, HBondAcidity=0.257, HBondBasicity=0.117, SurfaceTensionAtInterface=40.107, CarbonAromaticity = 0.0 and ElectronegativeHalogenicity = 0.611). Conformation searches were performed with ABCluster (version 3.0) using GFN2-xtb method. The Fukui function and the condensed Fukui function were conducted by Multiwfn (version 3.8). The Hirshfeld charge was applied for the condensed Fukui function. The coloring maps of the Fukui function on the molecular surface were drawn at the isosurface with an electron density of 0.001 a.u. using GaussView (version 6.0.16). By SambVca web application (version 2. A), the topographic steric maps of allenylmethylsilane (**2a**) in each transition states were drawn and the percentages of buried volume (%V_{bur}) were calculated to quantify the steric effect. The parameters for drawing the topographic steric maps and calculating %V_{bur} were set as follows: i) the radical carbon atom of the benzoxazolyl acetate was selected as the atom coordinated to the center of the sphere; ii) the atomic radii parameter was set as the bondi radii scaled by 1.17; iii) sphere radius was set at 3.5 Å; iv) distance of the coordination point from the center of the sphere was set at 0 Å; v) mesh spacing for numerical integration was set at 0.10 unit; vi) all hydrogen atoms were included in the calculations. The 3D diagrams of molecules were generated using CYLview20.

Table S9. Gibbs free energies of various transition states of radical addition of benzoxazolyl acetate (**1a**) and allenylmethylsilane (**2a**). Gibbs free energies are in kcal/mol and relative to that of TS1.

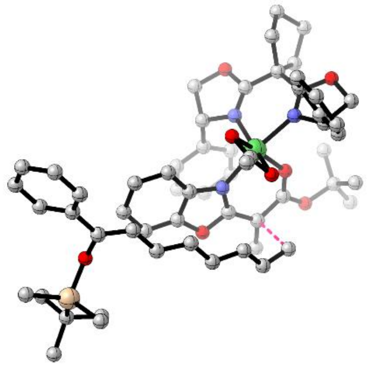<br>TS1 (η-addition)<br>(**0.0**)	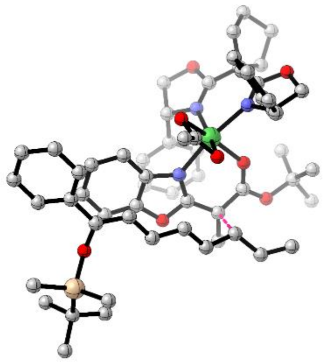<br>TS2 (ε-addition)<br>(**2.1**)
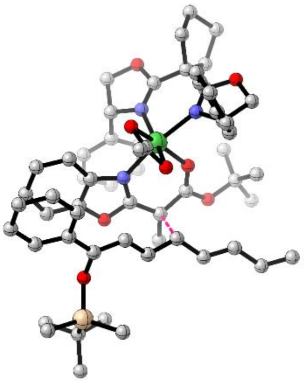<br>TS3 (γ-addition)<br>(**1.7**)	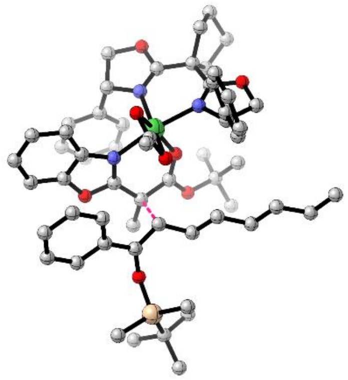<br>TS4 (α-addition)<br>(**6.0**)

The transition state relative energies indicate that η-addition is more favorable, while α-/γ-/ε-additions are relatively less favorable, in agreement with experimental results.

Table S10. The topographic steric maps and the percentages of buried volume of allenylmethylsilane (**2a**) in each transition states. Unit: Å.

<table>
<tr><td rowspan="2">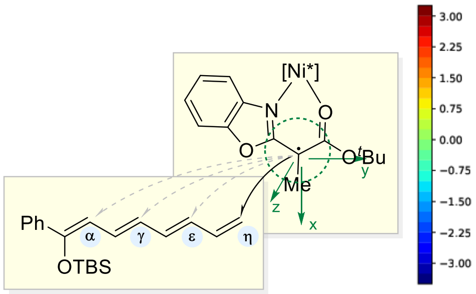
</td><td>TS1 (η-addition)
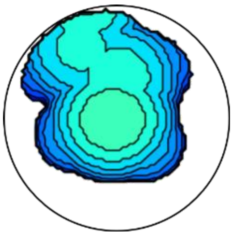
%V_{bur} = 19.9 %</td><td>TS2 (ε-addition)
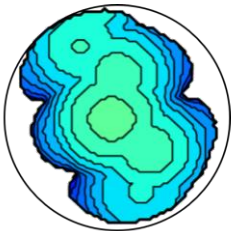
%V_{bur} = 25.7 %</td></tr>
<tr><td>TS3 (γ-addition)
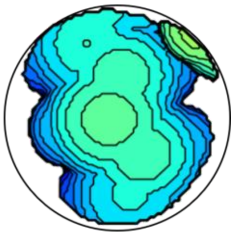
%V_{bur} = 27.0 %</td><td>TS4 (α-addition)
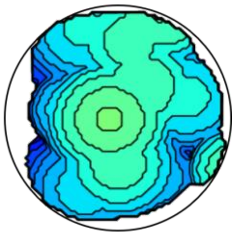
%V_{bur} = 28.9 %</td></tr>
</table>

The analysis of the topographic steric maps and the percentages of buried volume reveals that steric effects increase progressively from η-addition to α-addition, with %Vbur being most advantageous in η-addition.

8.2 Optimized Coordinates

A

C	0.91209600	1.27343100	-0.71902300
C	2.04778500	0.37213700	-0.37530500
C	1.94739700	-1.00026700	-0.63937200
C	3.21942500	0.85593200	0.22273100
C	2.99485900	-1.86331100	-0.32653700
H	1.03497000	-1.38553400	-1.09639400
C	4.26632400	-0.00700600	0.53521900
H	3.30563500	1.91680500	0.46624800
C	4.15881000	-1.37045200	0.26107500
H	2.89964700	-2.92969500	-0.54299800
H	5.16916300	0.38738100	1.00668000
H	4.97779900	-2.04801600	0.51203200
C	1.03435300	2.58535300	-0.96493000
H	2.00756500	3.07508000	-0.96246900
H	0.15282200	3.17891300	-1.21250200
O	-0.29276000	0.65400600	-0.81204000
Si	-1.37191300	0.40808100	0.47763300
C	-2.08260300	2.06881800	0.97561800
H	-2.82447200	1.96030500	1.78254700
H	-1.28099100	2.73004700	1.34027800
H	-2.57060100	2.56709000	0.12438500
C	-0.44590900	-0.34567300	1.92236000
H	-1.11461600	-0.47840100	2.78756100
H	-0.00891600	-1.32236700	1.66651000
H	0.37960900	0.31409300	2.23306800
C	-2.68192900	-0.75859700	-0.24042000
C	-3.45335300	-0.04036300	-1.35949000
H	-2.77681500	0.31183900	-2.15371100
H	-4.18633600	-0.72538800	-1.82044300
H	-4.01176000	0.82995600	-0.97979100
C	-1.99455700	-2.00575100	-0.81882600
H	-1.40331400	-2.54248100	-0.05922500
H	-2.74696100	-2.71294700	-1.20956000
H	-1.32057100	-1.74112600	-1.64790200
C	-3.65408900	-1.17459600	0.87545100
H	-4.15714900	-0.30607600	1.33122800
H	-4.44251900	-1.83377900	0.47219600
H	-3.14499100	-1.72880600	1.68040400

B

C	-0.89780300	0.97073200	0.35159500
C	-2.05286500	0.04377100	0.23726300

C	-2.01010500	-1.19750600	0.88805400
C	-3.18987300	0.36787300	-0.51609600
C	-3.08121900	-2.08234000	0.80163800
H	-1.12496600	-1.45975100	1.47011100
C	-4.26007800	-0.51886000	-0.60332100
H	-3.22694900	1.31439500	-1.05950500
C	-4.21098700	-1.74671000	0.05611200
H	-3.03415500	-3.04175400	1.32168000
H	-5.13485700	-0.25303700	-1.20089400
H	-5.04928300	-2.44295500	-0.01614400
C	-1.01061900	2.31662900	0.31126400
H	-2.00956000	2.74653500	0.20656200
O	0.30123500	0.37559800	0.56217800
Si	1.30559100	-0.30672300	-0.63272300
C	0.36317500	-1.61597900	-1.58530900
H	0.99696700	-2.04428200	-2.37820900
H	-0.52670400	-1.17817800	-2.06426100
H	0.01884700	-2.43428200	-0.93601700
C	1.83066600	1.05596000	-1.80524000
H	2.41061300	0.64728200	-2.64772500
H	2.44158300	1.82228700	-1.30595900
H	0.94140500	1.55739800	-2.21932900
C	2.74514800	-1.02157500	0.36916800
C	2.20593000	-2.06329500	1.36255900
H	1.48468300	-1.61358000	2.06243100
H	3.03153500	-2.48836100	1.95967600
H	1.70627300	-2.90171300	0.85112700
C	3.43805900	0.10949800	1.14589800
H	3.87356000	0.86443700	0.47195100
H	4.26140400	-0.29487300	1.76027500
H	2.73569200	0.62261400	1.82082600
C	3.74870100	-1.68606800	-0.58700200
H	3.29471200	-2.51810600	-1.14931600
H	4.60134700	-2.10069800	-0.02150900
H	4.15923400	-0.96984000	-1.31735200
C	0.10999700	3.23410900	0.44734800
H	1.08678400	2.77884500	0.63778700
C	0.01130100	4.56805400	0.36715700
H	-0.94874400	5.05989900	0.18247200
H	0.88585800	5.21130500	0.48546800

C

C	0.08818100	-1.08385800	0.28199700
C	1.44836400	-1.67453000	0.21737500

C	2.50744600	-1.04554900	0.88824600
C	1.71450200	-2.84777300	-0.50336900
C	3.79108700	-1.58272800	0.85213000
H	2.31080000	-0.12842100	1.44588700
C	2.99876400	-3.38400700	-0.53847400
H	0.91231900	-3.33453300	-1.06189000
C	4.04243100	-2.75487000	0.13999700
H	4.60243000	-1.08279800	1.38604400
H	3.18813700	-4.29614800	-1.10860700
H	5.04980500	-3.17536700	0.10834400
C	-1.05116000	-1.80804700	0.18602000
H	-0.96477600	-2.89048100	0.06843300
O	0.04568900	0.25006100	0.51036700
Si	0.22464700	1.48374800	-0.65061600
C	1.83258300	1.25035200	-1.58404600
H	1.93315400	2.01588200	-2.36991400
H	1.85672500	0.26238300	-2.06997900
H	2.70954600	1.31404400	-0.92325600
C	-1.21011400	1.37496900	-1.85002100
H	-1.10965000	2.12426900	-2.65118500
H	-2.17972300	1.53034100	-1.35466300
H	-1.23248900	0.37905300	-2.32021200
C	0.20674500	3.06458900	0.39380400
C	1.30587800	2.97430900	1.46442900
H	1.13170800	2.12731000	2.14545400
H	1.32715500	3.89589800	2.07199000
H	2.30656600	2.85234600	1.01901700
C	-1.16141000	3.21202800	1.07857500
H	-1.97566100	3.32946700	0.34583500
H	-1.17257300	4.10494000	1.72781300
H	-1.39386600	2.33821400	1.70734300
C	0.46553900	4.27600100	-0.51651000
H	1.45219800	4.22183300	-1.00395300
H	0.44105600	5.21078700	0.07026100
H	-0.29675200	4.36854100	-1.30752100
C	-2.38062800	-1.24314400	0.27953300
H	-2.44464300	-0.16836200	0.47793000
C	-3.52279100	-1.94988100	0.15249700
H	-3.46821600	-3.02807300	-0.04159200
C	-4.85046700	-1.36367600	0.26002600
H	-4.88898500	-0.28524100	0.45582000
C	-5.99766302	-2.04502200	0.13717900
H	-6.00097901	-3.12198700	-0.05707800
H	-6.96757098	-1.55184000	0.22904300

D

C	0.77055900	-1.03828300	0.32352800
C	2.05584100	-1.77458600	0.24326900
C	3.18674200	-1.26507400	0.89795300
C	2.18260000	-2.96868200	-0.48184700
C	4.40494400	-1.93595600	0.83977900
H	3.09575000	-0.33408200	1.45970700
C	3.40132500	-3.63892000	-0.53872900
H	1.32425300	-3.36573100	-1.02749300
C	4.51747700	-3.12619100	0.12216700
H	5.27382400	-1.52625900	1.35953900
H	3.48279400	-4.56453200	-1.11277400
H	5.47447599	-3.64985600	0.07191400
C	-0.44464300	-1.62714600	0.22284900
H	-0.48591500	-2.71144900	0.09864100
O	0.87704800	0.29110500	0.55466300
Si	1.12213200	1.51283500	-0.60728500
C	2.88404600	1.43530000	-1.24001700
H	3.06648000	2.23411800	-1.97658400
H	3.07054600	0.46977900	-1.73565100
H	3.61968600	1.53989500	-0.42897600
C	-0.06327500	1.22477900	-2.02862400
H	0.13173200	1.93877600	-2.84426900
H	-1.11585600	1.32626400	-1.72628000
H	0.07000600	0.20839800	-2.43188000
C	0.75709300	3.10643400	0.35032600
C	1.80071900	3.28695900	1.46443500
H	1.80699500	2.42886900	2.15484300
H	1.57652300	4.19091200	2.05737000
H	2.81808300	3.40576000	1.05875100
C	-0.64412700	3.01422400	0.97579900
H	-1.42725200	2.87086500	0.21334600
H	-0.88358900	3.94482900	1.51921400
H	-0.70966500	2.18049200	1.69131200
C	0.81592500	4.30169000	-0.61460300
H	1.80293000	4.39869700	-1.09569400
H	0.62505100	5.24367300	-0.07161400
H	0.05843400	4.22535600	-1.41122000
C	-1.69774300	-0.90933500	0.29725200
H	-1.63823700	0.16575900	0.49474400
C	-2.91556800	-1.47361600	0.14206800
H	-2.98758400	-2.55072900	-0.05116000
C	-4.15803900	-0.73168400	0.20614200

H	-4.07704900	0.34564700	0.39878600
C	-5.38343899	-1.27147600	0.04079200
H	-5.47040799	-2.34792200	-0.15087000
C	-6.62241302	-0.51018400	0.09800700
H	-6.52297401	0.56529300	0.28736400
C	-7.84441899	-1.03505800	-0.06511500
H	-7.98369002	-2.10362100	-0.25526800
H	-8.74320802	-0.41687800	-0.01368200

TS1

C	-7.17783100	1.12745000	2.96058800
C	-6.30035500	2.13028200	3.70530900
C	-5.18896800	2.43660100	2.70150000
C	-6.16743400	0.18485600	2.30151100
H	-7.87967200	0.59047600	3.61356300
H	-7.76641100	1.64327300	2.18622900
H	-5.87377700	1.66641800	4.60755300
H	-6.83862000	3.03693300	4.01495000
H	-5.53728600	3.18407900	1.97465500
H	-4.26969200	2.82735600	3.15850600
H	-6.56555100	-0.32085500	1.41278500
H	-5.85434200	-0.59037800	3.01380500
C	-4.92693300	1.08100800	1.94229600
C	-4.88789000	1.36866100	0.47138500
C	-4.33430300	1.89850900	-1.61373900
C	-5.87177600	1.89837100	-1.45363500
H	-4.00862100	1.21937700	-2.41304100
H	-6.34944800	2.83147100	-1.77427300
H	-6.35170700	1.04271200	-1.95174200
C	-3.67508700	0.44577800	2.47492200
C	-2.41593500	-0.25443200	4.18047400
C	-1.74370600	-0.60112200	2.83205500
H	-1.88477300	0.53345700	4.73304700
H	-2.56896300	-1.12373100	4.83137400
H	-0.74426100	-0.14925500	2.76596100
N	-3.89077700	1.36230600	-0.32342400
N	-2.62771600	0.05084300	1.85785400
O	-6.06019800	1.74885700	-0.03926100
O	-3.69671100	0.25857800	3.79424200
C	-3.76623400	3.27707800	-1.88809500
C	-3.54338500	3.67310800	-3.20975100
C	-3.52595800	4.18682500	-0.85595700
C	-3.11082000	4.96518200	-3.49852000
H	-3.70119700	2.95885400	-4.02188700

C	-3.09008500	5.47997100	-1.14280600
H	-3.65581400	3.88092300	0.18398800
C	-2.88827300	5.87490800	-2.46448600
H	-2.94044300	5.26109400	-4.53560200
H	-2.90252200	6.18185100	-0.32778900
H	-2.54877900	6.88814700	-2.68838700
C	-1.62501000	-2.08923300	2.57438500
C	-2.71476900	-2.82909400	2.10606200
C	-0.42059400	-2.74444900	2.84098600
C	-2.59955100	-4.20205900	1.90281000
H	-3.65534800	-2.32641900	1.87124700
C	-0.30318000	-4.11795400	2.63489000
H	0.44276000	-2.17235100	3.19209900
C	-1.39320000	-4.85031700	2.16778700
H	-3.45386100	-4.76942100	1.52730600
H	0.64743200	-4.61616600	2.83480800
H	-1.30115000	-5.92635500	2.00682700
Ni	-2.07876700	0.36029500	-0.12147800
O	-1.39143200	1.26167500	-1.90148200
O	-0.94319600	2.10578700	0.06063500
C	-0.83388500	2.15065100	-1.19514400
C	-0.06874600	3.26307700	-1.85325200
H	0.37173400	2.92664200	-2.80054700
H	-0.77500400	4.07578300	-2.08132500
H	0.70133600	3.65631900	-1.17804000
C	2.84194600	-2.06728500	0.80935500
C	1.55672200	-1.88527000	0.32330400
C	0.82256800	-0.71116600	0.48954700
C	1.38080300	0.37507400	1.17229100
C	2.66505000	0.20693500	1.68162200
C	3.38480100	-0.98803300	1.50355800
H	3.38457000	-3.00120900	0.66133400
H	0.82428300	1.30786500	1.27379100
H	3.14059500	1.03246000	2.21457200
H	4.39916200	-1.06394300	1.89914200
N	-0.42427500	-0.91495700	-0.08156300
O	0.77599500	-2.76904300	-0.34838200
C	-0.41006800	-2.15079000	-0.54159400
C	-1.41374900	-2.89958700	-1.22675300
C	-1.13431000	-4.34521400	-1.52372400
H	-1.18392500	-4.94923500	-0.60324000
H	-1.87015600	-4.74435900	-2.23052600
H	-0.12849400	-4.46908500	-1.94589200
C	-2.77672600	-2.40221800	-1.19696300

O	-3.11647400	-1.26669700	-0.85241100
O	-3.66298600	-3.28814800	-1.61605600
C	-5.08483700	-2.98756300	-1.75362700
C	-5.66299700	-4.30256500	-2.26299400
H	-5.48348300	-5.10960600	-1.53865600
H	-6.74674200	-4.20517500	-2.41644800
H	-5.19907900	-4.58350500	-3.21889000
C	-5.68123500	-2.62598600	-0.39436800
H	-5.47707200	-3.41989500	0.33926500
H	-5.26986100	-1.68120000	-0.01859300
H	-6.77218800	-2.52615100	-0.49028100
C	-5.27964900	-1.87608300	-2.78325700
H	-4.86276300	-0.92752300	-2.42396700
H	-4.79148400	-2.14114200	-3.73242000
H	-6.35367000	-1.74320000	-2.97873300
C	5.72864700	1.34551600	0.07594800
C	6.26351400	2.40112000	0.96192600
C	6.96809300	2.03570400	2.11937600
C	6.07609300	3.76436700	0.68573800
C	7.45992700	3.00720500	2.98548700
H	7.11851400	0.97713200	2.33727300
C	6.57267100	4.73377800	1.55155900
H	5.56115200	4.06930900	-0.22758600
C	7.26323500	4.35905900	2.70474100
H	7.99947500	2.70826000	3.88647800
H	6.42819100	5.79111900	1.32085800
H	7.65242000	5.12176800	3.38223900
C	4.60262200	1.49965300	-0.68501000
H	4.05148300	2.43912200	-0.61057400
O	6.34774400	0.15902400	0.12757900
Si	7.77040100	-0.36857400	-0.67499100
C	9.25821200	0.47784700	0.07474700
H	10.17868800	0.16568200	-0.44353800
H	9.16951900	1.57069400	-0.02650200
H	9.37388100	0.24879600	1.14419100
C	7.59558300	0.10478700	-2.47593300
H	8.50652200	-0.15775100	-3.03619900
H	6.74186500	-0.39233200	-2.95961600
H	7.44857400	1.19231800	-2.57139600
C	7.73041400	-2.23677700	-0.37296400
C	7.81729900	-2.51280500	1.13691700
H	6.98636600	-2.03865500	1.68328600
H	7.76918500	-3.59783100	1.33202800
H	8.76052000	-2.14496700	1.57041800

C	6.41348600	-2.81258400	-0.91834200
H	6.30947800	-2.65287600	-2.00380200
H	6.36789800	-3.90139000	-0.74348000
H	5.54271900	-2.35433300	-0.42381100
C	8.92231600	-2.89090900	-1.09138300
H	9.88740800	-2.50746400	-0.72294200
H	8.92023400	-3.98137700	-0.92332700
H	8.88769600	-2.72837300	-2.18064800
C	4.05085500	0.44962700	-1.47314300
H	4.63214100	-0.47303900	-1.55911400
C	2.80463700	0.48501400	-2.04440200
H	2.18717500	1.38083200	-1.91800700
C	2.22832300	-0.62603700	-2.70833100
H	2.85973200	-1.50818200	-2.87108800
C	0.89418800	-0.68697600	-3.04470500
H	0.25191100	0.17478200	-2.83232500
C	0.25198700	-1.85165100	-3.52568300
H	0.86567200	-2.71406800	-3.80972900
C	-1.11459400	-1.97654500	-3.48965900
H	-1.72194600	-1.09535900	-3.26310700
H	-1.61635600	-2.85277400	-3.90335900

TS2

C	-7.34789500	0.64458100	2.03554500
C	-6.76355000	1.75969500	2.89858800
C	-5.52348300	2.19462300	2.11714000
C	-6.12049200	-0.16719700	1.61397300
H	-8.09122400	0.02909500	2.56093200
H	-7.83443000	1.07225700	1.14543200
H	-6.46885500	1.36426500	3.88239400
H	-7.46092400	2.59239400	3.06549700
H	-5.81244700	2.88488000	1.31214000
H	-4.76710600	2.70344300	2.72982000
H	-6.27289200	-0.72403000	0.68090700
H	-5.85884800	-0.89400500	2.39517000
C	-4.95297300	0.87568600	1.47106100
C	-4.66239400	1.14933200	0.02564700
C	-3.77439000	1.70092000	-1.93591600
C	-5.31067600	1.56784000	-2.06474400
H	-3.24871400	1.03103600	-2.63018500
H	-5.79483800	2.46131800	-2.47631300
H	-5.61868700	0.68127200	-2.63823400
C	-3.76182600	0.41047700	2.25998500
C	-2.78464000	-0.08007300	4.20862800

C	-1.81921100	-0.36119300	3.03247200
H	-2.46816800	0.76767600	4.83207000
H	-2.96609300	-0.95642900	4.84295100
H	-0.90387500	0.23960900	3.12602300
N	-3.53620100	1.24139300	-0.56492200
N	-2.58035400	0.11692400	1.87006300
O	-5.74796700	1.39300600	-0.70970100
O	-4.01237300	0.27546800	3.56160800
C	-3.28243700	3.11713800	-2.16088800
C	-2.86797900	3.50260400	-3.43872000
C	-3.30070000	4.06605700	-1.13630200
C	-2.50081500	4.82137000	-3.69621800
H	-2.82426800	2.76049100	-4.23990800
C	-2.92968400	5.38582800	-1.39118400
H	-3.58047400	3.77075800	-0.12302200
C	-2.53646600	5.76871700	-2.67262100
H	-2.18062000	5.10979500	-4.69952800
H	-2.94427600	6.11852300	-0.58197300
H	-2.25063400	6.80333400	-2.87239700
C	-1.43008800	-1.81902600	2.90235000
C	-2.28523200	-2.73645300	2.28714500
C	-0.22012700	-2.26791000	3.43665900
C	-1.93236600	-4.08034600	2.19679600
H	-3.22568000	-2.39244200	1.85434000
C	0.13499700	-3.61298100	3.34625100
H	0.46415600	-1.55624200	3.90685800
C	-0.71952800	-4.52225700	2.72569600
H	-2.60364300	-4.78841400	1.70581700
H	1.08848400	-3.94959200	3.75798700
H	-0.44107300	-5.57546200	2.65239900
Ni	-1.70780200	0.44156300	0.00903800
O	-0.77090900	1.36481500	-1.64226700
O	-0.79852200	2.29795100	0.33129000
C	-0.44345900	2.31853900	-0.87843300
C	0.35046200	3.47373000	-1.41862300
H	1.05821000	3.13341800	-2.18613300
H	-0.34854500	4.17574400	-1.89850100
H	0.86970600	3.99963500	-0.60787300
C	3.17124900	-1.42894700	1.90827500
C	1.98325200	-1.39255000	1.19765300
C	1.11186000	-0.30503400	1.19727800
C	1.42060200	0.84385300	1.93005100
C	2.60662900	0.82578000	2.65975300
C	3.46393900	-0.28768900	2.65255500

H	3.83281400	-2.29496800	1.88309900
H	0.76428000	1.71469300	1.89688300
H	2.89484400	1.70963600	3.23240100
H	4.39037000	-0.25020600	3.22824900
N	0.02440400	-0.64444100	0.40183000
O	1.43729700	-2.35729700	0.41082800
C	0.26063700	-1.86411300	-0.02614300
C	-0.48698500	-2.69841600	-0.93096000
C	-0.03902600	-4.13210900	-1.04712900
H	-0.24210200	-4.68340400	-0.11525700
H	-0.57065000	-4.63478200	-1.86254500
H	1.04141600	-4.18848100	-1.23503500
C	-1.90531500	-2.39351300	-1.07989700
O	-2.42813100	-1.30034900	-0.84324100
O	-2.61164900	-3.40753300	-1.54092000
C	-4.06003400	-3.37113300	-1.73004300
C	-4.36237400	-4.76580300	-2.26413500
H	-4.04761300	-5.53205300	-1.54185100
H	-5.44026800	-4.87856000	-2.44632900
H	-3.82745200	-4.93858200	-3.20867500
C	-4.74716100	-3.14186900	-0.38627400
H	-4.42393100	-3.89407300	0.34736800
H	-4.52626600	-2.14048900	0.00393800
H	-5.83505100	-3.23929900	-0.51215100
C	-4.45668100	-2.30549100	-2.74911700
H	-4.19087000	-1.30172600	-2.39641500
H	-3.96704700	-2.49100500	-3.71411300
H	-5.54382400	-2.35245900	-2.90887900
C	4.69117500	1.23180800	-0.07337800
C	4.95612200	2.39845500	0.78752200
C	5.87810200	2.27727300	1.83921500
C	4.28799400	3.62242000	0.61687800
C	6.11709000	3.34178800	2.70220600
H	6.39223500	1.32658300	1.98336600
C	4.53034500	4.68610100	1.47947700
H	3.58474600	3.75414900	-0.20735700
C	5.44332300	4.55016700	2.52667900
H	6.83484900	3.22872500	3.51728200
H	4.01045200	5.63422400	1.32816800
H	5.63318400	5.38752100	3.20115400
C	3.53740600	1.08082100	-0.81138100
H	2.77807100	1.86176700	-0.75050200
O	5.58334800	0.24035200	-0.02531800
Si	7.01554600	-0.05130300	-0.92151400

C	8.36020500	1.09139200	-0.30518300
H	9.28787600	0.93965800	-0.87928200
H	8.05902300	2.14360300	-0.42612700
H	8.58934800	0.92670000	0.75788500
C	6.64209400	0.30042000	-2.71975800
H	7.55945500	0.22228300	-3.32391300
H	5.89741100	-0.39145700	-3.13968500
H	6.25307900	1.32439100	-2.83681800
C	7.35470600	-1.87677500	-0.55431900
C	7.59095000	-2.06014900	0.95387500
H	6.72336800	-1.71923500	1.54160000
H	7.75804900	-3.12542100	1.18826100
H	8.47695900	-1.50778000	1.30426900
C	6.14406900	-2.72368700	-0.98008100
H	5.92641700	-2.62315300	-2.05589600
H	6.33709700	-3.79264000	-0.78593600
H	5.23890700	-2.44019200	-0.42007200
C	8.60057300	-2.32172500	-1.33845800
H	9.49640400	-1.74626700	-1.05476800
H	8.82002700	-3.38444000	-1.13904500
H	8.46197900	-2.21616900	-2.42650200
C	3.22362100	-0.08850200	-1.53426600
H	3.97197200	-0.88397100	-1.58405100
C	1.96487400	-0.31770700	-2.07471700
H	1.19971000	0.45990100	-1.98417700
C	1.56437700	-1.52511600	-2.64649000
H	2.30542500	-2.31703800	-2.80690900
C	0.21129300	-1.79591400	-2.91661800
H	-0.47593700	-0.94620500	-2.82785200
C	-0.16169500	-2.86634000	-3.83673600
H	0.54545700	-3.69637800	-3.93789100
C	-1.27440100	-2.85023300	-4.57925300
H	-1.97522200	-2.01288600	-4.52281500
H	-1.50329900	-3.64976800	-5.28684800

TS3

C	-7.23581000	0.52960800	-1.58620700
C	-7.09674200	-0.85240300	-2.21891800
C	-5.92374200	-1.46928000	-1.45773200
C	-5.78946800	1.01435700	-1.45387900
H	-7.85249400	1.22019800	-2.17792700
H	-7.69262700	0.44266700	-0.58820800
H	-6.84630500	-0.75834900	-3.28633900
H	-8.00771400	-1.46193100	-2.13952500

H	-6.27388900	-1.86649600	-0.49416100
H	-5.42592700	-2.29141800	-1.98976000
H	-5.65650700	1.75856900	-0.65868700
H	-5.45537300	1.47171500	-2.39514500
C	-4.93378800	-0.27555400	-1.17928400
C	-4.51444300	-0.34370100	0.25887300
C	-3.53714200	-0.76924700	2.21007700
C	-4.93120300	-0.13566900	2.43925400
H	-2.73863200	-0.17474800	2.67553400
H	-5.58880900	-0.74755100	3.06841700
H	-4.88038000	0.88614100	2.84194100
C	-3.79377800	-0.33772400	-2.15509600
C	-3.02267300	-0.50950900	-4.24449500
C	-1.85436500	-0.31970900	-3.24868200
H	-3.04734100	-1.51229500	-4.69319800
H	-3.04993200	0.24960800	-5.03562400
H	-1.15313500	-1.16348000	-3.30670100
N	-3.39218000	-0.69234700	0.75344100
N	-2.53276800	-0.35402100	-1.94598600
O	-5.49345100	-0.06827500	1.12179500
O	-4.18940200	-0.35769800	-3.42700300
C	-3.44924800	-2.19116600	2.72597000
C	-2.87681300	-2.43576400	3.97648600
C	-4.00406400	-3.25555000	2.01089300
C	-2.88248800	-3.71895800	4.51879000
H	-2.41377900	-1.61392900	4.52831300
C	-4.00903500	-4.54098900	2.55076300
H	-4.42194300	-3.08735400	1.01588400
C	-3.45574900	-4.77418000	3.80947100
H	-2.43234000	-3.89673400	5.49749500
H	-4.44503300	-5.36580100	1.98365000
H	-3.46278500	-5.77990000	4.23426300
C	-1.08331600	0.96736400	-3.45833200
C	-1.54770000	2.17754000	-2.93711200
C	0.09068100	0.95795000	-4.21533900
C	-0.84444600	3.35863900	-3.15958700
H	-2.45447400	2.19277600	-2.33054200
C	0.79524700	2.13971300	-4.43930900
H	0.47443400	0.01435900	-4.61343300
C	0.33038200	3.34261000	-3.91136100
H	-1.21099400	4.29802600	-2.73969500
H	1.71844800	2.11687700	-5.02175500
H	0.88274800	4.26832600	-4.08467800
Ni	-1.51451200	-0.60380400	-0.14643400

O	-0.61123700	-1.45494300	1.56636400
O	-1.24395000	-2.67177900	-0.13313700
C	-0.74524500	-2.58945700	1.02268800
C	-0.35471900	-3.83833800	1.75994700
H	0.30334200	-3.60944100	2.60686700
H	-1.27158900	-4.30445200	2.14991000
H	0.11606400	-4.55341900	1.07305400
C	3.27264500	-0.55802000	-2.96249000
C	2.26670900	-0.14090500	-2.10781200
C	1.17250900	-0.92801100	-1.75435100
C	1.05509200	-2.23212100	-2.23897600
C	2.05645700	-2.67314100	-3.10036100
C	3.13899700	-1.85246800	-3.46183800
H	4.11123400	0.08821200	-3.22161500
H	0.22344000	-2.86584800	-1.92652800
H	2.00810200	-3.68862300	-3.49748500
H	3.90112100	-2.24382400	-4.13811000
N	0.35767300	-0.16136800	-0.93039800
O	2.12255000	1.06288700	-1.49189800
C	0.96201900	0.99647000	-0.81109500
C	0.63004600	2.13777800	0.00619700
C	1.47315200	3.36993800	-0.19028900
H	1.29885400	3.81203800	-1.18403200
H	1.22873800	4.12561600	0.56397900
H	2.54153200	3.12569100	-0.12061400
C	-0.77619900	2.30179600	0.36239600
O	-1.60979900	1.39234800	0.39599900
O	-1.08902800	3.53820600	0.69306700
C	-2.44254600	3.97587100	1.03388900
C	-2.25436100	5.46340900	1.30340500
H	-1.86332100	5.97211500	0.41094700
H	-3.21414700	5.92369700	1.57665600
H	-1.54622200	5.61259800	2.13105200
C	-3.37026600	3.74516800	-0.15621700
H	-2.96483400	4.21917500	-1.06146100
H	-3.51218600	2.67332200	-0.34390100
H	-4.34976500	4.19904200	0.05181600
C	-2.94999700	3.26526100	2.28552300
H	-2.99788600	2.18089400	2.13115600
H	-2.30260800	3.48709100	3.14320700
H	-3.95844200	3.63668600	2.51992500
C	3.81579200	-1.12080100	0.58284300
C	3.90624200	-2.51644700	0.13176500
C	4.89258900	-2.88513100	-0.79733300

C	3.00854200	-3.49939200	0.58311300
C	4.97436800	-4.19191600	-1.26574400
H	5.58181900	-2.12632700	-1.16742100
C	3.09043700	-4.80336200	0.11176300
H	2.24999500	-3.24671200	1.32314200
C	4.07195500	-5.15533900	-0.81658000
H	5.74428500	-4.46030200	-1.99188200
H	2.38907200	-5.55586500	0.47843800
H	4.13735900	-6.18161800	-1.18373600
C	2.68832500	-0.58894300	1.19439400
H	1.80773000	-1.22035500	1.32251500
O	4.83824400	-0.32413200	0.28027400
Si	6.29340300	0.11739600	1.07243000
C	7.52428200	-1.27756600	0.89737400
H	8.46489900	-1.02503300	1.41189600
H	7.13045400	-2.19967600	1.35263500
H	7.76146800	-1.49407800	-0.15446600
C	5.91024500	0.40317200	2.88087700
H	6.84636500	0.54497300	3.44341100
H	5.27506000	1.28396300	3.05354900
H	5.39686900	-0.47293400	3.30798000
C	6.78791800	1.68649000	0.13715200
C	7.11151700	1.33053000	-1.32343400
H	6.25498800	0.84546900	-1.81855000
H	7.35614800	2.24224300	-1.89475500
H	7.97727400	0.65446200	-1.40195100
C	5.61884400	2.68498200	0.16363400
H	5.34312900	2.97083800	1.19253800
H	5.89337900	3.61252300	-0.36711800
H	4.72696800	2.26593500	-0.32824300
C	8.02337500	2.30847300	0.80888100
H	8.88406900	1.62029900	0.80870500
H	8.33528800	3.21935200	0.27031900
H	7.82392500	2.59828300	1.85306600
C	2.57763300	0.74814700	1.59066300
H	3.45416500	1.39723200	1.52235300
C	1.35019100	1.32209900	1.97740300
H	0.50869300	0.62767500	2.09158500
C	1.32848000	2.53294800	2.78249100
H	2.18571800	3.20996000	2.69436400
C	0.33280900	2.83517900	3.63771600
H	-0.49916900	2.12882800	3.74374600
C	0.29325200	4.03784200	4.45625400
H	1.11294600	4.75216500	4.31962800

C	-0.67392000	4.30844100	5.34343900
H	-1.50010500	3.61143300	5.51538500
H	-0.66602000	5.22554400	5.93580100

TS4

C	-6.24187400	0.80838200	-2.67391000
C	-7.06006500	-0.00686800	-1.67565200
C	-6.20316500	0.01992900	-0.40860300
C	-4.81128500	0.31583000	-2.44884700
H	-6.56541100	0.67592800	-3.71574500
H	-6.31039200	1.88049700	-2.43390500
H	-7.17162800	-1.04219900	-2.03217200
H	-8.06630900	0.39949200	-1.50323700
H	-6.37872100	0.95510900	0.14020800
H	-6.40868700	-0.81086200	0.27914400
H	-4.04446000	1.03775700	-2.75908000
H	-4.63944200	-0.61218900	-3.01259500
C	-4.71286400	0.00098700	-0.90876200
C	-3.94654400	1.09038000	-0.21860700
C	-2.60404700	2.34469200	1.03733900
C	-3.56404500	3.24706200	0.22164300
H	-1.55671100	2.54816400	0.77739800
H	-4.18850700	3.89370700	0.85089600
H	-3.04730300	3.85475600	-0.53456400
C	-4.12415500	-1.37076600	-0.74186700
C	-4.25370800	-3.58960200	-0.93496200
C	-2.82349200	-3.16476500	-0.53247400
H	-4.79558000	-4.08951500	-0.11939700
H	-4.29087500	-4.20845700	-1.83945700
H	-2.51579600	-3.65699700	0.40012900
N	-2.94060200	1.00084900	0.55935200
N	-2.98077400	-1.72348400	-0.29233000
O	-4.41831800	2.31447800	-0.45577800
O	-4.90742700	-2.34322400	-1.20668900
C	-2.77722400	2.48817200	2.53450100
C	-1.94385000	3.34999700	3.25211300
C	-3.80311700	1.81953900	3.20627400
C	-2.14866600	3.56410300	4.61356500
H	-1.11466200	3.85075000	2.74550100
C	-4.00604100	2.02664100	4.56970500
H	-4.43835600	1.11488000	2.66536200
C	-3.18529600	2.90583700	5.27519900
H	-1.49079500	4.24059000	5.16265600
H	-4.80958600	1.49593700	5.08433200

H	-3.34693500	3.07076700	6.34221800
C	-1.78298200	-3.43959900	-1.60104500
C	-1.62191200	-2.57014100	-2.68251900
C	-0.99146000	-4.58829400	-1.53023600
C	-0.67544000	-2.83339100	-3.66899600
H	-2.22006900	-1.65923300	-2.73624100
C	-0.04399800	-4.85478300	-2.51775400
H	-1.09441100	-5.26903800	-0.68052700
C	0.11964600	-3.97640800	-3.58719600
H	-0.55584300	-2.14029900	-4.50393000
H	0.57668000	-5.74984800	-2.44314300
H	0.86577600	-4.18296100	-4.35697100
Ni	-1.55343800	-0.54563500	0.63405100
O	-0.40754500	0.52198800	2.06922400
O	-1.86089400	-1.00023500	2.64196900
C	-0.99027700	-0.15148600	2.97096500
C	-0.66626600	0.07901400	4.41783700
H	0.32796900	0.52730100	4.53395600
H	-1.41035300	0.78158800	4.82328900
H	-0.74353700	-0.85890900	4.98198100
C	1.87902200	-4.90979800	0.89608100
C	1.36108000	-3.68590700	0.50890300
C	0.23633300	-3.10034300	1.08679500
C	-0.42927100	-3.73545800	2.13668700
C	0.07716900	-4.96673000	2.54516300
C	1.20492000	-5.54629700	1.93623800
H	2.76264000	-5.33628100	0.42198400
H	-1.28372700	-3.25865300	2.61947700
H	-0.40873900	-5.49532700	3.36770200
H	1.56677200	-6.51208700	2.29398400
N	0.00976000	-1.89918100	0.42870600
O	1.80596300	-2.84780800	-0.46459400
C	0.96872000	-1.79525900	-0.45891900
C	1.31647200	-0.67530700	-1.31289400
C	2.42147000	-0.91333200	-2.31139900
H	2.05964000	-1.52619000	-3.15260800
H	2.78647900	0.03848000	-2.71437100
H	3.26252100	-1.43957900	-1.84540800
C	0.19821900	0.17572700	-1.73908800
O	-0.88783000	0.25790200	-1.16447100
O	0.48490400	0.88961500	-2.80515100
C	-0.45050700	1.74916700	-3.53114300
C	0.47718900	2.57861100	-4.41100000
H	1.11044000	1.92762300	-5.02977800

H	-0.11029700	3.22931600	-5.07353000
H	1.12760600	3.20973200	-3.78825900
C	-1.34971400	0.84483700	-4.36650700
H	-0.75074600	0.20862300	-5.03342400
H	-1.96641300	0.20735800	-3.71873700
H	-2.02229500	1.45549200	-4.98577500
C	-1.26921800	2.66097200	-2.62036200
H	-2.02075700	2.10116700	-2.05475200
H	-0.62181800	3.19610400	-1.91484500
H	-1.77754000	3.40793800	-3.24756400
C	3.40846900	-0.02719300	0.47216600
C	3.52772700	-1.10993900	1.44868900
C	4.58262900	-2.03735500	1.35069800
C	2.59937100	-1.25407100	2.49840000
C	4.69951500	-3.07429800	2.26850700
H	5.29932000	-1.94267800	0.53393500
C	2.72317500	-2.29283300	3.41353700
H	1.77808700	-0.54354300	2.60189100
C	3.77112200	-3.20675700	3.30356500
H	5.51826000	-3.79076600	2.17493500
H	1.99341900	-2.38898700	4.21977000
H	3.86381200	-4.02236400	4.02323200
C	2.17620200	0.60454200	0.19185900
H	1.37344600	0.42047400	0.90903500
O	4.47998800	0.29349900	-0.25274400
Si	5.81729000	1.31242300	0.12437900
C	7.00372400	0.37893900	1.22580500
H	7.86335000	1.01886600	1.48122700
H	6.51253300	0.08646300	2.16659400
H	7.39135200	-0.53215900	0.74691500
C	5.14988900	2.80591400	1.03082300
H	5.98138700	3.42777000	1.39796000
H	4.50458800	3.43373500	0.40000500
H	4.55910600	2.49093500	1.90592400
C	6.53920100	1.71147700	-1.57734200
C	7.07982800	0.42415800	-2.22149000
H	6.29315600	-0.34033100	-2.32438500
H	7.47196000	0.63603000	-3.23078800
H	7.90518400	-0.01347200	-1.63853300
C	5.45069700	2.31180800	-2.48174200
H	5.01919100	3.23262200	-2.05606900
H	5.87409600	2.57481600	-3.46585200
H	4.63206400	1.59547300	-2.65261700
C	7.68129000	2.72707900	-1.40083600

H	8.48047600	2.34343900	-0.74641500
H	8.14307000	2.95662100	-2.37597900
H	7.32446600	3.67881100	-0.97541700
C	2.14447600	1.94297500	-0.39297200
H	2.87165900	2.19576600	-1.16978900
C	1.26333500	2.86163600	0.05037200
H	0.57227200	2.56282200	0.84850200
C	1.17650600	4.22467400	-0.43493600
H	1.88783400	4.53089400	-1.21160700
C	0.25817100	5.11043200	0.00319200
H	-0.44791600	4.79444900	0.78136400
C	0.11282000	6.47074400	-0.49133000
H	0.82157400	6.79306500	-1.26223600
C	-0.82647300	7.32253000	-0.05935500
H	-1.54453500	7.03325600	0.71438900
H	-0.90680200	8.33555600	-0.45840700

9. NMR spectra

^{1}H NMR spectrum of **3a**

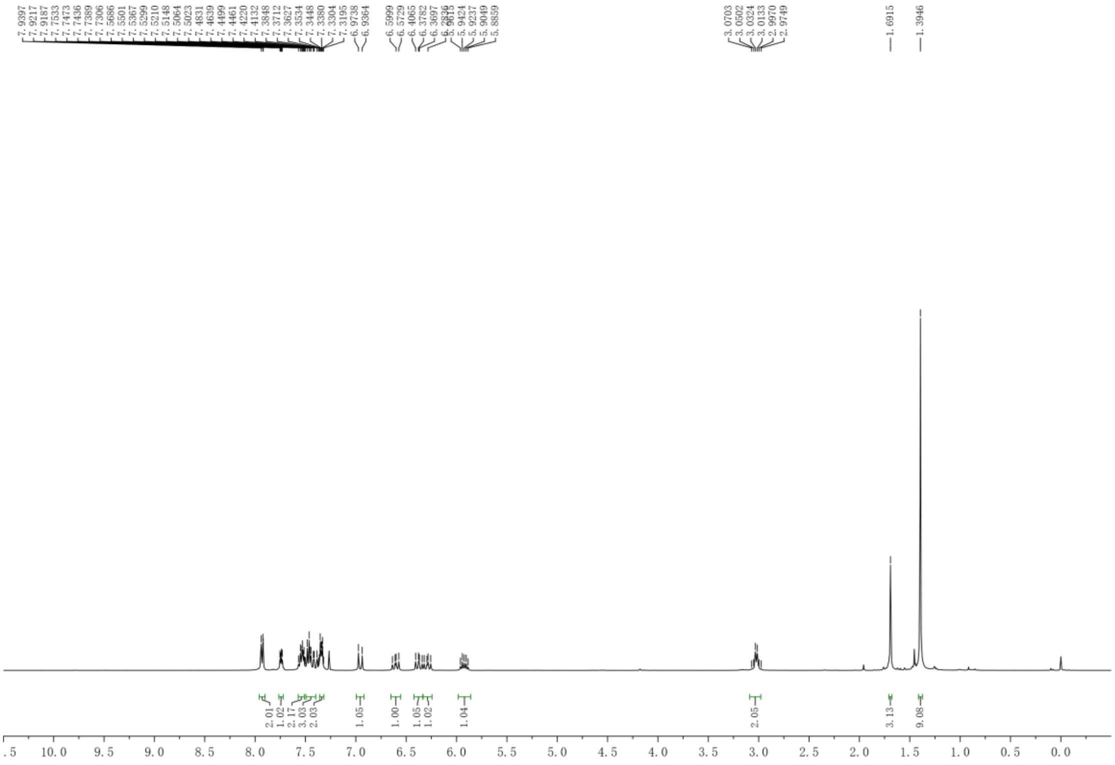

^{13}C NMR spectrum of **3a**

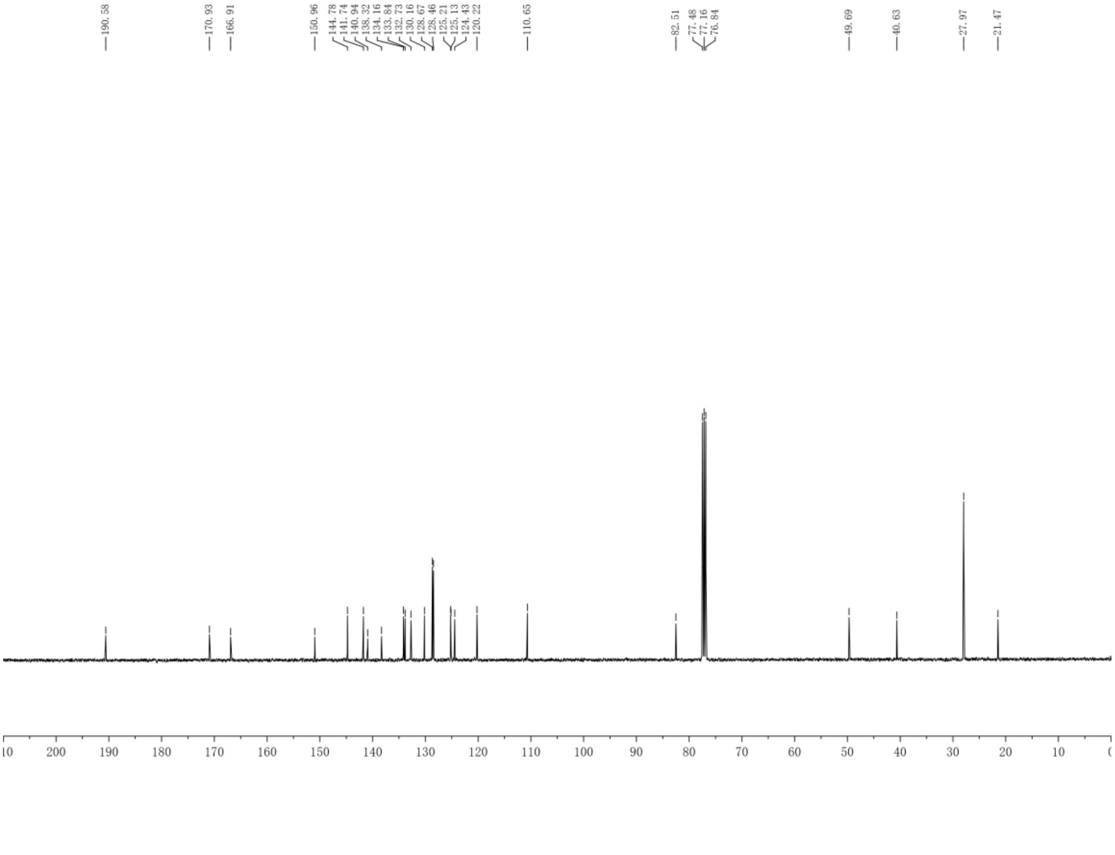

^{1}H NMR spectrum of **3b**

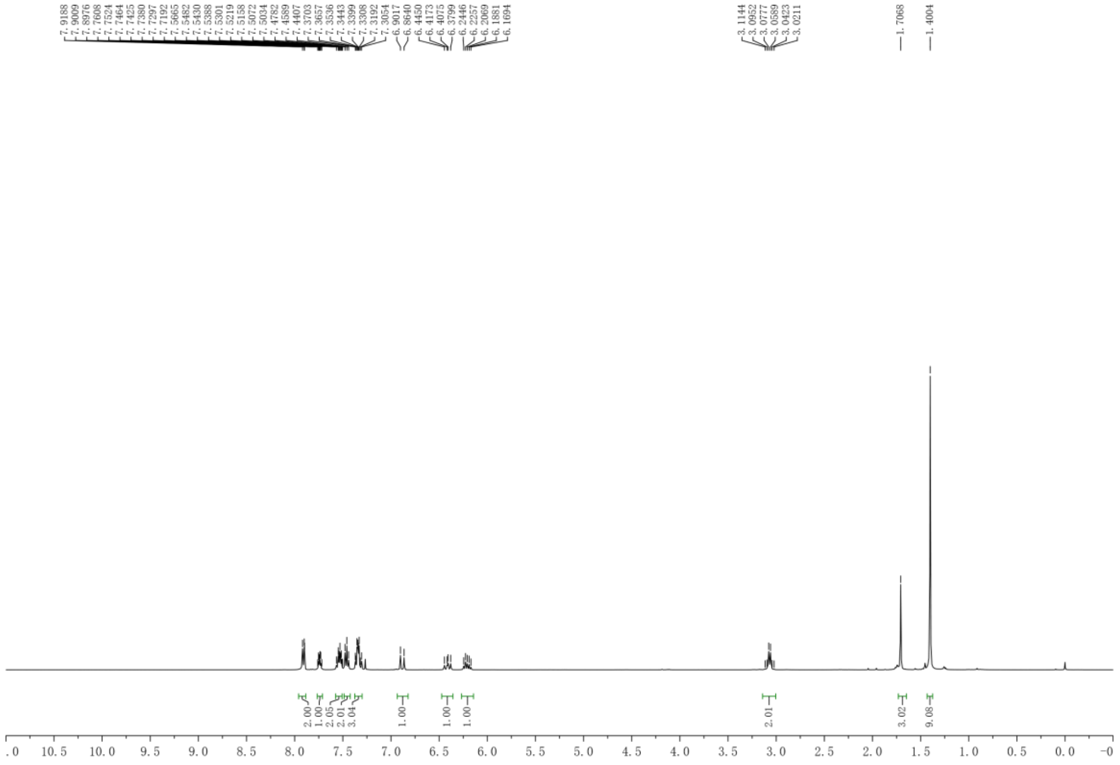

^{13}C NMR spectrum of **3b**

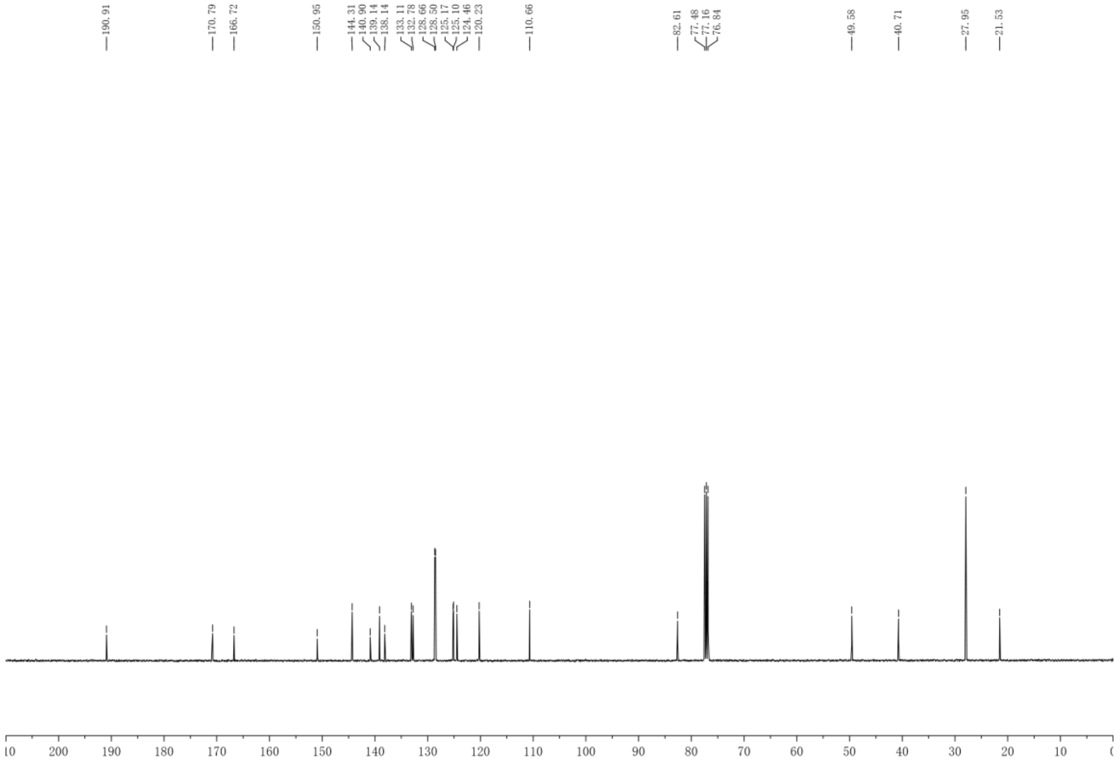

^{1}H NMR spectrum of **3c**

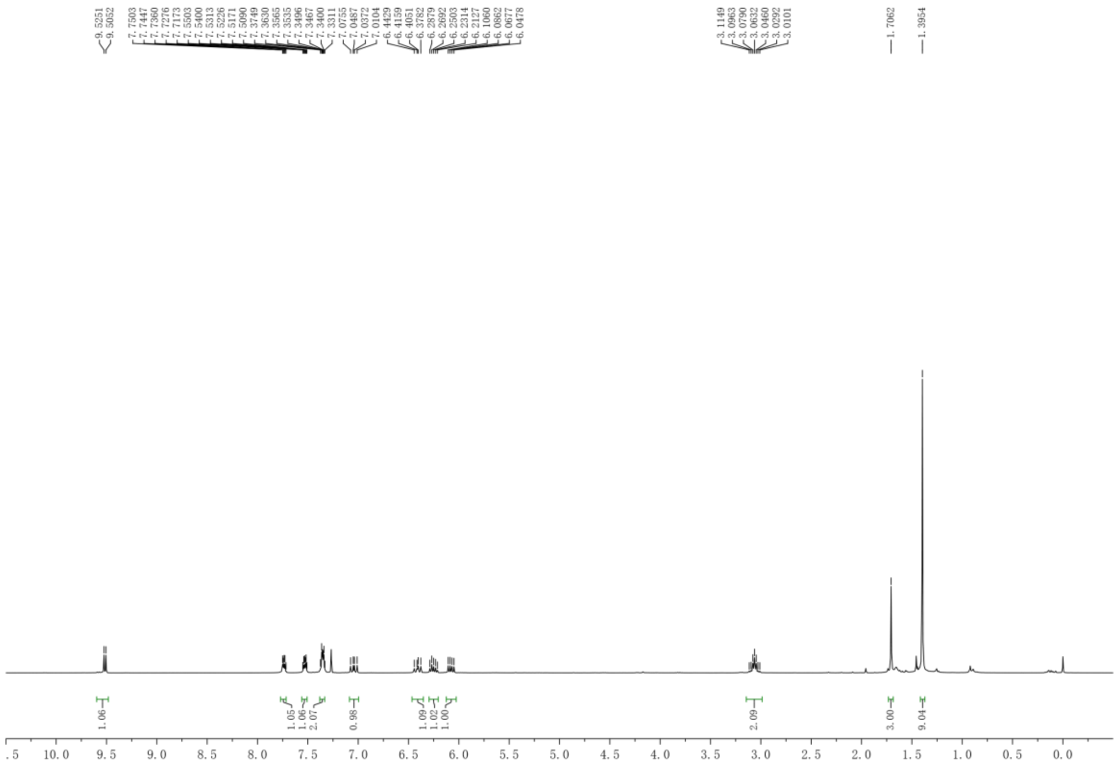

^{13}C NMR spectrum of **3c**

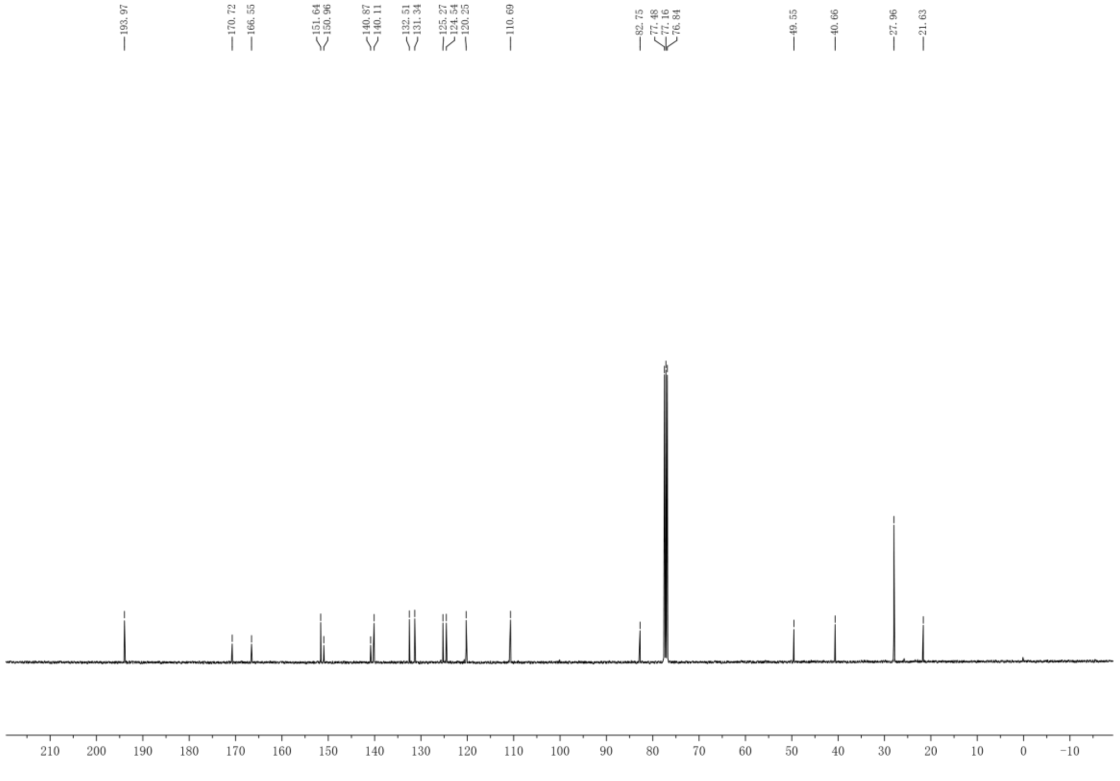

^{1}H NMR spectrum of **3d**

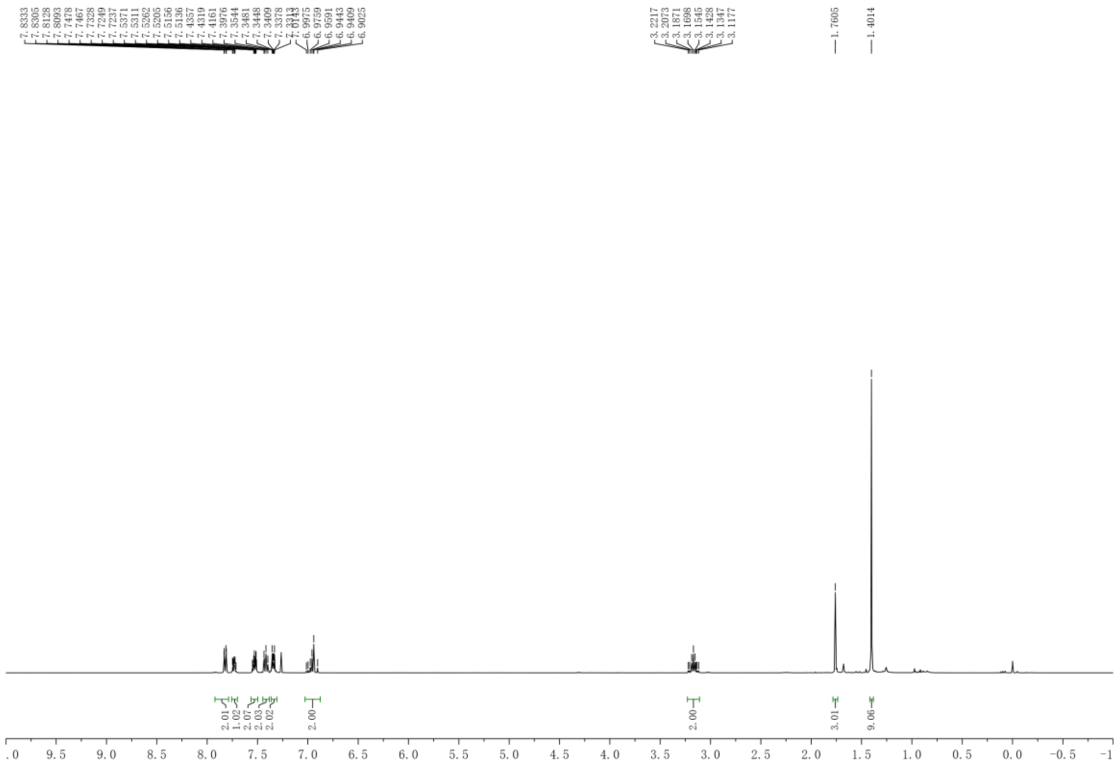

^{13}C NMR spectrum of **3d**

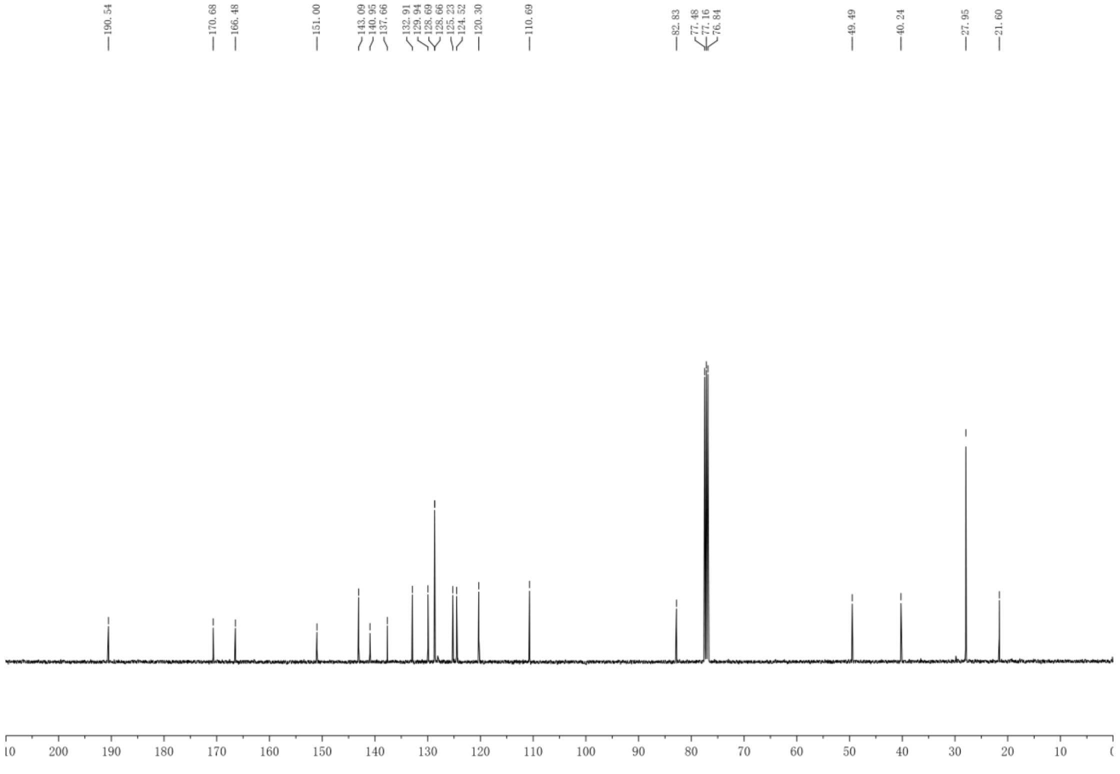

[1]**H NMR** spectrum of **3e**

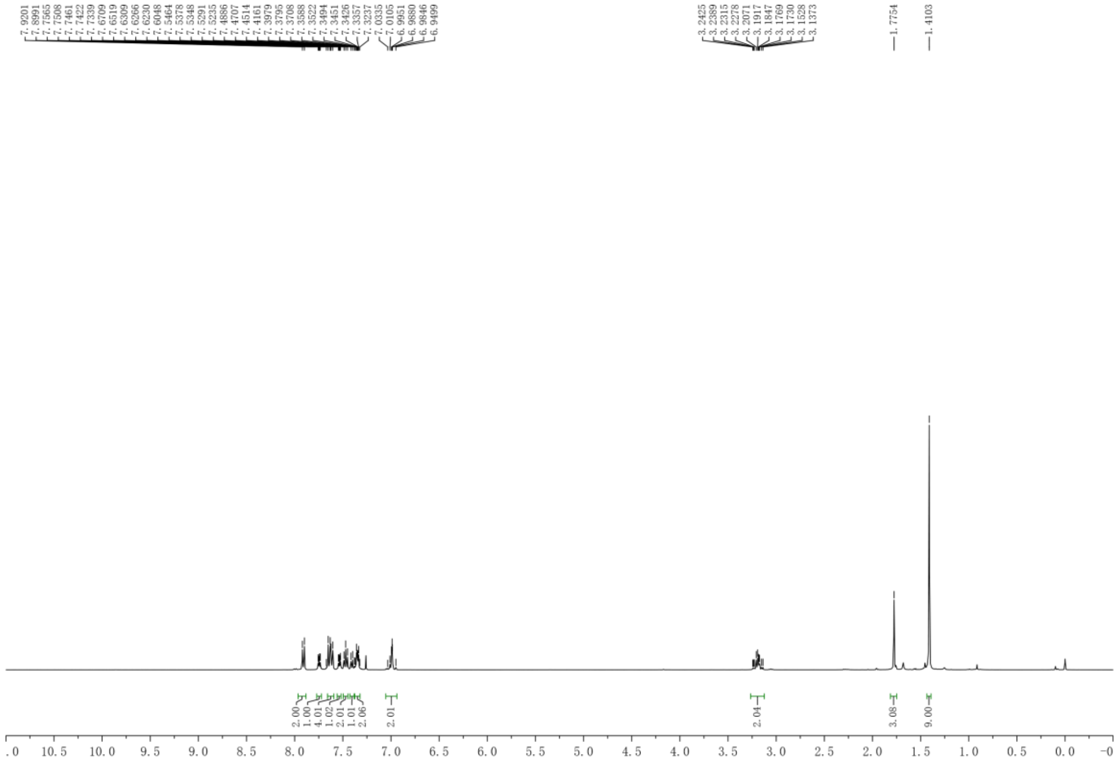

[13]**C NMR** spectrum of **3e**

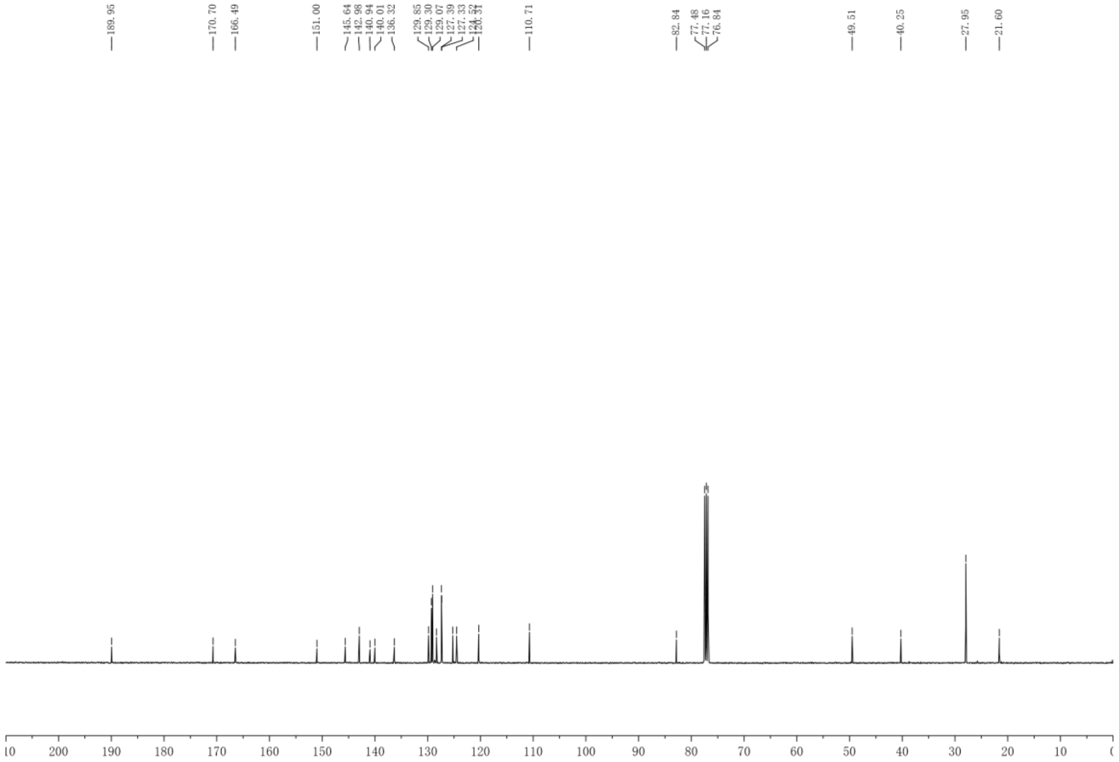

^{1}H NMR spectrum of **3f**

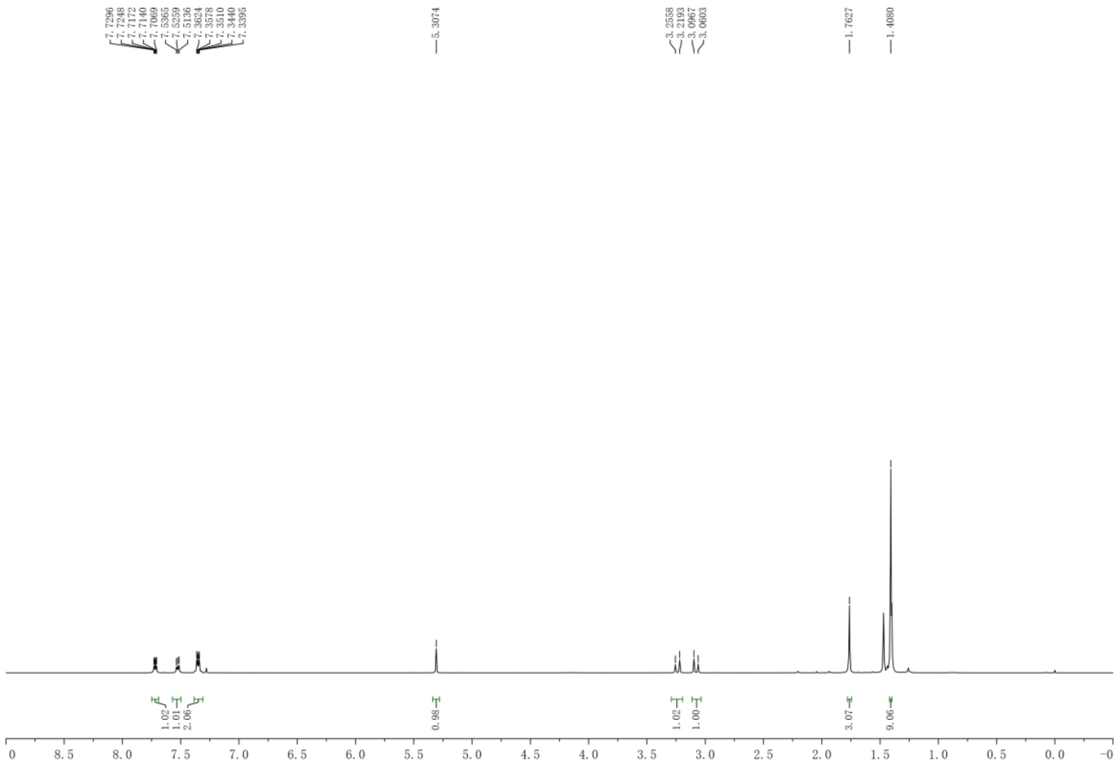

^{13}C NMR spectrum of **3f**

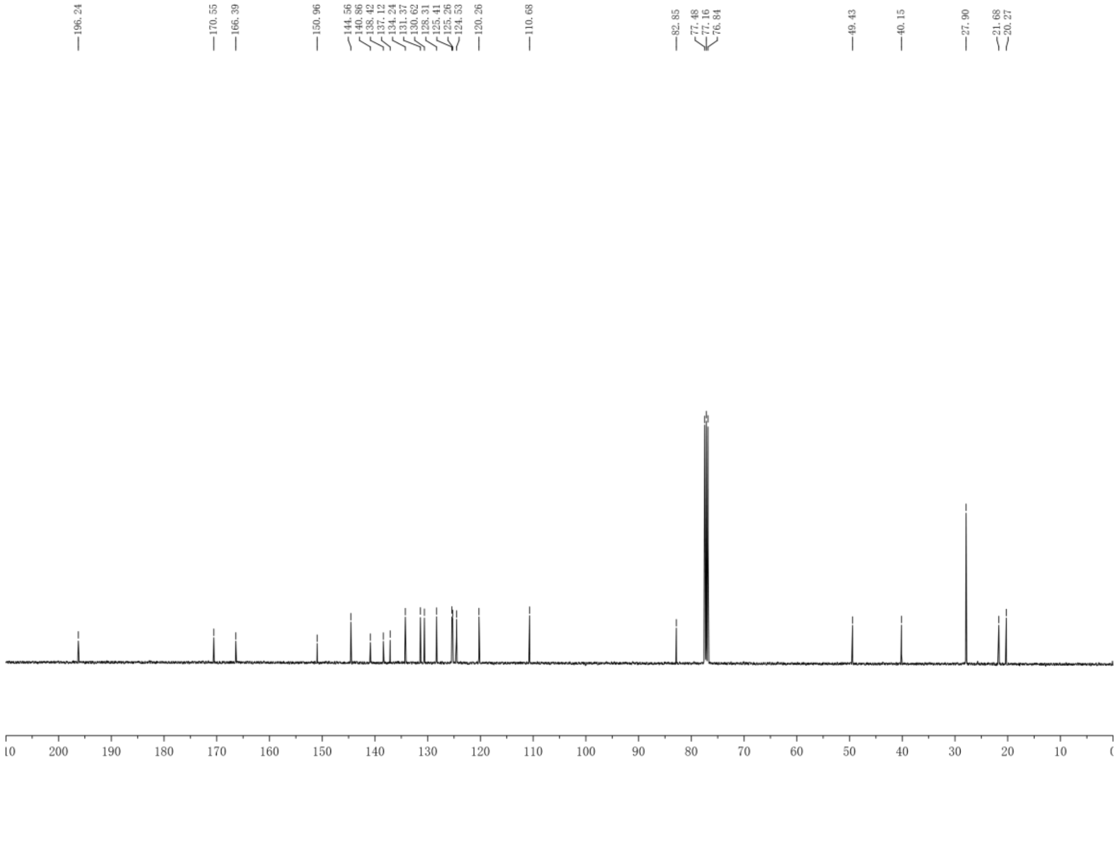

^{1}H NMR spectrum of 3g

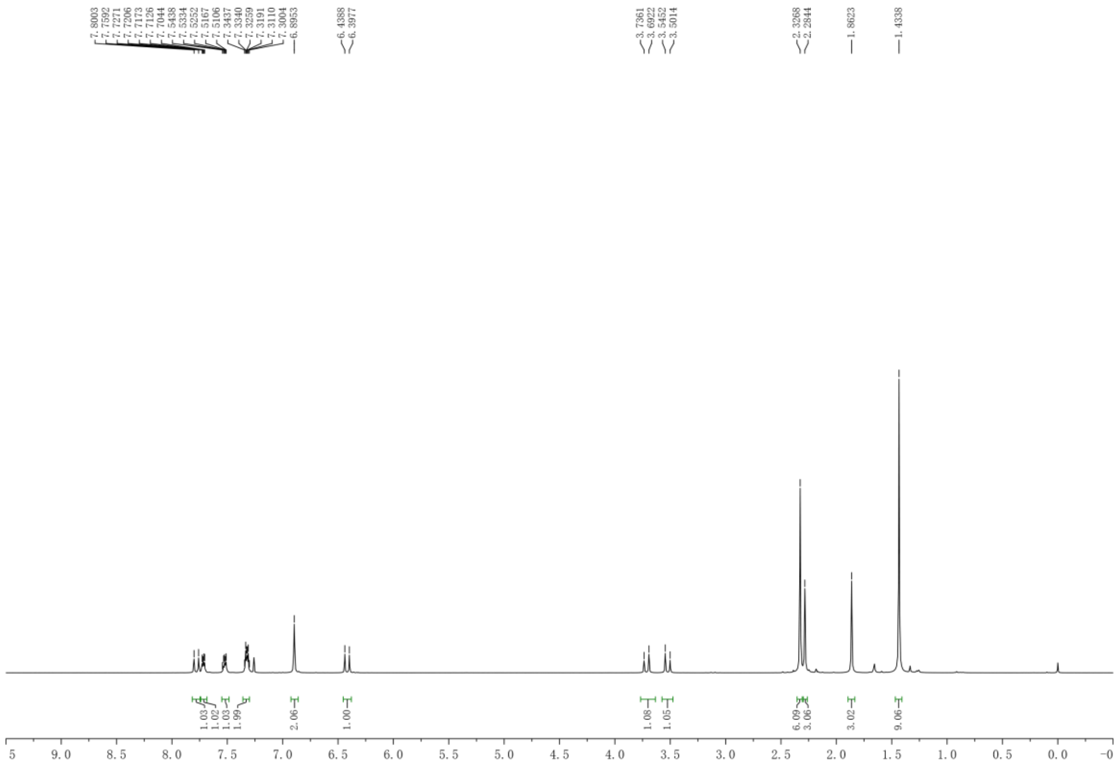

^{13}C NMR spectrum of 3g

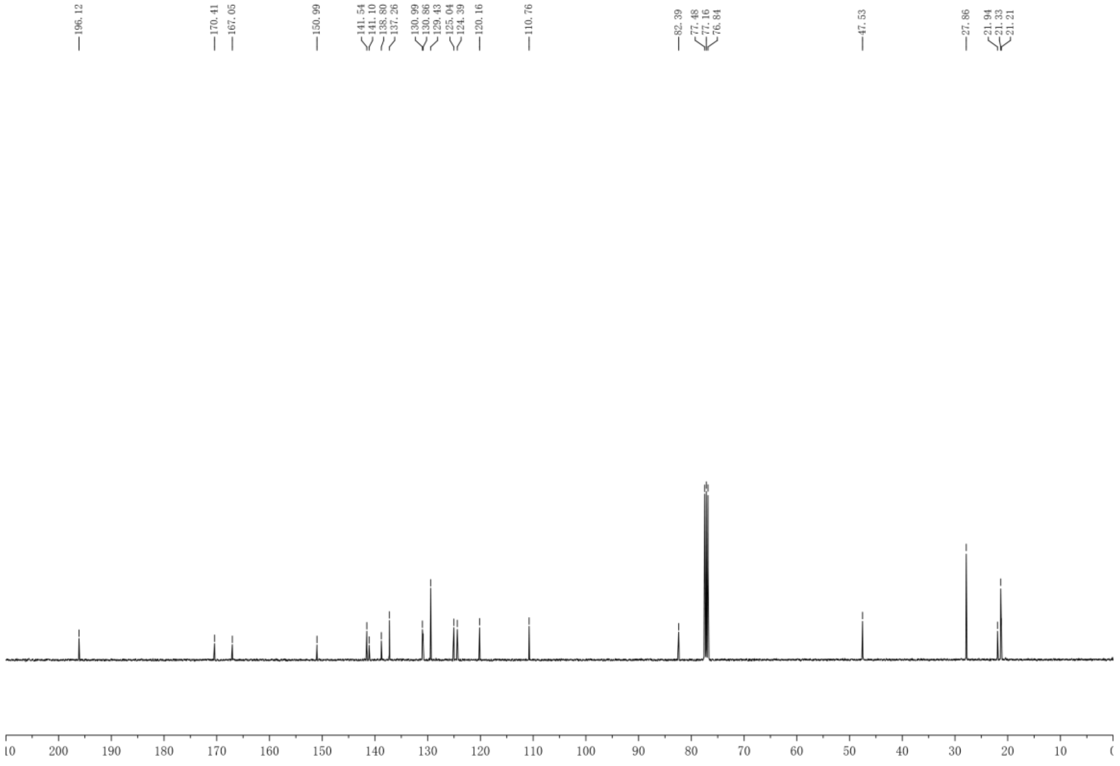

^{1}H NMR spectrum of **3h**

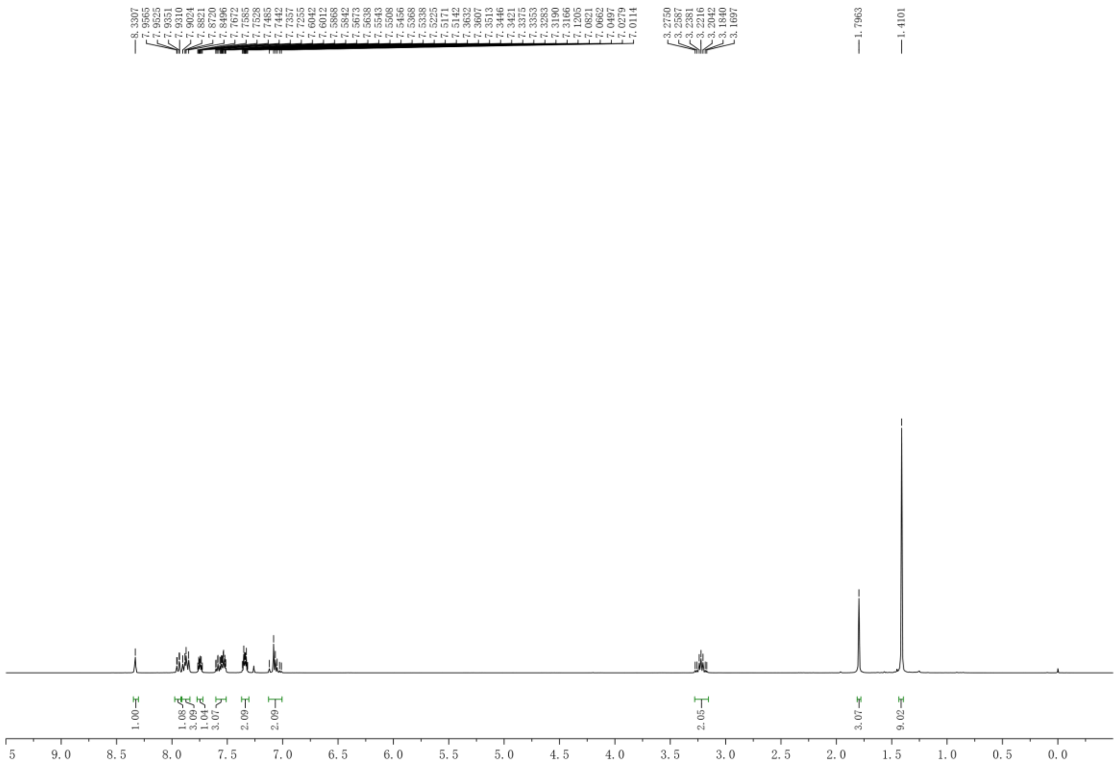

^{13}C NMR spectrum of **3h**

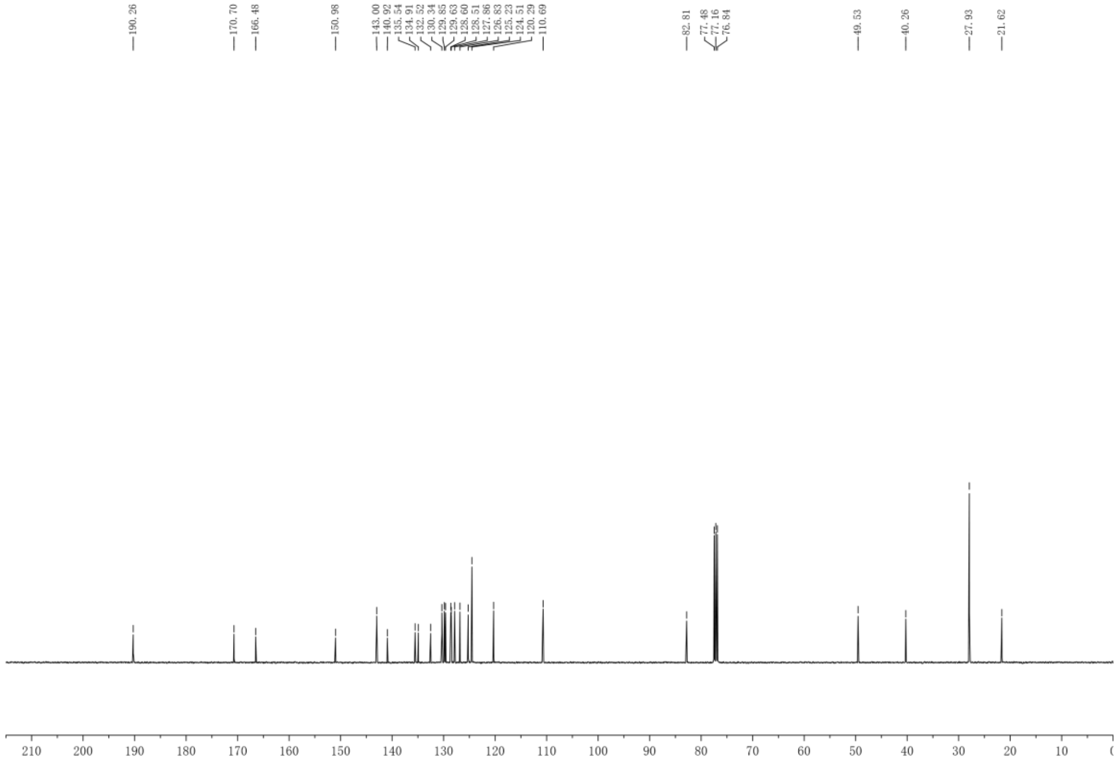

^{1}H NMR spectrum of **3i**

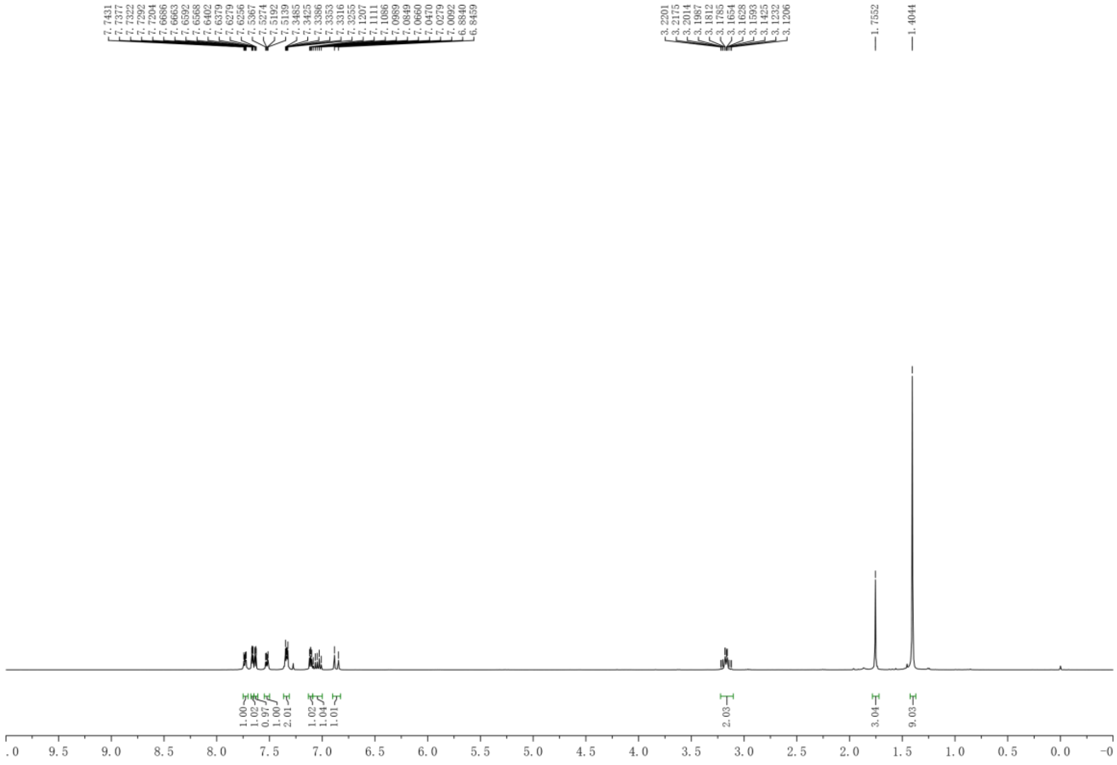

^{13}C NMR spectrum of **3i**

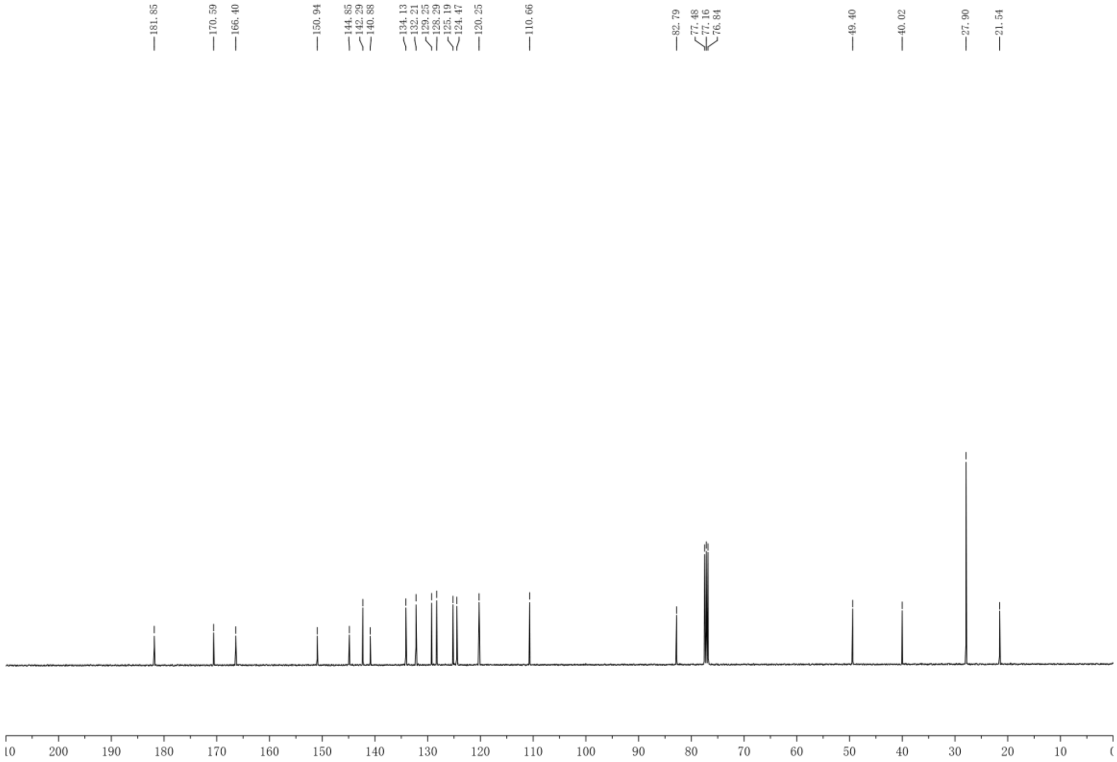

^{1}H NMR spectrum of **3j**

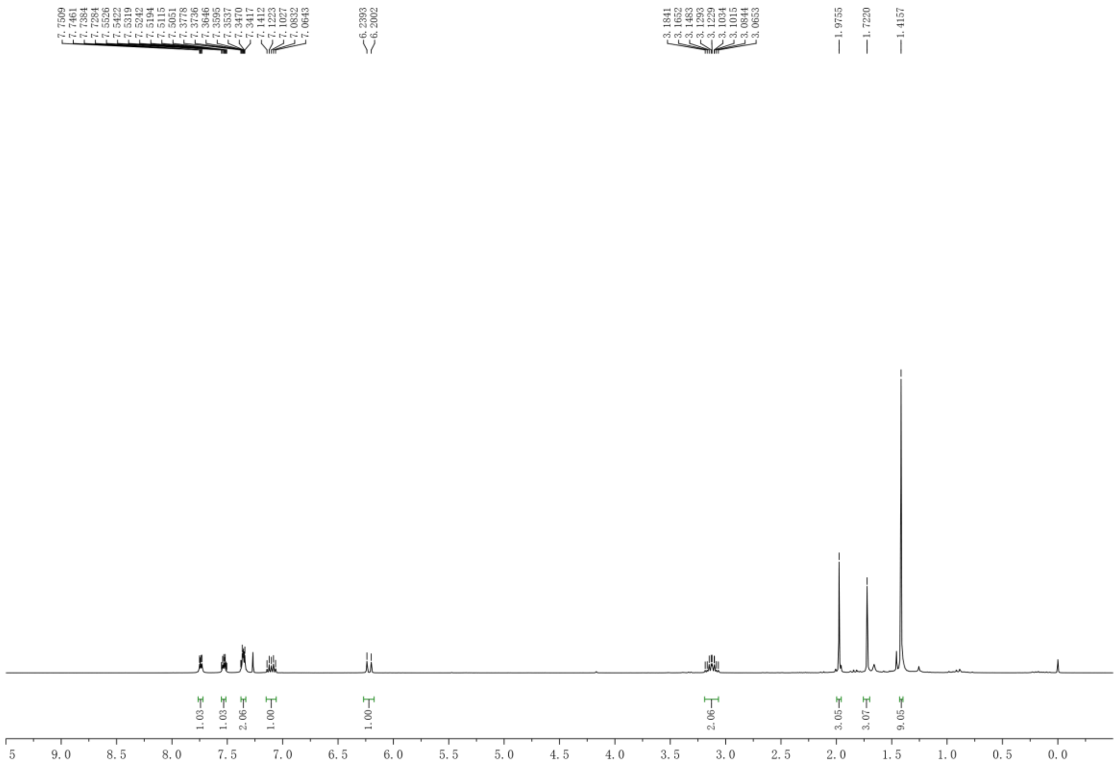

^{13}C NMR spectrum of **3j**

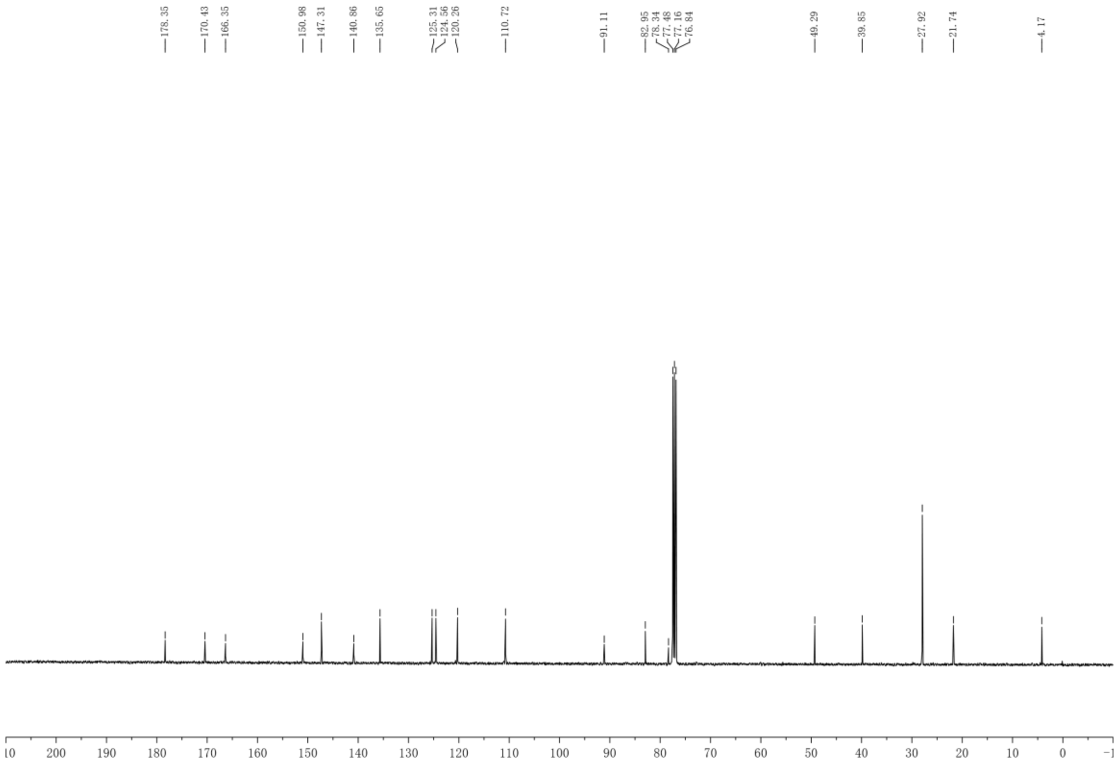

^{1}H NMR spectrum of **3k**

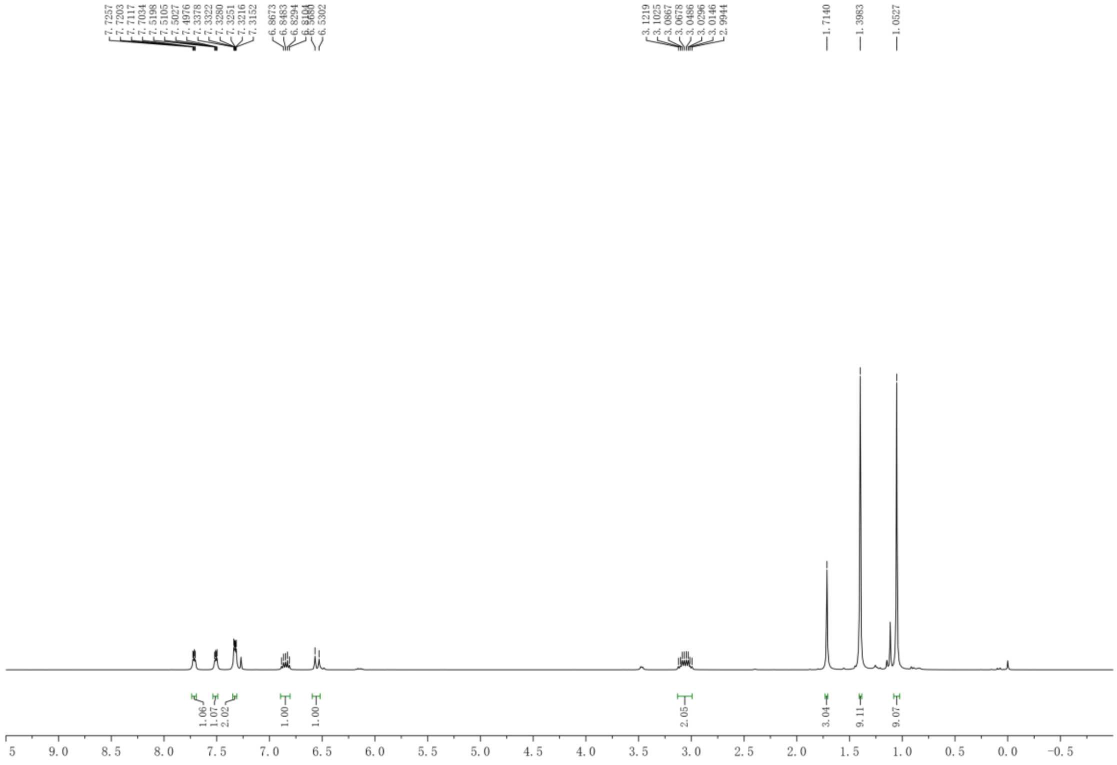

^{13}C NMR spectrum of **3k**

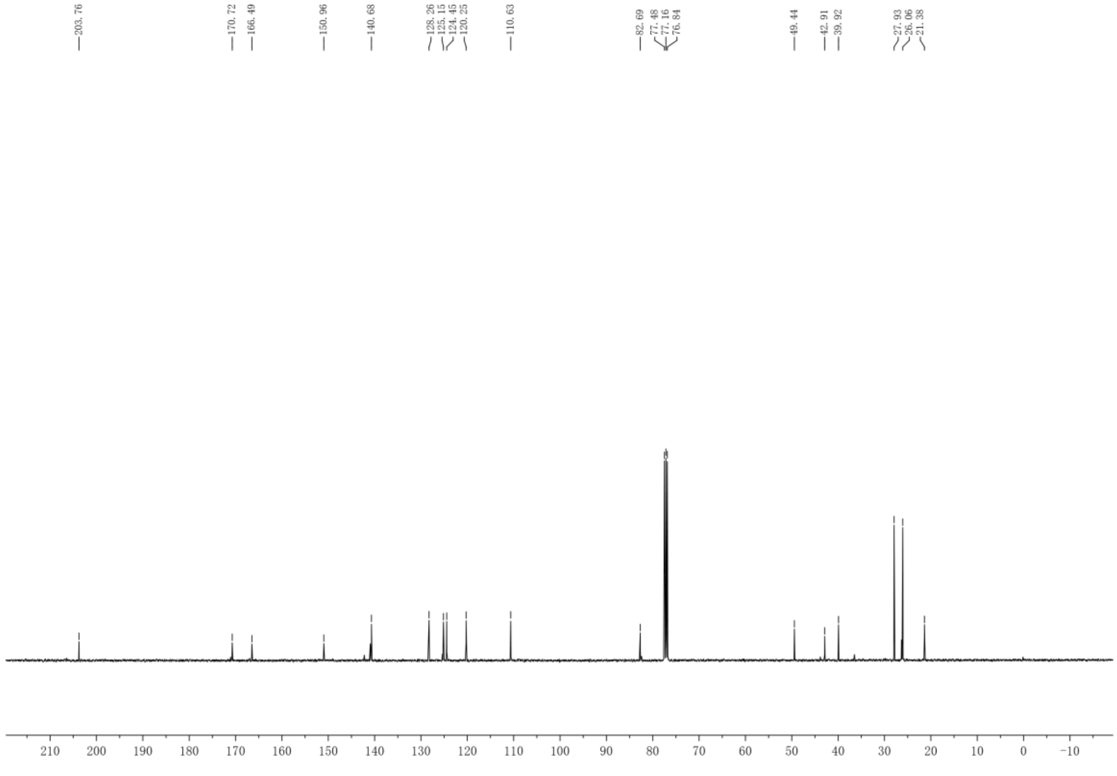

^{1}**H NMR** spectrum of **3l**

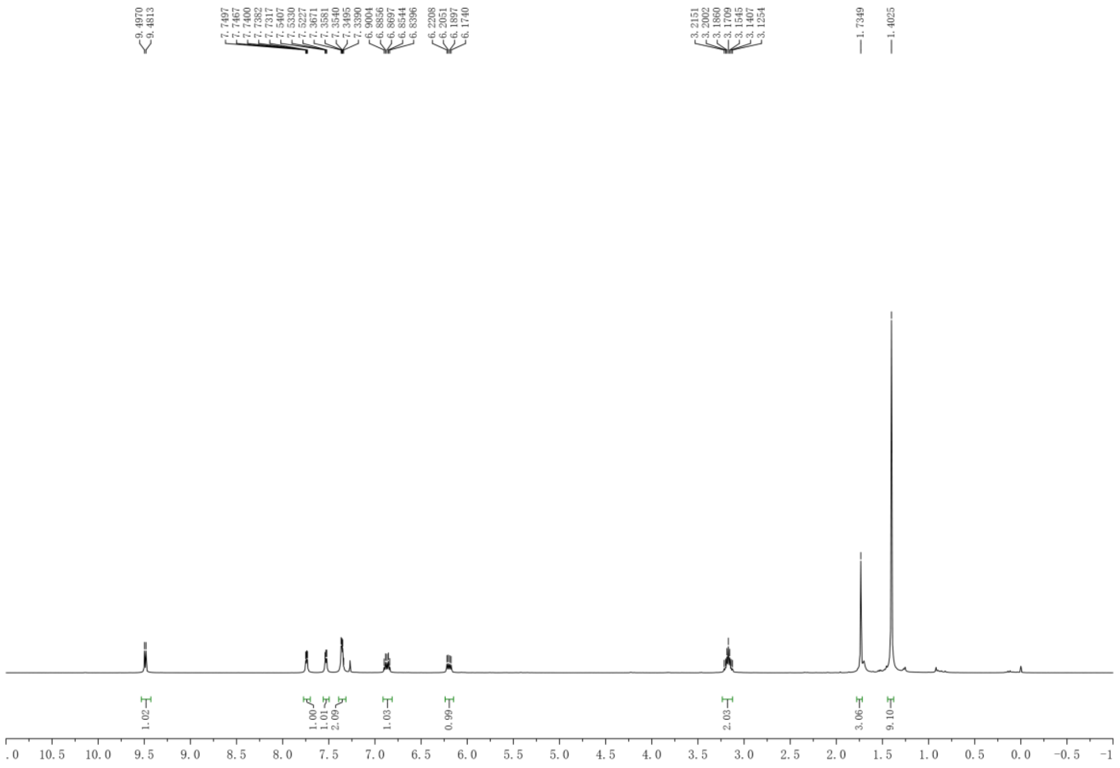

^{13}**C NMR** spectrum of **3l**

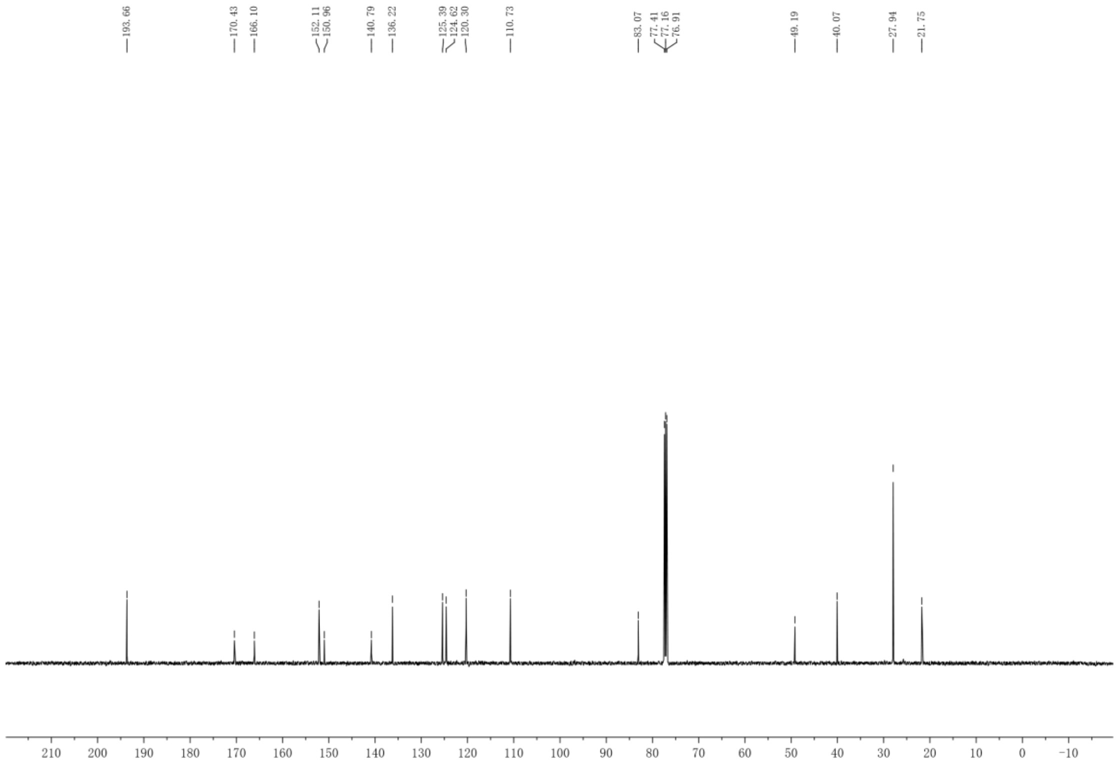

^{1}H NMR spectrum of **3m**

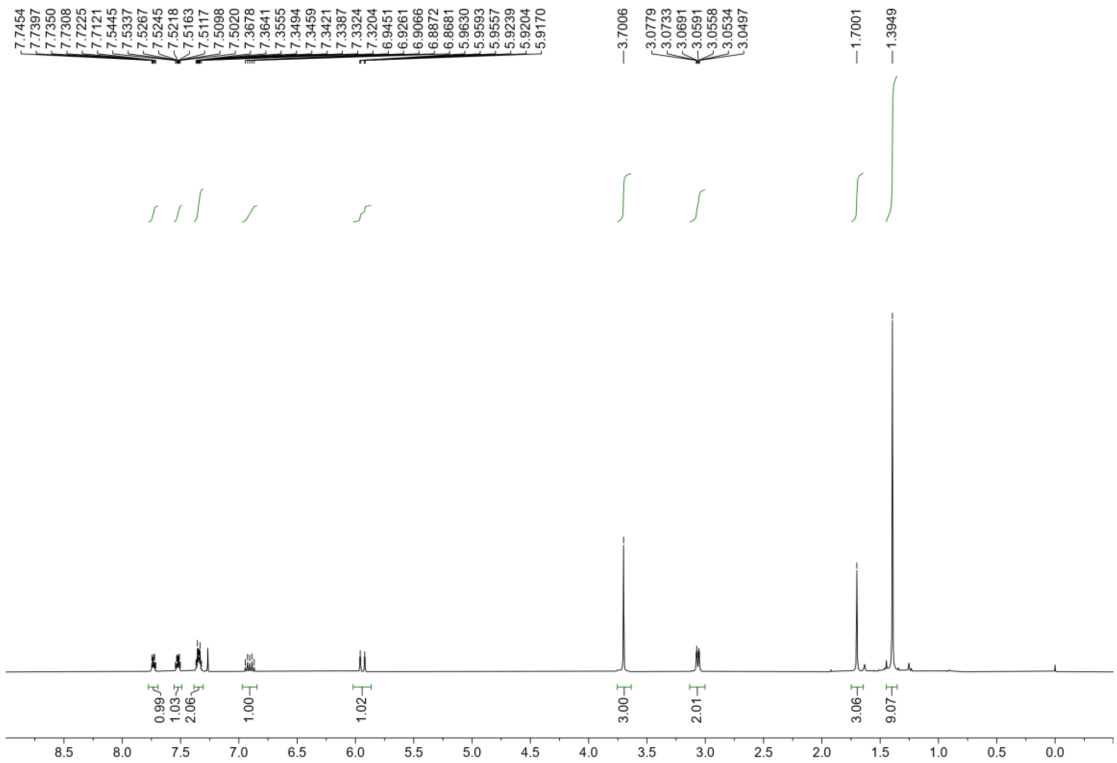

^{13}C NMR spectrum of **3m**

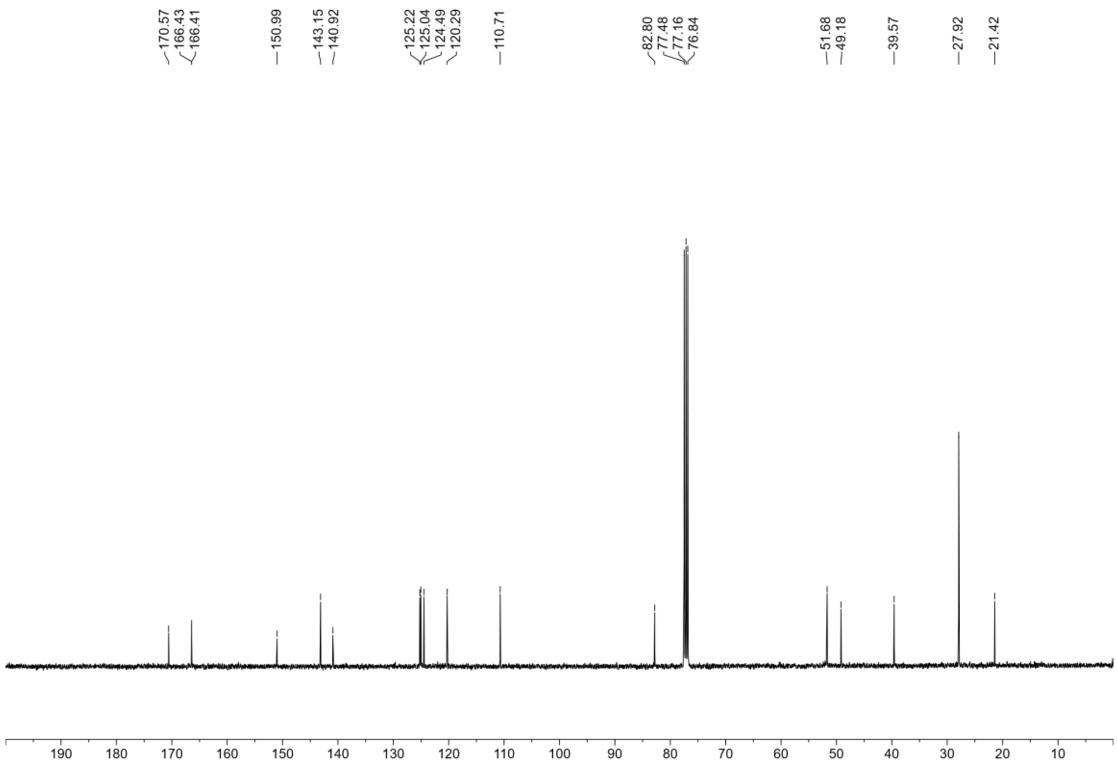

[1]**H NMR** spectrum of **3n**

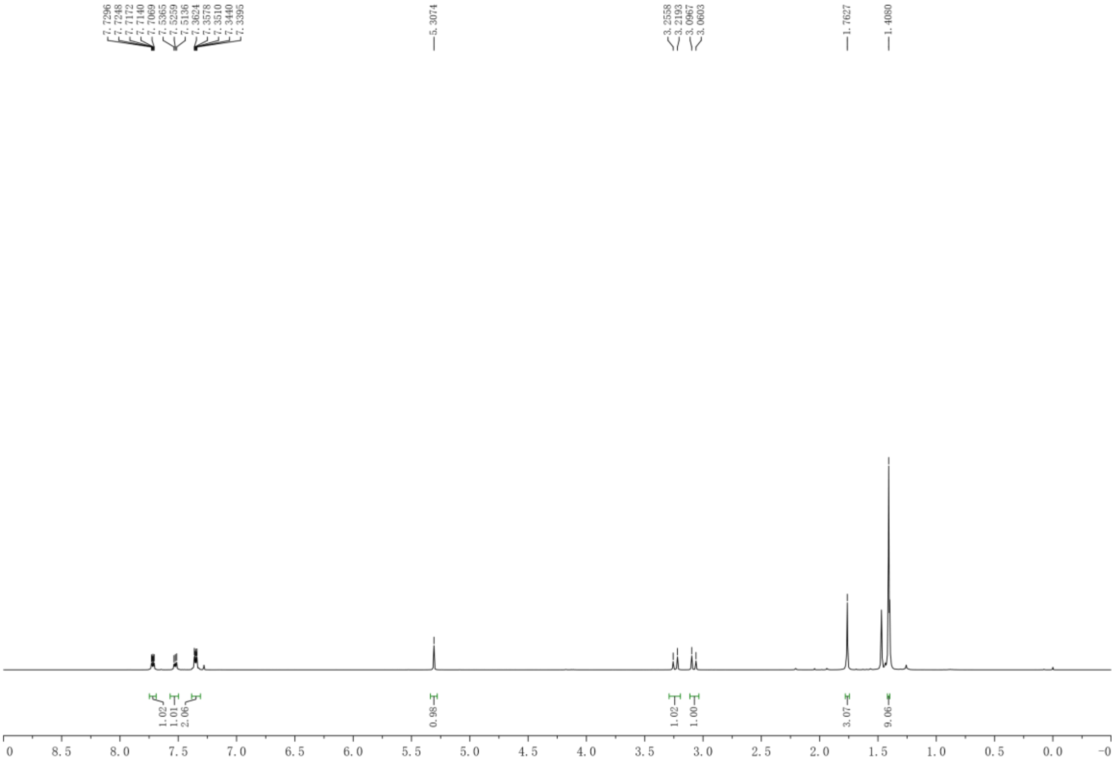

[13]**C NMR** spectrum of **3n**

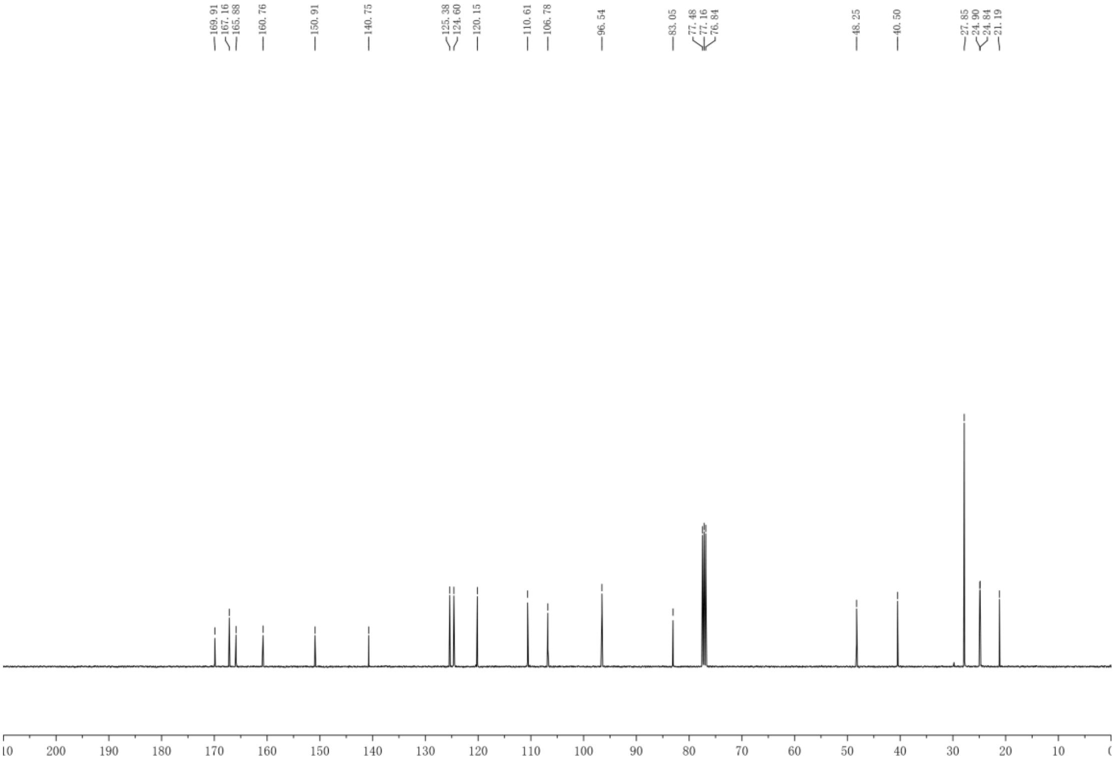

^{1}H NMR spectrum of **3o**

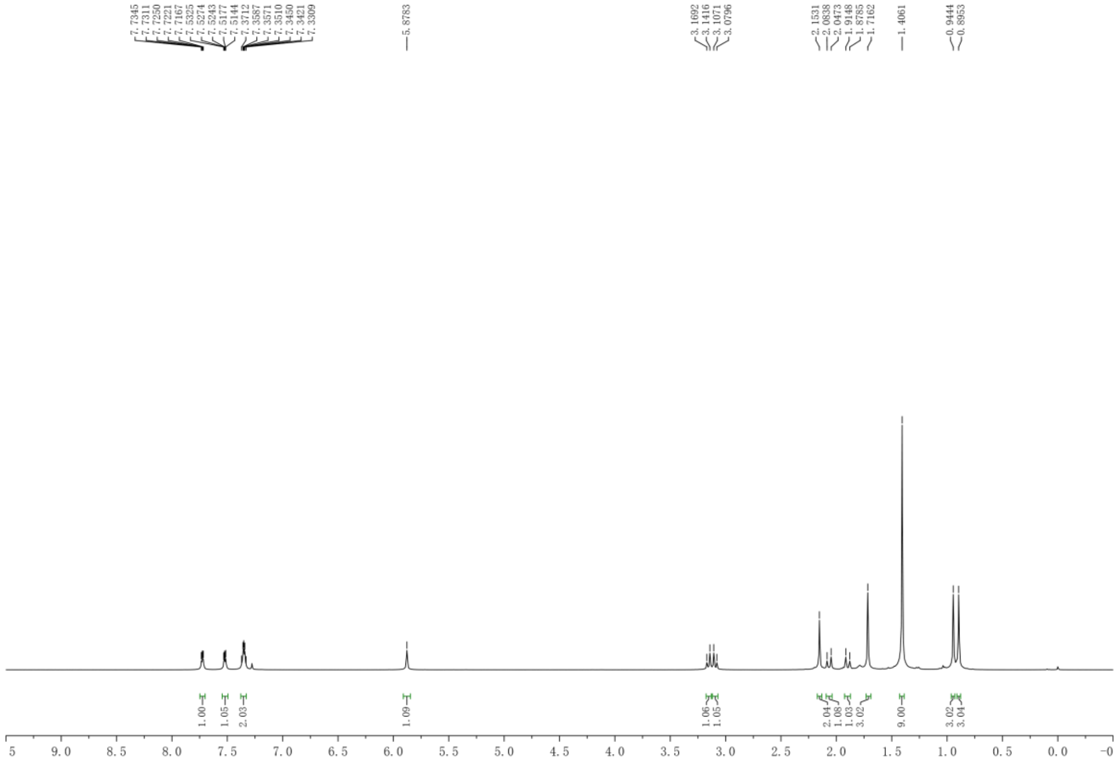

^{13}C NMR spectrum of **3o**

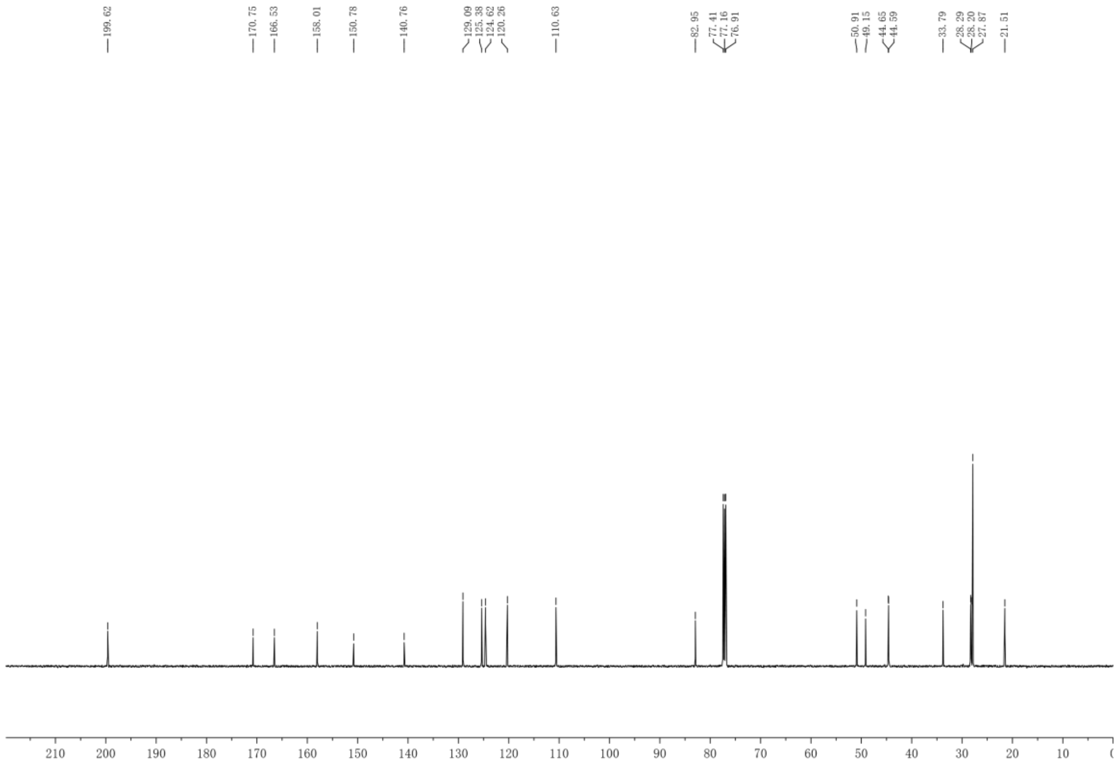

^{1}H NMR spectrum of **3p**

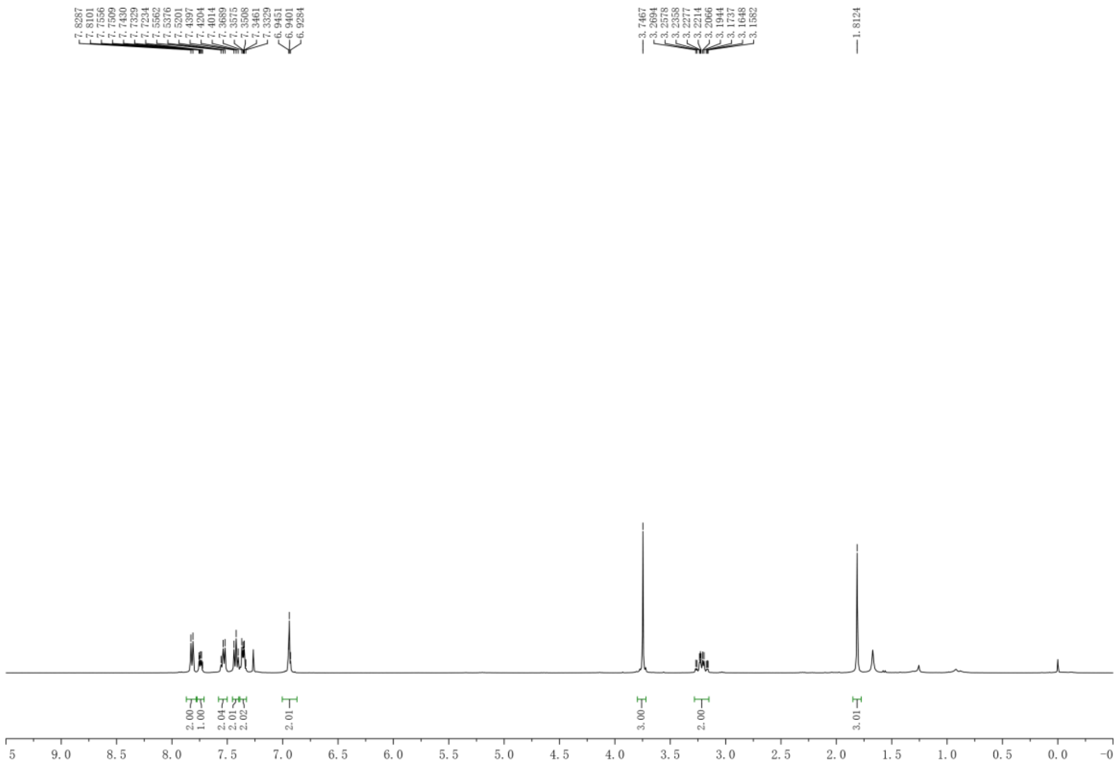

^{13}C NMR spectrum of **3p**

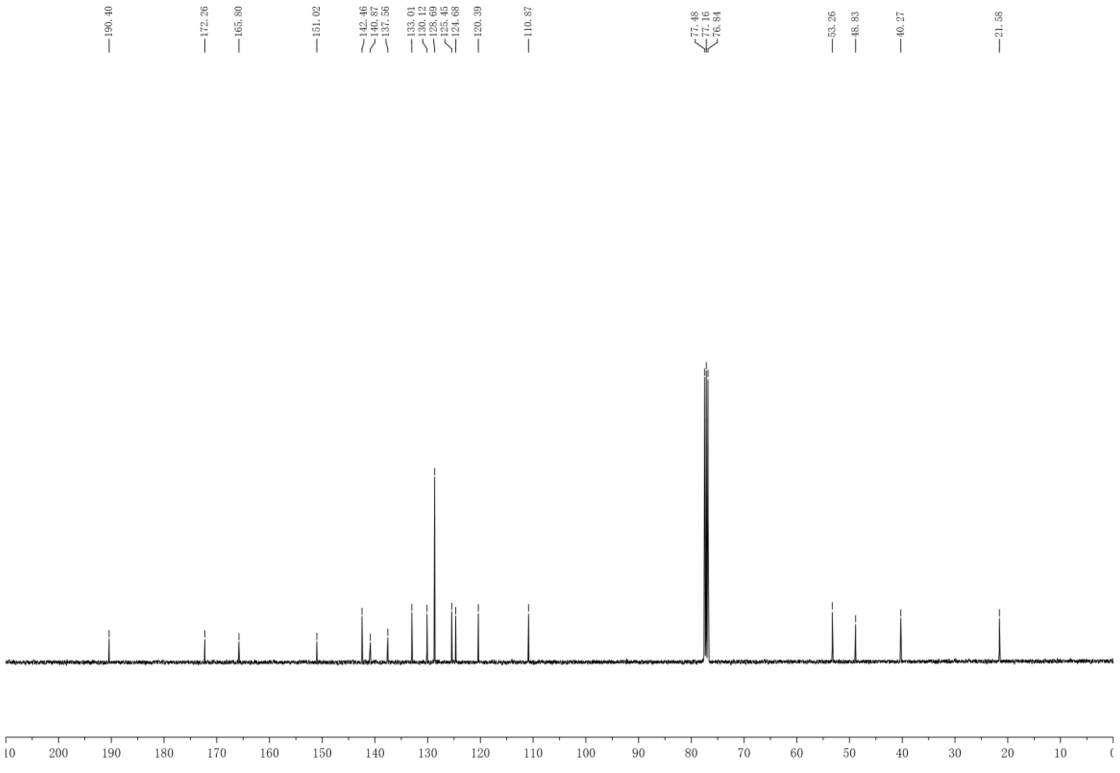

[1]**H NMR** spectrum of **3q**

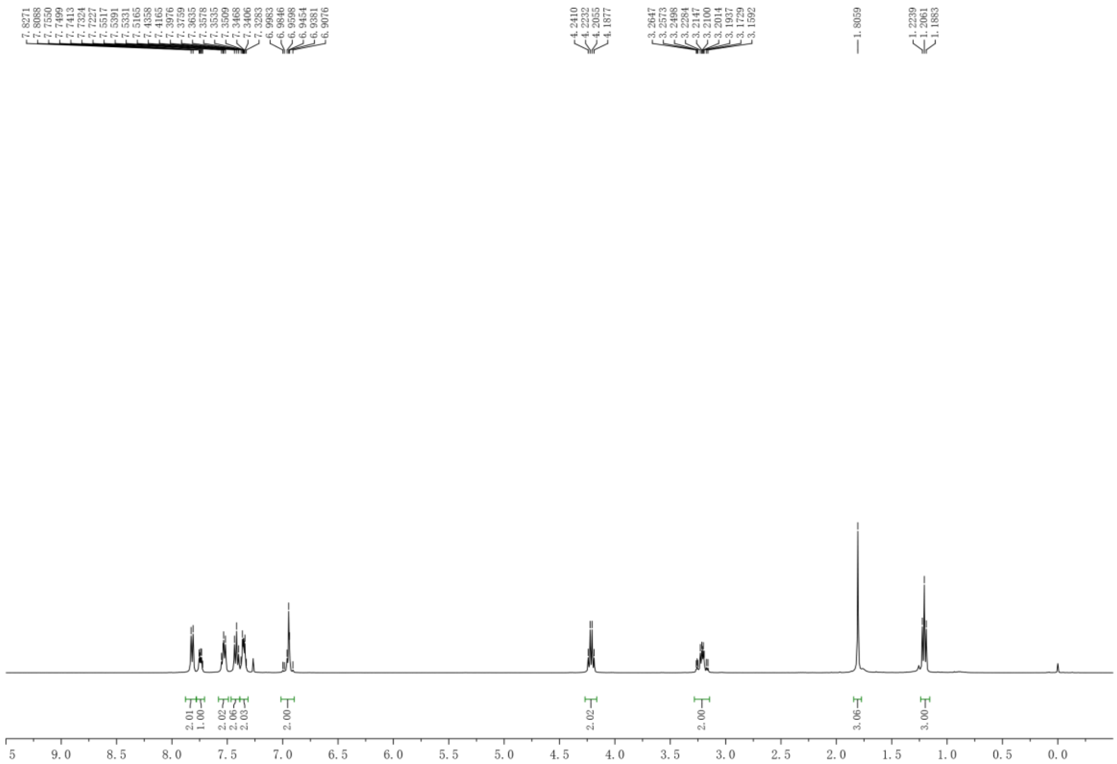

[13]**C NMR** spectrum of **3q**

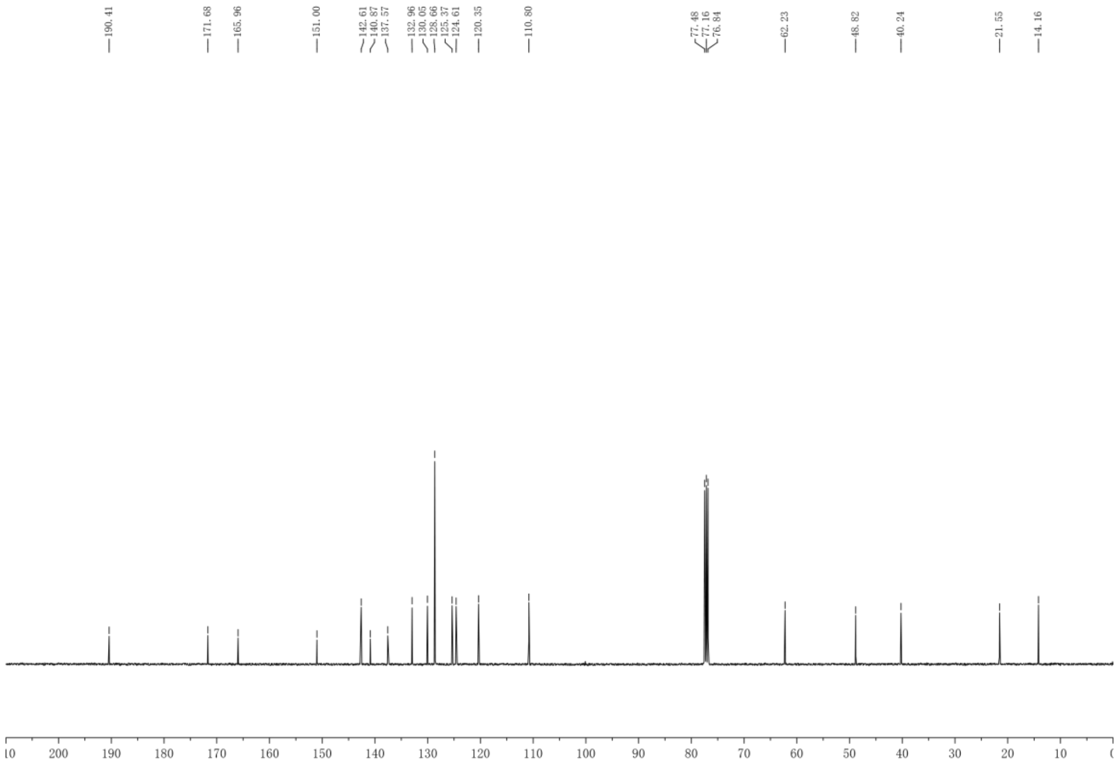

^{1}H NMR spectrum of **3r**

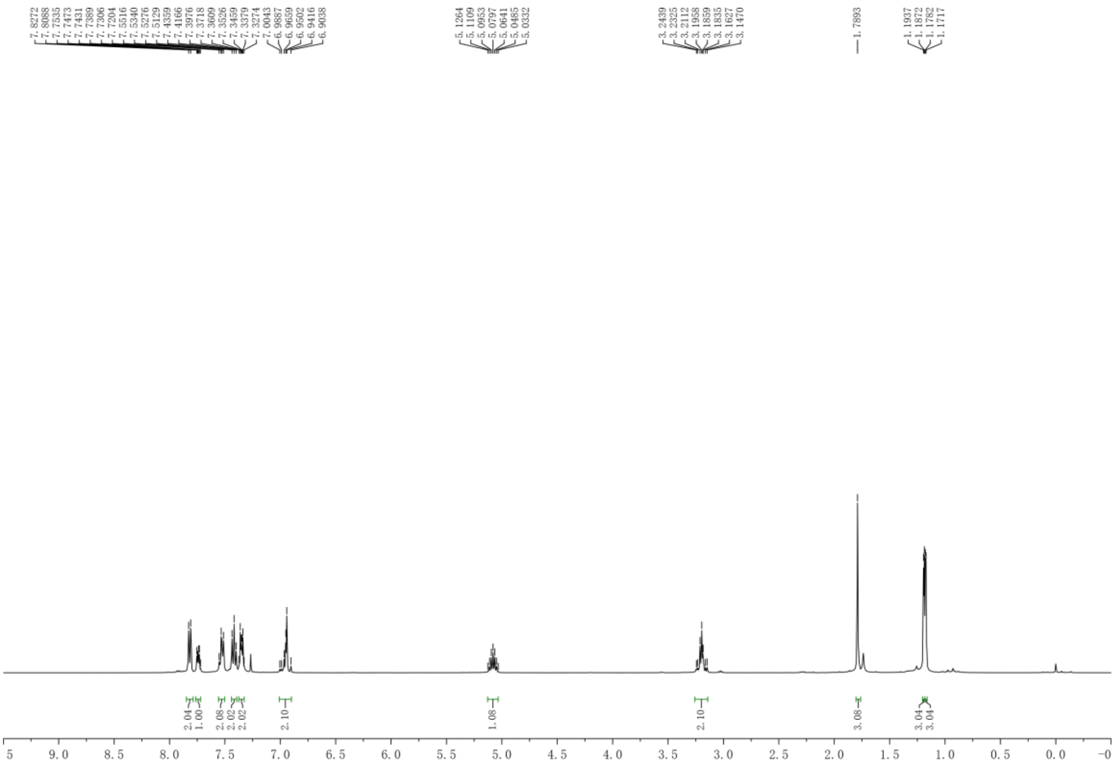

^{13}C NMR spectrum of **3r**

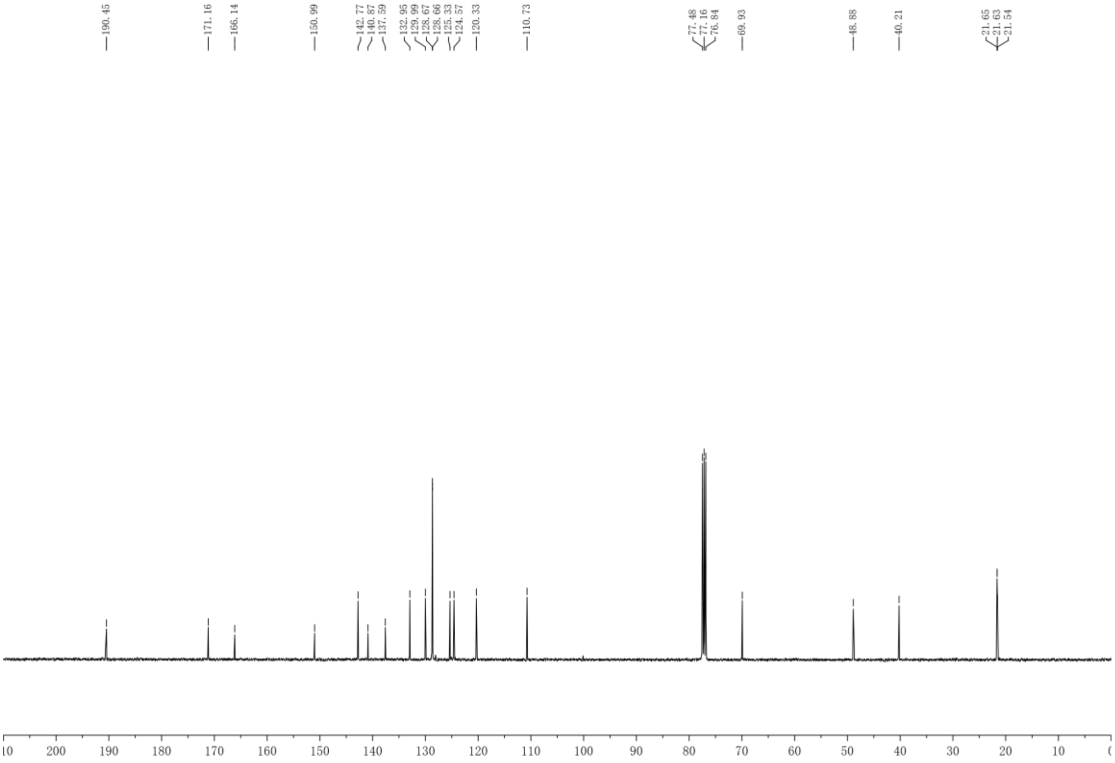

^{1}H NMR spectrum of **3s**

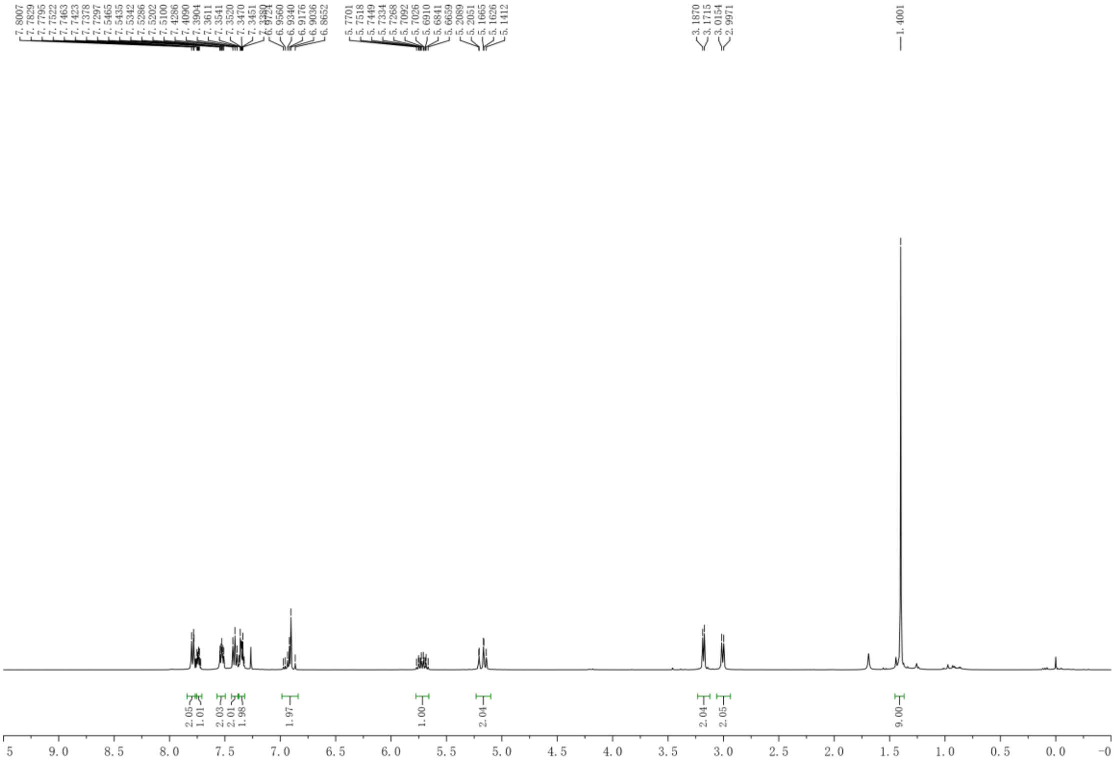

^{13}C NMR spectrum of **3s**

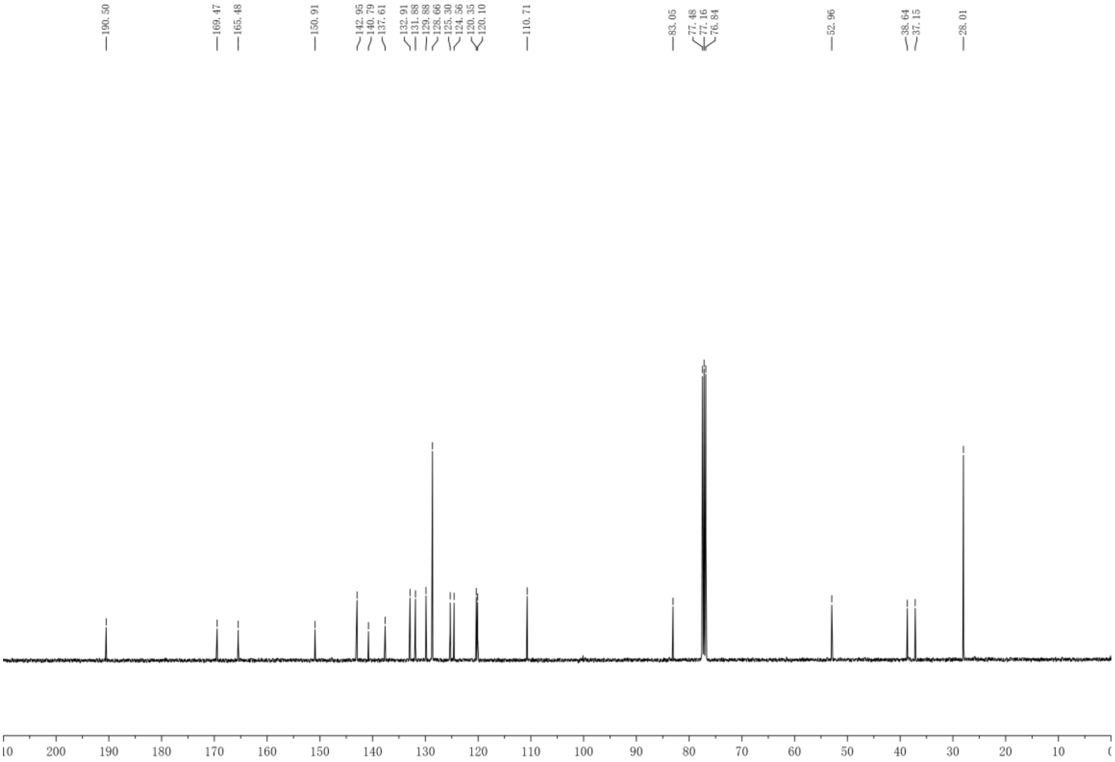

^{1}H NMR spectrum of **3t**

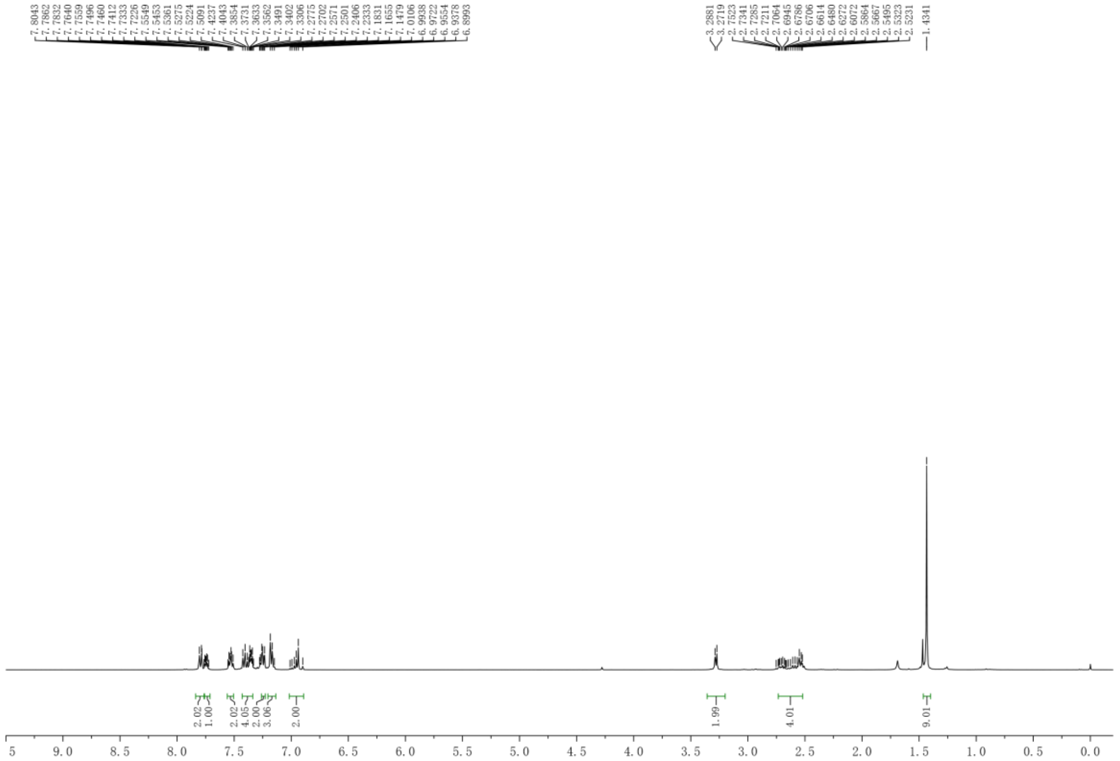

^{13}C NMR spectrum of **3t**

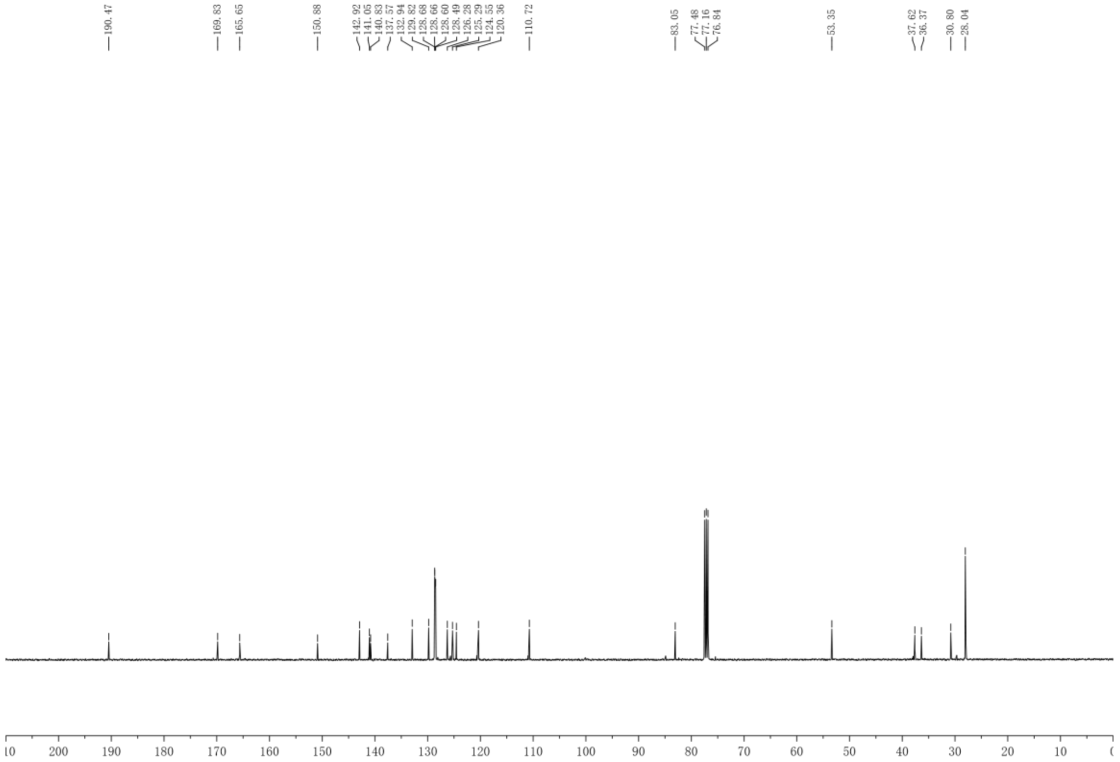

¹H NMR spectrum of **3u**

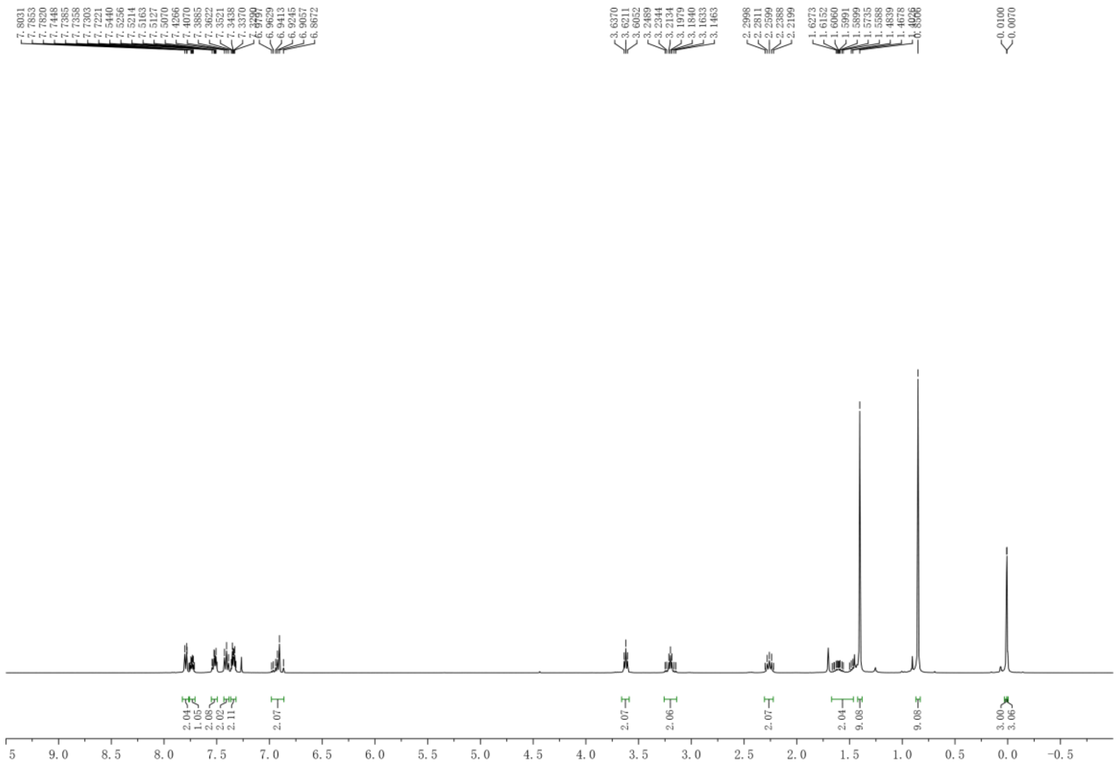

¹³C NMR spectrum of **3u**

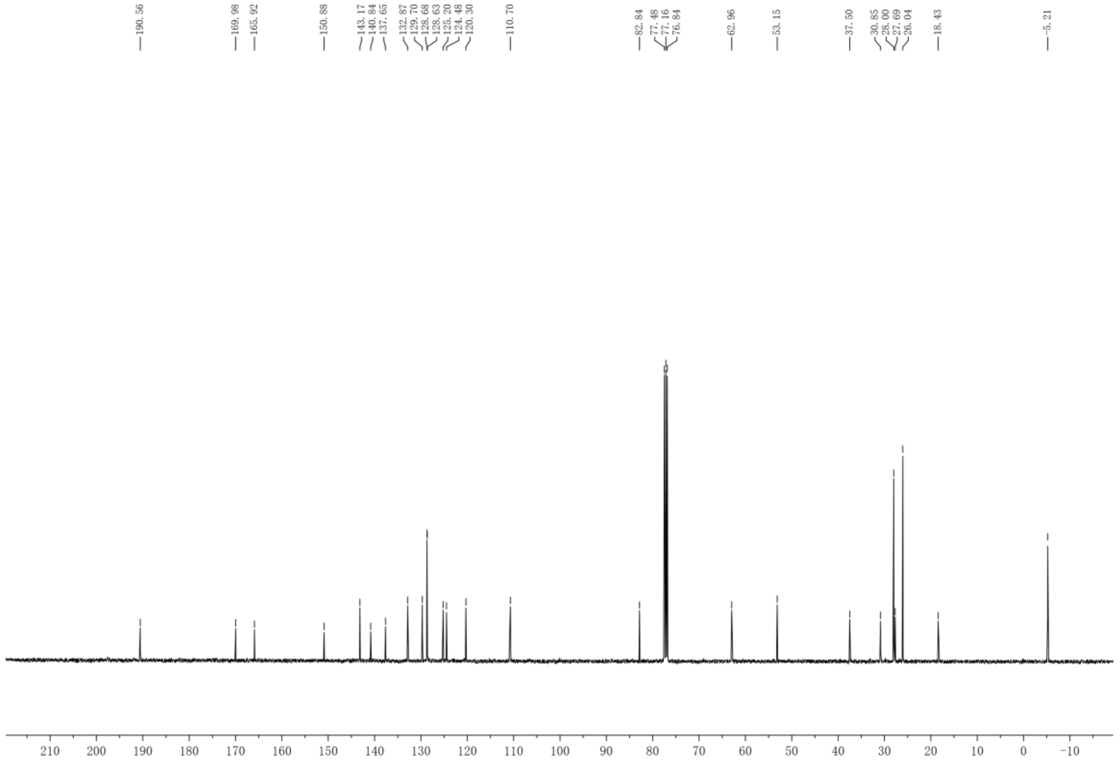

[1]**H NMR** spectrum of **3v**

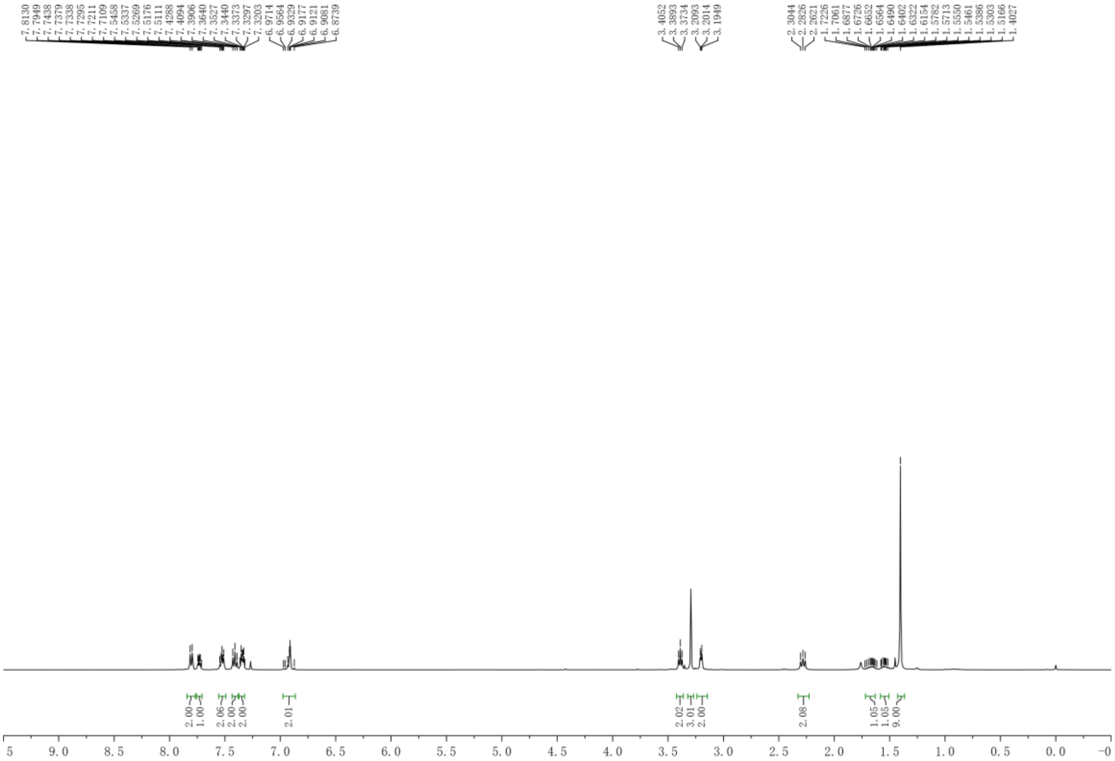

[13]**C NMR** spectrum of **3v**

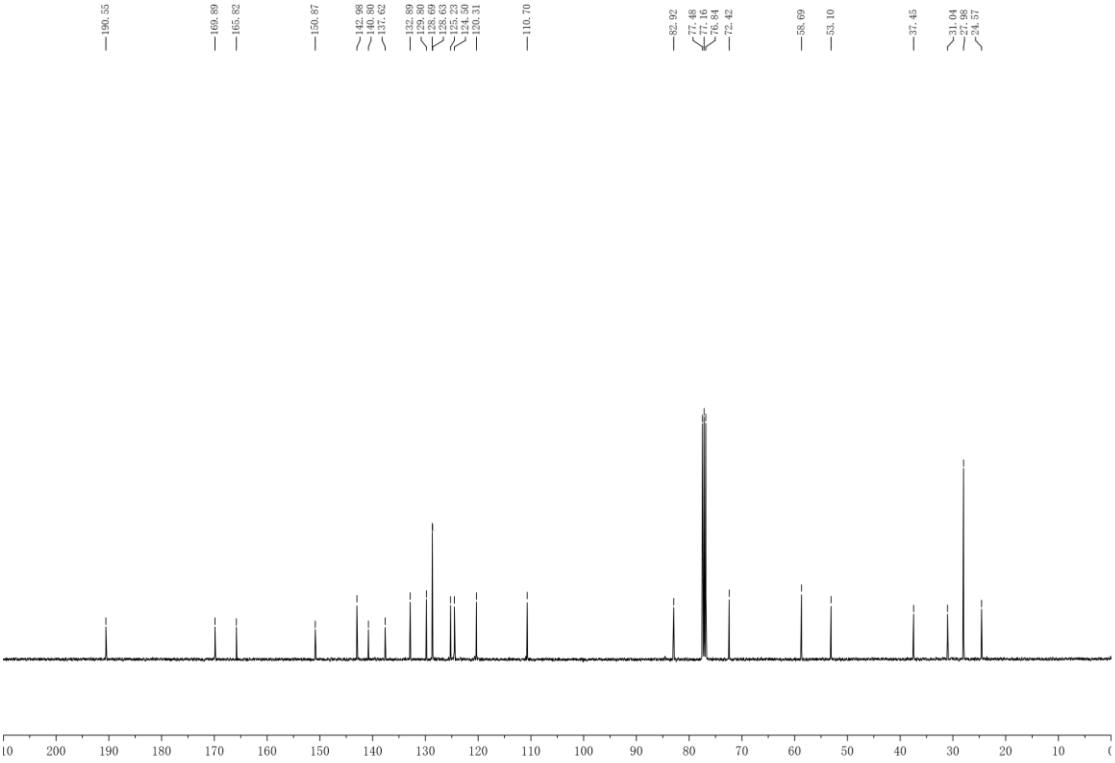

^{1}H NMR spectrum of **3w**

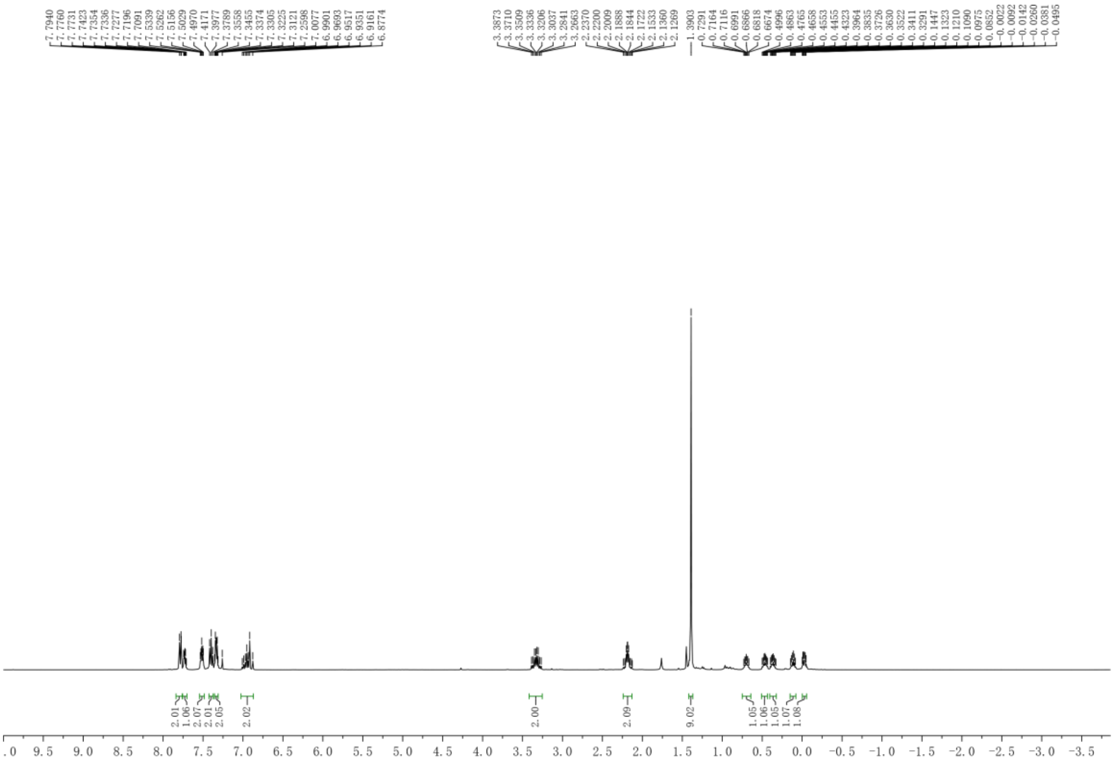

^{13}C NMR spectrum of **3w**

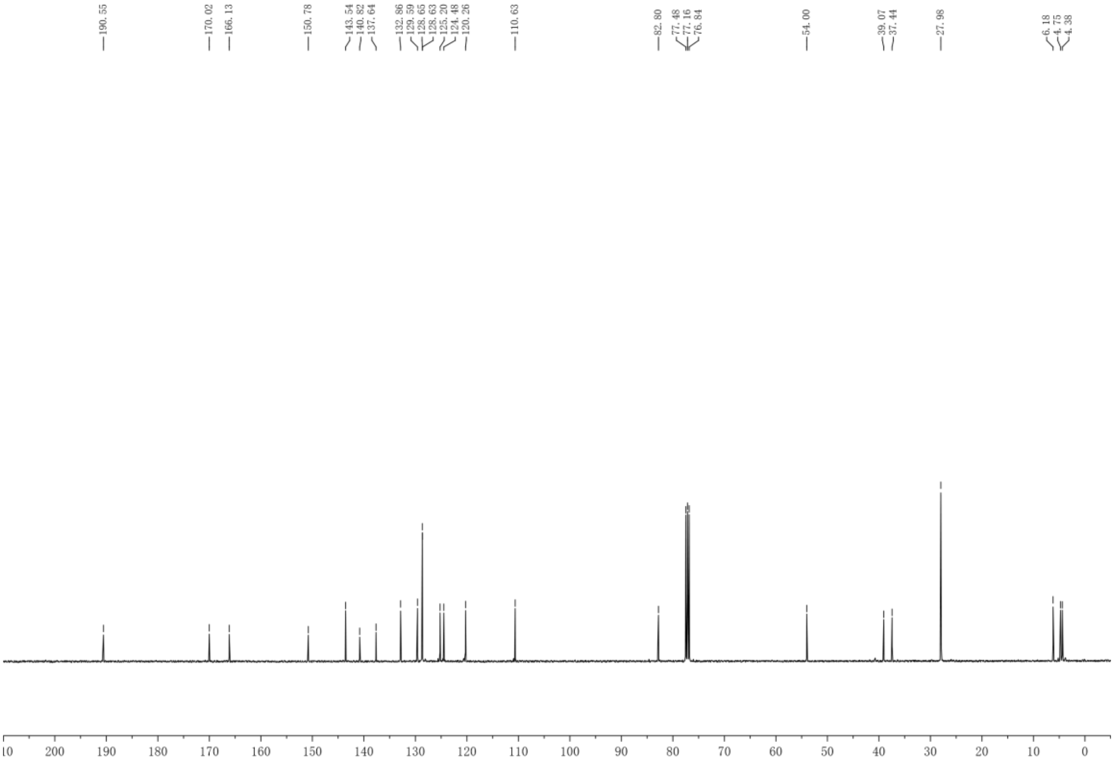

^{1}H NMR spectrum of **3x**

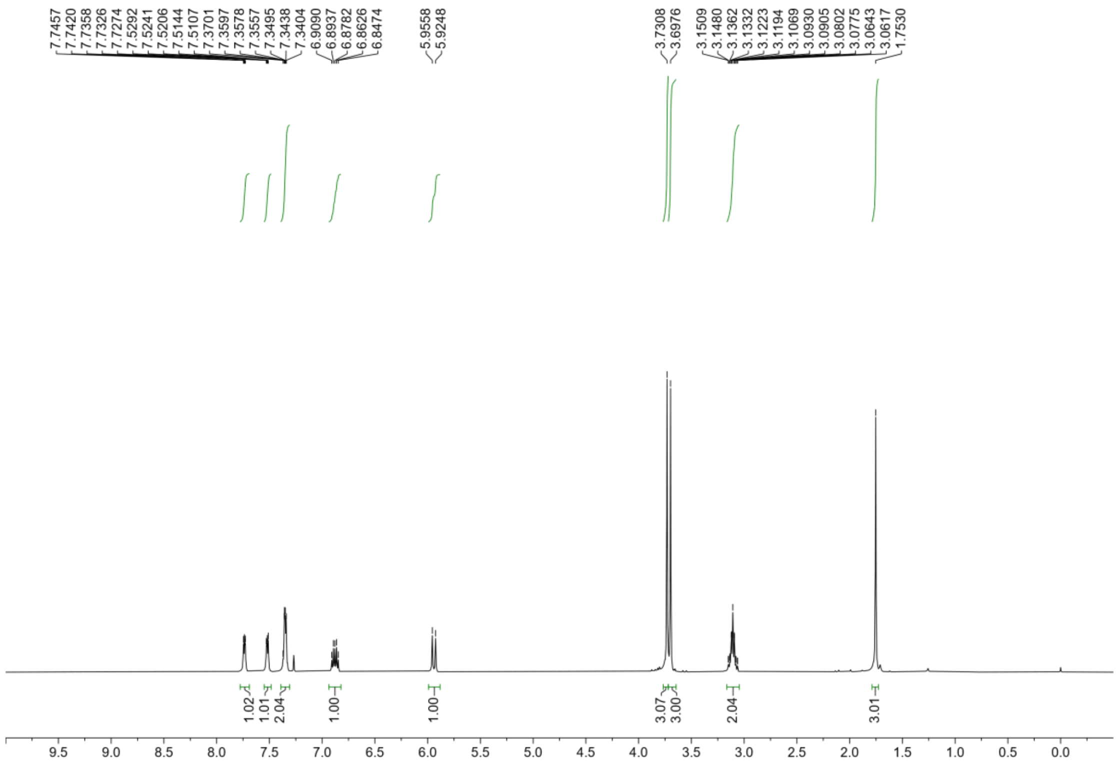

^{13}C NMR spectrum of **3x**

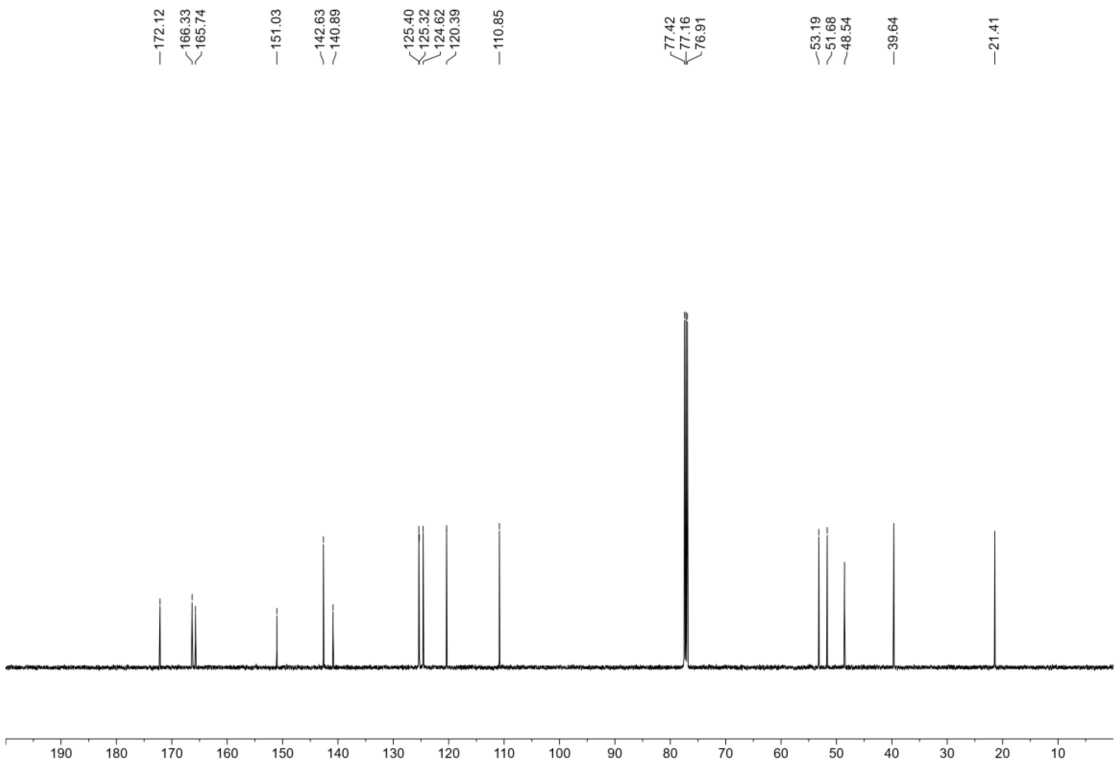

^{1}**H NMR** spectrum of **5a**

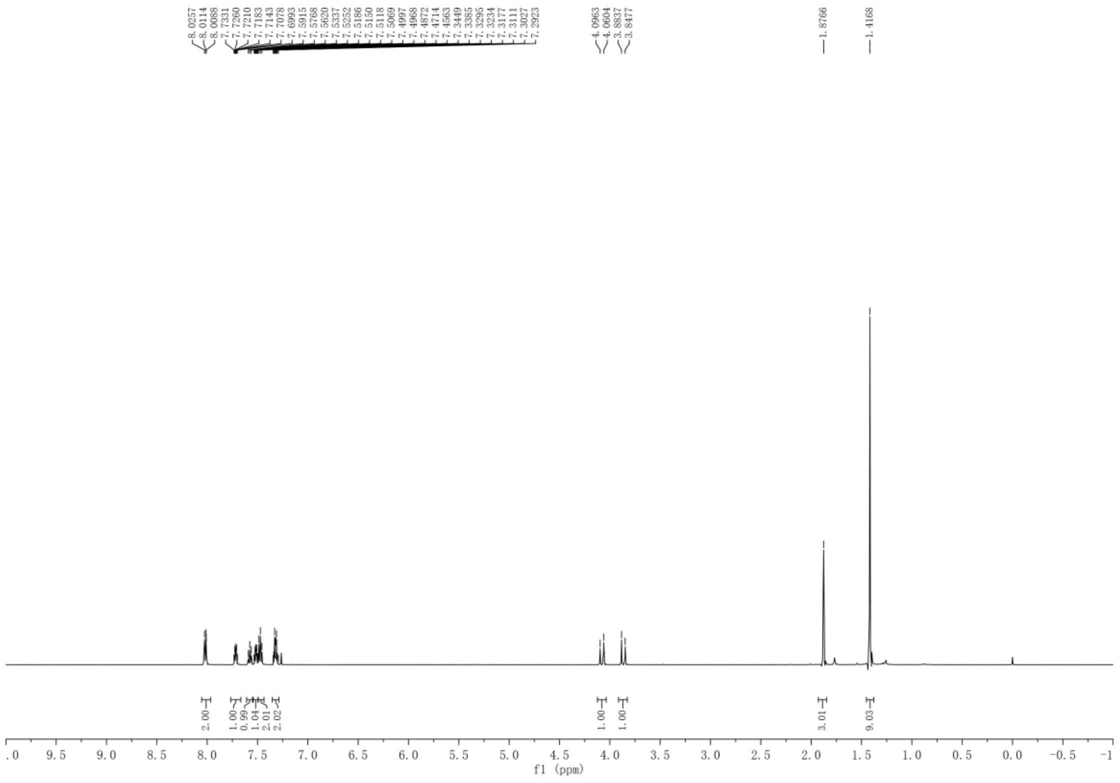

^{13}**C NMR** spectrum of **5a**

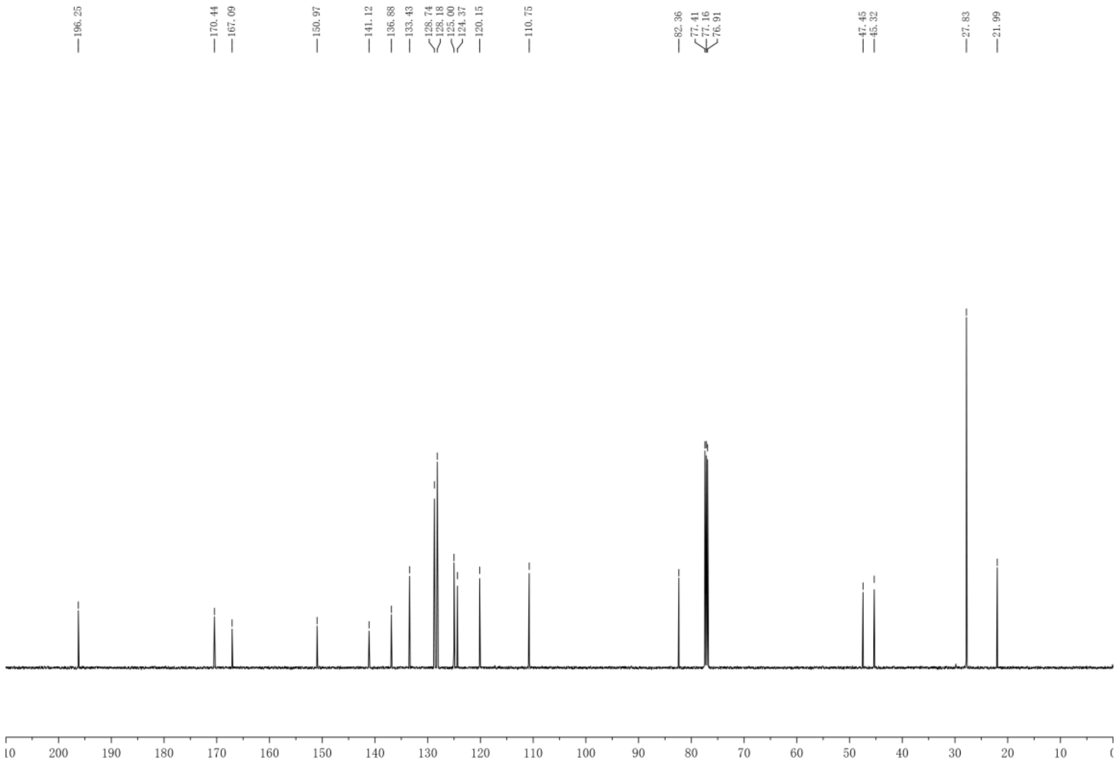

^{1}**H NMR** spectrum of **5b**

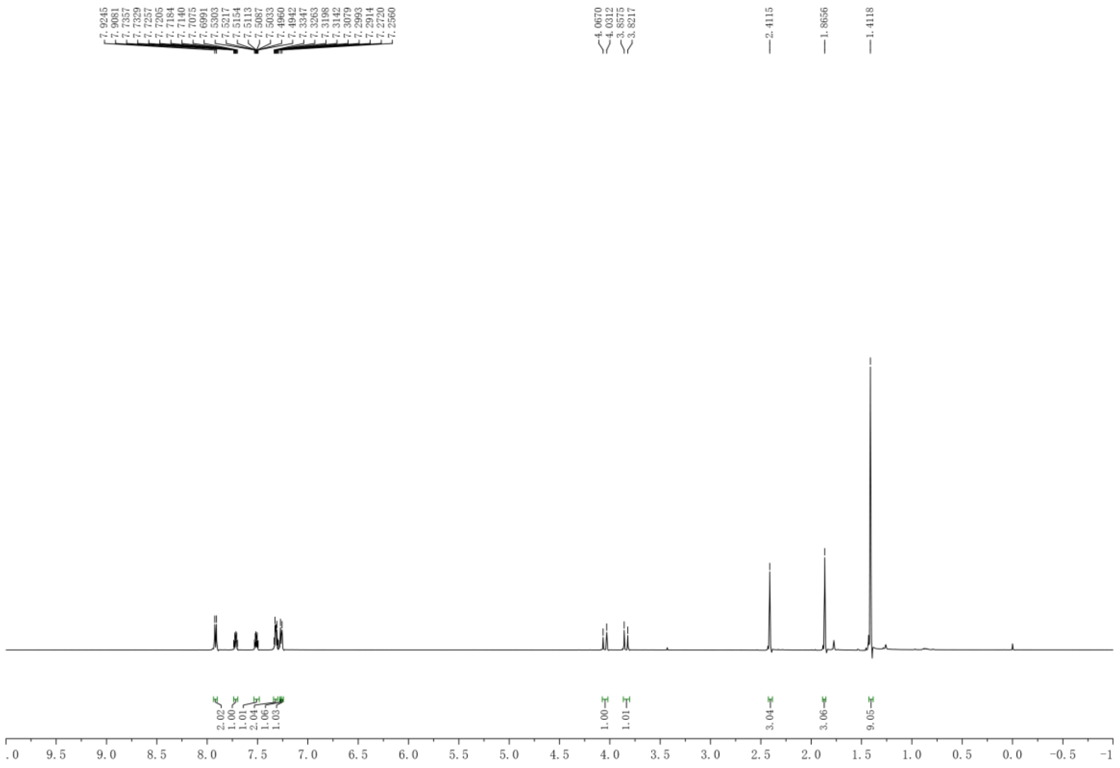

^{13}**C NMR** spectrum of **5b**

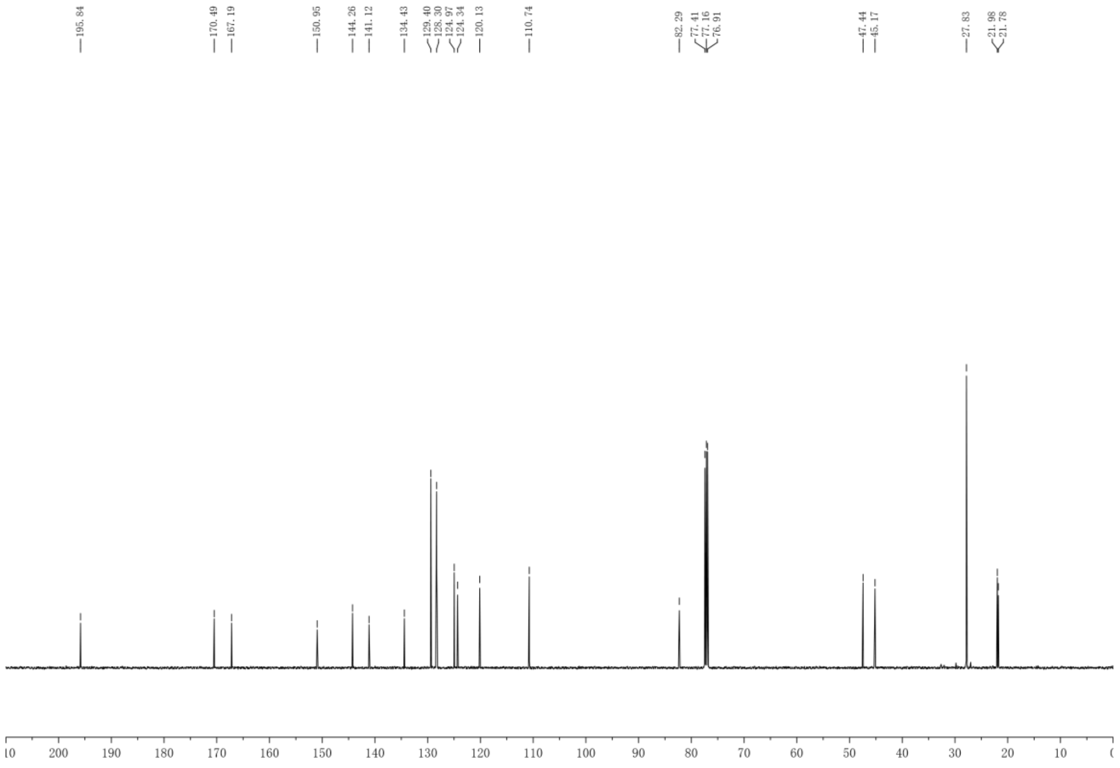

^{1}H NMR spectrum of **5c**

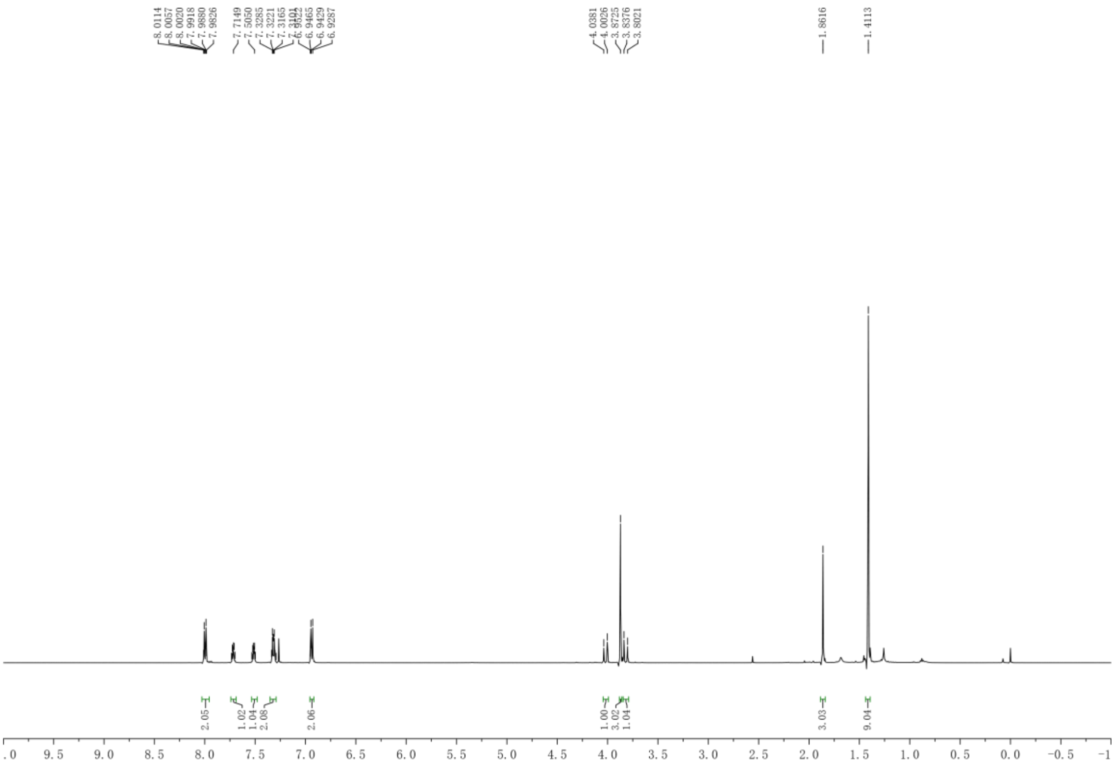

^{13}C NMR spectrum of **5c**

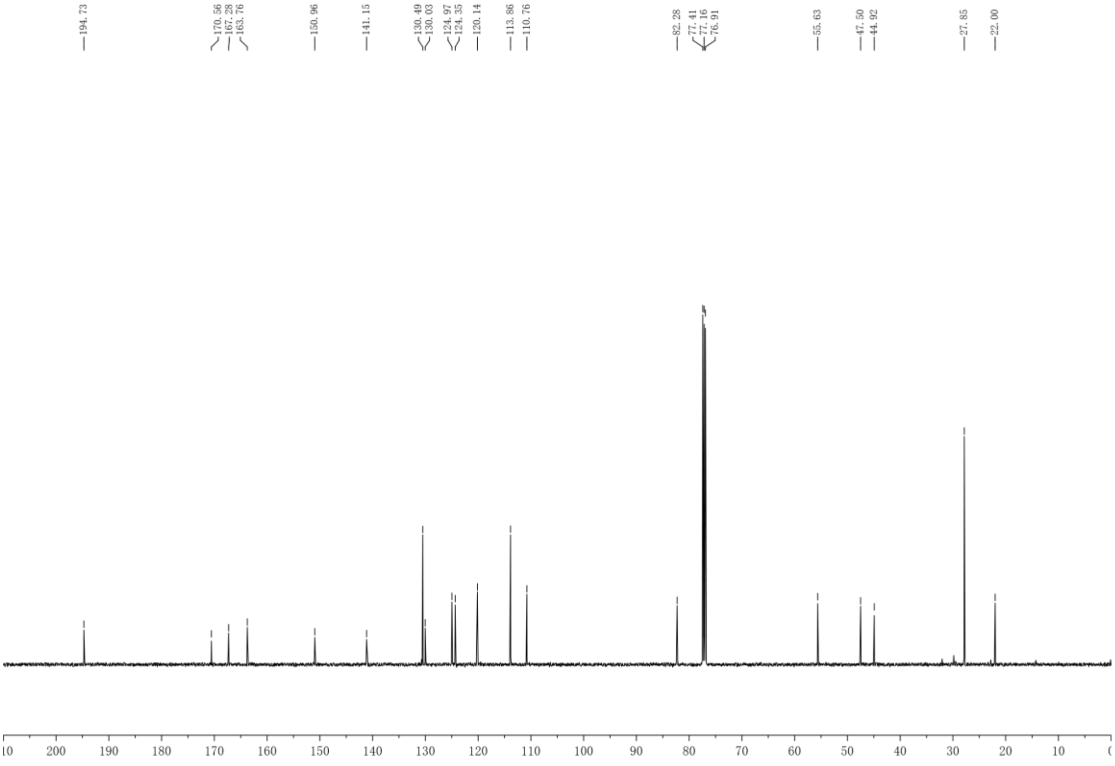

^{1}H NMR spectrum of **5d**

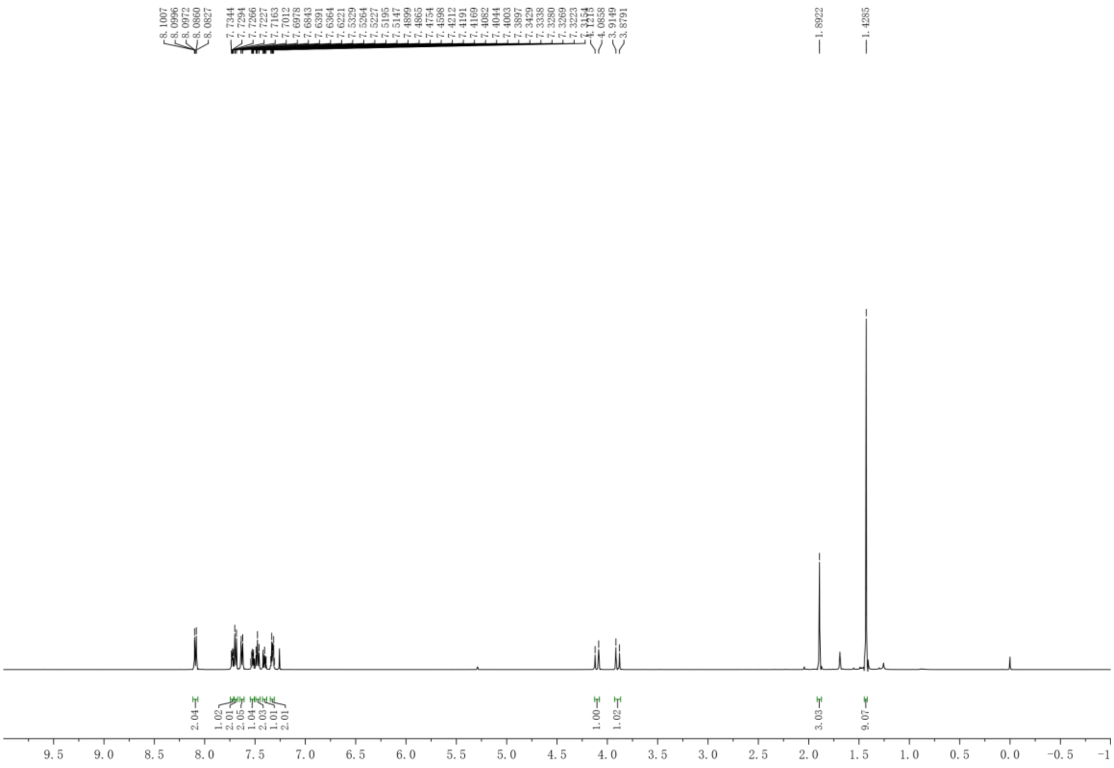

^{13}C NMR spectrum of **5d**

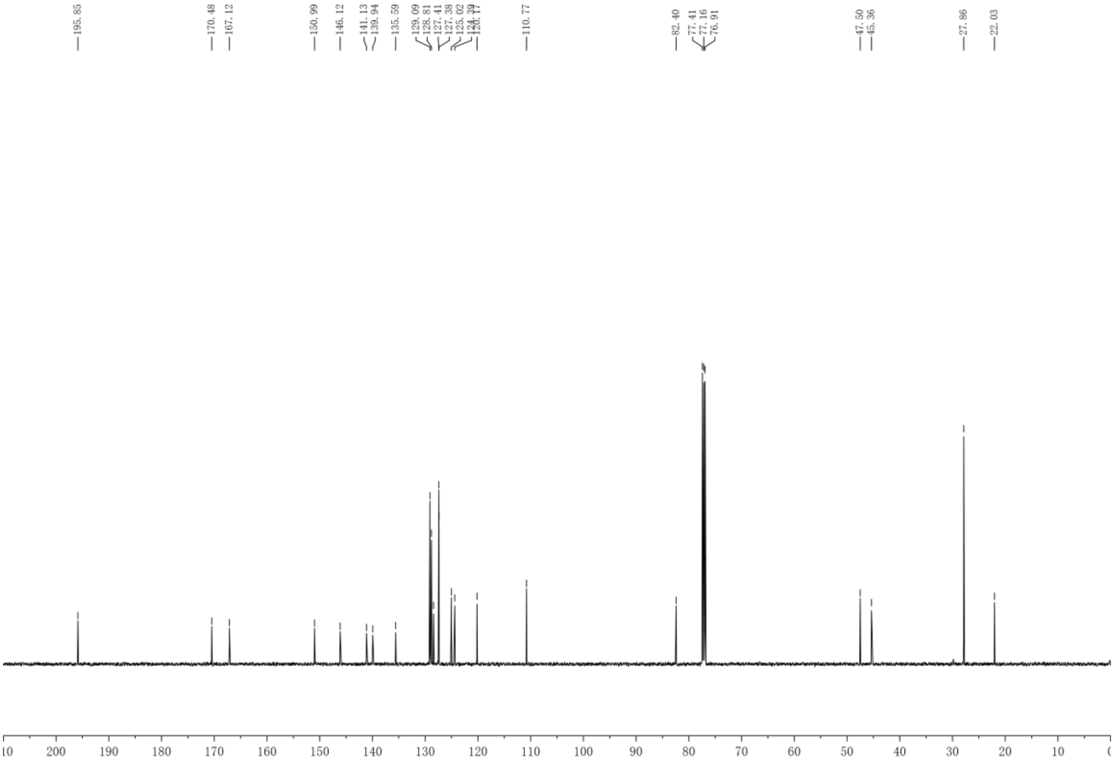

^{1}H NMR spectrum of **5e**

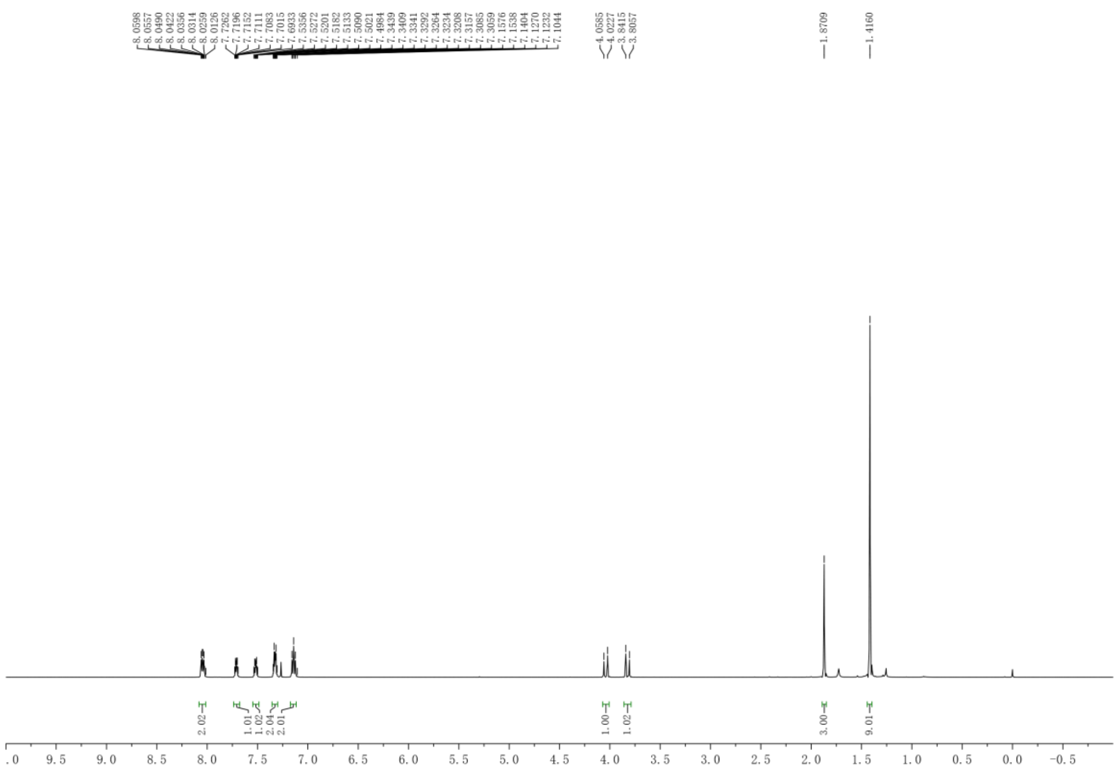

^{13}C NMR spectrum of **5e**

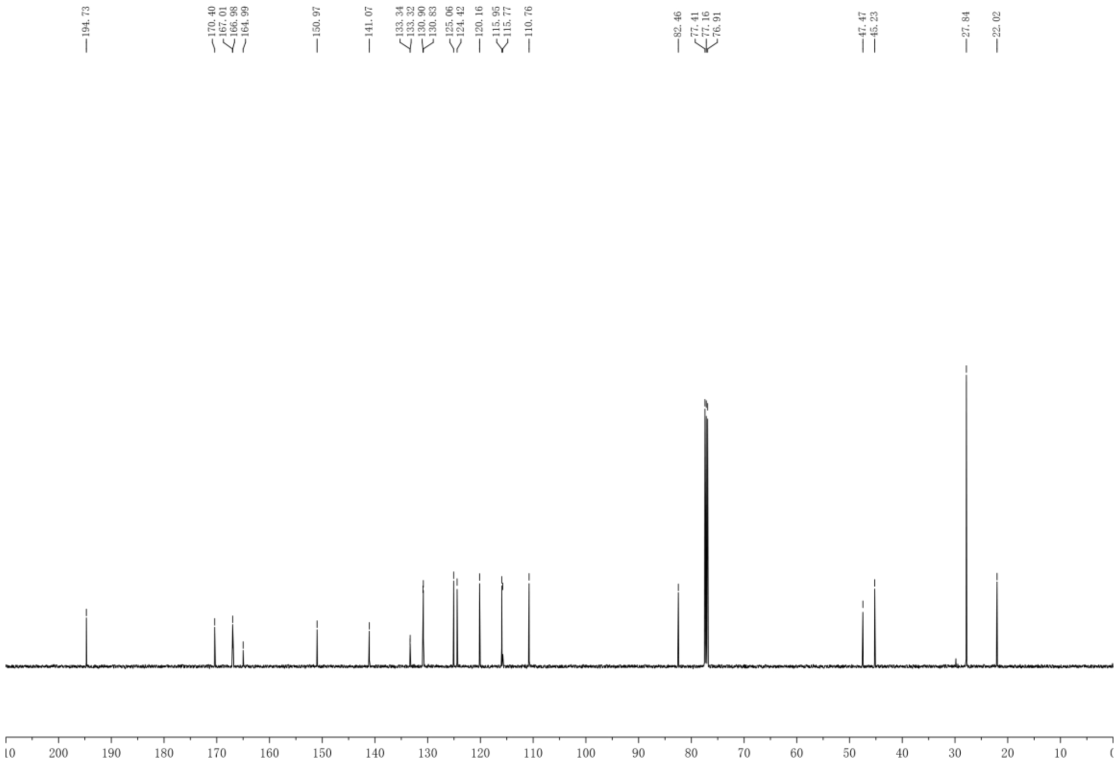

^{19}F NMR spectrum of **5e**

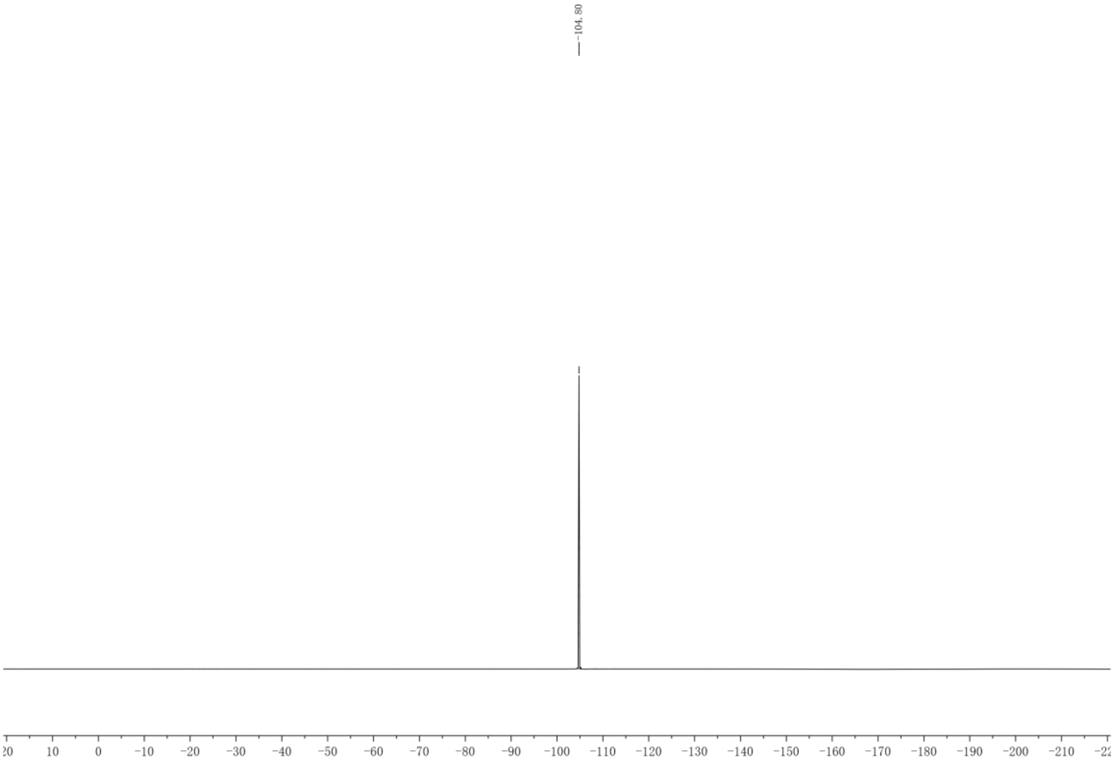

^{1}H NMR spectrum of **5f**

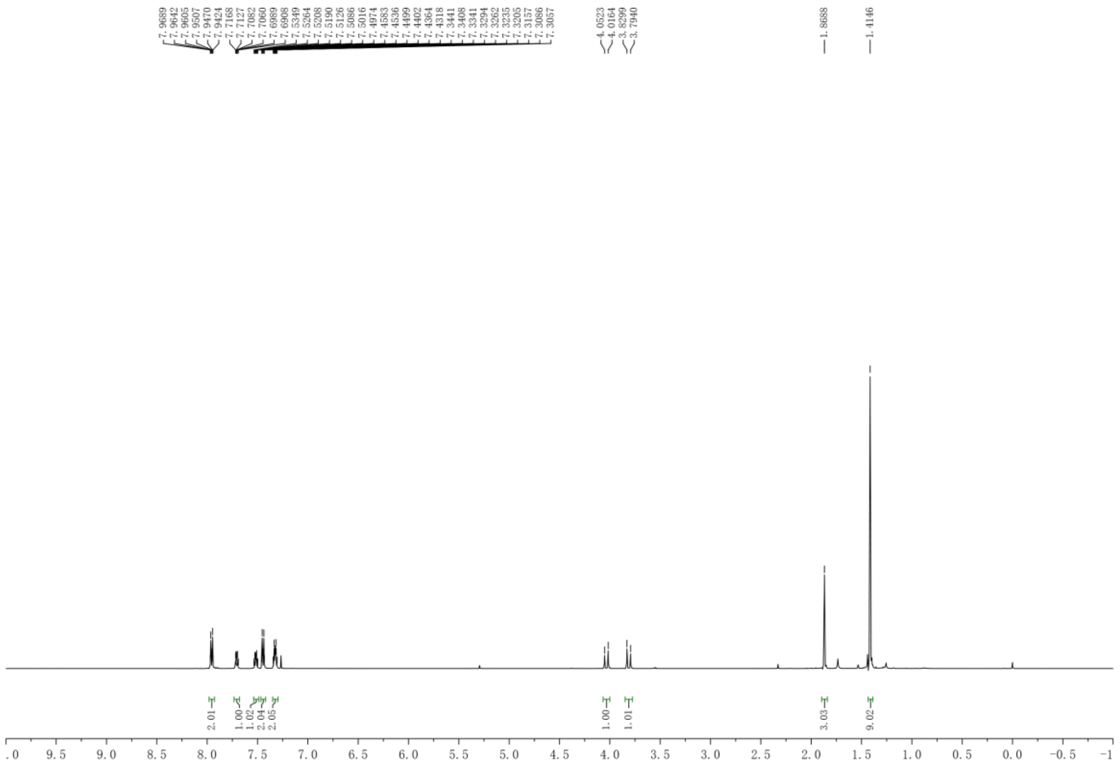

^{13}C NMR spectrum of **5f**

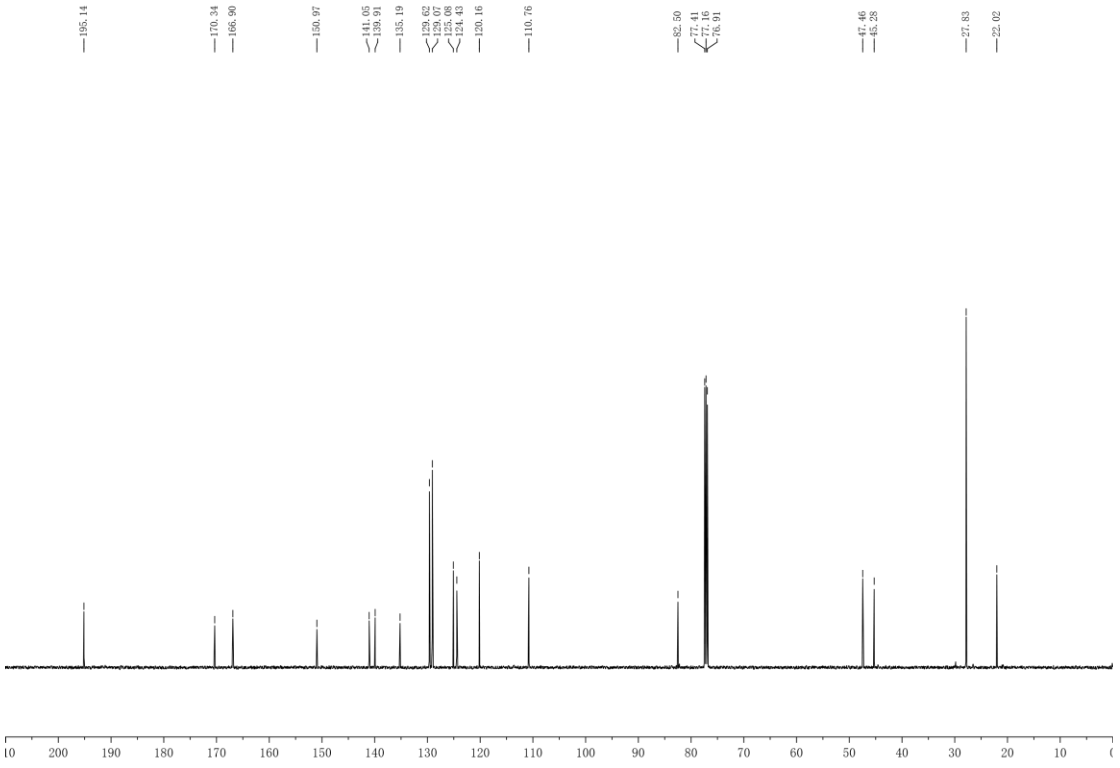

^{1}H NMR spectrum of **5g**

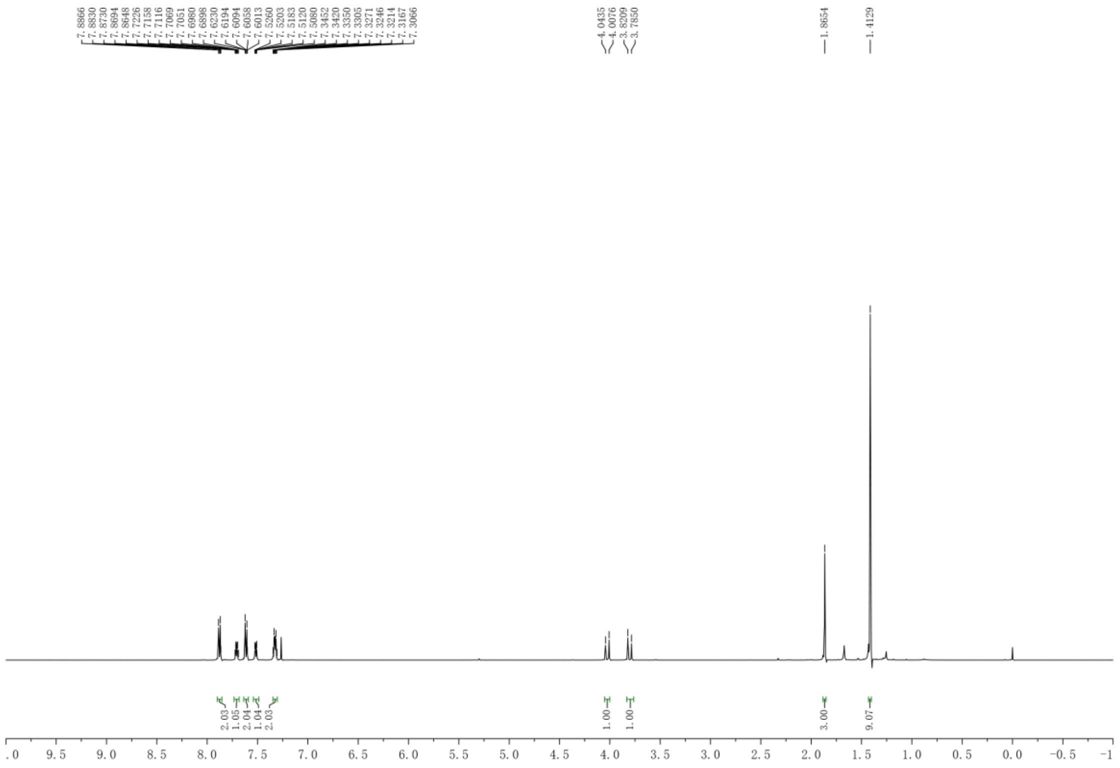

^{13}C NMR spectrum of **5g**

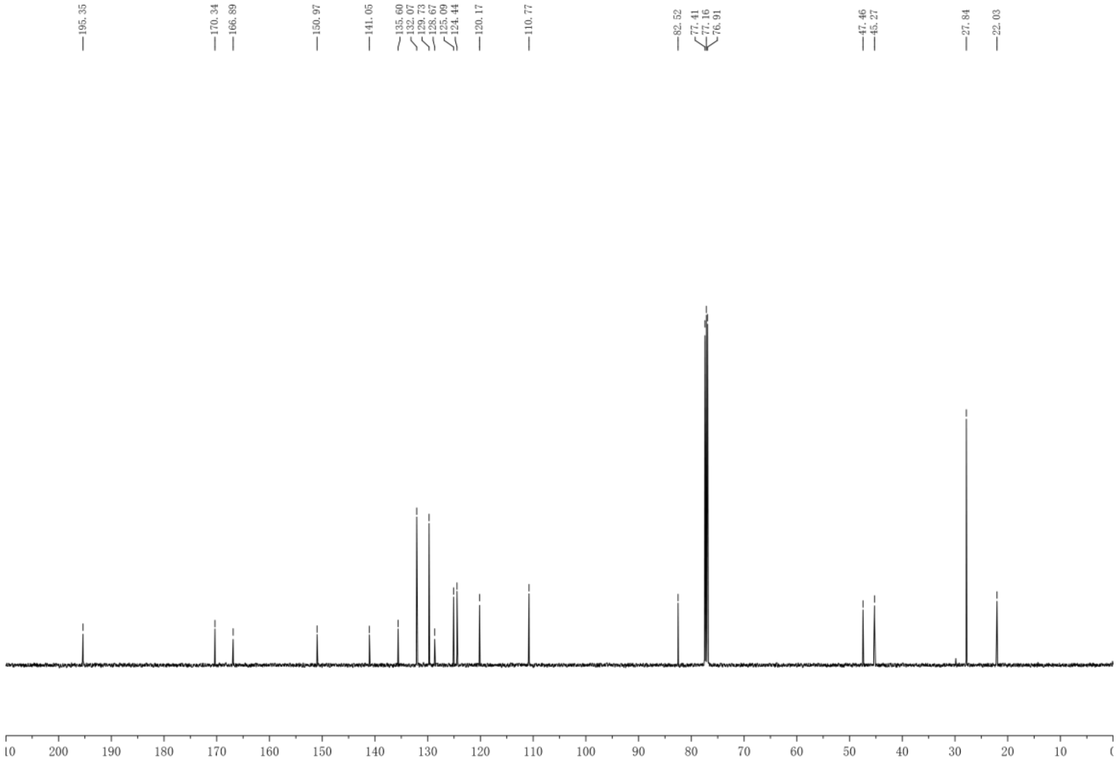

^{1}H NMR spectrum of **5h**

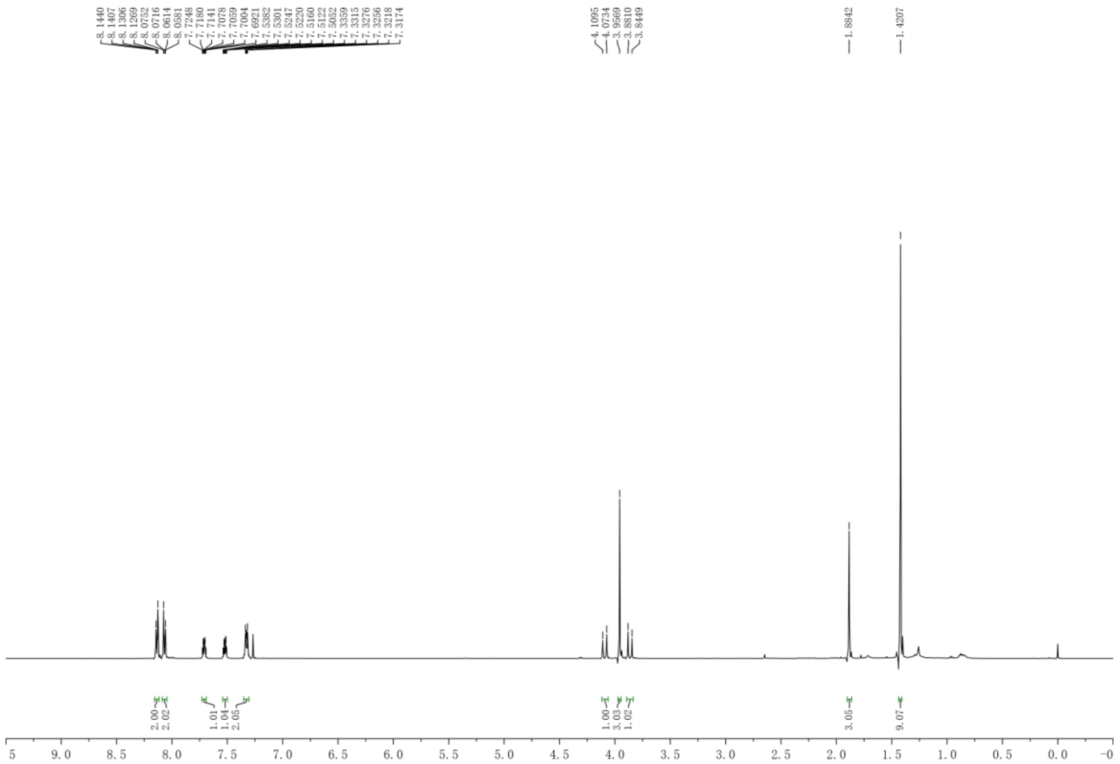

^{13}C NMR spectrum of **5h**

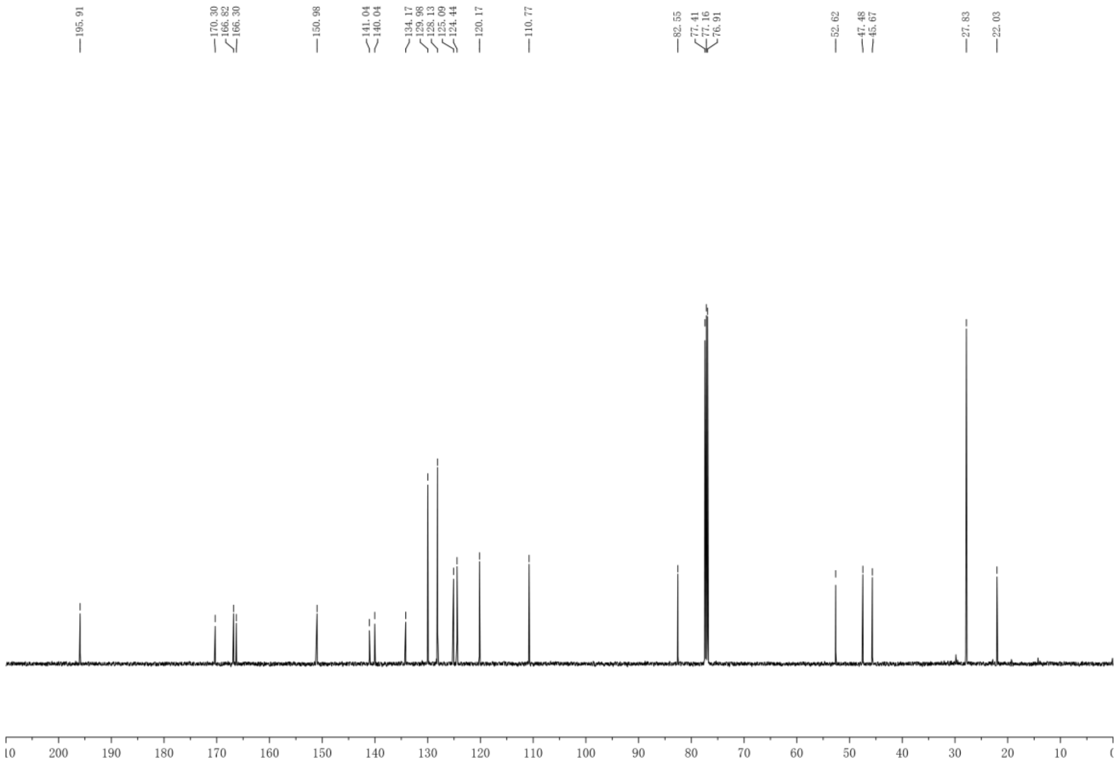

^{1}H NMR spectrum of **5i**

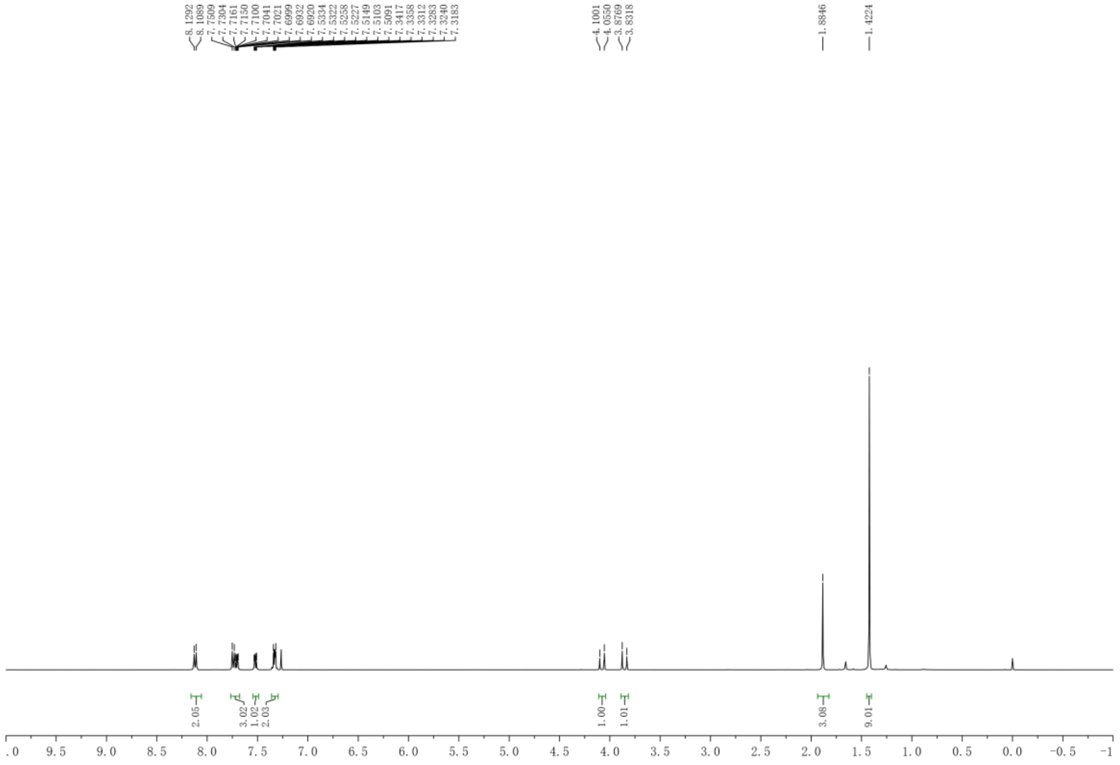

^{13}C NMR spectrum of **5i**

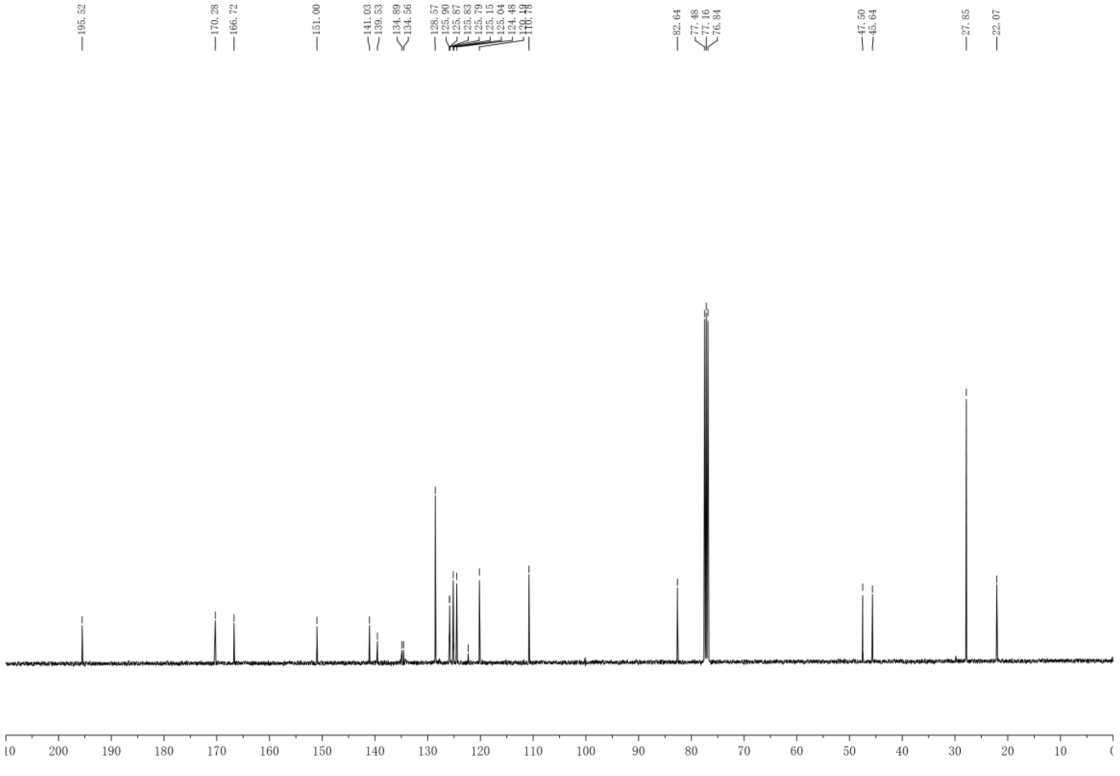

^{19}F NMR spectrum of **5i**

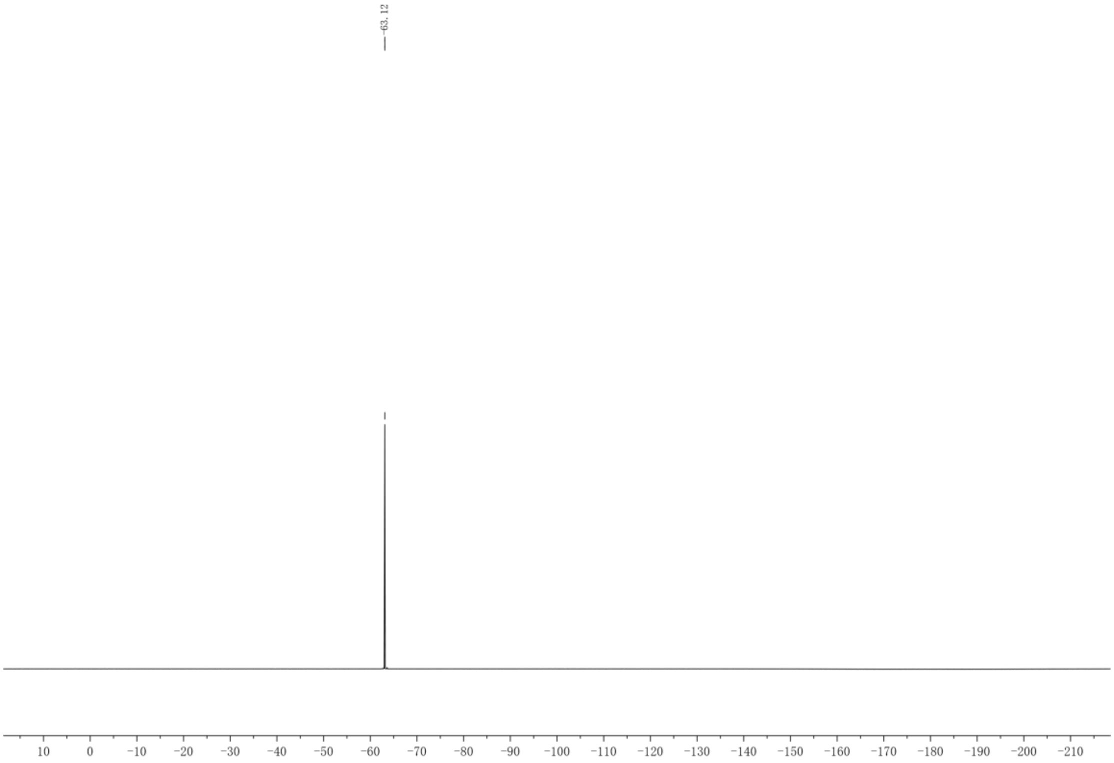

^{1}H NMR spectrum of **5j**

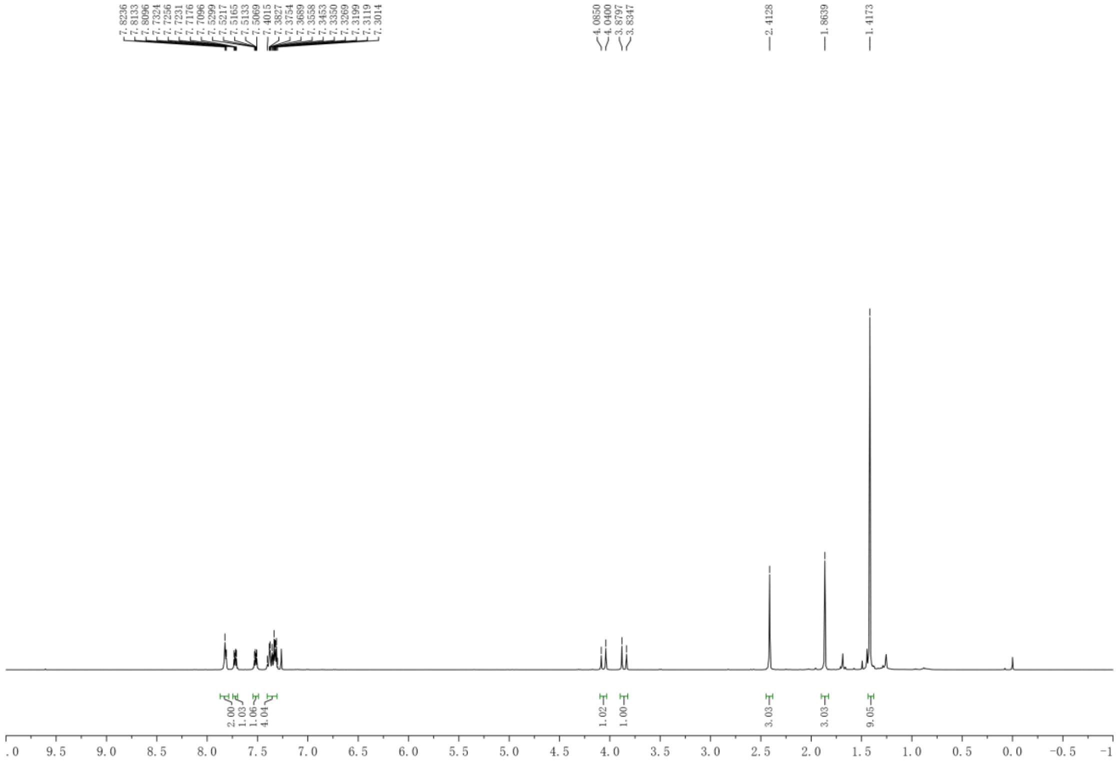

^{13}C NMR spectrum of **5j**

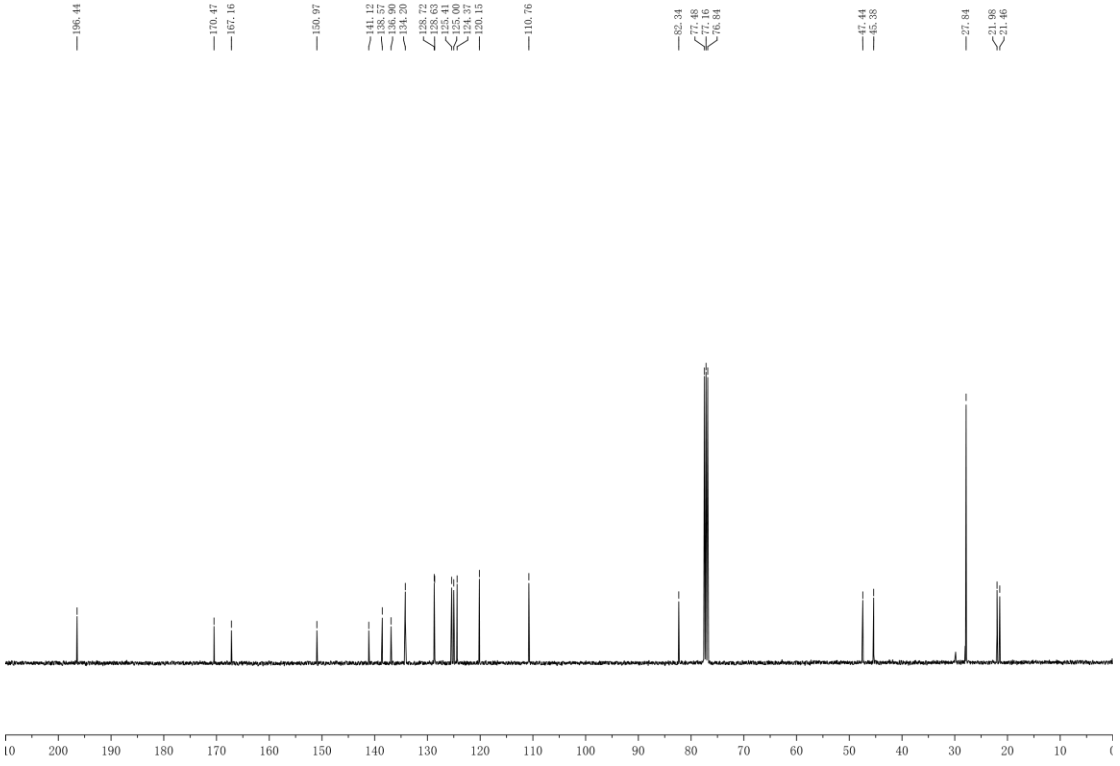

^{1}H NMR spectrum of **5k**

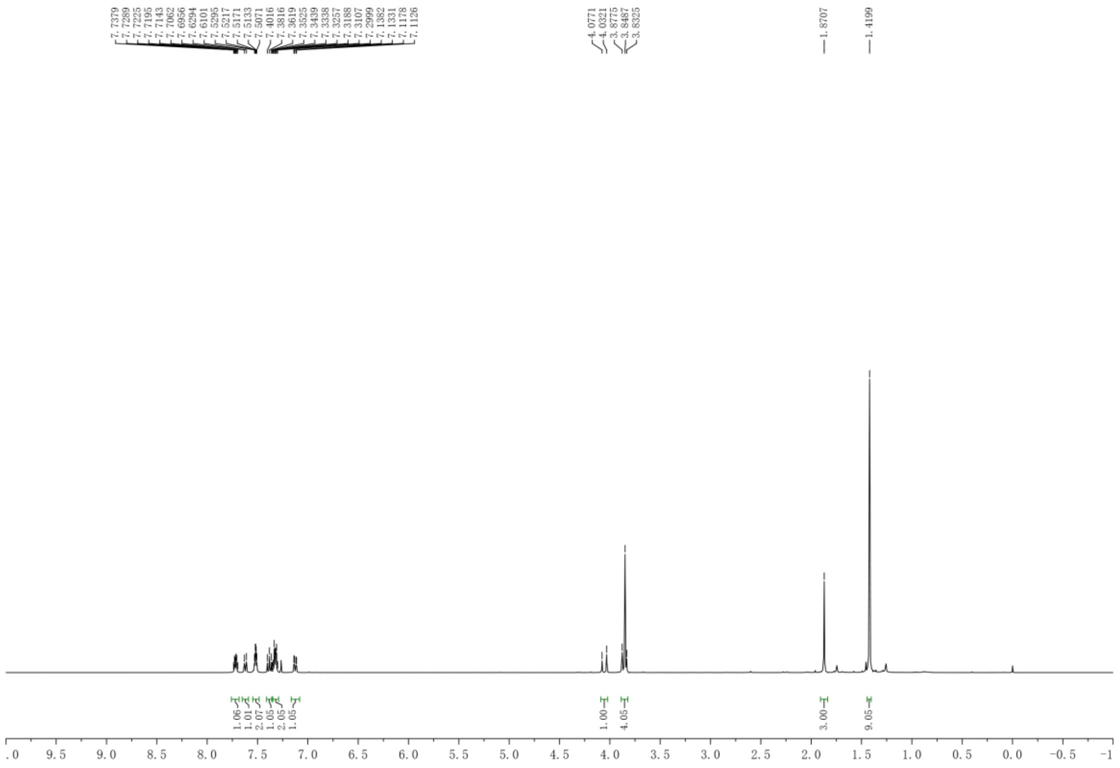

^{13}C NMR spectrum of **5k**

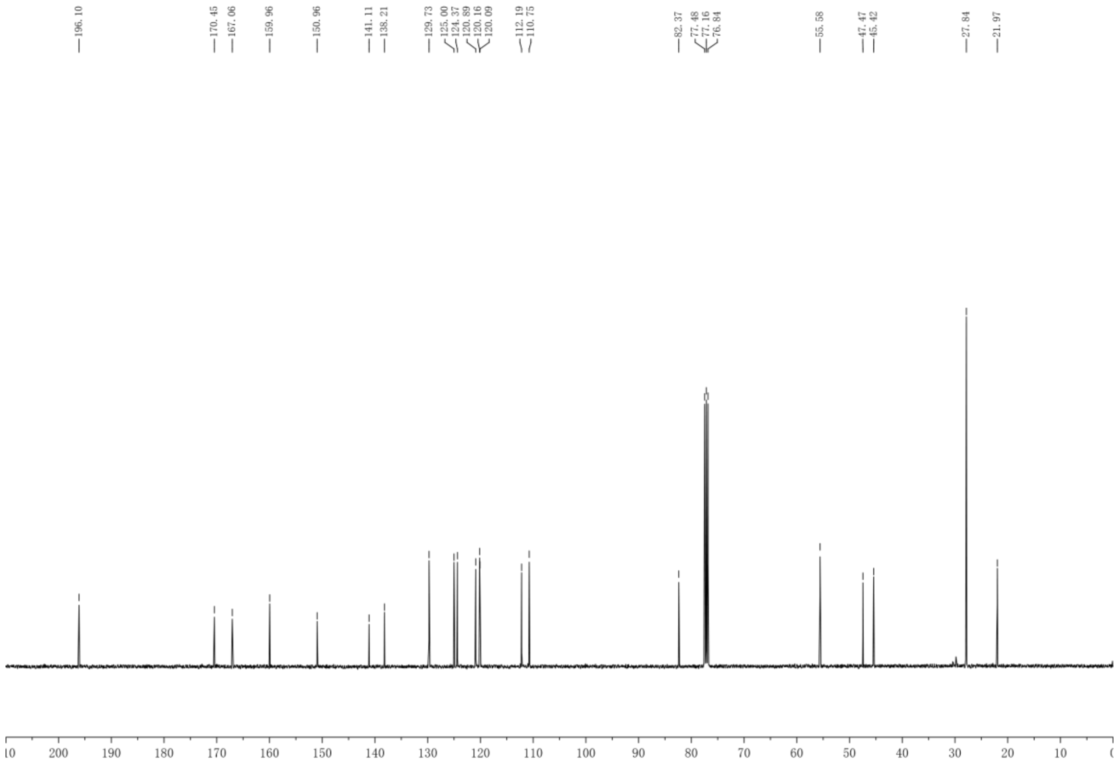

^{1}H NMR spectrum of **5l**

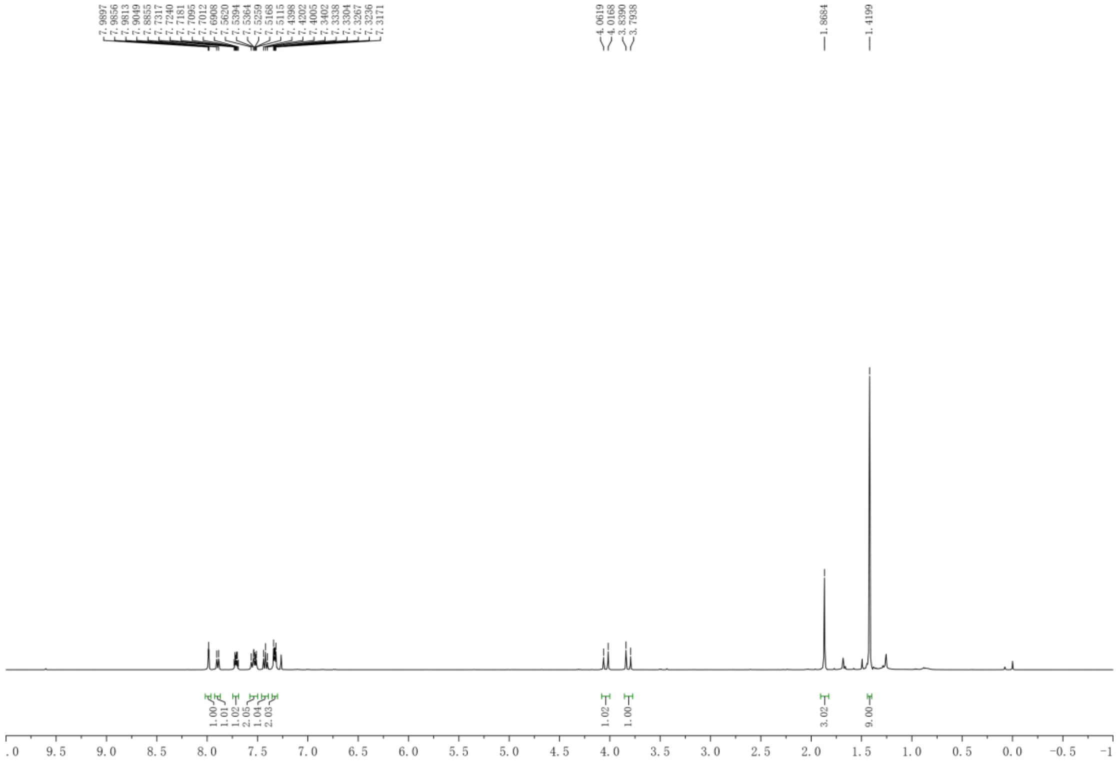

^{13}C NMR spectrum of **5l**

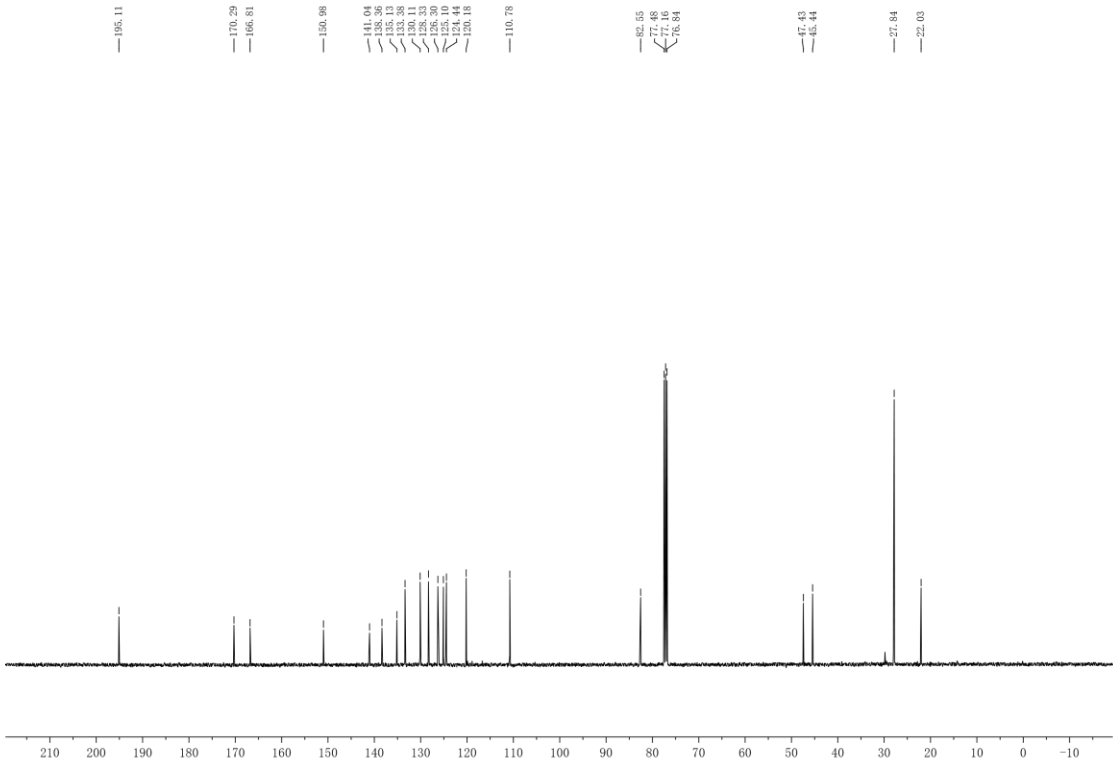

^{1}H NMR spectrum of **5m**

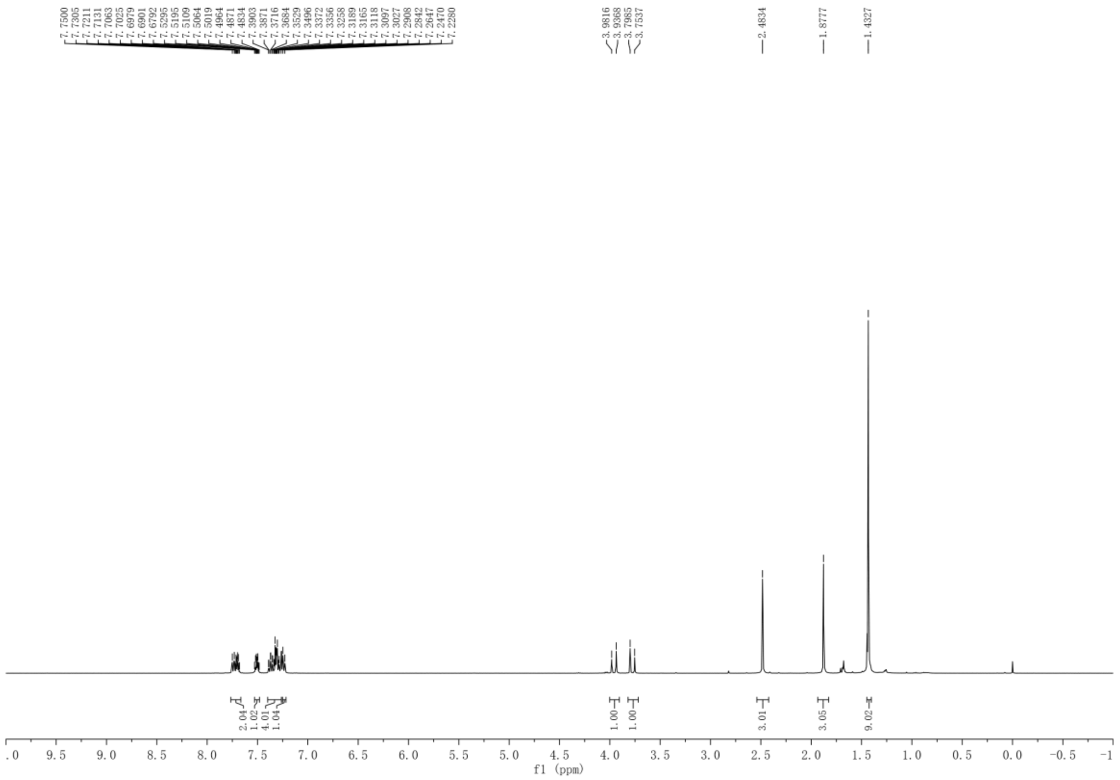

^{13}C NMR spectrum of **5m**

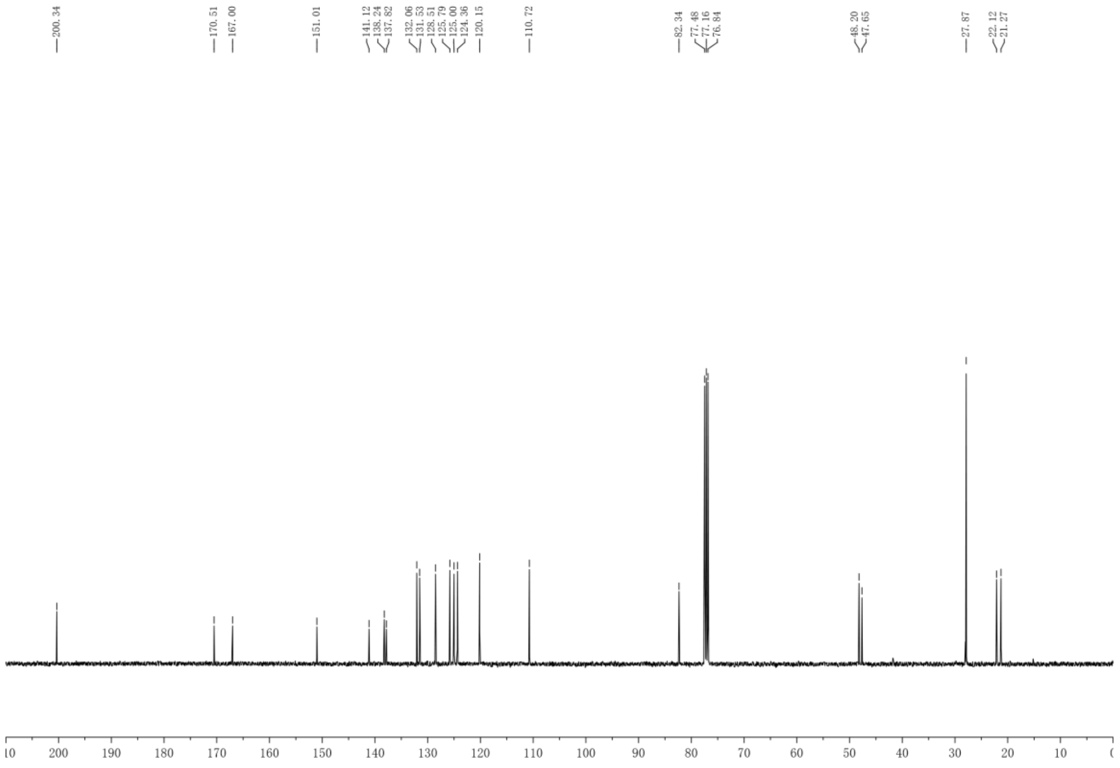

^{1}H NMR spectrum of **5n**

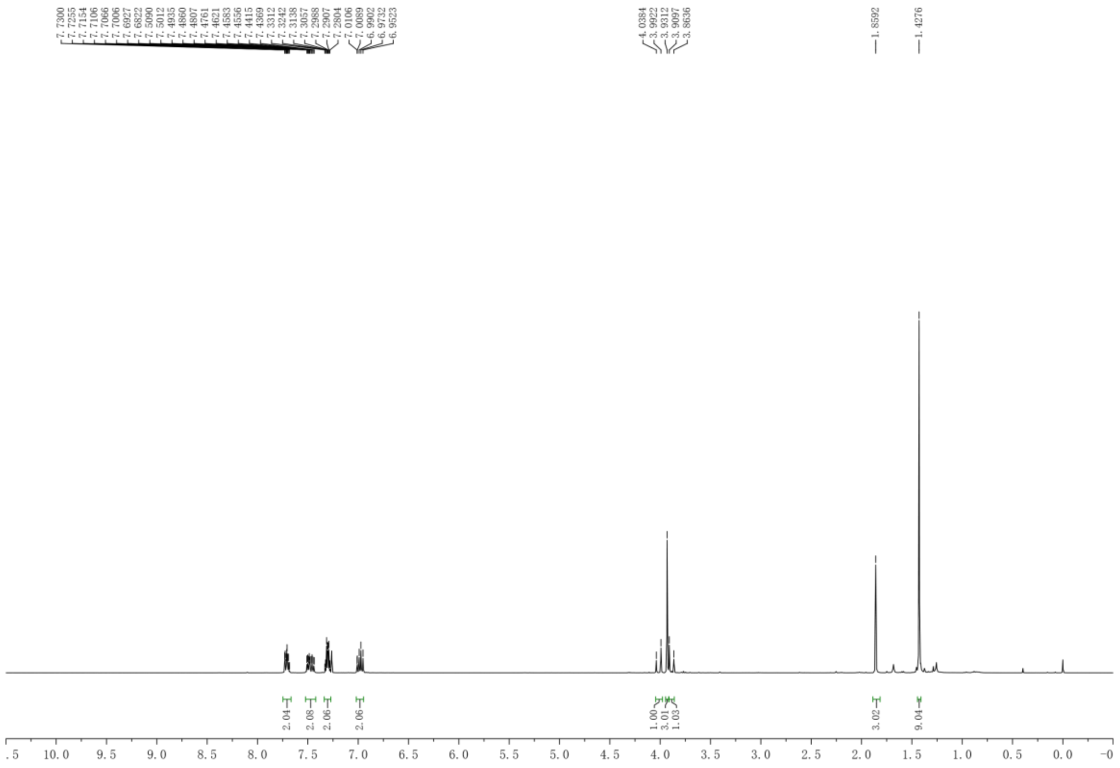

^{13}C NMR spectrum of **5n**

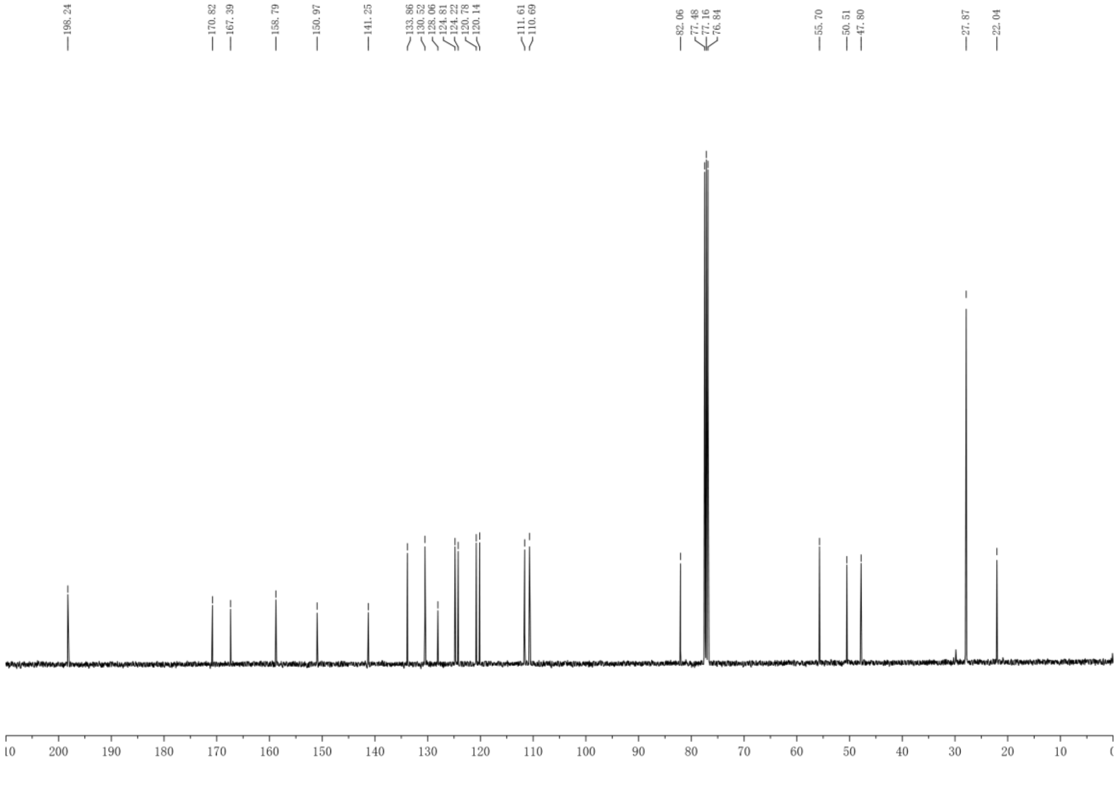

^{1}H NMR spectrum of **5o**

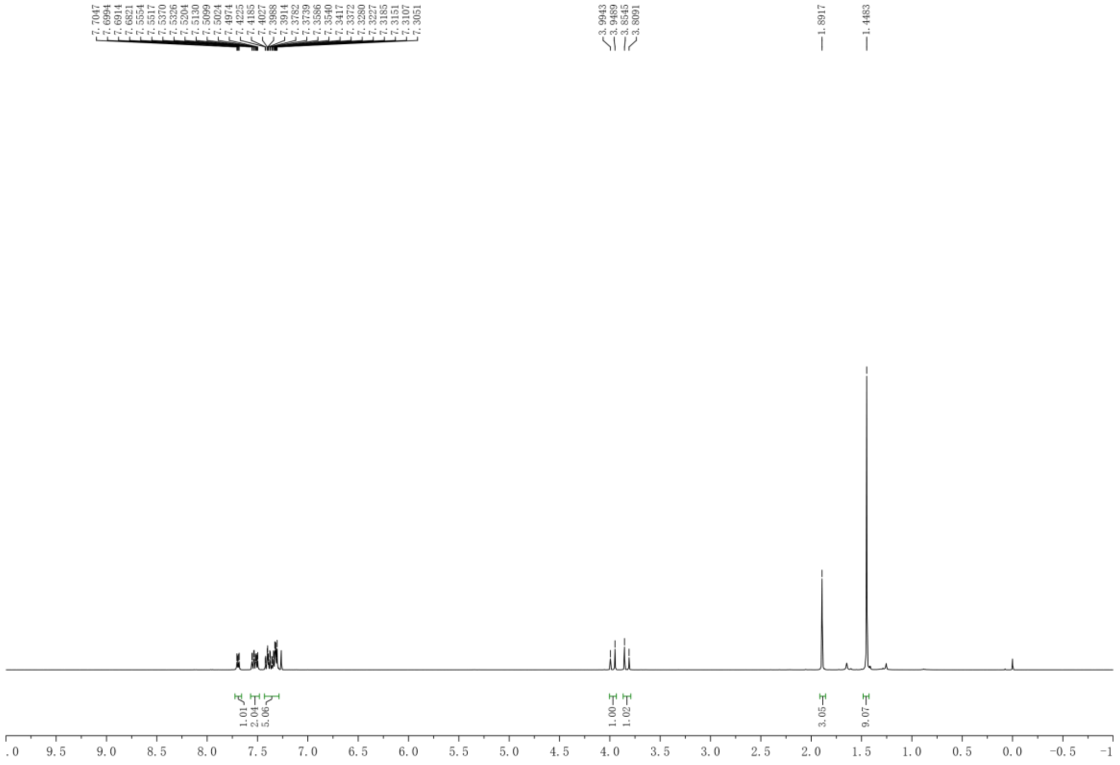

^{13}C NMR spectrum of **5o**

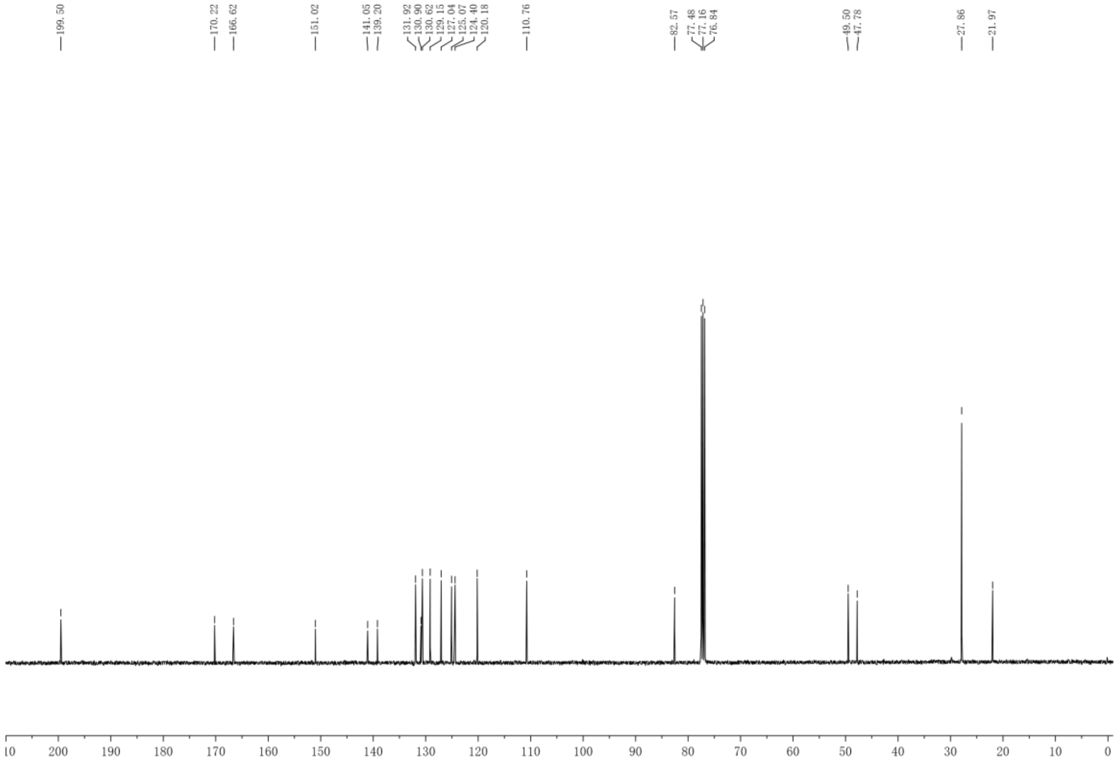

^{1}H NMR spectrum of **5p**

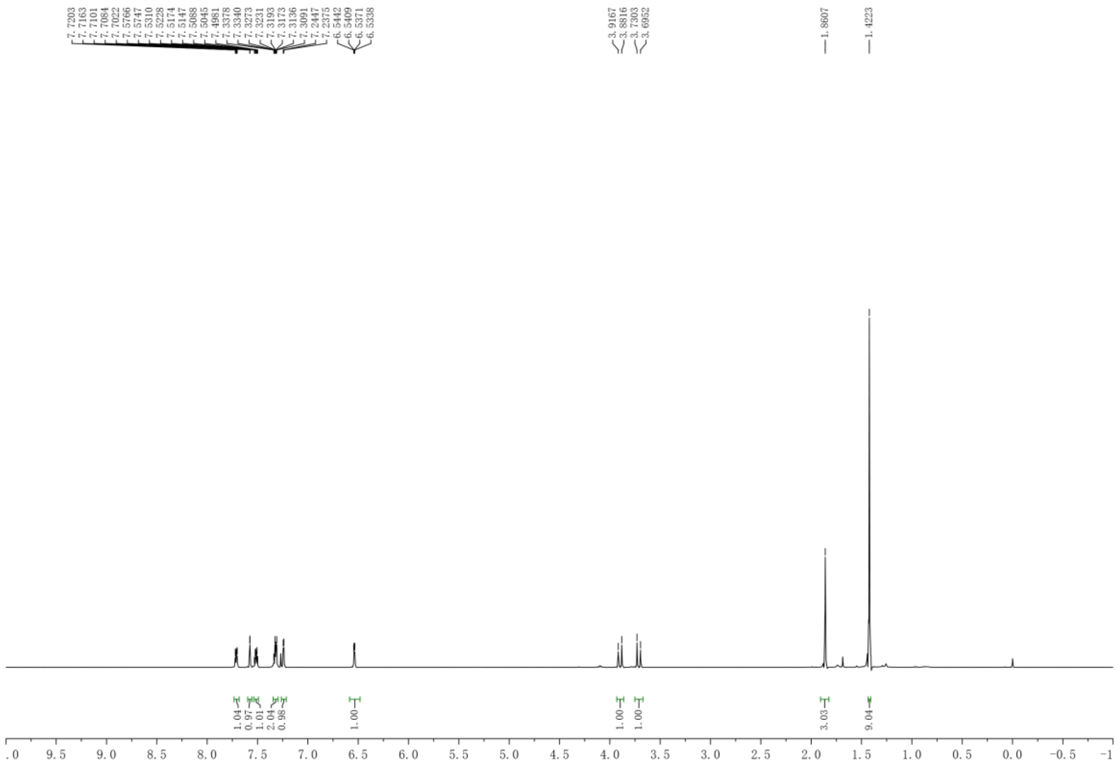

^{13}C NMR spectrum of **5p**

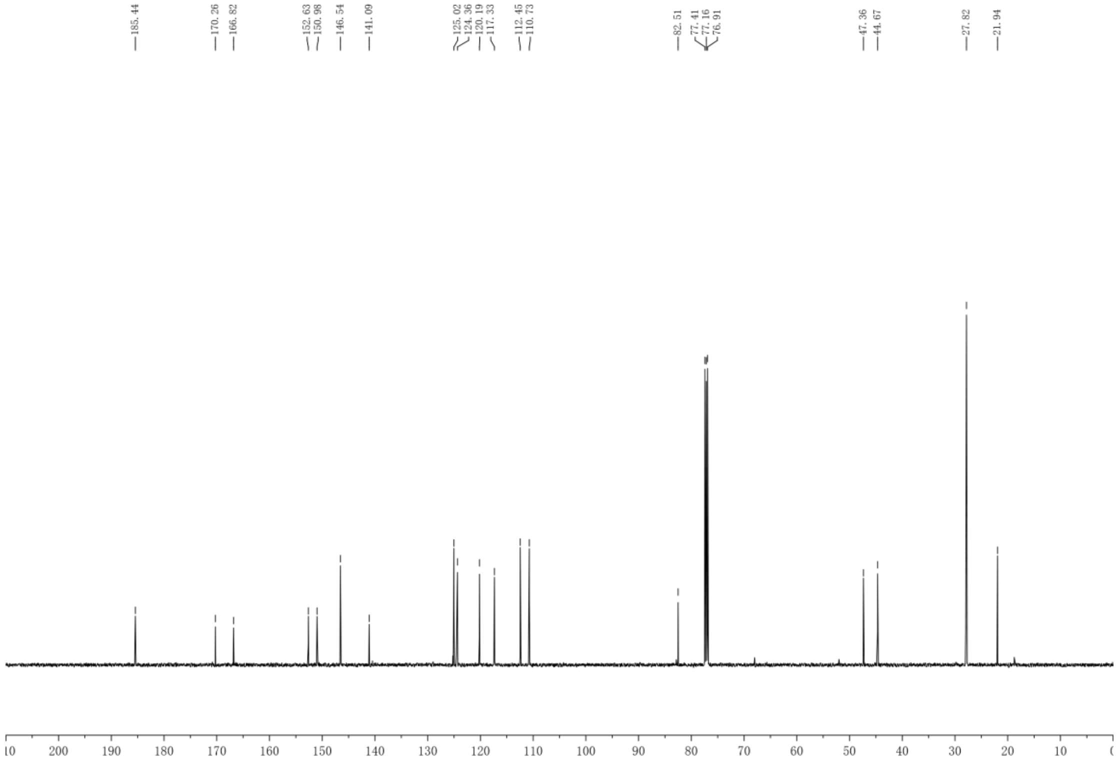

^{1}H NMR spectrum of **5q**

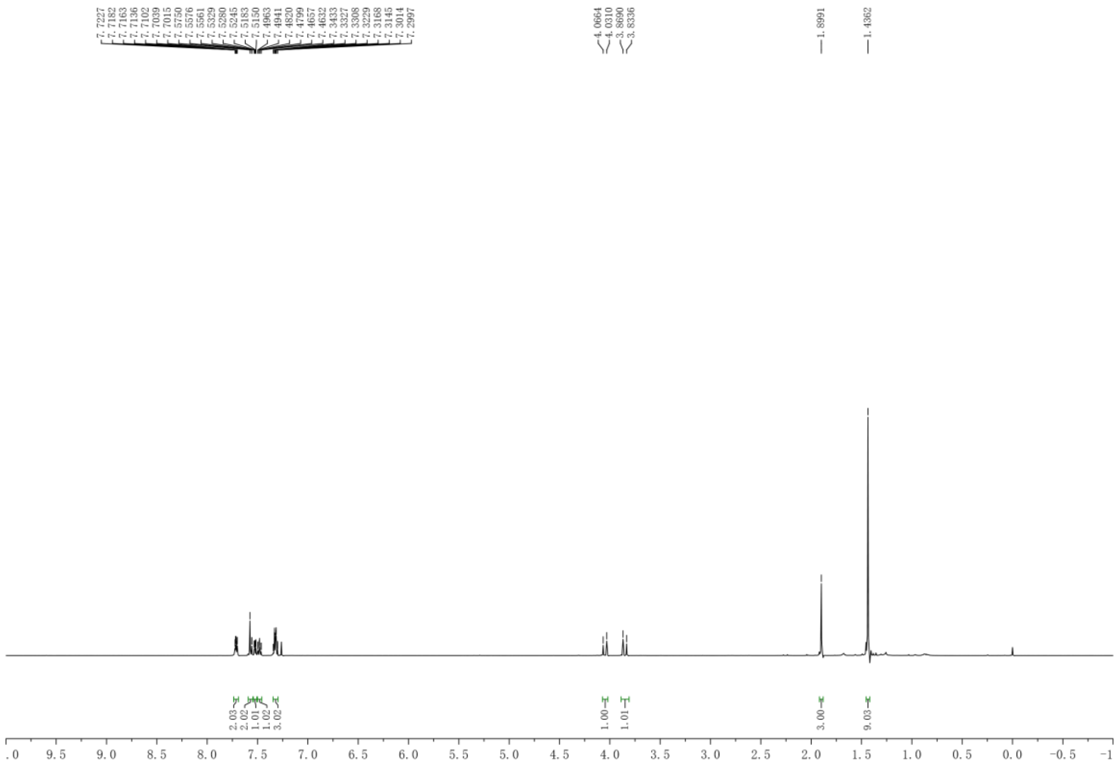

^{13}C NMR spectrum of **5q**

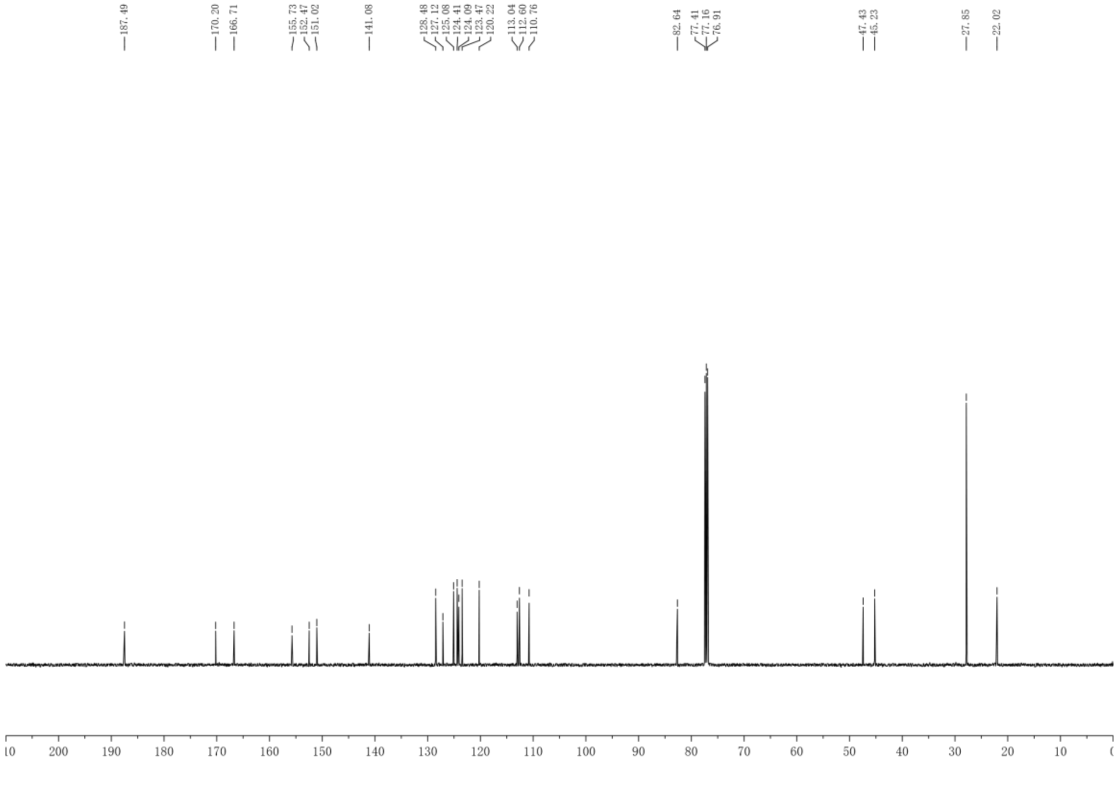

^{1}H NMR spectrum of **5r**

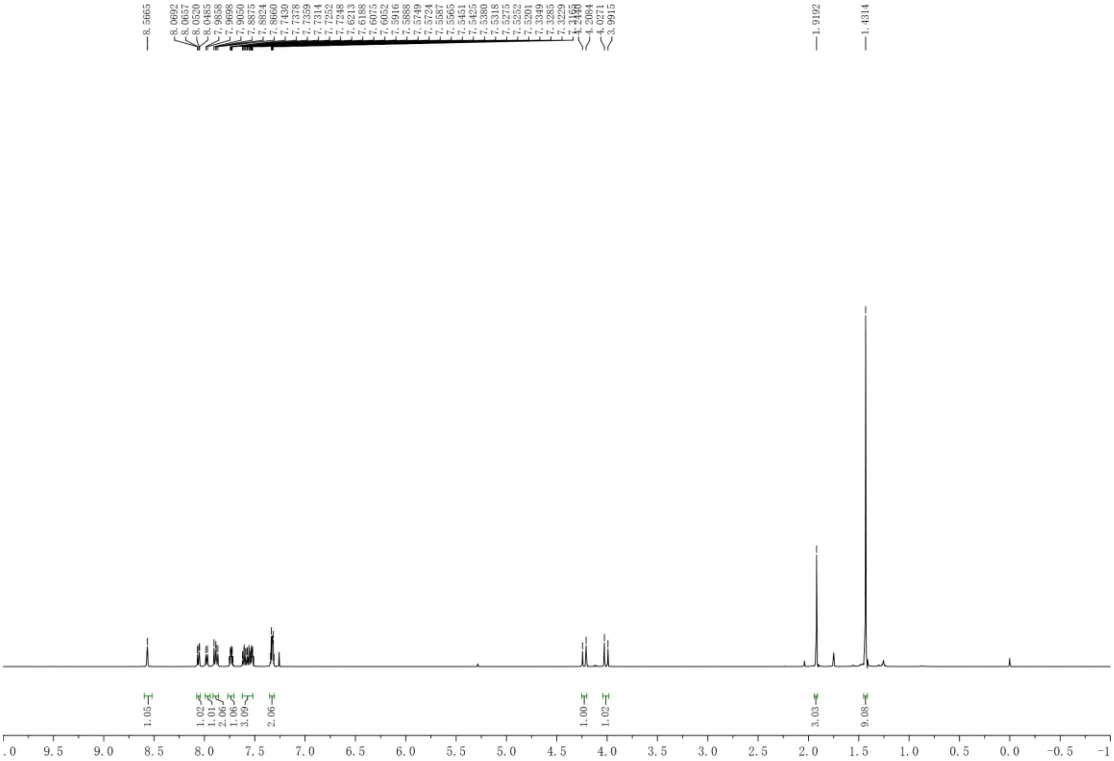

^{13}C NMR spectrum of **5r**

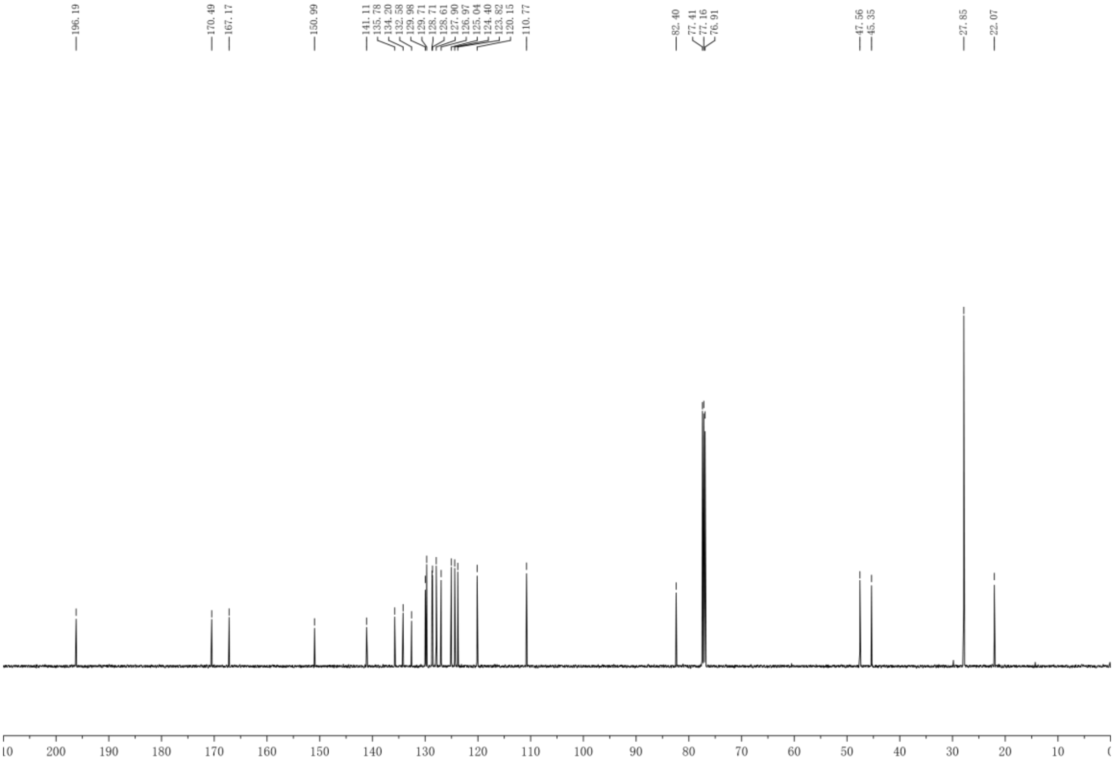

^{1}H NMR spectrum of **5s**

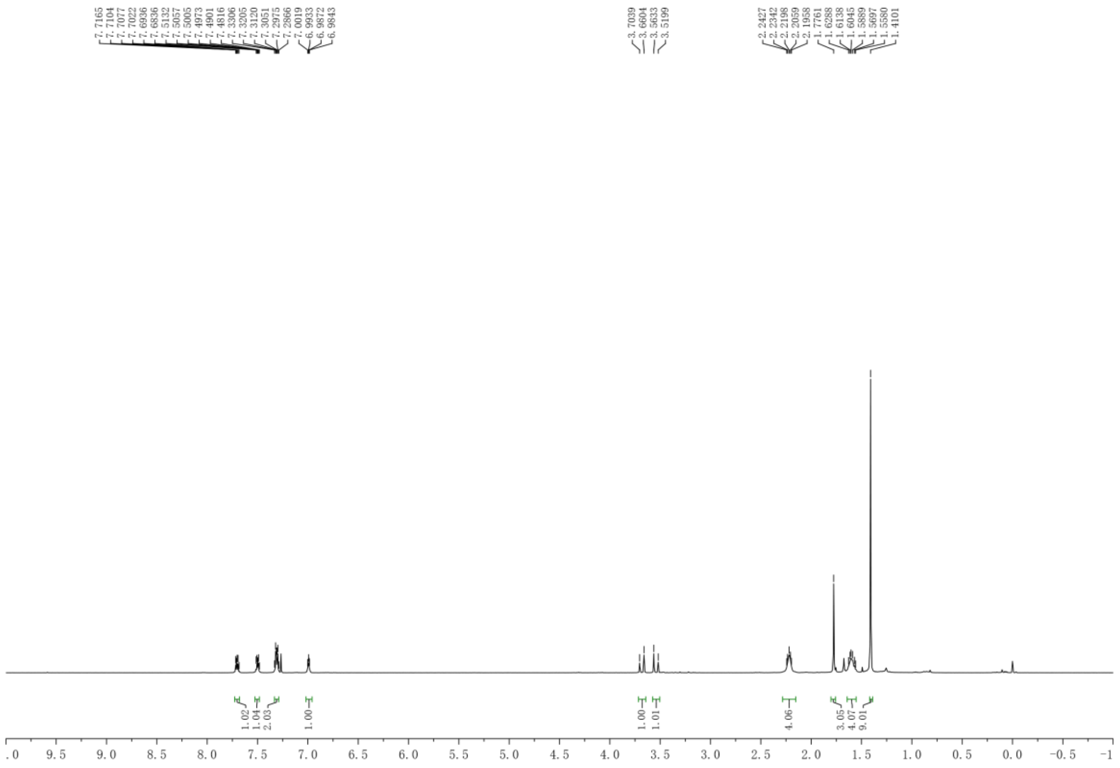

^{13}C NMR spectrum of **5s**

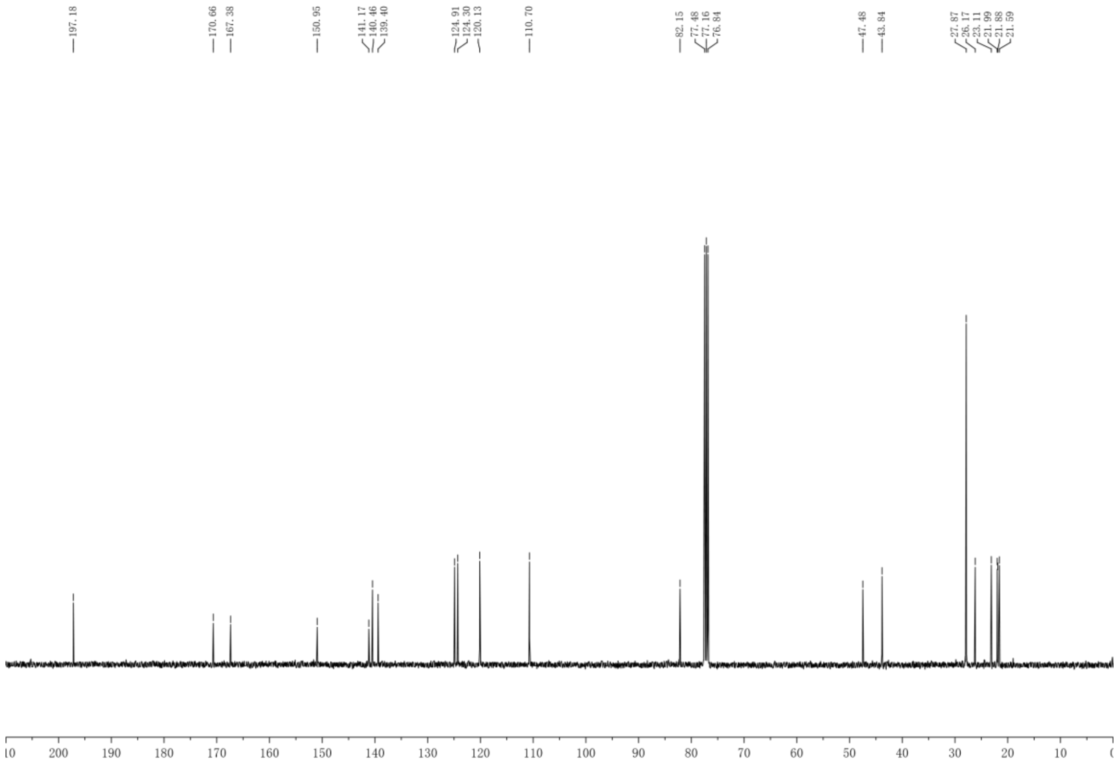

^{1}H NMR spectrum of **5t**

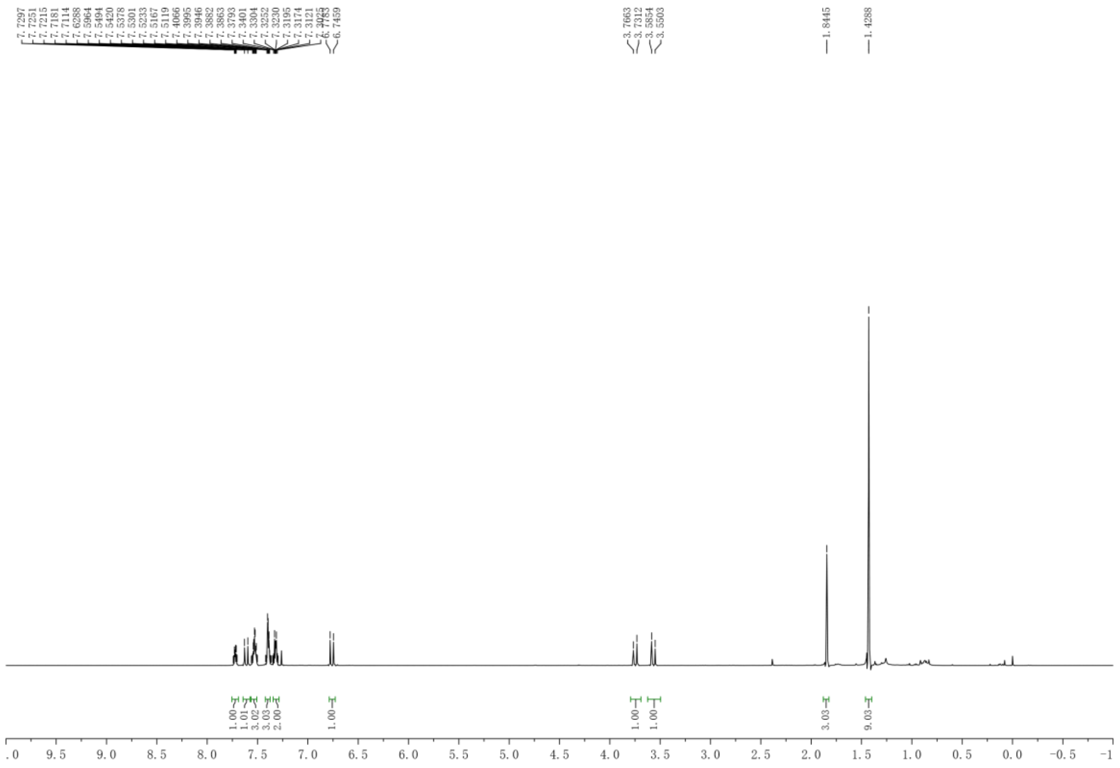

^{13}C NMR spectrum of **5t**

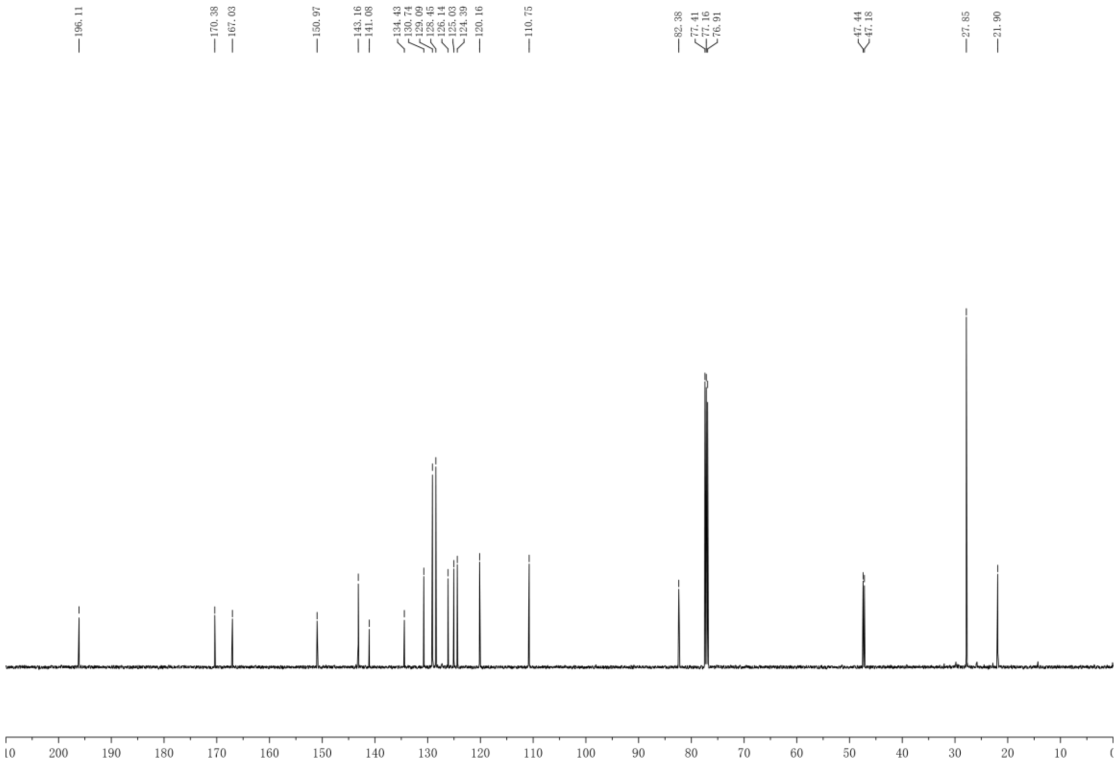

^{1}H NMR spectrum of **5u-***major isomer*

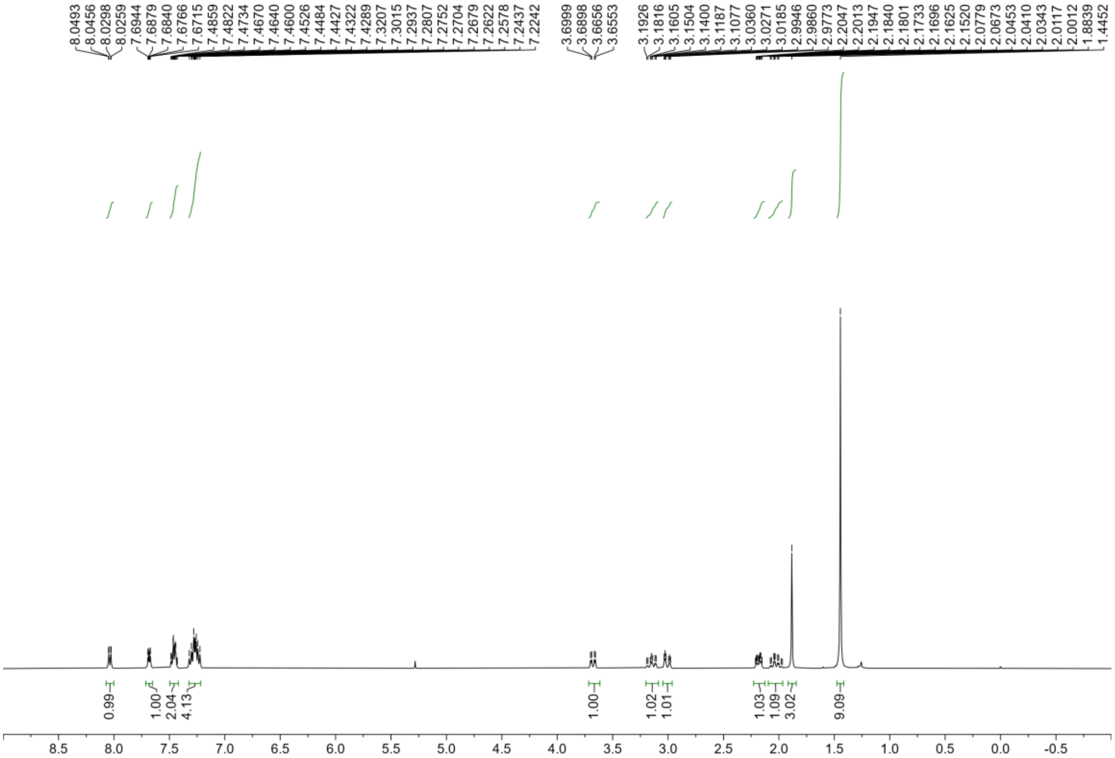

^{13}C NMR spectrum of **5u-***major isomer*

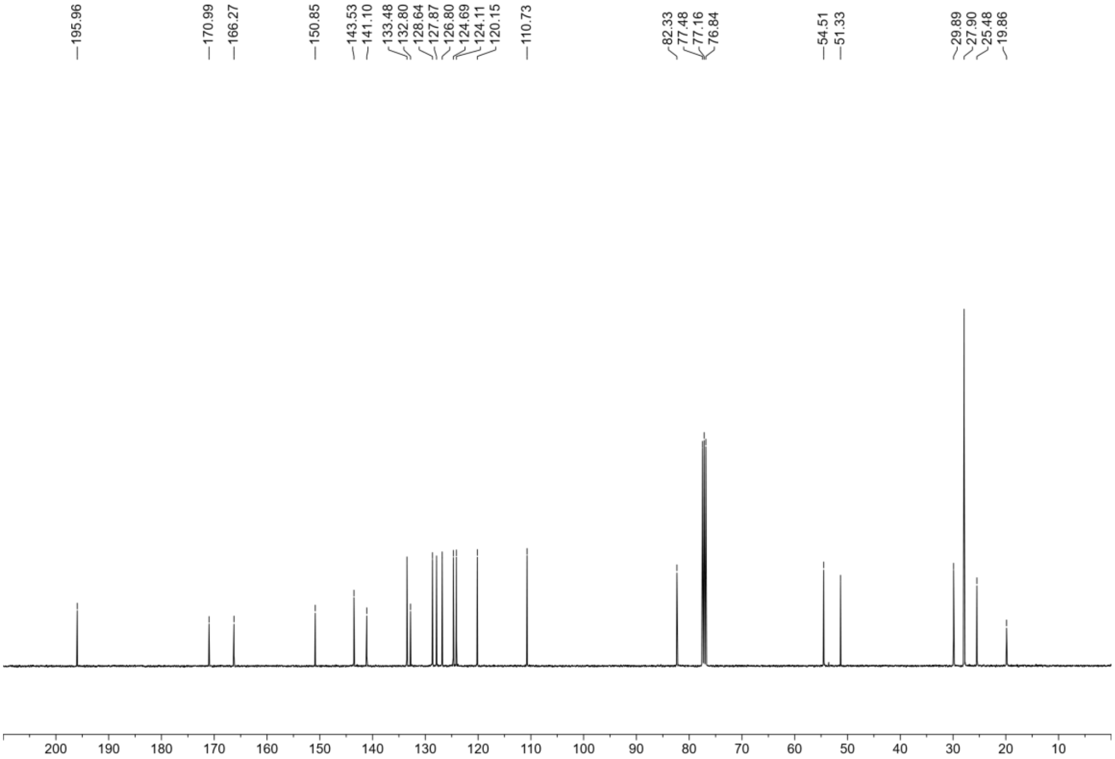

^{1}H NMR spectrum of **5u-***minor isomer*

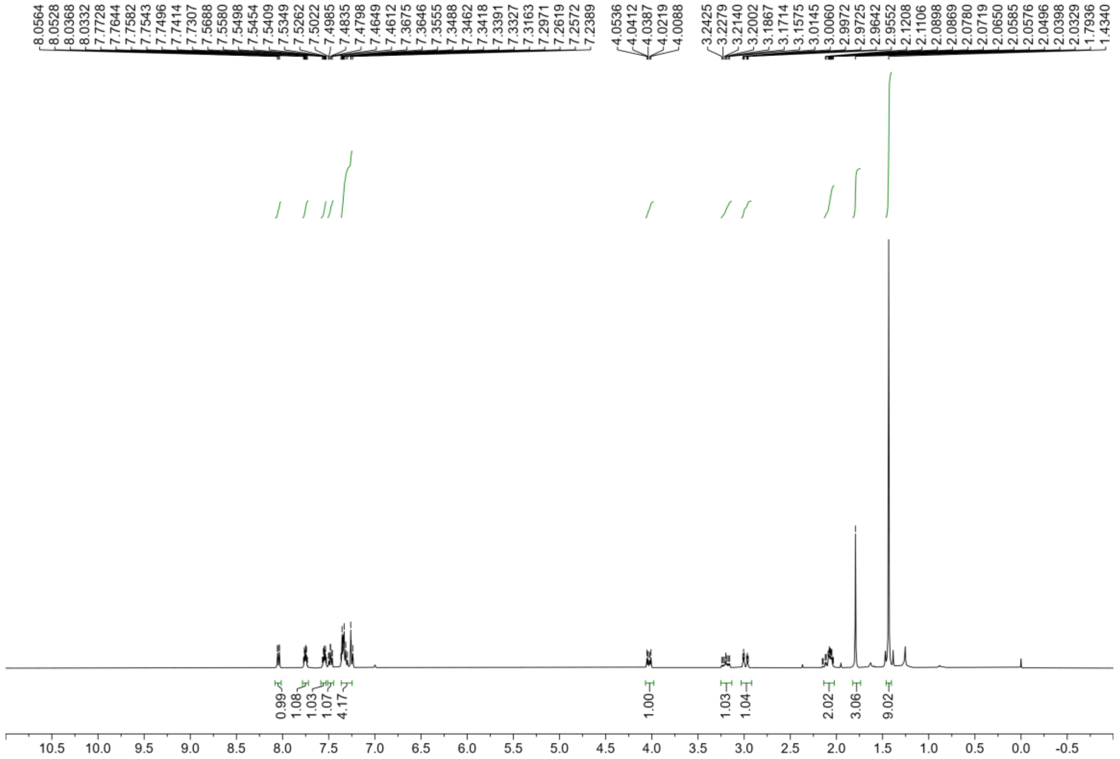

^{13}C NMR spectrum of **5u-***minor isomer*

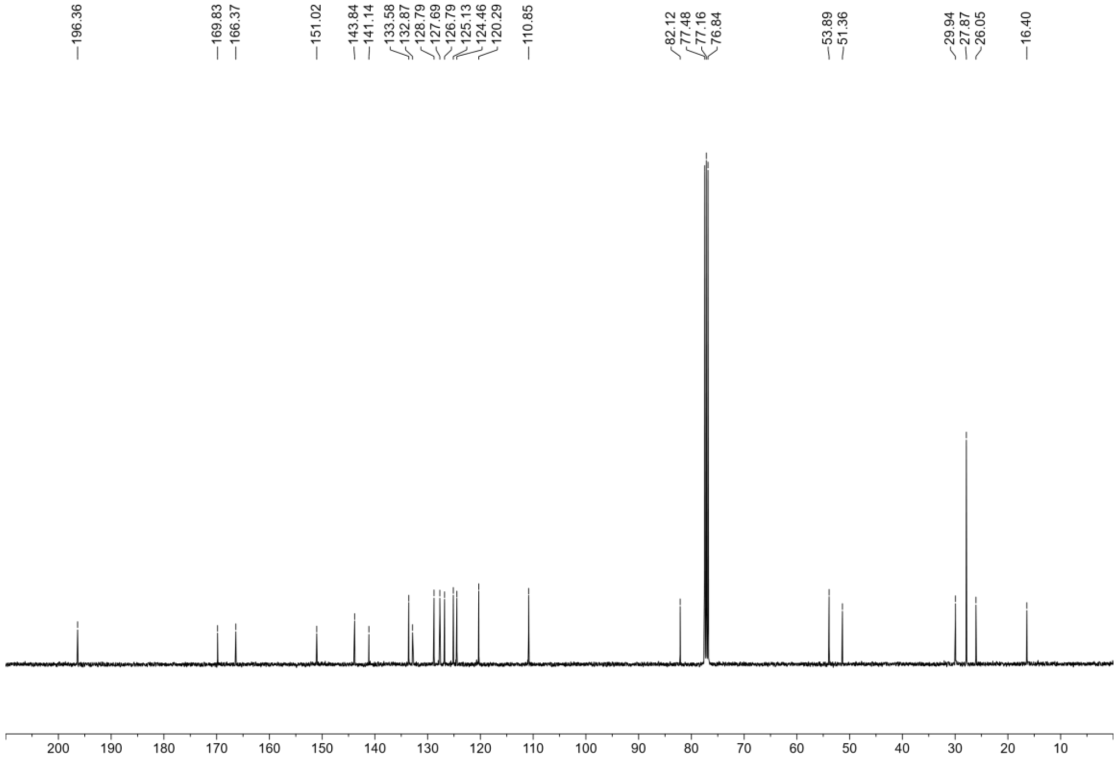

^{1}H NMR spectrum of **5v-***major isomer*

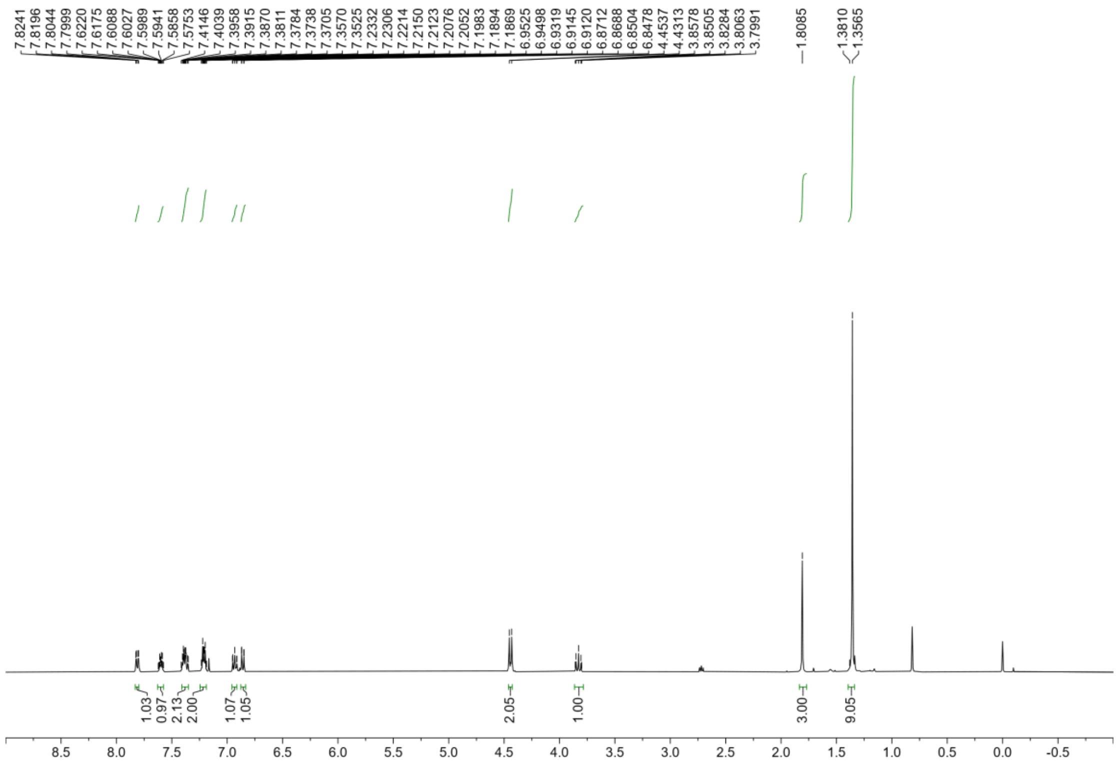

^{13}C NMR spectrum of **5v-***major isomer*

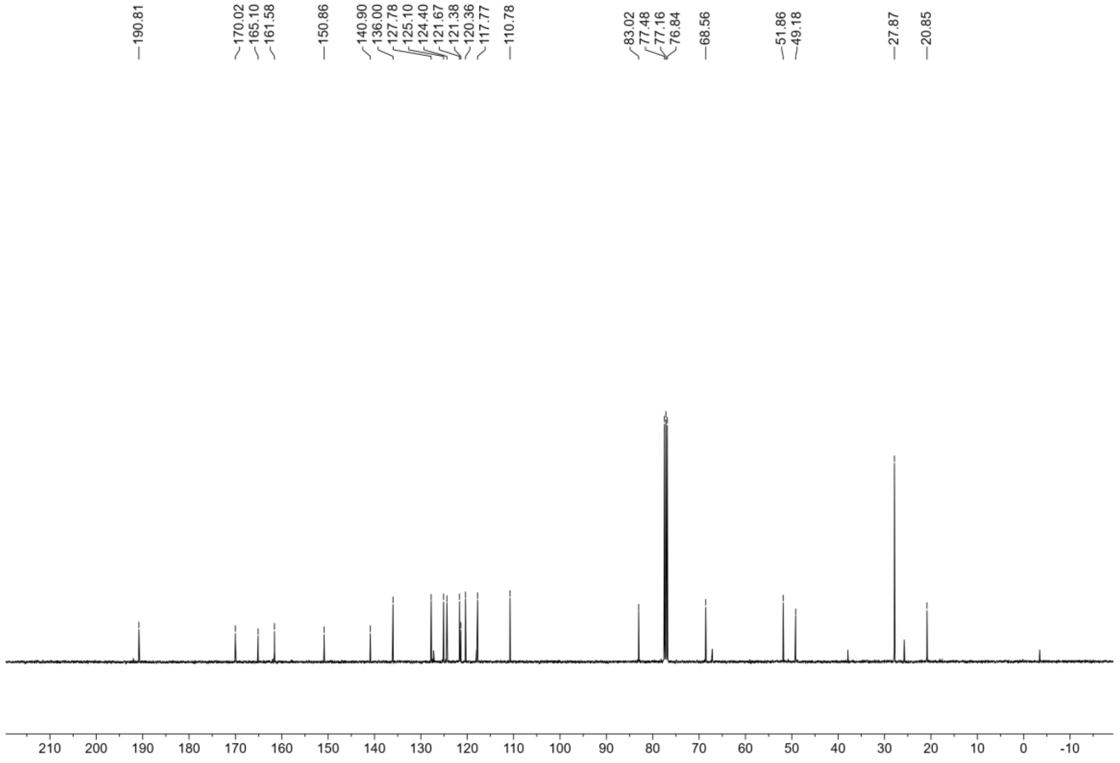

^{1}H NMR spectrum of **5v**-*minor isomer*

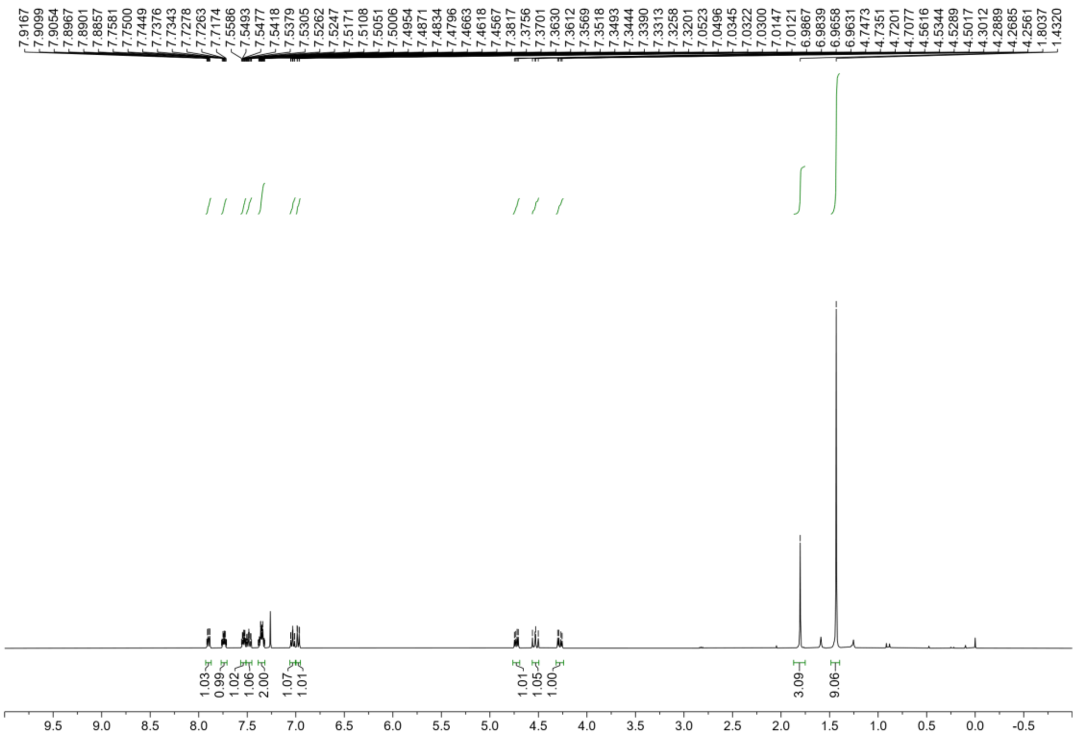

^{13}C NMR spectrum of **5v**-*minor isomer*

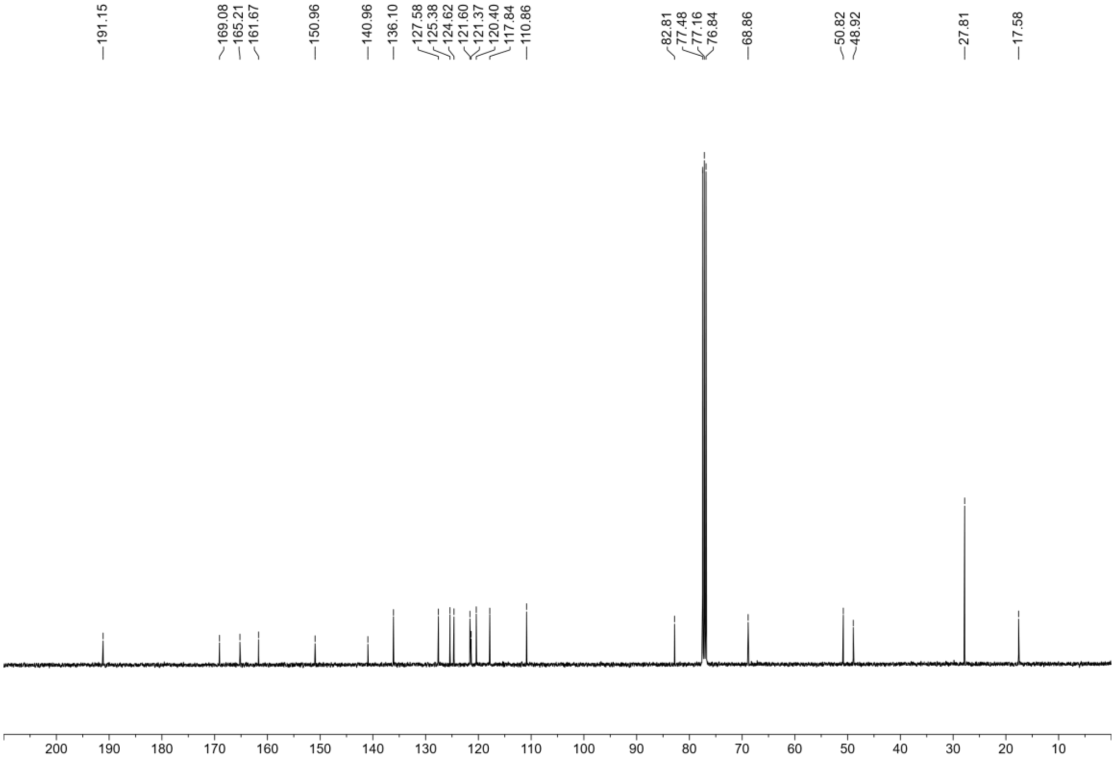

^{1}H NMR spectrum of **5w**

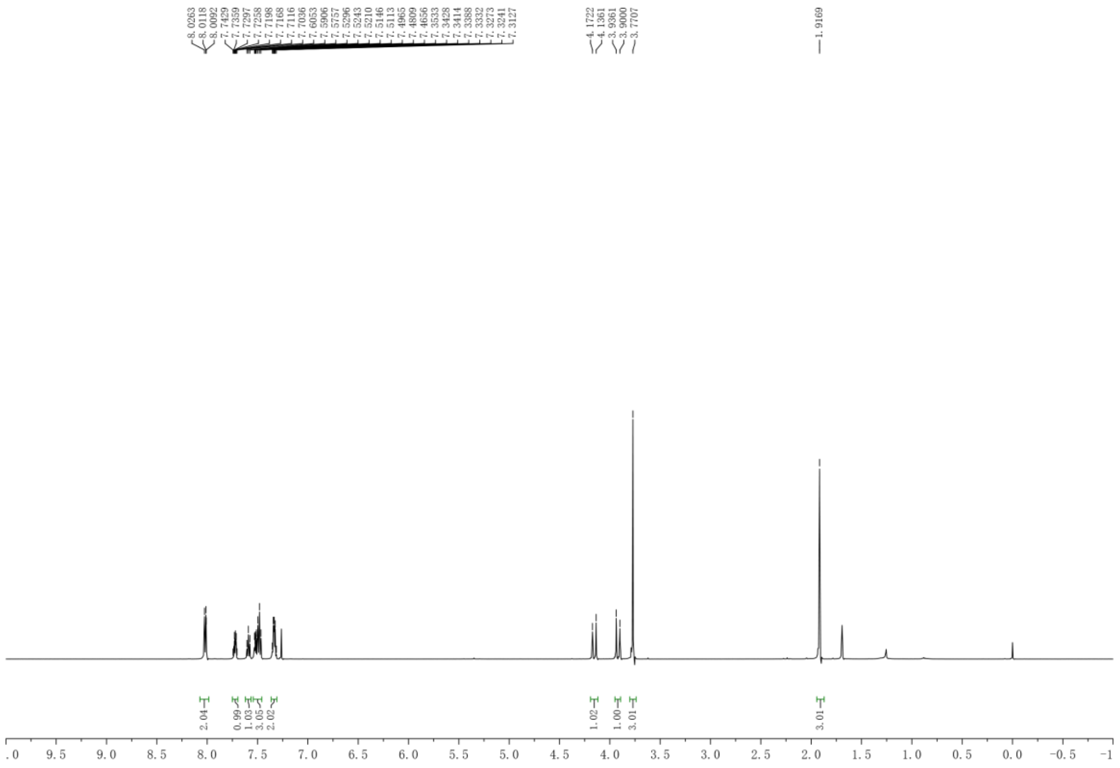

^{13}C NMR spectrum of **5w**

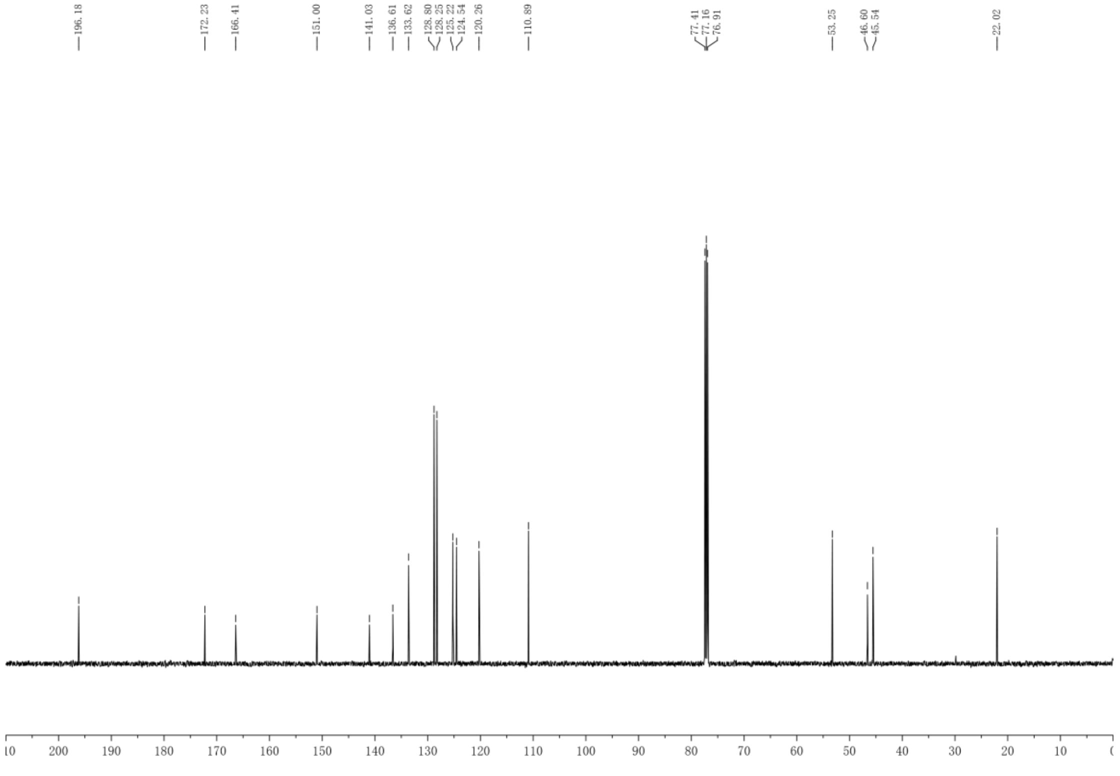

^{1}H NMR spectrum of **5x**

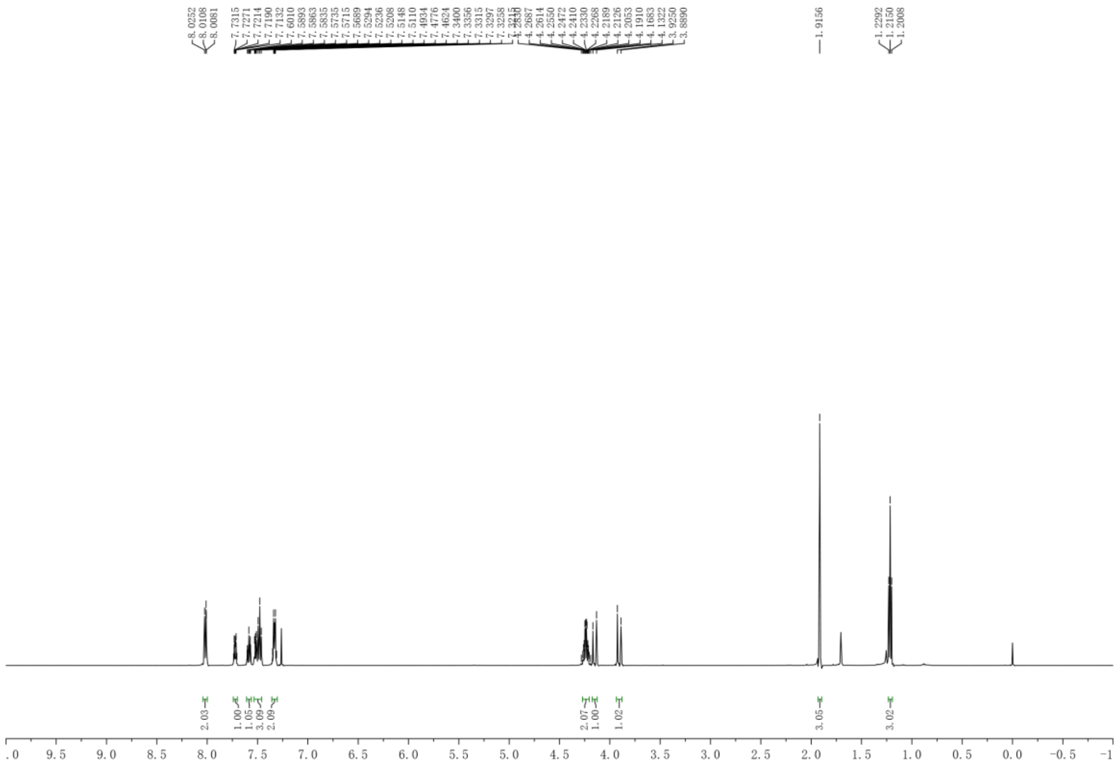

^{13}C NMR spectrum of **5x**

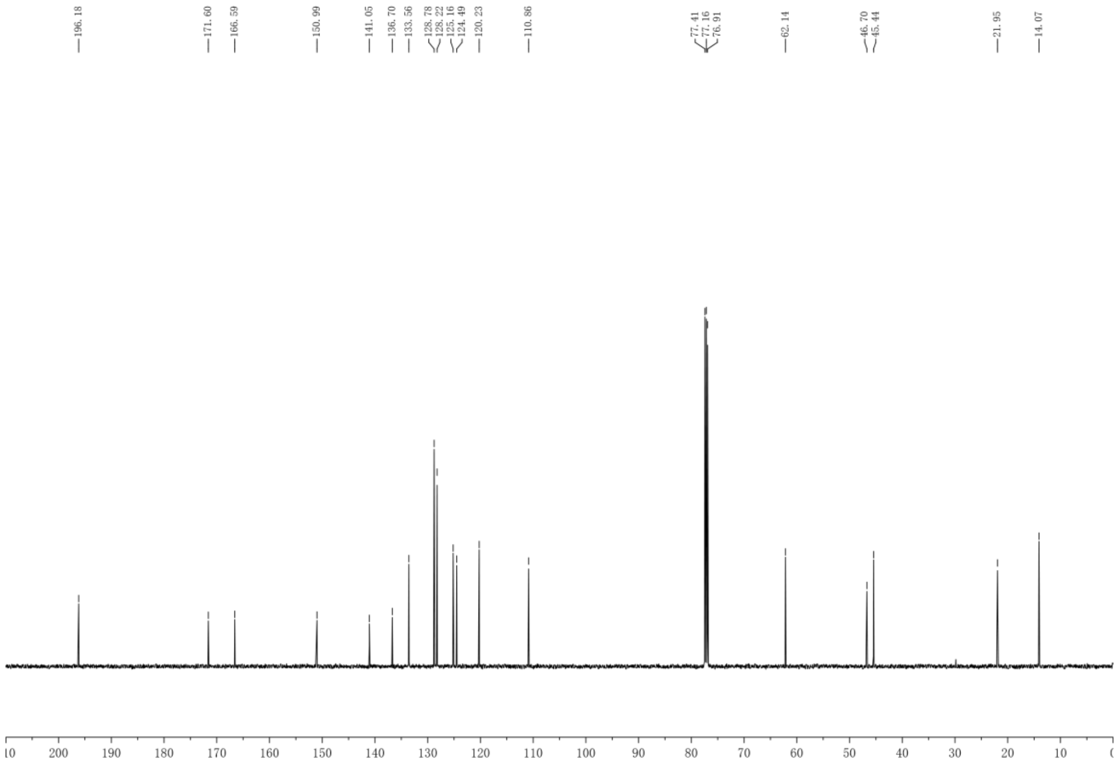

^{1}H NMR spectrum of **5y**

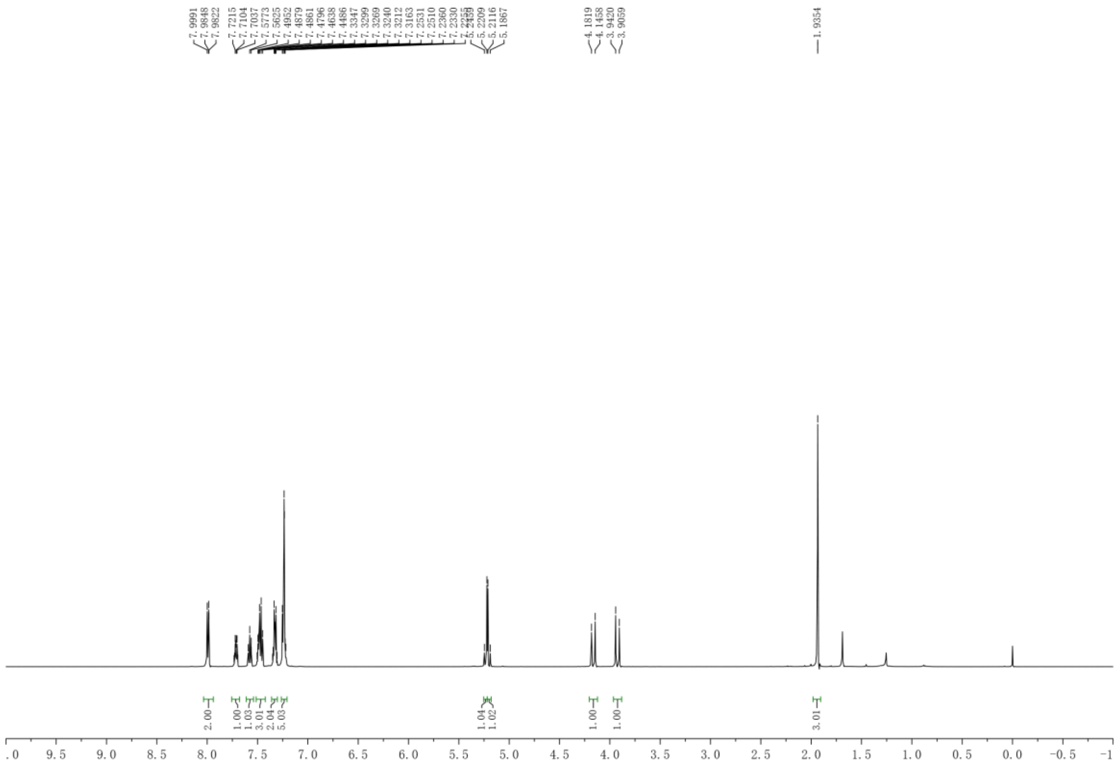

^{13}C NMR spectrum of **5y**

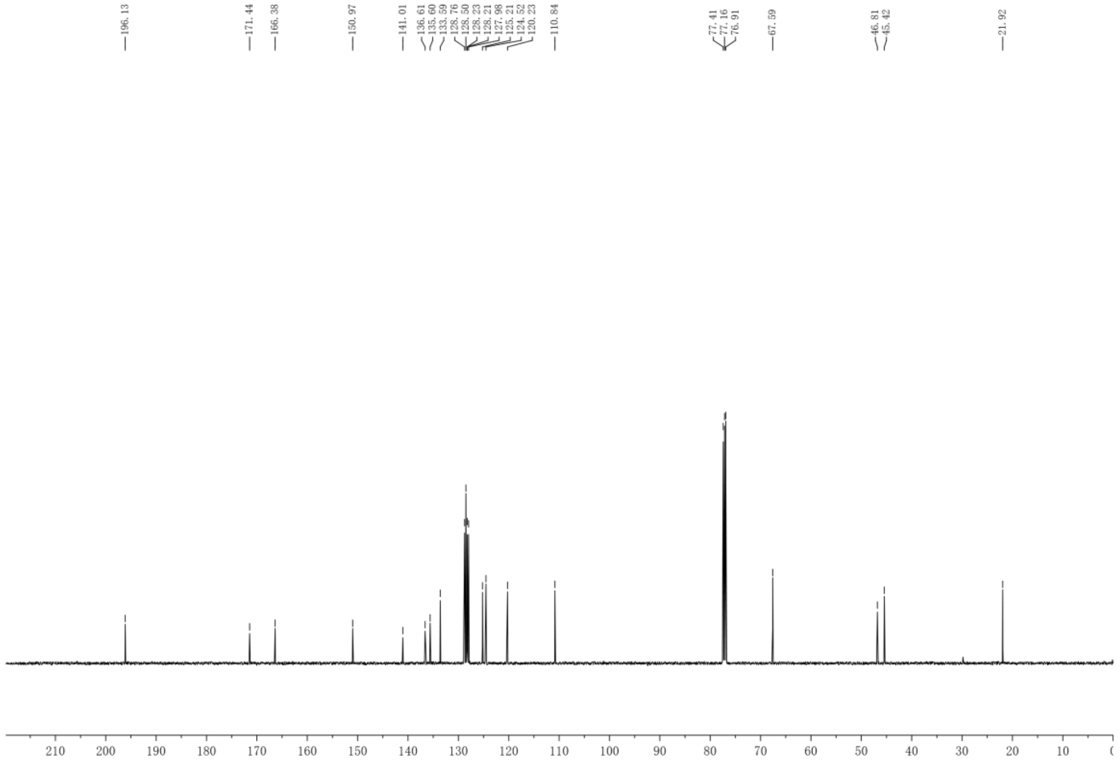

^{1}H NMR spectrum of **5z**

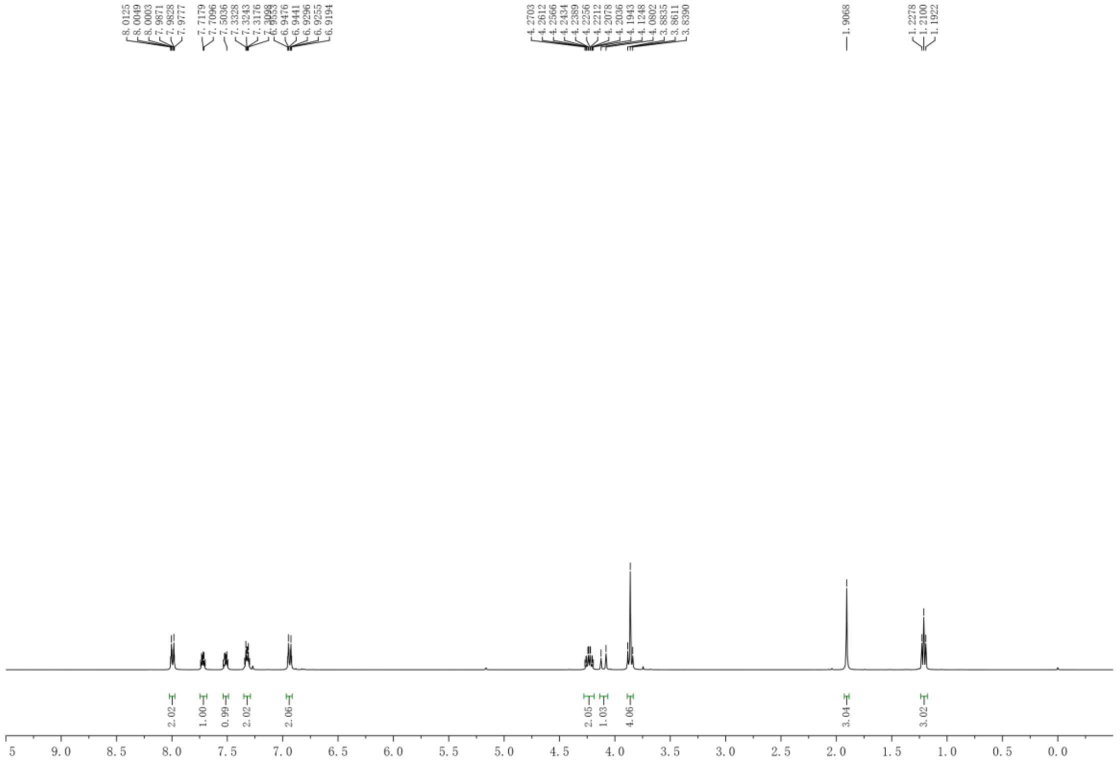

^{13}C NMR spectrum of **5z**

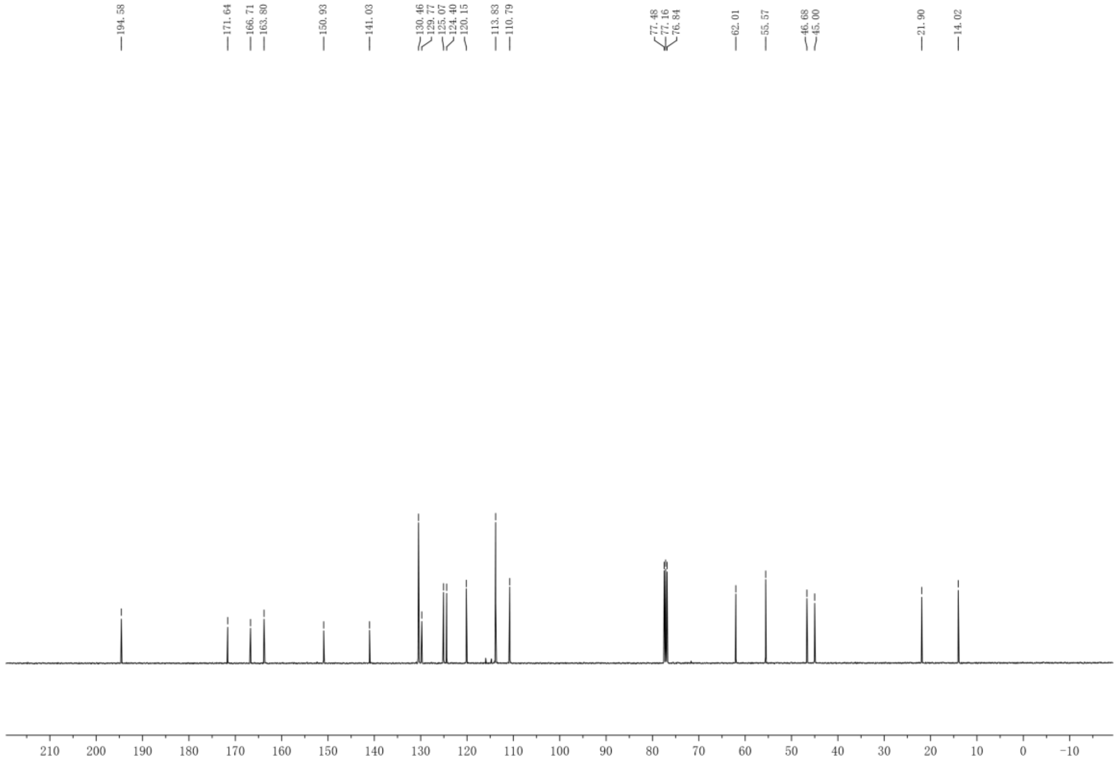

^{1}H NMR spectrum of **5aa**

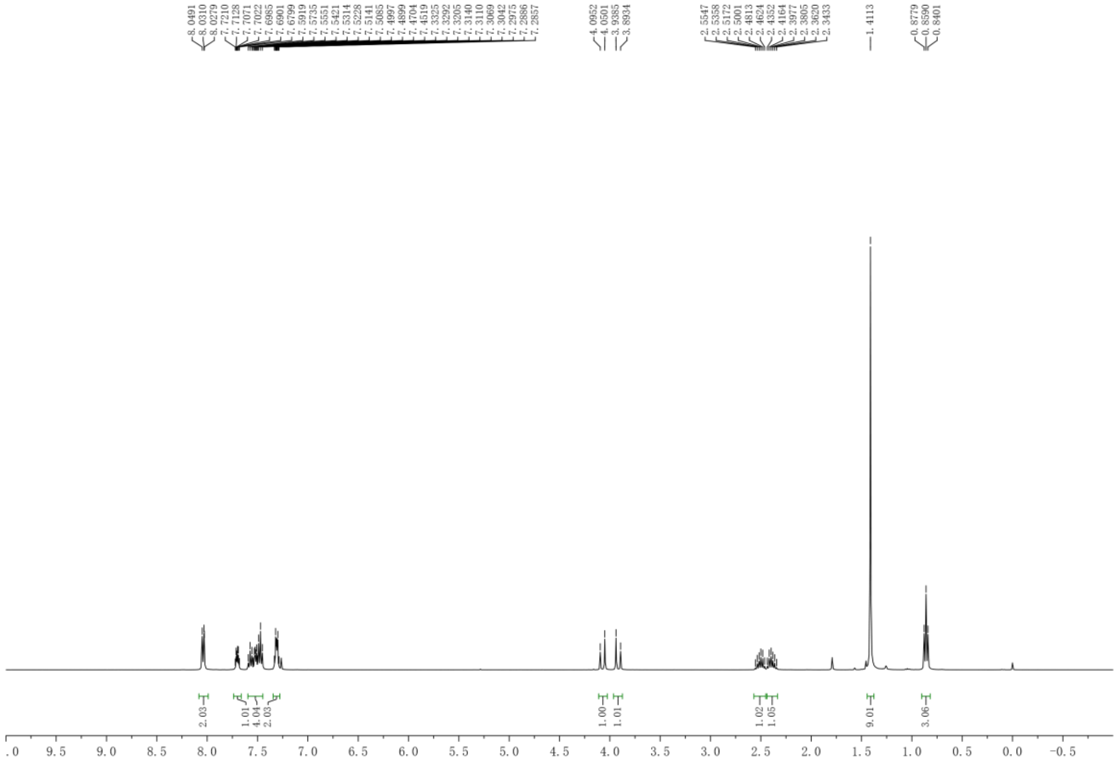

^{13}C NMR spectrum of **5aa**

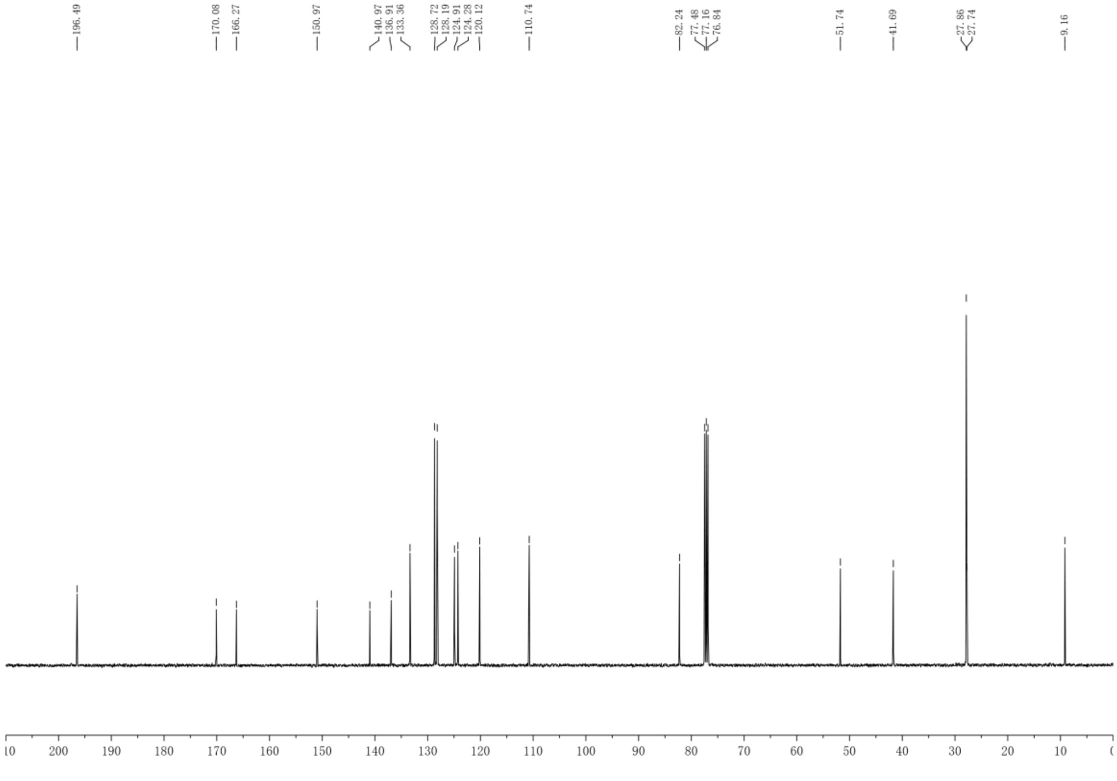

^{1}H NMR spectrum of 5ab

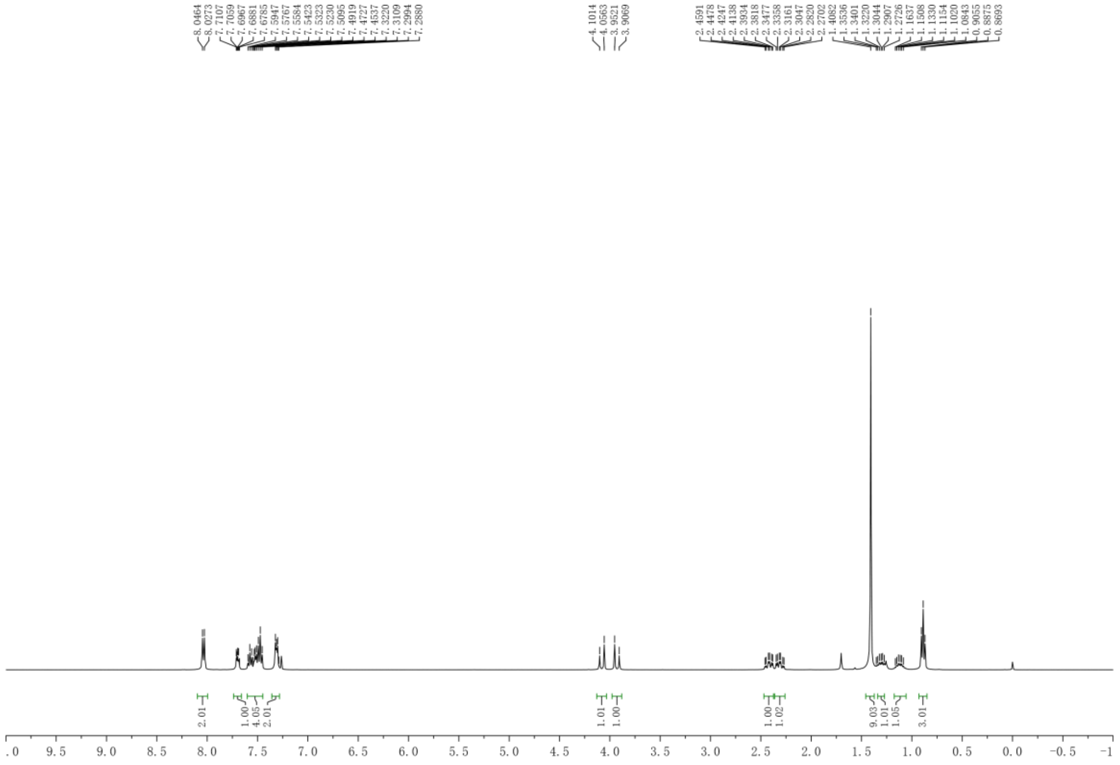

^{13}C NMR spectrum of 5ab

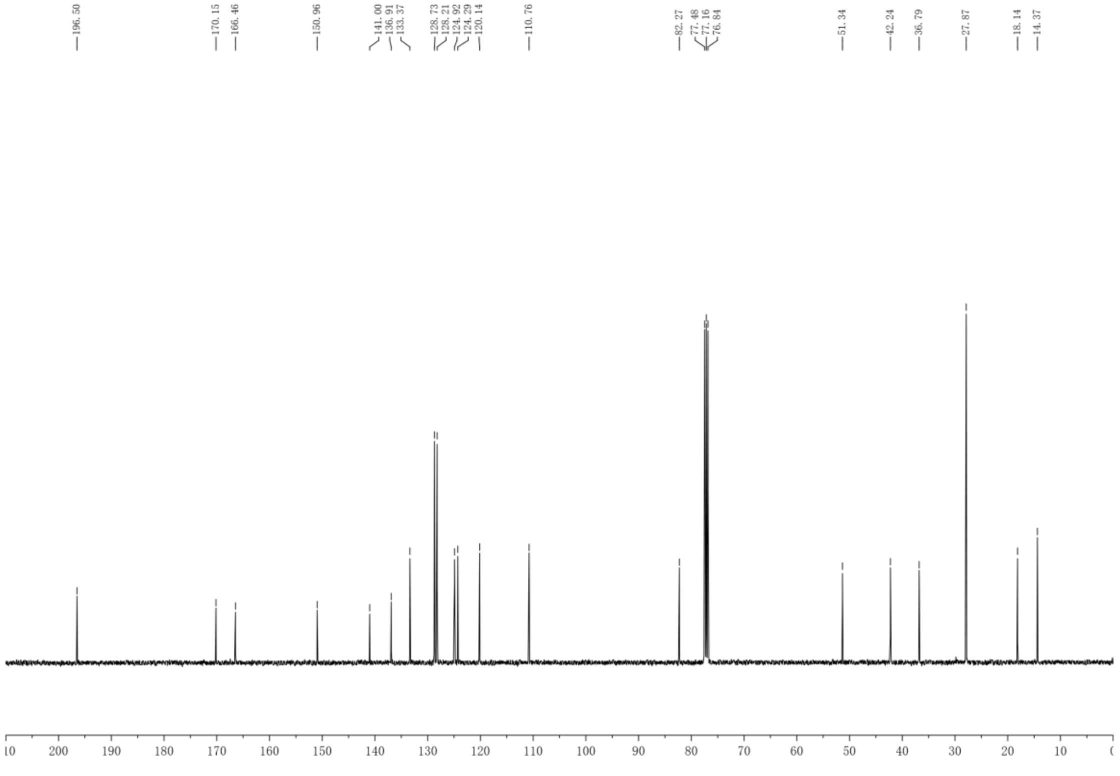

^{1}H NMR spectrum of **5ac**

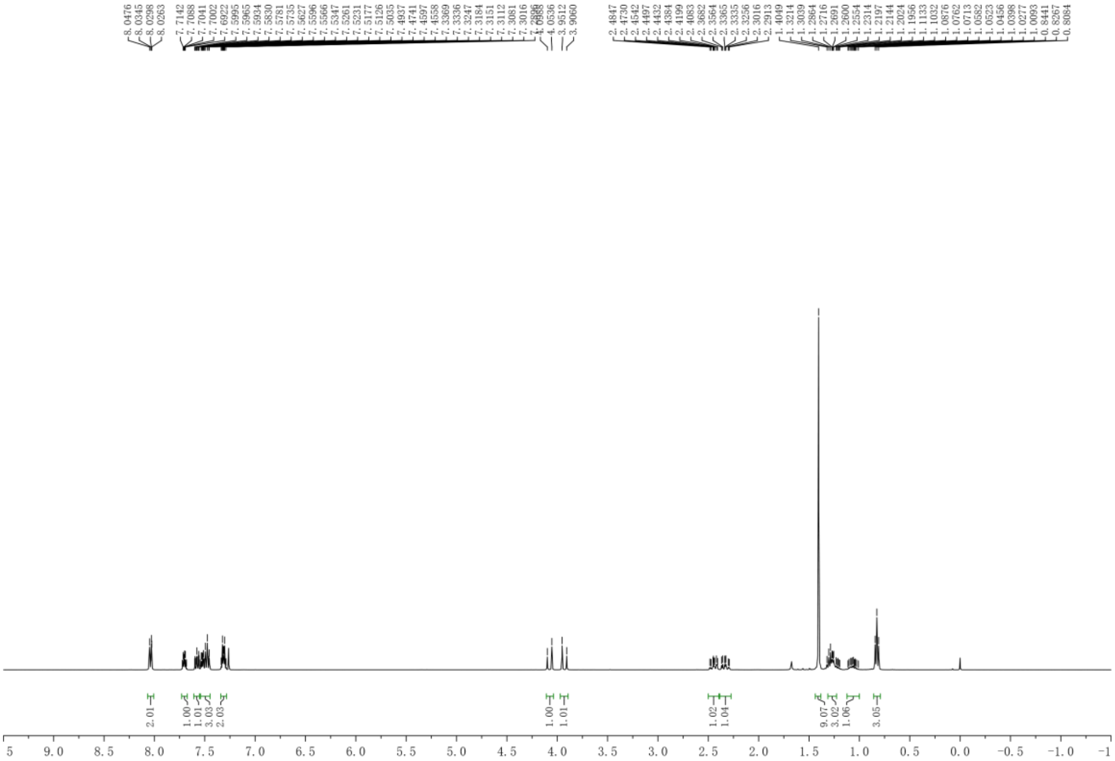

^{13}C NMR spectrum of **5ac**

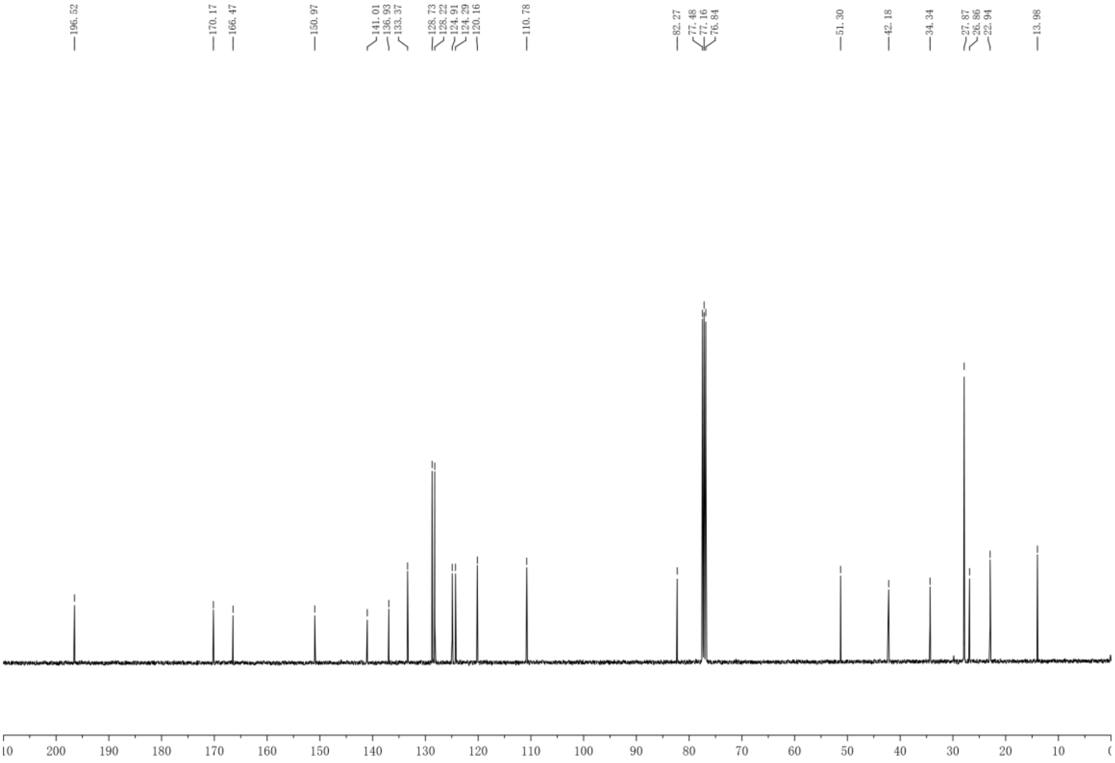

^{1}H NMR spectrum of **5ad**

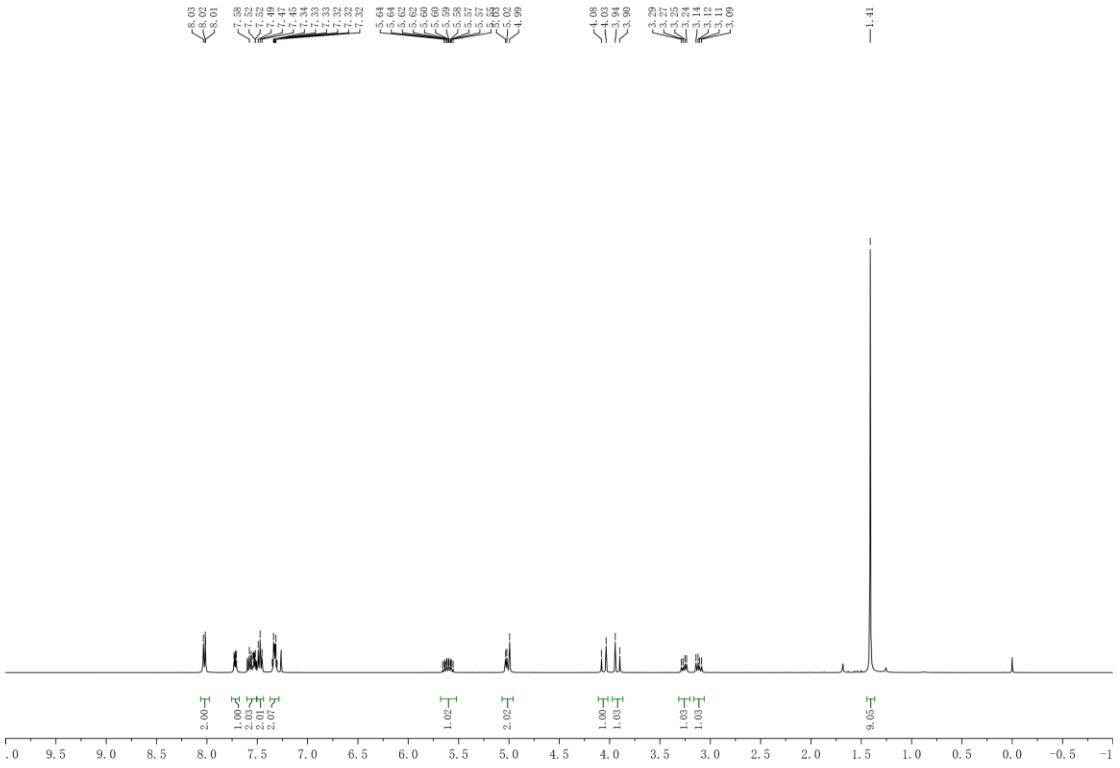

^{13}C NMR spectrum of **5ad**

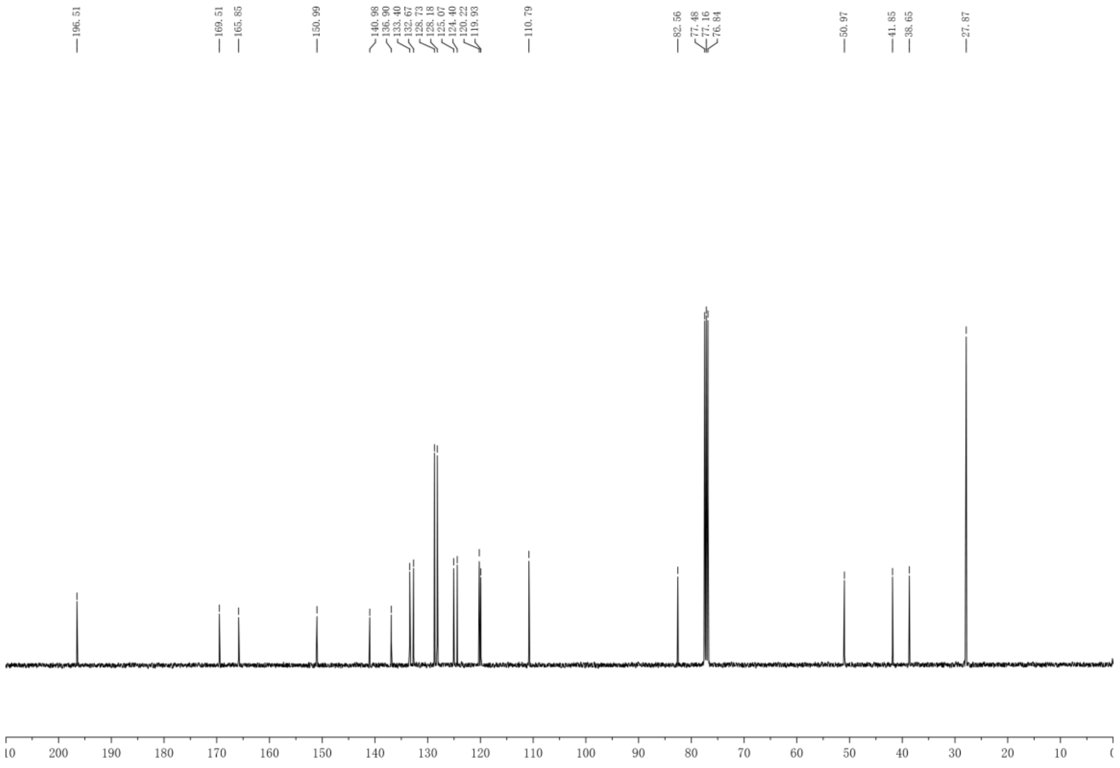

^{1}H NMR spectrum of **5ae**

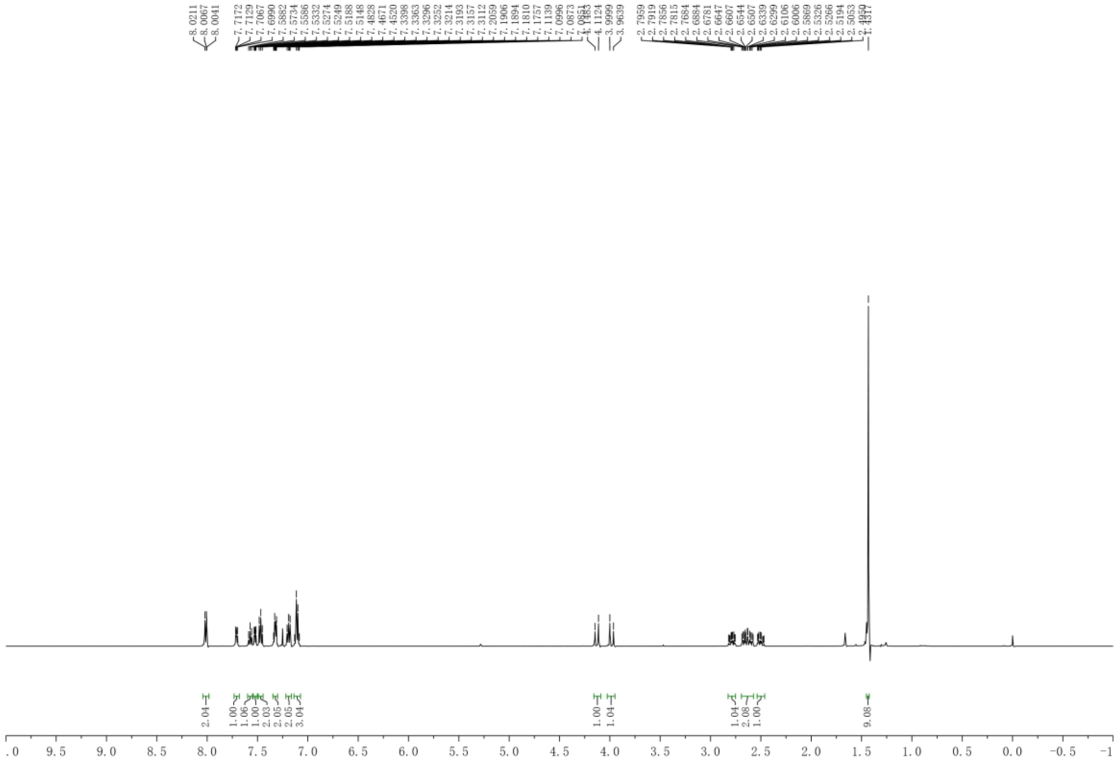

^{13}C NMR spectrum of **5ae**

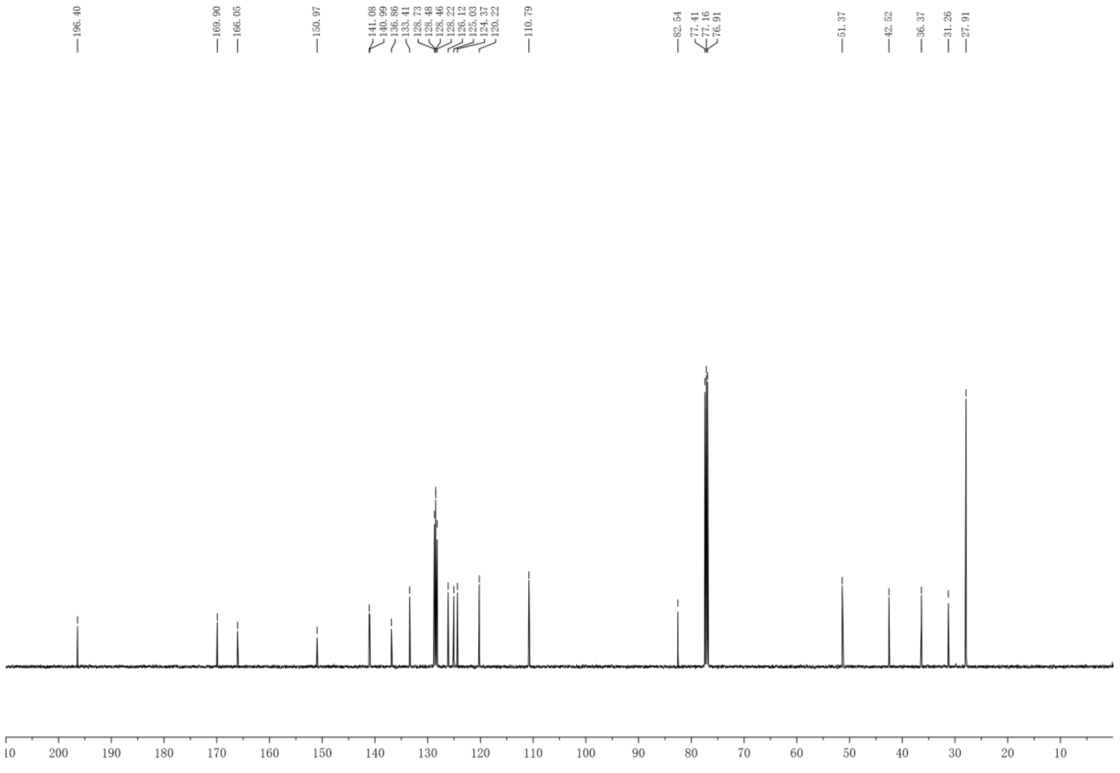

^{1}H NMR spectrum of **5af**

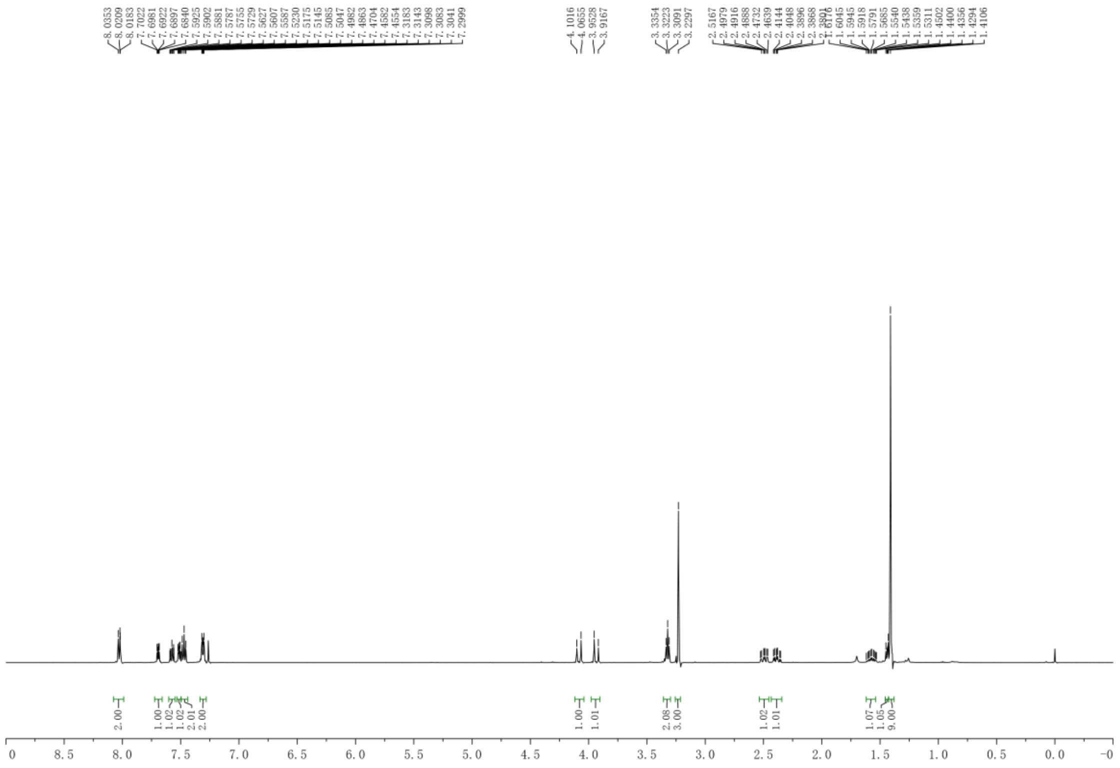

^{13}C NMR spectrum of **5af**

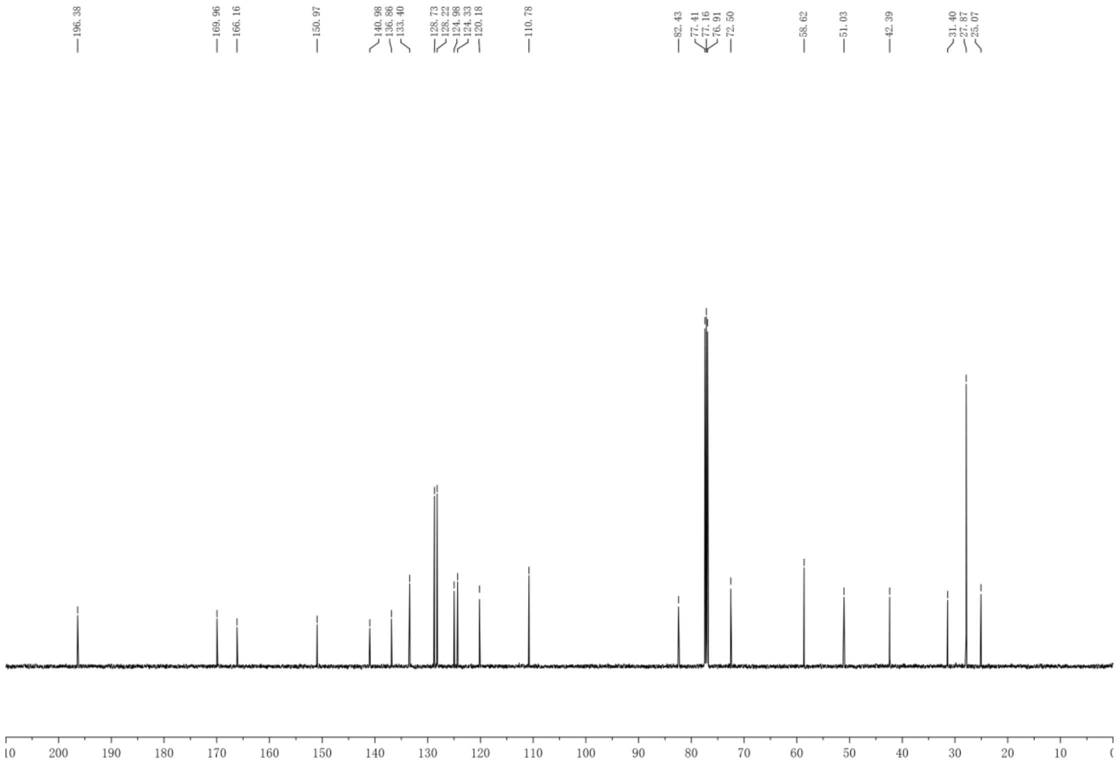

^{1}**H NMR** spectrum of **5ag**

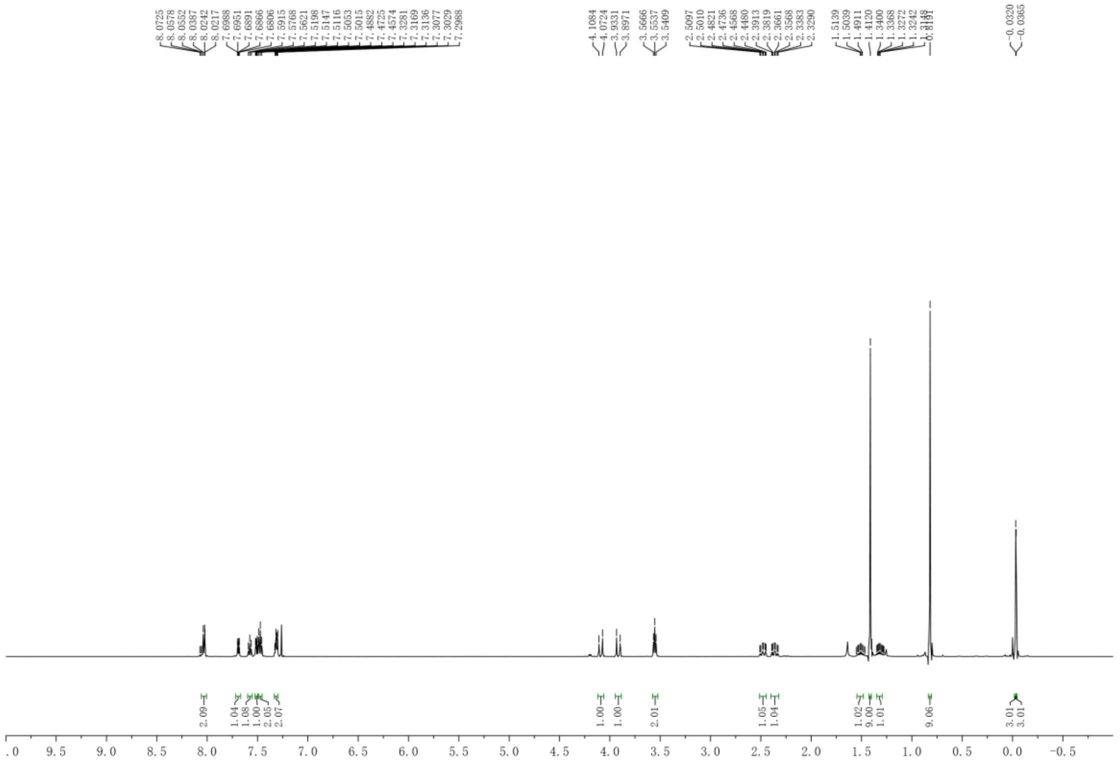

^{13}**C NMR** spectrum of **5ag**

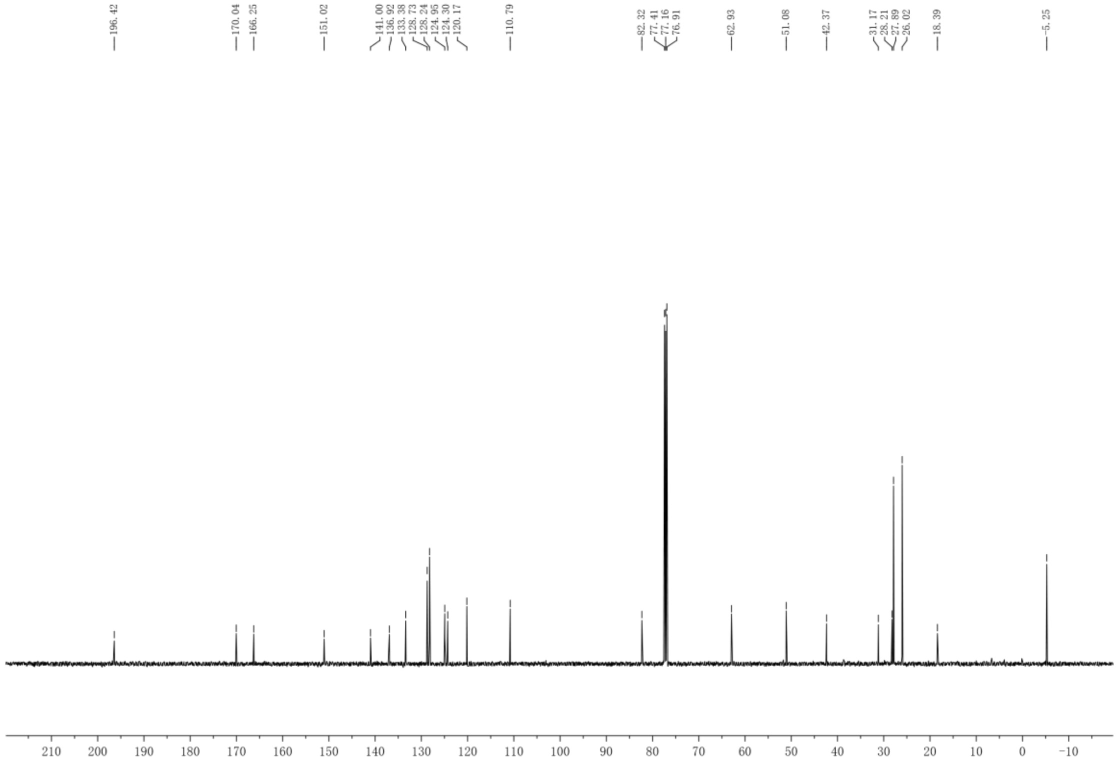

^{1}H NMR spectrum of **5ah**

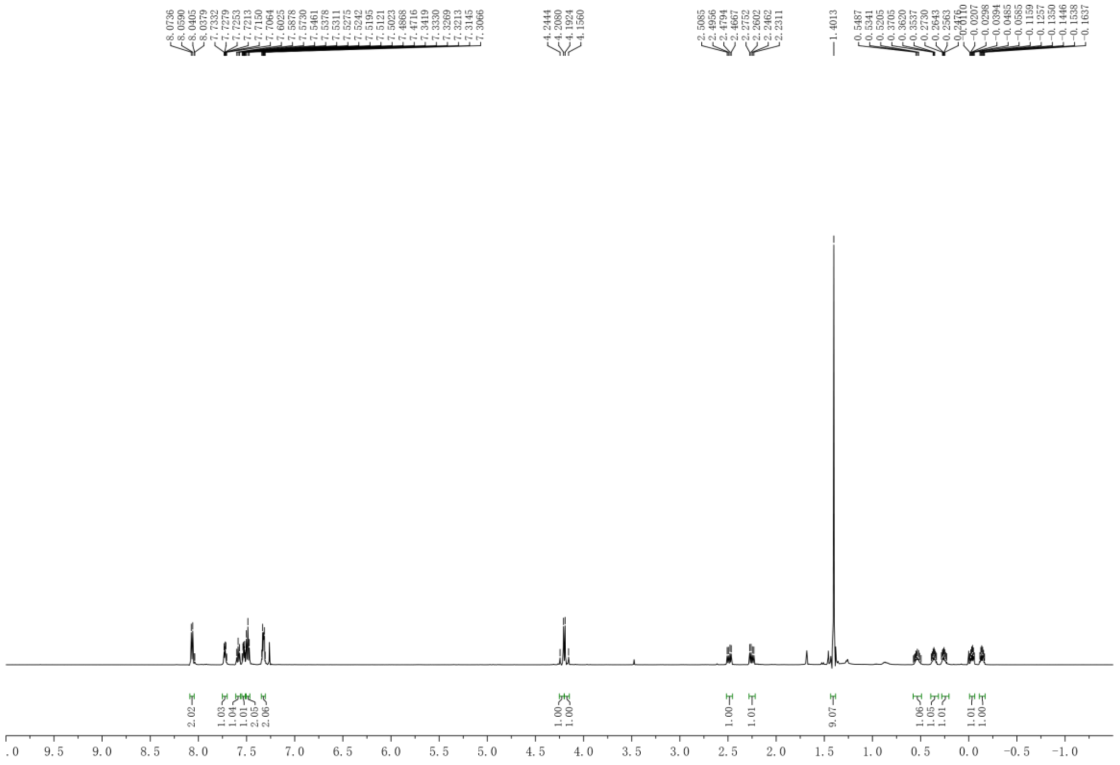

^{13}C NMR spectrum of **5ah**

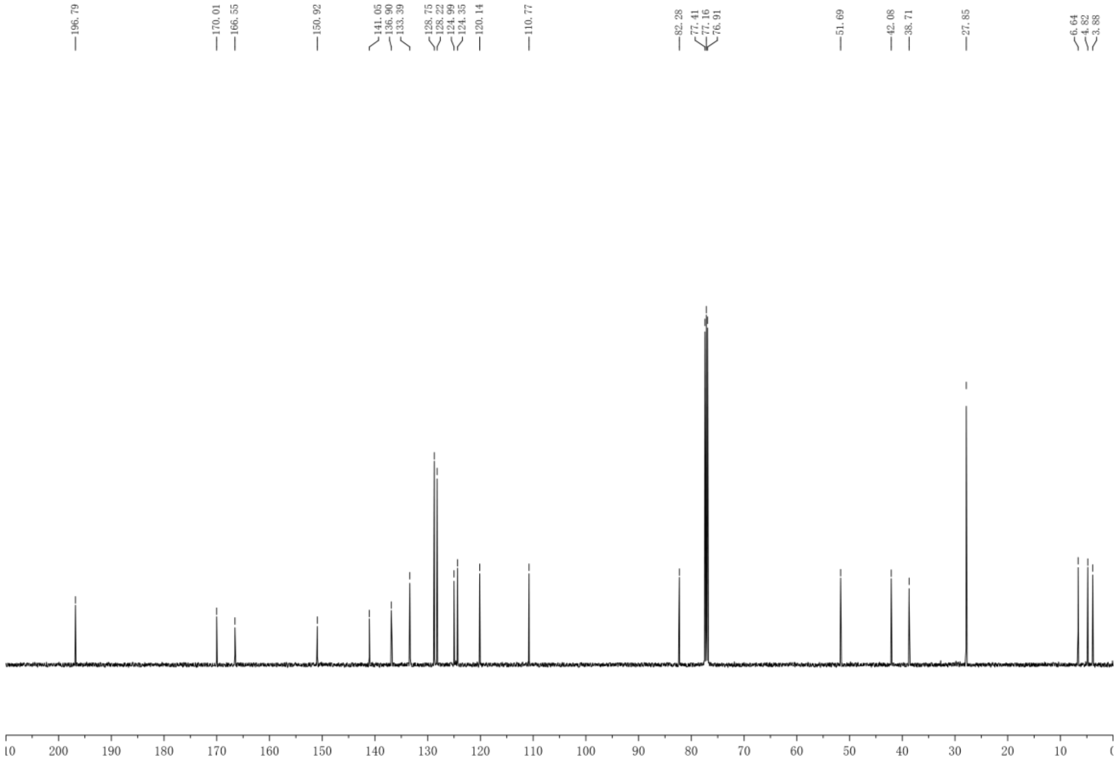

^{1}H NMR spectrum of **6**

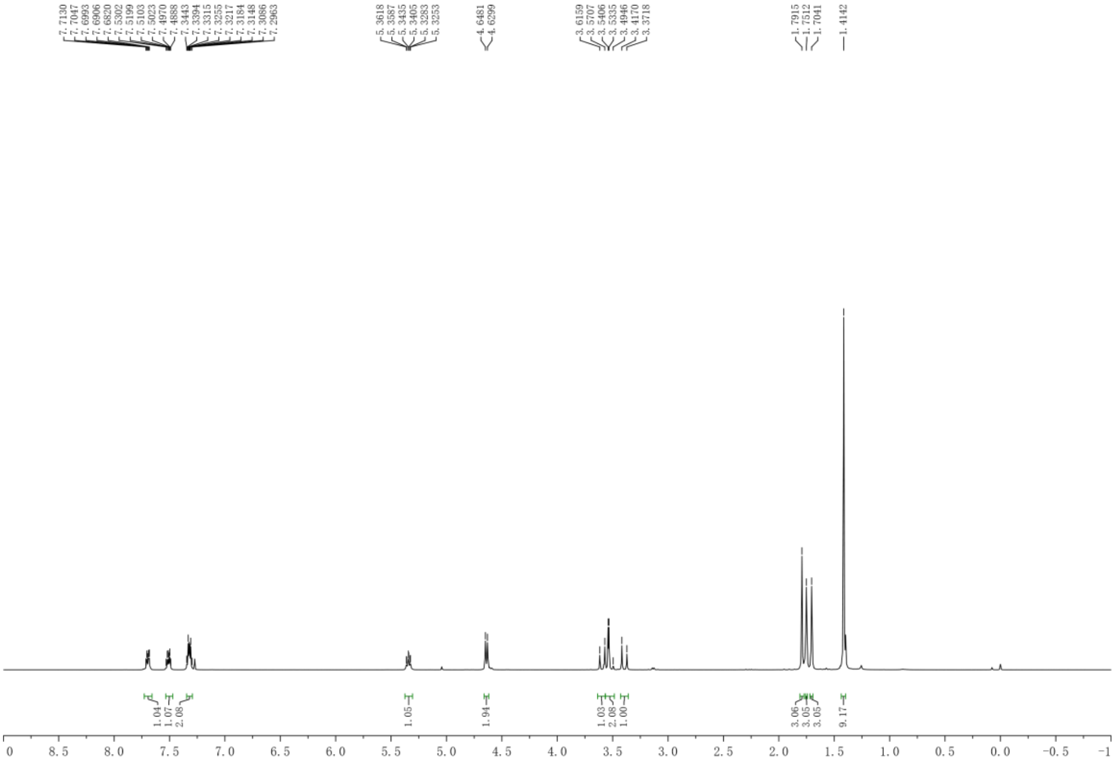

^{13}C NMR spectrum of **6**

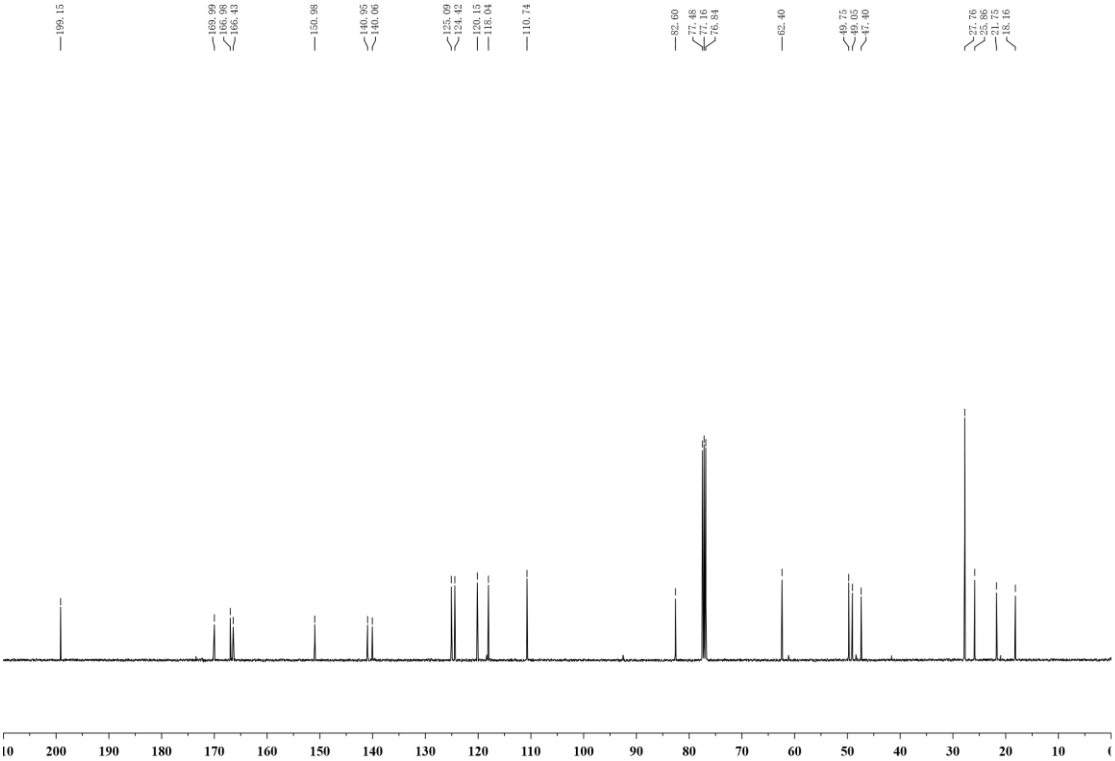

^{1}H NMR spectrum of **7**

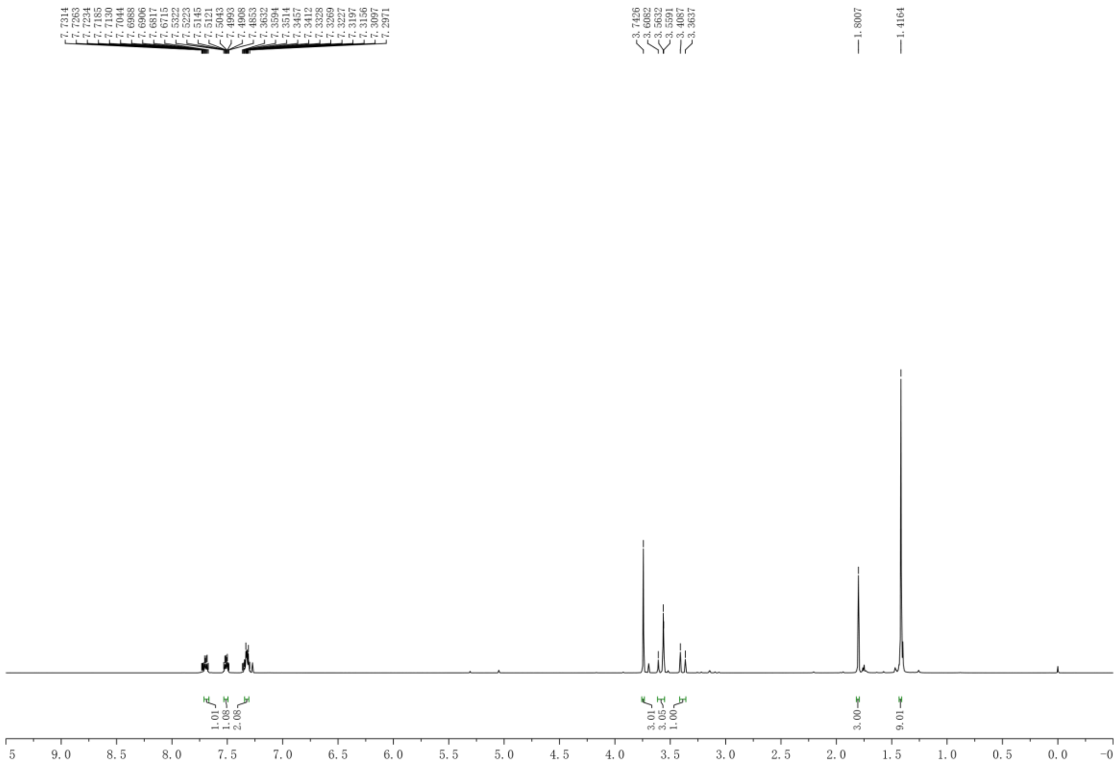

^{13}C NMR spectrum of **7**

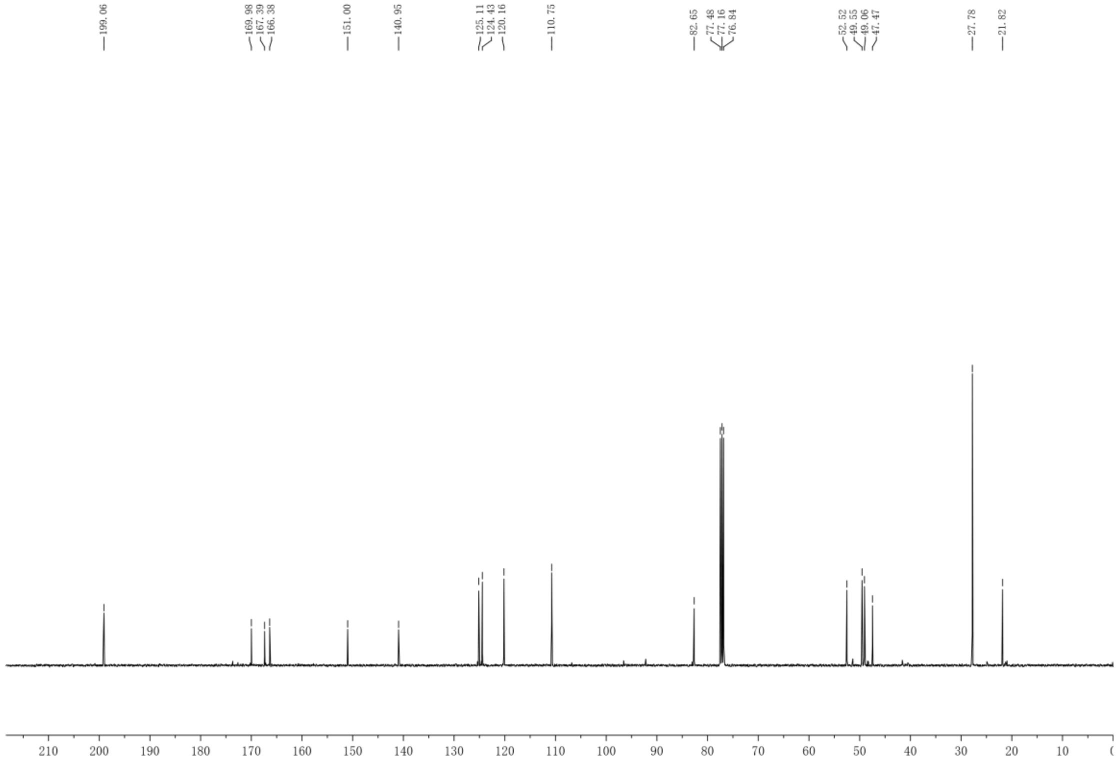

^{1}H NMR spectrum of **8**

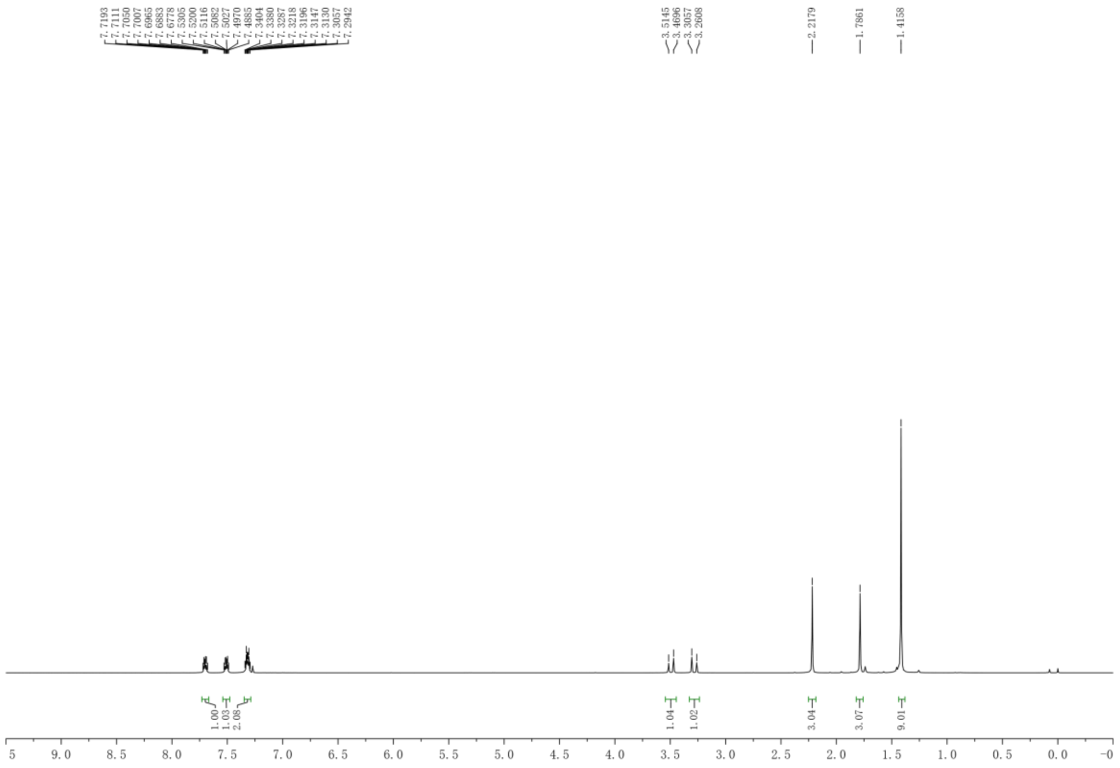

^{13}C NMR spectrum of **8**

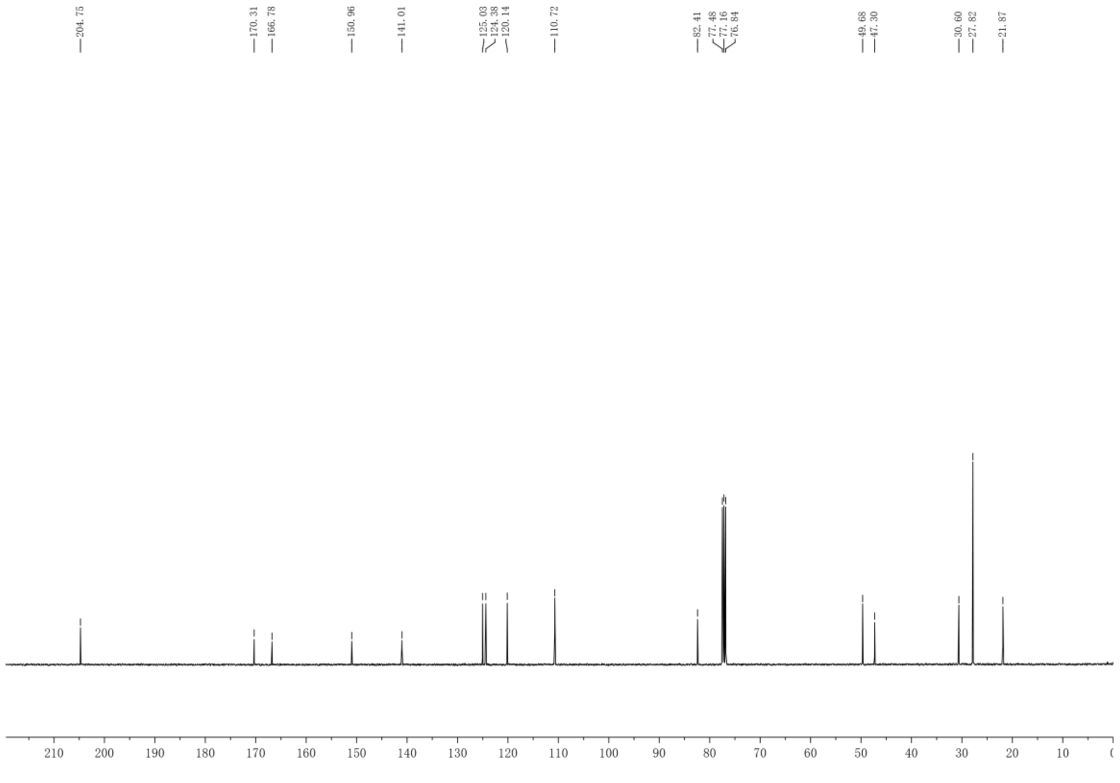

^{1}H NMR spectrum of **9**

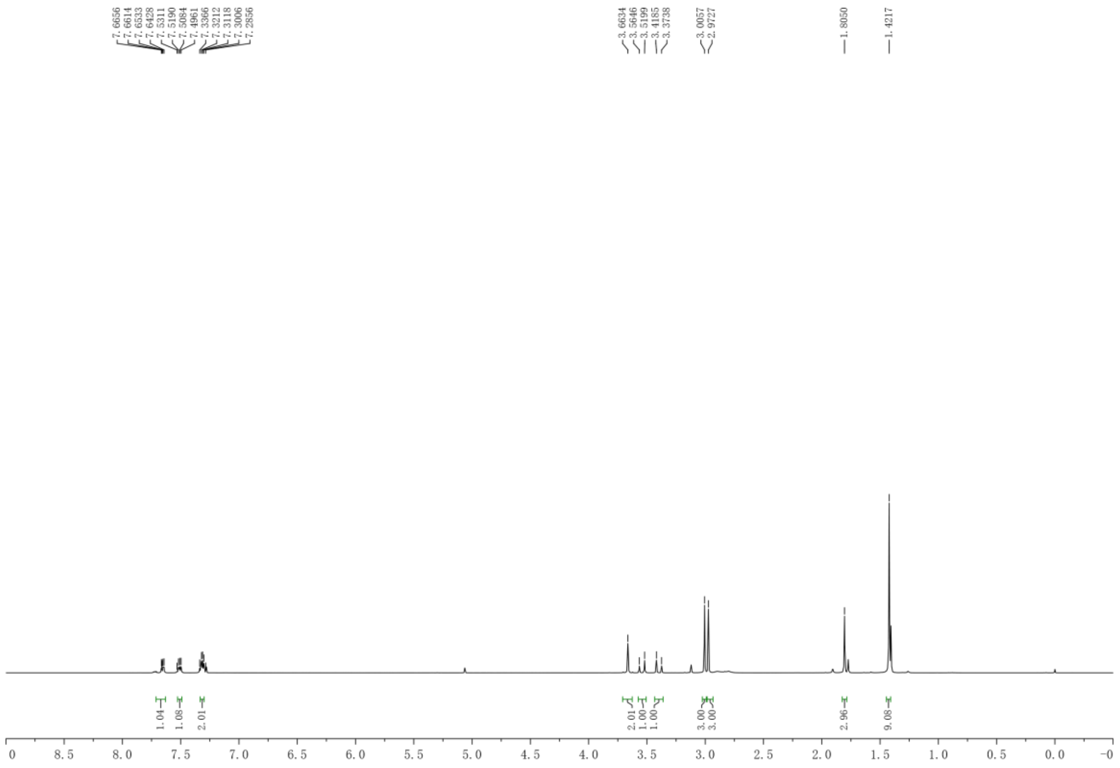

^{13}C NMR spectrum of **9**

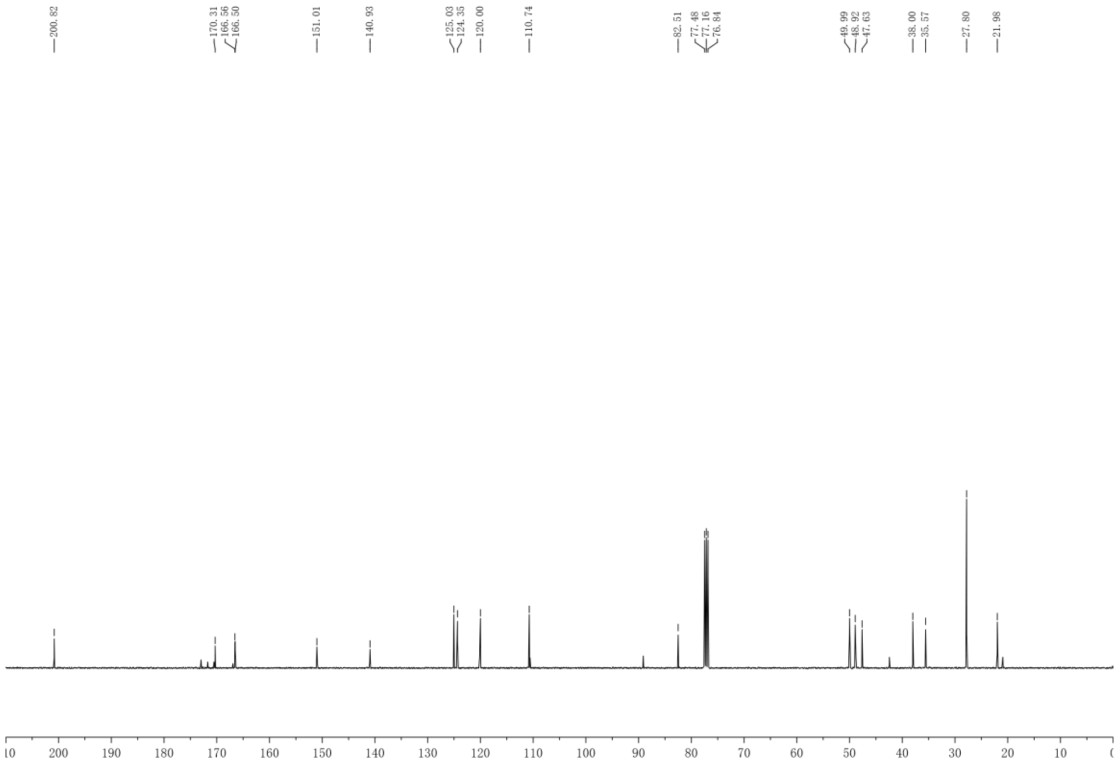

^{1}H NMR spectrum of **10**

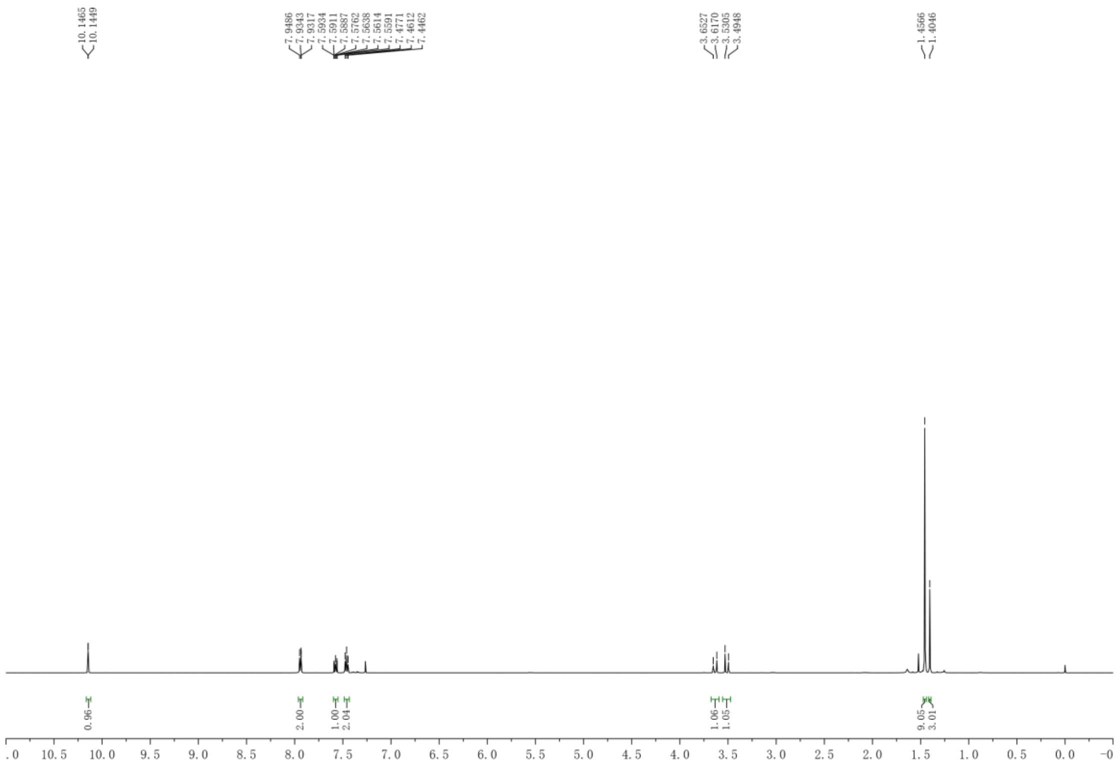

^{13}C NMR spectrum of **10**

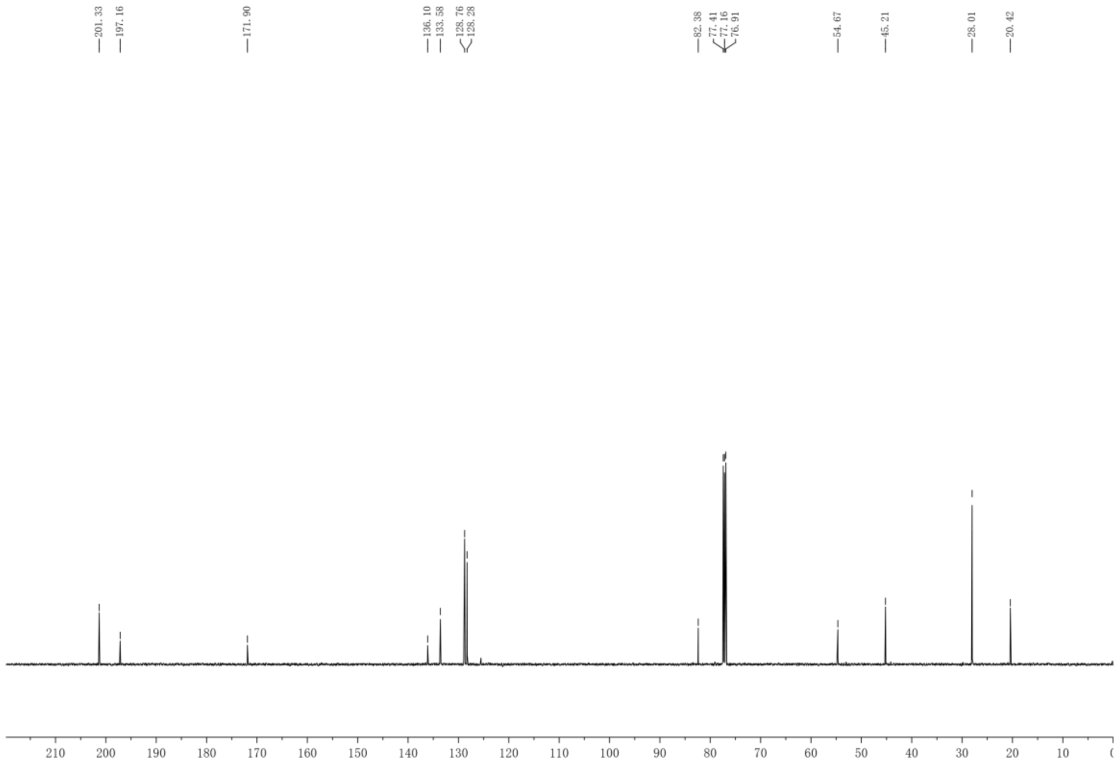

¹H NMR spectrum of **11**

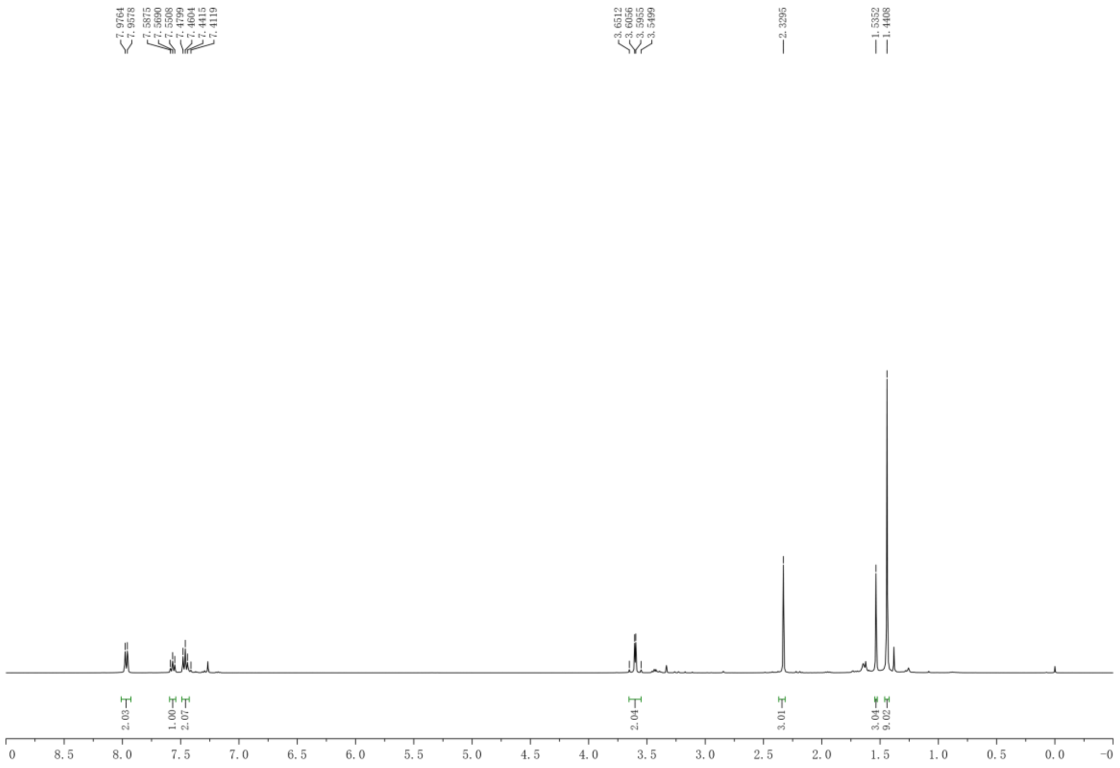

¹³C NMR spectrum of **11**

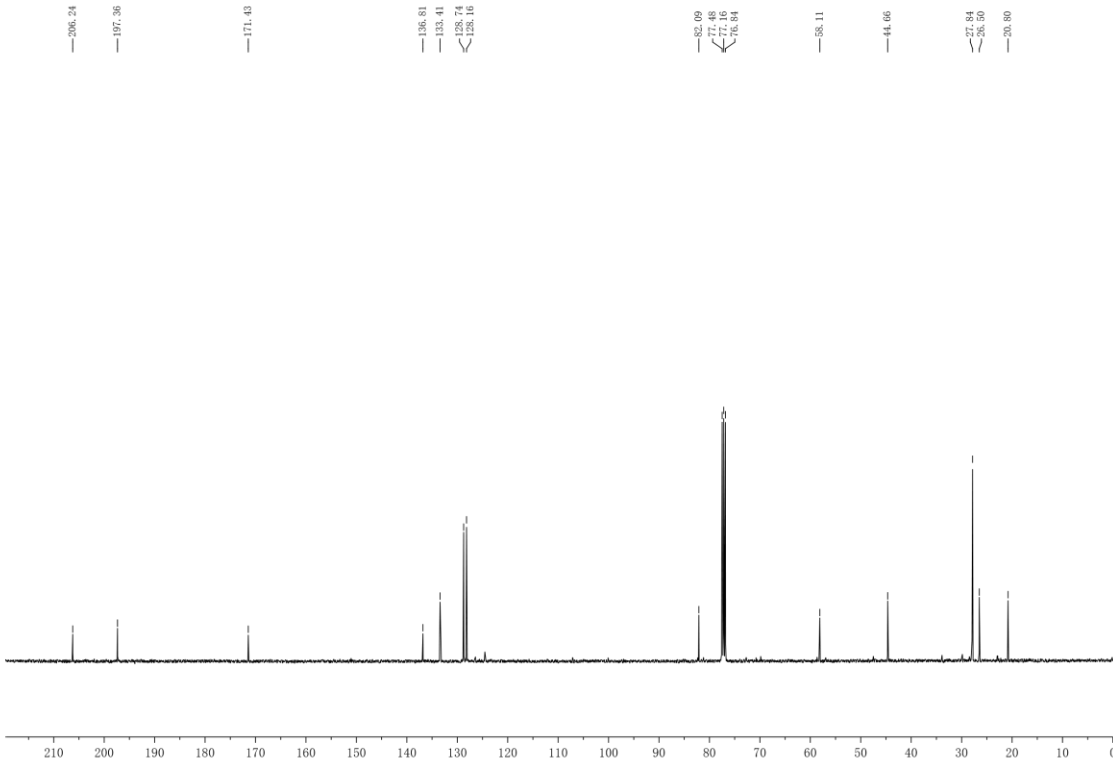

^{1}**H NMR** spectrum of **12**

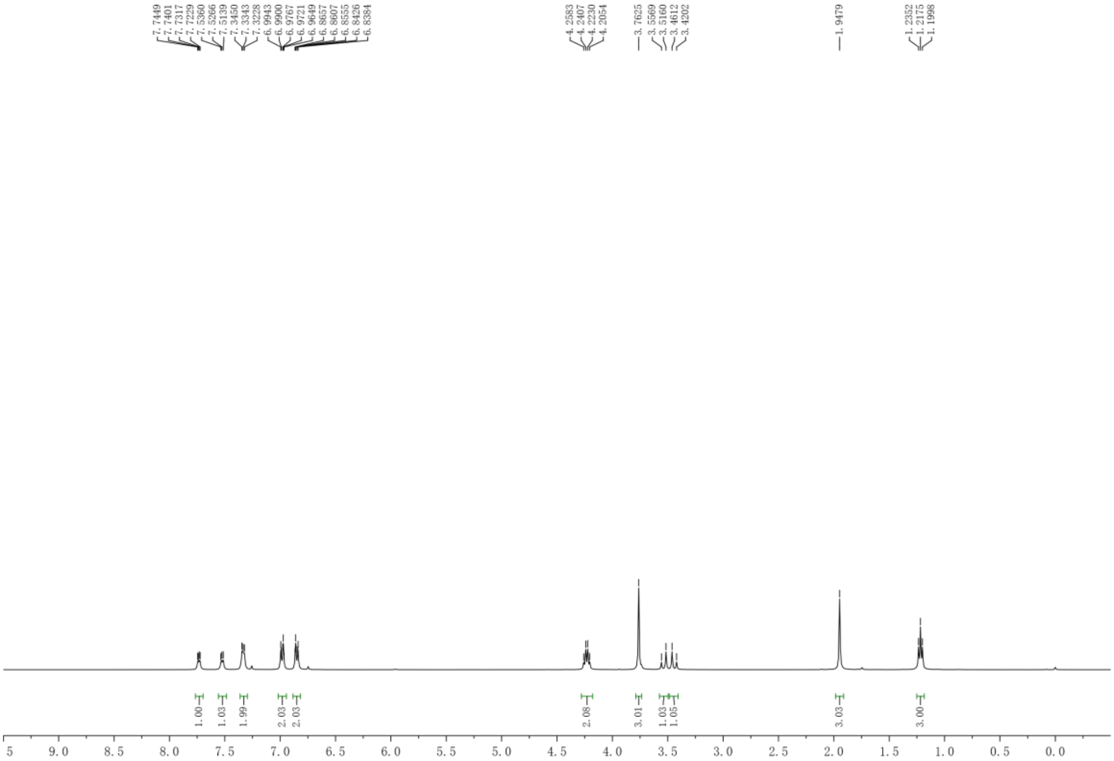

^{13}**C NMR** spectrum of **12**

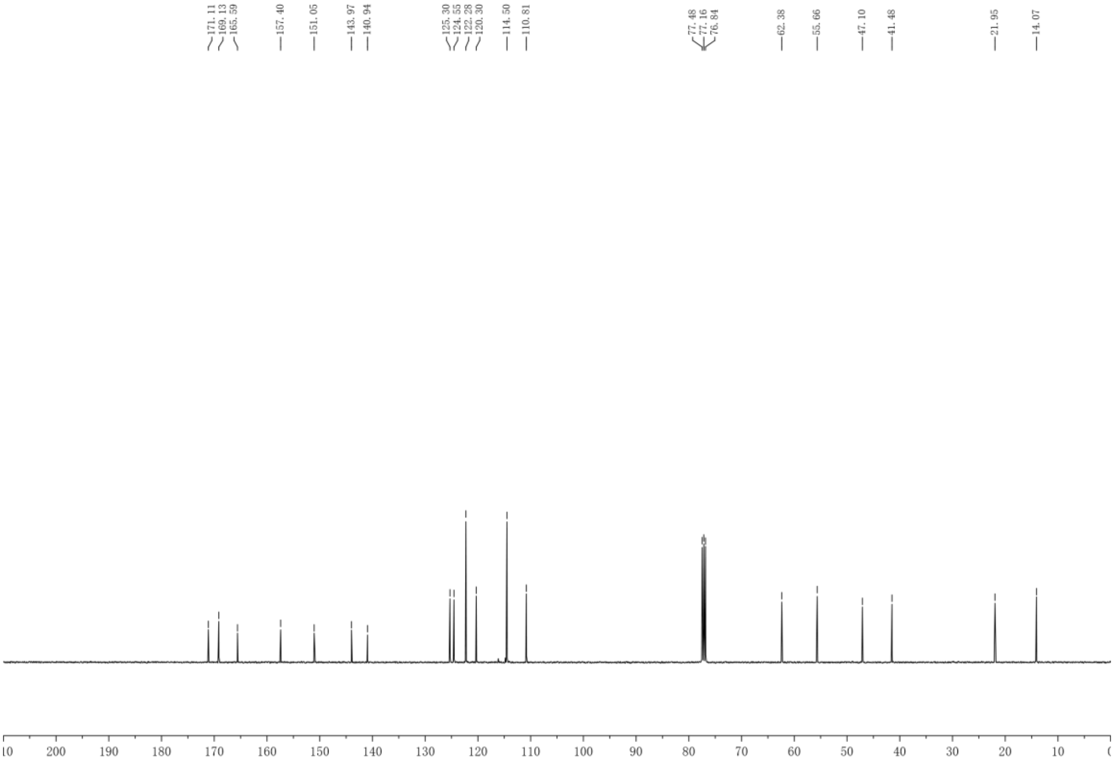

^{1}H NMR spectrum of **13**

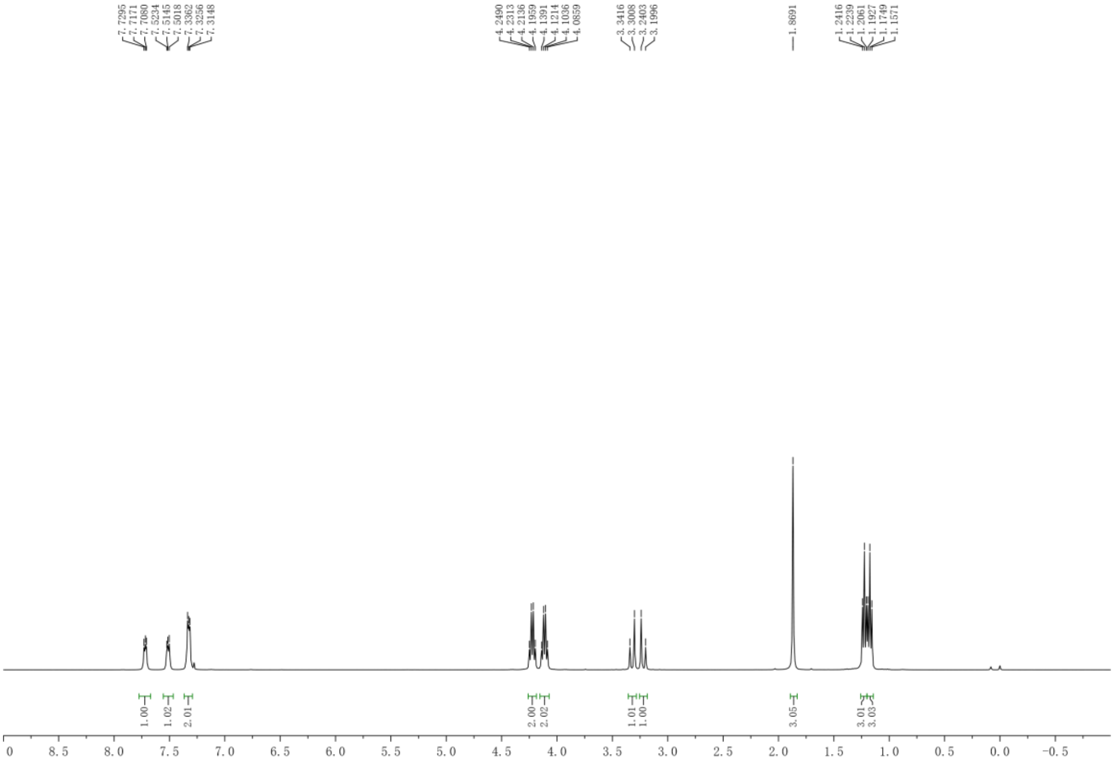

^{13}C NMR spectrum of **13**

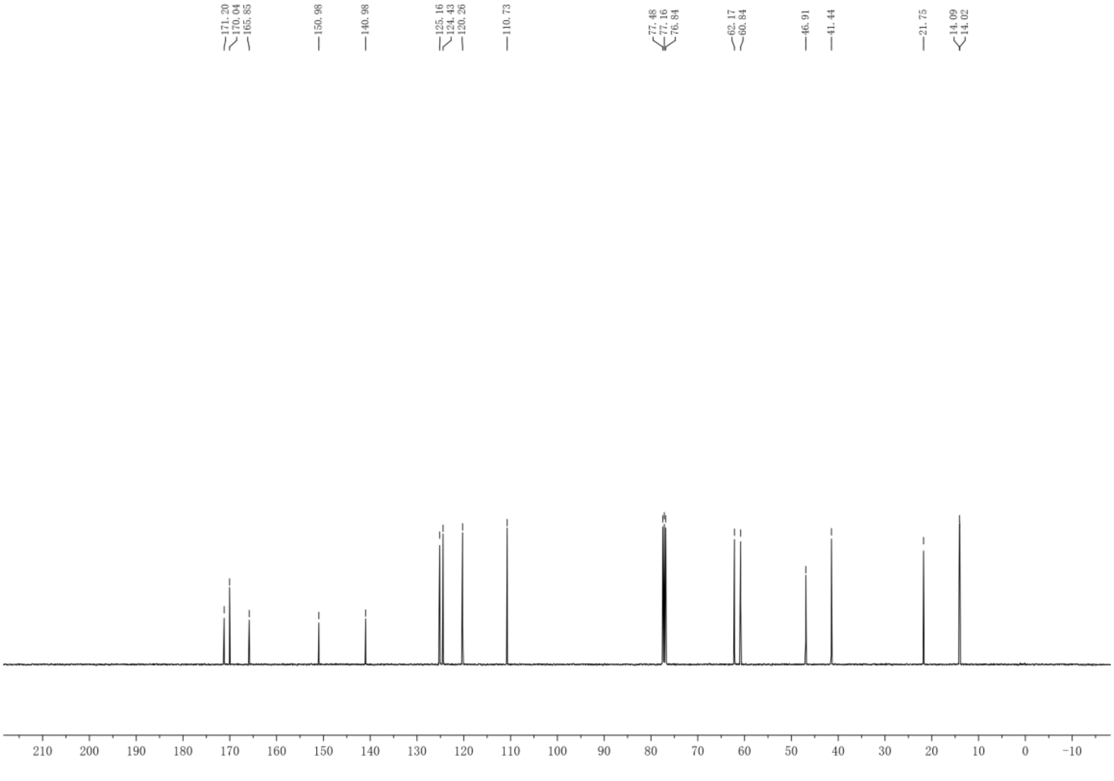

^{1}H NMR spectrum of **14**

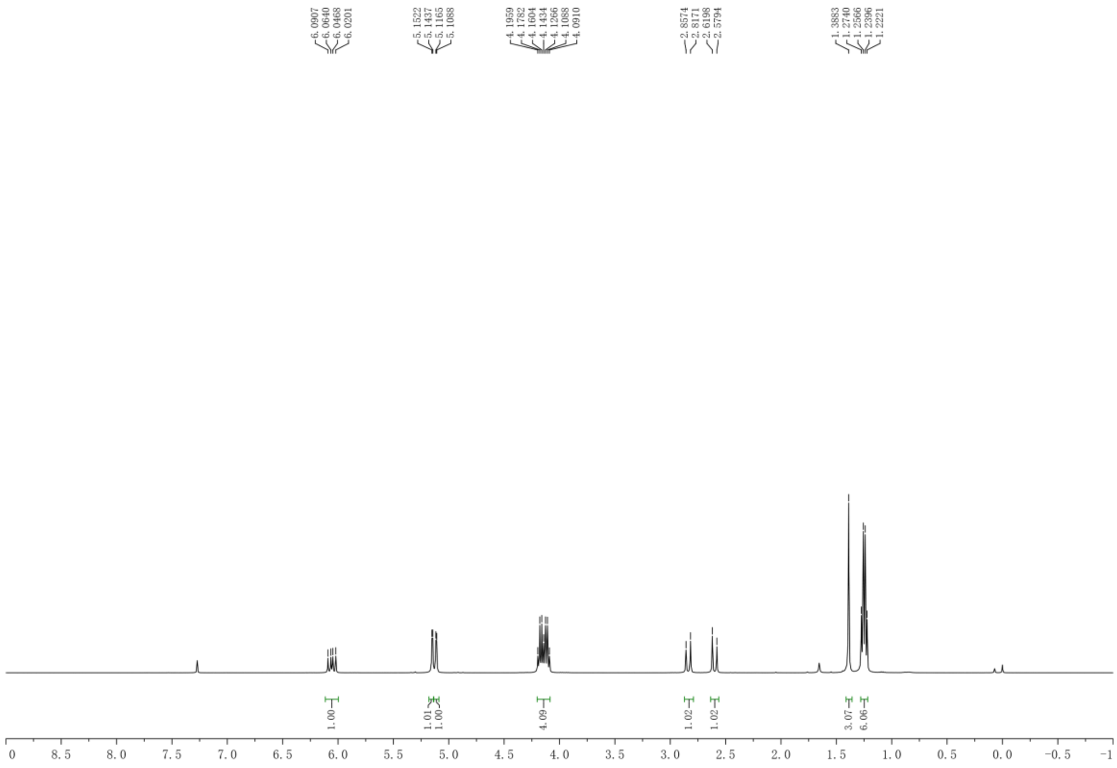

^{13}C NMR spectrum of **14**

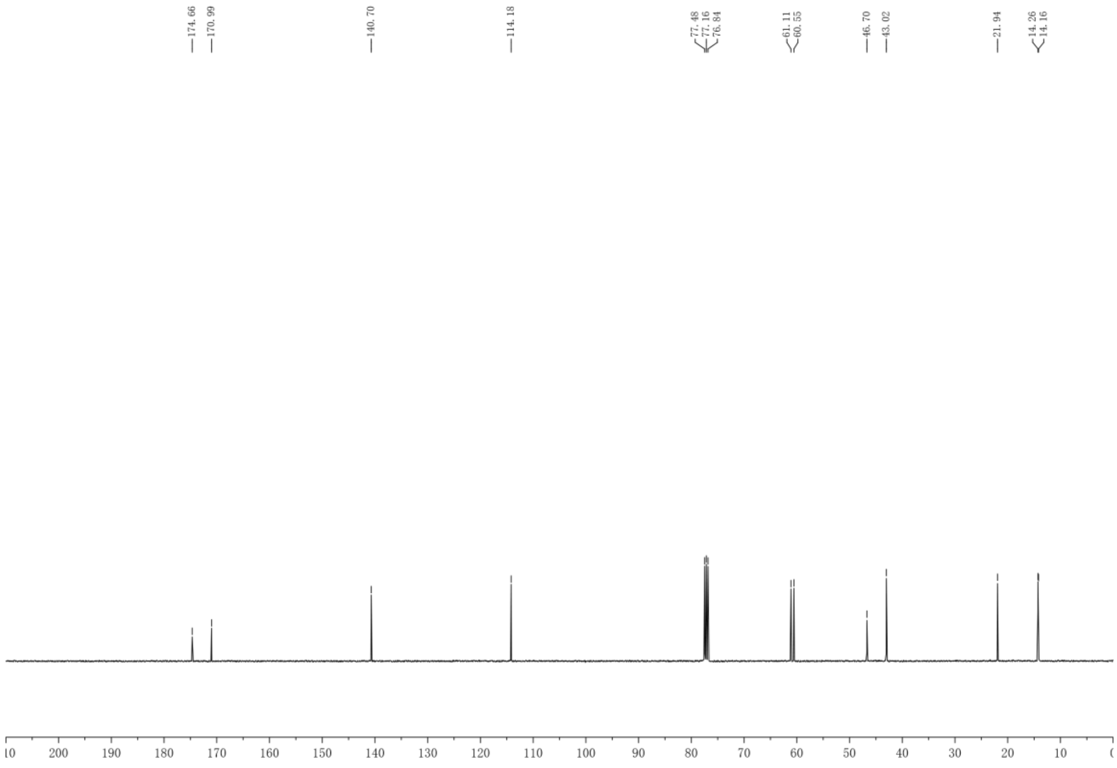

^{1}**H NMR** spectrum of **15**

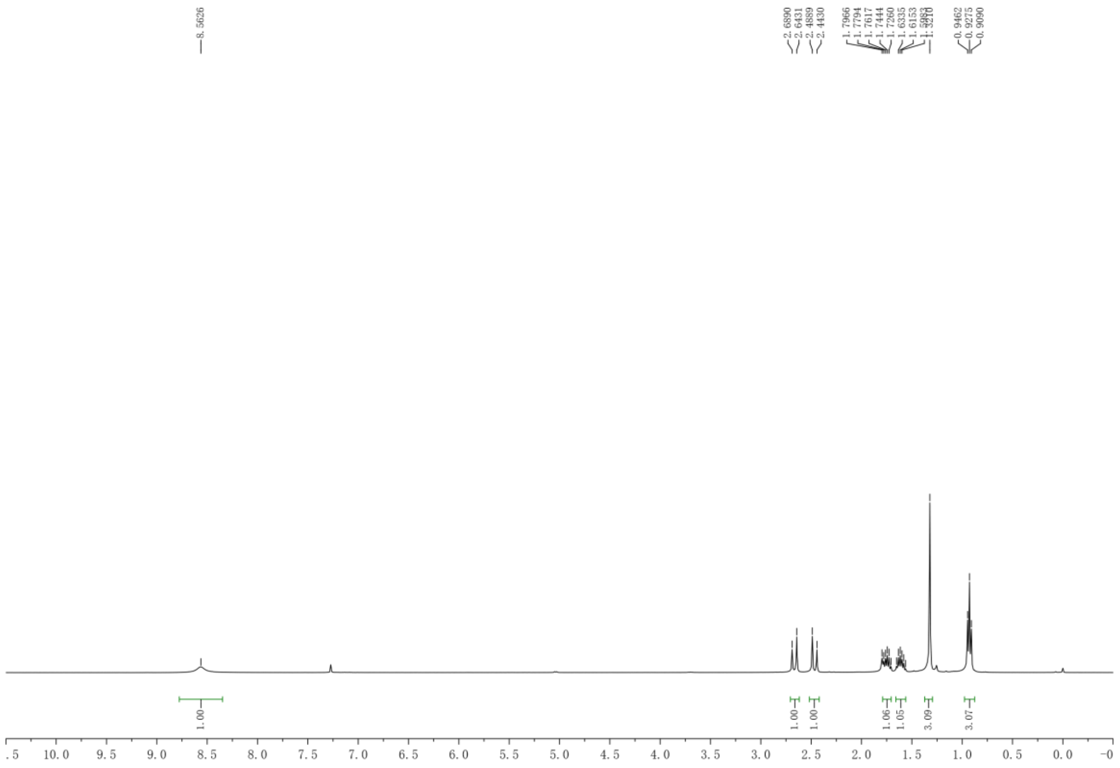

^{13}**C NMR** spectrum of **15**

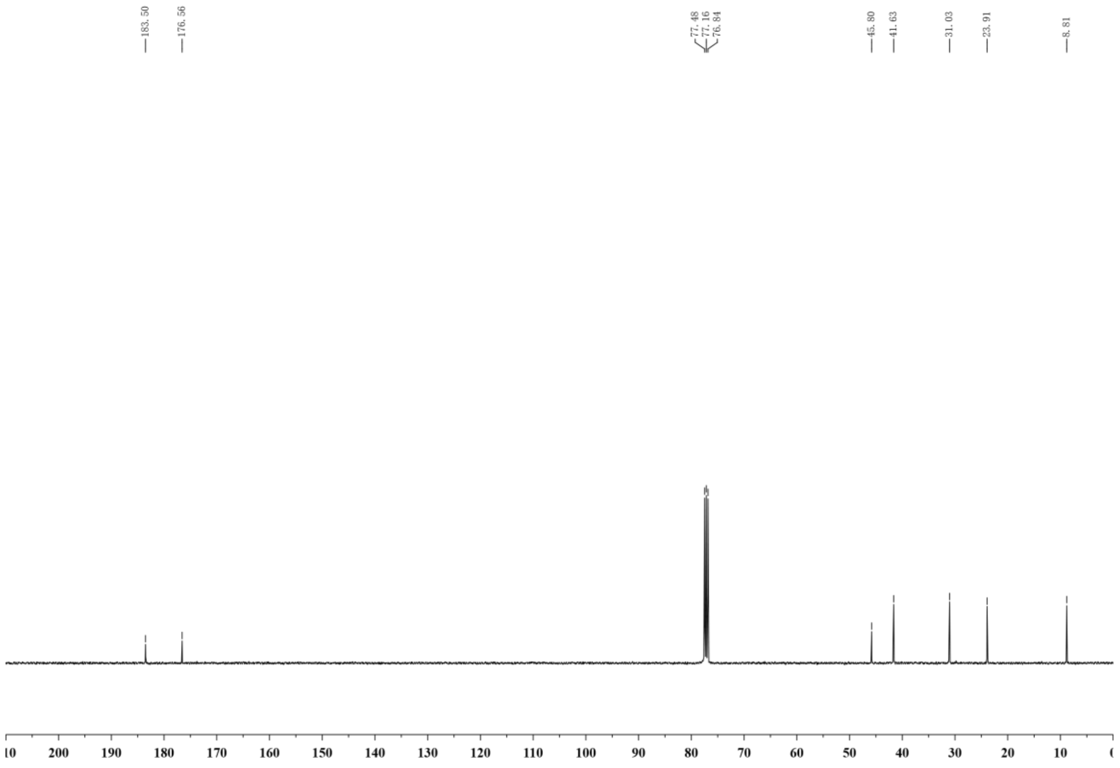

^{13}C NMR spectrum of **17**

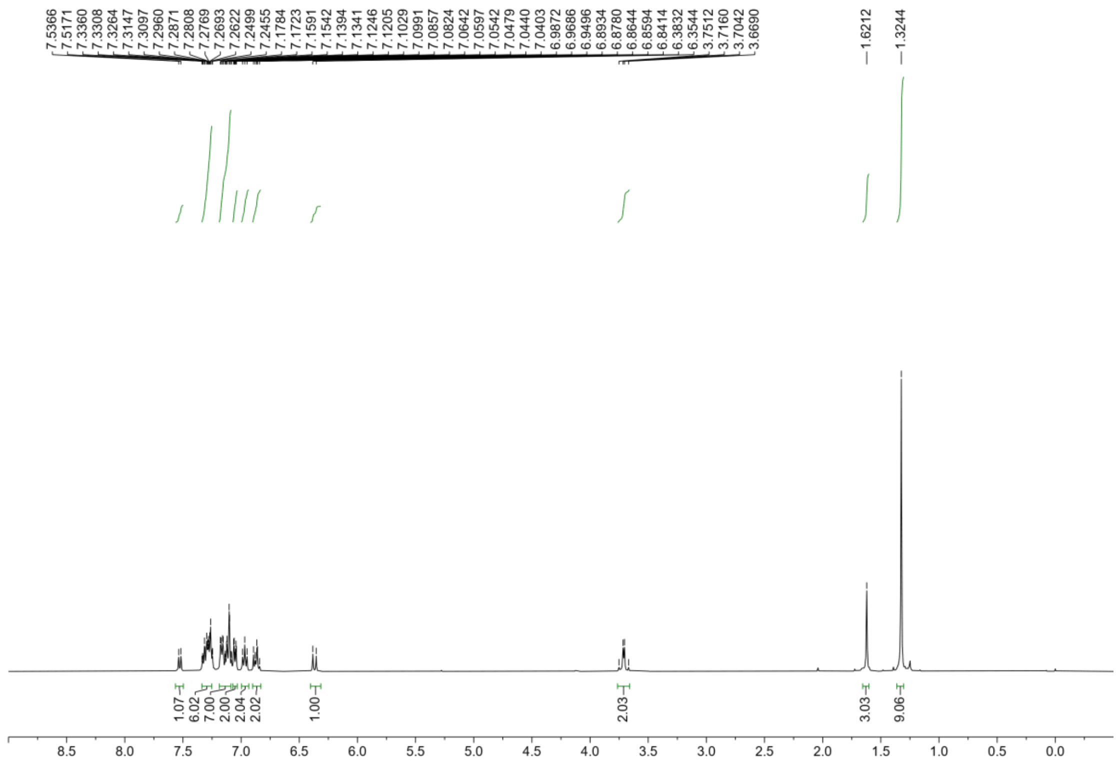

^{13}C NMR spectrum of **17**

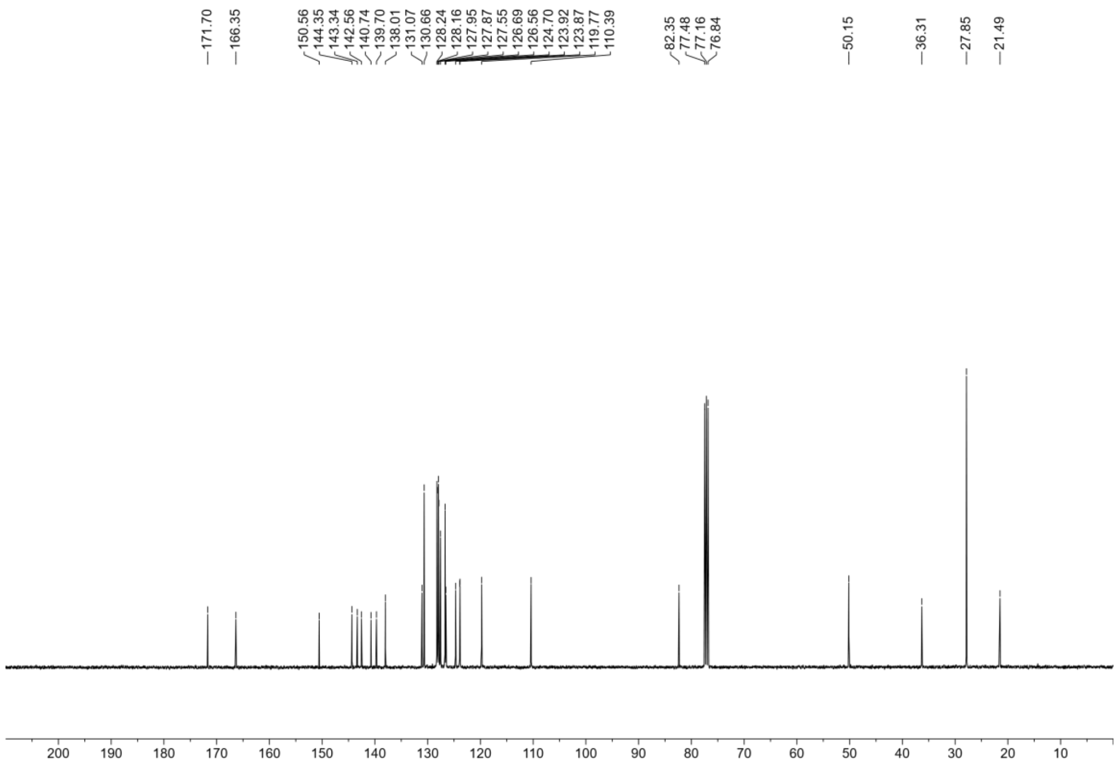

10. HPLC traces

rac-**3a**

SAMPLE INFORMATION

Sample Name:	zjy-12-182-10%-IC-rac	Acquired By:	System
Sample Type:	Unknown	Sample Set Name:	0
Vial:	49	Acq. Method Set:	10%quanbo
Injection #:	1	Processing Method:	54165463
Injection Volume:	10.00 ul	Channel Name:	320.0nm
Run Time:	35.0 Minutes	Proc. Chnl. Descr.:	2998 PDA 320.0 nm (2998
Date Acquired:	10/17/2023 9:36:18 AM CST		
Date Processed:	10/18/2023 9:13:01 AM CST		

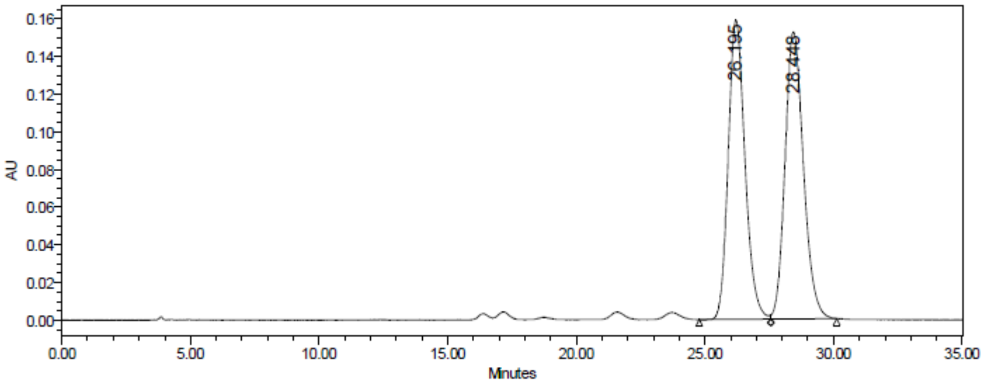

	RT	Area	% Area	Height
1	26.195	7250490	49.08	159122
2	28.448	7522045	50.92	152488

asy-**3a**

SAMPLE INFORMATION

Sample Name:	zjy-12-182-10%-IC-asy	Acquired By:	System
Sample Type:	Unknown	Sample Set Name:	0
Vial:	50	Acq. Method Set:	10%quanbo
Injection #:	1	Processing Method:	ZJY 5 199 ASY
Injection Volume:	10.00 ul	Channel Name:	320.0nm
Run Time:	35.0 Minutes	Proc. Chnl. Descr.:	2998 PDA 320.0 nm (2998
Date Acquired:	10/17/2023 10:12:15 AM CST		
Date Processed:	10/18/2023 9:11:36 AM CST		

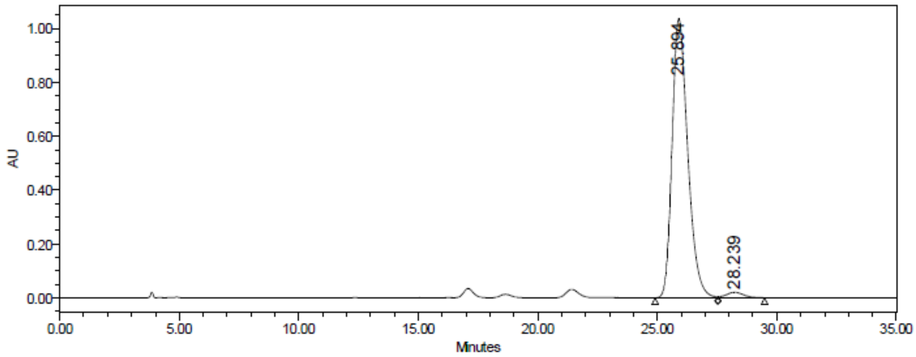

	RT	Area	% Area	Height
1	25.894	47177531	97.82	1035909
2	28.239	1052653	2.18	20817

rac-**3b**

S A M P L E   I N F O R M A T I O N

Sample Name:	zjy-12-176-10%-IE-rac	Acquired By:	System
Sample Type:	Unknown	Sample Set Name:	
Vial:	57	Acq. Method Set:	10%quanbo
Injection #:	1	Processing Method:	1354685746
Injection Volume:	10.00 ul	Channel Name:	270.0nm
Run Time:	60.0 Minutes	Proc. Chnl. Descr.:	2998 PDA 270.0 nm (2998
Date Acquired:	10/7/2023 7:01:05 PM CST		
Date Processed:	10/13/2023 9:53:29 PM CST		

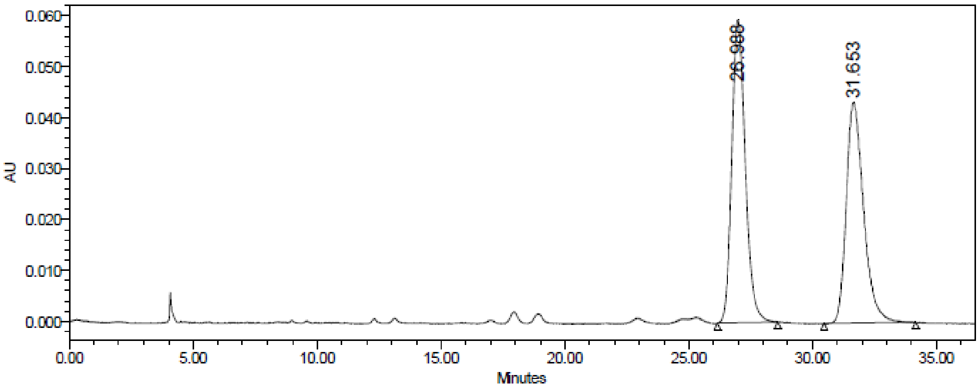

	RT	Area	% Area	Height
1	26.988	2249053	51.44	59412
2	31.653	2123104	48.56	43307

asy-**3b**

S A M P L E   I N F O R M A T I O N

Sample Name:	zjy-12-175-10%-IE-asy	Acquired By:	System
Sample Type:	Unknown	Sample Set Name:	0
Vial:	60	Acq. Method Set:	10%quanbo
Injection #:	1	Processing Method:	54165463
Injection Volume:	10.00 ul	Channel Name:	270.0nm
Run Time:	36.0 Minutes	Proc. Chnl. Descr.:	2998 PDA 270.0 nm (2998
Date Acquired:	10/7/2023 7:45:02 PM CST		
Date Processed:	10/13/2023 9:51:56 PM CST		

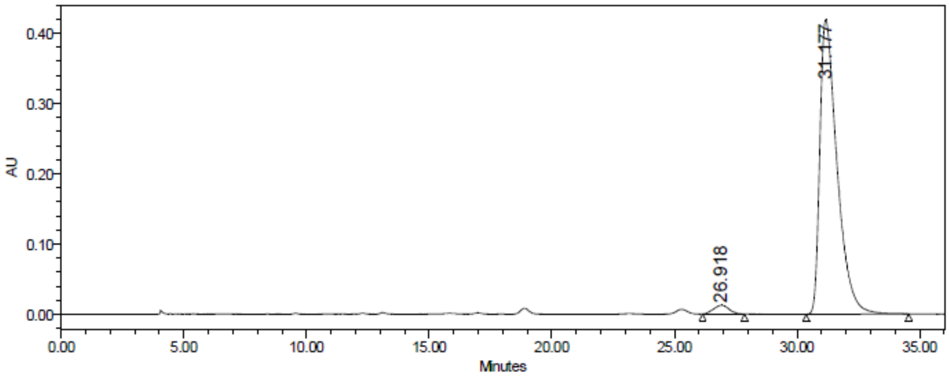

	RT	Area	% Area	Height
1	26.918	551808	2.62	12575
2	31.177	20484156	97.38	418957

rac-**3c**

SAMPLE INFORMATION

Sample Name:	zjy-12-181-10%-AD-H-rac	Acquired By:	System
Sample Type:	Unknown	Sample Set Name:	
Vial:	67	Acq. Method Set:	10%quanbo
Injection #:	1	Processing Method:	1354685746
Injection Volume:	10.00 ul	Channel Name:	259.6nm
Run Time:	60.0 Minutes	Proc. Chnl. Descr.:	2998 PDA 259.6 nm (2998
Date Acquired:	6/6/2024 3:08:38 PM CST		
Date Processed:	6/6/2024 9:24:55 PM CST		

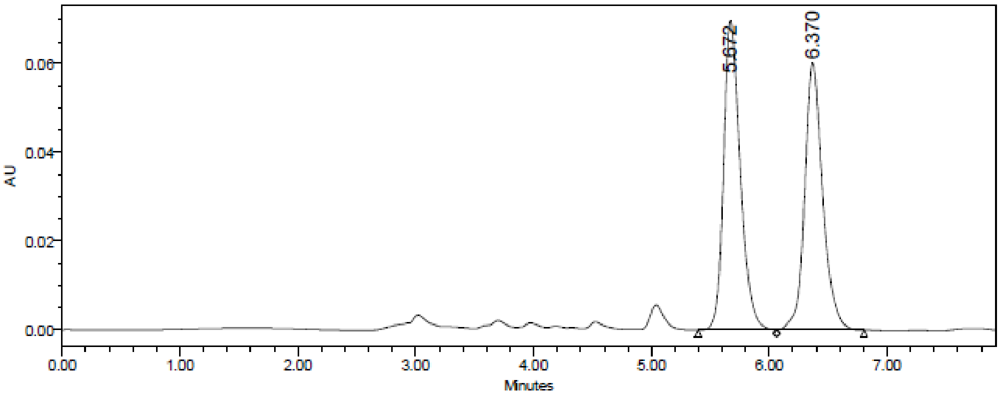

	RT	Area	% Area	Height
1	5.672	688687	51.70	69834
2	6.370	643367	48.30	60291

asy-**3c**

SAMPLE INFORMATION

Sample Name:	zjy-12-181-10%-AD-H-asy	Acquired By:	System
Sample Type:	Unknown	Sample Set Name:	
Vial:	68	Acq. Method Set:	10%quanbo
Injection #:	1	Processing Method:	1231
Injection Volume:	10.00 ul	Channel Name:	248.0nm
Run Time:	60.0 Minutes	Proc. Chnl. Descr.:	2998 PDA 248.0 nm (2998
Date Acquired:	6/6/2024 3:17:50 PM CST		
Date Processed:	6/6/2024 9:23:24 PM CST		

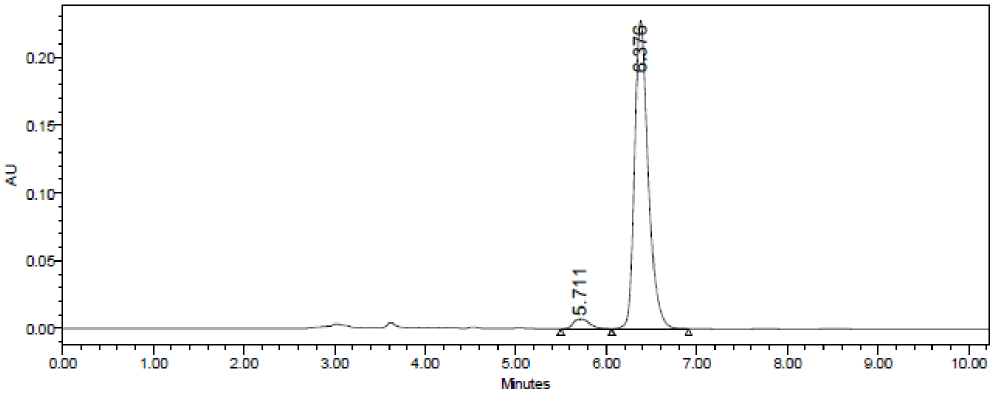

	RT	Area	% Area	Height
1	5.711	88400	3.59	7064
2	6.376	2371007	96.41	227076

rac-**3d**

SAMPLE INFORMATION

Sample Name:	zjy-9-336(337)-5%-IC-RAC	Acquired By:	System
Sample Type:	Unknown	Sample Set Name:	0
Vial:	60	Acq. Method Set:	5%quanbo
Injection #:	1	Processing Method:	1231
Injection Volume:	10.00 ul	Channel Name:	254.0nm
Run Time:	22.0 Minutes	Proc. Chnl. Descr.:	2998 PDA 254.0 nm (2998
Date Acquired:	10/27/2022 10:13:11 AM CST		
Date Processed:	12/31/2022 4:06:33 PM CST		

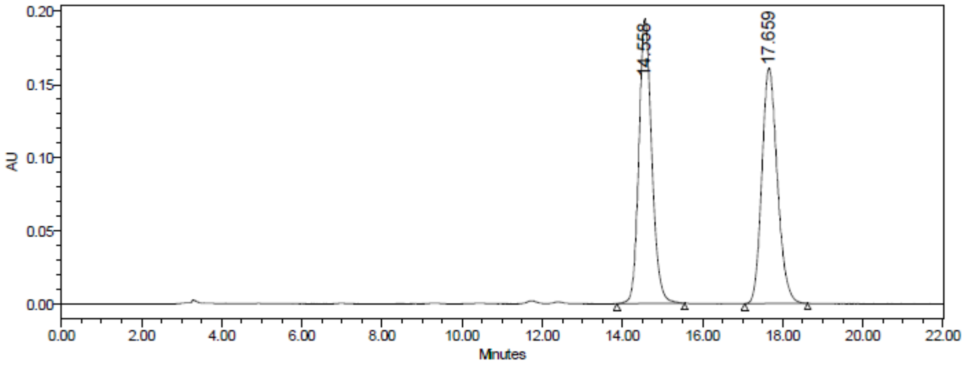

	RT	Area	% Area	Height
1	14.558	4348607	50.23	194431
2	17.659	4309564	49.77	160810

asy-**3d**

SAMPLE INFORMATION

Sample Name:	zjy-9-336-5%-IC-asy	Acquired By:	System
Sample Type:	Unknown	Sample Set Name:	0
Vial:	59	Acq. Method Set:	5%quanbo
Injection #:	1	Processing Method:	0
Injection Volume:	10.00 ul	Channel Name:	254.0nm
Run Time:	22.0 Minutes	Proc. Chnl. Descr.:	2998 PDA 254.0 nm (2998
Date Acquired:	10/27/2022 9:50:16 AM CST		
Date Processed:	12/31/2022 4:05:34 PM CST		

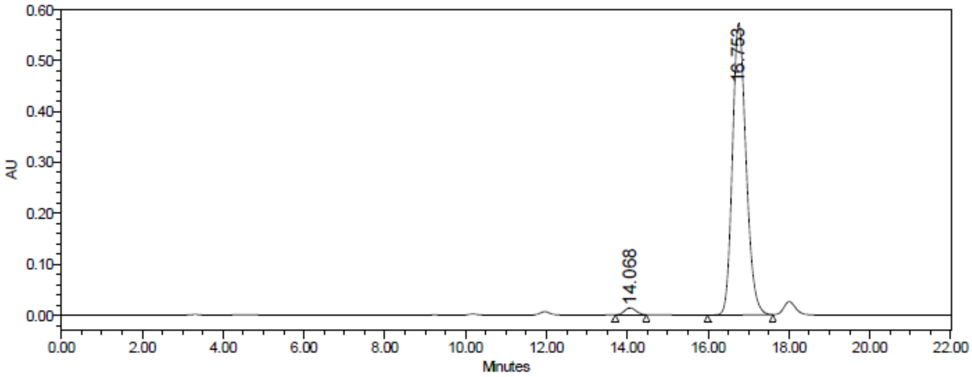

	RT	Area	% Area	Height
1	14.068	260793	1.90	13459
2	16.753	13484428	98.10	571074

rac-**3e**

SAMPLE INFORMATION

Sample Name:	zjy-12-196-10%-IC-rac	Acquired By:	System
Sample Type:	Unknown	Sample Set Name:	0
Vial:	51	Acq. Method Set:	10%quanbo
Injection #:	1	Processing Method:	54165463
Injection Volume:	10.00 ul	Channel Name:	240.0nm
Run Time:	22.0 Minutes	Proc. Chnl. Descr.:	2998 PDA 240.0 nm (2998
Date Acquired:	10/16/2023 9:40:32 AM CST		
Date Processed:	10/16/2023 9:33:43 PM CST		

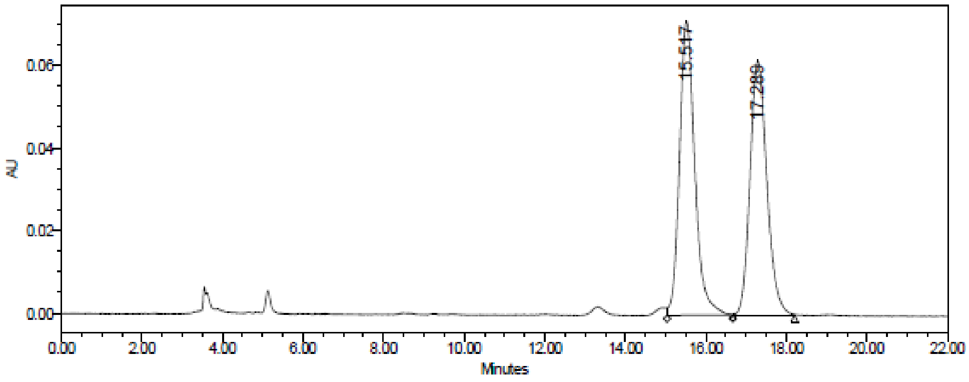

	RT	Area	% Area	Height
1	15.517	1879138	51.23	71159
2	17.289	1788834	48.77	61531

asy-**3e**

SAMPLE INFORMATION

Sample Name:	zjy-12-196-10%-IC-rac	Acquired By:	System
Sample Type:	Unknown	Sample Set Name:	0
Vial:	52	Acq. Method Set:	10%quanbo
Injection #:	1	Processing Method:	ZJY 5 184 2
Injection Volume:	10.00 ul	Channel Name:	254.0nm
Run Time:	22.0 Minutes	Proc. Chnl. Descr.:	2998 PDA 254.0 nm (2998
Date Acquired:	10/16/2023 10:03:26 AM CST		
Date Processed:	10/16/2023 10:28:27 AM CST		

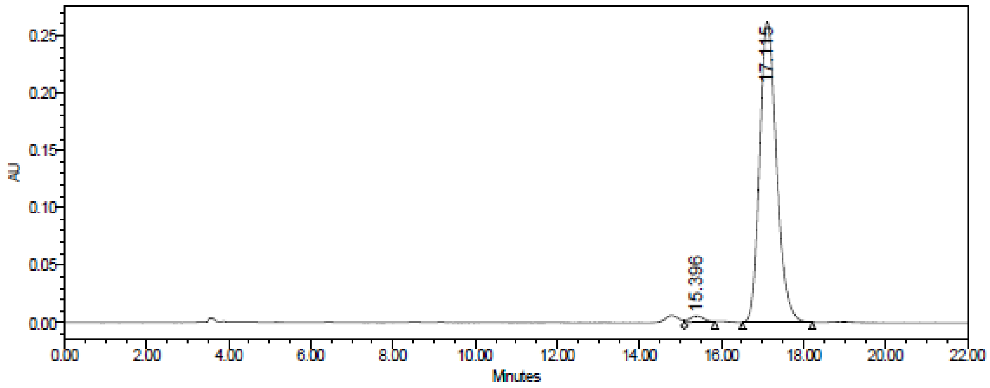

	RT	Area	% Area	Height
1	15.396	121473	1.59	5397
2	17.115	7502515	98.41	261387

rac-**3f**

SAMPLE INFORMATION

Sample Name:	zjy-12-195-10%-IC-asy	Acquired By:	System
Sample Type:	Unknown	Sample Set Name:	0
Vial:	52	Acq. Method Set:	10%quanbo
Injection #:	1	Processing Method:	54165463
Injection Volume:	10.00 ul	Channel Name:	240.0nm
Run Time:	15.0 Minutes	Proc. Chnl. Descr.:	2998 PDA 240.0 nm (2998
Date Acquired:	10/13/2023 8:26:50 PM CST		
Date Processed:	10/13/2023 8:59:41 PM CST		

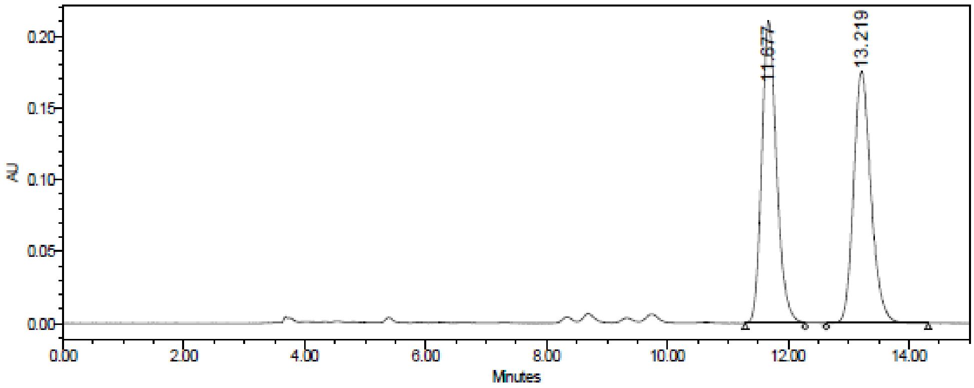

	RT	Area	% Area	Height
1	11.677	3638511	50.91	210178
2	13.219	3508799	49.09	175019

asy-**3f**

SAMPLE INFORMATION

Sample Name:	zjy-12-196-10%-IC-rac	Acquired By:	System
Sample Type:	Unknown	Sample Set Name:	0
Vial:	53	Acq. Method Set:	10%quanbo
Injection #:	1	Processing Method:	54165463
Injection Volume:	10.00 ul	Channel Name:	240.0nm
Run Time:	26.0 Minutes	Proc. Chnl. Descr.:	2998 PDA 240.0 nm (2998
Date Acquired:	10/13/2023 8:42:31 PM CST		
Date Processed:	10/13/2023 9:00:55 PM CST		

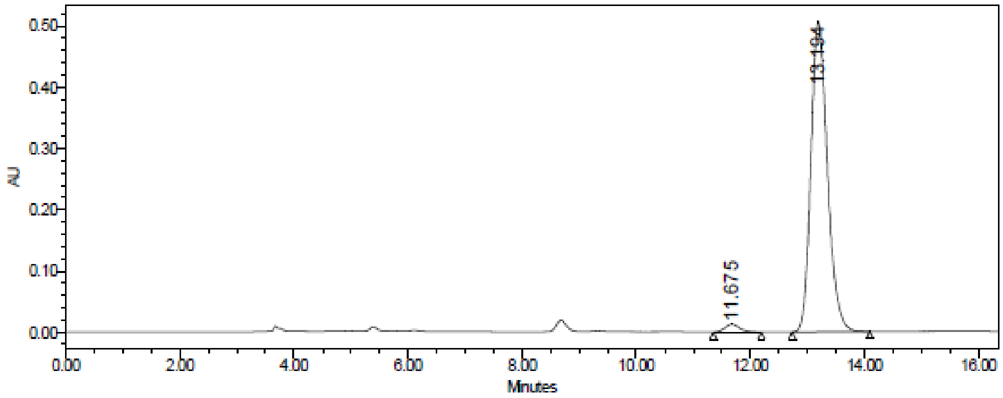

	RT	Area	% Area	Height
1	11.675	223545	2.16	12952
2	13.194	10144760	97.84	506880

rac-**3g**

SAMPLE INFORMATION

Sample Name:	zjy-12-201-10%-IG-RAC	Acquired By:	System
Sample Type:	Unknown	Sample Set Name:	
Vial:	66	Acq. Method Set:	10%quanbo
Injection #:	1	Processing Method:	1041
Injection Volume:	10.00 ul	Channel Name:	300.0nm
Run Time:	60.0 Minutes	Proc. Chnl. Descr.:	2998 PDA 300.0 nm (2998
Date Acquired:	6/6/2024 10:42:15 AM CST		
Date Processed:	6/6/2024 11:07:48 AM CST		

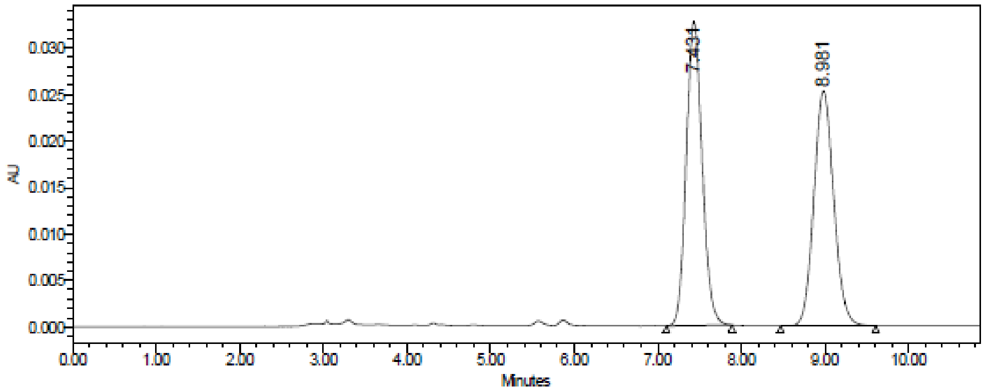

	RT	Area	% Area	Height
1	7.431	433315	50.94	32714
2	8.981	417380	49.06	25275

asy-**3g**

SAMPLE INFORMATION

Sample Name:	zjy-12-201-10%-IG-asy	Acquired By:	System
Sample Type:	Unknown	Sample Set Name:	
Vial:	68	Acq. Method Set:	10%quanbo
Injection #:	1	Processing Method:	1041
Injection Volume:	10.00 ul	Channel Name:	300.0nm
Run Time:	60.0 Minutes	Proc. Chnl. Descr.:	2998 PDA 300.0 nm (2998
Date Acquired:	6/6/2024 10:54:33 AM CST		
Date Processed:	6/6/2024 11:09:10 AM CST		

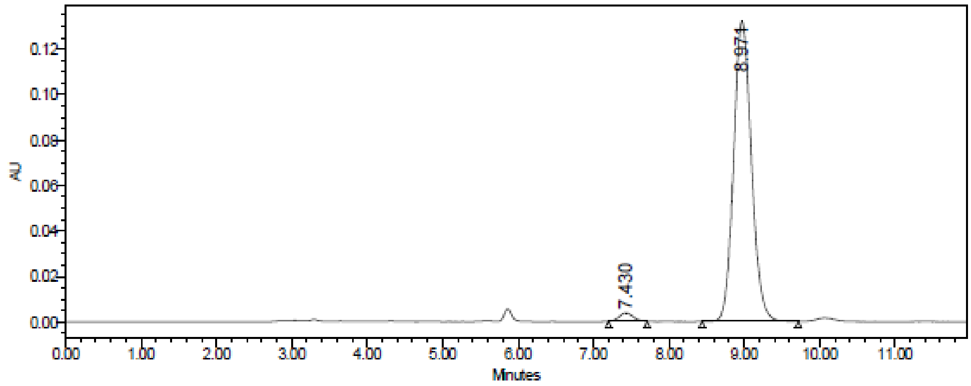

	RT	Area	% Area	Height
1	7.430	48478	2.18	3801
2	8.971	2172280	97.82	132056

rac-**3h**

SAMPLE INFORMATION

Sample Name:	zjy-12-199-10%-IC-rac	Acquired By:	System
Sample Type:	Unknown	Sample Set Name:	
Vial:	50	Acq. Method Set:	10%quanbo
Injection #:	1	Processing Method:	54165463
Injection Volume:	5.00 ul	Channel Name:	254.0nm
Run Time:	60.0 Minutes	Proc. Chnl. Descr.:	2998 PDA 254.0 nm (2998
Date Acquired:	10/13/2023 7:06:02 PM CST		
Date Processed:	10/14/2023 8:49:14 PM CST		

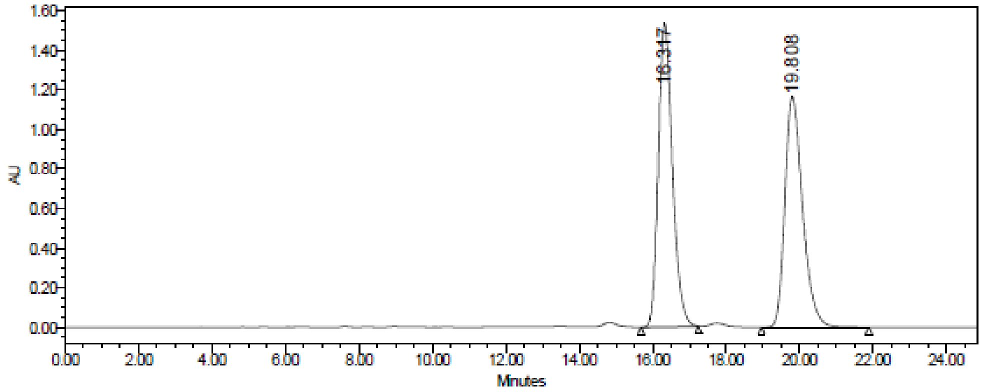

	RT	Area	% Area	Height
1	16.317	40498807	50.97	1535697
2	19.808	38955796	49.03	1164517

asy-**3h**

SAMPLE INFORMATION

Sample Name:	zjy-12-202-10%-IC-asy	Acquired By:	System
Sample Type:	Unknown	Sample Set Name:	0
Vial:	60	Acq. Method Set:	10%quanbo
Injection #:	1	Processing Method:	1354685746
Injection Volume:	10.00 ul	Channel Name:	254.0nm
Run Time:	24.0 Minutes	Proc. Chnl. Descr.:	2998 PDA 254.0 nm (2998
Date Acquired:	10/13/2023 11:18:12 PM CST		
Date Processed:	10/14/2023 8:48:06 PM CST		

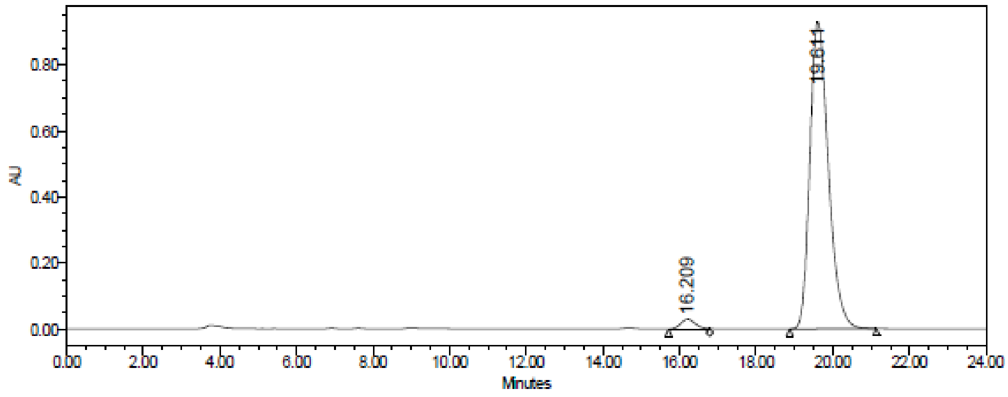

	RT	Area	% Area	Height
1	16.209	817085	2.58	30794
2	19.611	30848199	97.42	928449

rac-**3i**

SAMPLE INFORMATION

Sample Name:	zjy-12-200-10%-IC-rac	Acquired By:	System
Sample Type:	Unknown	Sample Set Name:	
Vial:	51	Acq. Method Set:	10%quanbo
Injection #:	1	Processing Method:	1354685746
Injection Volume:	5.00 ul	Channel Name:	300.0nm
Run Time:	60.0 Minutes	Proc. Chnl. Descr.:	2998 PDA 300.0 nm (2998
Date Acquired:	10/13/2023 7:31:41 PM CST		
Date Processed:	10/14/2023 8:46:59 PM CST		

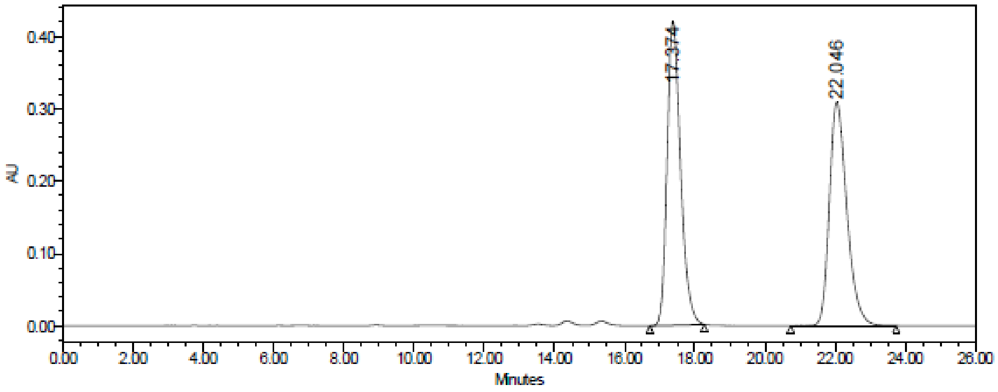

	RT	Area	% Area	Height
1	17.374	11196333	50.98	419657
2	22.046	10764549	49.02	309143

asy-**3i**

SAMPLE INFORMATION

Sample Name:	zjy-12-203-10%-IC-asy	Acquired By:	System
Sample Type:	Unknown	Sample Set Name:	0
Vial:	61	Acq. Method Set:	10%quanbo
Injection #:	1	Processing Method:	1354685746
Injection Volume:	10.00 ul	Channel Name:	300.0nm
Run Time:	26.0 Minutes	Proc. Chnl. Descr.:	2998 PDA 300.0 nm (2998
Date Acquired:	10/13/2023 11:42:53 PM CST		
Date Processed:	10/14/2023 8:44:51 PM CST		

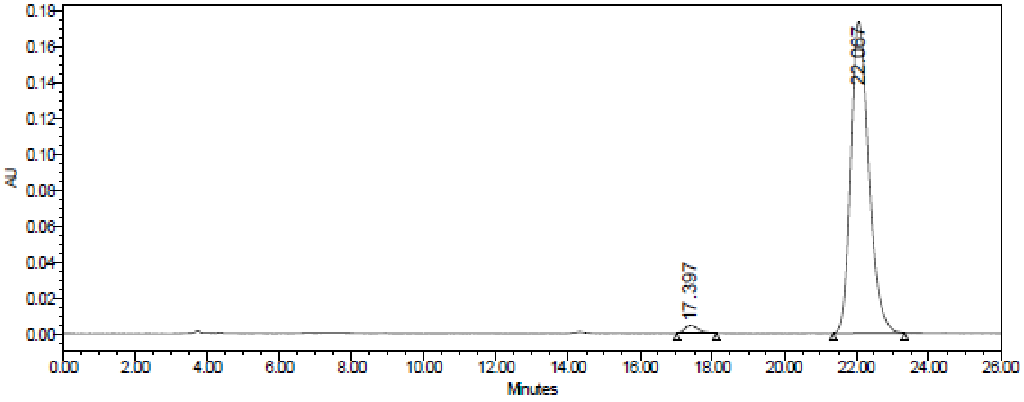

	RT	Area	% Area	Height
1	17.397	122574	1.98	4459
2	22.067	6076804	98.02	173817

rac-**3j**

SAMPLE INFORMATION

Sample Name:	zjy-12-197-10%-IC-rac	Acquired By:	System
Sample Type:	Unknown	Sample Set Name:	0
Vial:	56	Acq. Method Set:	10%quanbo
Injection #:	1	Processing Method:	54165463
Injection Volume:	10.00 ul	Channel Name:	240.0nm
Run Time:	30.0 Minutes	Proc. Chnl. Descr.:	2998 PDA 240.0 nm (2998
Date Acquired:	10/13/2023 9:49:26 PM CST		
Date Processed:	10/14/2023 8:52:57 PM CST		

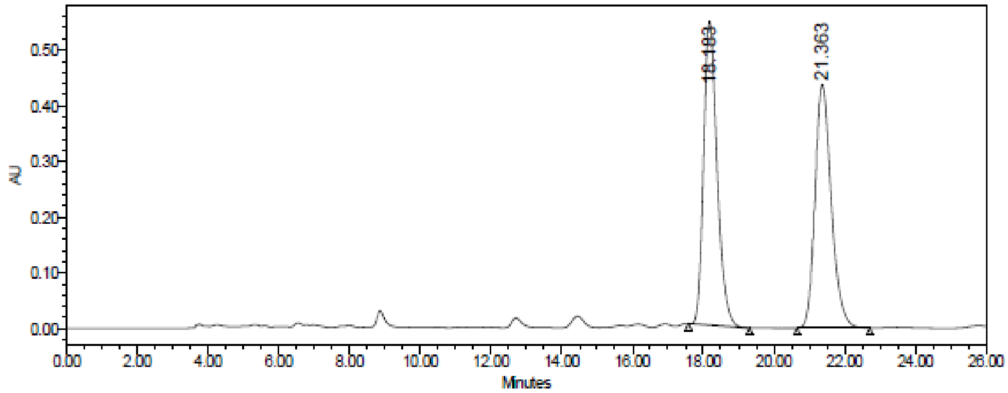

	RT	Area	% Area	Height
1	18.183	14503001	50.92	544534
2	21.363	13980025	49.08	437412

asy-**3j**

SAMPLE INFORMATION

Sample Name:	zjy-12-197-10%-IC-asy	Acquired By:	System
Sample Type:	Unknown	Sample Set Name:	0
Vial:	57	Acq. Method Set:	10%quanbo
Injection #:	1	Processing Method:	1231
Injection Volume:	10.00 ul	Channel Name:	240.0nm
Run Time:	26.0 Minutes	Proc. Chnl. Descr.:	2998 PDA 240.0 nm (2998
Date Acquired:	10/13/2023 10:20:07 PM CST		
Date Processed:	10/14/2023 8:53:35 PM CST		

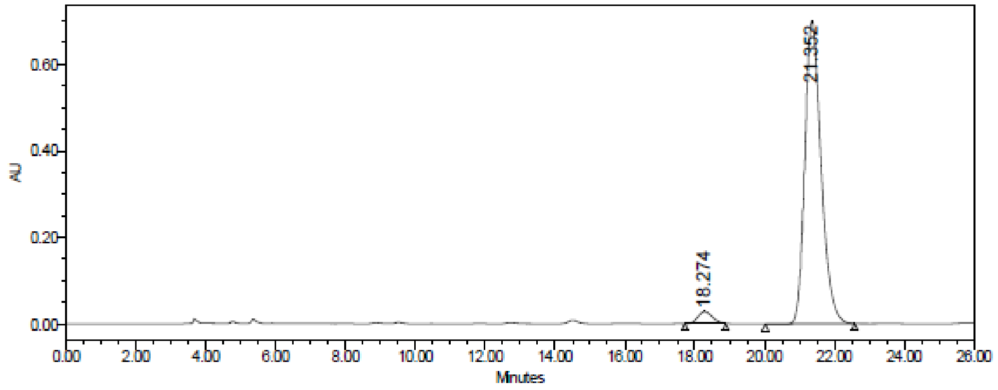

	RT	Area	% Area	Height
1	18.274	808316	3.48	27895
2	21.352	22423349	96.52	699440

rac-**3k**

SAMPLE INFORMATION

Sample Name:	zjy-12-222-5%-IC-rac	Acquired By:	System
Sample Type:	Unknown	Sample Set Name	
Vial:	57	Acq. Method Set:	5% quanbo
Injection #:	1	Processing Method	zjy 5 122 asy0
Injection Volume:	5.00 ul	Channel Name:	240.0nm
Run Time:	60.0 Minutes	Proc. Chnl. Descr.:	2998 PDA 240.0 nm (2998
Date Acquired:	10/20/2023 19:20:32 CST		
Date Processed:	6/3/2024 21:26:16 CST		

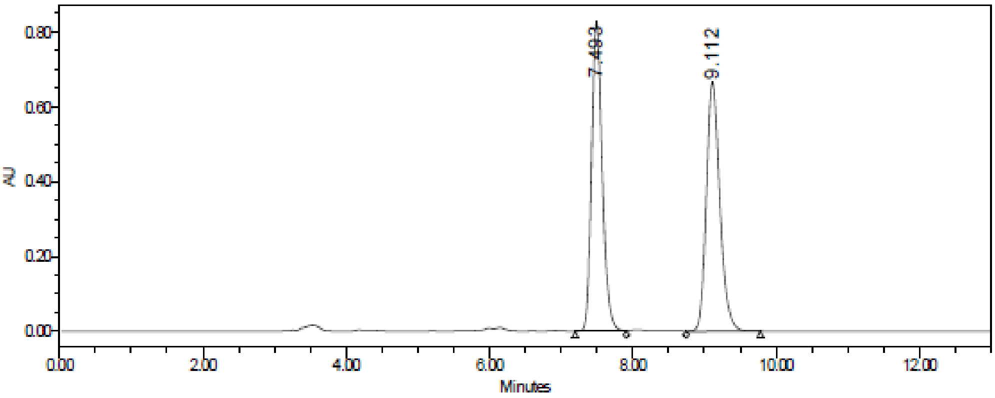

	RT	Area	% Area	Height
1	7.493	8591634	49.55	826110
2	9.112	8747078	50.45	663581

asy-**3k**

SAMPLE INFORMATION

Sample Name:	zjy-12-224-5%-IC-asy	Acquired By:	System
Sample Type:	Unknown	Sample Set Name	
Vial:	58	Acq. Method Set:	5% quanbo
Injection #:	1	Processing Method	zjy 5 122 asy0
Injection Volume:	10.00 ul	Channel Name:	240.0nm
Run Time:	60.0 Minutes	Proc. Chnl. Descr.:	2998 PDA 240.0 nm (2998
Date Acquired:	10/20/2023 20:41:20 CST		
Date Processed:	6/3/2024 21:24:17 CST		

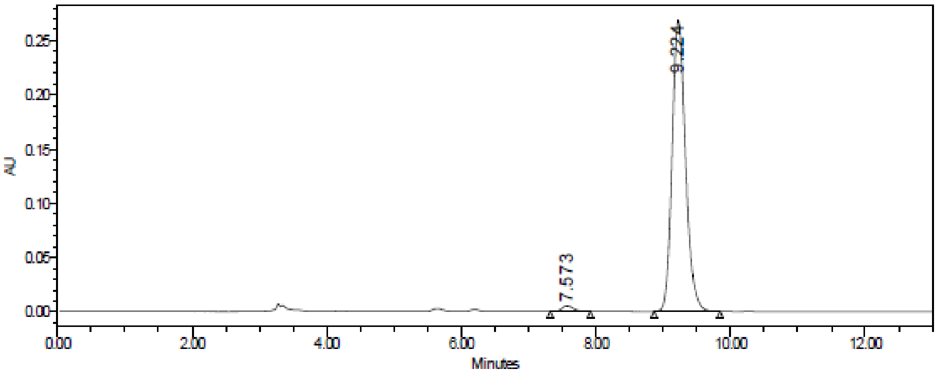

	RT	Area	% Area	Height
1	7.573	61899	1.63	5552
2	9.224	3732880	98.37	268499

rac-**3l**

SAMPLE INFORMATION

Sample Name:	zjy-12-150-10%-IC-rac	Acquired By:	System
Sample Type:	Unknown	Sample Set Name:	0
Vial:	49	Acq. Method Set:	10%quanbo
Injection #:	1	Processing Method:	ZJY 5 198
Injection Volume:	10.00 ul	Channel Name:	225.0nm
Run Time:	20.0 Minutes	Proc. Chnl. Descr.:	2998 PDA 225.0 nm (2998
Date Acquired:	10/16/2023 8:58:55 AM CST		
Date Processed:	10/16/2023 9:46:25 AM CST		

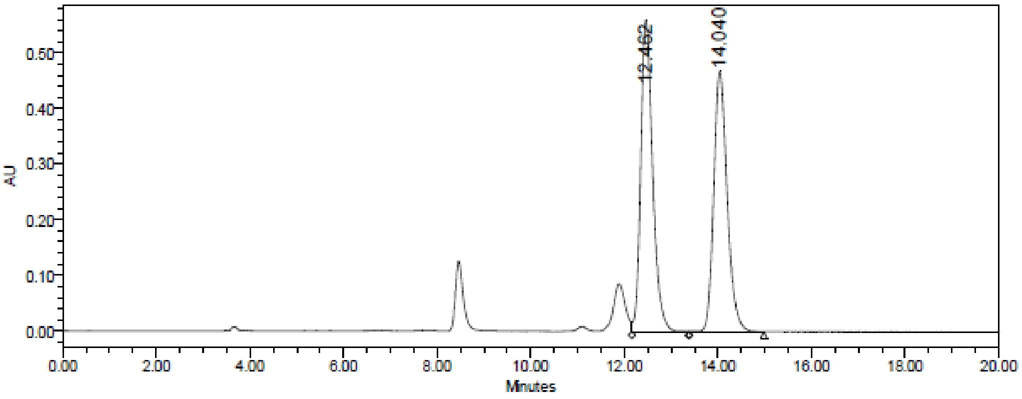

	RT	Area	% Area	Height
1	12.462	9890282	51.35	560599
2	14.040	9369868	48.65	468530

asy-**3l**

SAMPLE INFORMATION

Sample Name:	zjy-12-146-10%-IC-asy	Acquired By:	System
Sample Type:	Unknown	Sample Set Name:	0
Vial:	50	Acq. Method Set:	10%quanbo
Injection #:	1	Processing Method:	1354685746
Injection Volume:	10.00 ul	Channel Name:	225.0nm
Run Time:	20.0 Minutes	Proc. Chnl. Descr.:	2998 PDA 225.0 nm (2998
Date Acquired:	10/16/2023 9:19:37 AM CST		
Date Processed:	10/16/2023 9:44:01 AM CST		

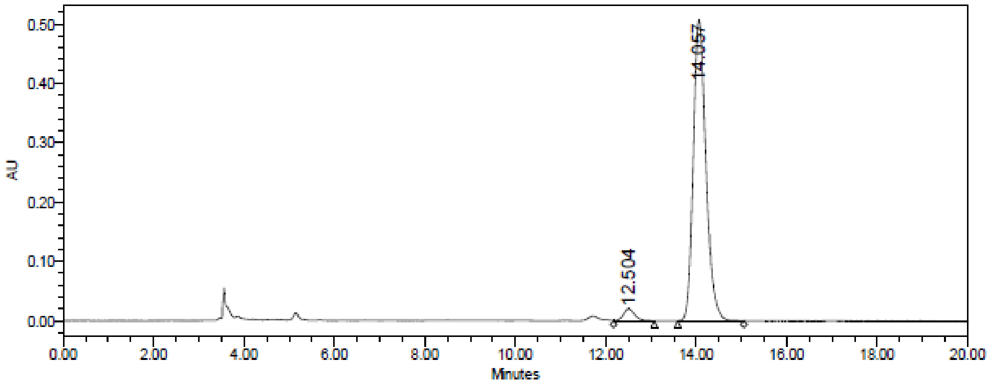

	RT	Area	% Area	Height
1	12.504	356658	3.46	20424
2	14.057	9936935	96.54	507645

rac-**3m**

SAMPLE INFORMATION

Sample Name:	zjy-15-161-10%-IE-rac	Acquired By:	System
Sample Type:	Unknown	Sample Set Name:	0
Vial:	62	Acq. Method Set:	10%quanbo
Injection #:	1	Processing Method:	54165463
Injection Volume:	10.00 ul	Channel Name:	254.0nm
Run Time:	10.0 Minutes	Proc. Chnl. Descr.:	2998 PDA 254.0 nm (2998
Date Acquired:	12/26/2024 7:22:51 PM CST		
Date Processed:	1/3/2025 10:44:24 PM CST		

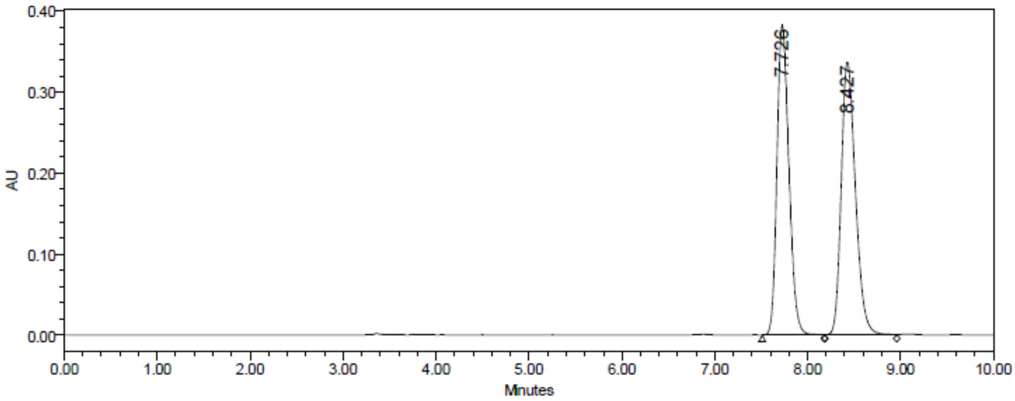

	RT	Area	% Area	Height
1	7.726	3423318	48.66	382246
2	8.427	3611156	51.34	336923

asy-**3m**

SAMPLE INFORMATION

Sample Name:	zjy-15-162-10%-IE-asy	Acquired By:	System
Sample Type:	Unknown	Sample Set Name:	0
Vial:	63	Acq. Method Set:	10%quanbo
Injection #:	1	Processing Method:	54165463
Injection Volume:	10.00 ul	Channel Name:	254.0nm
Run Time:	10.0 Minutes	Proc. Chnl. Descr.:	2998 PDA 254.0 nm (2998
Date Acquired:	12/26/2024 7:33:33 PM CST		
Date Processed:	1/3/2025 10:43:16 PM CST		

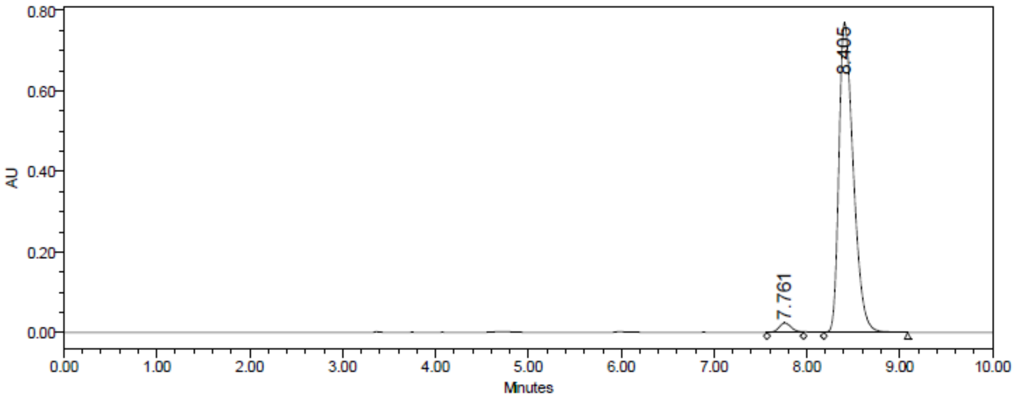

	RT	Area	% Area	Height
1	7.761	224914	2.63	23710
2	8.405	8333225	97.37	768999

rac-**3n**

SAMPLE INFORMATION

Sample Name:	zjy-13-227-5%-IE-rac	Acquired By:	System
Sample Type:	Unknown	Sample Set Name:	0
Vial:	110	Acq. Method Set:	15%quanbo
Injection #:	1	Processing Method:	1354685746
Injection Volume:	10.00 ul	Channel Name:	235.0nm
Run Time:	20.0 Minutes	Proc. Chnl. Descr.:	2998 PDA 235.0 nm (2998
Date Acquired:	5/8/2024 6:40:59 PM CST		
Date Processed:	6/4/2024 7:45:36 PM CST		

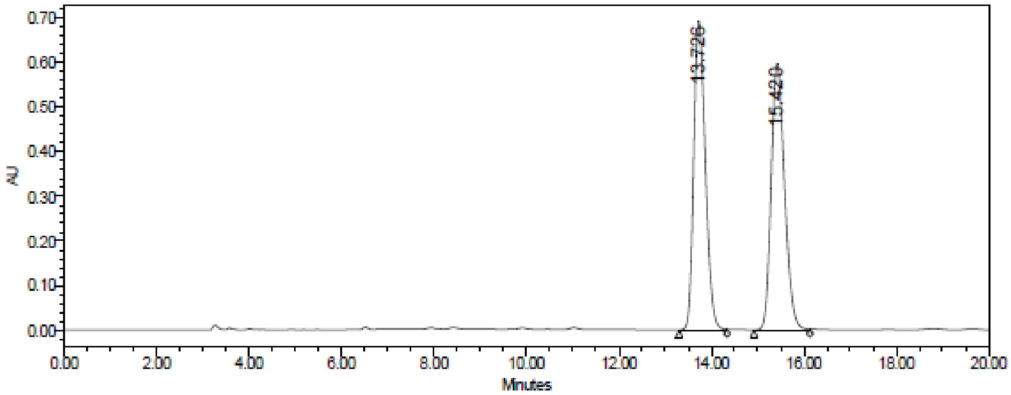

	RT	Area	% Area	Height
1	13.726	12072195	50.00	691615
2	15.420	12073888	50.00	596763

asy-**3n**

SAMPLE INFORMATION

Sample Name:	zjy-13-227-5%-IE-asy	Acquired By:	System
Sample Type:	Unknown	Sample Set Name:	0
Vial:	111	Acq. Method Set:	15%quanbo
Injection #:	1	Processing Method:	Default
Injection Volume:	10.00 ul	Channel Name:	235.0nm
Run Time:	20.0 Minutes	Proc. Chnl. Descr.:	2998 PDA 235.0 nm (2998
Date Acquired:	5/8/2024 7:01:39 PM CST		
Date Processed:	6/4/2024 7:48:05 PM CST		

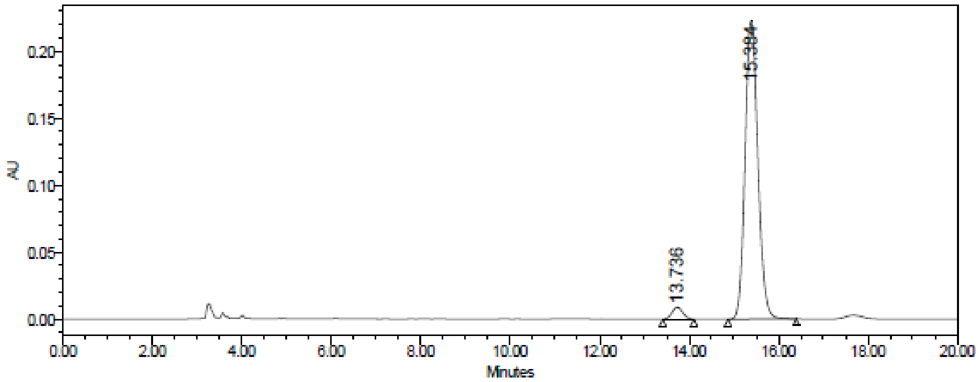

	RT	Area	% Area	Height
1	13.736	148236	3.22	8846
2	15.384	4449778	96.78	222525

rac-**3o**

SAMPLE INFORMATION

Sample Name:	zjy-12-149-10%-IC-rac	Acquired By:	System
Sample Type:	Unknown	Sample Set Name:	0
Vial:	61	Acq. Method Set:	10%quanbo
Injection #:	1	Processing Method:	1354685746
Injection Volume:	5.00 ul	Channel Name:	240.0nm
Run Time:	40.0 Minutes	Proc. Chnl. Descr.:	2998 PDA 240.0 nm (2998
Date Acquired:	9/23/2023 12:36:52 PM CST		
Date Processed:	10/13/2023 7:16:40 PM CST		

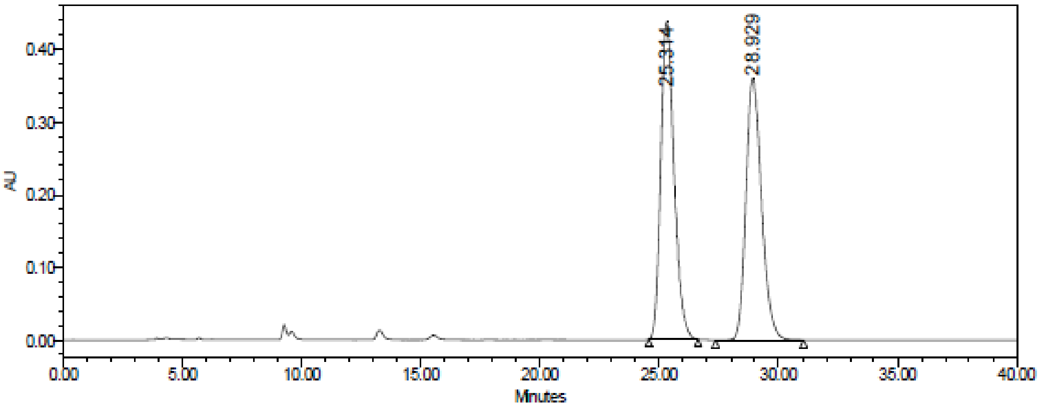

	RT	Area	% Area	Height
1	25.314	17015942	51.02	437426
2	28.929	16338281	48.98	360453

asy-**3o**

SAMPLE INFORMATION

Sample Name:	zjy-12-148-10%-IC-asy	Acquired By:	System
Sample Type:	Unknown	Sample Set Name:	0
Vial:	60	Acq. Method Set:	10%quanbo
Injection #:	1	Processing Method:	132
Injection Volume:	5.00 ul	Channel Name:	240.0nm
Run Time:	40.0 Minutes	Proc. Chnl. Descr.:	2998 PDA 240.0 nm (2998
Date Acquired:	9/23/2023 11:56:14 AM CST		
Date Processed:	10/13/2023 7:14:35 PM CST		

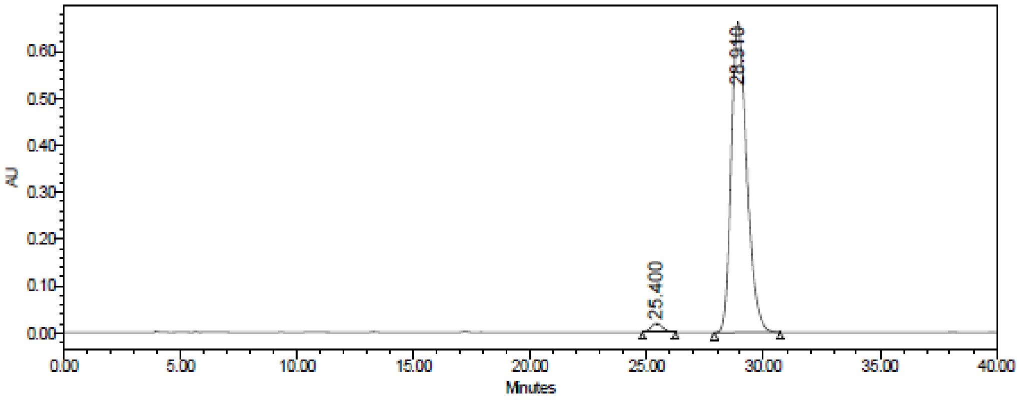

	RT	Area	% Area	Height
1	25.400	697180	2.24	19113
2	28.910	30379731	97.76	663219

rac-**3p**

SAMPLE INFORMATION

Sample Name:	zjy-12-163-10%-IF-rac	Acquired By:	System
Sample Type:	Unknown	Sample Set Name:	0
Vial:	56	Acq. Method Set:	10%quanbo
Injection #:	1	Processing Method:	54165463
Injection Volume:	10.00 ul	Channel Name:	240.0nm
Run Time:	20.0 Minutes	Proc. Chnl. Descr.:	2998 PDA 240.0 nm (2998
Date Acquired:	10/4/2023 8:47:49 PM CST		
Date Processed:	10/13/2023 7:22:34 PM CST		

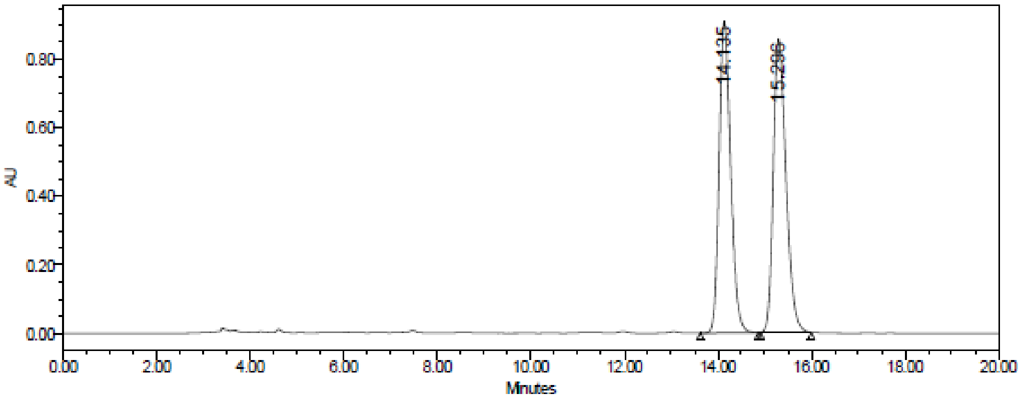

	RT	Area	% Area	Height
1	14.135	15547637	48.95	908523
2	15.296	16214939	51.05	856594

asy-**3p**

SAMPLE INFORMATION

Sample Name:	zjy-12-163-10%-IF-asy	Acquired By:	System
Sample Type:	Unknown	Sample Set Name:	0
Vial:	55	Acq. Method Set:	10%quanbo
Injection #:	1	Processing Method:	54165463
Injection Volume:	10.00 ul	Channel Name:	240.0nm
Run Time:	20.0 Minutes	Proc. Chnl. Descr.:	2998 PDA 240.0 nm (2998
Date Acquired:	10/4/2023 8:27:09 PM CST		
Date Processed:	10/13/2023 7:21:29 PM CST		

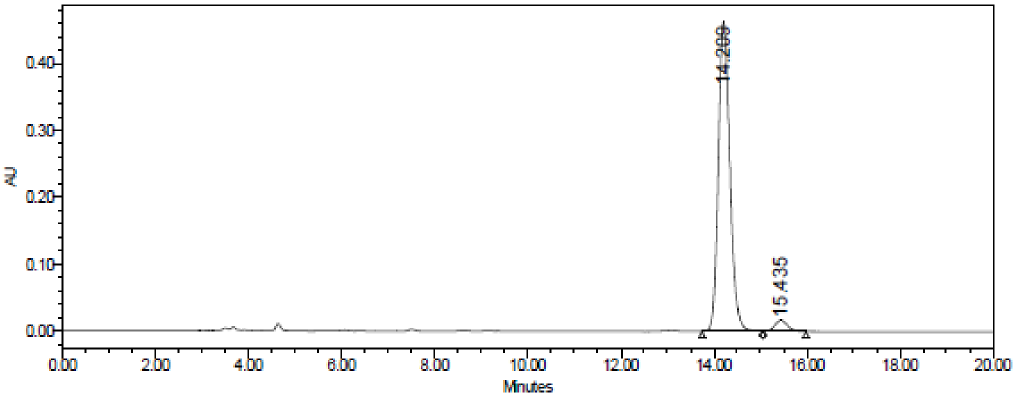

	RT	Area	% Area	Height
1	14.209	7956728	96.13	463930
2	15.435	320553	3.87	17097

rac-**3q**

SAMPLE INFORMATION

Sample Name:	zjy-12-164-10%-IF-rac	Acquired By:	System
Sample Type:	Unknown	Sample Set Name:	0
Vial:	58	Acq. Method Set:	10%quanbo
Injection #:	1	Processing Method:	1231
Injection Volume:	10.00 ul	Channel Name:	270.0nm
Run Time:	16.0 Minutes	Proc. Chnl. Descr.:	2998 PDA 270.0 nm (2998
Date Acquired:	10/4/2023 9:25:14 PM CST		
Date Processed:	10/13/2023 7:24:51 PM CST		

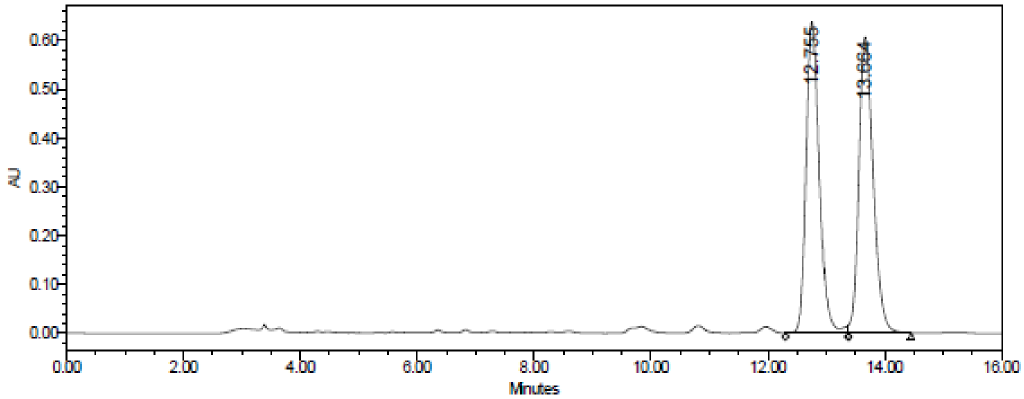

	RT	Area	% Area	Height
1	12.755	10002433	49.18	638032
2	13.664	10336360	50.82	606769

asy-**3q**

SAMPLE INFORMATION

Sample Name:	zjy-12-164-10%-IF-asy	Acquired By:	System
Sample Type:	Unknown	Sample Set Name:	0
Vial:	57	Acq. Method Set:	10%quanbo
Injection #:	1	Processing Method:	132
Injection Volume:	10.00 ul	Channel Name:	270.0nm
Run Time:	16.0 Minutes	Proc. Chnl. Descr.:	2998 PDA 270.0 nm (2998
Date Acquired:	10/4/2023 9:08:31 PM CST		
Date Processed:	10/13/2023 7:23:52 PM CST		

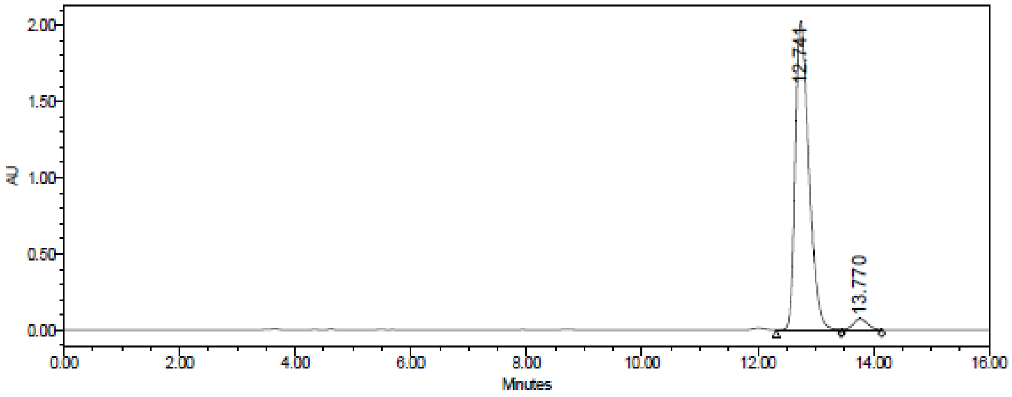

	RT	Area	% Area	Height
1	12.741	33072236	96.33	2025015
2	13.770	1258990	3.67	74172

rac-**3r**

SAMPLE INFORMATION

Sample Name:	zjy-12-166-10%-IC-rac	Acquired By:	System
Sample Type:	Unknown	Sample Set Name:	0
Vial:	57	Acq. Method Set:	10%quanbo
Injection #:	1	Processing Method:	1354685746
Injection Volume:	5.00 ul	Channel Name:	254.0nm
Run Time:	22.0 Minutes	Proc. Chnl. Descr.:	2998 PDA 254.0 nm (2998
Date Acquired:	10/5/2023 9:56:16 PM CST		
Date Processed:	10/13/2023 7:30:21 PM CST		

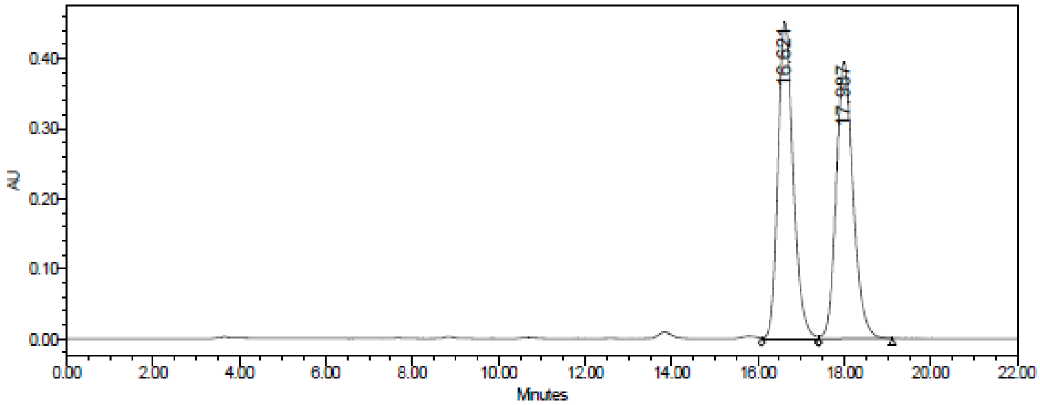

	RT	Area	% Area	Height
1	16.621	11113531	50.87	451820
2	17.987	10733254	49.13	395092

asy-**3r**

SAMPLE INFORMATION

Sample Name:	zjy-12-166-10%-IC-asy	Acquired By:	System
Sample Type:	Unknown	Sample Set Name:	0
Vial:	56	Acq. Method Set:	10%quanbo
Injection #:	1	Processing Method:	132
Injection Volume:	10.00 ul	Channel Name:	254.0nm
Run Time:	22.0 Minutes	Proc. Chnl. Descr.:	2998 PDA 254.0 nm (2998
Date Acquired:	10/5/2023 9:33:37 PM CST		
Date Processed:	10/13/2023 7:29:26 PM CST		

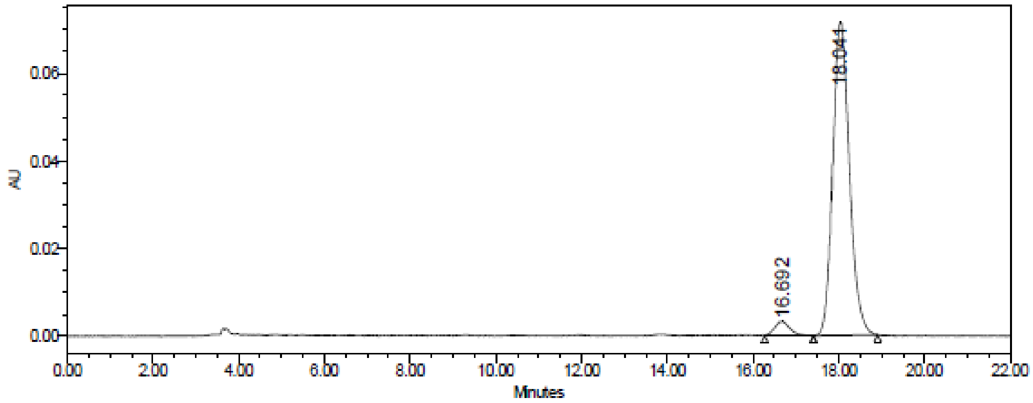

	RT	Area	% Area	Height
1	16.692	81913	4.08	3267
2	18.041	1925848	95.92	71576

rac-**3s**

SAMPLE INFORMATION

Sample Name:	zjy-12-169-5%-IC-rac	Acquired By:	System
Sample Type:	Unknown	Sample Set Name:	0
Vial:	55	Acq. Method Set:	5%quanbo
Injection #:	1	Processing Method:	54165463
Injection Volume:	5.00 ul	Channel Name:	254.0nm
Run Time:	21.0 Minutes	Proc. Chnl. Descr.:	2998 PDA 254.0 nm (2998
Date Acquired:	10/5/2023 8:17:23 PM CST		
Date Processed:	10/13/2023 7:28:30 PM CST		

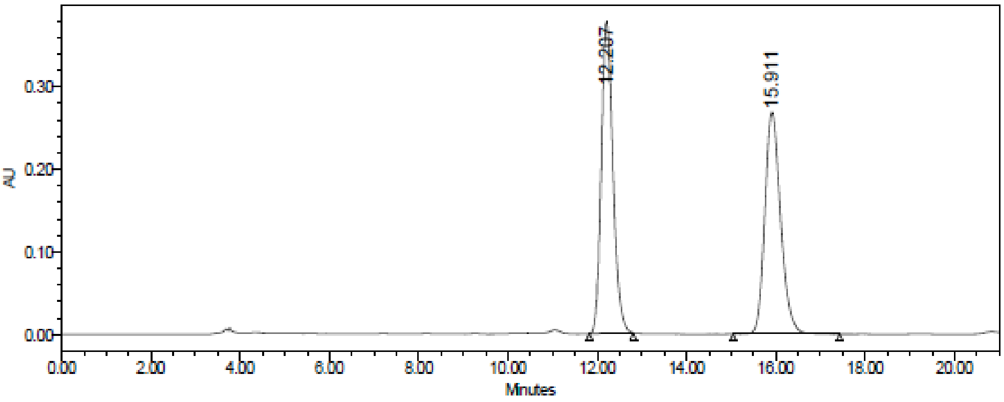

	RT	Area	% Area	Height
1	12.207	6840644	50.67	377821
2	15.911	6658656	49.33	268921

asy-**3s**

SAMPLE INFORMATION

Sample Name:	zjy-12-169-5%-IC-asy	Acquired By:	System
Sample Type:	Unknown	Sample Set Name:	0
Vial:	54	Acq. Method Set:	5%quanbo
Injection #:	1	Processing Method:	132
Injection Volume:	10.00 ul	Channel Name:	254.0nm
Run Time:	21.0 Minutes	Proc. Chnl. Descr.:	2998 PDA 254.0 nm (2998
Date Acquired:	10/5/2023 7:55:30 PM CST		
Date Processed:	10/13/2023 7:39:23 PM CST		

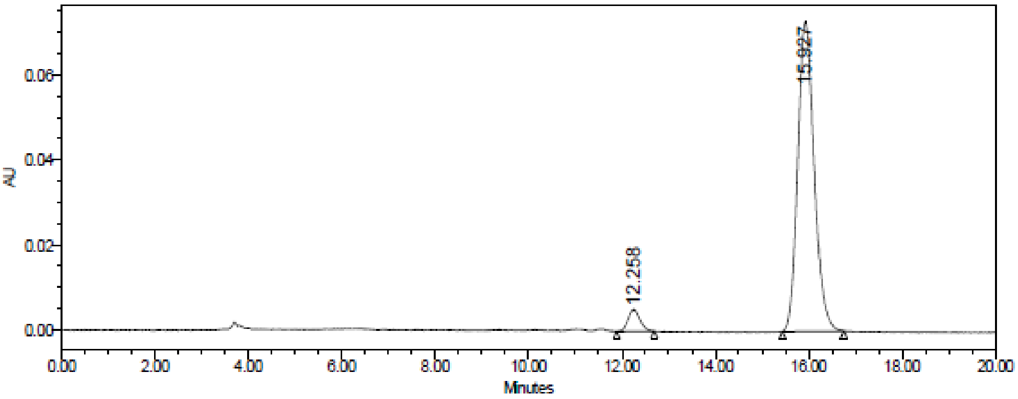

	RT	Area	% Area	Height
1	12.258	92349	4.93	5186
2	15.927	1782414	95.07	72874

rac-**3t**

SAMPLE INFORMATION

Sample Name:	zjy-12-173-5%-IC-rac	Acquired By:	System
Sample Type:	Unknown	Sample Set Name:	0
Vial:	56	Acq. Method Set:	5%quanbo
Injection #:	1	Processing Method:	ZJY 5 1261
Injection Volume:	10.00 ul	Channel Name:	254.0nm
Run Time:	20.0 Minutes	Proc. Chnl. Descr.:	2998 PDA 254.0 nm (2998
Date Acquired:	10/16/2023 11:30:44 AM CST		
Date Processed:	10/16/2023 9:31:32 PM CST		

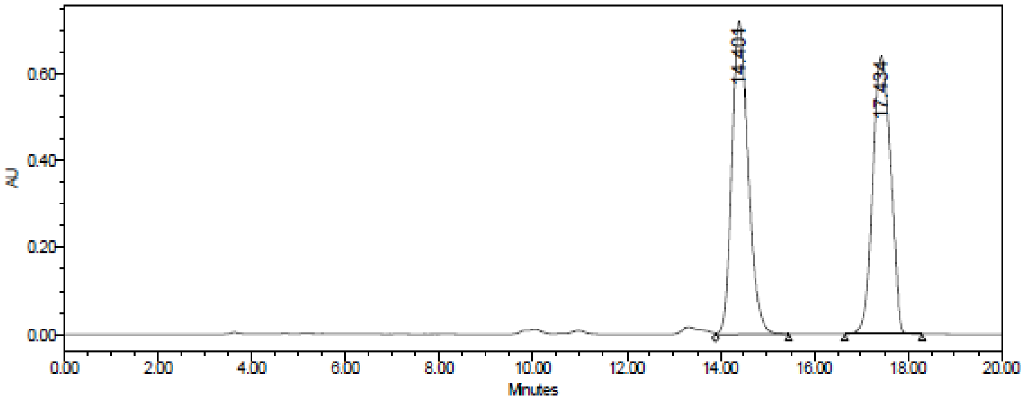

	RT	Area	% Area	Height
1	14.401	17254903	50.37	718019
2	17.434	16998491	49.63	639133

asy-**3t**

SAMPLE INFORMATION

Sample Name:	zjy-12-173-5%-IC-asy	Acquired By:	System
Sample Type:	Unknown	Sample Set Name:	0
Vial:	55	Acq. Method Set:	5%quanbo
Injection #:	1	Processing Method:	ZJY 5 22
Injection Volume:	10.00 ul	Channel Name:	254.0nm
Run Time:	20.0 Minutes	Proc. Chnl. Descr.:	2998 PDA 254.0 nm (2998
Date Acquired:	10/16/2023 11:07:41 AM CST		
Date Processed:	10/16/2023 9:30:52 PM CST		

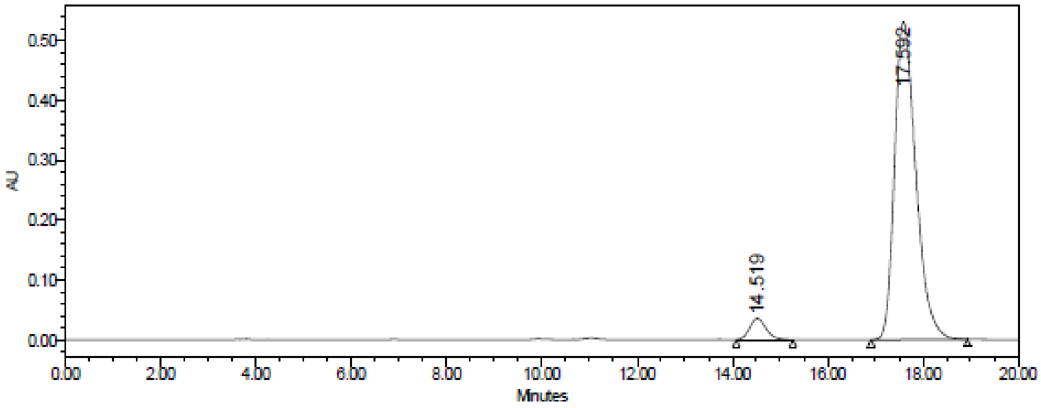

	RT	Area	% Area	Height
1	14.519	852065	4.95	35717
2	17.592	16365591	95.05	530502

rac-**3u**

SAMPLE INFORMATION

Sample Name:	zjy-12-167-5%-IC-rac	Acquired By:	System
Sample Type:	Unknown	Sample Set Name:	0
Vial:	61	Acq. Method Set:	5%quanbo
Injection #:	1	Processing Method:	1354685746
Injection Volume:	5.00 ul	Channel Name:	254.0nm
Run Time:	14.0 Minutes	Proc. Chnl. Descr.:	2998 PDA 254.0 nm (2998
Date Acquired:	10/5/2023 11:29:10 PM CST		
Date Processed:	10/13/2023 7:33:17 PM CST		

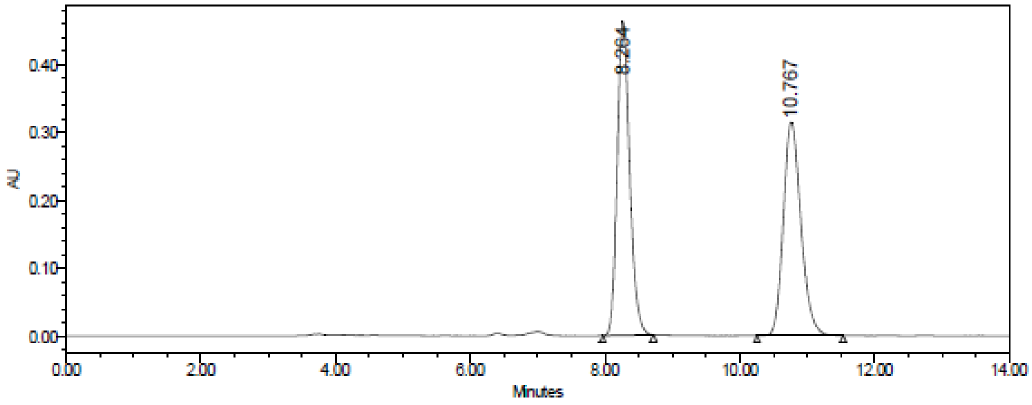

	RT	Area	% Area	Height
1	8.264	5967541	50.89	461995
2	10.767	5759674	49.11	314424

asy-**3u**

SAMPLE INFORMATION

Sample Name:	zjy-12-167-5%-IC-asy	Acquired By:	System
Sample Type:	Unknown	Sample Set Name:	0
Vial:	60	Acq. Method Set:	5%quanbo
Injection #:	1	Processing Method:	1231
Injection Volume:	10.00 ul	Channel Name:	254.0nm
Run Time:	14.0 Minutes	Proc. Chnl. Descr.:	2998 PDA 254.0 nm (2998
Date Acquired:	10/5/2023 11:14:31 PM CST		
Date Processed:	10/13/2023 7:32:31 PM CST		

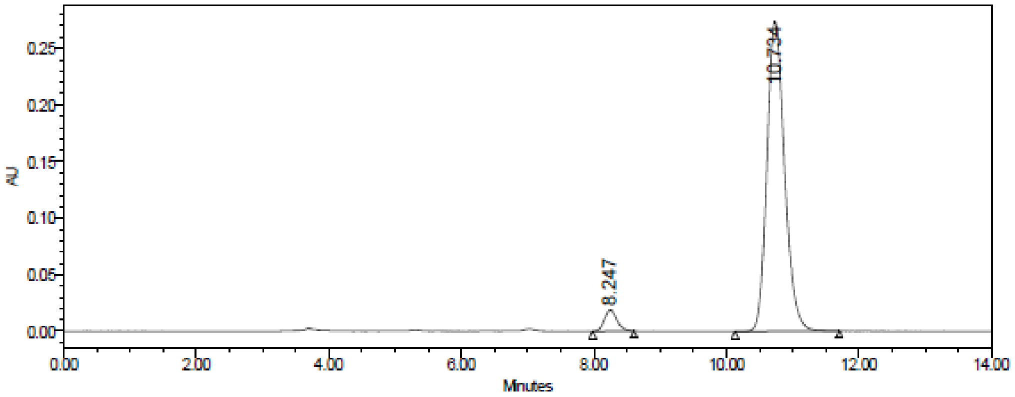

	RT	Area	% Area	Height
1	8.247	244543	4.59	18606
2	10.734	5080293	95.41	273730

rac-**3v**

SAMPLE INFORMATION

Sample Name:	zjy-12-168-10%-IC-rac	Acquired By:	System
Sample Type:	Unknown	Sample Set Name:	
Vial:	59	Acq. Method Set:	10%quanbo
Injection #:	1	Processing Method:	132
Injection Volume:	5.00 ul	Channel Name:	254.0nm
Run Time:	60.0 Minutes	Proc. Chnl. Descr.:	2998 PDA 254.0 nm (2998
Date Acquired:	10/6/2023 9:24:00 AM CST		
Date Processed:	10/13/2023 7:34:02 PM CST		

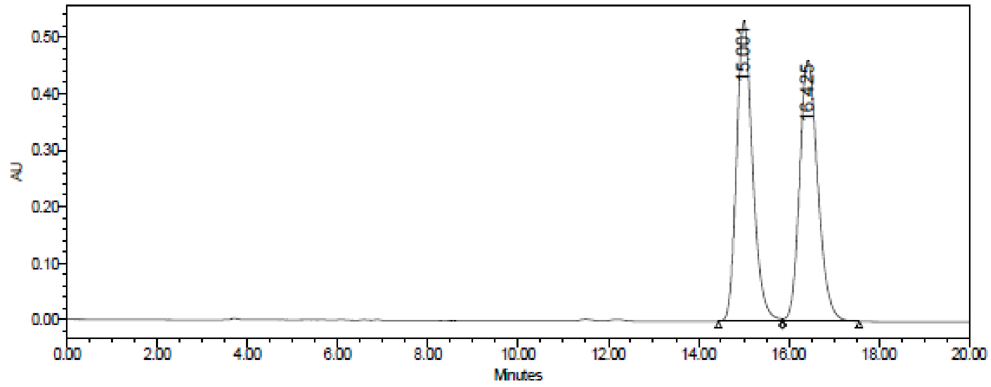

	RT	Area	% Area	Height
1	15.001	13241064	50.69	532176
2	16.425	12879234	49.31	462553

asy-**3v**

SAMPLE INFORMATION

Sample Name:	zjy-12-168-10%-IC-asy	Acquired By:	System
Sample Type:	Unknown	Sample Set Name:	0
Vial:	58	Acq. Method Set:	10%quanbo
Injection #:	1	Processing Method:	54165463
Injection Volume:	10.00 ul	Channel Name:	254.0nm
Run Time:	20.0 Minutes	Proc. Chnl. Descr.:	2998 PDA 254.0 nm (2998
Date Acquired:	10/6/2023 10:03:55 AM CST		
Date Processed:	10/13/2023 7:35:22 PM CST		

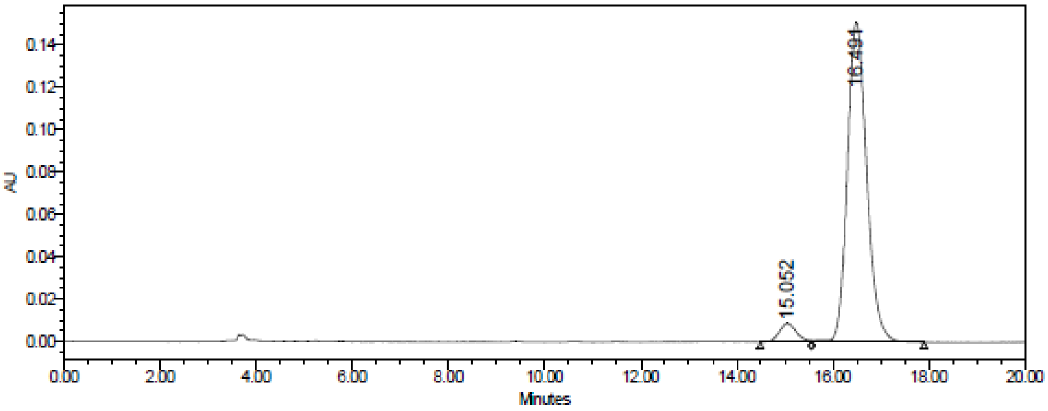

	RT	Area	% Area	Height
1	15.052	219708	4.97	8787
2	16.491	4200998	95.03	151062

rac-**3w**

SAMPLE INFORMATION

Sample Name:	zjy-12-174-5%-IC-rac	Acquired By:	System
Sample Type:	Unknown	Sample Set Name:	0
Vial:	63	Acq. Method Set:	5%quanbo
Injection #:	1	Processing Method:	54165463
Injection Volume:	10.00 ul	Channel Name:	254.0nm
Run Time:	20.0 Minutes	Proc. Chnl. Descr.:	2998 PDA 254.0 nm (2998
Date Acquired:	10/7/2023 1:37:37 PM CST		
Date Processed:	10/13/2023 7:42:29 PM CST		

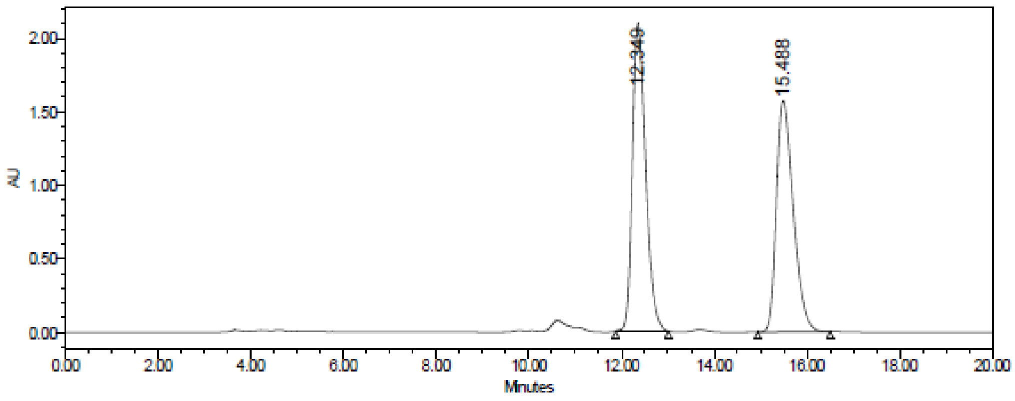

	RT	Area	% Area	Height
1	12.349	41736624	51.04	2091360
2	15.488	40040624	48.96	1573641

asy-**3w**

SAMPLE INFORMATION

Sample Name:	zjy-12-174-5%-IC-asy	Acquired By:	System
Sample Type:	Unknown	Sample Set Name:	0
Vial:	62	Acq. Method Set:	5%quanbo
Injection #:	1	Processing Method:	54165463
Injection Volume:	10.00 ul	Channel Name:	254.0nm
Run Time:	20.0 Minutes	Proc. Chnl. Descr.:	2998 PDA 254.0 nm (2998
Date Acquired:	10/7/2023 1:16:56 PM CST		
Date Processed:	10/13/2023 7:41:37 PM CST		

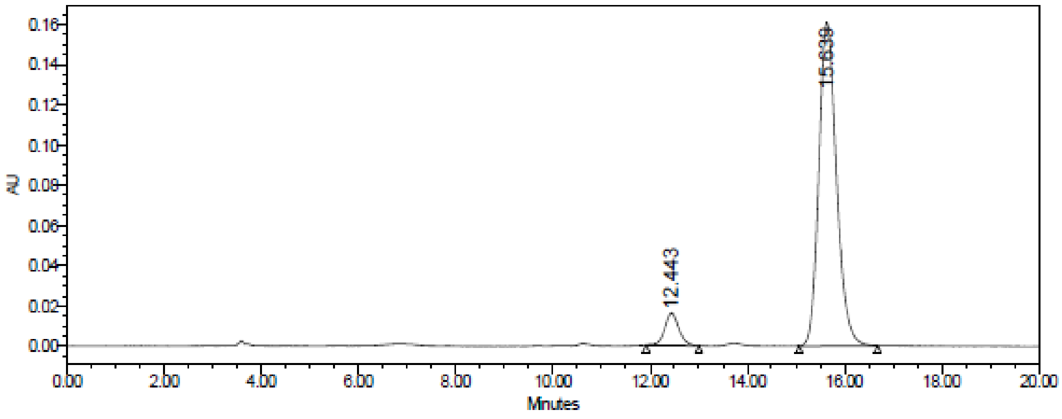

	RT	Area	% Area	Height
1	12.443	324892	7.38	16120
2	15.639	4074911	92.62	160617

rac-**3x**

SAMPLE INFORMATION			
Sample Name:	zjy-15-165-10%-IE-rac	Acquired By:	System
Sample Type:	Unknown	Sample Set Name:	
Vial:	52	Acq. Method Set:	10%quanbo
Injection #:	2	Processing Method:	54165463
Injection Volume:	5.00 ul	Channel Name:	212.0nm
Run Time:	60.0 Minutes	Proc. Chnl. Descr.:	2998 PDA 212.0 nm (2998
Date Acquired:	12/26/2024 8:05:20 PM CST		
Date Processed:	1/3/2025 10:41:42 PM CST		

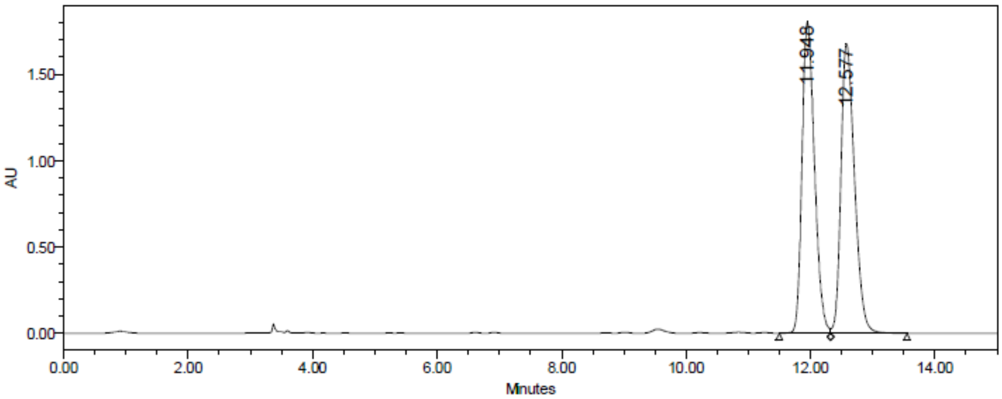

	RT	Area	% Area	Height
1	11.948	25210400	49.65	1802492
2	12.577	25568972	50.35	1676032

asy-**3x**

SAMPLE INFORMATION			
Sample Name:	zjy-15-166-10%-IE-asy	Acquired By:	System
Sample Type:	Unknown	Sample Set Name:	
Vial:	53	Acq. Method Set:	10%quanbo
Injection #:	1	Processing Method:	1354685746
Injection Volume:	5.00 ul	Channel Name:	212.0nm
Run Time:	60.0 Minutes	Proc. Chnl. Descr.:	2998 PDA 212.0 nm (2998
Date Acquired:	12/26/2024 8:30:18 PM CST		
Date Processed:	1/3/2025 10:40:22 PM CST		

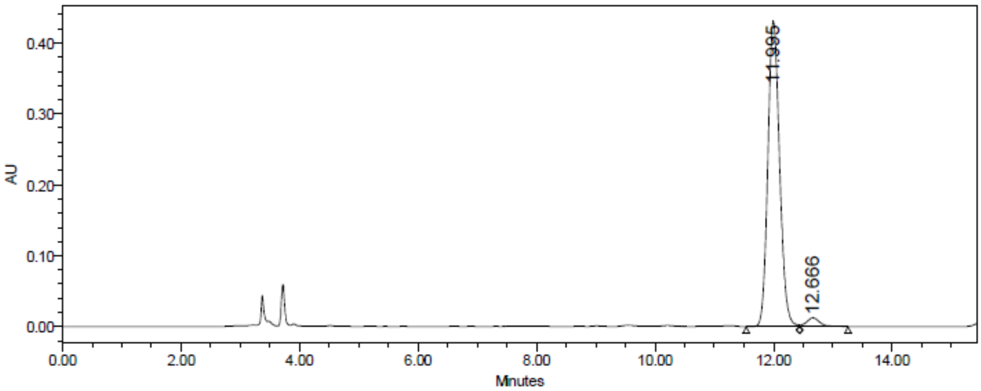

	RT	Area	% Area	Height
1	11.995	5932436	96.79	430504
2	12.666	196909	3.21	12418

rac-**5a**

SAMPLE INFORMATION

Sample Name:	zjy-9-39-5%-IA-rac	Acquired By:	System
Sample Type:	Unknown	Sample Set Name:	0
Vial:	54	Acq. Method Set:	5%quanbo
Injection #:	1	Processing Method:	1231
Injection Volume:	3.00 ul	Channel Name:	240.0nm
Run Time:	10.0 Minutes	Proc. Chnl. Descr.:	2998 PDA 240.0 nm (2998
Date Acquired:	7/27/2022 8:49:16 PM CST		
Date Processed:	12/31/2022 5:07:56 PM CST		

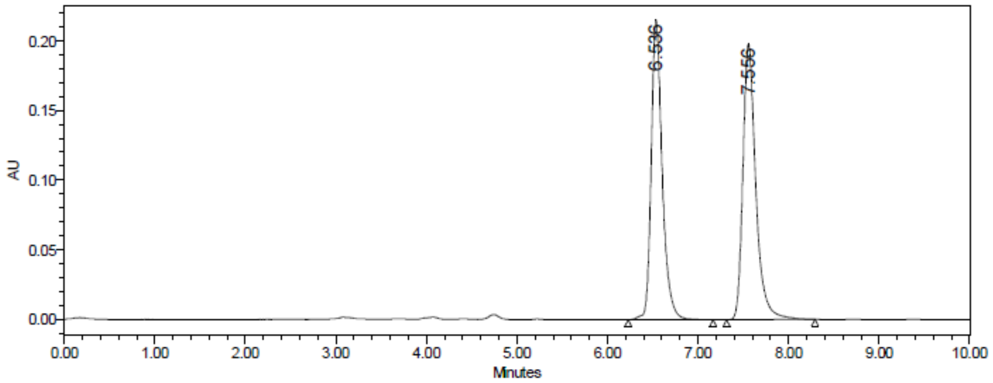

	RT	Area	% Area	Height
1	6.536	1812901	48.11	215099
2	7.556	1955289	51.89	197751

asy-**5a**

SAMPLE INFORMATION

Sample Name:	zjy-9-39-5%-IA-asy	Acquired By:	System
Sample Type:	Unknown	Sample Set Name:	0
Vial:	53	Acq. Method Set:	5%quanbo
Injection #:	1	Processing Method:	1231
Injection Volume:	3.00 ul	Channel Name:	240.0nm
Run Time:	10.0 Minutes	Proc. Chnl. Descr.:	2998 PDA 240.0 nm (2998
Date Acquired:	7/27/2022 8:38:38 PM CST		
Date Processed:	12/31/2022 5:06:07 PM CST		

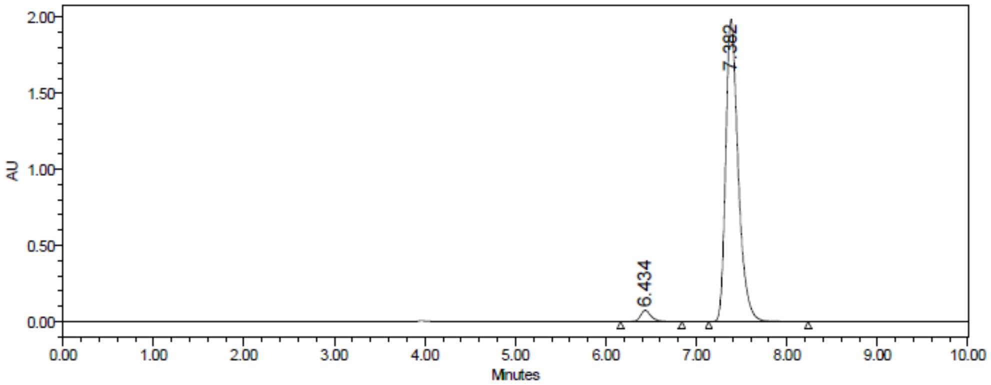

	RT	Area	% Area	Height
1	6.434	613429	3.13	74049
2	7.382	19003010	96.87	1982351

rac-**5b**

SAMPLE INFORMATION

Sample Name:	zjy-9-351(58)-5%-IC-RAC	Acquired By:	System
Sample Type:	Unknown	Sample Set Name:	0
Vial:	64	Acq. Method Set:	5%quanbo
Injection #:	1	Processing Method:	ZJY 5 207 asy
Injection Volume:	10.00 ul	Channel Name:	245.0nm
Run Time:	20.0 Minutes	Proc. Chnl. Descr.:	2998 PDA 245.0 nm (2998
Date Acquired:	10/27/2022 11:50:42 AM CST		
Date Processed:	12/31/2022 3:39:36 PM CST		

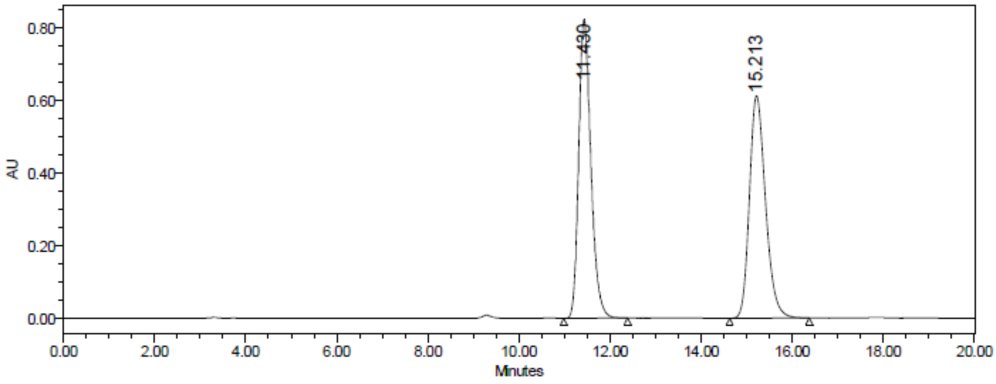

	RT	Area	% Area	Height
1	11.430	15118855	50.10	821102
2	15.213	15060028	49.90	612194

asy-**5b**

SAMPLE INFORMATION

Sample Name:	zjy-9-351-5%-IC-asy	Acquired By:	System
Sample Type:	Unknown	Sample Set Name:	0
Vial:	63	Acq. Method Set:	5%quanbo
Injection #:	1	Processing Method:	LC PQ
Injection Volume:	10.00 ul	Channel Name:	260.0nm
Run Time:	20.0 Minutes	Proc. Chnl. Descr.:	2998 PDA 260.0 nm (2998
Date Acquired:	10/27/2022 11:30:02 AM CST		
Date Processed:	12/31/2022 3:48:30 PM CST		

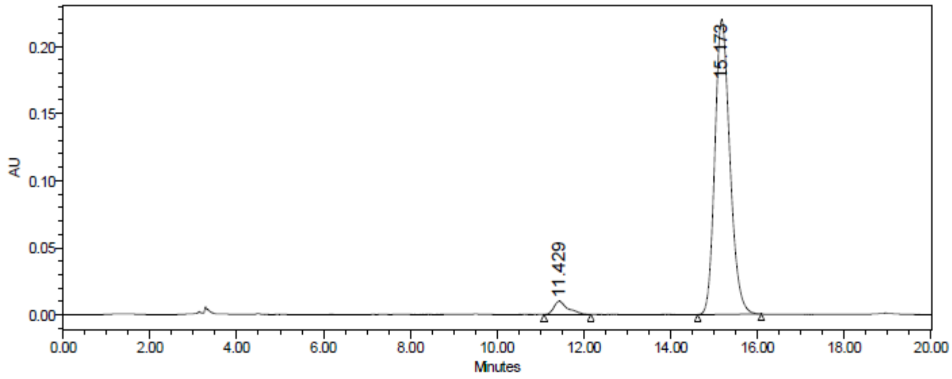

	RT	Area	% Area	Height
1	11.429	222684	4.01	9898
2	15.173	5331142	95.99	219783

rac-**5c**

SAMPLE INFORMATION			
Sample Name:	zjy-9-352(58)-5%-IC-RAC	Acquired By:	System
Sample Type:	Unknown	Sample Set Name:	0
Vial:	67	Acq. Method Set:	15%quanbo
Injection #:	1	Processing Method:	Default
Injection Volume:	10.00 ul	Channel Name:	270.0nm
Run Time:	15.0 Minutes	Proc. Chnl. Descr.:	2998 PDA 270.0 nm (2998
Date Acquired:	10/27/2022 12:37:25 PM CST		
Date Processed:	12/31/2022 3:36:44 PM CST		

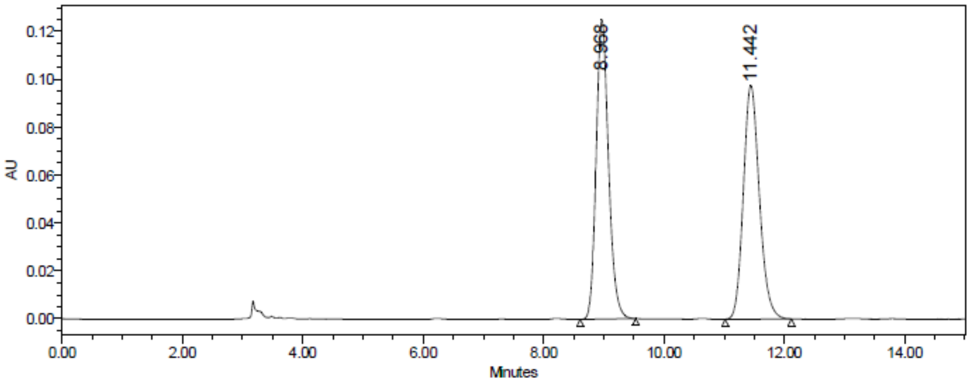

	RT	Area	% Area	Height
1	8.968	1791935	49.69	124995
2	11.442	1814366	50.31	97645

asy-**5c**

SAMPLE INFORMATION			
Sample Name:	zjy-9-352-5%-IC-asy	Acquired By:	System
Sample Type:	Unknown	Sample Set Name:	0
Vial:	66	Acq. Method Set:	15%quanbo
Injection #:	1	Processing Method:	1354685746
Injection Volume:	10.00 ul	Channel Name:	270.0nm
Run Time:	15.0 Minutes	Proc. Chnl. Descr.:	2998 PDA 270.0 nm (2998
Date Acquired:	10/27/2022 12:21:30 PM CST		
Date Processed:	12/31/2022 3:30:16 PM CST		

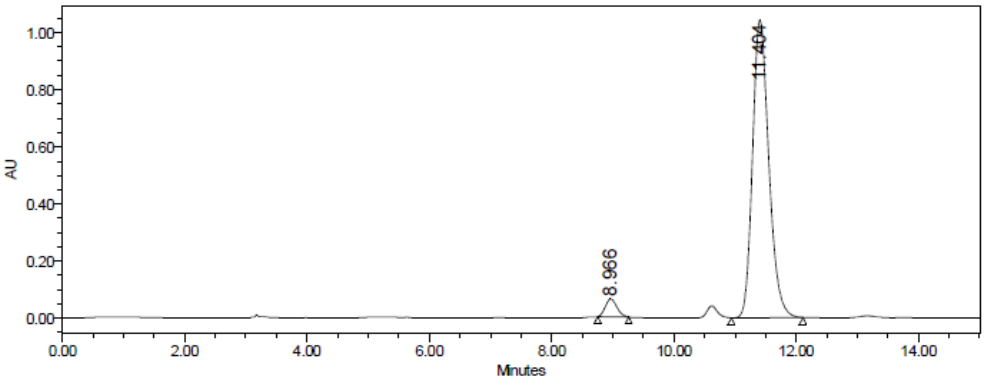

	RT	Area	% Area	Height
1	8.966	834204	4.13	63083
2	11.404	19360300	95.87	1041904

rac-**5d**

SAMPLE INFORMATION

Sample Name:	zjy-9-353(43)-5%-IA-RAC	Acquired By:	System
Sample Type:	Unknown	Sample Set Name:	0
Vial:	69	Acq. Method Set:	5%quanbo
Injection #:	1	Processing Method:	LC PQ
Injection Volume:	10.00 ul	Channel Name:	275.0nm
Run Time:	16.0 Minutes	Proc. Chnl. Descr.:	2998 PDA 275.0 nm (2998
Date Acquired:	10/27/2022 1:25:41 PM CST		
Date Processed:	12/31/2022 3:25:21 PM CST		

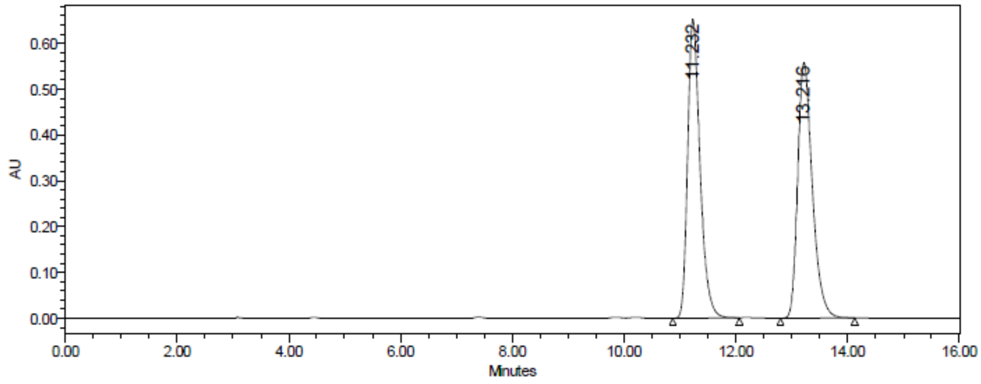

	RT	Area	% Area	Height
1	11.232	10059917	49.93	651162
2	13.216	10088925	50.07	557222

asy-**5d**

SAMPLE INFORMATION

Sample Name:	zjy-9-353-5%-IA-asy	Acquired By:	System
Sample Type:	Unknown	Sample Set Name:	0
Vial:	68	Acq. Method Set:	5%quanbo
Injection #:	1	Processing Method:	LC PQ
Injection Volume:	10.00 ul	Channel Name:	270.0nm
Run Time:	16.0 Minutes	Proc. Chnl. Descr.:	2998 PDA 270.0 nm (2998
Date Acquired:	10/27/2022 1:08:57 PM CST		
Date Processed:	12/31/2022 3:26:19 PM CST		

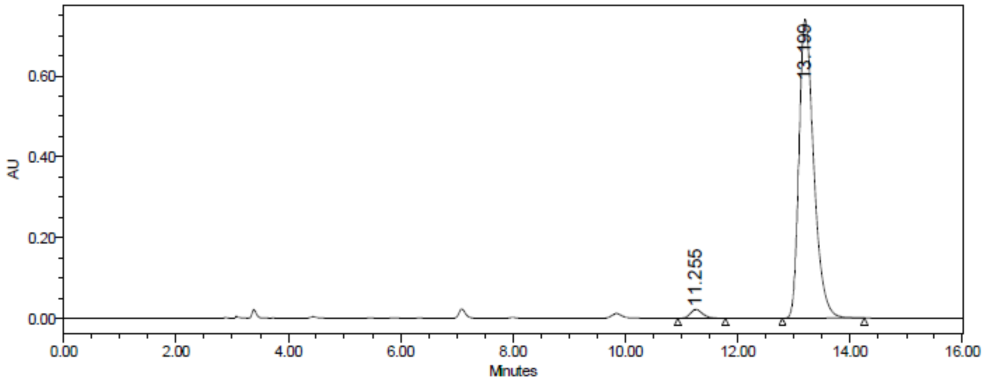

	RT	Area	% Area	Height
1	11.255	338753	2.46	22143
2	13.199	13444855	97.54	739113

rac-**5e**

SAMPLE INFORMATION

Sample Name:	zjy-9-301-5%-IA-RAC	Acquired By:	System
Sample Type:	Unknown	Sample Set Name:	0
Vial:	51	Acq. Method Set:	5%quanbo
Injection #:	1	Processing Method:	1231
Injection Volume:	5.00 ul	Channel Name:	240.0nm
Run Time:	13.0 Minutes	Proc. Chnl. Descr.:	2998 PDA 240.0 nm (2998
Date Acquired:	10/13/2022 10:01:01 PM CST		
Date Processed:	12/31/2022 4:48:15 PM CST		

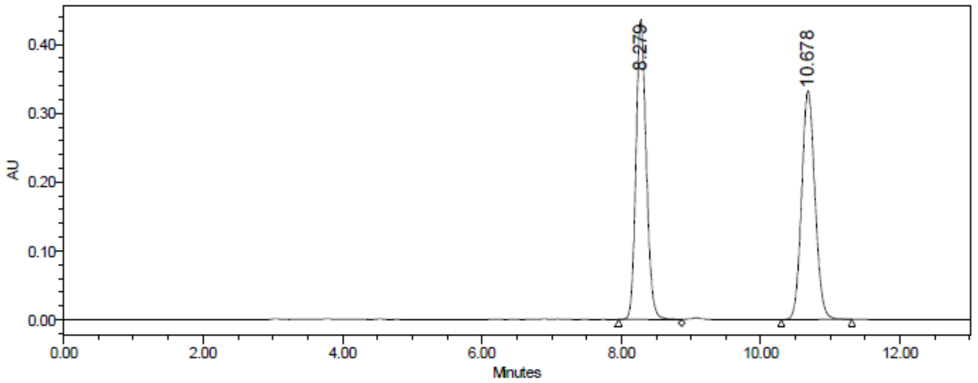

	RT	Area	% Area	Height
1	8.279	4449505	50.24	435016
2	10.678	4406556	49.76	332260

asy-**5e**

SAMPLE INFORMATION

Sample Name:	zjy-9-301-5%-IA-asy	Acquired By:	System
Sample Type:	Unknown	Sample Set Name:	0
Vial:	52	Acq. Method Set:	5%quanbo
Injection #:	1	Processing Method:	1231
Injection Volume:	5.00 ul	Channel Name:	240.0nm
Run Time:	13.0 Minutes	Proc. Chnl. Descr.:	2998 PDA 240.0 nm (2998
Date Acquired:	10/13/2022 10:14:40 PM CST		
Date Processed:	12/31/2022 4:47:13 PM CST		

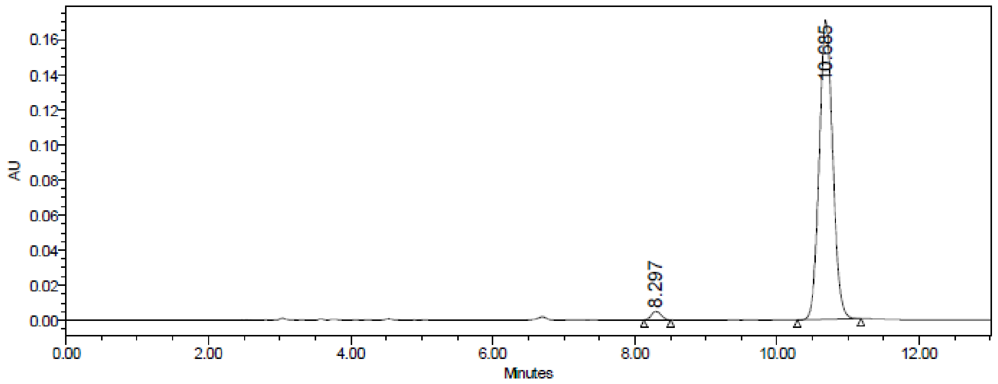

	RT	Area	% Area	Height
1	8.297	48730	2.12	5050
2	10.685	2249235	97.88	170332

rac-**5f**

SAMPLE INFORMATION			
Sample Name:	zjy-9-325-5%-IA-RAC	Acquired By:	System
Sample Type:	Unknown	Sample Set Name:	0
Vial:	49	Acq. Method Set:	5%quanbo
Injection #:	1	Processing Method:	1231
Injection Volume:	5.00 ul	Channel Name:	240.0nm
Run Time:	15.0 Minutes	Proc. Chnl. Descr.:	2998 PDA 240.0 nm (2998
Date Acquired:	10/13/2022 9:29:30 PM CST		
Date Processed:	12/31/2022 4:50:01 PM CST		

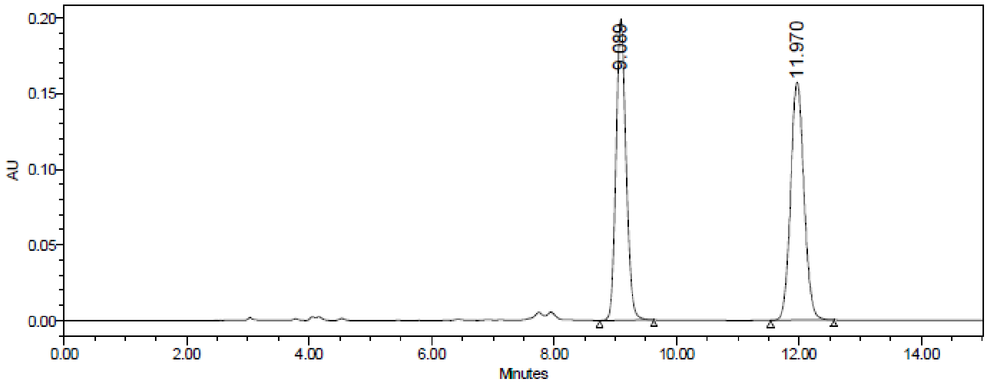

	RT	Area	% Area	Height
1	9.089	2274840	48.80	198581
2	11.970	2386359	51.20	157193

asy-**5f**

SAMPLE INFORMATION			
Sample Name:	zjy-9-325-5%-IA-asy	Acquired By:	System
Sample Type:	Unknown	Sample Set Name:	
Vial:	71	Acq. Method Set:	5%quanbo
Injection #:	1	Processing Method:	1231
Injection Volume:	5.00 ul	Channel Name:	240.0nm
Run Time:	15.0 Minutes	Proc. Chnl. Descr.:	2998 PDA 240.0 nm (2998
Date Acquired:	10/13/2022 8:36:48 PM CST		
Date Processed:	12/31/2022 4:51:41 PM CST		

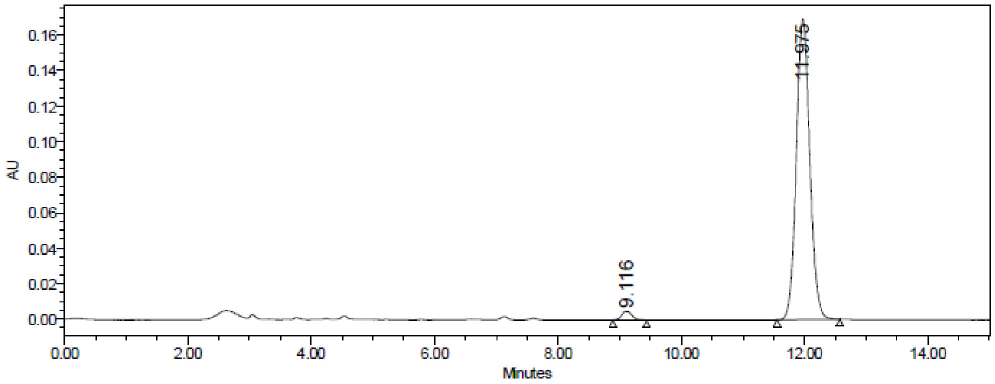

	RT	Area	% Area	Height
1	9.116	57204	2.18	5036
2	11.975	2570828	97.82	169100

rac-**5g**

SAMPLE INFORMATION

Sample Name:	zjy-9-326-5%-IA-RAC	Acquired By:	System
Sample Type:	Unknown	Sample Set Name:	0
Vial:	50	Acq. Method Set:	5%quanbo
Injection #:	1	Processing Method:	1231
Injection Volume:	5.00 ul	Channel Name:	240.0nm
Run Time:	15.0 Minutes	Proc. Chnl. Descr.:	2998 PDA 240.0 nm (2998
Date Acquired:	10/13/2022 9:45:21 PM CST		
Date Processed:	12/31/2022 4:49:05 PM CST		

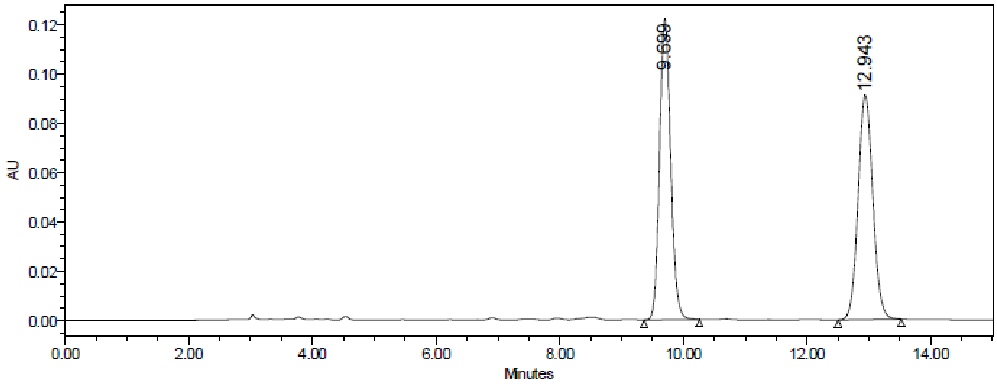

	RT	Area	% Area	Height
1	9.699	1513606	50.05	121993
2	12.943	1510683	49.95	91004

asy-**5g**

SAMPLE INFORMATION

Sample Name:	zjy-9-326-5%-IA-asy	Acquired By:	System
Sample Type:	Unknown	Sample Set Name:	
Vial:	72	Acq. Method Set:	5%quanbo
Injection #:	1	Processing Method:	1231
Injection Volume:	5.00 ul	Channel Name:	240.0nm
Run Time:	15.0 Minutes	Proc. Chnl. Descr.:	2998 PDA 240.0 nm (2998
Date Acquired:	10/13/2022 8:52:52 PM CST		
Date Processed:	12/31/2022 4:50:47 PM CST		

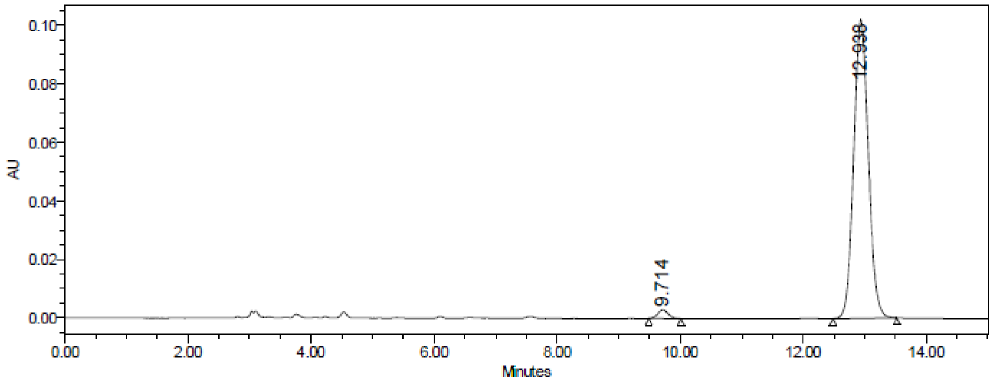

	RT	Area	% Area	Height
1	9.714	35904	2.07	2959
2	12.938	1695906	97.93	102102

rac-**5h**

SAMPLE INFORMATION

Sample Name:	zjy-9-339-5%-IA-RAC	Acquired By:	System
Sample Type:	Unknown	Sample Set Name:	0
Vial:	52	Acq. Method Set:	5%quanbo
Injection #:	1	Processing Method:	1231
Injection Volume:	5.00 ul	Channel Name:	240.0nm
Run Time:	18.0 Minutes	Proc. Chnl. Descr.:	2998 PDA 240.0 nm (2998
Date Acquired:	10/25/2022 3:53:35 PM CST		
Date Processed:	12/31/2022 4:28:19 PM CST		

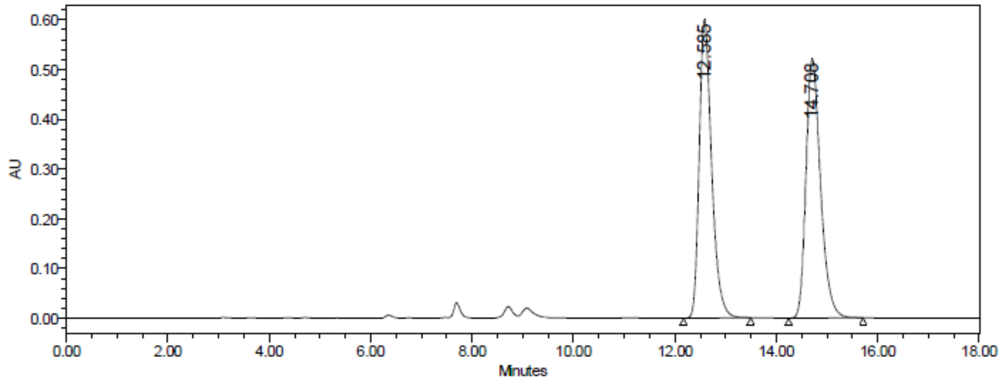

	RT	Area	% Area	Height
1	12.585	10259246	49.85	600195
2	14.708	10322540	50.15	521023

asy-**5h**

SAMPLE INFORMATION

Sample Name:	zjy-9-339-5%-IA-asy	Acquired By:	System
Sample Type:	Unknown	Sample Set Name:	0
Vial:	50	Acq. Method Set:	5%quanbo
Injection #:	1	Processing Method:	1231
Injection Volume:	5.00 ul	Channel Name:	240.0nm
Run Time:	18.0 Minutes	Proc. Chnl. Descr.:	2998 PDA 240.0 nm (2998
Date Acquired:	10/25/2022 3:20:03 PM CST		
Date Processed:	12/31/2022 4:25:11 PM CST		

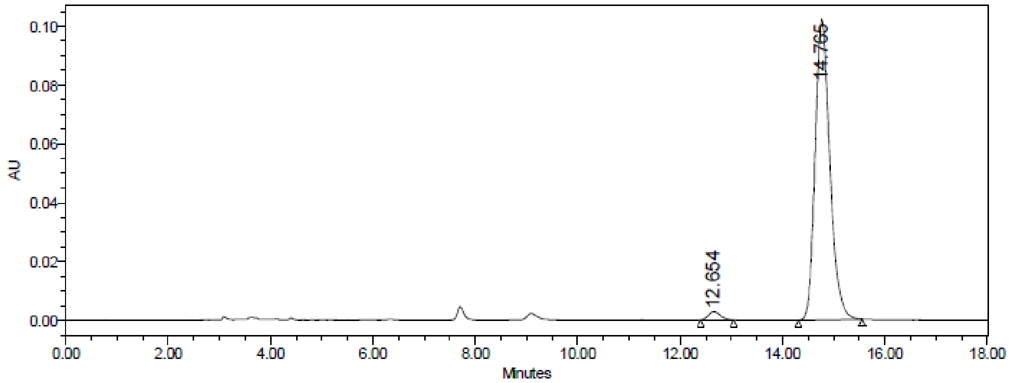

	RT	Area	% Area	Height
1	12.654	45554	2.20	2850
2	14.765	2028785	97.80	102174

rac-**5i**

SAMPLE INFORMATION			
Sample Name:	zjy-9-338-5%-IA-RAC	Acquired By:	System
Sample Type:	Unknown	Sample Set Name:	0
Vial:	51	Acq. Method Set:	5%quanbo
Injection #:	1	Processing Method:	1231
Injection Volume:	5.00 ul	Channel Name:	240.0nm
Run Time:	14.0 Minutes	Proc. Chnl. Descr.:	2998 PDA 240.0 nm (2998
Date Acquired:	10/25/2022 3:38:54 PM CST		
Date Processed:	12/31/2022 4:27:33 PM CST		

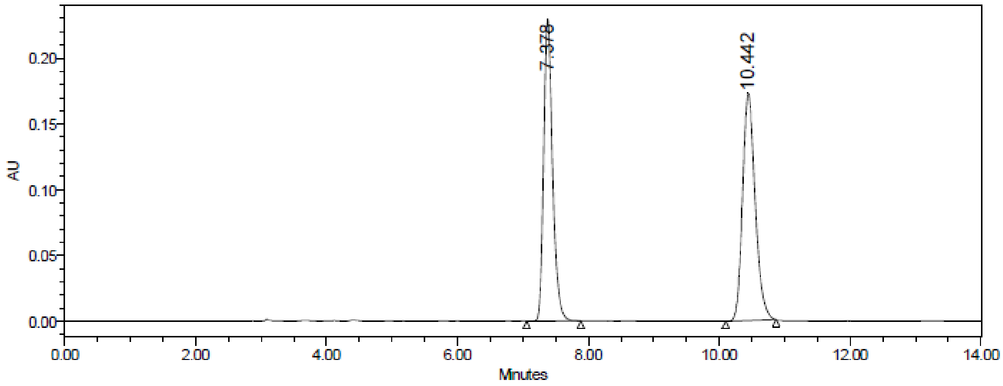

	RT	Area	% Area	Height
1	7.378	2179445	48.43	229020
2	10.442	2320421	51.57	172679

asy-**5i**

SAMPLE INFORMATION			
Sample Name:	zjy-9-338-5%-IA-asy	Acquired By:	System
Sample Type:	Unknown	Sample Set Name:	0
Vial:	49	Acq. Method Set:	5%quanbo
Injection #:	1	Processing Method:	1231
Injection Volume:	5.00 ul	Channel Name:	240.0nm
Run Time:	14.0 Minutes	Proc. Chnl. Descr.:	2998 PDA 240.0 nm (2998
Date Acquired:	10/25/2022 3:04:39 PM CST		
Date Processed:	12/31/2022 4:26:24 PM CST		

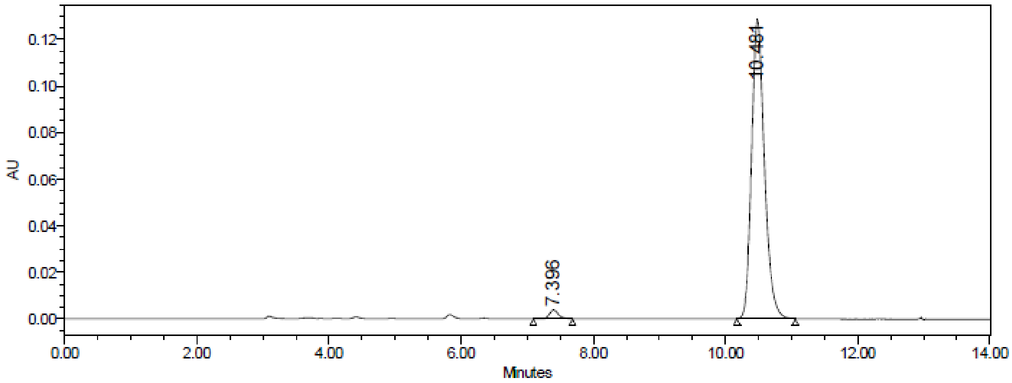

	RT	Area	% Area	Height
1	7.396	35985	2.02	3786
2	10.481	1745169	97.98	128538

rac-**5j**

SAMPLE INFORMATION

Sample Name:	zjy-9-364(323)-5%-IA-asy	Acquired By:	System
Sample Type:	Unknown	Sample Set Name:	0
Vial:	51	Acq. Method Set:	5%quanbo
Injection #:	1	Processing Method:	LC PQ
Injection Volume:	10.00 ul	Channel Name:	240.0nm
Run Time:	7.0 Minutes	Proc. Chnl. Descr.:	2998 PDA 240.0 nm (2998
Date Acquired:	10/27/2022 2:26:54 PM CST		
Date Processed:	12/31/2022 11:27:59 AM CST		

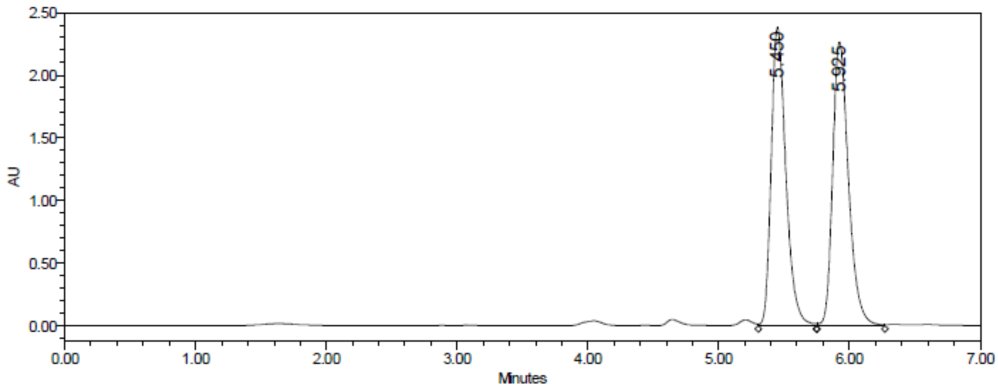

	RT	Area	% Area	Height
1	5.450	18577544	49.42	2380116
2	5.925	19014780	50.58	2262887

asy-**5j**

SAMPLE INFORMATION

Sample Name:	zjy-9-364-5%-IA-asy	Acquired By:	System
Sample Type:	Unknown	Sample Set Name:	0
Vial:	50	Acq. Method Set:	5%quanbo
Injection #:	1	Processing Method:	1354685746
Injection Volume:	10.00 ul	Channel Name:	245.0nm
Run Time:	7.0 Minutes	Proc. Chnl. Descr.:	2998 PDA 245.0 nm (2998
Date Acquired:	10/27/2022 2:19:12 PM CST		
Date Processed:	12/31/2022 11:26:35 AM CST		

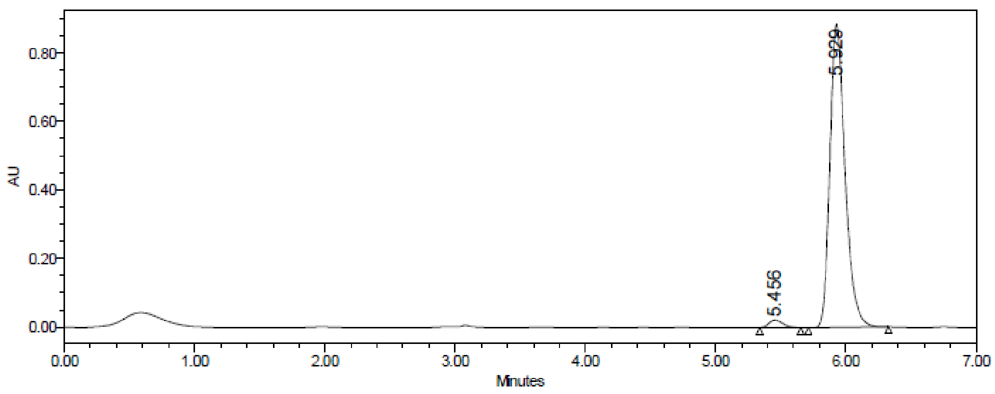

	RT	Area	% Area	Height
1	5.456	158544	2.17	21783
2	5.929	7140724	97.83	881868

rac-**5k**

SAMPLE INFORMATION

Sample Name:	zjy-9-346(324)-5%-IA-RAC	Acquired By:	System
Sample Type:	Unknown	Sample Set Name:	0
Vial:	53	Acq. Method Set:	5%quanbo
Injection #:	1	Processing Method:	1231
Injection Volume:	5.00 ul	Channel Name:	240.0nm
Run Time:	9.0 Minutes	Proc. Chnl. Descr.:	2998 PDA 240.0 nm (2998
Date Acquired:	10/25/2022 4:12:14 PM CST		
Date Processed:	12/31/2022 4:23:33 PM CST		

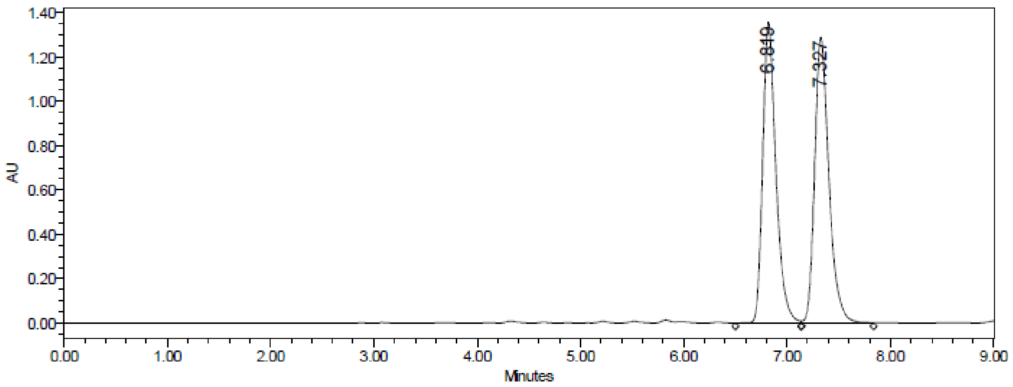

	RT	Area	% Area	Height
1	6.819	12031824	49.20	1355101
2	7.327	12424462	50.80	1289204

asy-**5k**

SAMPLE INFORMATION

Sample Name:	zjy-9-346-5%-IA-asy	Acquired By:	System
Sample Type:	Unknown	Sample Set Name:	0
Vial:	54	Acq. Method Set:	5%quanbo
Injection #:	1	Processing Method:	1231
Injection Volume:	10.00 ul	Channel Name:	240.0nm
Run Time:	9.0 Minutes	Proc. Chnl. Descr.:	2998 PDA 240.0 nm (2998
Date Acquired:	10/25/2022 4:21:53 PM CST		
Date Processed:	12/31/2022 4:22:12 PM CST		

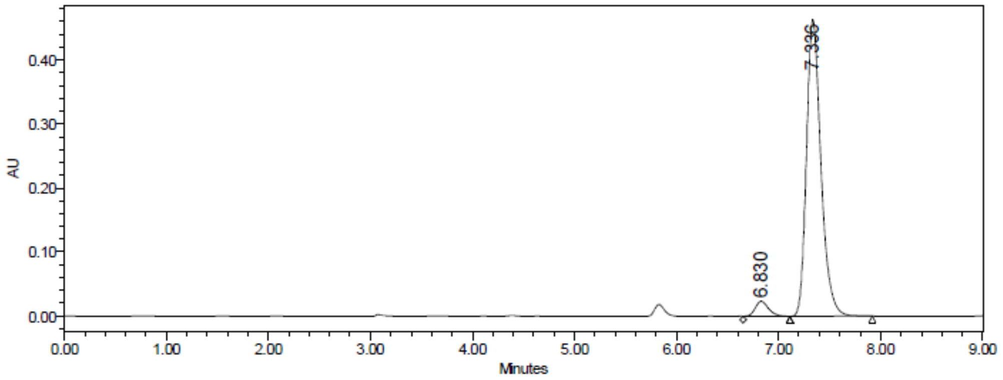

	RT	Area	% Area	Height
1	6.830	214092	4.46	23525
2	7.336	4580863	95.54	462959

rac-**5l**

SAMPLE INFORMATION

Sample Name:	zjy-9-363(322)-5%-IA-RAC	Acquired By:	System
Sample Type:	Unknown	Sample Set Name:	0
Vial:	49	Acq. Method Set:	5%quanbo
Injection #:	1	Processing Method:	LC PQ
Injection Volume:	10.00 ul	Channel Name:	240.0nm
Run Time:	8.0 Minutes	Proc. Chnl. Descr.:	2998 PDA 240.0 nm (2998
Date Acquired:	10/27/2022 2:10:30 PM CST		
Date Processed:	12/31/2022 11:34:20 AM CST		

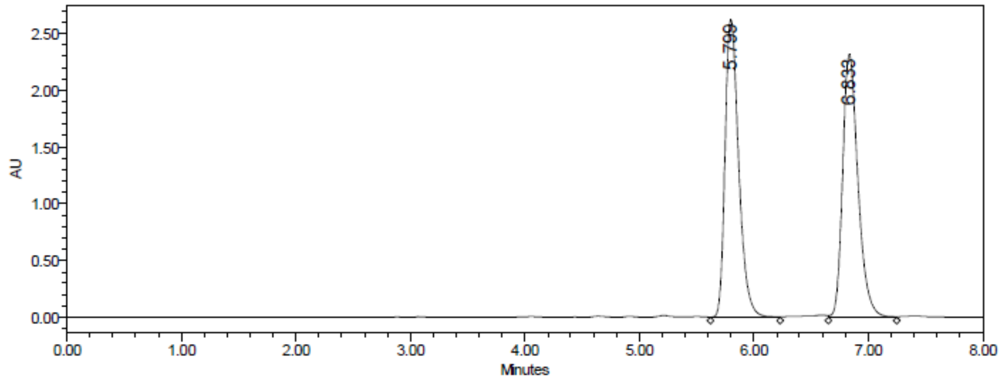

	RT	Area	% Area	Height
1	5.799	21824057	50.26	2619948
2	6.833	21597876	49.74	2312804

asy-**5l**

SAMPLE INFORMATION

Sample Name:	zjy-9-363-5%-IA-asy	Acquired By:	System
Sample Type:	Unknown	Sample Set Name:	0
Vial:	72	Acq. Method Set:	5%quanbo
Injection #:	1	Processing Method:	ZJY 5 113
Injection Volume:	10.00 ul	Channel Name:	240.0nm
Run Time:	8.0 Minutes	Proc. Chnl. Descr.:	2998 PDA 240.0 nm (2998
Date Acquired:	10/27/2022 2:01:49 PM CST		
Date Processed:	12/31/2022 11:32:20 AM CST		

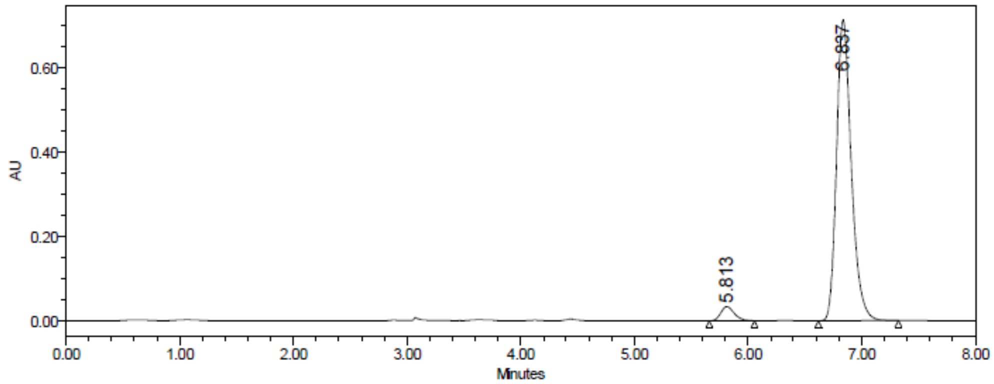

	RT	Area	% Area	Height
1	5.813	278695	4.14	33718
2	6.837	6454035	95.86	712266

rac-**5m**

SAMPLE INFORMATION

Sample Name:	zjy-9-361(306)-5%-IC-RAC	Acquired By:	System
Sample Type:	Unknown	Sample Set Name:	0
Vial:	53	Acq. Method Set:	5%quanbo
Injection #:	1	Processing Method:	LC PQ
Injection Volume:	10.00 ul	Channel Name:	240.0nm
Run Time:	12.0 Minutes	Proc. Chnl. Descr.:	2998 PDA 240.0 nm (2998
Date Acquired:	10/27/2022 3:07:39 PM CST		
Date Processed:	12/31/2022 11:23:23 AM CST		

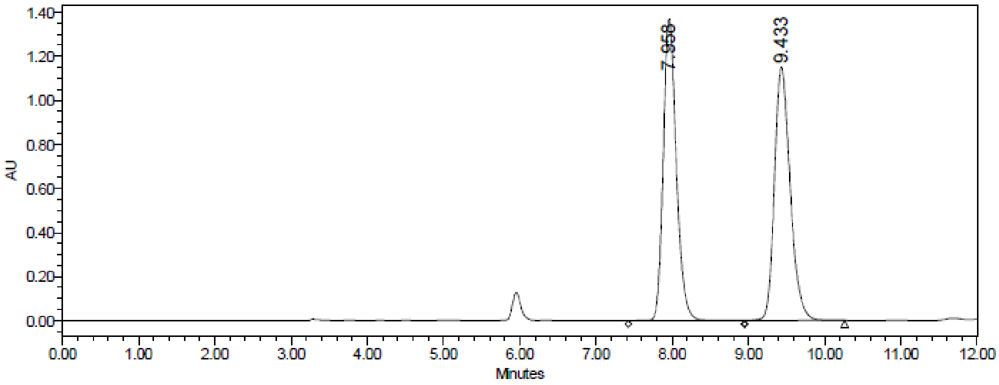

	RT	Area	% Area	Height
1	7.958	16247378	49.64	1364891
2	9.433	16481774	50.36	1149572

asy-**5m**

SAMPLE INFORMATION

Sample Name:	zjy-9-361-5%-IC-asy	Acquired By:	System
Sample Type:	Unknown	Sample Set Name:	0
Vial:	52	Acq. Method Set:	5%quanbo
Injection #:	1	Processing Method:	132
Injection Volume:	10.00 ul	Channel Name:	240.0nm
Run Time:	12.0 Minutes	Proc. Chnl. Descr.:	2998 PDA 240.0 nm (2998
Date Acquired:	10/27/2022 2:54:57 PM CST		
Date Processed:	12/31/2022 11:21:23 AM CST		

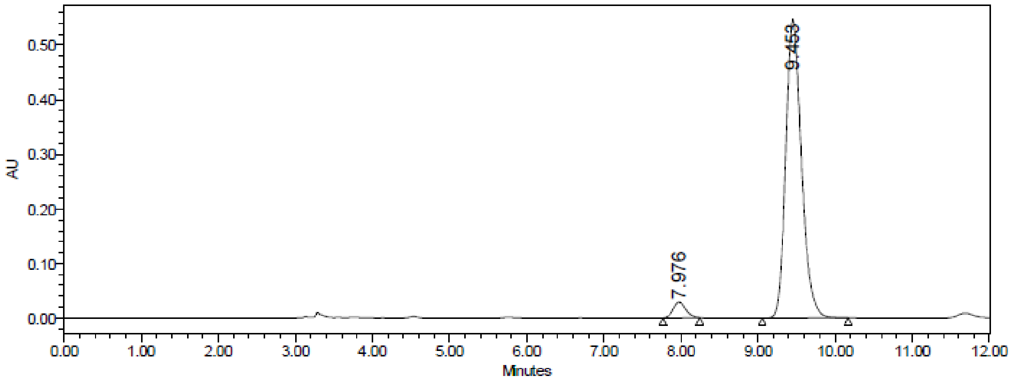

	RT	Area	% Area	Height
1	7.976	330060	4.08	28985
2	9.453	7754943	95.92	546244

rac-**5n**

SAMPLE INFORMATION

Sample Name:	zjy-9-362(329)-5%-IC-RAC	Acquired By:	System
Sample Type:	Unknown	Sample Set Name:	0
Vial:	62	Acq. Method Set:	5%quanbo
Injection #:	1	Processing Method:	54165463
Injection Volume:	10.00 ul	Channel Name:	254.0nm
Run Time:	26.0 Minutes	Proc. Chnl. Descr.:	2998 PDA 254.0 nm (2998
Date Acquired:	10/27/2022 11:03:20 AM CST		
Date Processed:	12/31/2022 4:01:38 PM CST		

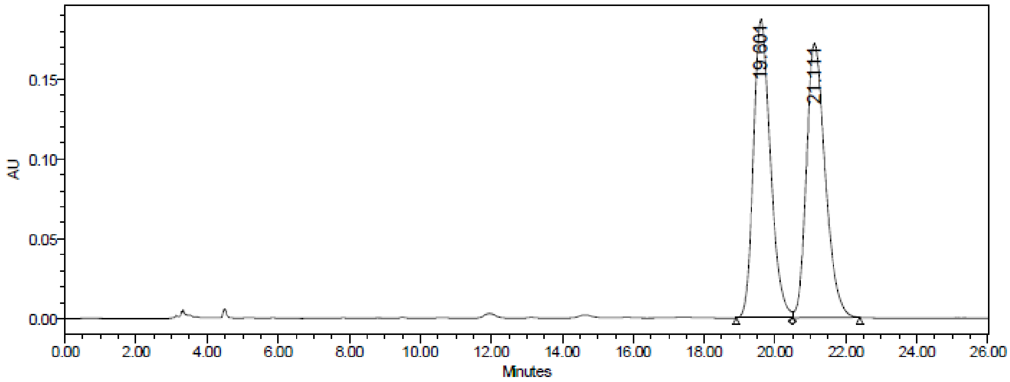

	RT	Area	% Area	Height
1	19.601	6255745	49.89	187000
2	21.111	6282527	50.11	171947

asy-**5n**

SAMPLE INFORMATION

Sample Name:	zjy-9-362-5%-IC-asy	Acquired By:	System
Sample Type:	Unknown	Sample Set Name:	0
Vial:	61	Acq. Method Set:	5%quanbo
Injection #:	1	Processing Method:	104
Injection Volume:	10.00 ul	Channel Name:	254.0nm
Run Time:	26.0 Minutes	Proc. Chnl. Descr.:	2998 PDA 254.0 nm (2998
Date Acquired:	10/27/2022 10:35:53 AM CST		
Date Processed:	12/31/2022 3:59:09 PM CST		

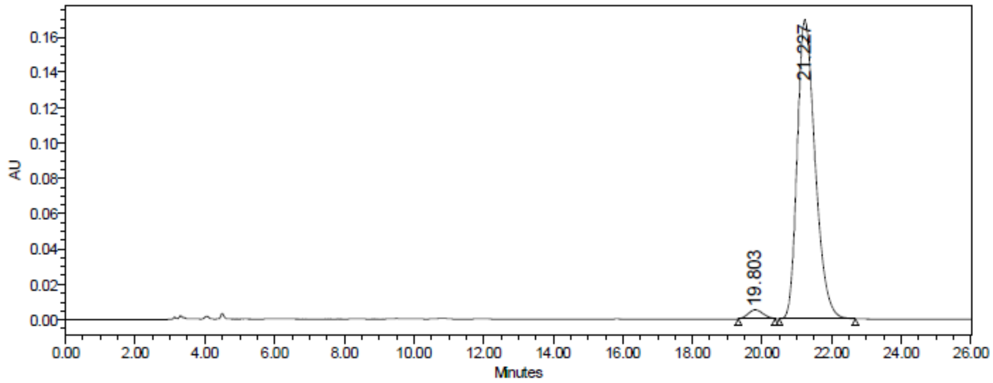

	RT	Area	% Area	Height
1	19.803	147532	2.40	5049
2	21.227	6001963	97.60	169191

rac-**5o**

SAMPLE INFORMATION

Sample Name:	zjy-9-360(309)-5%-IA-RAC	Acquired By:	System
Sample Type:	Unknown	Sample Set Name:	0
Vial:	71	Acq. Method Set:	5%quanbo
Injection #:	1	Processing Method:	54165463
Injection Volume:	10.00 ul	Channel Name:	240.0nm
Run Time:	9.0 Minutes	Proc. Chnl. Descr.:	2998 PDA 240.0 nm (2998
Date Acquired:	10/27/2022 1:52:07 PM CST		
Date Processed:	12/31/2022 3:23:44 PM CST		

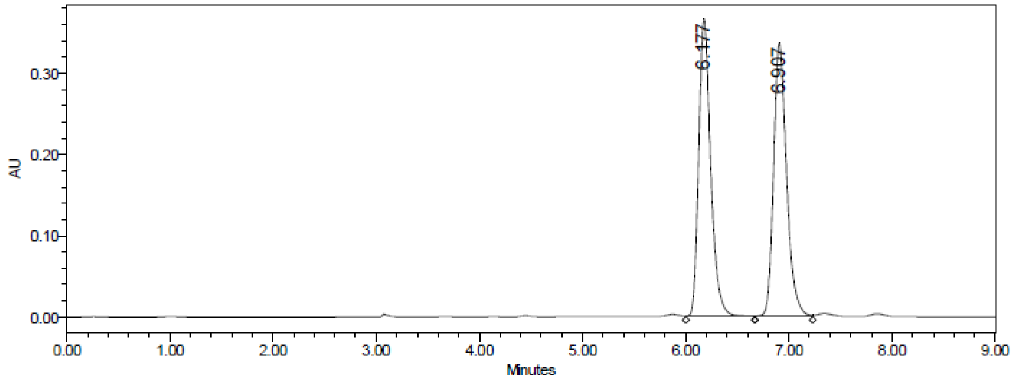

	RT	Area	% Area	Height
1	6.177	2975865	49.98	365899
2	6.907	2978035	50.02	336782

asy-**5o**

SAMPLE INFORMATION

Sample Name:	zjy-9-360-5%-IA-asy	Acquired By:	System
Sample Type:	Unknown	Sample Set Name:	0
Vial:	70	Acq. Method Set:	5%quanbo
Injection #:	1	Processing Method:	54165463
Injection Volume:	10.00 ul	Channel Name:	240.0nm
Run Time:	9.0 Minutes	Proc. Chnl. Descr.:	2998 PDA 240.0 nm (2998
Date Acquired:	10/27/2022 1:42:24 PM CST		
Date Processed:	12/31/2022 3:20:26 PM CST		

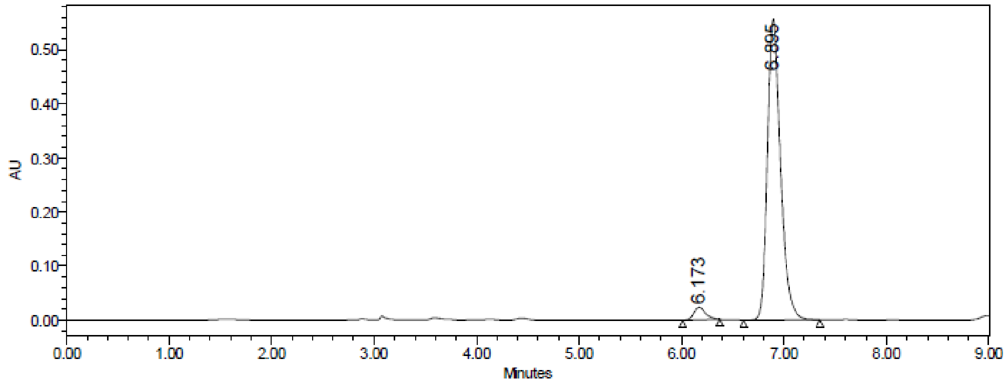

	RT	Area	% Area	Height
1	6.173	181509	3.53	22866
2	6.895	4960889	96.47	555760

rac-**5p**

SAMPLE INFORMATION

Sample Name:	zjy-9-319(308)-5%-IA-RAC	Acquired By:	System
Sample Type:	Unknown	Sample Set Name:	0
Vial:	49	Acq. Method Set:	5%quanbo
Injection #:	1	Processing Method:	1231
Injection Volume:	5.00 ul	Channel Name:	254.0nm@1
Run Time:	12.0 Minutes	Proc. Chnl. Descr.:	2998 PDA 254.0 nm (2998
Date Acquired:	10/15/2022 10:37:28 AM CST		
Date Processed:	12/31/2022 4:41:13 PM CST		

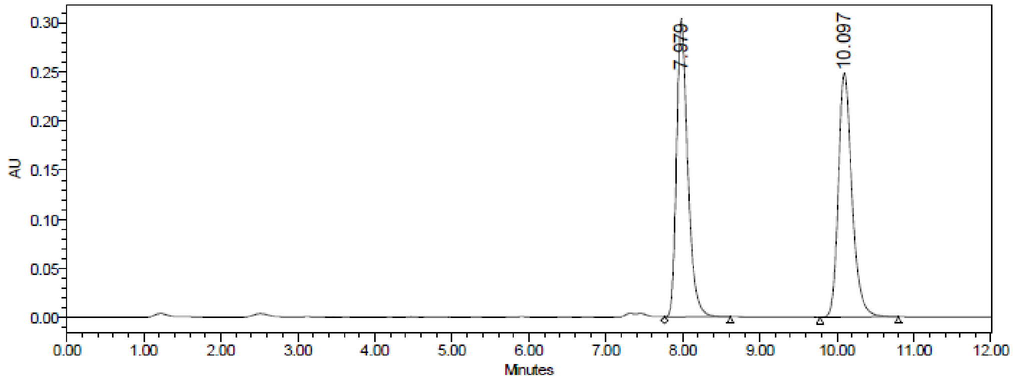

	RT	Area	% Area	Height
1	7.979	3076199	49.98	302809
2	10.097	3078524	50.02	247790

asy-**5p**

SAMPLE INFORMATION

Sample Name:	zjy-9-319-5%-IA-asy	Acquired By:	System
Sample Type:	Unknown	Sample Set Name:	0
Vial:	50	Acq. Method Set:	5%quanbo
Injection #:	1	Processing Method:	1231
Injection Volume:	5.00 ul	Channel Name:	240.0nm
Run Time:	12.0 Minutes	Proc. Chnl. Descr.:	2998 PDA 240.0 nm (2998
Date Acquired:	10/15/2022 10:50:07 AM CST		
Date Processed:	12/31/2022 4:39:57 PM CST		

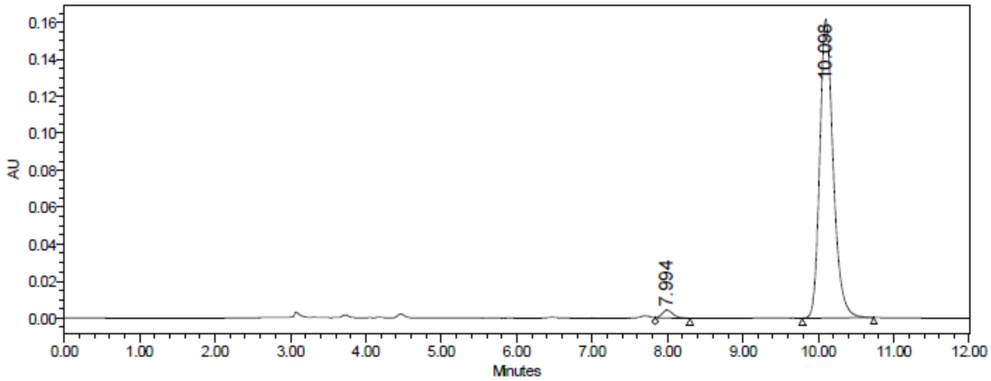

	RT	Area	% Area	Height
1	7.994	43674	2.12	4319
2	10.098	2013363	97.88	161486

rac-**5q**

SAMPLE INFORMATION

Sample Name:	zjy-9-320(317)-5%-IA-RAC	Acquired By:	System
Sample Type:	Unknown	Sample Set Name:	0
Vial:	51	Acq. Method Set:	5%quanbo
Injection #:	1	Processing Method:	1231
Injection Volume:	5.00 ul	Channel Name:	285.0nm
Run Time:	12.0 Minutes	Proc. Chnl. Descr.:	2998 PDA 285.0 nm (2998
Date Acquired:	10/15/2022 11:02:46 AM CST		
Date Processed:	12/31/2022 5:22:56 PM CST		

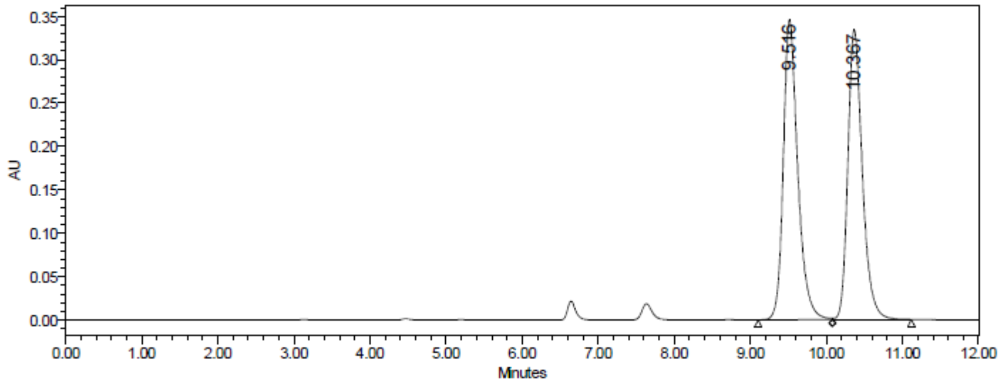

	RT	Area	% Area	Height
1	9.516	4572133	50.79	345810
2	10.367	4429814	49.21	334076

asy-**5q**

SAMPLE INFORMATION

Sample Name:	zjy-9-320-5%-IA-asy	Acquired By:	System
Sample Type:	Unknown	Sample Set Name:	0
Vial:	52	Acq. Method Set:	5%quanbo
Injection #:	1	Processing Method:	1231
Injection Volume:	5.00 ul	Channel Name:	285.0nm
Run Time:	12.0 Minutes	Proc. Chnl. Descr.:	2998 PDA 285.0 nm (2998
Date Acquired:	10/15/2022 11:15:26 AM CST		
Date Processed:	12/31/2022 5:21:26 PM CST		

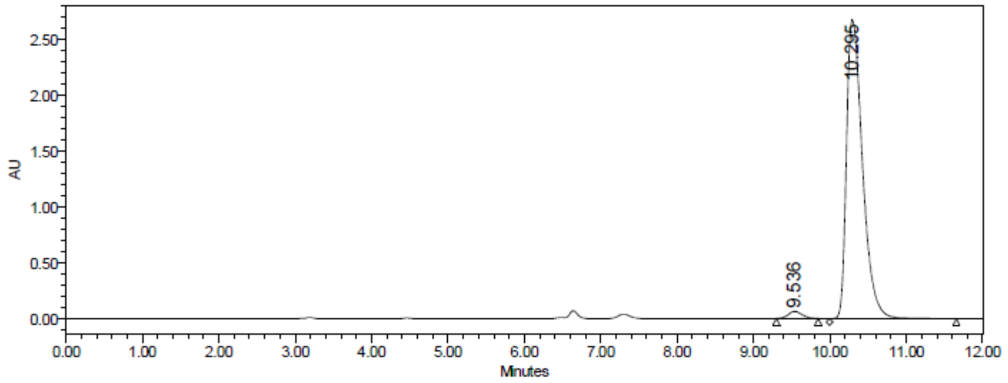

	RT	Area	% Area	Height
1	9.536	816061	2.04	64112
2	10.295	39104308	97.96	2673051

rac-**5r**

SAMPLE INFORMATION

Sample Name:	zjy-9-42-5%-IA-RAC	Acquired By:	System
Sample Type:	Unknown	Sample Set Name:	0
Vial:	55	Acq. Method Set:	5%quanbo
Injection #:	1	Processing Method:	1231
Injection Volume:	1.00 ul	Channel Name:	249.0nm
Run Time:	12.0 Minutes	Proc. Chnl. Descr.:	2998 PDA 249.0 nm (2998
Date Acquired:	7/22/2022 10:55:25 PM CST		
Date Processed:	12/31/2022 5:01:16 PM CST		

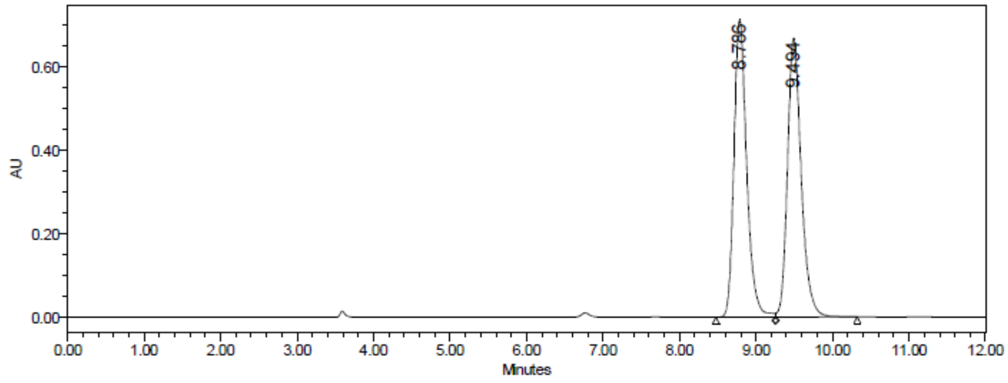

	RT	Area	% Area	Height
1	8.786	8173644	49.53	711471
2	9.494	8327894	50.47	667235

asy-**5r**

SAMPLE INFORMATION

Sample Name:	zjy-9-42-5%-IA-asy	Acquired By:	System
Sample Type:	Unknown	Sample Set Name:	0
Vial:	53	Acq. Method Set:	5%quanbo
Injection #:	1	Processing Method:	1231
Injection Volume:	10.00 ul	Channel Name:	249.0nm
Run Time:	12.0 Minutes	Proc. Chnl. Descr.:	2998 PDA 249.0 nm (2998
Date Acquired:	7/22/2022 10:25:53 PM CST		
Date Processed:	12/31/2022 4:59:42 PM CST		

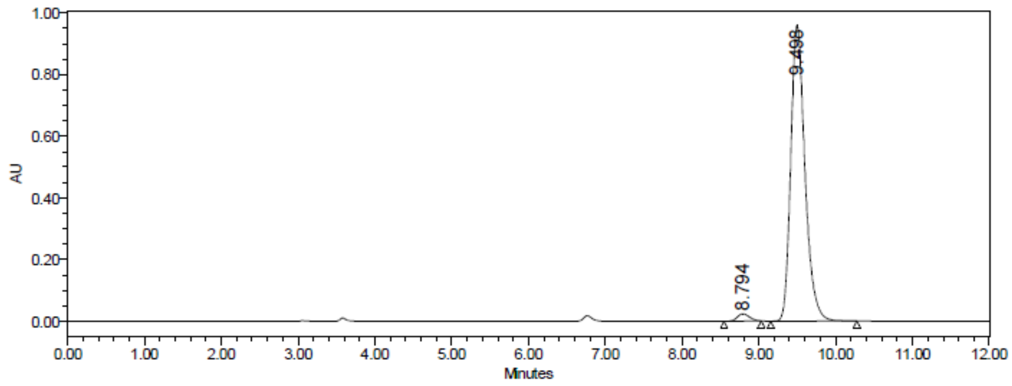

	RT	Area	% Area	Height
1	8.794	263993	2.09	23853
2	9.498	12339622	97.91	958480

rac-**5s**

SAMPLE INFORMATION

Sample Name:	zjy-9-340(307)-5%-IA-RAC	Acquired By:	System
Sample Type:	Unknown	Sample Set Name:	0
Vial:	55	Acq. Method Set:	5%quanbo
Injection #:	1	Processing Method:	1354685746
Injection Volume:	10.00 ul	Channel Name:	235.0nm
Run Time:	7.5 Minutes	Proc. Chnl. Descr.:	2998 PDA 235.0 nm (2998
Date Acquired:	10/25/2022 4:31:51 PM CST		
Date Processed:	12/31/2022 4:13:11 PM CST		

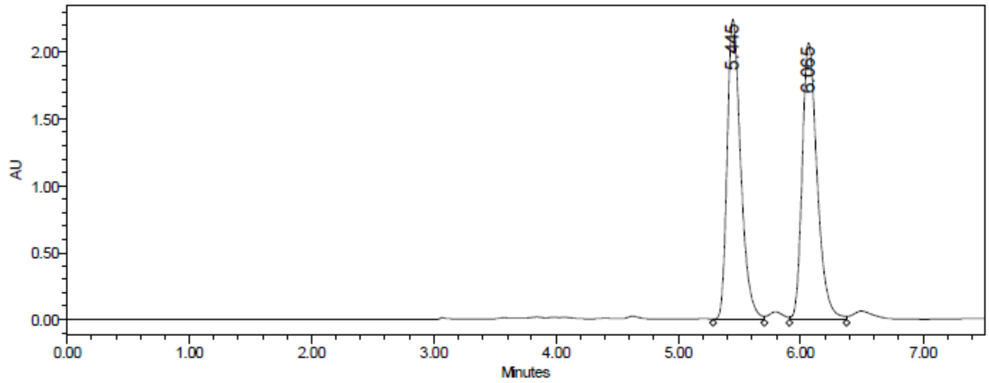

	RT	Area	% Area	Height
1	5.445	17367995	49.72	2242496
2	6.065	17561660	50.28	2068190

asy-**5s**

SAMPLE INFORMATION

Sample Name:	zjy-9-345-5%-IA-asy	Acquired By:	System
Sample Type:	Unknown	Sample Set Name:	0
Vial:	57	Acq. Method Set:	5%quanbo
Injection #:	1	Processing Method:	54165463
Injection Volume:	10.00 ul	Channel Name:	235.0nm
Run Time:	7.5 Minutes	Proc. Chnl. Descr.:	2998 PDA 235.0 nm (2998
Date Acquired:	10/25/2022 4:48:13 PM CST		
Date Processed:	12/31/2022 4:12:02 PM CST		

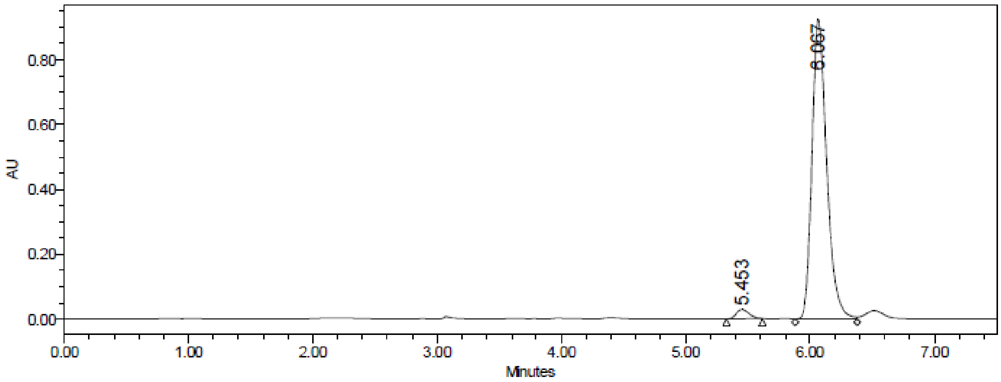

	RT	Area	% Area	Height
1	5.453	202956	2.60	28685
2	6.067	7601448	97.40	924533

rac-**5t**

SAMPLE INFORMATION

Sample Name:	zjy-9-321(318)-5%-IA-RAC	Acquired By:	System
Sample Type:	Unknown	Sample Set Name:	0
Vial:	53	Acq. Method Set:	5%quanbo
Injection #:	1	Processing Method:	1231
Injection Volume:	5.00 ul	Channel Name:	280.0nm
Run Time:	11.0 Minutes	Proc. Chnl. Descr.:	2998 PDA 280.0 nm (2998
Date Acquired:	10/15/2022 11:28:06 AM CST		
Date Processed:	12/31/2022 4:33:29 PM CST		

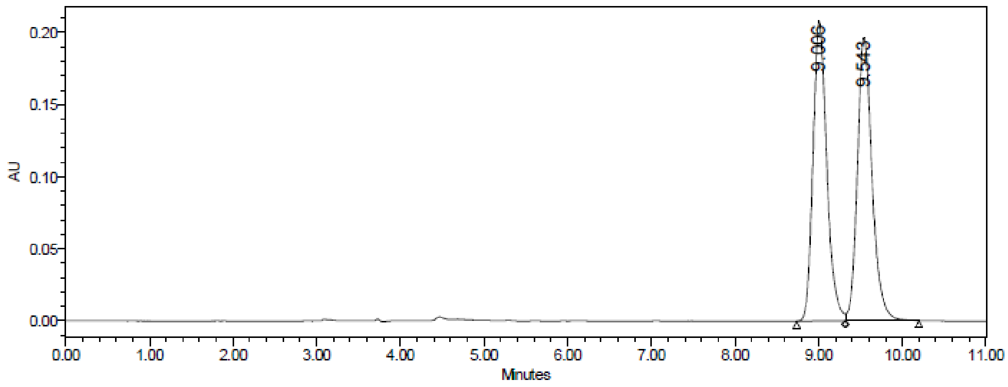

	RT	Area	% Area	Height
1	9.006	2364420	49.58	208239
2	9.543	2404825	50.42	196719

asy-**5t**

SAMPLE INFORMATION

Sample Name:	zjy-9-321-5%-IA-asy	Acquired By:	System
Sample Type:	Unknown	Sample Set Name:	0
Vial:	54	Acq. Method Set:	5%quanbo
Injection #:	1	Processing Method:	1231
Injection Volume:	5.00 ul	Channel Name:	280.0nm
Run Time:	11.0 Minutes	Proc. Chnl. Descr.:	2998 PDA 280.0 nm (2998
Date Acquired:	10/15/2022 11:39:45 AM CST		
Date Processed:	12/31/2022 4:32:29 PM CST		

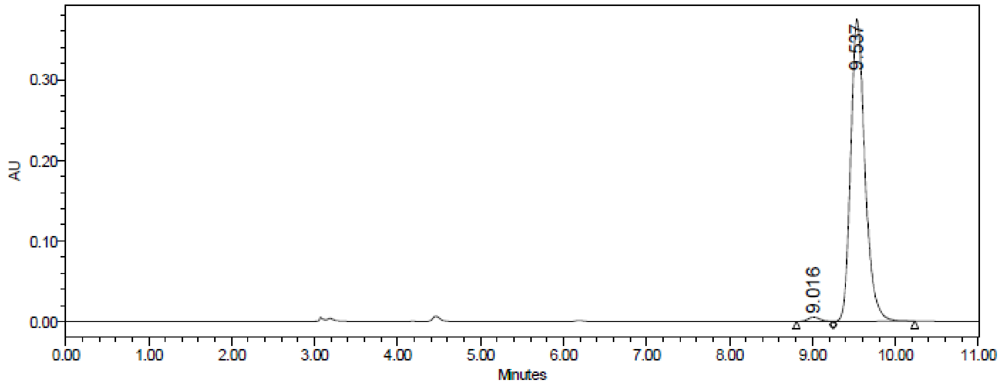

	RT	Area	% Area	Height
1	9.016	56856	1.25	5205
2	9.537	4495038	98.75	373510

rac-**5u**-*major isomer*

SAMPLE INFORMATION

Sample Name:	zjy-15-159(P2)-5%-IA-rac	Acquired By:	System
Sample Type:	Unknown	Sample Set Name:	
Vial:	52	Acq. Method Set:	5%quanbo
Injection #:	1	Processing Method:	54165463
Injection Volume:	10.00 ul	Channel Name:	245.0nm
Run Time:	60.0 Minutes	Proc. Chnl. Descr.:	2998 PDA 245.0 nm (2998
Date Acquired:	12/26/2024 9:55:39 AM CST		
Date Processed:	1/3/2025 10:50:50 PM CST		

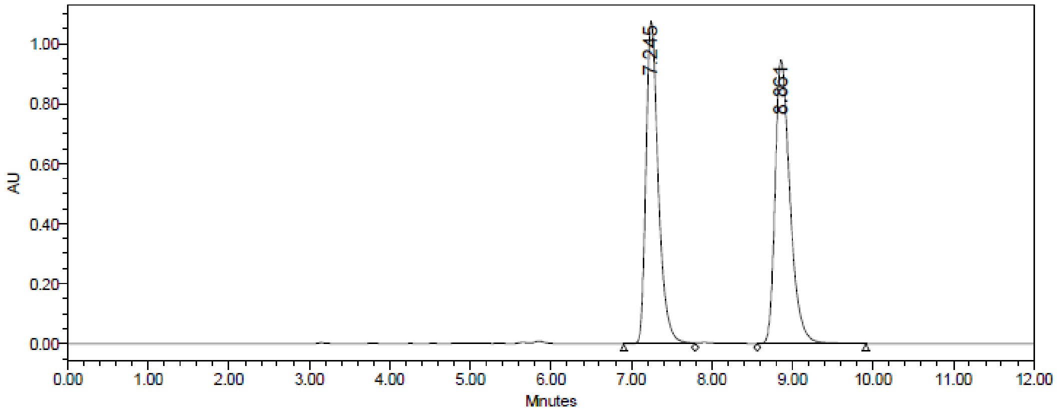

	RT	Area	% Area	Height
1	7.245	11453987	48.51	1072846
2	8.861	12159024	51.49	942820

asy-**5u**-*major isomer*

SAMPLE INFORMATION

Sample Name:	zjy-15-150(P2)-5%-IA-asy	Acquired By:	System
Sample Type:	Unknown	Sample Set Name:	0
Vial:	57	Acq. Method Set:	5%quanbo
Injection #:	1	Processing Method:	54165463
Injection Volume:	10.00 ul	Channel Name:	245.0nm
Run Time:	12.0 Minutes	Proc. Chnl. Descr.:	2998 PDA 245.0 nm (2998
Date Acquired:	12/26/2024 4:20:43 PM CST		
Date Processed:	1/3/2025 10:49:28 PM CST		

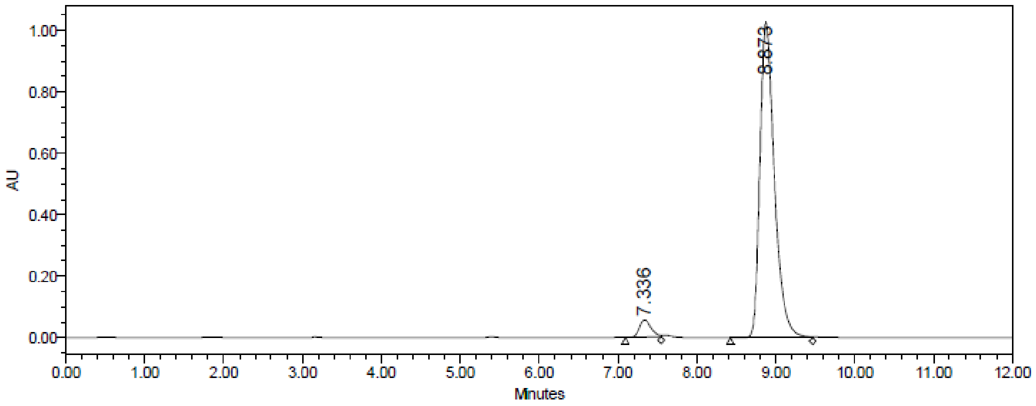

	RT	Area	% Area	Height
1	7.336	554571	4.07	55937
2	8.873	13074899	95.93	1025891

rac-**5u**-*minor isomer*

SAMPLE INFORMATION

Sample Name:	zjy-15-159(P1)-10%-IG-rac	Acquired By:	System
Sample Type:	Unknown	Sample Set Name:	0
Vial:	58	Acq. Method Set:	10%quanbo
Injection #:	1	Processing Method:	132
Injection Volume:	2.00 ul	Channel Name:	254.0nm
Run Time:	14.0 Minutes	Proc. Chnl. Descr.:	2998 PDA 254.0 nm (2998
Date Acquired:	12/27/2024 12:21:55 PM CST		
Date Processed:	1/3/2025 10:28:15 PM CST		

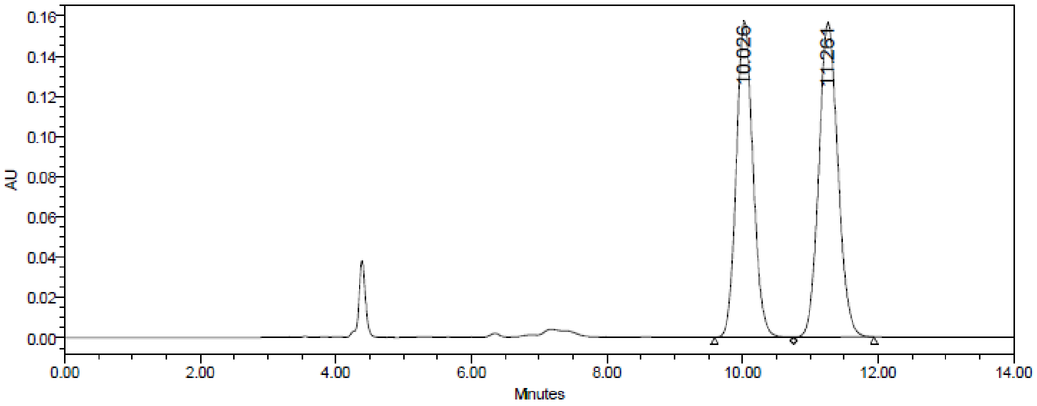

	RT	Area	% Area	Height
1	10.026	2677530	46.71	156966
2	11.261	3054862	53.29	156267

asy-**5u**-*minor isomer*

SAMPLE INFORMATION

Sample Name:	zjy-15-150(P1)-10%-IG-asy	Acquired By:	System
Sample Type:	Unknown	Sample Set Name:	0
Vial:	56	Acq. Method Set:	10%quanbo
Injection #:	1	Processing Method:	54165463
Injection Volume:	10.00 ul	Channel Name:	254.0nm
Run Time:	14.0 Minutes	Proc. Chnl. Descr.:	2998 PDA 254.0 nm (2998
Date Acquired:	12/27/2024 11:52:37 AM CST		
Date Processed:	1/3/2025 10:30:21 PM CST		

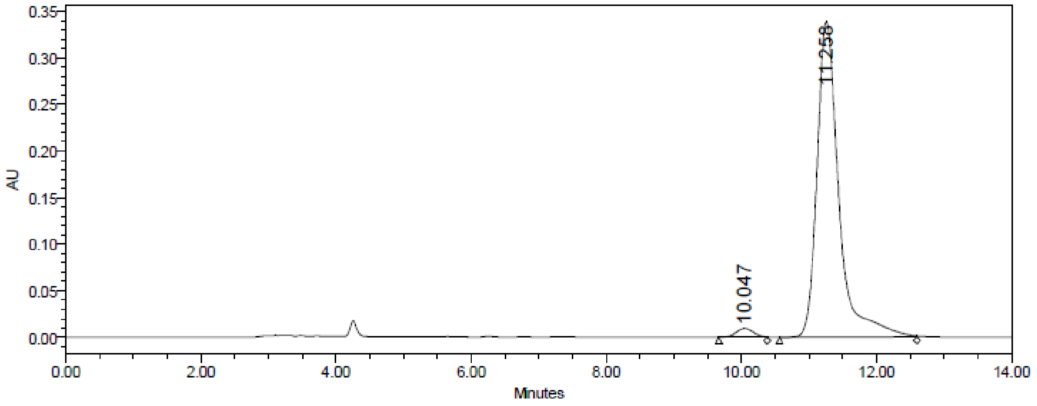

	RT	Area	% Area	Height
1	10.047	166791	2.15	9467
2	11.258	7587814	97.85	338737

rac-**5v**-*major isomer*

SAMPLE INFORMATION

Sample Name:	zjy-15-167(P2)-10%-IG-rac	Acquired By:	System
Sample Type:	Unknown	Sample Set Name:	
Vial:	56	Acq. Method Set:	10%quanbo
Injection #:	1	Processing Method:	54165463
Injection Volume:	5.00 ul	Channel Name:	240.0nm
Run Time:	60.0 Minutes	Proc. Chnl. Descr.:	2998 PDA 240.0 nm (2998
Date Acquired:	12/27/2024 10:15:55 AM CST		
Date Processed:	1/3/2025 10:35:40 PM CST		

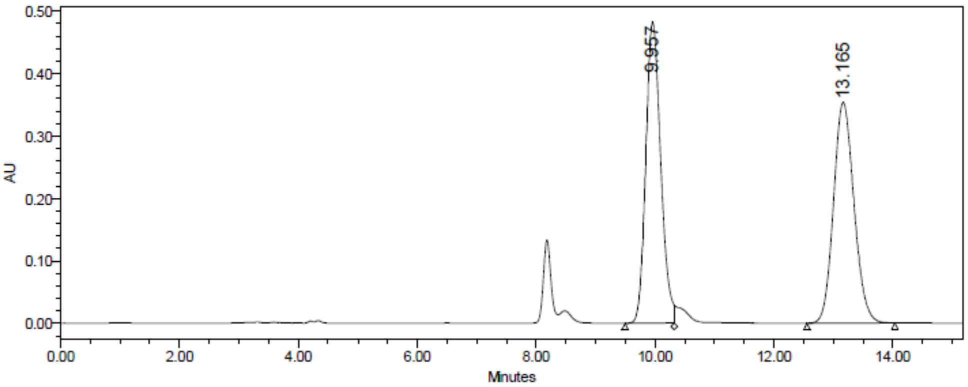

	RT	Area	% Area	Height
1	9.957	8356751	49.69	481815
2	13.165	8462519	50.31	353660

asy-**5v**-*major isomer*

SAMPLE INFORMATION

Sample Name:	zjy-15-168(P2)-10%-IG-asy	Acquired By:	System
Sample Type:	Unknown	Sample Set Name:	
Vial:	57	Acq. Method Set:	10%quanbo
Injection #:	1	Processing Method:	1354685746
Injection Volume:	5.00 ul	Channel Name:	240.0nm
Run Time:	60.0 Minutes	Proc. Chnl. Descr.:	2998 PDA 240.0 nm (2998
Date Acquired:	12/27/2024 10:32:33 AM CST		
Date Processed:	1/3/2025 10:34:35 PM CST		

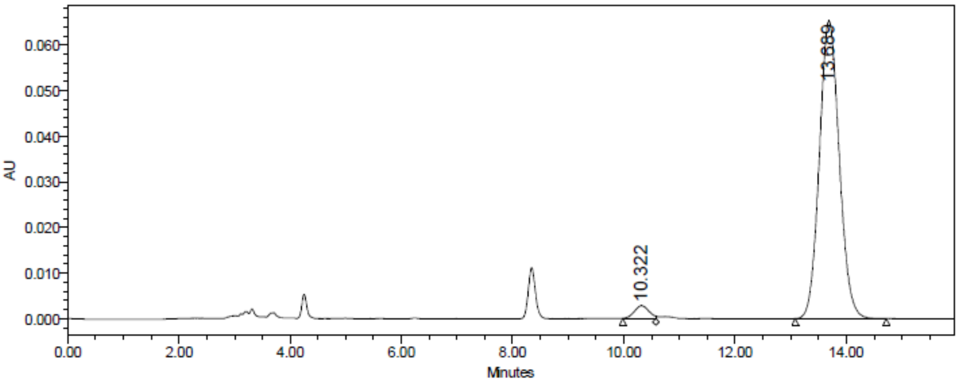

	RT	Area	% Area	Height
1	10.322	49790	2.97	2784
2	13.689	1624674	97.03	65295

rac-**5v**-*minor isomer*

SAMPLE INFORMATION

Sample Name:	zjy-15-167(P1)-10%-IE-rac	Acquired By:	System
Sample Type:	Unknown	Sample Set Name:	
Vial:	56	Acq. Method Set:	10%quanbo
Injection #:	1	Processing Method:	54165463
Injection Volume:	5.00 ul	Channel Name:	240.0nm
Run Time:	60.0 Minutes	Proc. Chnl. Descr.:	2998 PDA 240.0 nm (2998
Date Acquired:	12/26/2024 10:31:17 PM CST		
Date Processed:	1/3/2025 10:38:06 PM CST		

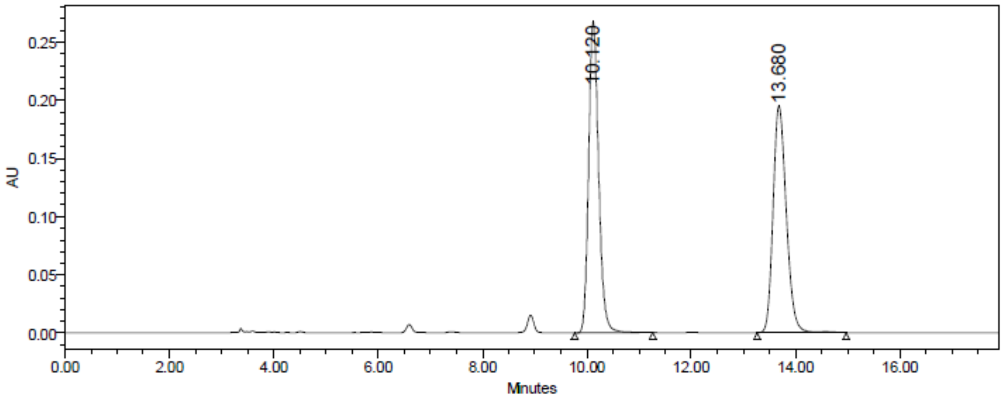

	RT	Area	% Area	Height
1	10.120	3539956	50.56	267503
2	13.680	3461574	49.44	194953

asy-**5v**-*minor isomer*

SAMPLE INFORMATION

Sample Name:	zjy-15-168(P1)-10%-IE-asy	Acquired By:	System
Sample Type:	Unknown	Sample Set Name:	0
Vial:	58	Acq. Method Set:	10%quanbo
Injection #:	1	Processing Method:	54165463
Injection Volume:	10.00 ul	Channel Name:	240.0nm
Run Time:	16.0 Minutes	Proc. Chnl. Descr.:	2998 PDA 240.0 nm (2998
Date Acquired:	12/26/2024 11:19:28 PM CST		
Date Processed:	1/3/2025 10:36:41 PM CST		

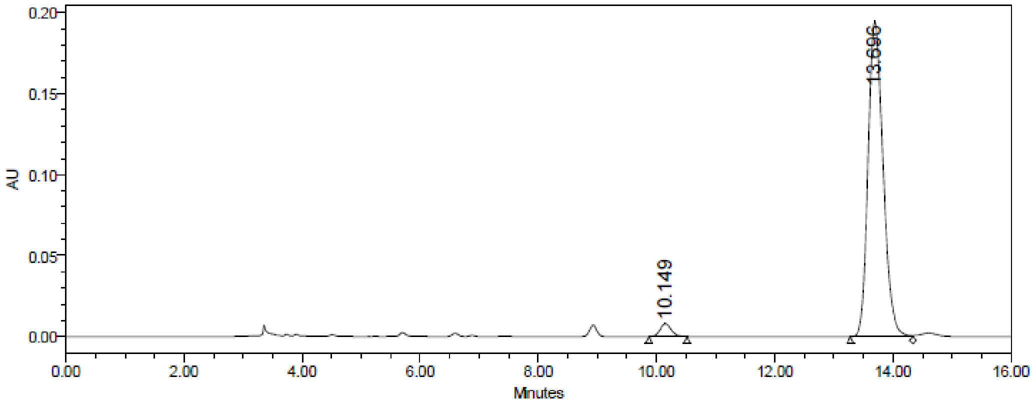

	RT	Area	% Area	Height
1	10.149	104894	2.95	7898
2	13.696	3454298	97.05	194531

rac-**5w**

SAMPLE INFORMATION

Sample Name:	zjy-9-251-5%-IA-RAC	Acquired By:	System
Sample Type:	Unknown	Sample Set Name:	0
Vial:	51	Acq. Method Set:	5%quanbo
Injection #:	1	Processing Method:	0
Injection Volume:	10.00 ul	Channel Name:	266.0nm
Run Time:	17.0 Minutes	Proc. Chnl. Descr.:	2998 PDA 266.0 nm (2998
Date Acquired:	9/22/2022 9:28:53 PM CST		
Date Processed:	10/27/2022 9:36:42 PM CST		

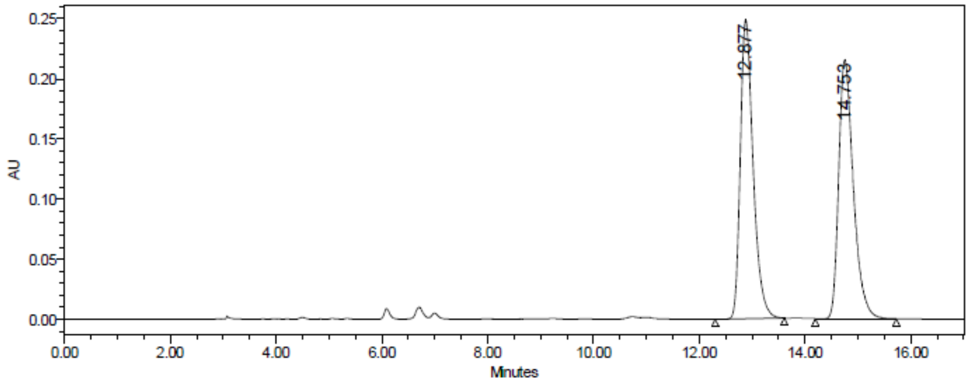

	RT	Area	% Area	Height
1	12.877	4205520	50.32	248721
2	14.753	4152665	49.68	215245

asy-**5w**

SAMPLE INFORMATION

Sample Name:	zjy-9-251-5%-IA-asy	Acquired By:	System
Sample Type:	Unknown	Sample Set Name:	0
Vial:	50	Acq. Method Set:	5%quanbo
Injection #:	1	Processing Method:	104
Injection Volume:	10.00 ul	Channel Name:	240.0nm
Run Time:	17.0 Minutes	Proc. Chnl. Descr.:	2998 PDA 240.0 nm (2998
Date Acquired:	9/22/2022 9:11:10 PM CST		
Date Processed:	10/27/2022 9:30:19 PM CST		

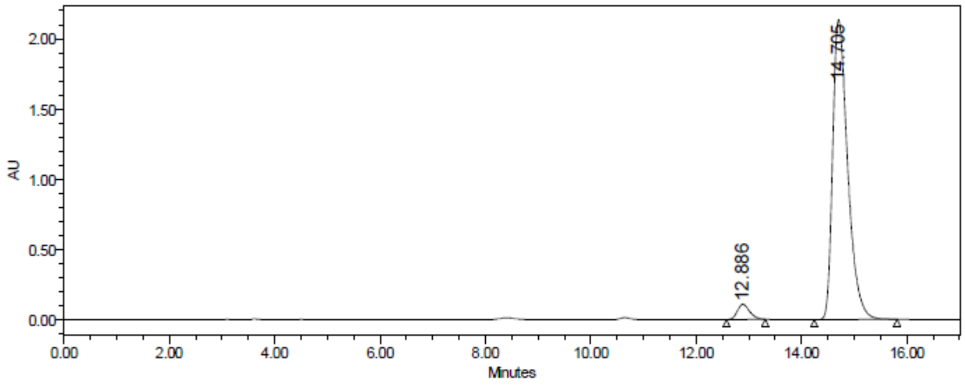

	RT	Area	% Area	Height
1	12.886	1752604	3.97	108698
2	14.705	42353524	96.03	2132316

rac-**5x**

SAMPLE INFORMATION

Sample Name:	zjy-9-252-5%-IA-RAC	Acquired By:	System
Sample Type:	Unknown	Sample Set Name:	0
Vial:	53	Acq. Method Set:	5%quanbo
Injection #:	1	Processing Method:	0
Injection Volume:	10.00 ul	Channel Name:	240.0nm
Run Time:	16.0 Minutes	Proc. Chnl. Descr.:	2998 PDA 240.0 nm (2998
Date Acquired:	9/22/2022 10:03:16 PM CST		
Date Processed:	10/27/2022 9:39:34 PM CST		

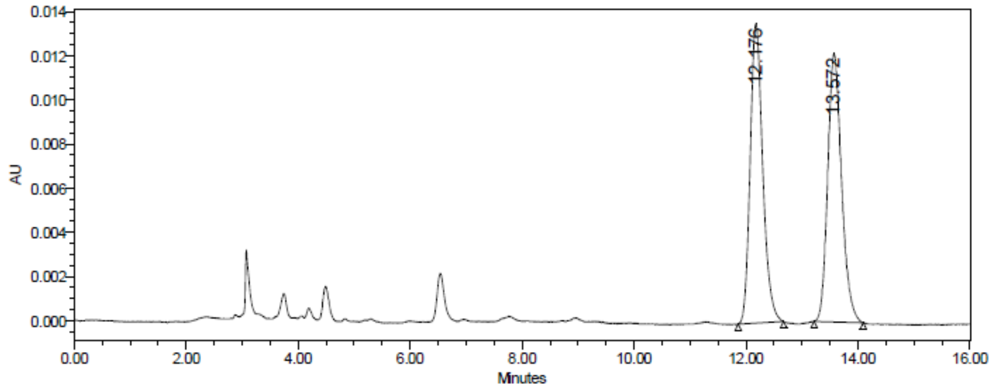

	RT	Area	% Area	Height
1	12.176	210376	50.15	13563
2	13.572	209107	49.85	12145

asy-**5x**

SAMPLE INFORMATION

Sample Name:	zjy-9-252-5%-IA-asy	Acquired By:	System
Sample Type:	Unknown	Sample Set Name:	0
Vial:	52	Acq. Method Set:	5%quanbo
Injection #:	1	Processing Method:	0
Injection Volume:	10.00 ul	Channel Name:	240.0nm
Run Time:	16.0 Minutes	Proc. Chnl. Descr.:	2998 PDA 240.0 nm (2998
Date Acquired:	9/22/2022 9:46:33 PM CST		
Date Processed:	10/27/2022 9:37:29 PM CST		

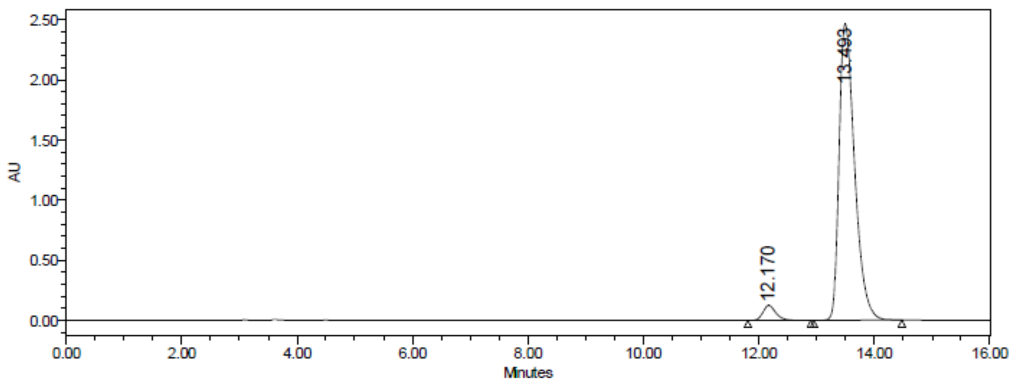

	RT	Area	% Area	Height
1	12.170	1938301	4.03	124551
2	13.493	46188032	95.97	2462421

rac-**5y**

SAMPLE INFORMATION

Sample Name:	zjy-9-243(30)-5%-IC-RAC	Acquired By:	System
Sample Type:	Unknown	Sample Set Name	0
Vial:	58	Acq. Method Set:	5% quanbo
Injection #:	1	Processing Method	zjy 5 122 asy
Injection Volume:	5.00 ul	Channel Name:	265.0nm
Run Time:	26.0 Minutes	Proc. Chnl. Descr.:	2998 PDA 265.0 nm (2998
Date Acquired:	9/16/2022 9:24:04 PM CST		
Date Processed:	10/27/2022 10:25:46 PM CST		

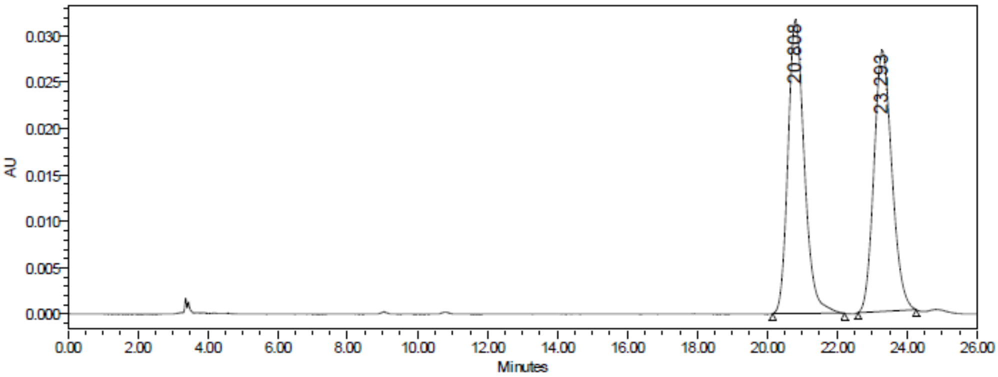

	RT	Area	% Area	Height
1	20.808	1029086	50.58	31684
2	23.293	1005507	49.42	28138

asy-**5y**

SAMPLE INFORMATION

Sample Name:	zjy-9-243-5%-IC-asy	Acquired By:	System
Sample Type:	Unknown	Sample Set Name	
Vial:	70	Acq. Method Set:	5% quanbo
Injection #:	1	Processing Method	Default
Injection Volume:	5.00 ul	Channel Name:	240.0nm
Run Time:	80.0 Minutes	Proc. Chnl. Descr.:	2998 PDA 240.0 nm (2998
Date Acquired:	9/16/2022 7:59:15 PM CST		
Date Processed:	10/27/2022 10:23:32 PM CST		

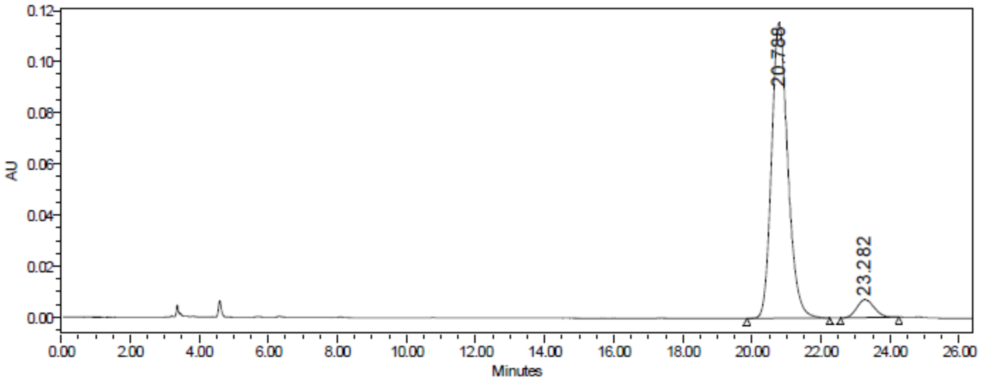

	RT	Area	% Area	Height
1	20.788	3745804	93.80	115590
2	23.282	247676	6.20	7091

rac-**5z**

SAMPLE INFORMATION

Sample Name:	zjy-13-125-20%-IC-rac	Acquired By:	System
Sample Type:	Unknown	Sample Set Name:	0
Vial:	59	Acq. Method Set:	20%quanbo
Injection #:	1	Processing Method:	54165463
Injection Volume:	10.00 ul	Channel Name:	254.0nm
Run Time:	24.0 Minutes	Proc. Chnl. Descr.:	2998 PDA 254.0 nm (2998
Date Acquired:	1/10/2024 5:44:06 PM CST		
Date Processed:	6/5/2024 3:56:08 PM CST		

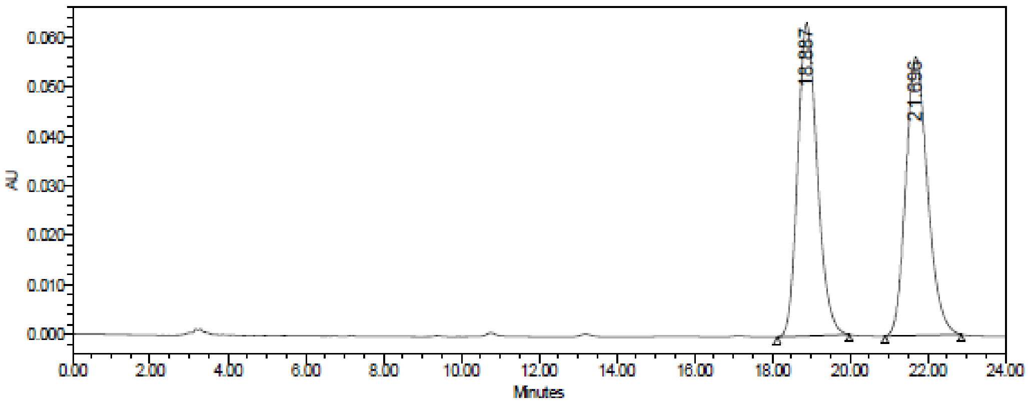

	RT	Area	% Area	Height
1	18.887	2204522	50.11	63223
2	21.696	2194585	49.89	56311

asy-**5z**

SAMPLE INFORMATION

Sample Name:	zjy-13-125-20%-IC-asy	Acquired By:	System
Sample Type:	Unknown	Sample Set Name:	0
Vial:	60	Acq. Method Set:	20%quanbo
Injection #:	1	Processing Method:	54165463
Injection Volume:	10.00 ul	Channel Name:	254.0nm
Run Time:	24.0 Minutes	Proc. Chnl. Descr.:	2998 PDA 254.0 nm (2998
Date Acquired:	1/10/2024 6:08:49 PM CST		
Date Processed:	6/5/2024 3:57:16 PM CST		

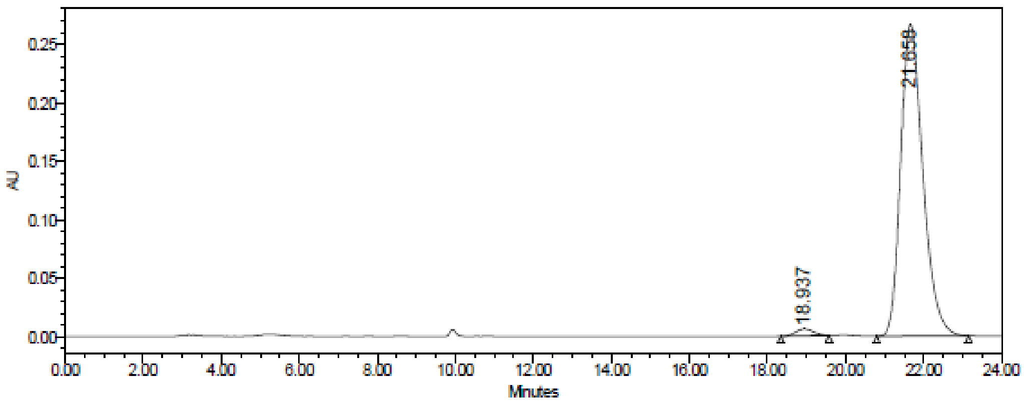

	RT	Area	% Area	Height
1	18.937	192543	1.80	5980
2	21.658	10483997	98.20	266468

rac-**5aa**

SAMPLE INFORMATION

Sample Name:	zjy-9-257(271)-3%-IA-RAC	Acquired By:	System
Sample Type:	Unknown	Sample Set Name:	0
Vial:	54	Acq. Method Set:	3%quanbo
Injection #:	1	Processing Method:	0
Injection Volume:	10.00 ul	Channel Name:	240.0nm
Run Time:	12.0 Minutes	Proc. Chnl. Descr.:	2998 PDA 240.0 nm (2998
Date Acquired:	9/22/2022 10:30:09 PM CST		
Date Processed:	10/27/2022 8:53:27 PM CST		

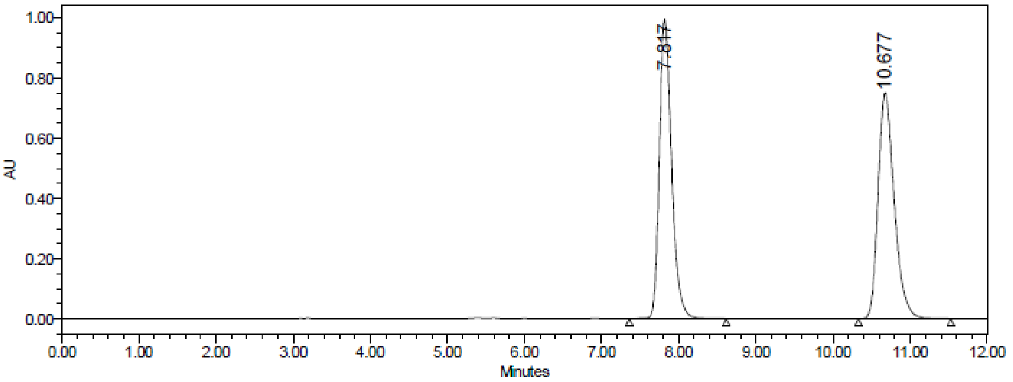

	RT	Area	% Area	Height
1	7.817	10902239	50.12	993486
2	10.677	10850416	49.88	750316

asy-**5aa**

SAMPLE INFORMATION

Sample Name:	zjy-9-271-3%-IA-asy	Acquired By:	System
Sample Type:	Unknown	Sample Set Name:	0
Vial:	63	Acq. Method Set:	3%quanbo
Injection #:	1	Processing Method:	0
Injection Volume:	10.00 ul	Channel Name:	240.0nm
Run Time:	12.0 Minutes	Proc. Chnl. Descr.:	2998 PDA 240.0 nm (2998
Date Acquired:	9/24/2022 9:51:39 PM CST		
Date Processed:	10/27/2022 8:58:10 PM CST		

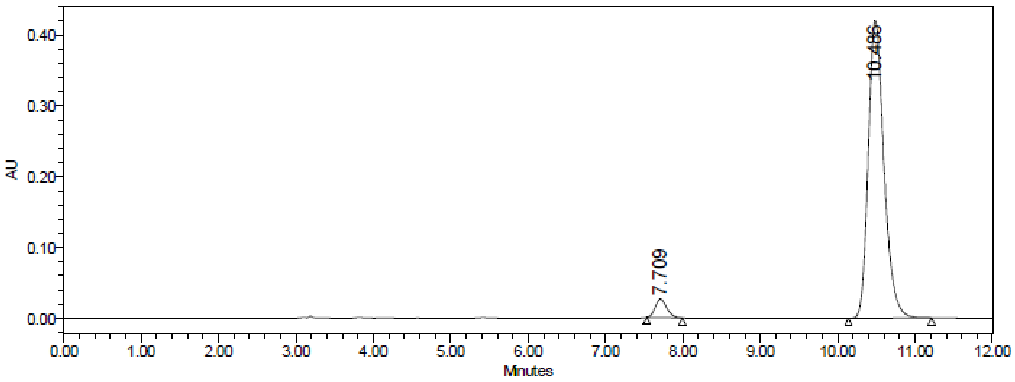

	RT	Area	% Area	Height
1	7.709	272051	4.41	26264
2	10.486	5903838	95.59	419962

rac-**5ab**

SAMPLE INFORMATION

Sample Name:	zjy-9-272-3%-IA-RAC	Acquired By:	System
Sample Type:	Unknown	Sample Set Name:	0
Vial:	50	Acq. Method Set:	3%quanbo
Injection #:	1	Processing Method:	54165463
Injection Volume:	5.00 ul	Channel Name:	240.0nm
Run Time:	11.0 Minutes	Proc. Chnl. Descr.:	2998 PDA 240.0 nm (2998
Date Acquired:	10/4/2022 10:10:12 PM CST		
Date Processed:	10/27/2022 9:44:26 PM CST		

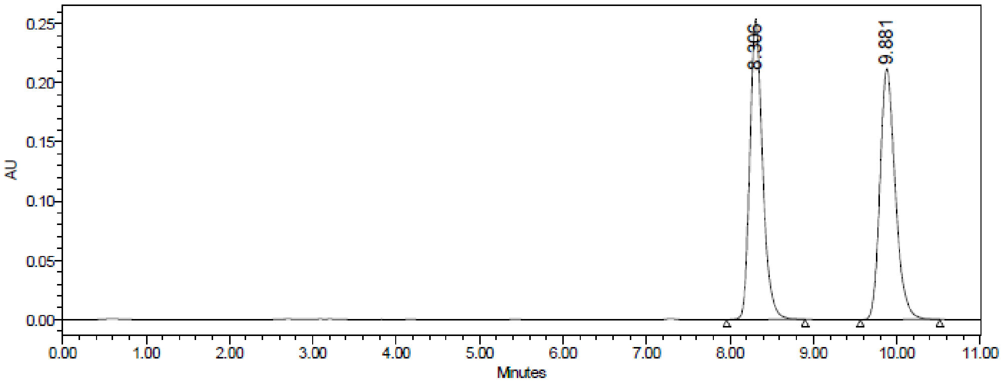

	RT	Area	% Area	Height
1	8.306	2710574	50.23	253332
2	9.881	2685853	49.77	211691

asy-**5ab**

SAMPLE INFORMATION

Sample Name:	zjy-9-272-3%-IA-asy	Acquired By:	System
Sample Type:	Unknown	Sample Set Name:	0
Vial:	49	Acq. Method Set:	3%quanbo
Injection #:	1	Processing Method:	1041
Injection Volume:	5.00 ul	Channel Name:	240.0nm
Run Time:	11.0 Minutes	Proc. Chnl. Descr.:	2998 PDA 240.0 nm (2998
Date Acquired:	10/4/2022 9:58:17 PM CST		
Date Processed:	10/27/2022 9:43:14 PM CST		

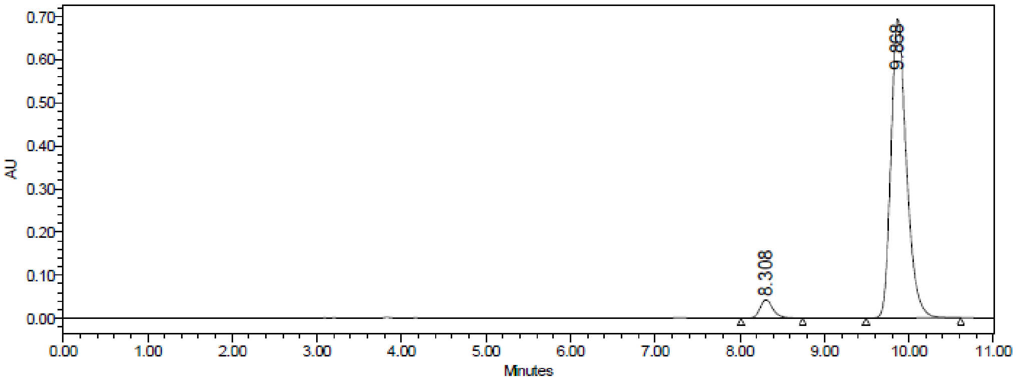

	RT	Area	% Area	Height
1	8.308	453635	4.89	42636
2	9.868	8832405	95.11	692111

rac-**5ac**

SAMPLE INFORMATION

Sample Name:	zjy-9-287(286)-5%-IA-RAC	Acquired By:	System
Sample Type:	Unknown	Sample Set Name:	0
Vial:	72	Acq. Method Set:	5%quanbo
Injection #:	1	Processing Method:	54165463
Injection Volume:	10.00 ul	Channel Name:	240.0nm
Run Time:	8.0 Minutes	Proc. Chnl. Descr.:	2998 PDA 240.0 nm (2998
Date Acquired:	10/15/2022 10:28:50 AM CST		
Date Processed:	10/27/2022 10:00:10 PM CST		

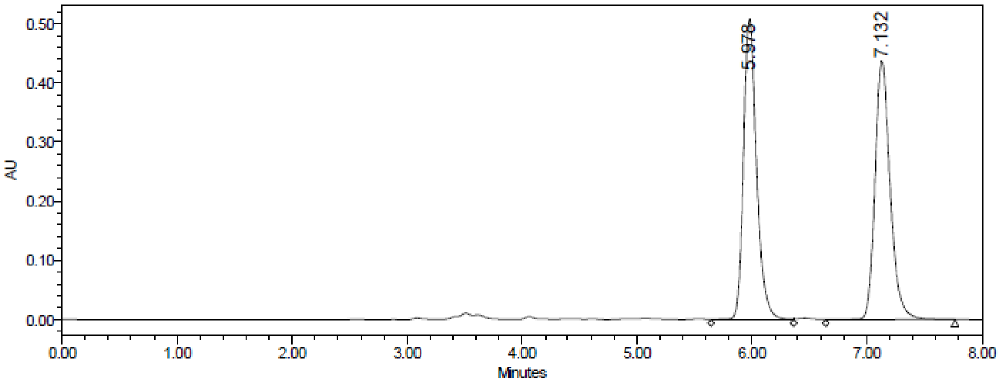

	RT	Area	% Area	Height
1	5.978	4009515	50.04	507141
2	7.132	4002776	49.96	435809

asy-**5ac**

SAMPLE INFORMATION

Sample Name:	zjy-9-287-5%-IA-asy	Acquired By:	System
Sample Type:	Unknown	Sample Set Name:	0
Vial:	71	Acq. Method Set:	5%quanbo
Injection #:	1	Processing Method:	1041
Injection Volume:	10.00 ul	Channel Name:	254.0nm
Run Time:	8.0 Minutes	Proc. Chnl. Descr.:	2998 PDA 254.0 nm (2998
Date Acquired:	10/15/2022 10:19:54 AM CST		
Date Processed:	10/27/2022 9:59:07 PM CST		

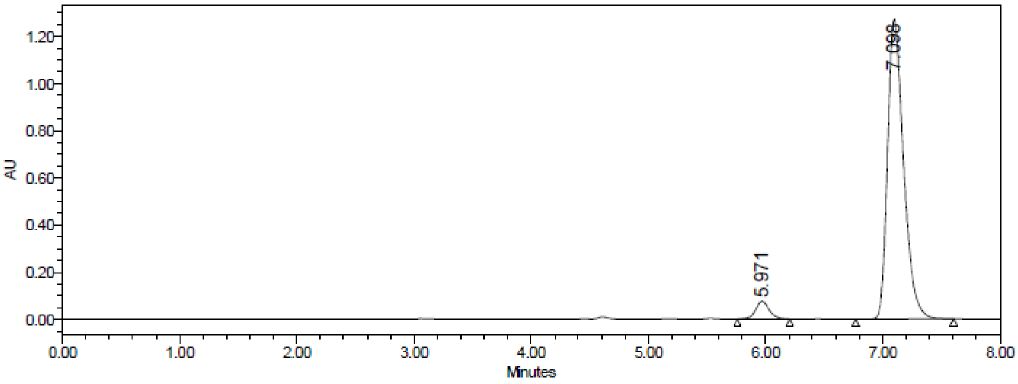

	RT	Area	% Area	Height
1	5.971	610149	4.82	75216
2	7.098	12050629	95.18	1270711

rac-**5ad**

SAMPLE INFORMATION

Sample Name:	zjy-9-273-5%-IA-RAC	Acquired By:	System
Sample Type:	Unknown	Sample Set Name:	0
Vial:	55	Acq. Method Set:	5%quanbo
Injection #:	1	Processing Method:	132
Injection Volume:	5.00 ul	Channel Name:	240.0nm
Run Time:	8.5 Minutes	Proc. Chnl. Descr.:	2998 PDA 240.0 nm (2998
Date Acquired:	10/4/2022 11:08:33 PM CST		
Date Processed:	10/27/2022 9:47:18 PM CST		

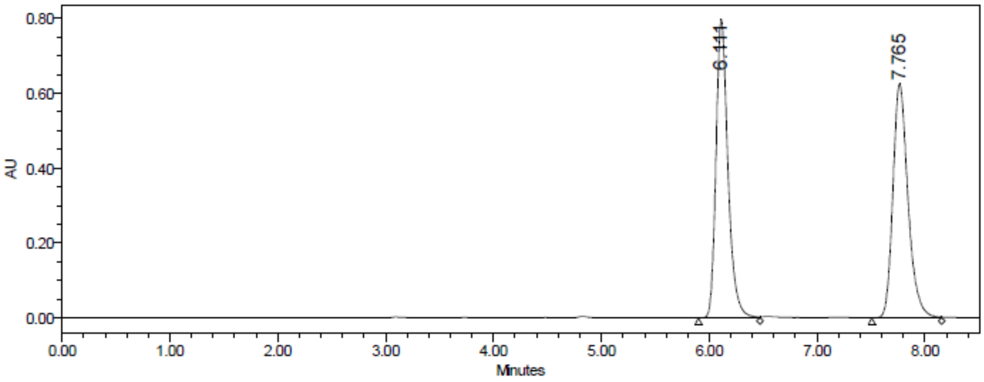

	RT	Area	% Area	Height
1	6.111	6157857	50.18	797968
2	7.765	6114479	49.82	625988

asy-**5ad**

SAMPLE INFORMATION

Sample Name:	zjy-9-273-5%-IA-asy	Acquired By:	System
Sample Type:	Unknown	Sample Set Name:	0
Vial:	54	Acq. Method Set:	5%quanbo
Injection #:	1	Processing Method:	1354685746
Injection Volume:	5.00 ul	Channel Name:	240.0nm
Run Time:	8.5 Minutes	Proc. Chnl. Descr.:	2998 PDA 240.0 nm (2998
Date Acquired:	10/4/2022 10:59:24 PM CST		
Date Processed:	10/27/2022 9:46:06 PM CST		

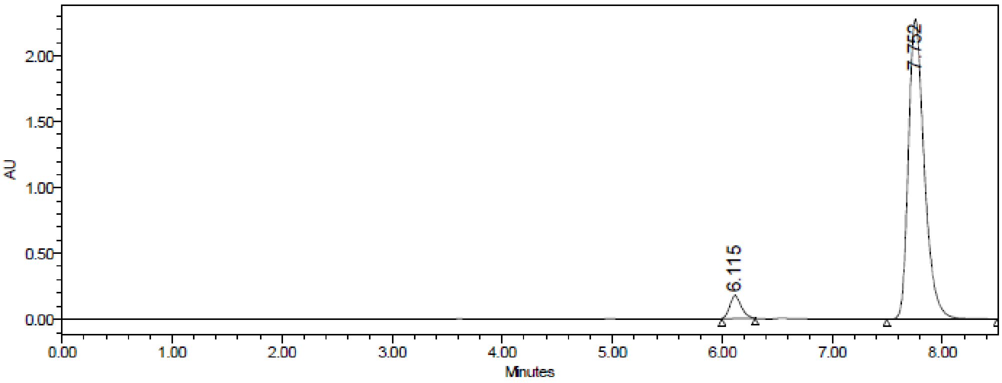

	RT	Area	% Area	Height
1	6.115	1261964	5.21	174036
2	7.752	22945201	94.79	2276149

rac-**5ae**

SAMPLE INFORMATION

Sample Name:	zjy-9-259-5%-IA-RAC	Acquired By:	System
Sample Type:	Unknown	Sample Set Name:	0
Vial:	49	Acq. Method Set:	5%quanbo
Injection #:	1	Processing Method:	1231
Injection Volume:	10.00 ul	Channel Name:	240.0nm
Run Time:	10.0 Minutes	Proc. Chnl. Descr.:	2998 PDA 240.0 nm (2998
Date Acquired:	9/22/2022 9:00:14 PM CST		
Date Processed:	10/27/2022 10:04:55 PM CST		

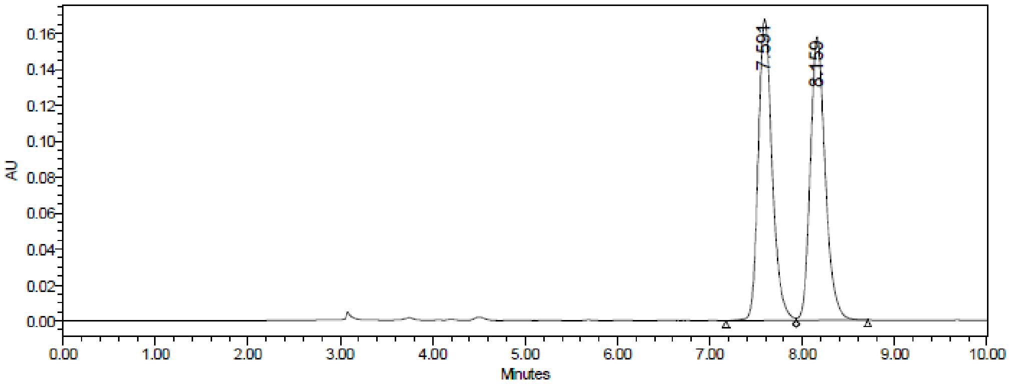

	RT	Area	% Area	Height
1	7.591	1763866	49.91	167543
2	8.159	1770491	50.09	157847

asy-**5ae**

SAMPLE INFORMATION

Sample Name:	zjy-9-259-5%-IA-asy	Acquired By:	System
Sample Type:	Unknown	Sample Set Name:	0
Vial:	70	Acq. Method Set:	5%quanbo
Injection #:	1	Processing Method:	LC PQ
Injection Volume:	10.00 ul	Channel Name:	254.0nm
Run Time:	10.0 Minutes	Proc. Chnl. Descr.:	2998 PDA 254.0 nm (2998
Date Acquired:	9/22/2022 6:56:59 PM CST		
Date Processed:	10/27/2022 10:03:47 PM CST		

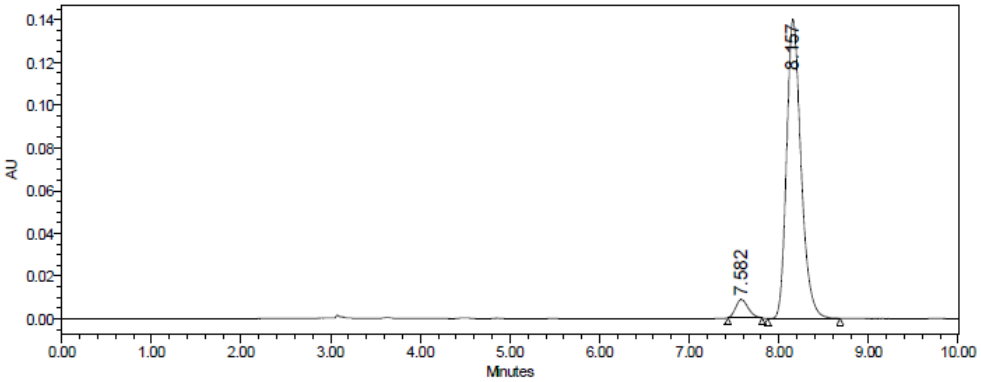

	RT	Area	% Area	Height
1	7.582	82564	5.01	8538
2	8.157	1564752	94.99	139908

rac-**5af**

SAMPLE INFORMATION

Sample Name:	zjy-9-260-5%-IC-RAC	Acquired By:	System
Sample Type:	Unknown	Sample Set Name:	0
Vial:	71	Acq. Method Set:	5%quanbo
Injection #:	1	Processing Method:	54165463
Injection Volume:	10.00 ul	Channel Name:	240.0nm
Run Time:	16.0 Minutes	Proc. Chnl. Descr.:	2998 PDA 240.0 nm (2998
Date Acquired:	9/22/2022 8:31:05 PM CST		
Date Processed:	10/27/2022 10:07:49 PM CST		

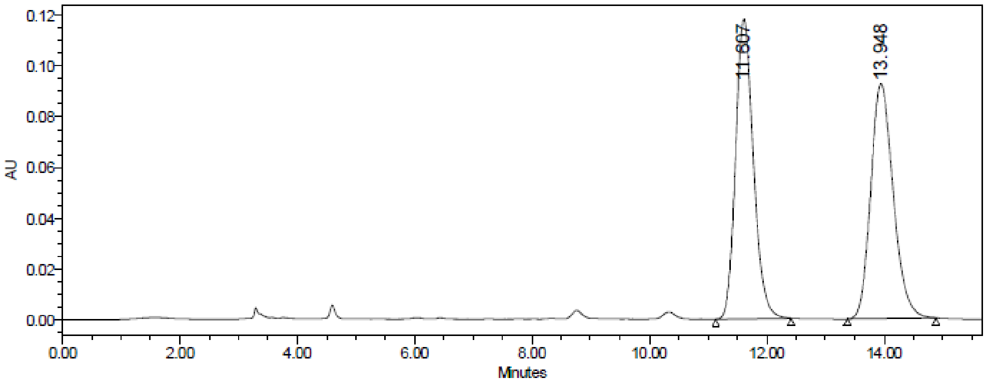

	RT	Area	% Area	Height
1	11.607	2489327	50.34	117902
2	13.948	2456107	49.66	92443

asy-**5af**

SAMPLE INFORMATION

Sample Name:	zjy-9-260-5%-IC-asy	Acquired By:	System
Sample Type:	Unknown	Sample Set Name:	0
Vial:	70	Acq. Method Set:	5%quanbo
Injection #:	1	Processing Method:	132
Injection Volume:	10.00 ul	Channel Name:	254.0nm
Run Time:	20.0 Minutes	Proc. Chnl. Descr.:	2998 PDA 254.0 nm (2998
Date Acquired:	9/22/2022 7:51:34 PM CST		
Date Processed:	10/27/2022 10:06:35 PM CST		

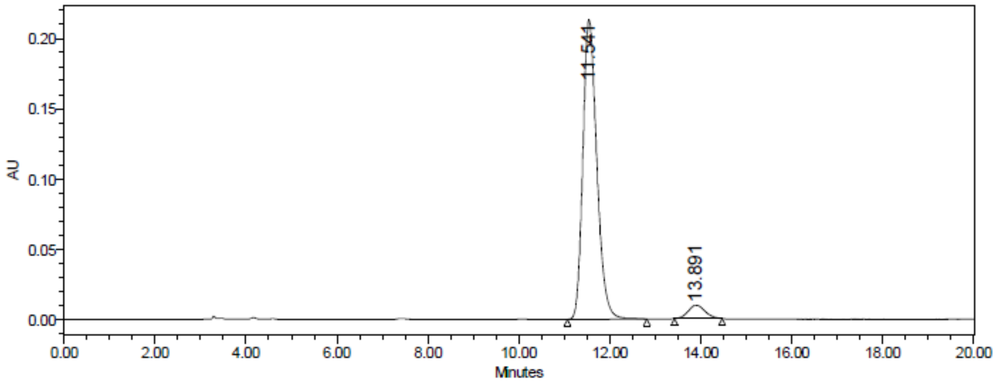

	RT	Area	% Area	Height
1	11.541	4498863	94.76	212963
2	13.891	248531	5.24	9669

rac-**5ag**

SAMPLE INFORMATION

Sample Name:	zjy-9-284-2%-IC-RAC	Acquired By:	System
Sample Type:	Unknown	Sample Set Name:	0
Vial:	58	Acq. Method Set:	2%quanbo
Injection #:	1	Processing Method:	1354685746
Injection Volume:	10.00 ul	Channel Name:	254.0nm
Run Time:	11.0 Minutes	Proc. Chnl. Descr.:	2998 PDA 254.0 nm (2998
Date Acquired:	10/5/2022 5:52:23 PM CST		
Date Processed:	10/27/2022 9:54:54 PM CST		

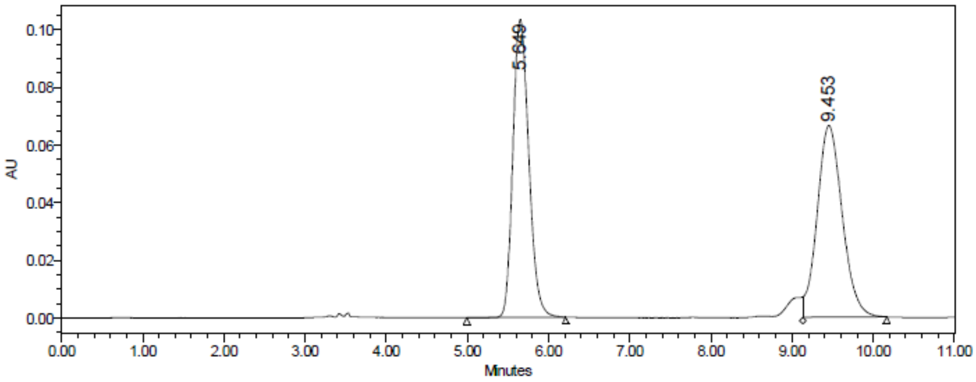

	RT	Area	% Area	Height
1	5.649	1407854	49.56	103213
2	9.453	1432924	50.44	66493

asy-**5ag**

SAMPLE INFORMATION

Sample Name:	zjy-9-284-2%-IC-asy	Acquired By:	System
Sample Type:	Unknown	Sample Set Name:	0
Vial:	57	Acq. Method Set:	2%quanbo
Injection #:	1	Processing Method:	132
Injection Volume:	10.00 ul	Channel Name:	254.0nm
Run Time:	11.0 Minutes	Proc. Chnl. Descr.:	2998 PDA 254.0 nm (2998
Date Acquired:	10/5/2022 5:40:42 PM CST		
Date Processed:	10/27/2022 9:52:53 PM CST		

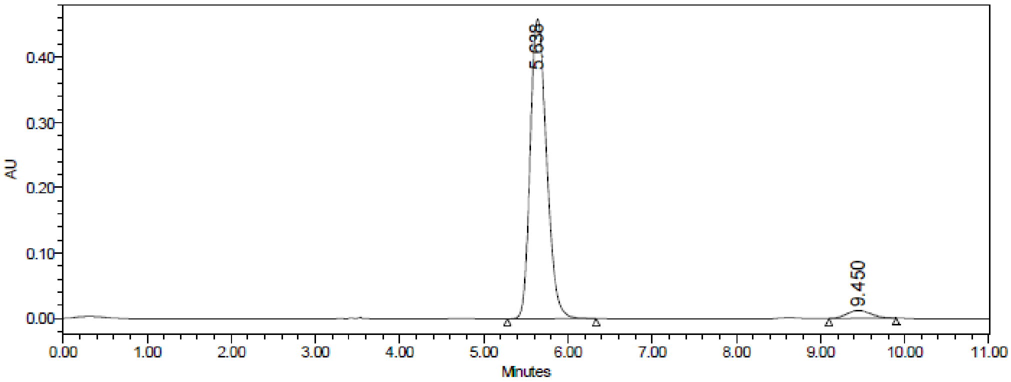

	RT	Area	% Area	Height
1	5.638	6198534	96.43	458303
2	9.450	229428	3.57	11864

rac-**5ah**

SAMPLE INFORMATION

Sample Name:	zjy-9-277-5%-IA-RAC	Acquired By:	System
Sample Type:	Unknown	Sample Set Name:	
Vial:	67	Acq. Method Set:	5%quanbo
Injection #:	1	Processing Method:	1354685746
Injection Volume:	5.00 ul	Channel Name:	240.0nm
Run Time:	60.0 Minutes	Proc. Chnl. Descr.:	2998 PDA 240.0 nm (2998
Date Acquired:	9/29/2022 5:07:04 PM CST		
Date Processed:	10/27/2022 10:11:40 PM CST		

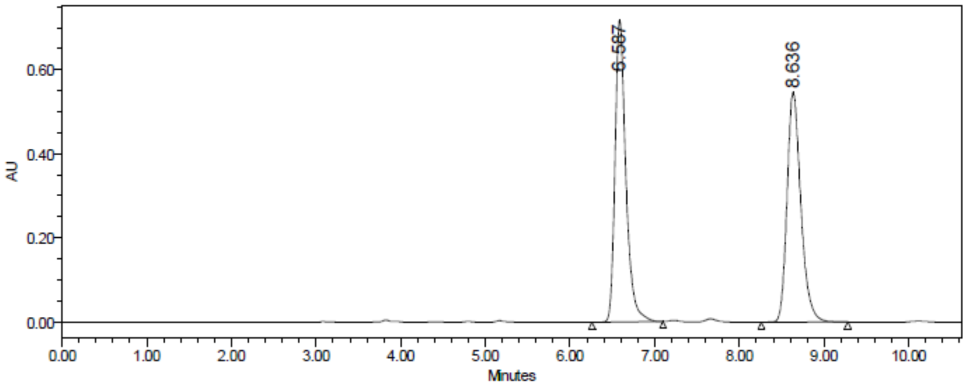

	RT	Area	% Area	Height
1	6.587	6375264	50.80	716460
2	8.636	6173602	49.20	545794

asy-**5ah**

SAMPLE INFORMATION

Sample Name:	zjy-9-283-5%-IA-asy	Acquired By:	System
Sample Type:	Unknown	Sample Set Name:	
Vial:	54	Acq. Method Set:	5%quanbo
Injection #:	1	Processing Method:	104
Injection Volume:	5.00 ul	Channel Name:	235.0nm
Run Time:	60.0 Minutes	Proc. Chnl. Descr.:	2998 PDA 235.0 nm (2998
Date Acquired:	9/29/2022 10:24:17 PM CST		
Date Processed:	10/27/2022 10:15:29 PM CST		

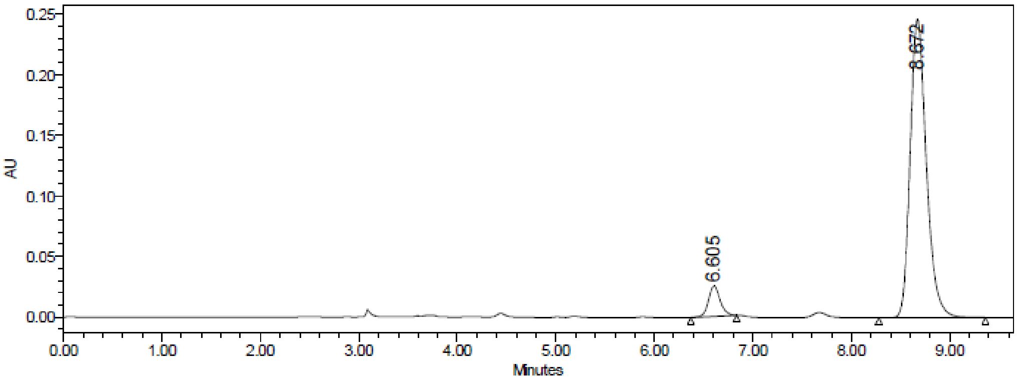

	RT	Area	% Area	Height
1	6.605	207102	6.90	25062
2	8.672	2792975	93.10	245988

rac-**6**

SAMPLE INFORMATION

Sample Name:	zjy-13-318-5%-AS-H-rac	Acquired By:	System
Sample Type:	Unknown	Sample Set Name:	
Vial:	67	Acq. Method Set:	5%quanbo
Injection #:	1	Processing Method:	54165463
Injection Volume:	10.00 ul	Channel Name:	268.0nm
Run Time:	60.0 Minutes	Proc. Chnl. Descr.:	2998 PDA 268.0 nm (2998
Date Acquired:	6/5/2024 4:31:04 PM CST		
Date Processed:	6/5/2024 5:07:26 PM CST		

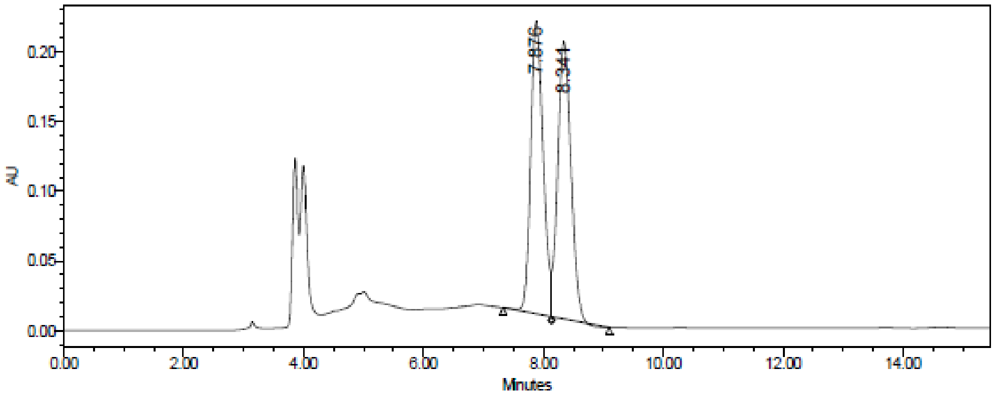

	RT	Area	% Area	Height
1	7.876	3041984	49.56	209417
2	8.341	3095674	50.44	198817

asy-**6**

SAMPLE INFORMATION

Sample Name:	zjy-13-318-5%-AS-H-asy	Acquired By:	System
Sample Type:	Unknown	Sample Set Name:	
Vial:	68	Acq. Method Set:	5%quanbo
Injection #:	1	Processing Method:	132
Injection Volume:	10.00 ul	Channel Name:	268.0nm
Run Time:	60.0 Minutes	Proc. Chnl. Descr.:	2998 PDA 268.0 nm (2998
Date Acquired:	6/5/2024 4:47:34 PM CST		
Date Processed:	6/5/2024 5:06:00 PM CST		

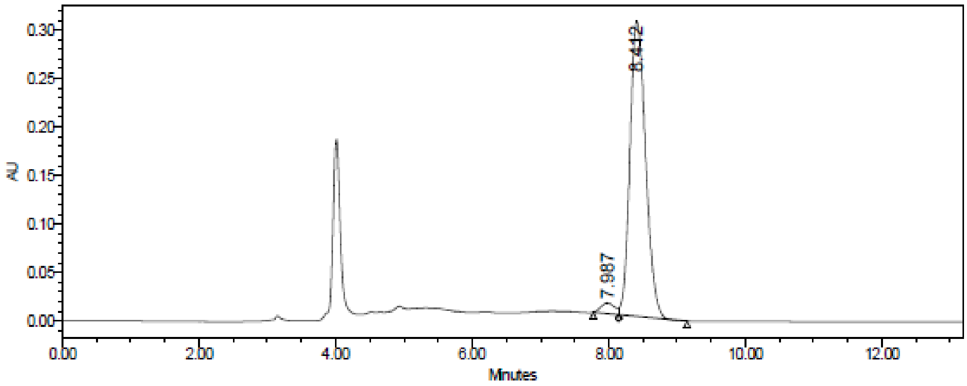

	RT	Area	% Area	Height
1	7.987	152724	3.06	11185
2	8.412	4830879	96.94	303960

rac-**7**

SAMPLE INFORMATION

Sample Name:	zjy-12-290-5%-OD-H-asy	Acquired By:	System
Sample Type:	Unknown	Sample Set Name:	
Vial:	68	Acq. Method Set:	5%quanbo
Injection #:	2	Processing Method:	1354685746
Injection Volume:	10.00 ul	Channel Name:	245.0nm
Run Time:	60.0 Minutes	Proc. Chnl. Descr.:	2998 PDA 245.0 nm (2998
Date Acquired:	6/7/2024 7:50:10 PM CST		
Date Processed:	6/7/2024 8:53:32 PM CST		

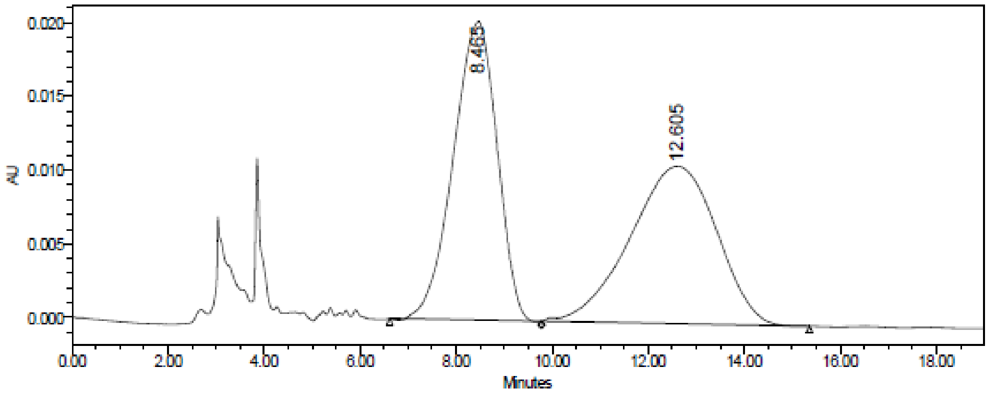

	RT	Area	% Area	Height
1	8.465	1287907	48.99	20251
2	12.605	1340914	51.01	10702

asy-**7**

SAMPLE INFORMATION

Sample Name:	zjy-13-316-5%-OD-H-asy	Acquired By:	System
Sample Type:	Unknown	Sample Set Name:	
Vial:	69	Acq. Method Set:	5%quanbo
Injection #:	1	Processing Method:	54165463
Injection Volume:	10.00 ul	Channel Name:	246.0nm
Run Time:	60.0 Minutes	Proc. Chnl. Descr.:	2998 PDA 246.0 nm (2998
Date Acquired:	6/7/2024 8:10:05 PM CST		
Date Processed:	6/7/2024 8:54:43 PM CST		

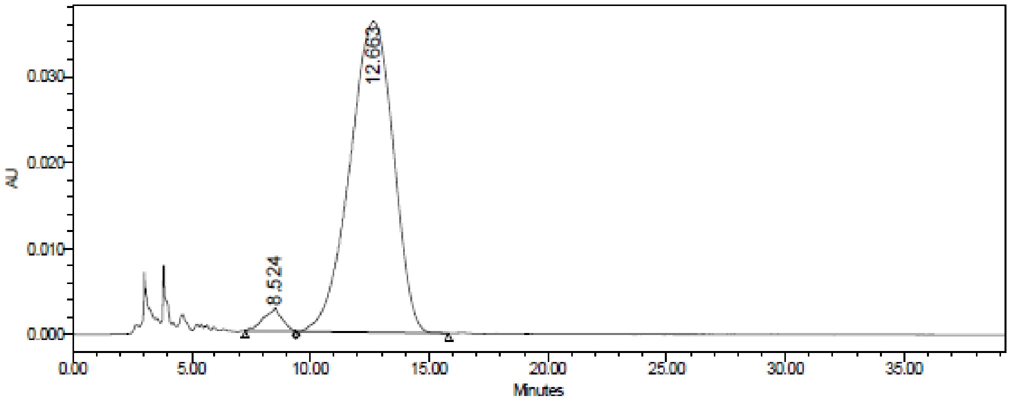

	RT	Area	% Area	Height
1	8.524	142949	3.01	2628
2	12.663	4607134	96.99	36132

rac-**8**

SAMPLE INFORMATION			
Sample Name:	zjy-13-311-15%-IE-rac	Acquired By:	System
Sample Type:	Unknown	Sample Set Name:	0
Vial:	70	Acq. Method Set:	15%quanbo
Injection #:	1	Processing Method:	ZJY 5 145
Injection Volume:	10.00 ul	Channel Name:	240.0nm
Run Time:	12.0 Minutes	Proc. Chnl. Descr.:	2998 PDA 240.0 nm (2998
Date Acquired:	6/5/2024 5:39:53 PM CST		
Date Processed:	6/5/2024 6:45:58 PM CST		

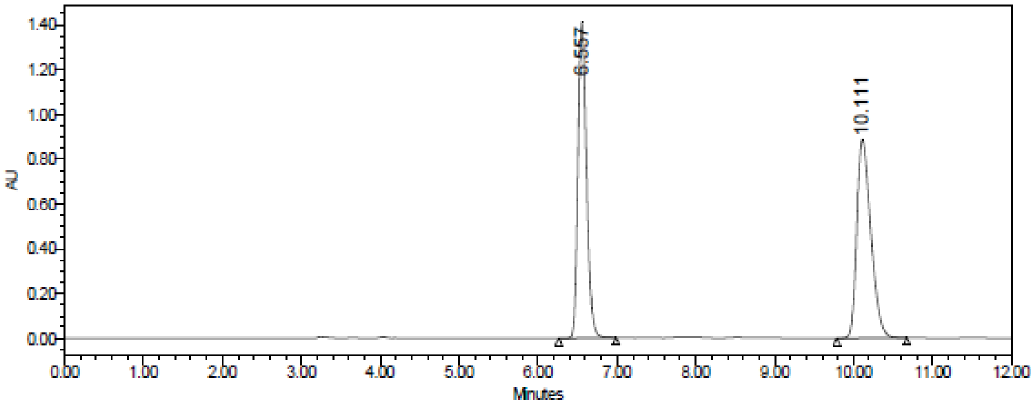

	RT	Area	% Area	Height
1	6.557	10565478	49.04	1410236
2	10.111	10977741	50.96	885300

asy-**8**

SAMPLE INFORMATION			
Sample Name:	zjy-13-311-15%-IE-rac	Acquired By:	System
Sample Type:	Unknown	Sample Set Name:	0
Vial:	71	Acq. Method Set:	15%quanbo
Injection #:	1	Processing Method:	132
Injection Volume:	10.00 ul	Channel Name:	240.0nm
Run Time:	12.0 Minutes	Proc. Chnl. Descr.:	2998 PDA 240.0 nm (2998
Date Acquired:	6/5/2024 5:52:35 PM CST		
Date Processed:	6/5/2024 6:43:43 PM CST		

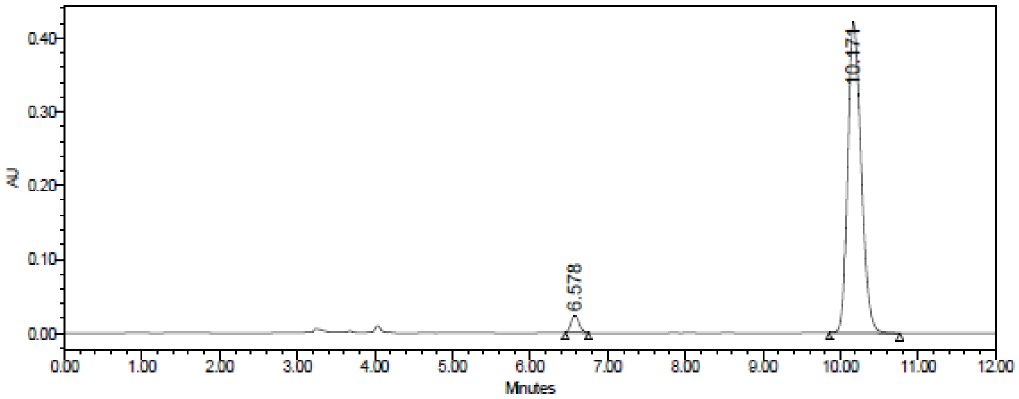

	RT	Area	% Area	Height
1	6.578	167825	3.21	23503
2	10.171	5054197	96.79	420780

rac-**9**

SAMPLE INFORMATION

Sample Name:	zjy-12-139-5%-AS-H-rac	Acquired By:	System
Sample Type:	Unknown	Sample Set Name:	
Vial:	66	Acq. Method Set:	5%quanbo
Injection #:	2	Processing Method:	1354685746
Injection Volume:	10.00 ul	Channel Name:	257.2nm
Run Time:	60.0 Minutes	Proc. Chnl. Descr.:	2998 PDA 257.2 nm (2998
Date Acquired:	6/7/2024 10:18:19 AM CST		
Date Processed:	6/7/2024 11:43:36 AM CST		

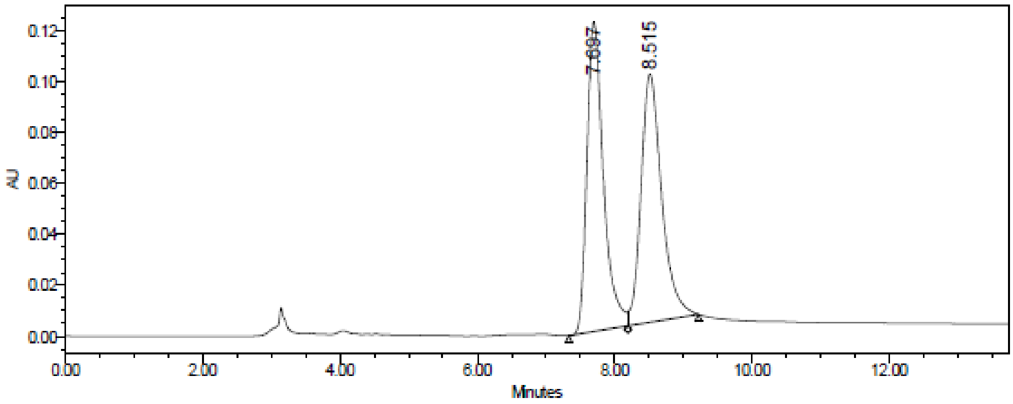

	RT	Area	% Area	Height
1	7.697	2021193	49.60	121348
2	8.515	2053424	50.40	97390

asy-**9**

SAMPLE INFORMATION

Sample Name:	zjy-13-317-5%-AS-H-asy	Acquired By:	System
Sample Type:	Unknown	Sample Set Name:	
Vial:	67	Acq. Method Set:	5%quanbo
Injection #:	1	Processing Method:	1354685746
Injection Volume:	10.00 ul	Channel Name:	272.8nm
Run Time:	60.0 Minutes	Proc. Chnl. Descr.:	2998 PDA 272.8 nm (2998
Date Acquired:	6/7/2024 11:25:49 AM CST		
Date Processed:	6/7/2024 11:45:14 AM CST		

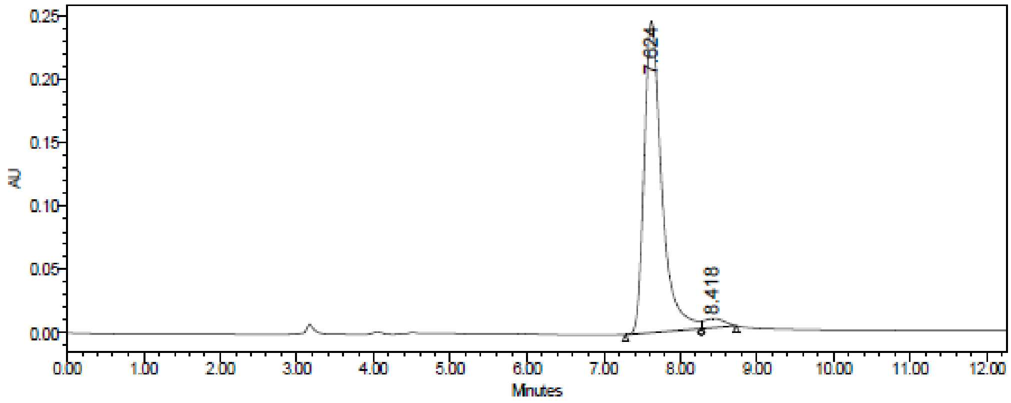

	RT	Area	% Area	Height
1	7.624	3941298	96.79	245544
2	8.418	130784	3.21	7168

rac-**10**

SAMPLE INFORMATION

Sample Name:	zjy-11-118-5%-IC-RAC	Acquired By:	System
Sample Type:	Unknown	Sample Set Name:	0
Vial:	56	Acq. Method Set:	5%quanbo
Injection #:	1	Processing Method:	54165463
Injection Volume:	10.00 ul	Channel Name:	240.0nm
Run Time:	25.0 Minutes	Proc. Chnl. Descr.:	2998 PDA 240.0 nm (2998
Date Acquired:	5/30/2023 4:38:33 PM CST		
Date Processed:	7/10/2023 10:45:30 AM CST		

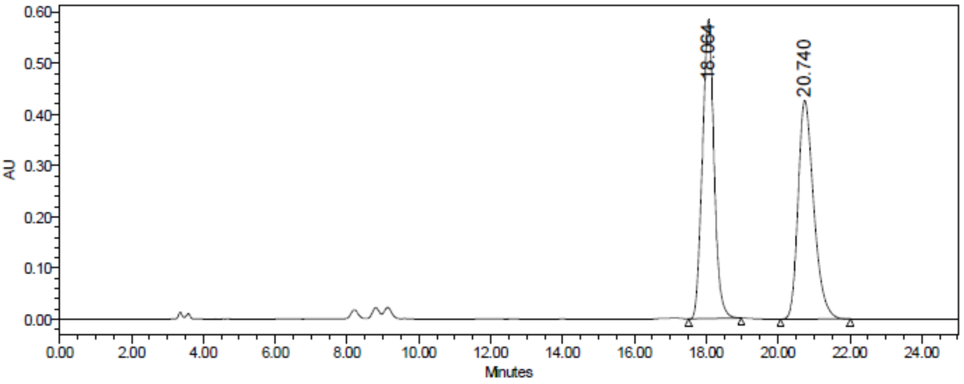

	RT	Area	% Area	Height
1	18.064	12979654	49.95	583732
2	20.740	13007765	50.05	427373

asy-**10**

SAMPLE INFORMATION

Sample Name:	zjy-11-122-5%-IC-asy	Acquired By:	System
Sample Type:	Unknown	Sample Set Name:	0
Vial:	57	Acq. Method Set:	5%quanbo
Injection #:	1	Processing Method:	54165463
Injection Volume:	10.00 ul	Channel Name:	240.0nm
Run Time:	25.0 Minutes	Proc. Chnl. Descr.:	2998 PDA 240.0 nm (2998
Date Acquired:	5/30/2023 5:04:13 PM CST		
Date Processed:	7/10/2023 10:47:00 AM CST		

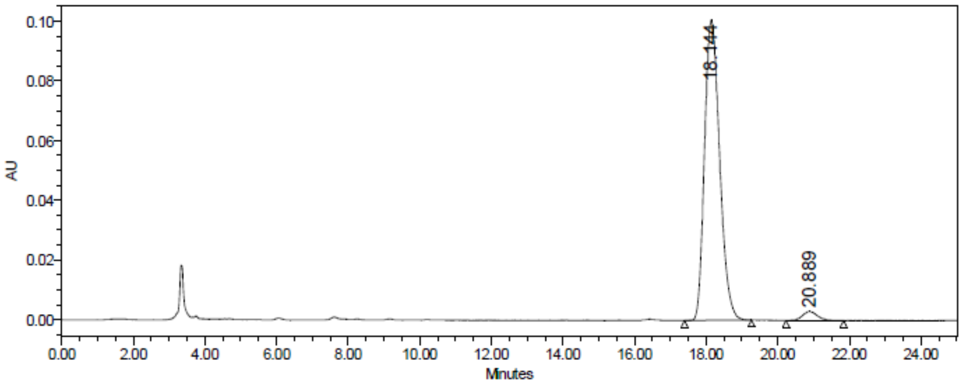

	RT	Area	% Area	Height
1	18.144	2914177	97.06	100529
2	20.889	88389	2.94	3045

rac-**11**

SAMPLE INFORMATION

Sample Name:	zjy-11-323-5%-IC-rac	Acquired By:	System
Sample Type:	Unknown	Sample Set Name:	0
Vial:	50	Acq. Method Set:	5%quanbo
Injection #:	1	Processing Method:	54165463
Injection Volume:	10.00 ul	Channel Name:	234.0nm
Run Time:	20.0 Minutes	Proc. Chnl. Descr.:	2998 PDA 234.0 nm (2998
Date Acquired:	7/18/2023 10:00:55 PM CST		
Date Processed:	7/20/2023 3:13:55 PM CST		

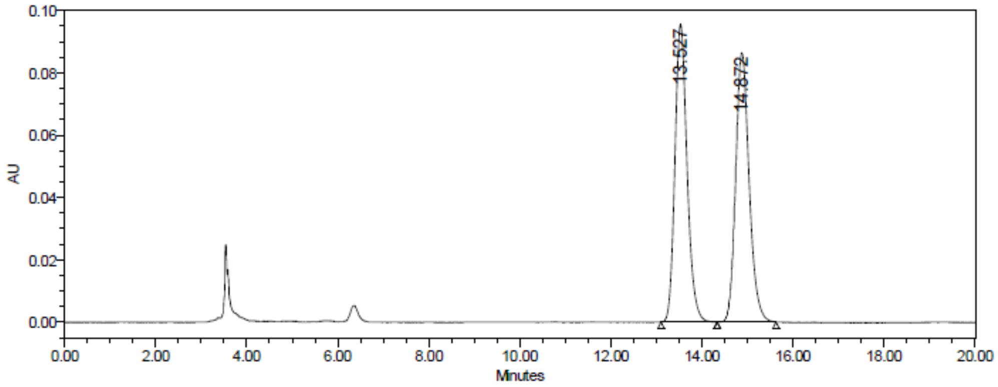

	RT	Area	% Area	Height
1	13.527	1776569	50.00	95418
2	14.872	1776838	50.00	86365

asy-**11**

SAMPLE INFORMATION

Sample Name:	zjy-11-323-5%-IC-asy	Acquired By:	System
Sample Type:	Unknown	Sample Set Name:	0
Vial:	49	Acq. Method Set:	5%quanbo
Injection #:	1	Processing Method:	1354685746
Injection Volume:	10.00 ul	Channel Name:	240.0nm
Run Time:	20.0 Minutes	Proc. Chnl. Descr.:	2998 PDA 240.0 nm (2998
Date Acquired:	7/18/2023 9:39:59 PM CST		
Date Processed:	7/20/2023 3:13:07 PM CST		

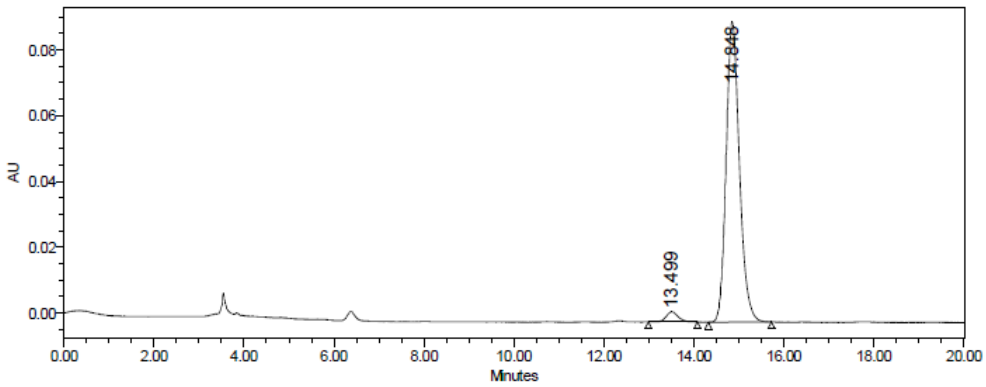

	RT	Area	% Area	Height
1	13.499	57167	2.97	3136
2	14.848	1868461	97.03	91190

rac-**12**

SAMPLE INFORMATION

Sample Name:	zjy-13-308-10%-IC-RAC	Acquired By:	System
Sample Type:	Unknown	Sample Set Name:	
Vial:	104	Acq. Method Set:	10%quanbo
Injection #:	1	Processing Method:	1354685746
Injection Volume:	10.00 ul	Channel Name:	234.0nm
Run Time:	60.0 Minutes	Proc. Chnl. Descr.:	2998 PDA 234.0 nm (2998
Date Acquired:	5/8/2024 3:22:51 PM CST		
Date Processed:	6/5/2024 3:35:52 PM CST		

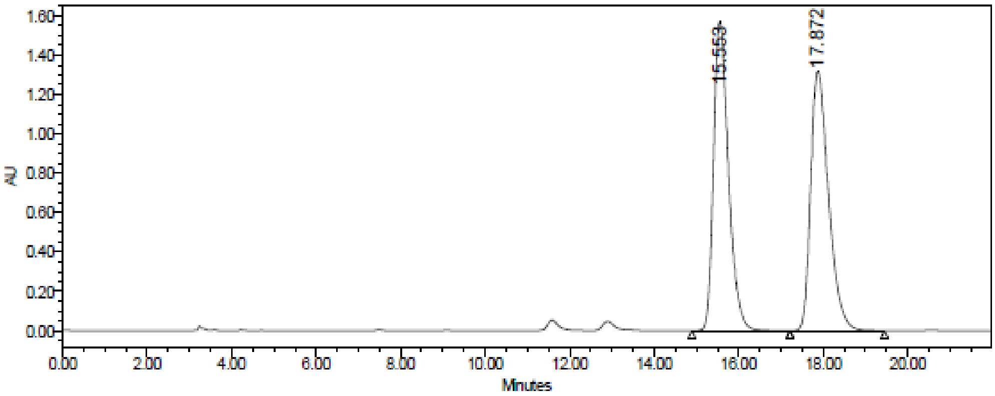

	RT	Area	% Area	Height
1	15.553	38491989	50.02	1572259
2	17.872	38464473	49.98	1319840

asy-**12**

SAMPLE INFORMATION

Sample Name:	zjy-13-308-10%-IC-asy	Acquired By:	System
Sample Type:	Unknown	Sample Set Name:	
Vial:	105	Acq. Method Set:	10%quanbo
Injection #:	1	Processing Method:	54165463
Injection Volume:	10.00 ul	Channel Name:	230.0nm
Run Time:	60.0 Minutes	Proc. Chnl. Descr.:	2998 PDA 230.0 nm (2998
Date Acquired:	5/8/2024 3:45:47 PM CST		
Date Processed:	6/5/2024 3:34:33 PM CST		

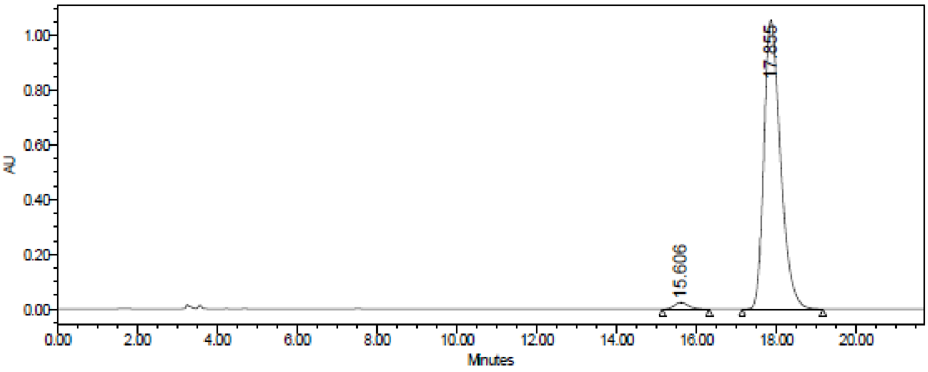

	RT	Area	% Area	Height
1	15.606	581803	1.88	24476
2	17.855	30371383	98.12	1055732

rac-**13**

SAMPLE INFORMATION

Sample Name:	zjy-14-61-15%-IC-rac	Acquired By:	System
Sample Type:	Unknown	Sample Set Name:	
Vial:	69	Acq. Method Set:	15%quanbo
Injection #:	2	Processing Method:	1354685746
Injection Volume:	10.00 ul	Channel Name:	240.0nm
Run Time:	60.0 Minutes	Proc. Chnl. Descr.:	2998 PDA 240.0 nm (2998
Date Acquired:	6/5/2024 7:08:38 PM CST		
Date Processed:	6/6/2024 9:29:01 PM CST		

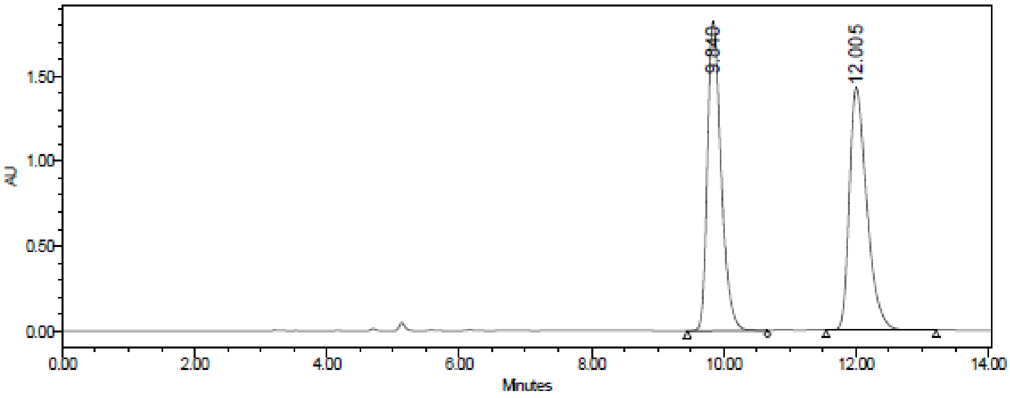

	RT	Area	% Area	Height
1	9.840	26202810	49.88	1822395
2	12.005	26330283	50.12	1429716

asy-**13**

SAMPLE INFORMATION

Sample Name:	zjy-14-61-15%-IC-asy	Acquired By:	System
Sample Type:	Unknown	Sample Set Name:	
Vial:	72	Acq. Method Set:	15%quanbo
Injection #:	1	Processing Method:	54165463
Injection Volume:	10.00 ul	Channel Name:	230.0nm
Run Time:	60.0 Minutes	Proc. Chnl. Descr.:	2998 PDA 230.0 nm (2998
Date Acquired:	6/5/2024 7:23:59 PM CST		
Date Processed:	6/6/2024 9:29:55 PM CST		

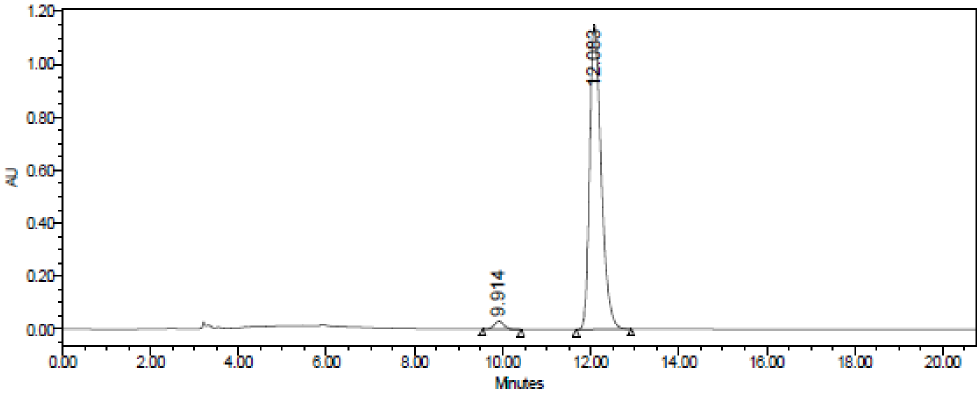

	RT	Area	% Area	Height
1	9.914	467564	2.19	30878
2	12.083	20926326	97.81	1149232

rac-**15**

SAMPLE INFORMATION

Sample Name:	zjy-14-64-10%-IC-rac	Acquired By:	System
Sample Type:	Unknown	Sample Set Name	0
Vial:	100	Acq. Method Set:	10% quan bo
Injection #:	1	Processing Method	zjy 5 122 asy0
Injection Volume:	5.00 ul	Channel Name:	240.1nm
Run Time:	25.0 Minutes	Proc. Chnl. Descr.:	2998 PDA 240.1 nm (2998
Date Acquired:	6/4/2024 18:29:49 CST		
Date Processed:	6/5/2024 15:49:07 CST		

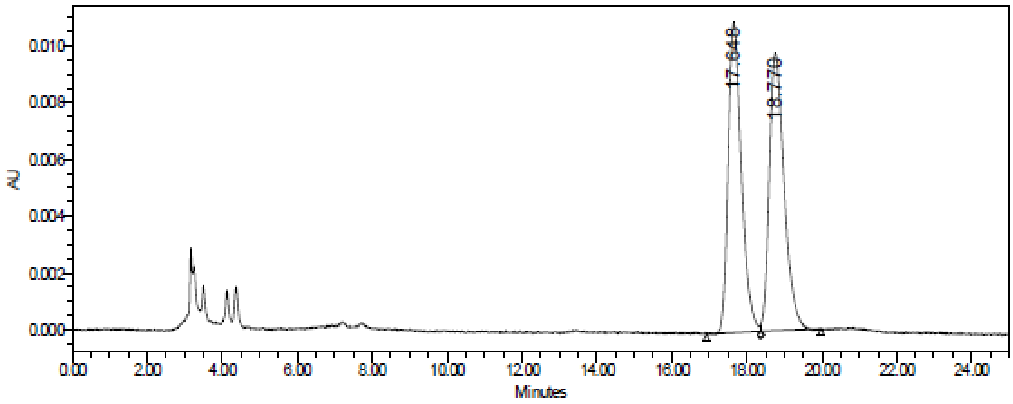

	RT	Area	% Area	Height
1	17.648	269593	50.00	10934
2	18.770	269620	50.00	9809

asy-**15**

SAMPLE INFORMATION

Sample Name:	zjy-14-64-10%-IC-asy	Acquired By:	System
Sample Type:	Unknown	Sample Set Name	0
Vial:	99	Acq. Method Set:	10% quan bo
Injection #:	1	Processing Method	44564
Injection Volume:	30.00 ul	Channel Name:	253.0nm
Run Time:	25.0 Minutes	Proc. Chnl. Descr.:	2998 PDA 253.0 nm (2998
Date Acquired:	6/4/2024 18:04:10 CST		
Date Processed:	6/5/2024 15:45:48 CST		

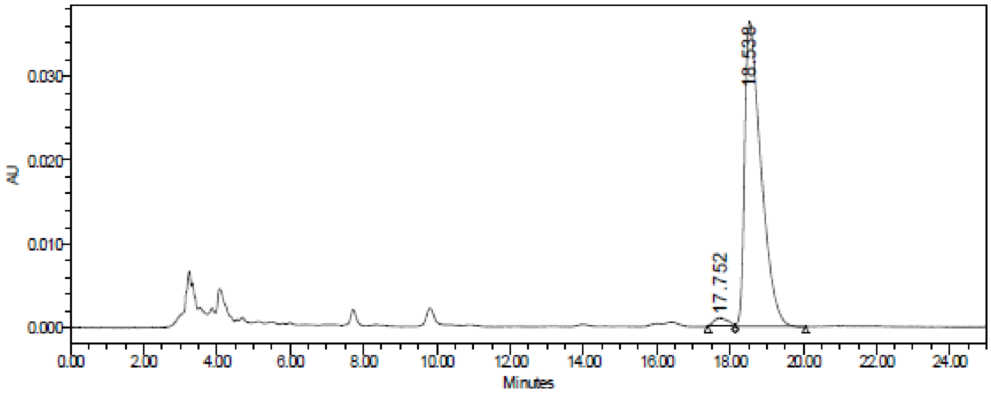

	RT	Area	% Area	Height
1	17.752	25113	2.18	931
2	18.538	1128694	97.82	36361

rac-**17**

SAMPLE INFORMATION

Sample Name:	zjy-15-169-10%-IE-RAC	Acquired By:	System
Sample Type:	Unknown	Sample Set Name:	
Vial:	59	Acq. Method Set:	10%quanbo
Injection #:	1	Processing Method:	54165463
Injection Volume:	5.00 ul	Channel Name:	330.0nm
Run Time:	60.0 Minutes	Proc. Chnl. Descr.:	2998 PDA 330.0 nm (2998
Date Acquired:	12/27/2024 7:30:48 PM CST		
Date Processed:	1/3/2025 10:25:13 PM CST		

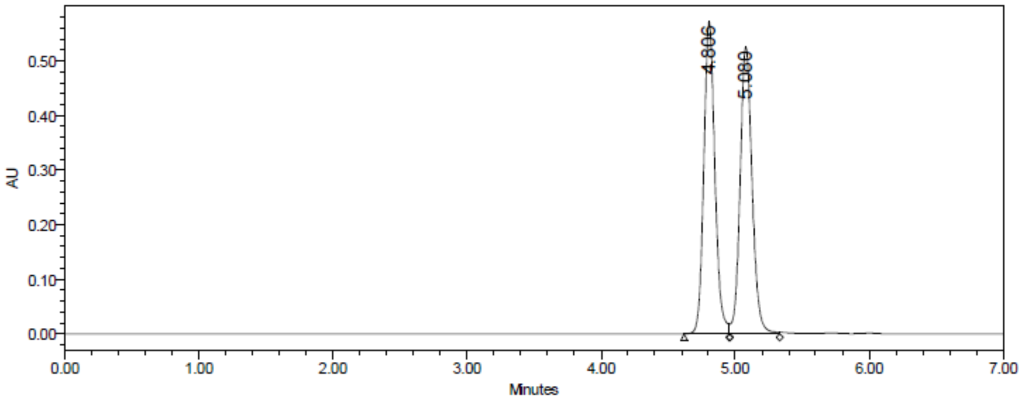

	RT	Area	% Area	Height
1	4.806	3283976	50.09	570923
2	5.080	3272470	49.91	524820

asy-**17**

SAMPLE INFORMATION

Sample Name:	zjy-15-154-10%-IE-asy	Acquired By:	System
Sample Type:	Unknown	Sample Set Name:	
Vial:	60	Acq. Method Set:	10%quanbo
Injection #:	2	Processing Method:	54165463
Injection Volume:	3.00 ul	Channel Name:	330.0nm
Run Time:	60.0 Minutes	Proc. Chnl. Descr.:	2998 PDA 330.0 nm (2998
Date Acquired:	12/27/2024 7:56:00 PM CST		
Date Processed:	1/3/2025 10:23:12 PM CST		

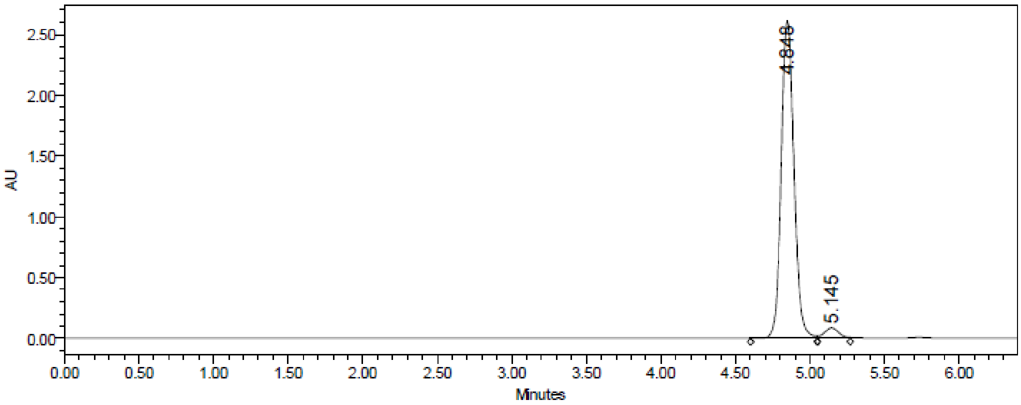

	RT	Area	% Area	Height
1	4.848	15454070	96.47	2607052
2	5.145	564944	3.53	86146
